# Characterization of the Human Oropharyngeal Microbiomes in SARS‐CoV‐2 Infection and Recovery Patients

**DOI:** 10.1002/advs.202102785

**Published:** 2021-08-22

**Authors:** Ming Gao, Haiyu Wang, Hong Luo, Ying Sun, Ling Wang, Suying Ding, Hongyan Ren, Jiaqi Gang, Benchen Rao, Shanshuo Liu, Xuemei Wang, Xinxin Gao, Mengyi Li, Yawen Zou, Chao Liu, Chengyu Yuan, Jiarui Sun, Guangying Cui, Zhigang Ren

**Affiliations:** ^1^ Department of Oncology The First Affiliated Hospital of Zhengzhou University Zhengzhou 450052 China; ^2^ Gene Hospital of Henan Province; Precision Medicine Center The First Affiliated Hospital of Zhengzhou University Zhengzhou 450052 China; ^3^ Department of Infectious Diseases The First Affiliated Hospital of Zhengzhou University Zhengzhou 450052 China; ^4^ Guangshan County People's Hospital Guangshan County Xinyang 465450 China; ^5^ Department of Clinical Laboratory Henan Provincial Chest Hospital Zhengzhou 450008 China; ^6^ Health Management Center The First Affiliated Hospital of Zhengzhou University Zhengzhou 450052 China; ^7^ Shanghai Mobio Biomedical Technology Co., Ltd. Shanghai 201111 China; ^8^ Xiuwu County People's Hospital Xiuwu County Jiaozuo 454350 China; ^9^ Department of Oncology Zhengzhou First People's Hospital Zhengzhou 450004 China

**Keywords:** COVID‐19, noninvasive biomarkers, oropharyngeal microbiome, SARS‐CoV‐2

## Abstract

Respiratory tract microbiome is closely related to respiratory tract infections, while characterization of oropharyngeal microbiome in recovered coronavirus disease 2019 (COVID‐19) patients is not studied. Herein, oropharyngeal swabs are collected from confirmed cases (CCs) with COVID‐19 (73 subjects), suspected cases (SCs) (36), confirmed cases who recovered (21), suspected cases who recovered (36), and healthy controls (Hs) (140) and then completed MiSeq sequencing. Oropharyngeal microbial *α*‐diversity is markedly reduced in CCs versus Hs. Opportunistic pathogens are increased, while butyrate‐producing genera are decreased in CCs versus Hs. The classifier based on eight optimal microbial markers is constructed through a random forest model and reached great diagnostic efficacy in both discovery and validation cohorts. Notably, the classifier successfully diagnosed SCs with positive IgG antibody as CCs and is demonstrated from the perspective of the microbiome. Importantly, several genera with significant differences gradually increase and decrease along with recovery from COVID‐19. Forty‐four oropharyngeal operational taxonomy units (OTUs) are closely correlated with 11 clinical indicators of SARS‐CoV‐2 infection and Hs based on Spearman correlation analysis. Together, this research is the first to characterize oropharyngeal microbiota in recovered COVID‐19 cases and suspected cases, to successfully construct and validate the diagnostic model for COVID‐19 and to depict the correlations between microbial OTUs and clinical indicators.

## Graphical abstract

This research is the first to characterize oropharyngeal microbiota in recovered COVID‐19 cases and suspected cases, constructs and validates the diagnostic model for COVID‐19, successfully diagnosing suspected cases with positive IgG antibody as confirmed cases and providing an auxiliary noninvasive diagnosis tool for COVID‐19, and depicts the correlations between microbial operational taxonomy unit and clinical indicators.

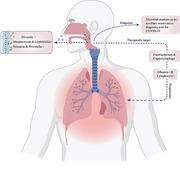

## 1 Introduction

Coronavirus disease 2019 (COVID‐19) is a respiratory infectious disease caused by severe acute respiratory syndrome coronavirus 2 (SARS‐CoV‐2).^[^ ^1^ ^]^ As of August 12, 2021, COVID‐19 has spread globally, causing more than 200 million infections and over 4 million deaths, and this number is still growing rapidly.^[^ ^2^ ^]^ Therefore, fast and early detection of potentially infected people, especially those with asymptomatic infections, is the focus of reducing the scope of transmission.^[^ ^3^ ^]^ Currently, the gold standard for diagnosing COVID‐19 is nucleic acid testing of upper respiratory tract specimens through reverse transcription‐polymerase chain reaction (RT‐PCR).^[^ ^4^ ^]^ However, the false‐negative rate of RT‐PCR is at least 20% for various reasons,^[^ ^5^ ^]^ such as virus mutation,^[^ ^6^ ^]^ sampling mistakes, and low virus titers. Thus, it is imperative to seek out a more accurate diagnostic method.

The respiratory tract, as the first barrier against the invasion of pathogens, is also a place for pathogens to replicate and move.^[^ ^7^ ^]^ SARS‐CoV‐2 can be detected in human upper respiratory tract samples, including nasopharyngeal and oropharyngeal specimens,^[^ ^8^ ^]^ and is spread through respiratory droplets. The virus enters the host cell by binding its spike protein to angiotensin‐converting enzyme 2 (ACE2) on human cells.^[^ ^9^ ^]^ TMPRSS2 helps to activate the spike protein on SARS‐CoV‐2.^[^ ^10^ ^]^ After entering host cells, the virus can not only cause inflammation and immune disorders but also destroy the upper respiratory tract microbial balance.

The human respiratory tract microbiome is closely related to respiratory tract infections,^[^ ^11^ ^]^ as an important part of the upper respiratory tract barrier. During viral infection, the disruption of the airway microbiome could impact the host innate immune response.^[^ ^12^ ^]^ At the same time, virus colonization and proliferation are also affected by the respiratory microbiome.^[^ ^13^ ^]^ Man et al^[^ ^14^ ^]^ found that the loss of microbial topography between oral and nasopharyngeal microbiota promotes respiratory infections in infants and young children. Further research reported that the nose/throat microbiota at the time of exposure is associated with susceptibility to influenza infection.^[^ ^15^ ^]^ A few current studies have characterized the nasopharyngeal,^[^ ^16^ ^]^ and oropharyngeal^[^ ^17^ ^]^ microbiota in SARS‐CoV‐2 infection, suggesting the function of the respiratory microbiome in the development of COVID‐19. Nevertheless, the oropharyngeal microbiome in recovered patients with SARS‐CoV‐2 infection has not been reported.

The concept of using microbial markers as a noninvasive diagnostic tool for diseases, including liver cirrhosis^[^ ^18^ ^]^ and type 2 diabetes (T2D),^[^ ^19^ ^]^ has been gradually formed. Our study previously reported that oral and gut microbial markers could be a potential noninvasive diagnostic tool for COVID‐19, achieving cross‐regional validation and successfully diagnosing suspected cases (SCs) with positive immunoglobulin G (IgG) as confirmed COVID‐19 cases.^[^ ^20^ ^]^ However, the diagnostic potential of applying oropharyngeal microbial markers in SARS‐CoV‐2 infection has not been assessed. In this study, we characterized the upper respiratory tract microbiota in SARS‐CoV‐2 infection and constructed a noninvasive microbial diagnostic model.

## 2 Experimental Section

### 2.1 Study Design

This research was designed in accordance with the principle of the prospective specimen collection and retrospective blinded evaluation^[^ ^21^ ^]^ and carried out following guidelines of the Helsinki Declaration and Rules of Good Clinical Practice. All patients and healthy controls (Hs) signed informed consent before sample collection. Our protocol was ratified by the Ethics Committee of the First Affiliated Hospital of Zhengzhou University (2020‐ KY‐055) and Guangshan County People's Hospital (2020‐001).

There were five study groups. We enrolled confirmed cases (CCs) and SCs from Xinyang City, Henan, China, from February to March 2020. Patients were diagnosed according to the “COVID‐19 diagnosis and treatment program trial V.5 (or V.6) guidelines” issued by the National Health Commission of the People's Republic of China^[^ ^22^ ^]^ (Supporting Information). All enrolled patients received a standard treatment plan. After strict screening, 73 CCs and 36 SCs were included for further analysis. Among them, 21 CCs who recovered (CCRs) and 36 SCs who recovered (SCRs) were collected specimens twice. The first time when they were just admitted to the hospital (CC and SC), and the second time when they left the quarantine area (CCR and SCR). Then, we selected 140 Hs to matched the age, sex, and body mass index (BMI) of confirmed cases (**Table** **1**). These Hs were sampled from volunteers in the Physical Examination Center in the First Affiliated Hospital of Zhengzhou University. The details of the exclusion, inclusion, and diagnostic criteria are shown in the Supporting Information.

**Table 1:** Demographics and clinical characteristics of subjects in discovery and validation cohorts

Clinical indicators	Discovery Cohort (*n* = 142)		*p*‐value	Validation cohort (*n* = 71)		*p*‐value
	Healthy controls (*n* = 94)	Confirmed cases (*n* = 48)		Healthy controls (*n* = 46)	Confirmed cases (*n* = 25)	
Age (years)	45.94 ± 9.11	46.98 ± 14.83	0.228	46.37 ± 9.80	49.08 ± 13.12	0.328
Sex (female/male)	57/37	29/19	0.980	29/17	16/9	0.936
Body mass index (BMI)	25.25 ± 3.61	24.39 ± 3.38	0.172	24.68 ± 3.92	24.50 ± 3.00	0.923
Comorbidities		9(18.8%)			7(28%)	
Confirmed cases or exposure to Wuhan		43(89.6%)			18(72%)	
Symptoms at admission						
Fever		28(58.3%)			18(72%)	
Cough		23(47.92%)			8(32%)	
Sputum		6(12.5%)			2(8%)	
Headache		3(6.25%)			3(12%)	
Fatigue		3(6.25%)			9 (36%)	
Diarrhea		1(2.1%)			0(0%)	
Dyspnea		1(2.1%)			2(8%)	
Laboratory results						
Red blood cells (10^12^ L^−1^)	4.82 ± 0.47	4.65 ± 0.58	0.077	4.70 ± 0.44	4.67 ± 0.78	0.75
White blood cells (10^9^ L^−1^)	5.76 ± 1.31	5.74 ± 2.21	0.342	5.92 ± 1.63	5.10 ± 2.02	0.038
Neutrophils (10^9^ L^−1^)	3.38 ± 0.98	4.70 ± 2.33	0.001	3.54 ± 1.17	4.00 ± 1.73	0.247
Lymphocytes (10^9^ L^−1^)	1.85 ± 0.45	1.88 ± 2.12	0.002	1.86 ± 0.61	1.25 ± 0.45	<0.0001
Blood Platelet (10^9^ L^−1^)	245.65 ± 67.04	198.21 ± 78.99	<0.0001	245.09 ± 56.70	200.68 ± 65.31	0.004
Hemoglobin (g L^−1^)	146.88 ± 17.96	139.88 ± 33.99	<0.0001	144.80 ± 17.73	141.36 ± 20.61	0.323
Alanine aminotransferase (U L^−1^)	21.48 ± 9.91	26.72 ± 19.11	0.406	23.78 ± 13.33	26.23 ± 13.90	0.332
Aspartate aminotransferase (U L^−1^)	21.46 ± 4.95	26.41 ± 13.20	0.022	21.91 ± 5.50	25.22 ± 10.56	0.086
Albumin (g L^−1^)	48.06 ± 2.82	43.16 ± 5.35	<0.0001	47.79 ± 2.78	41.47 ± 8.38	<0.0001
Total bilirubin (µmol L^−1^)	11.58 ± 5.00	14.71 ± 11.32	0.560	11.31 ± 3.92	11.42 ± 6.75	0.222
Serum creatinine (µmol L^−1^)	68.03 ± 14.13	93.33 ± 163.34	0.712	77.37 ± 14.85	69.28 ± 18.67	0.315

We presented continuous variables as the means (standard deviations) and categorical variables as percentages. Differences between subjects with SARS‐CoV‐2 infection (*n* = 48, *n* = 25) and healthy controls (*n* = 94, *n* = 46) were carried out by using Student's *t*‐test for normally distributed continuous variables, the Wilcoxon rank‐sum test for non‐normally distributed continuous variables, and the chi‐square test or Fisher's exact test for categorical variables. Statistical significance was defined by *p* < 0.05 (two‐tailed). Comorbidities included high blood pressure, chronic obstructive pulmonary disease, diabetes, malignant tumor, cardiovascular disease, and chronic liver disease (Table S1, Supporting Information).

### 2.2 Oropharyngeal Specimen Collection and DNA Extraction

Each enrolled subject provided an oropharyngeal sample. Before taking samples, participants used saline to gargle twice. The posterior pharynx and the tonsils on both sides were scraped with a pharyngeal swab by a professional operator. Then, the swab head was straightway immersed in a tube containing 2–3 mL virus preservation solution. All samples were immediately inactivated at 56 °C for at least 30 min, and then they were stored in a freezer at −80 °C. According to the requirements of the “COVID‐19 Prevention and Control Plan (Fifth Edition),”^[^ ^23^ ^]^ the collection, transport, stockpile and testing of specimens were temporarily managed under the second category of highly pathogenic microorganisms.

Oropharyngeal microbial DNA was extracted using a Qiagen Mini Kit (Qiagen, Hilden, Germany) following the manufacturer's instructions. The DNA samples were quantified by a Qubit 2.0 Fluorometer (Invitrogen, Carlsbad, CA, USA), and molecular size was estimated using agarose gel electrophoresis. Microbial DNA samples were diluted to 10 ng µL^−1^ for further analysis.

### 2.3 PCR Amplification and 16S rRNA Gene Sequencing

PCR amplification and DNA library construction was performed as described before. The 16S rRNA V3‐V4 region was sequenced using the Illumina MiSeq platform in the Shanghai Mobio Biomedical Technology, China. All raw Illumina read data were deposited in the European Bioinformatics Institute European Nucleotide Archive database (PRJNA739539). The amplified reads were processed by us. The details are shown in the Supporting Information.

### 2.4 Operational Taxonomy Unit (OTU) Clustering and Taxonomy Annotation

Quantity‐controlled sequences obtained from all samples by abundance were chosen to identify representative sequences through the UPARSE pipeline.^[^ ^24^ ^]^ The identity threshold was set at 0.97 to cluster gene sequences into OTUs and annotated the phylogenetic affiliation of each OTU through RDP classifier V.2.6^[^ ^25^ ^]^ according to the developer's documents (http://rdp.cme.msu.edu/classifier/class_help.jsp#conf).

### 2.5 Bioinformatic Analysis of 16S rRNA Sequencing

Rarefaction curves and species accumulation curves were used to make certain that sample size and sequencing depth achieved saturation in this research. Bacterial *α*‐diversity was presented by the Shannon index and Simpson index using the R program package “vegan.” Principal coordinates analysis (PCoA) and nonmetric multidimensional scaling (NMDS) were generated by the R package (http://www.R‐project.org/) to visualize the microbial space between samples. The heatmap for the pivotal variables was constructed through Heatmap Builder. Compositional differences from phylum level to genus level were conducted by Wilcoxon rank‐sum test.

Oropharyngeal microbial variations between different groups were analyzed through the linear discriminant analysis (LDA) effect size (LEfSe) method (http://huttenhower.sph.harvard.edu/lefse/)e/).^[^ ^26^ ^]^ We used LEfSe (Kruskal–Wallis rank‐sum test, *p* < 0.05) to identify the significantly different taxa. Then, we used LDA to evaluate variation at the taxonomic level, and the cutoff value was set as LDA score (log 10) = 2 or 2.5.^[^ ^27^ ^]^

The discovery and validation OTU frequency profile was generated through mapping reads from the corresponding phase. Then, the OTU biomarkers by the Wilcoxon rank‐sum test were selected for further analysis. Five times of fivefold cross‐validation were conducted to construct a diagnostic model (R 3.4.1, randomForest 4.6–12 package). The possibility of disease (POD) was evaluated by the identified optimal set of OTUs. The receiver operating characteristic (ROC) curve was constructed by using the “R 3.3.0, pROC package.” Microbial biomarkers were considered successful if the area under the ROC curve (AUC) was greater than 0.7. The detailed process of bioinformatic analysis was described previously (Method section in the Supporting Information).^[^ ^28^ ^]^

### 2.6 Detection of IgG Against SARS‐CoV‐2

The levels of serum IgG antibodies were tested against SARS‐CoV‐2 in recovered subjects based on chemiluminescence immunoassay kits (Shenzhen Mairui Biomedical Electronics Co., Ltd., Guangdong) according to the manufacturer's instructions and under stringent biosafety conditions. The positive judgment value was 10 U mL^−1^ in this kit (>10 U mL^−1^ was positive, and <10 U mL^−1^ was negative). The antibody levels were presented with log 2 (value).

### 2.7 Statistical Analysis

Continuous variables with the form of means (standard deviations) or median (interquartile ranges) were presented, and categorical variables with the form of percentages. To compare differences between CCs (*n* = 48 and *n* = 25) and Hs (*n* = 94 and *n* = 46), Student's *t*‐test was performed for normally distributed continuous variables, Wilcoxon rank‐sum test was used for non‐normally distributed continuous variables, and the *χ* ^2^‐test or Fisher's exact test was used for categorical variables. To compare differences among three groups, one‐way analysis of variance was used for normally distributed continuous variables, and the Kruskal–Wallis test was used for non‐normally distributed continuous variables. Statistical analyses were performed using SPSS V.20.0 for Windows (SPSS, Chicago, Illinois, USA). *p* < 0.05 (two‐tailed) was defined as statistical significance, without *α* adjustment and postanalysis.

## 3 Results

### 3.1 Study Design and Characteristics of the Participants

**Figure 1:**
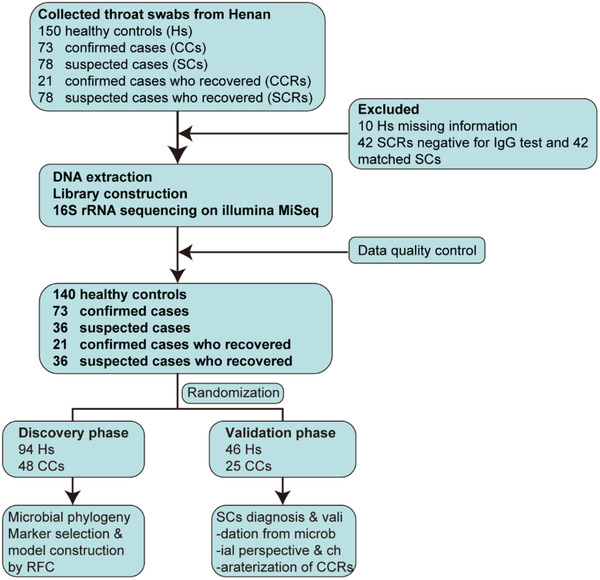
Study design. A total of 400 oropharyngeal specimens from Central China were collected. After screening, 306 samples were used for further sequencing using 16S rRNA MiSeq, including 140 Hs, 73 CCs, 36 SCs, 21 CCRs, and 36 SCRs. Hs, healthy controls; CCs, confirmed cases; SCs, suspected cases; CCRs, confirmed cases who recovered; SCRs, suspected cases who recovered; RFC, random forest classifier.

Together, 400 oropharyngeal specimens from Central China were prospectively collected. After a rigorous process of inclusion and exclusion process, 306 oropharyngeal specimens were sequenced by 16S rRNA MiSeq (**Figure** **1**). Among them, 140 H samples and 73 CC specimens were randomly divided into the discovery cohort (94 H and 48 CC) and the validation cohort (46 H and 25 CC), respectively. We characterized oropharyngeal microbiomes, identified the key microbial markers, and constructed a COVID‐19 classifier in the discovery cohort. In the validation phase, we used 46 H samples and 25 CC samples to verify the efficacy of the COVID‐19 diagnostic classifier. We further applied this model to diagnose suspected cases with positive serum IgG antibodies to verify the potential of the COVID‐19 diagnostic classifier.

The clinical characteristics of confirmed cases and Hs in the discovery and validation cohorts are shown in Table 1 and Table S1 (Supporting Information). The mean age of the COVID‐19 patients in the discovery phase was 46.98 years, with a 29:19 ratio of males to females. The most common signs or symptoms of COVID‐19 patients at admission were fever and cough. We further analyzed the routine blood and biochemical indicators of CCs and Hs and mainly investigated eleven indicators in the blood tests. We found that blood platelets (*p* < 0.01) and albumin (*p* < 0.0001) were decreased in the confirmed cases versus Hs.

### 3.2 Oropharyngeal Microbial Diversity in COVID‐19

**Figure 2:**
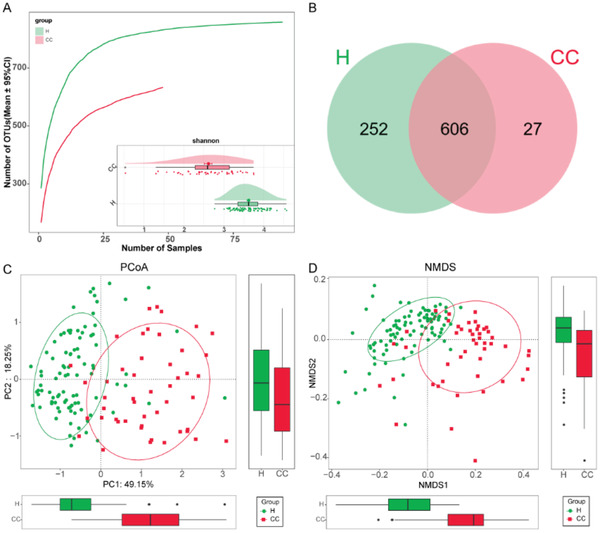
Oropharyngeal microbial diversity of confirmed cases and healthy controls in the discovery cohort. A) Rarefaction analysis showed as the number of samples raised, the number of OTUs approached saturation in CCs (*n* = 48) and Hs (*n* = 94). The number of OTUs in CCs was reduced versus Hs. As evaluated by the Shannon index, oropharyngeal microbial diversity was remarkedly reduced in CCs versus Hs (*p* < 0.001). B) A Venn diagram displaying the overlaps between groups showed that 606 of 885 OTUs were shared in CC and H groups, while 27 of 885 OTUs were unique to the CC group. The PCoA C) and NMDS D) based on OTU distribution showed the oropharyngeal taxonomic composition was conspicuously different between the two groups. COVID‐19, coronavirus disease 2019; Hs, healthy controls; CCs, confirmed cases; OTUs, operational taxonomy units; PCoA, principal coordinate analysis; NMDS, nonmetric multidimensional scaling; centerline, median; box limits, upper and lower quartiles; error bars, 95% CI.

In the discovery cohort, we carried out rarefaction analysis (**Figure** **2**a) and results showed that OTU richness in the H group and the CC group approached stable, and it was significantly decreased in the CCs versus Hs. The oropharyngeal microbial diversity was compared between CCs and Hs by using Shannon index and Simpson index for alpha diversity and PCoA and NMDS analysis for beta diversity. The results showed that oropharyngeal microbial alpha diversity in the CCs was remarkedly reduced versus in Hs (*p* < 0.001) (Figure 2a; Figure S1a,b and Table S2, Supporting Information). The beta diversity results showed that there was a significant distinction in oropharyngeal microbial community distribution between the two groups, indicating that COVID‐19 oropharyngeal microbiota dysbiosis occurred (Figure 2c,d). A Venn diagram revealed that 606 of 885 OTUs were shared between both groups, while 27 OTUs were sole to CCs (Figure 2b).

### 3.3 Phylogenetic Profiles of Oropharyngeal Microbial Communities in COVID‐19

**Figure 3:**
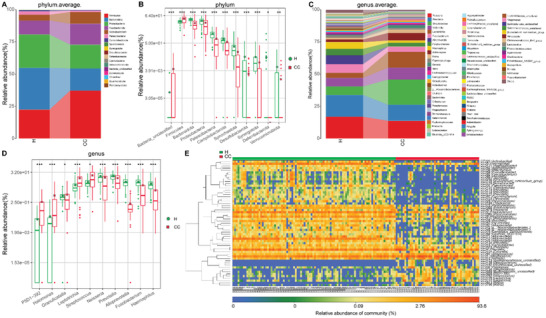
Phylogenetic profiles of the oropharyngeal microbiome in CCs (*n* = 48) and Hs (*n* = 94). A) Average compositions and relative abundance of the bacterial community in both groups at the phylum level. B) Two phyla were remarkedly increased, while 8 phyla were remarkedly reduced in CCs versus Hs. C) Average compositions and relative abundance of the bacterial community in both groups at the genus level. D) Compared with Hs, five genera were remarkedly decreased, while five genera were remarkedly increased in CCs. E) Heatmap showed the relative abundances of differential OTUs for each sample in both groups. ^*^ *p* < 0.05, ^**^ *p* < 0.01, ^***^ *p* < 0.001. COVID‐19, coronavirus disease 2019; Hs, healthy controls; CCs, confirmed cases; OTUs, operational taxonomy units; centerline, median; box limits, upper and lower quartiles; circle or square symbol, mean; error bars, 95% CI.

We further identified the microbial composition and alterations of the oropharyngeal microbiome in the CCs and Hs in the discovery phase. We found that the phyla *Firmicutes*, *Bacteroidota*, *Proteobacteria*, and *Fusobacteriota* together accounted for 85% of sequences on average and were the four leading bacteria in the CCs and Hs (**Figure** **3**a). The average composition and relative abundance of the oropharyngeal microbiome at the genus level were exhibited in Figure 3c (Table S3, Supporting Information).

Next, we performed differential expression analysis of bacteria between CCs and Hs through Wilcoxon rank‐sum test (with *p* < 0.05). At the phylum level, the phyla *Firmicutes* and *Bacteria_unclassified* were increased in the CCs (*p* < 0.001) compared with Hs, while 9 phyla, including *Bacteroidota*, *Proteobacteria*, and *Patescibacteria*, were decreased (*p* < 0.05) (Figure 3b). At the genus level, 62 differentiating genera were identified between CCs and Hs. Among them, 9 genera, including *Streptococcus*, *Leptotrichia*, and *Granulicatella*, were significantly increased (*p* < 0.05), while 53 genera, including *Neisseria*, *Prevotella*, and *Alloprevotella*, were significantly depleted in the CCs (*p* < 0.05) (Figure 3d; Table S4, Supporting Information). Then, 57 OTUs that were significantly increased or decreased in the CCs versus Hs were selected and presented in the heatmap with abundance (Figure 3e; Table S5, Supporting Information). In summary, our results revealed a unique oropharyngeal microbiota composition in COVID‐19 patients, characterized by the enrichment of lipopolysaccharide‐producing bacteria such as *Leptotrichia* and opportunistic pathogens such as *Granulicatella* and the depletion of butyrate‐producing bacterial families such as *Bifidobacterium*, *Fusobacterium*, and *Porphyromonas*.

Moreover, we performed LEfSe analysis to select specific bacterial taxa associated with COVID‐19. The cladogram, representing oropharyngeal microbial structure and their predominant bacteria, displayed the most differences in taxa between CCs and Hs (Figure S2a; Table S6, Supporting Information). In addition, we predicted the microbial community function profiles through 16S rRNA marker gene sequences according to the Kyoto Encyclopedia of Genes and Genomes (KEGG) orthology (KO) and KEGG pathway/module profile. A total of 13 enriched pathways were identified with the most significant differences between CCs and Hs, were displayed in Figure S2b and Table S7 (Supporting Information). Among them, 6 functions, such as biosynthesis of ansamycins and alanine metabolism, were remarkably increased, while 7 functions, such as lipopolysaccharide biosynthesis and photosynthesis, were remarkably decreased in the CCs.

### 3.4 Diagnostic Potential of the Oropharyngeal Microbial Classifier for COVID‐19

**Figure 4:**
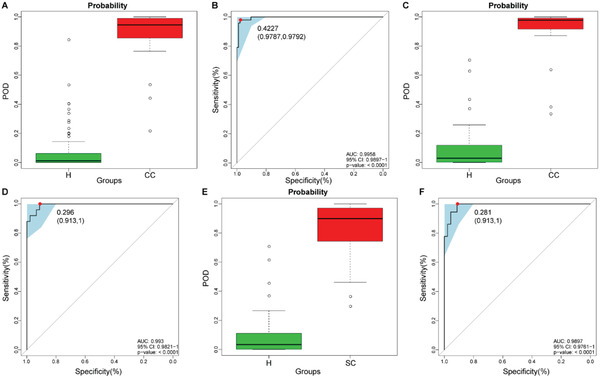
Diagnostic potential of the oropharyngeal microbial classifier for COVID‐19. The POD value was markedly increased in CCs (*n* = 48 and *n* = 25) versus Hs (*n* = 94 and *n* = 46) and achieved good diagnostic efficacy in the discovery cohort A,B) (*p* < 0.0001) and the validation cohort C&D) (*p* < 0.0001). Compared with Hs (*n* = 46), the POD value was significantly increased in SCs (*n* = 36) E), achieving an AUC value of 0.9897 F) (*p* < 0.0001). COVID‐19, coronavirus disease 2019; Hs, healthy controls; CCs, confirmed cases; SCs, suspected cases; OTUs, operational taxonomy units; POD, probability of disease; AUC, area under the curve. Centerline, median; box limits, upper and lower quartiles; circle or square symbol, mean; error bars, 95% CI.

To demonstrate the diagnostic potential of the oropharyngeal microbial classifier for SARS‐CoV‐2 infection, the fivefold cross‐validation random forest model was constructed in the discovery phase (94 Hs and 48 CCs). The results showed that 8 OTU markers, which could accurately differentiate CCs and Hs, were identified as the best microbial marker set (Figure S1c,d, Supporting Information). Then, we computed the probability of disease (POD) index for discovery phase and validation phase by using an 8 OTU set. The POD value was markedly higher in the CCs compared with Hs in the discovery phase (*p* < 0.05) (**Figure** **4**a; Table S8, Supporting Information), with an AUC of 99.58% (95% CI 98.97% to 100%, *p* < 0.0001) (Figure 4b). The above results justified that oropharyngeal microbial marker could effectively diagnose confirmed cases with COVID‐19.

Moreover, in the validation phase, 46 Hs and 25 CCs were included to further certify the diagnostic efficacy of the classifier for COVID‐19. POD index was markedly raised in CCs compared with Hs (*p* < 0.05) (Figure 4c; Table S9, Supporting Information), with an AUC value of 99.3% (95% CI 98.21% to 100%) between both groups (*p* < 0.0001) (Figure 4d). The above data validated the powerful diagnostic efficacy of oropharyngeal microbial markers for COVID‐19.

### 3.5 Oropharyngeal Microbial Characterization Among CCs, SCs, and Hs

**Figure 5:**
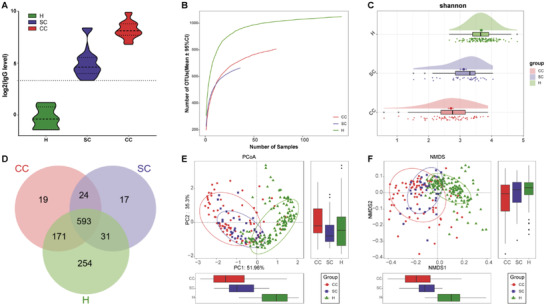
Oropharyngeal microbial diversity among CCs, SCs, and Hs. A) Levels of antibodies against SARS‐CoV‐2 in CCs (*n* = 21), SCs (*n* = 36), and Hs (*n* = 6) after recovery. The positive judgment value was 10 U mL^−1^ in the kit (>10 U mL^−1^ was positive, and <10 U mL^−1^ was negative). The antibody levels in the figure were computed as log_2_(value). B) Rarefaction analysis displayed that as the number of samples increased, the number of OTUs approached stable in CCs (*n* = 73), SCs (*n* = 36) and Hs (*n* = 140). Compared with the Hs, the number of OTUs was reduced in CCs and SCs. C) As evaluated by the Shannon index, the oropharyngeal microbial diversity of CCs and SCs was lower than that of Hs (all *p* < 0.001). D) A Venn diagram revealed that 593 of 1109 OTUs were shared in the CC, SC, and H groups, while 254 OTUs were unique to Hs. The PCoA E) and NMDS F) exhibited that the oropharyngeal microbial communities in the CCs and SCs were similar but significantly different from those in the Hs. CCs, confirmed cases; SCs, suspected cases; Hs, healthy controls; SARS‐CoV‐2, severe acute respiratory syndrome coronavirus 2; OTUs, operational taxonomic units; PCoA, principal coordinate analysis; NMDS, nonmetric multidimensional scaling; centerline, median; box limits, upper and lower quartiles; error bars, 95% CI.

To reduce the false‐negative rate of RT‐PCR for COVID‐19 and identify potentially infected people, we collected oropharyngeal samples from 78 SCs on admission and before discharge. After excluding patients with negative serum IgG antibody for SARS‐CoV‐2, samples from 36 SCs and their matched SCRs were included for further analysis. We applied the microbial marker classifier to 36 selected SCs and found that the average POD index was markedly increased in SCs versus Hs (*p* < 0.05) (Figure 4e; Table S10, Supporting Information), with an AUC value of 98.97% (95% CI 97.61% to 100%) between SCs and Hs (Figure 4f). These results indicated that this oropharyngeal microbial classifier could increase the efficacy of the diagnosis of SARS‐CoV‐2 infection and may be used as an auxiliary noninvasive diagnosis tool. In addition, we detected the serum IgG antibody levels among 6 Hs, 21 CCs, and 36 SCs (**Figure** **5**a; Table S11, Supporting Information). The data showed that antibody levels in SCs were higher than those in Hs but lower than those in CCs.

**Figure 6:**
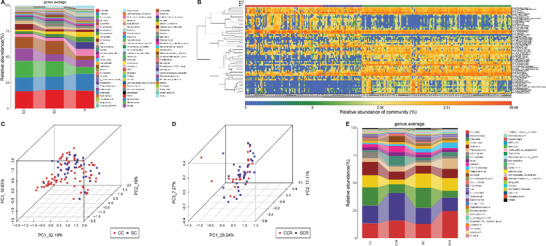
Oropharyngeal microbial composition and abundance among CCs, SCs, and Hs. A) Average compositions and relative abundance of the bacterial community in the three groups at the genus level. B) Heatmap of the relative abundances of differential OTUs for each sample in the three groups. The PCoA showed that there was no significant difference in the oropharyngeal microbiome distribution between CCs (*n* = 73) and SCs (*n* = 36) C) or between CCRs (*n* = 21) and SCRs (*n* = 36) D). E) Average compositions and relative abundance of the bacterial community in the four groups at the genus level. OTUs, operational taxonomic units; CCs, confirmed cases; CCRs, confirmed cases who recovered; SCs, suspected cases; SCRs suspected cases who recovered; Hs, healthy controls; PCoA, principal coordinate analysis.

To further demonstrate the possibility of diagnosing selected SCs as CCs by an oropharyngeal microbial model, we analyzed the microbial characterization among 73 CCs, 36 SCs, and 140 Hs. The rarefaction analysis showed that OTU richness in the CC, SC, and H groups approached saturation (Figure 5b). The alpha diversity of CCs and SCs was lower than that of Hs (*p* < 0.05) (Figure 5c; Figure S3a and Table S12, Supporting Information). PCoA and NMDS analysis were applied to compare the beta diversity of the microbial communities, and the microbial distributions of CCs and SCs were similar, but both were significantly different from those of Hs (Figure 5e,f). A Venn diagram showed that 593 OTUs were shared among three groups, and 254 OTUs were unique in Hs, but only 19 and 17 OTUs were unique to CCs and SCs, respectively (Figure 5d). The average abundances and compositions for oropharyngeal microbiome at the OTU, genus, and phylum levels are presented in **Figure** **6**a,b and Figure S3b in the Supporting Information (Tables S13 and S14, Supporting Information), the abundances and composition of oropharyngeal microbial in SCs were similar to those in CCs, but both were markedly different from those in Hs.

Additionally, we next compared the microbial differences between CCs and SCs, as well as CCRs and SCRs, and no obvious difference was observed in the oropharyngeal microbial distribution using PCoA analysis (Figure 6c,d). In addition, the oropharyngeal microbiome variation between CCs and CCRs was roughly the same as the oropharyngeal microbiome variation between SCs and SCRs (Figure 6e; Table S15, Supporting Information).

In total, these data showed that the oropharyngeal microbial characterization of SCs was similar to that of CCs from the perspective of the microbiome. This coincides with our speculation that the same disease has similar microbial characteristics.^[^ ^20^ ^]^ Furthermore, these results could also demonstrate the feasibility of using our model to diagnose SCs with positive IgG antibodies for SARS‐CoV‐2 as CCs.

### 3.6 Association between Oropharyngeal Microbial and the Recovery of Patients with SARS‐CoV‐2 Infection

**Figure 7:**
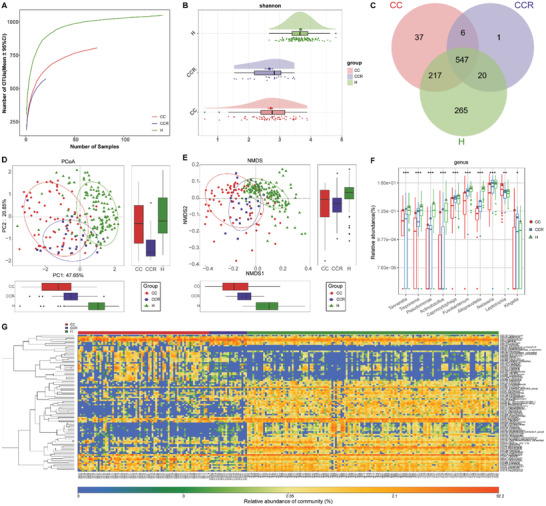
Association between oropharyngeal microbial and the recovery of patients with SARS‐CoV‐2 infection. A) Rarefaction analysis revealed that as the number of samples increased, the number of OTUs approached stable in CCs (*n* = 73), CCRs (*n* = 21) and Hs (*n* = 140). Compared with the Hs, the number of OTUs in CCs and CCRs was depleted. B) As evaluated by the Shannon index, oropharyngeal microbial diversity in the CCRs was similar to that in the CCs (*p* > 0.05) but remarkedly reduced compared with that in the Hs (all *p* < 0.001). C) A Venn diagram exhibited that 547 of 1093 OTUs were shared in the CC, CCR and H groups, while 265 OTUs were unique to Hs. The PCoA D) and NMDS E) showed that the oropharyngeal microbiota in the CCRs were different from those in the CCs and Hs. F) The relative abundances of 8 genera gradually enriched and were remarkedly different among three groups, while the abundances of 2 genera gradually reduced and were remarkedly different among three groups along with recovery of COVID‐19. G) Heatmap for the relative abundances of differential OTUs for each sample in the three groups. ^*^ *p* < 0.05, ^**^ *p* < 0.01, ^***^ *p* < 0.001. OTUs, operational taxonomic units; CCs, confirmed cases; CCRs, confirmed cases who recovered; Hs, healthy controls; PCoA, principal coordinate analysis; NMDS, nonmetric multidimensional scaling; COVID‐19, coronavirus disease 2019. Centerline, median; box limits, upper and lower quartiles; circle or square or triangle symbol, mean; error bars, 95% CI.

To identify the potential microbiome involved in recovery of SARS‐CoV‐2 infection, 73 CCs, 21 CCRs, and 140 Hs were used for further analysis. The OTU richness in the CC, SC, and H groups approached saturation by using rarefaction analysis (**Figure** **7**a; Figure S4a, Supporting Information). The alpha diversity of CCs and CCRs was similar (*p* > 0.05), but both were lower than that of Hs (*p* < 0.001) (Figure 7b; Figure S4b and Table S16, Supporting Information). The PCoA and NMDS analysis exhibited the microbial community distribution in CCRs was significantly different from that in CCs and Hs (Figure 7d,e). A Venn diagram displayed 547 OTUs were shared among the three groups, and 265 OTUs were unique to Hs (Figure 7c).

The average oropharyngeal microbial composition and relative abundance in the CC, CCR, H groups at the genus level are presented in Figure S4c in the Supporting Information (Table S17, Supporting Information). The genera *Prevotella*, *Neisseria*, *Streptococcus*, *Veillonella*, and *Leptotrichia* together accounted for 50% of the sequences on average and were the five predominant bacteria among three groups. The most prominent bacterium in CC and Hs was *Prevotella* but was *Neisseria* in CCRs, indicating a unique feature associated with CCRs. The difference analysis was performed at the phylum and genus levels (Figure 7f; Figure S4d and Table S18, Supporting Information). The results showed that along with recovery from SARS‐CoV‐2 infection, the abundances of eight genera including *Fusobacterium*, *Capnocytophaga*, and *Actinobacillus*, gradually raised (*p* < 0.001), while two genera, *Leptotrichia* and *Kingella*, persistently reduced (*p* < 0.05), indicating that these bacteria may be involved in SARS‐CoV‐2 infection recovery. The heatmap displayed that the relative abundance of most different OTUs gradually increased or decreased as the individuals recovered (Figure 7g; Table S19, Supporting Information). Then, we performed LEfSe analysis to find the greatest differences in taxa between CCs, CCRs, and Hs, and the results are displayed in Figure S5a in the Supporting Information (Table S20, Supporting Information). A total of 13 enriched pathways with the most significant differences among the three groups were identified (Figure S5b and Table S21, Supporting Information). Among them, seven functions, such as beta‐alanine metabolism and glycosaminoglycan degradation, were remarkably increased in the Hs; four functions, such as fatty acid biosynthesis, were increased in the CCRs; and two functions, such as D alanine metabolism, were increased in the CCs.

### 3.7 Correlation between the Oropharyngeal Microbiota and Clinical Indicators

**Figure 8:**
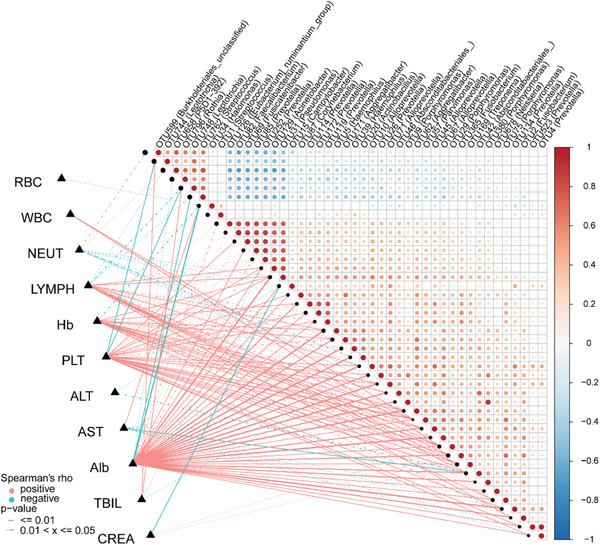
Correlation between the oropharyngeal microbiota and clinical indicators. Heatmap showing the partial Spearman's correlation coefficients among 44 distinctive oropharyngeal OTUs and 11 clinical indicators between CCs (*n* = 48) and Hs (*n* = 94). OTUs, operational taxonomic units; CCs, confirmed cases; Hs, healthy controls; RBC, red blood cell; WBC, white blood cell; NEUT, neutrophils; LYMPH, lymphocytes; Hb, hemoglobin; PLT, platelets; ALT, alanine aminotransferase; AST, aspartate aminotransferase; Alb, albumin; TBIL, total bilirubin; CREA, creatinine. The red line represents a positive correlation, and the blue line represents a negative correlation.

We further analyzed the association between 44 oropharyngeal OTUs and 11 clinical indicators of confirmed cases and healthy controls based on Spearman correlation analysis (**Figure** **8**; Table S22, Supporting Information). We found that five clinical indicators (RBCs, WBCs, ALT, TBIL, and CREA) were only closely related, with no more than five OTUs. Among them, RBCs were only negatively correlated with OTU34 (*Halomonas*). Six clinical indicators (NEUT, LYMPH, Hb, PLT, AST, ALB) were closely related to more than five OTUs. Among them, ALB was negatively related with 3 OTUs, including OTU762 (*Streptococcus*) and OTU34 (*Halomonas*), but positively correlated with 36 OTUs, including OTU2 (*Fusobacterium*) and OTU4 (*Prevotella*). LYMPH was negatively correlated with 3 OTUs, including OTU658 (P5D1‐392), OTU748 (*Leptotrichia*), and OTU762 (*Streptococcus*), and positively related with 25 OTUs, including OTU20 (*Actinobacillus*) and OTU57 (*Parvimonas*). Taken together, these results suggest that alterations of the oropharyngeal microbiome may impact disease severity and progression.

## 4 Discussion

Our study is the first to report the characteristics of the oropharyngeal microbiome in a large sample of patients with SARS‐CoV‐2 infection, suspected cases, and recovered patients using 16S rRNA sequencing. We found that oropharyngeal microbial diversity was dramatically decreased in patients with SARS‐CoV‐2 infection, which was similar to a previous study,^[^ ^17^ ^]^ indicating the presence of oropharyngeal microbial dysbiosis. Moreover, the composition and abundance of the oropharyngeal microbiome were altered after SARS‐CoV‐2 infection. Compared with Hs, the genera *Leptotrichia*, *Granulicatella*, and *Streptococcus*, belonging to opportunistic pathogens, were increased, while *Fusobacterium* and *Prevotella*, belonging to butyrate‐producing bacteria, were reduced in CCs. A previous study reported that respiratory viral infections increase susceptibility to secondary bacterial infection of the lungs.^[^ ^29^ ^]^ Lu et al. further revealed that *Leptotrichia* and *Granulicatella* were overrepresented in H7N9 patients with secondary bacterial lung infection (SBLI).^[^ ^30^ ^]^ Hence, we speculated that the overgrowth of opportunistic pathogens may facilitate the progression of COVID‐19 by promoting secondary infections in the patient. Oropharyngeal microbiome dysbiosis may induce the translocation of opportunistic pathogens,^[^ ^17^ ^]^ causing SBLI and even other organ injuries in COVID‐19 patients, while oropharyngeal microbial markers may be used as biomarkers to identify and even predict SBLI. Butyric acid, a short‐chain fatty acid, is the main nutrient of human intestinal epithelial cells^[^ ^31^ ^]^ and is mainly derived from the fermentation of dietary fiber by the intestinal microbiome in the human colon.^[^ ^32^ ^]^ Butyric acid plays an important role in inhibiting cancer, anti‐inflammatory, protecting the intestinal mucosa, and promoting nutrient absorption and growth.^[^ ^33^ ^]^ Therefore, the reduction of butyric acid‐producing bacteria may lead to the progression of pneumonia by promoting inflammation and exacerbating the destruction of the intestinal environment.

Several researches have illustrated that upper tract microbiome is closely correlated with viral diseases and could be used as a noninvasive diagnostic tool for various diseases. Surette et al.^[^ ^34^ ^]^ found that the oral and nasopharyngeal microbiomes changed significantly one month before respiratory tract infection occurred, manifested by the appearance of oral microbiota in the nasopharynx. Man et al.^[^ ^35^ ^]^ suggested that combining bacteria and viruses in the nasopharyngeal microbiome and the characteristics of the host can more accurately distinguish children with lower respiratory tract infections from healthy children, with an AUC of 0.92. Our previous study^[^ ^20^ ^]^ identified alterations in the oral microbiomes of COVID‐19 and constructed a diagnostic model based on a set of eight oral OTUs, achieving cross‐regional validation with an AUC of 0.9211. Ma et al.^[^ ^3^ ^]^ demonstrated that based on 20 oropharyngeal genera associated with COVID‐19, oropharyngeal microbial markers achieved high classification power. However, the sample size in Ma's study was small, with only 31 COVID‐19 patients, and the diagnostic efficacy of the model was not verified. In our present study, we identified a set of eight optimal OTUs to construct the diagnostic model in the discovery cohort (48 CCs and 94 Hs), which achieved power potential for distinguishing COVID‐19 from healty controls. More importantly, the model achieved good diagnostic efficacy with an AUC of 0.993 in the validation cohort. We first applied the model to diagnose SCs with positivity for IgG antibody and successfully diagnosed them as having COVID‐19, indicating that oropharyngeal microbial markers could be used as an auxiliary diagnostic tool for COVID‐19, making up for the deficiencies of RT‐PCR.

‌We have also validated the feasibility of applying this model in diagnosing SCs as CCs from the perspective of the microbiome. Alterations in the upper respiratory tract microbiome are unique for each disease, such as bronchiectasis,^[^ ^36^ ^]^ colorectal cancer (CRC),^[^ ^37^ ^]^ rheumatoid arthritis,^[^ ^38^ ^]^ and HIV.^[^ ^39^ ^]^ For example, eight oral microbial OTUs in CRCs were differentially abundant from Hs, including *Haemophilus*, *Streptococcus*, and *Prevotella spp*. In our study, the compositions and abundances of the oropharyngeal microbiome in SCs were similar to those of CCs, and the beta diversity of both groups was not obviously different. Therefore, we speculated that suspected cases with positive IgG antibodies are indeed COVID‐19 patients undiagnosed by RT‐PCR due to low virus titer or operator error. A combination of the oropharyngeal microbiome and RT‐PCR may improve the efficacy of the diagnosis of potential COVID‐19 patients.

We are the first to report the oropharyngeal characteristics of recovered patients with COVID‐19. We found an increase in butyrate‐producing *Fusobacterium* and a decrease in the opportunistic pathogen *Leptotrichia*. The increase of butyrate‐producing bacteria could promote the intestinal mucosal barrier repair and contribute to the recovery of COVID‐19. *Leptotrichia* could produce lipopolysaccharides (LPS).^[^ ^20^ ^]^ High level of LPS can induce the initiation of systemic proinflammatory phase by activating toll‐like receptors (TLR) such as TLR4, leading the release of cytokines, reactive oxygen species,^[^ ^40^ ^]^ thereby mediating acute lung injury^[^ ^41^ ^]^ and promoting COVID‐19 development.^[^ ^42^ ^]^ Together, the increase of butyrate‐producing bacteria and the decrease of LPS‐producing bacteria, contribute to the recovery of SARS‐CoV‐2 infection.

Clinical indicators are reported to closely related the severity of COVID‐19. Herein, we studied the association between clinical indicators and oropharyngeal microbiome. Lymphocytes are negatively correlated with OTU762 (*Streptococcus*) (*p* = 0.018, rho = −2.0), which was increased in the CCs. The *Streptococcus pneumoniae* (*Spn*), could mediate various kinds of cell death^[^ ^43^ ^]^ through expression of pore‐forming cytolysin pneumolysin (PLY).^[^ ^44^ ^]^ Grayson KM et al found CD4+ T cells, CD8+ T cells, and NK cells increase their sensitivity to *Spn*‐mediated death under activated conditions.^[^ ^45^ ^]^ Xiang et al. further observed that postinfluenza *Spn* secondary infection induce the suppression and reducing of B lymphocyte compared with *influenza A virus* infection alone.^[^ ^46^ ^]^ In short, we thought that the increase of *Spn* after SARS‐CoV‐2 infection may promote COVID‐19 development through suppressing lymphocyte, which attributed to favorable conditions for *Spn* colonization and multiplication provided by SARS‐CoV‐2 infection.

However, due to the limitations of the actual situation, we included both mild and moderate patients but not severe patients. Therefore, we cannot explore the relationship between the microbiome and disease severity. At the same time, we did not carry out further molecular mechanism research to explore possible pathways and targets of the microbiome affecting disease progression. Furthermore, due to experimental conditions, animal models cannot be established to verify our results in vivo. With further study of the microbiome in individuals with COVID‐19, the use of oropharyngeal microbial markers may be applied to the diagnosis, therapy, and even prevention of the disease.

## Conflict of Interest

The authors declare no conflict of interest.

## Author Contributions

M.G., H.W., H.L., Y.S., and L.W. contributed equally to this work. R.Z.G., C.G.Y., and G.M. designed the study. R.Z.G., L.H., S.Y., W.L., D.S.Y., G.J.Q., R.B.C., L.S.S., W.X.M., L.M.Y., G.X.X., Z.Y.W., and Y.C.Y. collected clinical samples. R.H.Y. and S.J.R. extracted the bacterial DNA. G.M. and S.J.R. performed MiSeq sequencing. W.H.Y., G.M., L.C., S.Y., and Y.C.Y. analyzed the data. W.H.Y., G.M., S.Y., C.G.Y., and R.Z.G. wrote the manuscript. All authors reviewed and approved the manuscript.

## Ethics Approval and Consent to Participate

This study was approved by the Institutional Review Board from The First Affiliated Hospital of Zhengzhou University (2020‐KY‐055) and Guangshan County People's Hospital (2020‐001). The study was performed in accordance with the Helsinki Declaration and Rules of Good Clinical Practice. All participants signed written informed consent after the study protocol was fully explained.

## Patient and Public Involvement

Patients or the public were not involved in the design, conduct, reporting, or dissemination plans of our research.

## Supporting information

Supporting Information

## Data Availability

The data that support the findings of this study are openly available in European Bioinformatics Institute European Nucleotide Archive database at https://www.ncbi.nlm.nih.gov/sars‐cov‐2/, reference number [PRJNA739539].
