# Supplementary material for: Characterization of the Human Oropharyngeal Microbiomes in SARS‐CoV‐2 Infection and Recovery Patients
Source: Adv Sci (Weinh). 2021 Aug 22;8(20):2102785. doi: 10.1002/advs.202102785 (PMC8529429; doi:10.1002/advs.202102785)
Supplement: Supplementary file 1 — Supporting Information [file ADVS-8-2102785-s001.pdf]

© 2021 Wiley-VCH GmbH

Supporting Information

for *Adv. Sci.*, DOI: 10.1002/advs.202102785

Characterization of the human oropharyngeal microbiomes in SARS-CoV-2 infection and recovery patients

Ming Gao[1,2†], Haiyu Wang[2,3†], Hong Luo[4†], Ying Sun[2,3†], Ling Wang[5†], Suying Ding[6], Hongyan Ren[7], Jiaqi Gang[8], Benchen Rao[2,3], Shanshuo Liu[2,3], Xuemei Wang[2,3], Xinxin Gao[6], Mengyi Li[9], Yawen Zou[2,3], Chao Liu[7], Chengyu Yuan[4], Jiarui Sun[7], Guangying Cui[2,3], Zhigang Ren[2,3*]*

Supplementary figures

Characterization of the human oropharyngeal microbiomes in SARS-CoV-2 infection and recovery patients

Authors

Ming Gao [1,2†], Haiyu Wang [2,3†], Hong Luo [4†], Ying Sun [2,3†], Ling Wang [5†], Suying Ding [6], Hongyan Ren [7], Jiaqi Gang [8], Benchen Rao [2,3], Shanshuo Liu [2,3], Xuemei Wang [2,3], Xinxin Gao [6], Mengyi Li [9], Yawen Zou [2,3], Chao Liu [7], Chengyu Yuan [4], Jiarui Sun [7], Guangying Cui [2,3*], Zhigang Ren [2,3*]

Affiliations

[1] Department of Oncology, the First Affiliated Hospital of Zhengzhou University, Zhengzhou 450052, China;

[2] Gene Hospital of Henan Province; Precision Medicine Center, the First Affiliated Hospital of Zhengzhou University, Zhengzhou 450052, China;

[3] Department of Infectious Diseases, the First Affiliated Hospital of Zhengzhou University, Zhengzhou 450052, China;

[4] Guangshan County People's Hospital, Guangshan County, Xinyang 465450, China;

[5] Department of Clinical Laboratory, Henan Provincial Chest Hospital, Zhengzhou 450008, China;

[6] Physical Examination Center, the First Affiliated Hospital of Zhengzhou University, Zhengzhou 450052, China;

[7] Shanghai Mobio Biomedical Technology Co., Ltd., Shanghai 201111, China;

[8] Xiuwu County People's Hospital, Xiuwu County, Jiaozuo 454350, China;

[9] Department of Oncology, Zhengzhou First People's Hospital, Zhengzhou 450004, China;

[†]These authors contributed equally to this work.

***Address correspondence to:**

Zhigang Ren, Ph.D, M.D., Department of Infectious Diseases, the First Affiliated Hospital of Zhengzhou University, #1 Jianshe East Road, Zhengzhou 450052, China. E-mail: fccrenzg@zzu.edu.cn

Guangying Cui, Ph.D, M.D., Department of Infectious Diseases, the First Affiliated Hospital of Zhengzhou University, #1 Jianshe East Road, Zhengzhou 450052, China. E-mail: fcccuigy@zzu.edu.cn

Fig. S1.

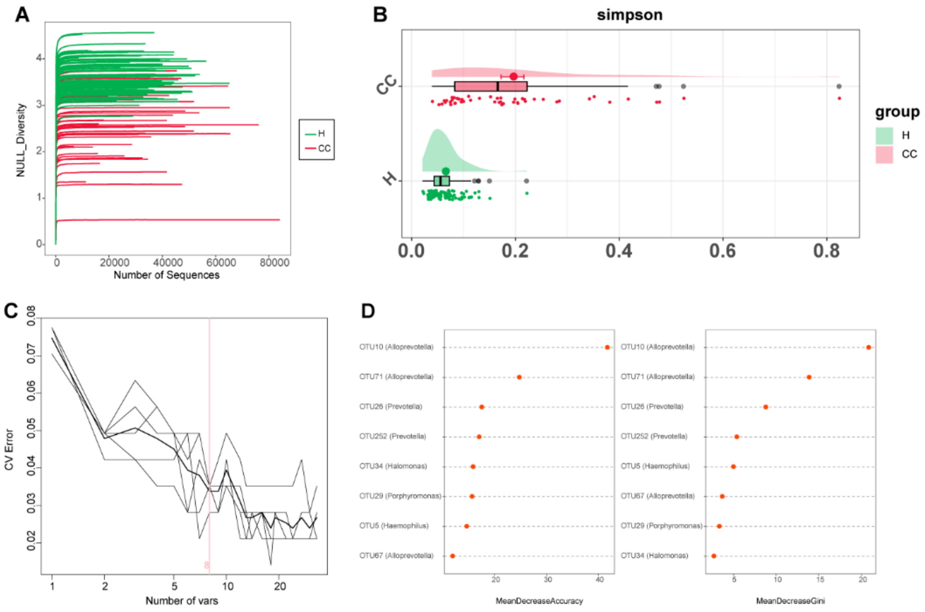

Fig. S1. The oropharyngeal microbial diversity between CC and H groups. (A) A shannon-wiener curve between the number of sequences and the null diversity in CC (n=48) and H (n=94) group. As evaluated by Simpson index (B), oropharyngeal microbial diversity was remarkedly reduced in CC (n=48) versus H (n=94) (p<0.001). (C) Importance distribution map of the selected microbial markers in the model. (D) Eight microbial markers were selected as the best markers set by random forest model. CCs, confirmed cases; Hs, healthy controls; OTUs, operational taxonomic units; CV Error, the cross-validation error. Center line, median; box limits, upper and lower quartiles; circle symbol, mean; error bars, 95% CI.

Fig. S2.

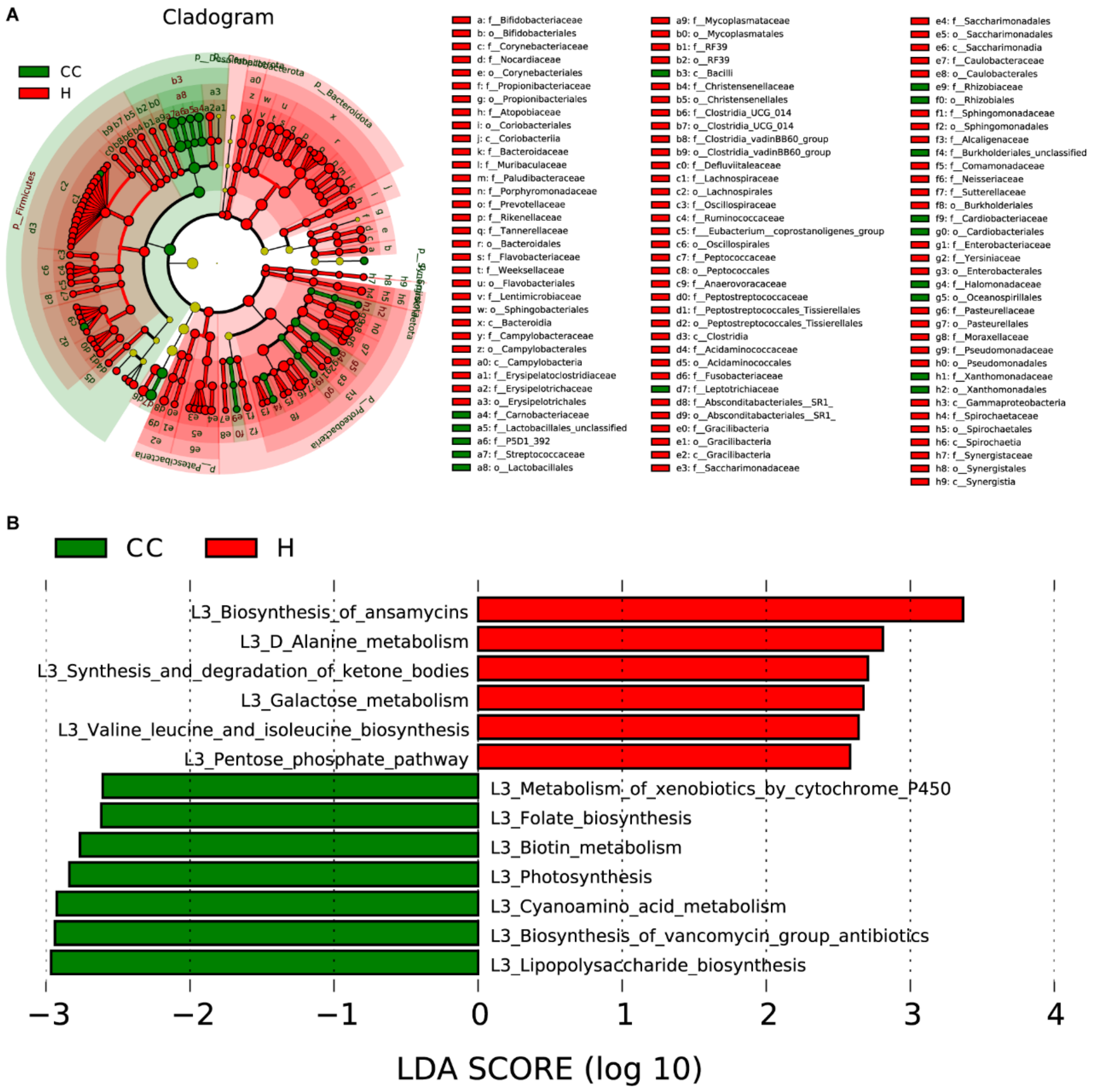

Fig. S2. LEfSe analysis between Hs and CCs. (A) The cladogram, representing oropharyngeal microbial structure and their predominant bacteria, revealed the greatest differences in taxa between CCs (n=48) and Hs (n=94). (B) Based on the LDA selection, 6 gene functions were remarkedly raised, while 7 gene functions were remarkedly reduced in CC versus H (p<0.05, LDA>2.5). CCs, confirmed cases; Hs, healthy controls; LEfSe, linear discriminant analysis (LDA) effect size.

Fig. S3.

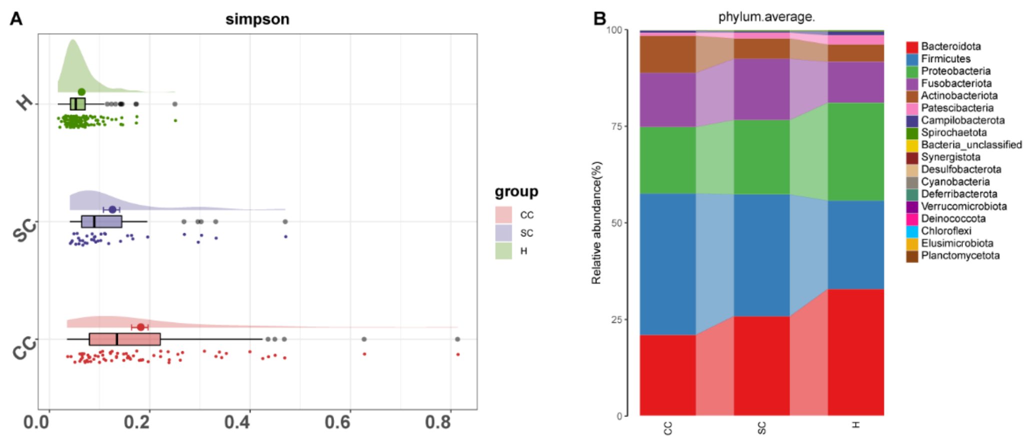

Fig. S3. The oropharyngeal microbial diversity and abundances among CC, SC, and H groups. (A) As evaluated by Simpson index, oropharyngeal microbial diversity of CCs (n=73) and SCs (n=36) was remarkedly decreased versus that in the H (n=140) (p<0.001). (B) Average compositions and relative abundance of the bacterial community among three groups at the phylum level. CCs, confirmed cases; SCs, suspected cases; Hs, healthy controls. Center line, median; box limits, upper and lower quartiles; circle symbol, mean; error bars, 95% CI.

Fig. S4.

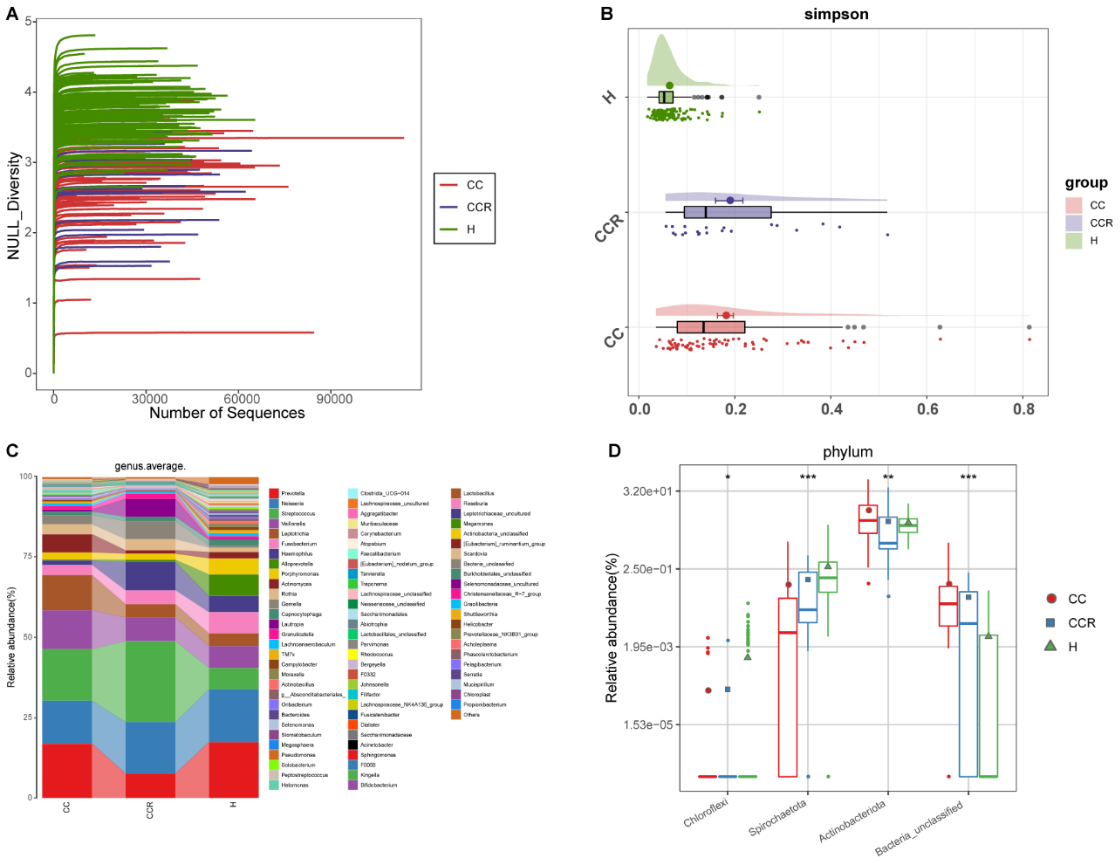

Fig. S4. The characterization and comparison of oral microbial community among CC, CCR, and H groups. (A) A shannon-wiener curve between the number of sequences and the null diversity in CC (n=73), CCR (n=21), and H (n=140). As estimated by simpson index (B), oropharyngeal microbial diversity was significantly decreased in CC and CCR groups compared with that in the H group (p<0.001). (C) Average compositions and relative abundance of the bacterial community among three groups at the genus level. (D) phylum of *Chloroflexi*, *Spirochaetota*, *actinobacteriota* were gradually increased among CC, CCR, and H groups (p<0.05). CCs, confirmed cases; CCRs, confirmed cases who recovered; Hs, healthy controls. Center line, median; box limits, upper and lower quartiles; circle symbol, mean; error bars, 95% CI.

Fig. S5.

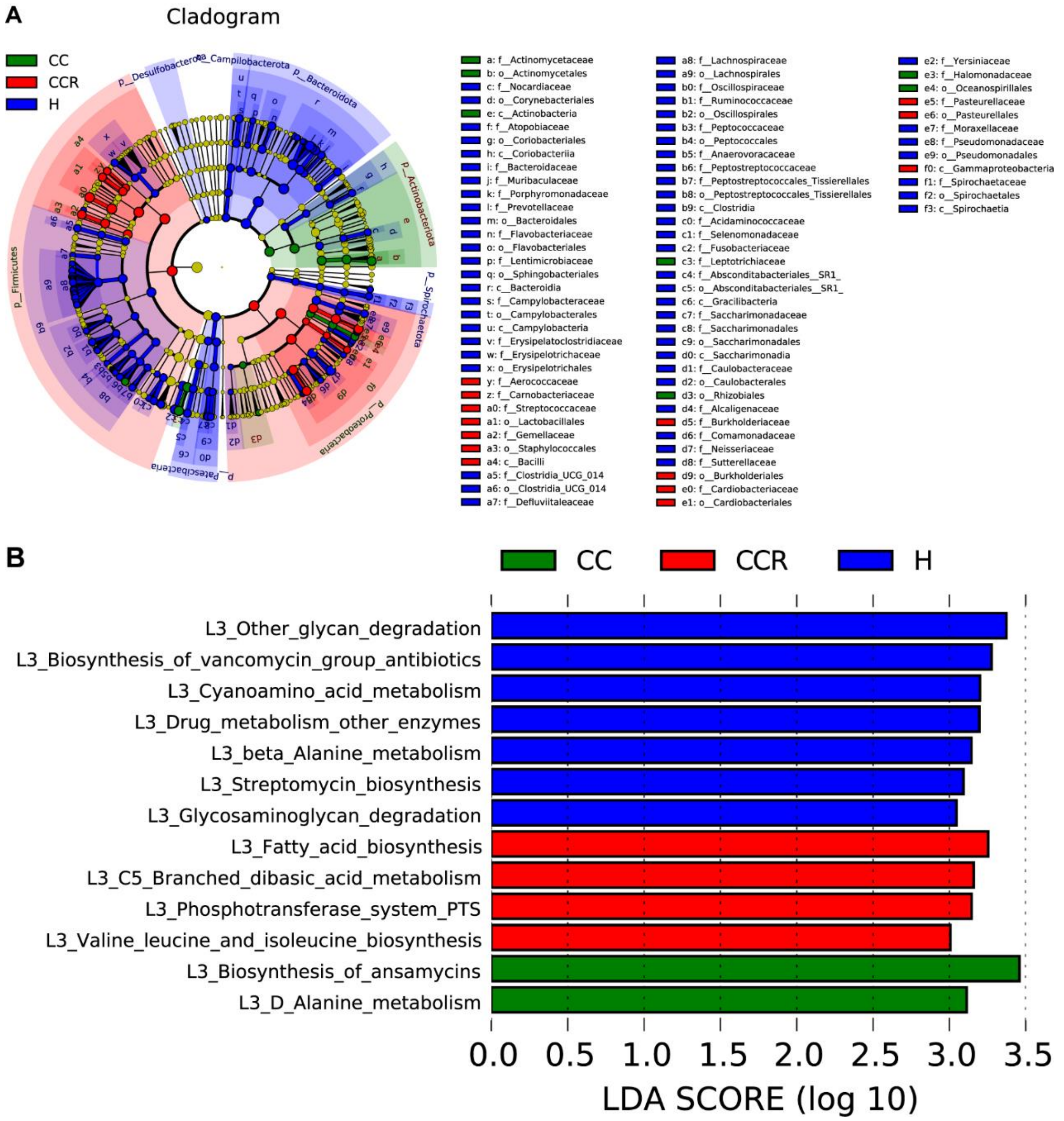

Fig. S5. LEfSe analysis among CC, CCR, and H groups. (A) The cladogram, representing oropharyngeal microbial structure and their predominant bacteria, showed the greatest differences in taxa among CC (n=73), CCR (n=21), and H (n=140) groups. (B) Based on the LDA selection, 7 gene functions were remarkedly enriched in Hs, 4 gene functions were significantly enriched in CCRs, and 2 gene functions were remarkedly enriched in CCs (p<0.05, LDA>3.0). CCs, confirmed cases; CCRs, confirmed cases who recovered; Hs, healthy controls. LEfSe, linear discriminant analysis (LDA) effect size.

Supplementary table legend

Supplementary table S1. Clinical data of all confirmed cases (CCs, n=73) and healthy controls (Hs, n=140).

Supplementary table S2. The detailed values of oral microbial diversity index and observed OTUs in the discovery cohort (94 healthy controls and 48 CCs).

Supplementary table S3. The abundance and composition at the genus and phylum level of each sample in the discovery cohort (94 healthy controls and 48 CCs).

Supplementary table S4. The different degree of microbiome at genus and phylum level (p value) between the healthy controls (n=94) and CCs (n=48) in the discovery cohort.

Supplementary table S5. The relative abundance and distribution of the key 57 OTUs in the discovery cohort (94 healthy controls and 48 CCs).

Supplementary table S6. The cladogram of oropharyngeal microbial structure and their predominant bacteria between CCs and HCs (94 healthy controls and 48 CCs).

Supplementary table S7. The corresponding LDA value and p value of microbial community gene function for samples in the discovery cohort (94 healthy controls and 48 CCs).

Supplementary table S8. By random forest classifier model, the corresponding output value of each optimal microbial marker in the discovery cohort, and the corresponding POD value for each sample in the discovery cohort (94 healthy controls and 48 CCs).

Supplementary table S9. By random forest classifier model, the corresponding output value of each optimal microbial marker in the validation cohort, and the corresponding POD value for each sample in validation cohort (46 healthy controls and 25 CCs).

Supplementary table S10. By random forest classifier model, the corresponding output value of each optimal microbial marker, and the corresponding POD value of each sample in the suspected cases (SCs, n=36) and H (n=46) groups.

Supplementary table S11. The levels of Ig-G antibody in CC (n=21), SC (n=36), and H (n=6).

Supplementary table S12. The detailed values of oral microbial diversity index and observed OTUs among CC (n=73), SC (n=36), and H (n=140) groups.

Supplementary table S13. The abundance and composition at the genus level and phylum level of each sample among CC (n=73), SC (n=36), and H (n=140) groups.

Supplementary table S14. The relative abundance and distribution of the key 58 OTUs among CC (n=73), SC (n=36), and H (n=140) groups.

Supplementary table S15. The average abundance and composition at the genus level among CC (n=73), SC (n=36), CCR (n=21), and SCR (n=36) groups.

Supplementary table S16. The detailed values of oral microbial diversity index and observed OTUs among CC (n=73), CCR (n=21), and H (n=140) groups.

Supplementary table S17. The abundance and composition at the genus and phylum level of each sample among CC (n=73), CCR (n=21), and H (n=140) groups.

Supplementary table S18. The different degree of microbiome at genus and phylum level (p value) among the CC (n=73), CCR (n=21), and H (n=140) groups.

Supplementary table S19. The relative abundance and distribution of the key 90 OTUs among the CC (n=73), CCR (n=21), and H (n=140) groups.

Supplementary table S20. The cladogram of oropharyngeal microbial structure and their predominant bacteria among the CC (n=73), CCR (n=21), and H (n=140) groups.

Supplementary table S21. The corresponding LDA value and p value of microbial community gene function for samples in the discovery cohort (140 healthy controls, 73 CCs, and 21 CCRs).

Supplementary table S22. The p value between 44 oropharyngeal OTUs and 11 clinical indicators of CC (n=48) and H (n=94) obtained by spearman correlation analysis.

Table S1. Clinical data of all enrolled subjects																						
	Cohort	nucleic tesing	ICU or not	sex(1,male;2,female)	age (year)	nationality	height(cm)	weight (kg)	BMI (kg/m2)	Confirmed patient or Wuhan exposure	Smoking or not	comorbidities	diabetes	high blood pressure	cardiovascular diseases	COPD	malignant tumor	chronic liver diseases	signs and symptoms	The highest temperature（°）	cough	muscle pain
CC01	Discovery	positive	N	2	50	Han	168	64	22.68	y	n	n	n	n	n	n	n	n		37.5	n	n
CC02	Discovery	positive	N	2	45	Han	160	73	28.52	y	n	n	n	n	n	n	n	n		38.2	n	n
CC05	Discovery	positive	N	1	39	Han	165	75	27.55	y	y	n	n	n	n	n	n	n		38.8	n	n
CC06	Discovery	positive	N	1	46	Han	176	70	22.6	y	n	y	n	n	n	n	n	y		39.3	n	n
CC08	Discovery	positive	N	1	47	Han	176	72	23.24	y	n	n	n	n	n	n	n	n		37.9	n	n
CC09	Discovery	positive	N	1	39	Han	179	80	24.97	y	y	n	n	n	n	n	n	n		36.8	n	n
CC12	Discovery	positive	N	2	55	Han	169	65	22.76	y	n	y	y	n	n	n	n	n		39.5	n	n
CC16	Discovery	positive	N	2	49	Han	159	56	22.15	y	n	n	n	n	n	n	n	n		38.1	n	n
CC17	Discovery	positive	N	1	51	Han	172	85	28.73	y	n	n	n	n	n	n	n	n		38.5	n	n
CC20	Discovery	positive	N	1	47	Han	175	72	23.51	y	n	y	n	y	n	n	n	n		37.3	y	n
CC21	Discovery	positive	N	1	38	Han	155	50	20.81	y	n	n	n	n	n	n	n	n		38.7	n	n
CC24	Discovery	positive	N	1	36	Han	174	75	24.77	y	n	n	n	n	n	n	n	n		37.9	n	n
CC25	Discovery	positive	N	1	56	Han	170	60	20.76	y	n	n	n	n	n	n	n	n		38.5	n	n
CC26	Discovery	positive	N	1	62	Han	175	60	19.59	y	n	n	n	n	n	n	n	n		38	n	n
CC27	Discovery	positive	N	1	61	Han	175	60	19.59	y	n	y	n	y	n	n	n	n		37.1	y	n
CC28	Discovery	positive	N	2	64	Han	155	70	29.14	y	n	y	n	n	y	y	n	n		37.4	y	n
CC29	Discovery	positive	N	2	46	Han	162	56	21.34	y	n	n	n	n	n	n	n	n		37.2	y	n
CC30	Discovery	positive	N	2	55	Han	157	60	24.34	y	n	n	n	n	n	n	n	n		37	y	n
CC31	Discovery	positive	N	2	46	Han	155	60	24.97	y	n	n	n	n	n	n	n	n		36.5	n	n
CC33	Discovery	positive	N	1	62	Han	170	82	28.37	y	n	y	n	y	y	n	n	n		39.3	n	n
CC34	Discovery	positive	N	1	35	Han	170	74	25.61	y	y	n	n	n	n	n	n	n		37	y	n
CC35	Discovery	positive	N	2	54	Han	148	44	20.09	y	n	y	n	y	n	n	n	n		37.2	y	n
CC36	Discovery	positive	N	2	48	Han	175	68	22.2	y	n	n	n	n	n	n	n	n		37.2	y	n
CC37	Discovery	positive	N	1	54	Han	175	62	20.24	y	n	y	y	n	y	n	n	n		37	y	n
CC39	Discovery	positive	N	2	58	Han	165	55	20.2	y	n	y	n	n	y	n	n	n		36.8	n	n
CC42	Discovery	positive	N	1	71	Han	168	69	24.45	y	n	n	n	n	n	n	n	n		38.2	y	n
CC43	Discovery	positive	N	2	68	Han	155	56	23.31	n	n	n	n	n	n	n	n	n		39.2	y	n
CC44	Discovery	positive	N	1	48	Han	173	90	30.07	y	n	n	n	n	n	n	n	n		38.0	y	n
CC45	Discovery	positive	N	1	12	Han	165	52	19.1	n	n	n	n	n	n	n	n	n		37.2	y	n
CC46	Discovery	positive	N	1	30	Han	178	75	23.67	y	n	n	n	n	n	n	n	n		38.0	n	n
CC48	Discovery	positive	N	2	58	Han	153	55	23.5	y	n	n	n	n	n	n	n	n		38.6	y	n
CC52	Discovery	positive	N	1	22	Han	180	100	30.86	y	n	n	n	n	n	n	n	n		37.7	n	n
CC54	Discovery	positive	N	2	49	Han	165	50	18.37	y	n	n	n	n	n	n	n	n		38.2	y	n
CC55	Discovery	positive	N	2	56	Han	157	60	24.34	y	n	n	n	n	n	n	n	n		37.5	y	n
CC57	Discovery	positive	N	1	35	Han	176	74	23.89	y	n	n	n	n	n	n	n	n		39	y	y
CC58	Discovery	positive	N	1	30	Han	172	85	28.73	y	n	n	n	n	n	n	n	n		38.2	y	n
CC60	Discovery	positive	N	1	65	Han	170	68	23.53	n	n	n	n	n	n	n	n	n		38.2	y	n
CC62	Discovery	positive	N	2	53	Han	158	60	24.03	y	n	n	n	n	n	n	n	n		38	y	n
CC63	Discovery	positive	N	1	48	Han	168	67	23.74	y	y	n	n	n	n	n	n	n		37.4	n	n
CC64	Discovery	positive	N	1	17	Han	165	70	25.71	y	y	n	n	n	n	n	n	n		36.5	n	n
CC65	Discovery	positive	N	2	76	Han	154	60	25.3	y	n	n	n	n	n	n	n	n		36.6	n	n
CC67	Discovery	positive	N	2	43	Han	153	54	23.07	n	n	n	n	n	n	n	n	n		37.6	n	n
CC68	Discovery	positive	N	2	24	Han	154	50	21.08	y	n	n	n	n	n	n	n	n		36.2	n	n
CC69	Discovery	positive	N	1	57	Han	172	80	27.04	y	n	n	n	n	n	n	n	n		36.5	n	n
CC70	Discovery	positive	N	1	68	Han	175	69	28.04	y	n	n	n	n	n	n	n	n		38.5	y	n
CC71	Discovery	positive	N	1	29	Han	178	75	29.04	y	y	n	n	n	n	n	n	n		38.1	y	n
CC72	Discovery	positive	N	1	14	Han	163	52	30.04	n	n	n	n	n	n	n	n	n		37.2	n	n
CC73	Discovery	positive	N	1	39	Han	160	80	31.04	y	n	n	n	n	n	n	n	n		37.4	y	n
CC03	Validation	positive	N	2	56	Han	156	62	25.48	y	n	n	n	n	n	n	n	n		36.8	y	y
CC04	Validation	positive	N	1	40	Han	168	89	26.48	n	n	n	n	n	n	n	n	n		39.2	y	n
CC07	Validation	positive	N	2	56	Han	160	60	23.44	y	n	y	y	n	n	n	n	n		38.1	n	n

CC10	Validation	positive	N	1	50	Han	173	80	26.73	y	n	n	n	n	n	n	n	n		37.7	n	n
CC11	Validation	positive	N	2	51	Han	160	60	23.44	y	n	n	n	n	n	n	n	n		38.1	y	n
CC13	Validation	positive	N	1	40	Han	170	61	21.11	y	n	n	n	n	n	n	n	n		38.8	n	n
CC15	Validation	positive	N	2	44	Han	162	62	23.62	y	n	n	n	n	n	n	n	n		37.6	n	n
CC18	Validation	positive	N	2	65	Han	155	68	28.3	N	n	y	n	y	n	n	n	n		38.1	n	n
CC19	Validation	positive	N	1	39	Han	178	78	24.62	Y	y	y	n	n	n	n	n	y		36.8	n	n
CC22	Validation	positive	N	2	50	Han	159	59	23.34	n	n	y	y	n	n	n	n	n		38.2	n	n
CC23	Validation	positive	N	1	36	Han	175	72	23.51	y	n	n	n	n	n	n	n	n		38.5	n	n
CC32	Validation	positive	N	1	53	Han	170	60	20.76	y	n	n	n	n	n	n	n	n		38	y	n
CC38	Validation	positive	N	1	56	Han	170	85	29.41	y	y	n	n	n	n	n	n	n		38.3	n	n
CC40	Validation	positive	N	1	25	Han	176	68	21.95	y	n	n	n	n	n	n	n	n		38.0	y	n
CC41	Validation	positive	N	2	36	Han	160	53	20.7	n	n	n	n	n	n	n	n	n		37.2	n	n
CC47	Validation	positive	N	2	46	Han	161	54	20.83	y	n	n	n	n	n	n	n	n		37.4	y	n
CC49	Validation	positive	N	1	25	Han	170	65	22.49	n	n	n	n	n	n	n	n	n		38.0	n	n
CC50	Validation	positive	N	1	46	Han	175	80	26.12	y	n	n	n	n	n	n	n	n		38.5	n	n
CC51	Validation	positive	N	2	79	Han	150	65	28.89	n	n	n	n	n	n	n	n	n		36.9	n	n
CC53	Validation	positive	N	1	46	Han	168	76	26.93	y	y	y	n	y	n	n	n	n		36.6	y	n
CC56	Validation	positive	N	1	55	Han	172	70	23.66	y	n	n	n	n	n	n	n	n		38	y	n
CC59	Validation	positive	N	1	57	Han	175	72	23.51	y	n	n	n	n	n	n	n	n		38.5	n	n
CC61	Validation	positive	N	1	61	Han	164	57	21.19	n	y	y	y	n	n	n	n	n		39	n	y
CC66	Validation	positive	N	1	75	Han	165	65	23.88	y	n	n	n	n	n	n	n	n		36.8	n	n
CC74	Validation	positive	N	1	40	Han	178	78	32.04	y	y	y	n	n	n	n	n	y		38.9	n	n
SC01	Validation	negative	N	2	49	Han	160	72	28.13	N	n	n	n	n	n	n	n	n		37.7	n	n
SC02	Validation	negative	N	1	24	Han	170	65	22.49	y	n	n	n	n	n	n	n	n		37.6	y	n
SC03	Validation	negative	N	2	49	Han	155	52	21.64	y	n	y	n	n	n	n	n	y		38	n	n
SC04	Validation	negative	N	2	59	Han	163	65	24.46	N	n	n	n	n	n	n	n	n		38.2	y	n
SC05	Validation	negative	N	2	48	Han	165	62	22.77	N	n	n	n	n	n	n	n	n		38.1	y	n
SC06	Validation	negative	N	1	30	Han	158	71	28.44	y	n	n	n	n	n	n	n	n		37.6	y	n
SC07	Validation	negative	N	1	39	Han	175	80	26.12	y	n	n	n	n	n	n	n	n		38.6	n	n
SC08	Validation	negative	N	1	59	Han	160	65	25.39	y	n	y	y	n	n	n	n	n		38.1	n	n
SC09	Validation	negative	N	2	52	Han	158	60	24.03	y	n	y	y	n	n	n	n	n		37.9	n	n
SC10	Validation	negative	N	1	63	Han	162	69	26.29	y	n	n	n	n	n	n	n	n		37.7	n	n
SC11	Validation	negative	N	1	57	Han	164	68	25.28	y	n	n	n	n	n	n	n	n		38.3	n	n
SC12	Validation	negative	N	1	50	Han	170	77	26.64	y	y	y	n	n	n	n	n	y		37.9	n	n
SC13	Validation	negative	N	2	47	Han	156	54	22.19	y	n	y	y	n	n	n	n	n		38.1	y	n
SC14	Validation	negative	N	1	32	Han	157	69	27.99	y	n	y	n	y	n	n	n	n		37.8	y	n
SC15	Validation	negative	N	2	31	Han	160	62	24.22	y	n	n	n	n	n	n	n	n		37.5	n	n
SC16	Validation	negative	N	2	39	Han	162	64	24.39	y	n	n	n	n	n	n	n	n		38.2	n	n
SC17	Validation	negative	N	1	56	Han	162	74	28.2	y	n	n	n	n	n	n	n	n		376	y	n
SC18	Validation	negative	N	2	39	Han	164	70	26.03	N	n	n	n	n	n	n	n	n		37.7	n	n
SC19	Validation	negative	N	2	45	Han	160	70	27.34	y	n	n	n	n	n	n	n	n		37.6	n	n
SC20	Validation	negative	N	1	25	Han	168	70	24.8	N	n	n	n	n	n	n	n	n		38.7	n	n
SC21	Validation	negative	N	1	2	Han	80	11	17.19	y	n	n	n	n	n	n	n	n		37.9	n	y
SC22	Validation	negative	N	1	33	Han	175	65	21.22	y	n	y	n	n	n	n	y	n		39.2	n	n
SC23	Validation	negative	N	2	40	Han	165	55	20.2	y	n	n	n	n	n	n	n	n		37.5	n	n
SC24	Validation	negative	N	2	0.83	Han	75	9	16	y	n	n	n	n	n	n	n	n		38.1	n	n
SC25	Validation	negative	N	1	45	Han	172	65	21.97	y	y	n	n	n	n	n	n	n		37	n	n
SC26	Validation	negative	N	1	58	Han	169	80	28.01	y	y	y	n	n	n	n	n	y		38.8	y	n
SC27	Validation	negative	N	2	58	Han	160	57	22.27	N	n	n	n	n	n	n	n	n		37.7	y	n
SC28	Validation	negative	N	1	28	Han	198	58	14.79	N	n	n	n	n	n	n	n	n		38	n	n
SC29	Validation	negative	N	1	26	Han	175	64	20.9	y	n	n	n	n	n	n	n	n		37	n	n
SC30	Validation	negative	N	2	42	Han	160	65	25.39	N	n	n	n	n	n	n	n	n		38.2	n	n
SC31	Validation	negative	N	1	70	Han	170	70	24.22	N	n	n	n	n	n	n	n	n		39.2	y	n
SC32	Validation	negative	N	1	36	Han	167	75	26.89	y	n	n	n	n	n	n	n	n		39.3	y	n
SC33	Validation	negative	N	1	32	Han	168	60	21.26	y	n	n	n	n	n	n	n	n		37.6	n	n
SC34	Validation	negative	N	2	34	Han	170	61	21.11	N	n	n	n	n	n	n	n	n		37.9	n	n
SC35	Validation	negative	N	2	40	Han	172	66	22.31	y	n	n	n	n	n	n	n	n		39.3	y	n
SC36	Validation	negative	N	1	65	Han	170	77	26.64	Y	y	y	y	n	n	n	n	n		38.9	n	n

fatigue	cough phlegm	headache	diarrhea	dyspnea	Time from illness to dyspnea(day)	time from illness to admission(day)	Total hospital stay (day)	systolic pressure (mmHg)	diastolic pressure(mmHg)	heart rate(Times/min)	respiratory rate(Times/min)	laboratory testing results	Red blood cell （10^9/L）	White blood cell （10^9/L）	Neutrophil （10^9/L）	Lymphocyte （10^9/L）	Hemoglobin(g/L)	Platelet （10^9/L）	Prothrombin time (s)	Activated partial thromboplastin time	D-dimer (mg/L)	Albumin (g/L)	ALT (U/L)	AST (U/L)	Total bilirubin (mmol/L)
n	n	n	n	n	n	7	16	130	84	94	24		4.51	3.21	1.51	1.42	129	128	10.8	25.2	0.11	37.6	12.5	21.8	6.6
n	n	n	n	n	n	9	17	120	86	81	21		5	4.51	2.25	1.9	150	124	10.6	23.8	0.15	43.5	43.9	34.2	12.6
n	n	n	n	n	n	5	17	125	78	86	18		4.75	3.06	1.48	1.48	139	291	14.3	25.2	0.03	43.5	9.2	15.3	14.5
n	n	n	n	n	n	2	20	109	86	76	21		4.56	5.13	3.5	1.35	138	241	17.3	31.3	0.22	37.4	31.5	36.3	17.4
n	n	n	n	n	n	5	18	109	76	78	15		4.56	3.13	2.08	0.91	137	118	14.2	31.4	0.30	42.2	44.6	27.5	18.4
n	n	n	n	n	n	6	14	106	86	74	17		5.32	3.53	2.53	0.95	153	136	13.5	21.5	0.07	46.3	30.2	21.5	34.2
n	n	n	n	n	n	10	25	109	87	75	15		4.16	3.87	2.37	0.99	132	142	10.5	29.3	0.45	38.3	18.2	20.3	7.8
n	n	n	n	n	n	9	15	98	67	78	14		4.47	10.8	8.92	1.51	136	319	9.2	23.2	0.27	39.5	19.6	16.1	9.24
n	n	n	n	n	n	4	19	119	78	96	19		5.59	6.07	4.42	1.27	172	150	12.6	21.5	0.39	47.2	41.2	24.3	19.3
y	n	n	n	n	n	13	12	160	110	120	24		4.89	8.18	6.08	1.71	142	410	9.6	27.8	0.16	42.3	111.9	33.2	12.8
n	n	n	n	n	n	8	14	118	89	87	15		4.54	5.39	3.30	1.78	126	211	9.25	28.8	0.45	46.5	17.7	22.8	8.5
n	n	n	n	n	n	5	15	130	93	87	19		5.98	9.69	7.85	1.04	148	270	10.9	24.9	0.32	60.6	21.5	17	12.6
n	n	n	n	n	n	11	16	161	102	90	20		4.94	11.8	10.25	1.06	144	374	9.2	21.2	2.6	38.1	34	18	26.3
n	n	y	n	n	n	3	13	129	80	80	20		4.21	12.3	10.95	1.03	124	240	11	23.2	0.4	62.8	14	10	46.3
n	n	n	n	n	n	3	17	145	108	80	20		5.08	8.3	5.79	1.94	160	138	11.5	24.2	0.1	41.4	10	20	53.8
n	n	n	n	y	1	1	18	130	80	80	20		3.33	5.6	3.70	1.53	117	150	13.2	25.2	2.1	41.6	16	25	41.5
n	n	n	n	n	n	3	27	150	89	90	20		4.14	4.3	3.00	0.96	91	230	10	29.4	0.06	41.0	39	29	20.0
n	n	n	n	n	n	20	15	107	70	80	20		4.22	7.1	4.51	2.06	125	287	n	n	0.6	41.9	12	15	13.1
n	n	n	n	n	n	1	16	118	65	80	20		3.80	5.7	3.53	1.75	114	210	11	23.4	0.2	38.4	38	31	19.8
n	n	n	n	n	n	3	21	153	87	90	20		5.11	6.9	6.03	0.71	150	116	11.9	25.3	0.6	38.7	56	71	12.0
n	n	n	n	n	n	1	11	120	80	84	20		5.11	8.1	4.54	2.79	150	330	11.6	23.1	0.1	40.5	15	11	22.0
n	n	y	n	n	n	1	12	163	94	110	20		3.65	6.2	5.12	0.63	106	308	n	n	1.9	36.1	10	15	5.0
n	n	y	n	n	n	1	11	119	87	80	20		4.26	5.3	3.54	1.44	113	269	18.2	25.2	0.4	46.3	8	16	37.2
n	n	n	n	n	n	7	11	142	104	80	20		5.10	6.9	4.48	1.76	154	210	n	n	0.1	42.2	13	9	20.2
n	n	n	n	n	n	14	7	120	96	100	20		4.16	4.9	2.49	2.13	123	247	17.8	28.9	0.52	40.5	36.8	36.6	7.6
n	y	n	n	n	n	3	14	105	62	102	18		5.15	6.45	6.77	0.69	126	93	132	27.3	0.26	40	18	42	18.3
n	y	n	n	n	n	3	16	110	68	79	20		4	4.98	7.54	2.18	126	170	14.5	29.8	0.29	41.1	21	37	5.7
n	y	n	n	n	n	5	18	133	77	95	20		3.57	5.22	8.21	0.75	156	196	14.9	33	0.3	46.9	40	26	8.4
n	n	n	n	n	n	4	18	147	70	88	22		5.21	4.75	5.62	2.28	137	177	14	38.3	0.31	52.6	8	20	2.8
n	n	n	n	n	n	5	16	113	70	84	20		5.23	3.1	6.38	1.65	144	114	15.8	35.1	0.27	42.6	8	23	8.1
n	n	n	n	n	n	2	14	100	60	88	16		4.21	4.6	8.52	1.09	105	140	15.5	45.8	0.29	40.2	12	27	2.8
n	n	n	n	n	n	3	12	121	84	95	20		5.11	4.57	2.72	1.42	153	259	9.7	25.8	1.37	41.6	74.4	79.3	8.6
n	n	n	n	n	n	4	12	130	90	80	20		5.02	2.36	1.36	0.83	151	113	12.7	33.9	0.35	40.4	20	24	4.8
n	n	n	n	n	n	4	14	108	78	80	18		4.98	7.23	5.72	1.09	129	325	12.1	25.4	0.49	34.7	12	23	5.8
y	n	n	n	n	n	4	23	125	82	76	22		3.56	5.34	3.78	1.08	139	173	12.5	23.6	0.28	39.9	30	31	7.2
y	n	n	n	n	n	10	17	122	76	79	20		4.23	4.15	3.13	0.71	164	176	13	27.9	0.32	45.6	35	28	6.5
n	y	n	n	n	n	15	20	130	70	69	18		5.23	4.2	5.1	2.25	128	164	n	n	0.21	40.2	34	23	5.2
n	n	n	n	n	n	1	12	135	89	80	19		5.23	3.61	4.16	6	132	193	13.2	22.5	0.25	39.2	21	25.2	8.2
y	n	n	n	n	n	7	21	136	92	76	19		4.43	4.78	4.23	1.81	141	133	13.5	27.4	0.2	42.4	44.3	29.2	14.1
n	n	n	n	n	n	4	29	126	82	72	18		5.24	4.31	2.14	1.72	155	209	14.6	31.1	0.27	50	15	16	10.9
n	n	n	n	n	n	1	6	123	91	102	24		4.25	5.37	3.28	1.65	115	210	12.7	23.4	0.75	43.2	30	40	6.4
n	n	n	n	n	n	1	9	125	75	68	18		4.13	5.18	3.37	1.87	138	219	14.6	30.1	0.39	45.8	15	17	7.7
n	n	n	n	n	n	1	12	126	68	71	21		4.37	5.98	2.79	2.83	135	162	14.6	33.3	0.26	48.3	13	18	19.6
n	y	n	n	n	n	3	13	100	64	98	16		4.8	5.54	3.69	1.5	342	159	12.8	27.3	0.03	43.1	38	27	8.5
n	n	n	n	n	n	1	7	144	88	83	20		4.9	6.04	8.43	15	120	94	15.2	26.7	0.21	42	15	45	18.1
n	y	n	n	n	n	1	6	136	76	76	22		5.12	7.53	4.84	2.06	145	227	14.2	35.2	0.27	47.5	18	17	14
n	n	n	y	n	n	2	15	128	80	90	23		5.23	3.1	5.74	0.93	125	98	13.2	33.6	0.36	46	17	26	6.4
n	n	n	n	n	n	7	7	116	69	79	19		4.32	7.19	3.84	2.65	135	61	13.6	28.4	0.28	43.8	40	27	9.4
y	n	y	n	n	n	7	25	124	74	82	20		4.21	3.5	1.49	1.73	137	183	9.8	24.9	0.26	44.1	22.6	0.89	9.4
y	y	n	n	y	15	14	30	120	87	93	24		4.87	8.13	6.24	1.35	146	213	363	21.5	0.31	42.5	28.2	23.5	9.1
n	n	n	n	n	n	4	18	128	87	75	19		4.21	3.53	3.09	0.45	136	146	17.5	30.2	0.51	41.3	27.4	21.5	7.4

n	n	n	n	n	n	7	21	109	87	79	19		6.46	6.43	3.6	2.35	184	147	21.6	28.2	0.07	45.8	74.4	35	11.2
y	n	y	n	n	n	4	24	124	82	100	20		3.93	2.94	1.73	0.93	116	122	11.3	28.6	0.16	40.3	11	10.3	14.5
n	n	n	n	n	n	15	21	98	65	69	16		5.32	3.82	2.98	0.72	159	209	13.5	27.5	0.15	35.3	20.5	35.3	11.4
n	n	n	n	n	n	15	27	100	78	75	17		3.13	2.42	1.78	0.56	120	89	9.2	21	0.04	44.2	20.2	25.3	9.45
n	y	n	n	n	n	2	17	161	92	98	17		4.42	5.13	4.13	1.09	135	287	10.6	18.2	0.71	41.3	20.3	30	15.2
n	n	n	n	n	n	4	14	104	68	78	16		5.56	4.70	3.30	1.19	175	303	11.2	19.2	0.45	45.7	10.4	14.7	6.3
n	n	n	n	n	n	5	14	129	99	90	18		3.58	3.09	1.98	1.04	123	180	11.3	25.2	0.72	40.1	28.2	29.2	7.2
y	n	n	n	n	n	6	16	129	90	90	20		6.14	10.3	8.74	1.07	147	268	10.6	24.8	0.3	62.8	22	16	13.1
n	n	n	n	n	n	1	22	148	95	90	20		5.22	4.9	3.17	1.23	150	179	12.3	24.1	0.1	39.8	18	24	28.1
y	n	n	n	y	15	13	22	142	91	113	21		3.88	7	4.92	1.51	117	267	11.2	25.1	0.4	32.8	50	20	10.2
n	n	n	n	n	n	3	14	105	62	102	18		4.25	8.59	3.18	1.21	161	319	14.9	27.6	0.32	39.2	23	23	7.7
n	n	n	n	n	n	3	16	110	68	79	20		4.23	2.64	4.08	1.49	108	128	14.4	37.2	0.27	45.4	7	14	6
n	n	n	n	n	n	2	11	110	70	76	19		4.75	3.73	5.37	1.28	102	132	16.9	38.7	0.36	39	23	48	11.6
n	n	n	n	n	n	3	12	120	80	80	19		5.21	6	4.09	1.74	152	199	15	34	0.29	48.5	18	27	34.3
n	n	n	n	n	n	4	16	147	86	95	21		5.25	7.79	5.87	1.32	160	294	13.2	28.6	0.33	42	25	25	7.7
y	n	n	n	n	n	10	10	143	79	83	22		4	4.98	5.73	2.03	126	170	14.5	29.8	0.29	14.1	21	37	5.7
n	n	n	n	n	n	6	19	162	90	79	21		4.23	4.98	4.23	0.73	139	223	13.5	36.2	0.35	49	29	15	10.3
y	n	n	n	n	n	15	20	130	70	69	18		4.23	4.55	2.87	1.15	138	163	11	25.6	0.38	39.5	47	45	8.8
y	n	y	n	n	n	14	15	114	76	80	19		4.63	3.61	2.44	0.91	137	285	12.5	31	0.24	35.3	33	35	5.9
y	n	n	n	n	n	5	11	135	85	86	18		4.79	4.79	3.38	0.89	141	119	12.5	25.4	0.15	33.8	20.6	23.7	18.1
n	n	n	n	n	n	2	7	126	69	69	21		5.21	3.84	6.19	1.69	165	187	13.8	26.5	0.31	49	33	26	8.4
n	n	n	n	n	n	6	13	128	86	90	21		5.04	6.23	5.39	1.52	160	205	13.2	28.6	0.33	46	23	26	8.5
n	n	n	n	n	n	3	13	140	90	92	20		4.61	8.92	5.91	2.42	122	192	无	无	0.21	45.1	12.7	13.8	19.7
n	n	n	n	n	n	1	17	100	64	88	19		5.63	6.73	3.21	3.11	164	262	10.1	26.6	0.06	50.1	6.8	16.6	25.1
n	n	n	n	n	n	2	12	120	80	90	20		3.76	4.38	3.02	1.08	93	120	无	无	0.11	51.7	17	22.1	26.2
n	n	n	n	n	n	2	16	134	88	88	20		4.56	6.63	5.19	1.06	180	134	12.2	27.4	0.28	45.2	15.5	18.2	11.1
n	n	n	n	n	n	3	14	125	84	89	17		5.87	8.5	6.63	1.35	152	210	无	无	0.21	48.1	16.9	16.1	18.8
n	n	n	n	n	n	2	11	130	78	88	18		5.29	4.51	2.04	2.19	102	204	8.8	24	0.24	44.4	15.6	15.4	15.4
n	n	n	n	n	n	4	10	130	78	85	17		5.25	4.31	1.68	0.37	123	180	11.4	18.2	0.88	34.1	18.4	22.3	13.2
n	n	n	n	n	n	8	12	127	80	84	20		5.19	2.24	1.65	0.34	153	102	无	无	1.09	35.4	62.6	79.4	5.54
n	n	n	n	n	n	6	15	133	77	90	19		4.42	1.6	1.06	0.44	115	96	无	无	0.88	41.8	46.6	50.5	10.6
n	n	n	n	n	n	4	25	141	81	96	18		4.98	2.69	1.88	0.69	127	72	12.5	33.5	0.02	43.6	13.9	25.3	12.3
n	n	n	n	n	n	9	15	130	87	90	18		4.89	2.43	1.45	0.31	145	120	10.3	15.9	0.56	45.6	45.6	54.6	5.57
n	n	n	n	n	n	5	9	139	89	85	21		3.47	2.58	1.89	0.39	198	124	12.4	14.5	0.58	48.5	48.4	53.2	15.3
n	n	n	n	n	n	4	10	119	77	86	17		4.14	4.56	3.54	1.45	99	125	11.4	13.4	0.55	54.3	16	25.6	27.5
n	n	n	n	n	n	5	12	145	96	97	20		5.43	4.56	2.45	2.67	157	246	8.6	21	0.2	44.5	14.8	15.3	15.7
n	n	n	n	n	n	1	11	138	88	94	18		5.32	6.42	2.45	1.56	143	264	13.1	24.3	0.45	49.7	11.1	13.5	6
n	n	n	n	n	n	6	14	140	86	99	23		5.42	3.45	1.86	0.64	146	253	13.5	20.5	0.5	47.3	13.6	15.2	16.4
n	n	n	n	n	n	2	13	132	86	90	18		5.11	6.29	4.48	1.17	161	138	12	17.6	0.68	42.5	54.1	38.8	21.3
n	n	n	n	n	n	2	9	118	89	87	22		4.31	1.45	1.87	0.46	136	137	14	18.6	0.69	46.4	48.5	58.3	14.5
n	n	n	n	n	n	1	13	120	72	84	20		4.26	2.55	1.59	0.76	93	215	14	33.6	0.36	46	14.5	16	19.7
n	n	n	n	n	n	1	12	116	90	132	22		4.65	6.63	5.16	0.97	143	175	11.7	24.3	1.09	50.2	18.5	19.5	12
n	n	n	n	n	n	3	7	113	88	90	26		4.3	6.65	2.39	3.68	114	134	13.8	33.6	0.46	46.7	10.9	42.2	8.2
n	n	n	n	n	n	14	6	112	72	118	20		3.47	1.5	0.83	0.62	97	48	11.8	24.3	0.12	38.9	67.6	82.1	9.2
n	n	n	n	n	n	3	12	114	70	100	20		3.72	4.34	2.49	1.58	99	146	无	无	0.17	45.6	8.4	11.6	16.2
n	n	n	n	n	n	4	13	103	61	108	21		3.87	3.85	2.8	0.8	111	91	无	无	0.34	39.3	7.4	10.7	9.4
n	n	n	n	n	n	1	13	117	81	88	19		5.74	8	5.44	2.05	165	199	10.6	32.5	0.21	46.8	17.2	11.6	16.9
n	n	n	n	n	n	7	17	120	77	79	17		6.42	7.85	6.42	2.57	146	221	11.5	35.5	0.26	45.3	10.3.	12.6	14.7
n	n	n	n	n	n	3	12	140	88	90	20		4.26	5.72	3.95	1.27	131	128	10.5	27.7	0.21	42.3	10.8	13.2	16.5
n	n	n	n	n	n	3	13	109	78	98	20		4.68	5.16	3.37	1.28	142	182	无	无	0.26	41.6	7.8	10.6	8.8
n	n	n	n	n	n	3	14	115	76	104	22		5.01	19.1	16.29	2.05	141	210	无	无	0.21	40.4	18.6	11.2	19.1
n	n	n	n	n	n	2	18	120	84	90	20		4.45	6.25	5.37	0.48	126	79	12.3	15.2	0.45	40.9	10.7	12.2	11.1
n	n	n	n	n	n	7	11	126	72	80	20		4.72	11.3	10.28	0.55	137	239	12	24.4	0.56	36.2	51.2	42	12.5
n	n	n	n	n	n	1	11	140	90	98	28		4.31	15.3	5.75	0.42	175	218	16.8	29.1	0.37	42.1	34	17.3	16.8
n	n	n	n	n	n	3	12	112	73	90	18		4.73	5.16	3.26	1.58	139	288	12	21.9	0.19	45.9	14.9	14.5	12.5
n	n	n	n	n	n	5	15	108	77	85	17		4.32	5.31	4.2	1.46	147	247	10.3	24.1	0.16	46.3	14.2	16.2	11.5
n	n	n	n	n	n	1	11	115	79	105	20		3.93	8.76	7.27	0.96	109	202	9.34	24	0.56	38	14.4	17.3	8.5
y	n	n	n	n	n	4	16	130	83	101	23		4.44	5.37	3.85	1.31	137	186	10.4	24.3	0.24	44.6	15.2	14.5	6

Potassium ion,(mmol/L)	Sodion (mmol/L)	Ccreatinine (μmol/L)	Creatine kinase (U/L)	Lactic dehydrogenase (U/L)	hs-cTnI, pg/mL3	PCT (ng/mL)	CRP(ng/mL)	HS-CRP(ng/mL)	ESR(mm/h)
4	144.3	46.7	33.2	179.8	0.53	0.14	13.4	》10	44
3.46	139.6	67.4	104	266.9	0.52	0.2	≤10	《0.499	64
3.97	109.5	57	101	153.6	0.53	《0.1	《10	2.05	26
4.75	136.3	85.3	41	245.2	0.41	0.24	23.5	》10	21
3.88	140.8	63.3	65.9	303.9	0.60	《0.1	22	》10	14
3.51	146.2	135	85.3	204.2	0.4	0.14	35.2	》10	10
5.23	139.4	70.2	103	180.3	0.45	0.14	26.8	》10	46
5.56	140.8	47.9	32	221.2	0.01	0.13	10.1	》10	5
4.01	139.0	67.5	28.5	180.1	0.01	0.19	《10	6.43	47
4.12	140.6	54.9	139	240.9	0.64	<0.1	《10	《0.499	30
4.49	138.3	43	51.8	234.8	0.68	0.11	《10	2.06	12
3.57	143.2	53.7	7	226	0.55	0.15	29.5	3.97	80
3.33	142.2	65.3	148	343	0.02	0.13	61.4	》10	118
3.99	139.8	86.6	23	112	0.2	0.2	22.3	》10	40
3.66	140.4	77.3	23	148	0.23	0.14	37.3	》10	52
3.69	140.7	84.2	58	190	0.04	0.19	55.5	》10	135
3.20	139.0	52.8	25	129	0.06	0.02	18.01	》10	73
3.56	139.7	57.8	32	163	0.01	0.19	5.3		115
3.77	138.9	49.7	37	182	0.67	0.16	6.8		34
3.87	138.5	76.7	436	402	0.52	0.29	67.2	》10	48
3.64	141.8	72.1	25	133	0.57	0.34	0.3		15
6.14	131.4	1187	22	196	0.43	6.85	110.6	》10	128
2.97	141.2	68.0	44	191	0.47	0.19	4.90		110
3.61	140.3	57.0	65	141	0.48	0.16	10.6	》10	25
3.1	139	46.4	55.4	249.1	0.38	N	<10	1.31	44
3.52	146.1	94.2	125	363	0.67	0.05	28.1	＞3	37
3.64	139.2	59.9	61	249	0.45	0.051	20.67	＞3	39
3.51	138	202	45	215	0.64	0.34	16.65	＞3	67
3.55	142.8	52.2	66	186	0.34	0.04	<3	2.27	23
3.8	138.9	76.9	43	215	n	0.04	<3	0.63	8
3.08	141.2	71.1	158	296	0.05	0.132	117.77	＞3	32
5.77	142.4	94.5	540	561.6	0.80	0.11	136.3	》10	87
3.6	139.4	57	42	179	0.01	0.13	<10	<0.499	13
3.8	140.1	55	25	236	0.02	0	38.08	>10	127
3.4	139	83	388	215	0.25	0.09	<10	<0.5	6
4.2	142.2	77	83	204	0.27	0	<10	<0.499	16
3.8	139.2	69	42	198	0.07	0.14	1.3	1.3	19
6.2	139.2	46	45	153	0.23	0.21	20.4	20.4	29
3.9	138	93	136	164.8	0.028	0.05	10.31	>10	8
4.35	139.9	74.9	98	140	0.25	0.04	<3	<0.5	7
4.25	142.9	46.8	86	192	0.025	0.04	<3	<0.5	20
4.41	138.5	47.3	72	141	0.036	0.04	<3	<0.5	2
3.58	137	42.1	59	110	0.043	0.04	<3	<0.5	9
4.31	136.4	79.4	56	127	0.06	0.12	<3	<0.5	6
5.02	140.6	94.8	124	408	0.36	0.05	27.8	＞3	14
4.27	140.3	64.2	74	137	0.025	0.18	<3	<0.5	4
4.45	138.1	48.4	129	156	0.64	0.20	3.54	＞3	12
4.38	140.1	78	101	152	0.025	0.03	<3	<0.5	5
4.82	142.2	53.6	76.2	183.6	0.38	0.21	<10	0.91	20
5.75	146.2	68.3	46.1	275.4	0.26	0.14	15	>10	81
5.32	135.3	67.3	63	274.3	0.53	0.21	53.2	》10	87

5.25	140.7	129	164	249.4	1.26	0.19	10.3	》10	34
4.02	141.5	60.5	36.2	180.8	0.72	<0.1	>10	13.5	60
4.25	135.3	74.2	180	370.4	0.01	0.13	17.5	》10	7
4.01	139.2	56	102	198.2	0.02	0.13	53.4	》10	99
4.2	145.2	48.5	30.1	213	0.67	《0.1	《10	《0.499	53
2.87	143.3	49.3	69	195.8	0.51	0.32	69.4	》10	17
4.3	140.2	60.2	58.3	210.3	0.56	0.2	《10	3.45	60
3.14	141.6	54.4	21	206	0.47	0.18	28.7	3.76	85
3.16	138.0	79.5	7	225	0.68	0.29	24.5	》10	50
2.9	135	67.6	5	304	0.05	0.17	<10	2.57	130
3.84	138.1	103	101	196	0.31	0.07	35.3	》10	46
3.67	141.6	53.7	46	123	0.42	0.04	26.3	》10	45
3.98	140.9	88.5	50	219	0.02	0.04	<3	<0.5	14
3.73	140.1	92.7	61	172	0.13	0.04	<3	1.14	10
4.03	137.1	78.2	68	165	0.05	0.04	8.8	>3	11
		59.9	61	249		0.051	20.67	> 3	
3.73	142.3	56.2	46	159.3	0.53	0.12	<10	<0.499	13
3.2	138.9	57	56	212	0.34	0	10.63	>10	2
3.7	138.5	71	38	205	0.31	0	15.4	>10	43
4.28	139.7	60.5	204	210.9	0.31	0.05	57.06	>10	11
4.32	140.9	62.5	59	151	0.03	0.06	<3	<0.5	4
4.03	137.1	80.3	68	165	0.05	0.04	8.8	>3	11
4.67	139.8	53.3	61.6	167.3	0.71	0.13	≤10	≤0.499	25
4.84	144.4	81.8	164	135.6	0.54	《0.1	≤10	≤0.499	40
3.87	140.1	44.1	56.9	206.3	0.54	0.16	≤10	≤0.499	7
4.48	145.6	57.3	123	196.4	0.5	《0.1	≤10	≤0.499	26
4.82	145.6	96.1	92.3	180.1	0.63	《0.1	≤10	3	21
4.55	142.5	49.2	47.7	128.8	0.46	《0.1	≤10	8.54	7
4.67	138.1	45.3	54.3	241.3	0.41	《0.1	≤10	≤0.499	24
4.78	136.1	65.9	33	228.4	0.02	0.39	14.4	≥10	22
4	145.8	51.1	52.6	270.1	0.52	《0.1	≤10	9.37	11
3.91	142	65.3	173	228.9	0.48	0.11	132.6	≥10	110
4.36	146.1	64.9	27	210.9	0.04	0.45	15.3	≥10	25
4.35	143.6	64.2	48	201.3	0.67	0.46	≤10	≤0.499	18
3.98	143.1	46.5	58.7	202.3	0.45	0.14	≤10	≤0.499	8
4.32	154.7	47.4	48.6	143.5	0.56	《0.1	≤10	8.69	6
4.59	139.4	53.9	105	197	0.6	《0.1	《10	《0.499	11
4.75	147.4	55.3	98.4	145.1	0.53	《0.1	《10	《0.499	15
4.88	140.1	71.1	37.8	136.7	0.51	0.17	14.8	》10	62
4.67	157.4	59.5	53.6	265.3	0.46	《0.1	≤10	8.35	15
4.29	140.2	61.8	52.3	155.3	0.66	《0.1	《10	《0.499	8
3.72	137.9	98.5	123	194.9	0.64	0.17	29.9	》10	6
4.45	134.1	38.3	114	367.2	0.73	1.5	《10	0.51	8
3.9	129.6	67.5	40.8	755	0.57	1.16	78.6	》10	18
4.04	141.1	47.5	35.8	130.5	0.65	0.13	《10	《0.499	35
4.3	138.9	62.2	42	126.9	0.49	0.13	≤10	《0.499	30
4.23	142.1	69.1	52.6	156.8	0.83	《0.1	《10	《0.499	3
5.32	145.7	70.4	46.2	164	0.35	《0.1	《10	《0.499	6
4.63	138.4	53.2	23.1	138.9	0.46	0.17	《10	5.35	2
4.5	142.4	73.1	64.5	155	0.34	1.06	28.1	》10	25
4.47	143.2	72.5	17.7	156.8	0.57	3.91	《10	0.6	5
3.7	136.9	50.9	77.7	183	0.44	《0.1	45	》10	47
3.78	136.8	69.6	112	271.8	0.81	0.27	91.8	》10	89
3.91	138.9	86.5	150	187.4	0.6	《0.1	82.8	》10	28
4.32	136.9	58.4	73.3	180.8	0.36	0.2	《10	《0.499	6
4.62	147.2	74.3	78.3	153.6	0.41	1.06	53.2	》10	16
3.21	140.8	43.5	105	213	0.56	《0.1	65.6	》10	79
4.22	146.5	55.9	56.8	149.7	0.44	0.18	《10	《0.499	14

	Cohort	sex(1,male;2,female)	age(year)	waist line(cm)	blood pressure(mmHg)	height(cm)	weight(kg)	BMI(kg/m2)	White blood cell（10^9/L）	Red blood cell（10^12/L	Hemoglobin(g/L)	Platelet（**10^9/L**）	Neutrophil（10^9/L）	Lymphocyte（10^9/L）	ALT(U/L)	AST(U/L)	albumin(g/L)	Total bilirubin(mmol/L)	Creatinine (μmol/L)
H002	Discovery	2	49	64	95/62	158.5	49.2	19.58	5	4.19	137	248	2.54	1.98	12	19	46.4	7.7	52
H003	Discovery	1	41	96	158/107	169.5	82.6	28.75	4.3	4.4	145	250	2.76	1.16	42	23	48.3	8.57	68
H004	Discovery	1	53	92.5	124/85	181	75.5	23.26	4.8	4.6	143	233	2.31	1.86	23	26	46.4	7.71	81
H005	Discovery	1	52	83	122/80	168.5	67.7	23.84	6.61	5.18	160	199	3.6	2.65	27	21	49.9	9.03	70
H008	Discovery	1	38	83	129/80	177.5	73.2	23.23	4.71	5.83	182	187	2.64	1.81	23	22	53.1	11.2	85
H009	Discovery	1	44	93	122/79	167	74.5	26.71	5.69	5.55	161	232	3.85	1.45	45	29	50	7.08	70
H010	Discovery	2	45	70	116/68	164.5	58.7	21.69	4.8	3.73	87	349	2.85	1.45	10	19	44.2	8	57
H011	Discovery	2	37			168.5	70.2	24.725	4.33	4.06	114	300	2.79	1.27	11	16	45.6	4.57	47
H013	Discovery	1	42	96	143/88	165	80.8	29.68	6	5	155	246	2.96	2.32	21	20	49.3	13.45	65
H015	Discovery	1	35	73	109/65	165	54.8	20.13	4.2	5.05	157	169	2.22	1.56	17	22	49	11.8	72
H017	Discovery	1	61	91	141/83	171	74.2	25.38	7.46	5.31	167	333	4.33	2.51	27	19	44.6	11.67	76
H019	Discovery	2	37	71	113/72	167.5	62.7	22.35	4.85	4.54	138	173	2.41	2.05	18	18	47.4	11.48	57
H020	Discovery	1	50	101	145/98	170.5	84.3	29	7.8	4.95	151	229	4.88	2.25	52	30	46.7	12.5	80
H021	Discovery	1	35	89	118/74	179	81.7	25.5	6.69	4.91	161	253	4.65	1.49	26	21	46.2	10	62
H023	Discovery	1	37	97	109/76	177	91.6	29.24	7.8	4.99	147	243	4.91	2.31	24	22	51.1	18.74	86
H024	Discovery	2	52	77	137/78	160	60.2	23.52	5	4.82	82	554	3.03	1.33	14	15	41.8	5.7	36
H025	Discovery	2	57	85	130/69	147.5	54	24.82	6.5	4.44	134	338	3.57	2.39	33	34	47.3	10.8	55
H027	Discovery	2	56	71	122/70	161.5	53.4	20.47	3.9	4.55	137	252	1.49	1.98	23	26	44.5	5.9	54
H028	Discovery	2	39	72	95/55	156	55.9	22.97	3.35	4.4	128	199	2.11	0.88	13	20	47.2	7.1	58
H029	Discovery	1	39	95	127/86	167.5	83.8	29.87	6.43	4.82	149	187	4.08	1.75	12	18	47.8	9.1	87
H032	Discovery	2	58	77	153/99	162.5	57.6	21.81	5.29	4.32	130	264	3.02	1.89	13	20	45.9	6.44	55
H034	Discovery	1	45	82	111/60	161.5	61	23.39	5.96	5.04	157	286	3.25	1.92	24	24	52.1	18.45	71
H035	Discovery	2	46	87	126/72	158.5	65.6	26.11	6.62	4.8	143	299	4.61	1.59	19	25	46.9	7.57	64
H036	Discovery	2	34	71	131/65	176	71.4	23.05	2.97	3.98	91	278	1.4	1.34	23	20	45.3	8.45	56
H039	Discovery	1	53	86	139/92	155.5	64	26.47	6.7	4.85	154	262	3.82	2.26	35	25	52.1	13.19	67
H040	Discovery	2	50	95	142/82	166.5	79	28.5	6.76	4.34	125	304	4	2.05	22	31	42.1	15.35	61
H041	Discovery	1	61	96	147/97	168	79.9	28.52	4.75	5.08	155	190	2.81	1.44	16	18	43.3	26.04	81
H044	Discovery	1	37	94	111/72	179	86.4	26.97	5.99	5.09	161	229	3.19	2.31	19	16	44.6	10.68	76
H048	Discovery	1	50	98	120/77	172	86.3	29.17	7.7	5.2	162	272	4.89	2.02	19	18	46.2	6.62	73
H049	Discovery	2	52	75	134/78	158	59.6	23.87	6.42	4.86	144	313	3.51	2.4	12	18	42.7	10.29	54
H051	Discovery	1	43	87	140/101	175.5	70.9	23.02	7.3	4.96	155	210	4.52	1.91	41	35	51.7	9.1	64
H053	Discovery	1	41	90	128/88	176.5	80.5	26.1	6	5.46	172	303	3.53	2.08	23	21	43.6	12.6	72
H057	Discovery	2	65	108	171/83	154	79.8	33.65	5.94	4.8	129	239	3.39	1.97	14	19	48.7	7.73	49
H058	Discovery	1	48	91	124/86	168.5	68.7	24.2	8.31	5.51	169	233	5.14	2.47	36	28	50.7	15.1	53
H059	Discovery	2	36	84	79/44	159	60.1	23.77	4.21	4.42	140	211	2.31	1.58	16	17	48.4	14.31	55
H060	Discovery	2	38	82	123/80	159	78.5	31.05	7.2	4.11	127	361	4.59	2.04	13	18	45.6	7.8	48
H061	Discovery	1	64	98	122/78	161.5	76.8	29.45	7.64	4.68	136	269	4.85	2.09	23	21	49.4	11.28	84
H062	Discovery	2	35	68	109/70	165.5	58.3	21.28	6.31	4.26	128	238	3.95	1.83	16	18	46.5	4.3	51
H063	Discovery	1	56	80	101/62	166	61.5	22.32	4.5	4.44	143	134	2.25	1.76	43	29	47.9	9	64
H064	Discovery	2	66	86	135/61	164	56.9	21.16	6.01	3.96	124	210	2.99	2.25	22	23	46.4	5.7	70
H065	Discovery	2	34	68	101/59	168	54.7	19.38	4.28	4.44	138	252	2.17	1.68	17	35	46.9	9.43	58
H066	Discovery	2	39	72	96/53	167	55.7	19.97	5.67	4.67	138	247	3.49	1.63	10	14	50	13.12	60
H067	Discovery	1	63	92	111/67	170	80.2	27.75	5.3	5.07	154	266	2.82	2	22	19	47.3	12	71
H070	Discovery	2	58	83	104/66	161	62.4	23.92	5.13	3.97	167	165	2.12	1.53	22	20	51.6	18.2	74
H071	Discovery	2	57	89	115/73	154	58	24.46	4.7	3.86	122	186	3.09	1.17	11	13	46.6	12.31	56
H073	Discovery	1	42	90	132/77	172	81	27.38	6	5.1	162	232	3	2.31	22	21	44.5	19.86	66
H074	Discovery	1	44	88	124/74	161	68	26.23	7.2	5.77	175	182	4.26	2.12	28	22	50.5	11.27	100
H075	Discovery	1	37	81	116/76	179	72	22.47	4.42	5.08	162	141	2.34	1.25	22	29	48	14.96	84
H076	Discovery	2	35	68	109/72	162	50	19.05	4	4.5	134	192	2.09	1.63	15	17	51.9	14.4	52
H078	Discovery	1	37	79	141/94	174	57.5	19.16	5.65	5.63	165	163	3.44	1.65	13	18	50.7	15.54	80
H079	Discovery	2	44	77	150/97	154	62	26.14	6.11	4.26	127	284	3.34	1.83	30	28	48.1	19.77	53
H083	Discovery	1	40	96	171/106	180	92	28.7	5.34	4.95	160	257	3.27	1.65	17	16	52.9	28.99	75
H084	Discovery	1	51	90	151/93	162	67	25.91	6.94	5.59	168	235	3.62	2.45	35	32	54.2	17.26	79
H086	Discovery	1	52	89	111/71	177	81	25.85	5.87	4.37	147	287	3.73	1.66	18	24	50.2	12.75	88
H088	Discovery	1	62	100	184/96	164	77	28.63	5.2	4.94	141	168	3.16	1.61	18	24	50.5	14.27	76

H089	Discovery	1	45	93	137/96	172	81	27.38	6.57	5.33	163	333	4.14	1.56	20	21	49.7	17.6	81
H090	Discovery	2	62	94	147/74	154	63	26.56	5.39	5.15	146	208	3.09	1.68	32	24	49.8	15	51
H091	Discovery	2	52	71	99/59	160	53	20.7	5.06	4.71	145	235	2.65	1.95	7	20	45.3	7.99	50
H092	Discovery	1	34	98	136/73	169	86.9	30.46	7.69	5.31	160	257	5.08	2.1	19	16	52.5	6.78	82
H093	Discovery	1	38	90	109/69	172	79	26.7	6.52	5.04	159	201	4.25	1.77	18	18	51	10.88	74
H094	Discovery	1	44	78	133/96	164	65	24.17	5.81	5.11	169	218	3.71	1.66	22	24	53	9.58	78
H096	Discovery	1	35			170	70	24.221	3.81	4.94	156	163	2.29	1.23	23	17	48.6	6.73	64
H097	Discovery	1	58	94	128/77	171	82	28.04	6.91	5.02	156	305	4.56	1.73	29	23	51.4	22.1	85
H098	Discovery	2	41	70	83/52	160	50	19.53	4.91	4.09	135	227	2.94	1.61	9	16	47.4	12.37	55
H099	Discovery	1	36	84	114/74	171	69	23.6	6.45	5.19	167	283	3.67	2.3	21	25	49.5	21.19	69
H100	Discovery	2	34	81	122/75	156	66	27.12	7.09	4.7	144	212	4.67	1.52	4	18	44.9	5.88	44
H102	Discovery	1	74	93	142/76	176	78	25.18	8.16	4.82	151	241	5.14	2.37	14	20	47.1	11.16	73
H104	Discovery	2	33	73	105/66	154	53	22.35	7.54	4.47	142	281	4.83	2.22	10	17	49	6.7	65
H107	Discovery	1	38	100	114/73	166	85	30.85	6.29	5.16	158	230	3.14	2.65	31	22	49.8	12.45	81
H108	Discovery	2	39	87	124/82	158	82	32.85	10.24	4.62	142	220	7.27	2.32	13	17	48.1	10.5	56
H109	Discovery	1	37	112	113/72	180	118	36.42	5.43	5.22	150	246	3.02	1.83	39	23	47.1	6.4	73
H110	Discovery	1	35	103	117/76	169	90	31.51	5.95	4.87	151	221	3.43	1.98	36	21	46.2	8	73
H113	Discovery	1	42	93	144/92	170.5	80.6	27.73	5.04	5.47	167	192	2.75	1.75	56	35	51.7	12.83	72
H114	Discovery	1	44	99	128/86	176	86	27.76	4.81	5.28	152	563	2.59	1.65	23	18	48.7	5.54	76
H116	Discovery	2	38	70	97/60	165	52	19.1	4.15	4.16	122	149	2.61	1.21	9	18	49.4	6.85	53
H118	Discovery	1	45			175	80	26.122	4.62	5.04	157	206	2.85	1.37	17	18	44.5	7.54	47
H120	Discovery	1	50	87	158/98	173	75.2	25.13	6.53	4.83	147	256	3.4	2.52	31	23	44.8	8.2	79
H122	Discovery	1	42	100	125/75	181	92	28.08	5.55	5.14	156	298	2.9	2.25	25	15	48.7	14.1	83
H123	Discovery	2	51	73	104/71	155	57	23.73	4.12	4.14	131	185	2.11	1.68	26	22	45.3	9.7	61
H124	Discovery	1	53	100	143/99	165	86	31.59	5.74	4.71	150	240	3.17	2.17	20	22	48.2	23.2	79
H125	Discovery	1	43	84	107/68	176	69	22.28	6.57	4.82	154	162	3.89	1.91	17	23	50	13.51	81
H126	Discovery	1	53	86	133/89	175	78	25.47	6.35	5.48	162	260	4.23	1.42	26	31	47.3	11.6	90
H127	Discovery	2	47	78	116/75	162	57	21.72	5.7	4.71	146	288	3.82	1.33	10	15	52.8	12.84	48
H130	Discovery	1	52	85	117/67	173	74	24.73	4.73	4.67	150	257	2.98	1.46	14	20	45.4	8.92	78
H131	Discovery	1	35	87	118/75	169	66	23.11	9.27	5.54	155	261	4.94	3.58	19	18	48	19.43	90
H133	Discovery	2	47	77	118/76	162	55.3	21.07	5.04	4.32	140	179	2.63	1.95	12	22	44.2	9.03	51
H137	Discovery	1	42	85	113/72	175	75	24.49	4.83	4.72	152	255	3.39	1.1	26	19	47.6	13.88	66
H139	Discovery	1	50	90	124/83	182	90	27.17	5.68	5.57	171	302	2.39	2.72	18	25	52.8	11.62	90
H141	Discovery	2	45	70	102/57	164	57.7	21.56	4.03	3.88	119	245	2.4	1.17	15	21	50.1	5.69	51
H142	Discovery	1	47	89	133/84	177	75	23.94	4.32	5.47	167	196	2.6	1.31	30	21	49	22.6	79
H146	Discovery	2	42	73	101/62	161	56.9	21.99	4.52	4.11	126	205	2.77	1.42	12	16	42.6	4.62	60
H147	Discovery	1	47	97	143/97	167	79	28.33	5.95	5.07	157	217	3.91	1.53	24	24	45.9	13	110
H149	Discovery	1	44	93	129/67	182	90.4	27.29	5.43	4.65	154	280	3.39	1.58	11	17	50.9	10.26	89
H150	Discovery	2	52	76	139/79	165	54	19.83	4.86	4.59	138	278	2.35	2.27	9	16	48.6	6.05	50
H006	Validation	1	50	75	146/96	161	59.5	22.95	6.7	4.79	154	283	3.54	2.21	17	19	49.5	10.5	63
H014	Validation	2	57	91	142/77	154.5	72.4	30.33	6.7	3.77	129	397	4.33	1.83	26	26	48.2	8.12	50
H016	Validation	2	58	92	157/86	159	71.7	28.36	5.5	4.8	137	289	2.45	2.56	26	28	47	8.9	44
H018	Validation	2	59	82	115/71	159.5	64.4	25.31	4.74	4.23	137	262	2.58	1.88	77	41	47.1	13.98	69
H026	Validation	2	35	80	123/77	167	71.2	25.53	5.14	4.21	133	273	2.67	1.64	12	15	43.7	3.4	70
H030	Validation	2	40			160	60.2	23.516	5.05	4.18	132	278	2.56	2.38	24	28	44.2	9.14	76
H031	Validation	1	44	92	134/84	195	97.9	25.75	12.9	4.93	157	289	8.23	4.09	53	27	56.7	10.39	82
H033	Validation	1	70	72	214/96	162.5	54.7	20.71	4.84	4.37	133	244	3.28	1.04	11	20	45.4	6.4	94
H038	Validation	1	42	85	129/75	171.5	69	23.46	4.26	4.98	152	174	2.61	1.19	14	16	47.1	8.6	80
H042	Validation	2	44	69	116/63	165.5	49.1	17.93	4.34	3.84	124	249	2.07	1.89	12	20	42.4	8.61	51
H043	Validation	1	35	90	116/72	172.5	79.6	26.75	6.7	5.04	156	324	3.87	2.35	19	20	49.6	10.5	91
H045	Validation	2	55	86	118/83	160	66.2	25.86	5.8	4.42	142	272	3.76	1.57	22	19	49.7	16.35	53
H046	Validation	1	36	90	129/76	183	82.8	24.72	4.56	5.17	156	185	2.6	1.5	16	19	50.1	13.82	88
H047	Validation	1	60	82	112/73	171	67.4	23.05	8.5	4.93	157	265	5.82	1.73	14	20	48.9	16.7	89
H050	Validation	1	33	--	158/103	169.5	79.8	27.78	5.67	5.06	152	303	2.79	2.36	36	22	50.8	14.81	69
H052	Validation	1	66	93	108/70	171.5	75.8	25.77	4.71	4.78	161	130	3.12	1.17	11	16	48.4	24.96	84
H054	Validation	1	39	119	123/79	183	134.4	40.85	7.5	4.63	145	251	4.68	2.03	38	21	45.7	9.27	97
H055	Validation	2	36	75	97/54	168	63.7	22.57	4.1	4.11	125	235	2.57	1.06	9	15	46.3	12.45	51
H056	Validation	1	66	83	138/59	171.5	65.1	22.13	6.2	3.72	80	365	4.59	1.15	15	17	45.3	8.85	48

H068	Validation	1	34	71	141/83	182	59	17.81	3.97	5.13	167	165	2.12	1.53	22	20	51.6	18.2	74
H069	Validation	2	58	83	127/72	170	62	21.45	4.54	4.75	145	145	3.06	1.13	25	27	48.1	8.36	87
H072	Validation	1	34	108	124/75	183	109.5	32.85	6.22	4.85	152	284	3.12	2.34	31	18	47.7	13.8	75
H077	Validation	1	48	90	142/89	169	74.3	26.01	6.38	4.29	99	267	3.87	1.35	14	18	50.7	10.24	96
H080	Validation	2	41	86	102/70	158	63.9	26.04	5.52	4.85	155	228	3.76	1.44	14	13	46.4	13.2	63
H081	Validation	2	52	74	114/76	161	54	20.83	6.01	4.11	125	293	3.8	1.82	13	19	47.6	10.1	59
H082	Validation	1	52	78	129/77	163	60.1	22.62	6.16	5.35	165	210	3.99	1.77	23	27	49	8.36	60
H085	Validation	1	48	89	105/80	174	69	22.79	7.82	5.27	167	227	4.98	2.24	36	15	49.6	5.11	62
H087	Validation	1	40	88	117/70	182	79	23.85	4.48	4.81	156	217	2.56	1.43	15	19	44.5	19.32	77
H095	Validation	1	34	88	112/73	174	76	25.1	7.27	5.36	160	302	4.11	2.4	23	19	52.2	10.17	100
H101	Validation	1	48	100	116/75	169	80	28.01	7.08	5.13	161	276	4.25	2.21	21	21	41.9	8.47	73
H103	Validation	1	52	88	120/71	166	67	24.31	8.07	5.16	158	252	4.7	2.8	20	22	45.8	11.59	94
H105	Validation	2	37	71	108/62	162	54	20.58	4.66	3.89	120	168	2.92	1.42	10	20	44.2	15.81	74
H106	Validation	1	57	84	114/56	160	67	26.17	5.91	4.9	158	258	3.43	1.97	26	26	46.2	11.32	68
H111	Validation	1	33	97	122/79	171	87	29.75	6.43	5.47	167	366	3.56	2.06	53	34	49.1	12.7	83
H112	Validation	1	42	87	124/77	172	76	25.69	4.87	4.65	147	179	3.35	1.02	28	21	45.3	11.2	89
H115	Validation	2	47	82	112/73	158	61	24.44	6.39	4.63	148	265	3.43	2.53	35	28	47.5	7.37	65
H117	Validation	2	52	75	103/64	161	55.9	21.6	3.47	4.54	141	208	1.45	1.78	13	17	50.1	14.97	67
H119	Validation	1	46	82	111/86	172	68	22.99	5.08	4.96	162	212	2.85	1.71	47	30	52.1	13.09	88
H121	Validation	1	50	85	136/78	176	69	22.28	7.42	5.06	149	239	3.5	3.36	22	21	47.3	10.4	75
H129	Validation	1	46	85	134/85	175	71	23.18	7.79	4.62	148	227	5.54	1.82	23	21	46.3	12.42	72
H132	Validation	2	47	68	119/71	167	52	18.65	5.82	4.93	120	191	4.02	1.32	11	20	46.9	5.41	56
H135	Validation	2	48	77	115/70	166	66	23.95	4.38	4.94	147	198	2.37	1.57	32	31	51.2	8.77	61
H136	Validation	2	36	69	89/59	158	54	21.63	4.96	4.21	133	210	3.04	1.52	22	20	48.3	11.5	58
H138	Validation	1	52	85	123/79	166	74	26.85	7.79	4.94	150	215	4.87	2.22	19	23	49.1	10.02	91
H140	Validation	1	36	82	121/74	166	73	26.49	4.64	4.77	151	209	2.84	1.38	23	22	47.1	10.33	85
H143	Validation	1	38	88	123/79	170	75	25.95	5.43	4.55	148	195	3.42	1.6	21	27	46.6	12.36	74

Table S2. The detailed values of oral microbial diversity index and observed OTUs

sample	reads	label	ace	chao	shannon	simpson	coverage	observed_otus
H002		0.97	393.001194	386.947368	3.626317	0.069363	0.998461	0
H003		0.97	313.516709	311.028571	3.663356	0.052214	0.998255	0
H004		0.97	379.303695	323.62069	3.077652	0.084126	0.998423	0
H005		0.97	288.457977	293.12	3.547417	0.052441	0.99874	0
H008		0.97	400.493814	346	3.304988	0.070381	0.998527	0
H009		0.97	405.101998	420.181818	3.872127	0.049142	0.998252	0
H010		0.97	375.242698	376.636364	3.852834	0.044048	0.998519	0
H011		0.97	404.467414	398.5	4.066462	0.038751	0.998631	0
H013		0.97	284.698688	280.4	3.127392	0.112333	0.997889	0
H015		0.97	271.137015	269.545455	3.521795	0.056429	0.998968	0
H017		0.97	299.069244	311.043478	2.767923	0.221506	0.998347	0
H019		0.97	281.879763	284	3.393171	0.064752	0.998824	0
H020		0.97	360.201795	357.871795	3.645244	0.051351	0.998874	0
H021		0.97	397.602672	408.384615	4.14723	0.033005	0.99892	0
H023		0.97	301.887509	300.24	3.743283	0.043285	0.998045	0
H024		0.97	382.063152	399.576923	3.323818	0.149996	0.997563	0
H025		0.97	547.376324	554.454545	3.840433	0.049574	0.998175	0
H027		0.97	482.094826	486.5	3.848147	0.071178	0.997347	0
H028		0.97	393.087759	417.964286	4.087172	0.032548	0.997387	0
H029		0.97	541.002141	555.227273	4.325284	0.030273	0.997505	0
H032		0.97	501.369451	502.25	4.566539	0.021108	0.99819	0
H034		0.97	434.777132	462.285714	4.169537	0.033686	0.998035	0
H035		0.97	384.965464	382.029412	3.643908	0.045131	0.998614	0
H036		0.97	215.888007	207.681818	3.371759	0.078175	0.997687	0
H039		0.97	288.185174	285.724138	3.502752	0.054795	0.998624	0
H040		0.97	301.727777	305	3.923017	0.034552	0.998311	0
H041		0.97	238.258229	254.176471	3.17776	0.073995	0.998507	0
H044		0.97	444.927194	379.885714	3.328979	0.06756	0.997596	0
H048		0.97	311.287961	327.869565	3.372457	0.068753	0.998331	0
H049		0.97	367.347252	376.785714	3.622437	0.054288	0.998357	0
H051		0.97	468.868811	467.857143	3.949637	0.040923	0.998654	0
H053		0.97	289.49602	333.588235	4.020266	0.040995	0.99346	0
H057		0.97	313.309177	298.885714	3.431635	0.081226	0.998723	0
H058		0.97	321.564349	330.956522	4.527754	0.022796	0.996294	0
H059		0.97	280.261418	293	4.182471	0.031467	0.996957	0
H060		0.97	374.905541	384.5	3.947248	0.052507	0.99864	0
H061		0.97	224.75926	218.884615	3.121565	0.1011	0.998477	0
H062		0.97	417.486084	436	4.084288	0.03851	0.998871	0
H063		0.97	310.643432	320.84	3.131096	0.128995	0.998753	0
H064		0.97	373.367253	335.55	3.154969	0.095489	0.998244	0
H065		0.97	372.416634	369	3.635714	0.05266	0.999002	0
H066		0.97	371.75157	374.7	3.937268	0.070356	0.998527	0
H067		0.97	272.95615	268.75	3.155743	0.095945	0.998199	0
H070		0.97	321.407607	297.555556	3.243833	0.080268	0.997909	0
H071		0.97	354.914726	346.733333	3.790552	0.042205	0.998068	0
H073		0.97	332.25594	336.535714	3.668108	0.055766	0.998584	0
H074		0.97	293.861752	255.04	3.001027	0.114752	0.998343	0
H075		0.97	359.345533	360.833333	3.326184	0.077438	0.998351	0
H076		0.97	331.645093	340.913043	3.51685	0.073197	0.998418	0
H078		0.97	280.444414	273.285714	3.754298	0.041656	0.998065	0
H079		0.97	237.105437	236.454545	3.442072	0.064504	0.998557	0
H083		0.97	340.914685	339.088235	3.499005	0.063592	0.998783	0
H084		0.97	362.04745	374.032258	3.143717	0.104884	0.998426	0
H086		0.97	325.179036	335.12	3.626195	0.064753	0.998515	0
H088		0.97	316.966125	316.333333	3.622894	0.06443	0.999097	0
H089		0.97	274.974536	292.75	3.615629	0.06411	0.998472	0
H090		0.97	228.457026	222.130435	3.395911	0.055679	0.997885	0
H091		0.97	373.87658	369.675676	4.135947	0.033913	0.998009	0
H092		0.97	298.155881	304.04	3.790043	0.043745	0.997976	0
H093		0.97	247.896194	236.241379	3.340329	0.069168	0.998759	0
H094		0.97	289.016516	298.5	3.615211	0.047918	0.99806	0
H096		0.97	385.064338	379.4	3.472033	0.070506	0.998359	0
H097		0.97	260.194542	251.12	3.21422	0.121316	0.997963	0
H098		0.97	300.296701	304.344828	3.655016	0.040085	0.99892	0
H099		0.97	374.872769	375.84375	3.97957	0.047036	0.998693	0
H100		0.97	289.165512	283.40625	3.410902	0.062577	0.998568	0
H102		0.97	277.718023	299.333333	3.476672	0.071915	0.998092	0
H104		0.97	377.359279	372.682927	3.761414	0.045326	0.998698	0
H107		0.97	377.160959	385.384615	4.001656	0.037103	0.998864	0
H108		0.97	319.499689	318.757576	3.467445	0.071318	0.99842	0
H109		0.97	326.259917	334.576923	3.451375	0.06592	0.998667	0
H110		0.97	417.948049	404.714286	3.889648	0.042934	0.998228	0
H113		0.97	380.291542	378.447368	3.956422	0.041643	0.998902	0
H114		0.97	311.549281	318.5	3.47346	0.055156	0.999156	0
H116		0.97	301.911709	304.037037	3.666083	0.044206	0.99902	0
H118		0.97	362.04503	363.675676	3.609565	0.054361	0.998474	0
H120		0.97	354.692422	350.447368	3.235137	0.093303	0.99808	0
H122		0.97	368.914518	361.578947	3.261097	0.1096	0.99873	0

H123		0.97	415.99377	419.384615	3.671256	0.055758	0.997753	0
H124		0.97	485.15303	455.34375	3.846044	0.04667	0.997866	0
H125		0.97	343.094753	353.777778	4.170402	0.031298	0.995508	0
H126		0.97	364.951335	379.04	4.084165	0.035867	0.998743	0
H127		0.97	386.60337	312.357143	3.805426	0.037085	0.997199	0
H130		0.97	270.90102	266.433333	3.326842	0.096151	0.998529	0
H131		0.97	380.521993	375.108108	3.999678	0.043138	0.998986	0
H133		0.97	329.157167	342.1	3.476503	0.070265	0.998251	0
H137		0.97	306.443441	318.291667	3.392412	0.090206	0.998342	0
H139		0.97	301.544503	299.5	3.288296	0.072273	0.999027	0
H141		0.97	294.255167	289.085714	3.795019	0.036265	0.999352	0
H142		0.97	263.846981	264.272727	3.747345	0.045653	0.9991	0
H146		0.97	285.111615	289.384615	3.266563	0.07823	0.998611	0
H147		0.97	246.318756	242.75	3.106195	0.128764	0.99816	0
H149		0.97	320.53772	336.875	3.139284	0.099069	0.99824	0
H150		0.97	232.416673	229.416667	3.419527	0.060584	0.998623	0
CC01		0.97	93.548053	184.5	2.116231	0.203338	0.996754	0
CC02		0.97	224.855744	185.928571	2.786594	0.099207	0.999113	0
CC05		0.97	99.877718	103.5	2.369005	0.170926	0.999519	0
CC06		0.97	135.172151	134.214286	2.839912	0.087129	0.999647	0
CC08		0.97	160.396095	185.5	1.840324	0.212807	0.999162	0
CC09		0.97	156.810259	165.111111	3.123665	0.073991	0.999113	0
CC12		0.97	206.284454	172	1.298597	0.476826	0.999156	0
CC16		0.97	359.089063	361.756757	2.389343	0.195877	0.998976	0
CC17		0.97	420.509723	435.193548	3.744352	0.052089	0.998546	0
CC20		0.97	330.86979	271.772727	2.446063	0.235701	0.998861	0
CC21		0.97	227.783539	285.625	3.21246	0.074705	0.999013	0
CC24		0.97	209.661513	206.3	3.076649	0.112787	0.999435	0
CC25		0.97	148.734383	142.4	3.192787	0.077144	0.997288	0
CC26		0.97	253.783839	232.058824	1.910883	0.38078	0.998057	0
CC27		0.97	274.347223	273.2	2.876784	0.198279	0.999177	0
CC28		0.97	193.953264	206.571429	2.603341	0.172917	0.997468	0
CC29		0.97	233.222162	244.4	3.082322	0.090328	0.999191	0
CC30		0.97	184.784746	193.363636	3.336873	0.057739	0.998894	0
CC31		0.97	158.976362	154.473684	1.749152	0.416689	0.998127	0
CC33		0.97	159.380981	163.714286	1.962792	0.283838	0.997912	0
CC34		0.97	236.859279	239	3.742065	0.039216	0.999163	0
CC35		0.97	116.518448	119	1.351468	0.471221	0.997786	0
CC36		0.97	394.21791	388	3.415332	0.066375	0.998826	0
CC37		0.97	184.98587	185.588235	3.218049	0.083311	0.997825	0
CC39		0.97	219.480762	216.5	1.867963	0.341797	0.998802	0
CC42		0.97	76.705764	74.875	2.102414	0.204233	0.999211	0
CC43		0.97	280.249145	279.322581	2.582549	0.108935	0.999238	0
CC44		0.97	209.654719	221	3.200669	0.081188	0.997658	0
CC45		0.97	252.589181	250.217391	3.074769	0.167906	0.998327	0
CC46		0.97	204.666533	213.235294	2.76316	0.151468	0.995806	0
CC48		0.97	234.370303	172.045455	2.380822	0.130127	0.999063	0
CC52		0.97	374.730916	370.025	3.581393	0.064019	0.999003	0
CC54		0.97	162.077282	157.368421	3.188146	0.077193	0.998525	0
CC55		0.97	186.911925	157.090909	2.674184	0.094898	0.999161	0
CC57		0.97	373.583346	376.527778	3.546245	0.082613	0.998426	0
CC58		0.97	174.956849	183.909091	2.683758	0.159488	0.9985	0
CC60		0.97	174.817909	173.789474	2.376175	0.279075	0.998155	0
CC62		0.97	156.291758	184	2.442547	0.170896	0.998916	0
CC63		0.97	186.963668	179.954545	2.398206	0.252727	0.999197	0
CC64		0.97	162.797151	151.857143	0.534513	0.823356	0.999607	0
CC65		0.97	164.692947	159	1.565839	0.523652	0.9994	0
CC67		0.97	194.157619	206.071429	2.157828	0.350896	0.998814	0
CC68		0.97	153.334292	158.25	2.548775	0.175965	0.998262	0
CC69		0.97	204.175102	172.565217	2.524762	0.164252	0.998912	0
CC70		0.97	217.885295	218	2.544308	0.216642	0.999238	0
CC71		0.97	171.293375	166	2.951642	0.096332	0.999509	0
CC72		0.97	228.824827	229	3.467724	0.062081	0.998962	0
CC73		0.97	174.086289	170.555556	2.322144	0.218192	0.99919	0

Table S3 . The abundance and composition at the phylum level of each sample												
ID	H139	H125	H066	H142	H053	H130	H091	H059	H058	H079	H041	H100
Firmicutes	0.47704	0.411526	0.392201	0.385085	0.375936	0.360043	0.337077	0.33312	0.331194	0.323297	0.323117	0.3129
Bacteroidota	0.177443	0.438124	0.232806	0.261483	0.410692	0.45784	0.339395	0.322307	0.359324	0.448986	0.398824	0.438689
Proteobacteria	0.188181	0.023705	0.2235	0.122514	0.129597	0.030054	0.095654	0.207375	0.130227	0.023281	0.01361	0.067179
Fusobacteriota	0.025813	0.070223	0.062516	0.130685	0.003004	0.065194	0.082197	0.032023	0.078111	0.105799	0.159473	0.078751
Actinobacteriota	0.103556	0.028292	0.065895	0.077126	0.076099	0.045344	0.083637	0.09464	0.077322	0.036597	0.048966	0.032522
Patescibacteria	0.018377	0.022289	0.004501	0.017981	0.002452	0.034853	0.036698	0.00616	0.010143	0.040615	0.051255	0.056033
Campilobacterota	0.004628	0.002317	0.006889	0.004747	0	0.00337	0.018767	0.001257	0.007136	0.020671	0.004755	0.01351
Spirochaetota	0.003476	0	0.009377	3.06E-04	0	0.002936	0.006175	4.31E-04	0.003827	5.73E-04	0	3.67E-04
Synergistota	0.001319	0	0.001028	4.70E-05	0	3.66E-04	2.70E-05	0	0	1.81E-04	0	4.90E-05
Desulfobacterota	1.21E-04	0.001362	4.83E-04	0	0.001743	0	0	7.24E-04	0.001189	0	0	0
Deferribacterota	0	2.22E-04	0	0	0	0	0	0	0	0	0	0
Cyanobacteria	4.60E-05	6.52E-04	7.10E-05	2.60E-05	1.14E-04	0	3.31E-04	3.28E-04	5.03E-04	0	0	0
Verrucomicrobiota	0	0.001288	1.49E-04	0	3.63E-04	0	0	0.001635	0.001024	0	0	0
Bacteria_unclassified	0	0	0	0	0	0	0	0	0	0	0	0
Deinococcota	0	0	4.34E-04	0	0	0	0	0	0	0	0	0
Chloroflexi	0	0	1.50E-04	0	0	0	4.20E-05	0	0	0	0	0
Elusimicrobiota	0	0	0	0	0	0	0	0	0	0	0	0
Planctomycetota	0	0	0	0	0	0	0	0	0	0	0	0

H073	H011	H141	H029	H133	H126	H034	H019	H032	H003	H039	H008	H028	H116
0.312389	0.29942	0.293889	0.293209	0.28461	0.283668	0.283423	0.276334	0.271776	0.271162	0.269851	0.269578	0.269181	0.265403
0.461071	0.402131	0.328786	0.429676	0.250099	0.400279	0.315383	0.511539	0.348819	0.327128	0.391141	0.516911	0.33312	0.367904
0.058046	0.077161	0.159107	0.167919	0.298065	0.092509	0.171889	0.02855	0.048342	0.259419	0.039102	0.041933	0.218414	0.058162
0.065647	0.05829	0.110149	0.036238	0.064477	0.107691	0.049147	0.103185	0.177967	0.071932	0.252758	0.088013	0.065974	0.225949
0.050572	0.146411	0.09681	0.033568	0.067214	0.040337	0.037991	0.044514	0.10273	0.064013	0.025239	0.045436	0.024453	0.039309
0.034693	0.001498	0.006124	0.018047	0.032517	0.020825	0.090604	0.011901	0.031222	0.001623	0.012754	0.025117	0.026462	0.035007
0.010112	0.012987	0.003973	0.013493	0.002004	0.010462	0.016585	0.023237	0.012595	0.004475	0.007304	0.012643	0.050191	0.00739
0.00661	0.001404	9.50E-04	0.002026	5.95E-04	0.039478	0.001065	7.40E-04	0.002932	1.08E-04	9.10E-04	3.25E-04	2.89E-04	8.76E-04
7.93E-04	2.30E-05	1.31E-04	8.06E-04	2.90E-04	0.004209	1.62E-04	0	1.15E-04	0	7.03E-04	2.20E-05	3.50E-05	0
3.50E-05	3.16E-04	8.10E-05	0.003626	6.50E-05	4.49E-04	0.007217	0	0.002514	3.10E-05	2.38E-04	0	0.002723	0
0	0	0	9.94E-04	0	0	0.026348	0	9.64E-04	0	0	0	0.008999	0
0	4.00E-05	0	3.40E-04	0	7.30E-05	1.86E-04	0	2.40E-05	3.20E-05	0	0	1.59E-04	0
0	3.19E-04	0	3.00E-05	6.40E-05	0	0	0	0	7.70E-05	0	2.20E-05	0	0
0	0	0	0	0	0	0	0	0	0	0	0	0	0
0	0	0	0	0	0	0	0	0	0	0	0	0	0
3.20E-05	0	0	2.80E-05	0	2.00E-05	0	0	0	0	0	0	0	0
0	0	0	0	0	0	0	0	0	0	0	0	0	0
0	0	0	0	0	0	0	0	0	0	0	0	0	0

H124	H107	H110	H010	H062	H131	H104	H098	H036	H122	H071	H150	H005	H070
0.263537	0.261084	0.258182	0.255584	0.252126	0.251721	0.248837	0.248459	0.247296	0.245459	0.244924	0.237854	0.233604	0.229274
0.313804	0.284064	0.240961	0.36052	0.215841	0.238546	0.290922	0.323015	0.463436	0.186635	0.428212	0.316358	0.280628	0.159167
0.296388	0.292453	0.267332	0.249578	0.330492	0.157361	0.325905	0.198415	0.097281	0.450923	0.085909	0.29518	0.148435	0.428049
0.061198	0.056559	0.141026	0.051337	0.089551	0.234206	0.065135	0.104035	0.138536	0.073506	0.146485	0.059158	0.232402	0.130789
0.03172	0.086572	0.068319	0.055976	0.071011	0.065439	0.036062	0.043607	0.034235	0.02331	0.021283	0.056028	0.065194	0.037959
0.013188	0.009891	0.014911	0.017975	0.029712	0.036987	0.027192	0.062314	0.013283	0.009375	0.043597	0.028932	0.015552	0.005236
0.012593	0.008477	0.007856	0.007814	0.005809	0.009962	0.002953	0.019103	0.00558	0.007998	0.02728	0.006163	0.023168	0.008809
0.006937	5.46E-04	0.001061	1.99E-04	0.004367	0.005245	0.002509	9.81E-04	2.34E-04	0.002706	0.002104	2.20E-04	9.02E-04	7.17E-04
4.44E-04	5.00E-05	2.34E-04	0	8.01E-04	4.36E-04	2.66E-04	0	5.90E-05	3.90E-05	2.06E-04	6.50E-05	9.90E-05	0
1.47E-04	7.10E-05	4.80E-05	4.91E-04	1.04E-04	1.20E-05	8.40E-05	0	6.00E-05	2.60E-05	0	0	0	0
0	0	0	0	0	0	0	0	0	0	0	0	0	0
0	9.70E-05	4.50E-05	1.50E-04	9.70E-05	4.10E-05	2.20E-05	7.10E-05	0	0	0	0	1.60E-05	0
2.20E-05	1.36E-04	2.50E-05	2.93E-04	8.90E-05	2.00E-05	1.13E-04	0	0	2.30E-05	0	0	0	0
0	0	0	0	0	0	0	0	0	0	0	0	0	0
2.20E-05	0	0	8.30E-05	0	0	0	0	0	0	0	0	0	0
0	0	0	0	0	2.40E-05	0	0	0	0	0	0	0	0
0	0	0	0	0	0	0	0	0	0	0	4.20E-05	0	0
0	0	0	0	0	0	0	0	0	0	0	0	0	0

H021	H088	H149	H092	H061	H044	H051	H108	H065	H113	H024	H025	H127	H002
0.228357	0.22736	0.226428	0.224463	0.223741	0.223585	0.221098	0.219245	0.216923	0.216856	0.215876	0.214903	0.211388	0.210165
0.377562	0.300058	0.115525	0.400815	0.286153	0.201184	0.368775	0.305579	0.353687	0.445105	0.222518	0.317625	0.339568	0.288754
0.1277	0.322583	0.539183	0.158558	0.277395	0.255547	0.230732	0.340519	0.290508	0.19639	0.504691	0.26087	0.225321	0.312051
0.163159	0.076613	0.071282	0.150045	0.186581	0.186495	0.114068	0.066772	0.076033	0.057248	0.022637	0.115137	0.14012	0.068292
0.050076	0.046399	0.02969	0.013555	0.015713	0.116469	0.021662	0.025217	0.038205	0.03511	0.012051	0.016857	0.032577	0.074865
0.022627	0.012604	0.00517	0.037813	0.005854	0.005863	0.023988	0.030242	0.017356	0.041133	0.012266	0.059367	0.034155	0.024986
0.019739	0.005503	0.004948	0.008086	0.003488	0.009792	0.012368	0.009974	0.00468	0.006431	0.00499	0.008126	0.015717	0.016543
0.009288	0.005824	0.005021	0.004606	5.39E-04	9.76E-04	0.006064	0.002156	0.0024	0.00123	4.50E-05	0.006174	9.63E-04	0.003719
7.48E-04	0.00247	0.001768	0.001484	4.08E-04	8.90E-05	8.27E-04	1.13E-04	9.30E-05	0	0	1.95E-04	1.14E-04	5.27E-04
5.25E-04	5.60E-04	8.46E-04	5.41E-04	1.28E-04	0	2.12E-04	0	1.70E-05	7.50E-05	0.004552	5.00E-04	0	2.90E-05
0	0	0	0	0	0	0	0	0	0	3.33E-04	5.20E-05	0	0
0	0	3.80E-05	0	0	0	2.40E-05	8.80E-05	0	3.73E-04	4.10E-05	5.80E-05	7.70E-05	4.20E-05
0	0	0	0	0	0	1.30E-05	0	9.80E-05	4.90E-05	0	1.36E-04	0	0
0	2.60E-05	2.70E-05	0	0	0	0	0	0	0	0	0	0	0
0	0	0	0	0	0	0	0	0	0	0	0	0	2.70E-05
0	0	7.40E-05	3.40E-05	0	0	0	9.50E-05	0	0	0	0	0	0
2.19E-04	0	0	0	0	0	1.69E-04	0	0	0	0	0	0	0
0	0	0	0	0	0	0	0	0	0	0	0	0	0

H096	H084	H040	H094	H060	H078	H035	H015	H090	H109	H057	H099	H118	H093
0.208809	0.206788	0.198863	0.19777	0.197657	0.197423	0.196162	0.1944	0.188941	0.188437	0.183752	0.181273	0.180473	0.17709
0.192498	0.166955	0.392879	0.401043	0.501353	0.410446	0.457789	0.313294	0.322065	0.314113	0.27318	0.443314	0.431688	0.352914
0.417844	0.518881	0.199205	0.194462	0.14397	0.13522	0.168196	0.253871	0.296907	0.351106	0.320279	0.217889	0.216002	0.329803
0.091991	0.073391	0.111862	0.108784	0.068603	0.137124	0.122566	0.176248	0.111174	0.088203	0.099195	0.070106	0.106551	0.096215
0.037433	0.017269	0.05001	0.045329	0.054627	0.022499	0.016634	0.021378	0.029935	0.030065	0.055933	0.0272	0.018959	0.018323
0.040217	0.006287	0.038345	0.040586	0.007224	0.084426	0.02958	0.028717	0.032092	0.024851	0.033963	0.027885	0.039315	0.020532
0.005662	0.004041	0.00742	0.011248	0.017088	0.010179	0.005117	0.011901	0.018641	0.002587	0.033291	0.030809	0.005421	0.004662
0.004888	0.00435	9.83E-04	6.29E-04	0.006673	0.002402	0.003379	1.70E-04	1.95E-04	5.12E-04	3.79E-04	9.33E-04	0.001338	3.98E-04
4.34E-04	0.001124	4.00E-04	1.49E-04	7.28E-04	2.81E-04	5.42E-04	0	5.00E-05	1.09E-04	0	1.02E-04	1.14E-04	2.80E-05
5.20E-05	9.14E-04	3.30E-05	0	0.001015	0	3.50E-05	0	0	0	0	0	5.10E-05	3.50E-05
0	0	0	0	0	0	0	0	0	0	0	0	0	0
3.10E-05	0	0	0	1.02E-04	0	0	2.10E-05	0	1.70E-05	0	4.53E-04	0	0
0	0	0	0	4.43E-04	0	0	0	0	0	2.80E-05	3.60E-05	8.80E-05	0
0	0	0	0	0	0	0	0	0	0	0	0	0	0
0	0	0	0	0	0	0	0	0	0	0	0	0	0
1.41E-04	0	0	0	6.80E-05	0	0	0	0	0	0	0	0	0
0	0	0	0	0	0	0	0	0	0	0	0	0	0
0	0	0	0	4.49E-04	0	0	0	0	0	0	0	0	0

H009	H114	H076	H023	H083	H120	H027	H020	H086	H049	H064	H013	H075	H089
0.174586	0.171798	0.169669	0.168998	0.16844	0.168264	0.167774	0.166146	0.165208	0.163562	0.163017	0.155407	0.147805	0.141842
0.391714	0.383642	0.301192	0.367606	0.293826	0.287221	0.296067	0.276764	0.332222	0.279202	0.233299	0.327815	0.19309	0.300638
0.14136	0.236358	0.107357	0.312789	0.384709	0.430861	0.417885	0.31516	0.325041	0.33627	0.431133	0.3608	0.565048	0.175974
0.219414	0.101952	0.340218	0.095792	0.103246	0.036796	0.028859	0.089928	0.101789	0.118524	0.14268	0.125903	0.042486	0.302672
0.018509	0.081043	0.022902	0.025523	0.02659	0.061595	0.034022	0.089943	0.025812	0.029977	0.019252	0.016173	0.021121	0.028817
0.034966	0.01297	0.032415	0.018549	0.016397	0.002363	0.041306	0.048707	0.030416	0.031591	0.001641	0.008449	0.021187	0.035463
0.018668	0.011928	0.025349	0.009084	0.004265	0.005041	0.006808	0.011171	0.013499	0.017643	0.007304	0.004673	0.003027	0.012773
4.72E-04	2.54E-04	8.51E-04	0.00147	0.001914	0.006332	1.16E-04	0.001904	0.004194	0.015177	0.001439	6.72E-04	0.005955	0.001442
0	4.20E-05	4.70E-05	1.89E-04	4.97E-04	0.001403	0	7.40E-05	0.001604	0.006689	1.49E-04	1.08E-04	2.10E-04	2.99E-04
7.50E-05	1.30E-05	0	0	9.00E-05	1.24E-04	0.005932	1.15E-04	1.85E-04	7.65E-04	0	0	3.40E-05	8.00E-05
0	0	0	0	0	0	5.28E-04	0	0	0	0	0	0	0
5.30E-05	0	0	0	0	0	5.85E-04	5.40E-05	0	5.59E-04	6.30E-05	0	3.70E-05	0
1.83E-04	0	0	0	0	0	1.18E-04	1.40E-05	0	0	0	0	0	0
0	0	0	0	2.60E-05	0	0	0	0	0	0	0	0	0
0	0	0	0	0	0	0	0	0	0	0	0	0	0
0	0	0	0	0	0	0	0	3.00E-05	4.10E-05	2.30E-05	0	0	0
0	0	0	0	0	0	0	2.00E-05	0	0	0	0	0	0
0	0	0	0	0	0	0	0	0	0	0	0	0	0

H067	H063	H137	H147	H097	H102	H123	H048	H017	H146	H074	H004	ID...96	CC64
0.141097	0.13146	0.129329	0.12534	0.124133	0.118435	0.11023	0.107126	0.097391	0.093929	0.072816	0.048721	Firmicutes	0.989153
0.262916	0.148703	0.26049	0.236947	0.264504	0.299742	0.241301	0.213957	0.159908	0.458718	0.323064	0.220959	Bacteroidot	0.003404
0.383759	0.571576	0.423048	0.429501	0.434846	0.364875	0.476419	0.52105	0.637591	0.130601	0.381905	0.582502	Proteobacte	0.004168
0.154644	0.055144	0.147918	0.149702	0.099854	0.159775	0.083794	0.045239	0.064596	0.196918	0.177516	0.078261	Fusobacteri	0.001397
0.036924	0.06703	0.016171	0.029619	0.033284	0.014199	0.045888	0.099558	0.02356	0.044845	0.010209	0.03181	Actinobacte	0.001412
0.013325	0.017935	0.014887	0.019169	0.035365	0.020476	0.030227	0.006823	0.01072	0.066554	0.023081	0.033097	Patescibacte	3.64E-04
0.00578	0.005912	0.006355	0.007729	0.007362	0.020161	0.009784	0.002619	0.001916	0.008363	0.009374	0.00386	Campilobac	5.50E-05
0.001037	0.001775	0.001521	0.001787	5.18E-04	0.002305	0.001955	0.00221	0.003832	0	0.001828	6.80E-04	Spirochaeto	2.50E-05
3.75E-04	2.71E-04	2.61E-04	2.06E-04	1.34E-04	3.20E-05	3.01E-04	0.00113	3.69E-04	0	2.07E-04	8.80E-05	Synergistota	0
1.43E-04	0	2.00E-05	0	0	0	0	2.88E-04	1.17E-04	2.70E-05	0	0	Desulfobact	0
0	0	0	0	0	0	0	0	0	0	0	0	Deferribacte	0
0	1.94E-04	0	0	0	0	3.10E-05	0	0	0	0	0	Cyanobacte	0
0	0	0	0	0	0	4.80E-05	0	0	4.50E-05	0	0	Verrucomic	0
0	0	0	0	0	0	0	0	0	0	0	0	Bacteria_un	2.20E-05
0	0	0	0	0	0	0	0	0	0	0	0	Deinococco	0
0	0	0	0	0	0	0	0	0	0	0	0	Chloroflexi	0
0	0	0	0	0	0	2.20E-05	0	0	0	0	2.20E-05	Elusimicrob	0
0	0	0	0	0	0	0	0	0	0	0	0	Planctomyc	0

CC26	CC39	CC67	CC12	CC45	CC27	CC46	CC43	CC16	CC42	CC06	CC28	CC68	CC37
0.849417	0.837206	0.734938	0.71711	0.655238	0.617094	0.575369	0.552325	0.531021	0.530656	0.506457	0.501299	0.492982	0.486067
0.010422	0.035848	0.100967	4.43E-04	0.170589	0.091317	0.095327	0.019044	0.035167	0.407479	0.337064	0.03914	0.022871	0.086928
0.025105	0.019844	0.05256	0.114165	0.076939	0.075619	0.213701	0.41923	0.358041	0.03076	5.85E-04	0.131327	0.402348	0.271043
0.057889	0.061324	0.040533	0.139323	0.046939	0.133872	0.01573	9.95E-04	0.040699	0.007365	0.077846	0.061403	0.067434	0.029776
0.054705	0.032429	0.066301	0.026765	0.036616	0.034666	0.017016	0.006733	0.017834	0.022315	0.0275	0.261203	0.009483	0.11852
4.91E-04	0.003576	0.003741	1.14E-04	0.010541	0.04015	0.052595	4.90E-05	4.01E-04	6.10E-05	0.049817	0.00475	0.003478	0.005882
0.001044	0.005475	5.67E-04	0	0.002362	0.004617	0.014852	8.47E-04	0.009752	0.001364	5.08E-04	3.35E-04	0.001147	0.001288
7.64E-04	0.001736	4.20E-05	0	5.98E-04	0.002489	0.014076	1.30E-05	0.006024	0	3.70E-05	3.50E-04	1.27E-04	2.99E-04
1.17E-04	0.00225	0	5.10E-05	8.20E-05	9.20E-05	5.33E-04	5.00E-05	2.00E-05	0	0	0	0	0
4.60E-05	3.12E-04	0	0	0	0	0	2.00E-05	3.50E-05	0	0	6.70E-05	0	1.36E-04
0	0	0	0	0	0	0	0	0	0	0	0	0	0
0	0	0	0.001573	0	8.40E-05	8.01E-04	6.14E-04	9.04E-04	0	1.86E-04	6.60E-05	1.30E-04	0
0	0	0	0	0	0	0	5.80E-05	1.60E-05	0	0	0	0	0
0	0	3.51E-04	0	9.60E-05	0	0	0	1.10E-05	0	0	6.00E-05	0	6.10E-05
0	0	0	4.56E-04	0	0	0	0	0	0	0	0	0	0
0	0	0	0	0	0	0	1.20E-05	0	0	0	0	0	0
0	0	0	0	0	0	0	1.00E-05	7.50E-05	0	0	0	0	0
0	0	0	0	0	0	0	0	0	0	0	0	0	0

CC57	CC58	CC55	CC02	CC30	CC21	CC36	CC52	CC17	CC34	CC54	CC29	CC44	CC48
0.450932	0.435993	0.417968	0.401591	0.395558	0.393057	0.380547	0.375541	0.369646	0.353933	0.350364	0.346822	0.325217	0.319542
0.073418	0.055699	0.115178	0.287728	0.07034	0.114758	0.143261	0.060878	0.282462	0.348331	0.183473	0.246964	0.232711	0.45682
0.200502	0.225228	0.026164	0.006261	0.152757	0.36389	0.295499	0.207897	0.026586	0.077828	0.002071	0.027064	0.127644	0.107252
0.096453	0.220188	7.13E-04	0.085894	0.287069	0.101469	0.08647	0.109163	0.238036	0.08296	0.177151	0.105002	0.212353	4.39E-04
0.161372	0.050583	0.428484	0.217269	0.08412	0.023053	0.080382	0.201943	0.057429	0.053173	0.267524	0.27083	0.090661	0.03407
0.010783	0.010719	8.20E-05	0	0.006563	0.002863	0.008231	0.011264	0.007724	0.082179	0.009741	0.001696	0.009984	1.08E-04
0.004745	7.15E-04	0.011411	0.001183	0.003412	7.34E-04	0.003205	0.00724	0.014212	6.50E-04	0.009626	0.001411	0.001163	0.080258
0.001238	0	0	0	1.36E-04	7.70E-05	0.001745	0.013134	0.002631	8.68E-04	5.00E-05	1.88E-04	5.00E-05	6.60E-05
2.75E-04	0	0	2.40E-05	4.50E-05	0	6.12E-04	0.003457	4.40E-05	5.50E-05	0	0	0	7.60E-05
0	0	0	0	0	0	1.60E-05	6.20E-05	3.53E-04	0	0	0	0	0
0	0	0	0	0	0	0	0	0	0	0	0	0	0
3.10E-05	0	0	3.10E-05	0	9.90E-05	1.60E-05	0.009421	7.67E-04	2.30E-05	0	0	6.20E-05	0.001351
2.26E-04	0	0	0	0	0	0	0	1.10E-04	0	0	0	0	0
2.50E-05	8.75E-04	0	1.90E-05	0	0	0	0	0	0	0	2.30E-05	1.55E-04	0
0	0	0	0	0	0	0	0	0	0	0	0	0	0
0	0	0	0	0	0	1.60E-05	0	0	0	0	0	0	1.80E-05
0	0	0	0	0	0	0	0	0	0	0	0	0	0
0	0	0	0	0	0	0	0	0	0	0	0	0	0

CC09	CC24	CC05	CC08	CC01	CC25	CC70	CC33	CC71	CC20	CC72	CC63	CC62	CC69
0.293209	0.273739	0.247725	0.236335	0.206339	0.205027	0.186465	0.175181	0.175007	0.14546	0.137555	0.123821	0.11002	0.109399
0.159135	0.42077	0.63192	0.202396	0.238401	0.105856	0.13724	0.29296	0.071937	0.079296	0.289614	0.127253	0.031201	0.140834
0.01254	0.030049	0.001043	0.001846	0.01146	0.384403	0.507041	0.007482	0.228221	0.028947	0.230958	0.548178	0.59288	0.594171
0.498856	0.165166	0.026879	0.315624	0.526079	0.150975	0.06685	0.491764	0.467915	0.069281	0.266162	0.169674	0.165351	0.108366
0.015758	0.07929	0.003358	0.24316	0.015923	0.151287	0.052145	0.028148	0.037034	0.654535	0.03755	0.025216	0.09961	0.044619
0.018086	0.00962	0.085332	4.95E-04	1.60E-04	0.001148	0	0.00121	0.008651	0.021448	0.021498	0.005506	5.63E-04	0.002184
0.001898	0.020699	0.003743	1.44E-04	0.001638	0.001065	0.048873	0.003121	0.011081	5.96E-04	0.014767	1.29E-04	3.42E-04	2.14E-04
5.18E-04	6.67E-04	0	0	0	0	4.50E-05	6.40E-05	1.54E-04	3.82E-04	0.001896	7.20E-05	3.30E-05	0
0	0	0	0	0	0	0	7.00E-05	0	0	0	6.20E-05	0	0
0	0	0	0	0	0	0	0	0	0	0	3.20E-05	0	0
0	0	0	0	0	0	0	0	0	0	0	0	0	0
0	0	0	0	0	0	0.001316	0	0	5.50E-05	0	0	0	0
0	0	0	0	0	0	0	0	0	0	0	0	0	0
0	0	0	0	0	2.39E-04	2.50E-05	0	0	0	0	5.70E-05	0	2.13E-04
0	0	0	0	0	0	0	0	0	0	0	0	0	0
0	0	0	0	0	0	0	0	0	0	0	0	0	0
0	0	0	0	0	0	0	0	0	0	0	0	0	0
0	0	0	0	0	0	0	0	0	0	0	0	0	0

CC60	CC65	CC31	CC73	CC35
0.108681	0.095131	0.063811	0.063084	0.019632
0.100983	0.034034	0.134971	0.043338	0.877624
0.026721	0.785831	0.003531	0.781265	0.002412
0.75545	0.06638	0.77802	0.095063	0.001526
0.005247	0.013591	0.01732	0.010149	0.0978
0.001759	0.004518	0.001081	0.001844	4.40E-04
7.23E-04	2.36E-04	0.001016	0.005207	5.66E-04
3.30E-04	0	2.50E-04	5.00E-05	0
1.06E-04	0	0	0	0
0	0	0	0	0
0	0	0	0	0
0	0	0	0	0
0	0	0	0	0
0	2.79E-04	0	0	0
0	0	0	0	0
0	0	0	0	0
0	0	0	0	0
0	0	0	0	0

Table S3 .The abundance and composition at the genus level of each sample

ID	H017	H024	H063	H149	H147	H097	H027	H048	H137	H004	H064
Neisseria	0.592341	0.487396	0.427697	0.407569	0.378587	0.375387	0.366007	0.364857	0.351371	0.348748	0.345873
Prevotella	0.059092	0.100076	0.012221	0.02978	0.071873	0.111644	0.084919	0.11817	0.083403	0.054648	0.132146
Streptococcus	0.025139	0.01569	0.074578	0.158869	0.032215	0.014999	0.033665	0.045466	0.036809	0.010835	0.050375
Veillonella	0.029849	0.073451	0.005236	0.014336	0.037441	0.046353	0.015317	0.017868	0.034378	0.009426	0.051611
Leptotrichia	0.013513	0.020762	0.019473	0.017147	0.019427	0.039369	0.009573	0.002477	0.036458	0.011289	0.029468
Fusobacterium	0.051083	0.001311	0.035671	0.05407	0.130275	0.060485	0.019286	0.042734	0.11146	0.065947	0.113212
Alloprevotella	0.023144	0.049278	0.015376	0.008225	0.045055	0.060652	0.031339	0.0143	0.045153	0.051446	0.031686
Porphyromonas	0.062731	0.005937	0.078401	0.050498	0.078587	0.039031	0.06451	0.06468	0.077807	0.07496	0.018717
Haemophilus	0.037641	0.006796	0.070354	0.093558	0.039897	0.042977	0.035072	0.141334	0.048534	0.060807	0.07313
Actinomyces	0.009407	0.006377	0.019444	0.012973	0.014498	0.018887	0.012263	0.014297	0.00997	0.003662	0.010843
Rothia	0.004014	0.001558	0.036355	0.015289	0.013771	0.012381	0.017571	0.082465	0.003597	0.027796	0.007186
Capnocytophaga	0.010417	0.006893	0.033672	0.013489	0.039021	0.047785	0.023143	0.011365	0.047469	0.035214	0.046566
Gemella	0.002706	6.48E-04	0.005705	0.006465	0.002295	5.90E-04	0.001921	0.00864	0.004112	5.96E-04	0.001762
TM7x	0.002855	0.005644	0.003148	0.001067	0.010906	0.019467	0.002562	0.001794	0.002765	0.002307	0.001035
Lachnoanaerobaculum	0.004089	0.004048	0.001862	0.002667	0.010999	0.00925	0.002987	0.004799	0.01069	0.005544	0.015475
Campylobacter	0.001916	0.002362	0.005912	0.004948	0.007729	0.007362	0.005665	0.002619	0.006355	0.00386	0.007304
Granulicatella	0.007609	0.004525	0.02586	0.019562	0.008173	0.002173	0.005463	0.007037	0.003749	0.009324	0.007687
Actinobacillus	1.03E-04	0.001087	0.002558	0.019316	0.003222	0.001533	3.58E-04	6.00E-05	0.00594	2.18E-04	0.007635
g__Absconditabacteriales_	0.002632	6.78E-04	0.006455	0.001623	0.004047	0.012491	0.034298	0.001353	0.008419	0.00707	1.20E-04
Lautropia	0.001939	0.001915	0.031781	0.002159	8.47E-04	0.002267	0.006872	0.001502	0.005633	0.002374	2.60E-05
Bacteroides	1.69E-04	0.001639	2.94E-04	3.29E-04	0	1.12E-04	0.009605	7.60E-05	6.80E-05	1.86E-04	2.67E-04
Oribacterium	0.003347	0.002013	9.64E-04	0.001569	0.003645	0.008616	0.00125	0.002199	0.005343	0.00192	0.004862
Pseudomonas	1.41E-04	3.40E-05	0.002866	0.001667	1.69E-04	2.32E-04	0.002062	0.001041	5.48E-04	7.38E-04	6.84E-04
Megasphaera	4.16E-04	4.70E-05	0	0	5.10E-05	3.17E-04	8.59E-04	8.33E-04	0.001262	0	6.20E-04
Stomatobaculum	0.001561	0	4.60E-05	5.12E-04	0.001261	8.04E-04	6.40E-04	5.12E-04	0.003894	1.01E-04	0.007112
Selenomonas	0.004515	9.87E-04	3.05E-04	3.20E-04	5.15E-04	0.01268	6.34E-04	4.73E-04	0.002413	1.70E-05	0.001429
Peptostreptococcus	0.001937	0	7.19E-04	8.05E-04	0.004227	0.002023	7.49E-04	0.002503	0.002235	0.001464	0.002865
Moraxella	0	0.003258	0	4.54E-04	5.83E-04	0	0	0	0	0.143582	0
Solobacterium	0.002598	0.003916	2.77E-04	8.48E-04	0.002304	0.003644	0.003829	0.002916	0.003066	0.001971	0.001367
Clostridia_UCG-014	7.41E-04	0.002803	0.001722	0.002187	0.004831	0.005542	0.004248	5.94E-04	0.002481	8.63E-04	5.54E-04
Aggregatibacter	0.003089	0.002637	0.008065	0.007188	0.00291	0.010143	3.55E-04	0.008115	0.008318	2.63E-04	3.17E-04
Faecalibacterium	3.20E-05	2.50E-05	2.21E-04	3.20E-05	5.10E-05	0	0.004606	0	0	1.69E-04	4.60E-05
Lachnospiraceae_uncultured	2.70E-04	0.001154	0	2.08E-04	1.32E-04	0.008508	8.44E-04	6.10E-05	0.001474	2.06E-04	0
Corynebacterium	0.007398	0.001056	0.010211	9.85E-04	5.30E-04	2.24E-04	0.001142	0.001556	4.70E-04	2.74E-04	1.57E-04
Halomonas	3.40E-05	0	1.34E-04	1.44E-04	0	0	1.63E-04	2.13E-04	0	4.10E-05	8.70E-05
Muribaculaceae	4.00E-05	0.041873	4.10E-05	0	0	0	0.060398	9.00E-05	0	2.20E-05	0
Tannerella	0.002436	0	0.001326	0.002711	0.00162	0.001407	4.56E-04	0.001187	0.003987	1.61E-04	0.001377
[Eubacterium]_nodatum_group	0.001749	0.001819	5.45E-04	0.002012	0.002475	0.001942	3.06E-04	0.002817	0.003293	7.05E-04	0.003086
Atopobium	5.47E-04	0.002521	9.40E-05	9.80E-05	5.57E-04	0.001792	6.17E-04	9.62E-04	0.001994	1.90E-05	6.91E-04
Treponema	0.003832	4.50E-05	0.001775	0.005021	0.001787	5.18E-04	1.16E-04	0.00221	0.001521	6.80E-04	0.001439
Lachnospiraceae_unclassified	1.42E-04	0.030147	1.05E-04	3.30E-05	4.30E-05	0	0.029084	3.00E-05	3.00E-05	4.40E-05	7.00E-05
Neisseriaceae_unclassified	4.43E-04	3.81E-04	0.023133	0.003488	0.001615	4.52E-04	8.28E-04	0.001982	0.001294	0.02331	0.001518
Saccharimonadales	0.002661	0.003747	0.003637	9.74E-04	6.61E-04	8.50E-04	0.001011	6.31E-04	0.002177	2.08E-04	8.50E-05
Abiotrophia	3.30E-04	4.90E-05	0.004179	1.33E-04	1.35E-04	0	1.94E-04	9.90E-05	6.35E-04	2.50E-05	0
Rhodococcus	0	0	1.66E-04	0	0	0	0.001484	0	0	0	1.90E-05
Parvimonas	0.002292	0	0.00121	0.001394	0.00224	3.92E-04	0	0.001463	0.002465	8.84E-04	0.003639
Johnsonella	6.30E-05	6.30E-05	0.001774	7.11E-04	0.003696	0.001341	0	3.00E-05	0.005188	7.26E-04	0.004791
Fusicatenibacter	3.00E-05	3.70E-05	2.30E-05	0	4.70E-05	0	0.002083	0	0	5.10E-05	0
Lachnospiraceae_NK4A136_group	0	0.027696	6.30E-05	1.02E-04	0	0	0.018859	0	0	3.20E-05	0
Lactobacillales_unclassified	3.57E-04	7.70E-05	2.95E-04	0.001749	1.36E-04	7.00E-05	3.60E-05	0.001299	1.48E-04	4.40E-05	0
F0332	0.002169	3.68E-04	2.95E-04	1.31E-04	1.36E-04	0	0	1.63E-04	5.30E-05	1.90E-05	3.33E-04
Bergeyella	5.21E-04	3.90E-05	0.006663	0.003374	4.73E-04	4.67E-04	0.001385	0.001439	6.12E-04	0.003322	0.001011
Filifactor	0.001709	1.39E-04	0.001384	0.003574	0.00244	3.22E-04	0	0.002138	9.10E-04	4.44E-04	0.001105
Dialister	9.62E-04	0	0	7.14E-04	6.30E-04	4.10E-05	8.60E-05	8.26E-04	1.47E-04	5.00E-05	1.61E-04
P5D1-392	1.09E-04	1.36E-04	0	3.58E-04	3.50E-04	1.31E-04	0	5.50E-05	1.76E-04	0	1.46E-04

Saccharimonadaceae	5.57E-04	1.93E-04	5.99E-04	2.89E-04	7.80E-05	0	2.29E-04	4.64E-04	6.30E-05	0	0
Acinetobacter	0	0	2.43E-04	1.05E-04	0	0	0.00202	6.10E-05	3.10E-05	1.01E-04	1.04E-04
Kingella	1.86E-04	0	2.90E-05	0	0	0	7.90E-05	5.10E-05	0	0	3.30E-05
Sphingomonas	0	0	6.20E-05	0	0	0	6.27E-04	0	0	0	2.60E-05
Bifidobacterium	0	4.40E-05	6.20E-05	0	0	0	6.76E-04	3.60E-05	0	0	2.30E-05
Leptotrichiaceae_uncultured	0	0	0	0	0	0	0	2.80E-05	0	0.001025	0
Megamonas	0	0	4.50E-05	0	0	3.00E-05	6.29E-04	5.40E-05	0	2.00E-05	2.50E-05
Burkholderiales_unclassified	5.57E-04	5.06E-04	0	8.40E-04	3.41E-04	3.78E-04	0	5.73E-04	3.28E-04	0	4.54E-04
Selenomonadaceae_uncultured	6.50E-05	0	0	2.70E-05	0	0	0	2.60E-05	0	0	0
[Eubacterium]_ruminantium_group	0	0	4.50E-05	0	0	0	7.33E-04	0	0	2.50E-05	0
Helicobacter	0	0.002628	0	0	0	0	0.001143	0	0	0	0
Christensenellaceae_R-7_group	0	0	1.76E-04	0	0	0	5.35E-04	0	0	1.20E-04	2.50E-05
Lactobacillus	0	0.002741	2.40E-05	0	0	0	8.65E-04	0	0	0	0
Phascolarctobacterium	0	0	2.30E-05	0	0	0	3.89E-04	0	0	0	0
Agathobacter	0	4.90E-05	0	0	0	4.90E-05	4.65E-04	0	0	0	2.10E-05
Gracilibacteria	0.001062	0	0.003656	5.72E-04	0	8.30E-05	3.40E-04	1.53E-04	2.98E-04	0.022938	0
Prevotellaceae_NK3B31_group	0	0.005545	0	0	0	0	0.004119	0	0	0	0
Mucispirillum	0	3.33E-04	0	0	0	0	5.28E-04	0	0	0	0
Serratia	0	0	1.22E-04	9.90E-05	0	0	0	0	2.40E-05	2.00E-05	2.20E-05
Acidovorax	0	0	2.10E-05	0	0	0	4.27E-04	0	0	0	0
Propionibacterium	0	0	0	0	0	0	0	0	0	0	0
Others	0.008713	0.062823	0.010602	0.020633	0.010466	0.011807	0.065476	0.012224	0.008982	0.007139	0.007572

H075	H013	H074	H067	H086	H083	H084	H057	H109	H049	H020	H093	H070	H150
0.34477	0.335146	0.308855	0.304995	0.292092	0.288331	0.285485	0.27495	0.273822	0.272606	0.27238	0.270214	0.268633	0.268449
0.066309	0.211452	0.088822	0.176947	0.141431	0.155242	0.048372	0.218746	0.111531	0.069611	0.116189	0.173611	0.117945	0.210015
0.077125	0.033248	0.011615	0.027529	0.046936	0.056098	0.136063	0.044886	0.0746	0.034759	0.058699	0.070059	0.116401	0.081533
0.010792	0.058267	0.021017	0.053197	0.032731	0.043858	0.012699	0.070345	0.051644	0.04339	0.053242	0.066035	0.035009	0.08946
0.004879	0.026779	0.028198	0.042336	0.021794	0.021705	0.007292	0.034352	0.014956	0.019731	0.021175	0.023664	0.107391	0.016506
0.037398	0.09898	0.149318	0.112308	0.079995	0.081541	0.066099	0.064843	0.072631	0.098793	0.06863	0.072551	0.023398	0.042652
0.029289	0.076226	0.060005	0.057173	0.077572	0.035891	0.030374	0.017874	0.116978	0.021466	0.099695	0.043677	0.015942	0.063454
0.083228	0.033342	0.072737	0.021185	0.078018	0.084547	0.061623	0.010783	0.071982	0.099656	0.045598	0.106271	0.01832	0.037148
0.090715	0.021981	0.063188	0.066784	0.021575	0.079797	0.077275	0.035488	0.043221	0.056453	0.031683	0.053471	0.146818	0.024823
0.002265	0.01017	0.004304	0.028517	0.012644	0.012585	0.00672	0.029663	0.019118	0.013356	0.040976	0.009362	0.016457	0.030365
0.015027	0.003614	0.005322	0.006175	0.009934	0.011228	0.008468	0.022074	0.008271	0.014498	0.037663	0.007152	0.016868	0.02271
0.008212	8.82E-04	0.095981	0.00436	0.024963	0.012295	0.017579	0.017403	0.009668	0.076725	0.009068	0.026068	0.004473	0.00478
0.027507	0.001573	0.003131	0.002499	0.003538	0.006615	0.012608	0.002921	0.012744	0.008068	0.005209	0.002491	0.024116	0.002371
0.003571	0.002706	0.009461	0.010575	0.014712	0.007377	0.001031	0.008069	0.004742	0.014527	0.016805	0.005977	0.002557	0.007545
0.003007	0.00936	0.008835	0.013628	0.011726	0.010652	0.001774	0.010579	0.006091	0.008659	0.00951	0.009591	0.005892	0.00803
0.002965	0.004673	0.009374	0.005703	0.013464	0.004265	0.00398	0.033291	0.002587	0.017643	0.011171	0.004662	0.008809	0.006138
0.006062	0.004047	0.003795	0.003817	0.008827	0.007683	0.003707	0.004837	0.015748	0.008692	0.005437	0.004901	0.00406	0.020166
0.071346	0.001533	2.21E-04	0.004509	5.27E-04	8.90E-05	0.137301	0.002364	0.005022	2.00E-05	0.002495	0.001542	3.60E-05	0
0.009703	0.003057	0.008478	5.19E-04	0.010037	0.006262	0.004323	0.023067	0.015524	0.014069	0.002273	0.008087	2.37E-04	0.007682
8.34E-04	5.10E-05	0.002907	0.004138	0.001374	0.001373	0.003815	0.002053	5.77E-04	2.92E-04	0.001277	1.91E-04	0.006026	1.91E-04
5.93E-04	0	9.30E-05	3.81E-04	1.08E-04	0.00175	0.00132	4.63E-04	1.34E-04	7.34E-04	0.001231	3.80E-05	1.21E-04	0
7.58E-04	0.003176	0.00116	0.005189	0.0045	0.006613	0.001536	0.005331	0.001199	0.002739	0.002506	0.001885	0.003407	0.0049
0.042187	0	1.11E-04	7.47E-04	0.001011	0.001052	0.006649	7.53E-04	9.36E-04	0.003494	0.001557	1.99E-04	8.46E-04	2.96E-04
1.92E-04	0.010542	0	7.35E-04	0	1.66E-04	7.44E-04	0.003152	4.50E-05	3.90E-05	0.001594	0.001304	0.004117	0.003658
9.10E-05	0.006193	5.51E-04	0.003524	0.001788	0.008773	8.43E-04	0.002054	0.001486	0.003417	9.02E-04	0.002144	0.00198	0.006166
4.81E-04	0.001276	7.04E-04	0.002855	9.58E-04	3.26E-04	3.12E-04	0.006323	8.00E-05	0.001324	2.20E-04	0.001279	0.010001	0.00151
9.38E-04	0.003246	0.002345	0.005852	0.005757	0.00328	0.002065	0.005403	0.005101	0.004392	0.002354	0.001892	0.00102	0.00224
0	5.07E-04	0	3.30E-05	0	0	1.14E-04	0	0.023914	0	0	6.80E-05	3.50E-05	0
0.001007	0.003021	0.001477	0.002238	0.005287	0.003506	8.36E-04	0.005833	0.003482	0.00217	0.004391	0.002994	0.002325	0.006466
6.29E-04	0.003762	0.002422	1.95E-04	0.004602	0.002236	6.78E-04	0.007501	0.001074	4.58E-04	0.006216	0.004263	0.002198	0.001588
0.001726	6.07E-04	8.59E-04	6.88E-04	0.00255	0.010767	0.003745	5.66E-04	0.002282	1.59E-04	0.003466	8.43E-04	0.003216	3.62E-04
4.08E-04	0	0	4.30E-05	2.70E-05	3.39E-04	8.76E-04	2.38E-04	7.40E-05	4.54E-04	9.24E-04	9.80E-05	7.20E-05	0
1.79E-04	0.00138	0	6.38E-04	0.001872	1.21E-04	6.41E-04	0	1.58E-04	5.48E-04	8.65E-04	0.001268	0.003989	0.003343
0.002497	9.10E-05	0	7.15E-04	0.001262	0.001766	4.63E-04	0.001508	0.001365	6.30E-04	0.004008	2.28E-04	0.003456	7.00E-05
0.002942	0	0	1.06E-04	1.81E-04	6.40E-05	3.12E-04	7.70E-05	3.60E-05	1.68E-04	1.13E-04	0	3.00E-05	0
6.10E-05	3.80E-05	0	0	0	0	5.60E-05	3.00E-05	0	6.20E-05	6.60E-05	0	0	0
3.19E-04	0.004962	0.001464	8.92E-04	0.004019	0.00246	0.003785	0.004195	0.002102	0.007013	0.002409	0.00225	7.01E-04	6.90E-04
5.87E-04	0.006018	5.38E-04	0.004537	0.004353	0.001584	0.001146	0.002487	0.001757	0.00261	0.001961	0.001244	6.75E-04	0.00297
9.60E-05	0.002167	5.13E-04	0.001146	0.001579	5.69E-04	3.14E-04	0.001571	6.61E-04	8.26E-04	0.003141	0.001366	5.96E-04	0.002709
0.005955	6.72E-04	0.001828	0.001037	0.004194	0.001914	0.00435	3.79E-04	5.12E-04	0.01513	0.001904	3.98E-04	7.17E-04	2.20E-04
1.18E-04	0	3.20E-05	0	3.12E-04	2.76E-04	2.63E-04	1.18E-04	1.55E-04	2.93E-04	1.71E-04	0	4.20E-05	3.10E-05
0.00145	1.00E-04	0.0039	1.07E-04	0.002355	0.001126	7.96E-04	0.001225	2.18E-04	6.09E-04	2.25E-04	4.11E-04	6.33E-04	9.30E-05
0.001603	3.10E-04	0.00376	5.30E-04	0.002796	6.50E-05	3.58E-04	4.51E-04	0.003235	2.05E-04	0.004016	0.004984	1.55E-04	0.009431
1.07E-04	0	8.50E-05	0	6.90E-05	9.30E-05	0.002173	1.54E-04	9.14E-04	0	2.28E-04	1.08E-04	2.38E-04	0
2.30E-04	0	0	1.35E-04	3.00E-05	2.10E-05	5.43E-04	1.40E-04	6.20E-05	2.73E-04	2.19E-04	4.00E-05	0	0
7.00E-04	0.002446	0.003009	7.27E-04	0.008582	0.001956	0.001149	0.001176	0.003287	0.004924	0.002138	5.85E-04	3.70E-05	2.66E-04
3.10E-04	9.00E-05	0.004456	0.00237	0.003409	0.003308	0.001402	1.45E-04	0.001507	0.00577	0.001234	0.001012	3.60E-05	6.60E-05
2.30E-04	0	0	0	2.30E-05	9.10E-05	2.58E-04	1.76E-04	0	1.93E-04	3.88E-04	0	0	0
7.50E-05	0	0	0	0	2.10E-05	2.10E-05	4.90E-05	0	4.70E-05	4.60E-05	0	0	0
0.002949	9.41E-04	6.80E-05	0	9.20E-05	7.60E-05	0.017471	1.80E-04	6.30E-04	0	0.001633	3.46E-04	0.010617	0
2.80E-05	4.00E-05	0	7.60E-05	3.07E-04	2.61E-04	3.36E-04	2.05E-04	3.51E-04	1.12E-04	0.001254	5.70E-05	3.91E-04	0
0.002779	2.80E-04	0.003384	5.47E-04	0.002138	8.58E-04	0.001874	0.002846	7.52E-04	0.001695	0.001366	8.68E-04	7.57E-04	1.67E-04
0.001401	0.001739	0.002404	0.001639	0.004055	0.002392	0.002292	2.96E-04	0.002052	0.012665	4.14E-04	2.35E-04	0	5.80E-05
9.70E-04	3.28E-04	2.69E-04	5.48E-04	2.05E-04	3.95E-04	3.99E-04	8.82E-04	6.58E-04	4.33E-04	1.77E-04	3.92E-04	1.60E-04	1.33E-04
0	1.29E-04	2.60E-05	8.70E-05	2.26E-04	1.93E-04	0	0	3.99E-04	0	0	9.44E-04	1.84E-04	3.99E-04

3.01E-04	0	3.50E-05	3.48E-04	2.44E-04	2.25E-04	2.50E-05	1.36E-04	3.30E-04	6.70E-05	0.022573	2.41E-04	0.001203	2.80E-05
0.002173	5.30E-05	0	0	4.90E-05	7.40E-05	4.63E-04	1.88E-04	3.90E-05	3.35E-04	3.31E-04	3.80E-05	8.80E-05	6.00E-05
2.30E-05	0	0	3.60E-05	1.98E-04	2.40E-05	1.28E-04	2.00E-05	7.50E-05	4.80E-05	2.21E-04	0	1.04E-04	8.20E-05
1.85E-04	0	0	0	0	0	4.36E-04	8.10E-05	0	3.77E-04	2.10E-04	0	0	0
1.14E-04	0	3.70E-05	0	0	2.30E-05	8.80E-05	2.88E-04	7.20E-05	6.40E-05	1.02E-04	3.60E-05	4.10E-05	0
0	0	0	0	0	0	0	0	4.79E-04	0	0	0	0	0
8.40E-05	0	3.10E-05	0	0	4.20E-05	7.80E-05	6.40E-05	4.10E-05	6.00E-05	9.50E-05	0	0	0
0	4.05E-04	6.50E-05	1.21E-04	1.59E-04	2.97E-04	0	0	3.90E-04	0	0	0.001144	3.34E-04	2.68E-04
0	0	0	0	0	0	0	2.40E-05	1.27E-04	0	3.50E-05	1.12E-04	0	0
9.80E-05	0	0	0	0	5.10E-05	1.00E-04	0	4.10E-05	9.00E-05	1.60E-04	0	3.60E-05	0
0	0	0	0	0	0	0	0	0	0	0	0	0	0
8.20E-05	0	2.28E-04	0	8.50E-05	0	1.56E-04	3.00E-05	4.10E-05	6.90E-05	1.43E-04	0	0	0
5.90E-05	0	0	0	2.40E-05	2.20E-05	0	7.00E-05	0	0	3.00E-05	0	0	0
1.80E-05	0	0	0	0	4.30E-05	1.17E-04	8.50E-05	0	2.60E-05	8.30E-05	0	0	0
2.50E-05	0	0	0	0	0	1.66E-04	1.90E-05	0	2.10E-05	8.30E-05	0	0	0
0.005478	0	1.98E-04	0	1.16E-04	9.40E-05	2.21E-04	5.22E-04	2.52E-04	9.10E-05	5.50E-05	2.90E-05	0	0
0	0	0	0	0	0	0	0	0	0	0	0	0	0
0	0	0	0	0	0	0	0	0	0	0	0	0	0
0.001209	0	2.70E-05	4.40E-05	1.08E-04	0	2.82E-04	0	0	1.72E-04	1.68E-04	0	4.00E-05	0
2.50E-05	0	0	0	2.40E-05	0	5.40E-05	4.20E-05	0	6.50E-05	1.90E-05	0	0	0
0	3.90E-05	0	0	0	0	0	0	0	0	0	0	0	0
0.020526	0.008355	0.008357	0.01424	0.026456	0.013253	0.010978	0.013936	0.006038	0.031915	0.01318	0.00508	0.006014	0.007712

H061	H002	H123	H088	H096	H090	H108	H133	H015	H062	H044	H124	H028	H023
0.265415	0.261114	0.255664	0.249024	0.237599	0.230278	0.21561	0.213058	0.207391	0.187942	0.187927	0.187745	0.174083	0.172769
0.101255	0.115684	0.071452	0.127827	0.072502	0.232579	0.103635	0.093037	0.135931	0.108873	0.172259	0.123702	0.176783	0.195312
0.053651	0.045012	0.039869	0.109741	0.123276	0.028453	0.11939	0.202303	0.037382	0.077977	0.044917	0.131027	0.023529	0.050827
0.105599	0.118995	0.021197	0.0475	0.024623	0.089623	0.041661	0.015936	0.078098	0.066904	0.112993	0.056596	0.050287	0.060958
0.017331	0.020831	0.025046	0.014167	0.012013	0.102323	0.028272	0.009117	0.047446	0.020696	0.06058	0.021356	0.028602	0.019138
0.16925	0.047461	0.058748	0.062446	0.079978	0.008851	0.037511	0.05536	0.128802	0.063177	0.125915	0.039842	0.037372	0.075394
0.03968	0.09427	0.091819	0.063336	0.048139	0.07903	0.122655	0.028148	0.069935	0.029586	0.008626	0.13006	0.06899	0.084441
0.123473	0.058431	0.029053	0.091311	0.049094	0.001614	0.059422	0.101632	0.076669	0.053785	0.010874	0.038232	0.037027	0.069747
0.002448	0.038189	0.086097	0.040462	0.159802	0.03787	0.087436	0.028115	0.043906	0.050142	0.059352	0.070874	0.027668	0.039079
0.003367	0.062709	0.014312	0.029738	0.008131	0.021017	0.017107	0.016361	0.007938	0.030528	0.08984	0.01683	0.013012	0.009317
0.0112	0.004035	0.023985	0.011814	0.02633	0.006724	0.005354	0.039729	0.005846	0.027919	0.021849	0.010914	0.008206	0.013261
0.018084	0.015967	0.040742	0.004325	0.014377	0.008233	0.014978	0.016532	0.026972	0.007116	0.004733	0.014017	0.002853	0.013801
0.006747	7.96E-04	0.004233	0.007248	0.010541	1.97E-04	0.010598	0.019186	0.001794	0.007035	0.006612	0.010993	0.002342	0.006827
4.69E-04	0.011485	0.005849	0.008817	0.005233	0.029306	0.012741	0.006395	0.005047	0.01746	0.004248	0.006777	0.013281	0.005464
0.01264	0.005186	0.004426	0.004186	0.003287	0.008175	0.006496	0.004109	0.018547	0.005735	0.010084	0.002734	0.006381	0.006804
0.003488	0.016543	0.009726	0.005395	0.005557	0.018641	0.009944	0.002004	0.011901	0.005784	0.00976	0.012467	0.012076	0.009084
0.007611	0.002819	0.007229	0.006416	0.015847	0.002717	0.00528	0.013313	0.019231	0.026406	0.006059	0.014948	0.00393	0.006654
7.10E-05	0.001539	0.10307	0.022249	0.003356	0.013023	0.004922	0.002898	2.70E-04	0.008241	6.95E-04	0.003498	0.014256	0.007829
0.004564	0.010427	0.022499	1.56E-04	0.02178	0	0.011474	0.016337	0.010918	0.003012	3.24E-04	0.002279	0.007983	0.009461
0.006381	0.001424	0.002463	0.001844	0.001522	4.12E-04	0.001741	0.039704	3.67E-04	0.002442	3.98E-04	0.002279	3.83E-04	0.003033
1.67E-04	0.001606	0.002297	0.002235	9.99E-04	1.04E-04	5.74E-04	0.003271	2.80E-05	0.00892	2.18E-04	0.00148	0.001215	3.90E-05
0.007342	0.003314	0.003291	0.003966	0.002515	0.003831	0.004115	0.001493	0.006682	0.003464	0.005028	0.002812	0.004649	0.003424
2.71E-04	0.002749	0.007043	0.002102	0.007858	1.45E-04	0.003553	0.003303	9.10E-05	0.046738	0.001123	0.003141	4.50E-04	4.53E-04
3.42E-04	1.72E-04	0.001231	0.001299	0.001075	0.003075	2.20E-05	3.50E-05	7.44E-04	0.004059	0.011706	0.003194	0.005722	0.003057
0	0.001379	0.003663	0.002444	0.001993	4.60E-04	0.001138	1.98E-04	0.005458	5.61E-04	0.007237	0.002293	0.002511	9.73E-04
9.10E-05	5.96E-04	0.002233	0.001311	0.001458	0.020337	0.001741	4.90E-04	0.003338	0.001485	6.50E-04	0.002096	0.002536	6.44E-04
0.004246	0.001946	0.00272	0.003758	0.001049	0.0012	0.003382	0.005026	0.004398	0.007278	0.00299	0.002005	0.002173	0.002655
0	0	1.08E-04	1.65E-04	8.90E-05	0	0.014275	0.001625	0	0	0	0	0	0.079678
0.002608	0.002295	0.002837	0.003021	0.001411	0.001499	0.002148	0.00246	0.002102	0.002635	0.002048	0.002777	0.004906	0.003833
0.005315	0.004737	0.002276	0.001237	0.002022	0	0.005218	0.002182	0.003098	0.004847	0.001969	0.00478	0.004241	0.001902
0	0.003738	0.007047	0.004824	0.001388	0.012631	0.010703	0.005671	6.91E-04	0.004545	0.003053	0.022918	7.42E-04	0.008805
0	0.001037	8.33E-04	0	0.001019	0	3.89E-04	0.001797	1.35E-04	0.006405	1.44E-04	8.50E-04	3.30E-05	9.90E-05
4.20E-05	0.001494	0.002045	0.001235	3.64E-04	0.022497	0.006902	0	0.001838	1.61E-04	2.70E-05	9.88E-04	0.006324	0.001636
8.60E-05	6.69E-04	0.005264	8.95E-04	8.57E-04	8.66E-04	8.44E-04	0.009359	0.002757	0.002652	6.85E-04	8.44E-04	7.60E-04	6.66E-04
0	1.30E-04	2.41E-04	2.28E-04	4.79E-04	0	2.06E-04	5.50E-05	2.10E-05	0.001767	1.26E-04	2.26E-04	0	0
0	1.69E-04	1.51E-04	0	2.60E-05	0	2.80E-05	3.15E-04	4.40E-05	7.59E-04	6.30E-05	1.04E-04	0.035126	0
0.001926	0.001032	0.002828	0.002563	0.001803	5.90E-05	0.001505	0.001247	0.002685	0.001998	0.00254	6.79E-04	0.001922	5.99E-04
0.002697	0.001689	0.001454	0.001736	0.001472	0.001645	0.001346	0.004054	0.002017	0.001157	0.001208	0.001751	0.0026	0.002874
8.88E-04	0.002649	7.38E-04	0.002446	8.25E-04	0.001198	7.53E-04	9.60E-05	0.001467	0.002126	0.0036	0.001364	0.002242	0.001816
5.39E-04	0.003719	0.001955	0.005824	0.004888	1.95E-04	0.002156	5.95E-04	1.70E-04	0.004367	9.76E-04	0.006937	2.89E-04	0.00147
0	3.45E-04	1.58E-04	1.80E-05	1.40E-04	0	1.20E-04	3.09E-04	2.40E-05	0.001496	1.33E-04	3.41E-04	0.04485	6.80E-05
9.34E-04	7.72E-04	0.010223	2.20E-04	9.41E-04	5.31E-04	6.06E-04	5.70E-04	2.72E-04	0.001084	7.07E-04	0.002581	2.31E-04	6.38E-04
3.40E-05	1.36E-04	4.38E-04	3.57E-04	0.010083	9.30E-05	0.002633	0.003701	0.009146	0.006139	1.25E-04	0.00155	0.002588	0.001457
3.50E-05	0	7.60E-05	9.50E-05	6.70E-05	0	2.90E-05	8.10E-05	0	0	0	0.001284	4.40E-05	8.16E-04
0	3.82E-04	9.11E-04	4.60E-05	6.30E-04	0	5.41E-04	1.38E-04	0	0.005279	5.30E-05	0.00119	3.30E-05	3.60E-05
6.99E-04	0.001068	0.001672	0.004206	0.002118	0	0.001277	0.002006	5.23E-04	0.001753	9.08E-04	8.50E-04	7.80E-04	0.003289
0.002843	0.001165	0.001491	5.77E-04	0.00172	7.80E-05	8.61E-04	1.84E-04	0.003528	4.86E-04	1.81E-04	0.001092	1.56E-04	6.10E-04
0	3.83E-04	3.97E-04	3.20E-05	3.29E-04	0	7.10E-05	9.98E-04	0	0.002197	3.20E-05	3.01E-04	0	3.40E-05
0	1.80E-05	2.40E-05	0	1.31E-04	0	0	6.90E-05	0	2.58E-04	5.90E-05	7.10E-05	0.041699	0
2.48E-04	0	9.10E-05	6.35E-04	0	0	7.81E-04	9.61E-04	3.30E-05	0	0	0.004099	4.00E-05	0.00435
1.30E-04	0.00384	1.40E-04	4.36E-04	8.20E-05	3.90E-05	5.13E-04	0.001025	0.003348	1.60E-04	1.55E-04	1.10E-04	0	9.30E-05
4.10E-04	0.001018	0.001518	4.76E-04	0.002606	1.85E-04	0.001875	0.002952	6.89E-04	0.001644	9.63E-04	0.002826	0.001079	0.002295
0.005022	0.001458	4.97E-04	0.009026	7.65E-04	9.80E-05	1.74E-04	4.52E-04	1.63E-04	0.001358	8.10E-04	0.001457	0	0.001145
4.10E-05	0.001541	4.07E-04	0.003887	0.002916	0	4.62E-04	1.56E-04	3.16E-04	6.08E-04	0.003801	0.001206	0.001608	6.66E-04
0.001439	0	3.50E-05	5.53E-04	0	1.52E-04	0	0	2.50E-04	0	0	0	0	3.40E-05

0	4.58E-04	2.40E-05	8.77E-04	0.001328	1.21E-04	1.55E-04	0.00334	5.42E-04	0.001064	2.14E-04	6.89E-04	1.26E-04	6.70E-04
0	1.62E-04	9.47E-04	0	7.81E-04	0	2.47E-04	1.17E-04	2.00E-05	0.008787	1.04E-04	5.85E-04	0	7.50E-05
0	6.00E-05	0	4.80E-05	9.40E-05	5.20E-05	1.09E-04	3.43E-04	4.50E-05	4.35E-04	0.001195	5.70E-05	0	0
0	5.60E-05	6.26E-04	3.10E-05	3.26E-04	0	2.00E-04	6.00E-05	2.90E-05	0.003281	7.60E-05	1.51E-04	4.20E-05	0
0	1.85E-04	3.02E-04	0	1.61E-04	4.70E-05	5.40E-05	3.32E-04	0	0.00117	2.60E-05	1.22E-04	0	7.10E-05
0	0	0	0	0	0	0	0	0	0.005678	0	0	0	0
8.90E-05	2.03E-04	1.28E-04	0	1.41E-04	0	9.40E-05	2.57E-04	0	0.001094	0	2.83E-04	0	0
7.23E-04	0	0	5.24E-04	0	6.22E-04	0	0	1.23E-04	0	0	2.80E-05	0	8.00E-05
0	0	2.40E-05	7.80E-05	2.80E-05	9.70E-05	0	0	0	0	0	2.73E-04	9.27E-04	2.90E-05
0	1.70E-04	1.87E-04	0	1.40E-04	0	8.50E-05	2.21E-04	2.80E-05	0.001006	2.40E-05	7.30E-05	0	4.50E-05
0	0	0	0	0	0	0	0	0	0	0	0	0.038115	0
0	1.64E-04	1.13E-04	0	8.00E-05	0	5.40E-05	3.23E-04	0	7.80E-04	2.90E-05	2.00E-04	0	3.70E-05
0	0	2.00E-05	7.00E-04	7.60E-05	0	0	0	0	2.00E-05	0	0	0.025968	4.00E-05
4.10E-05	9.70E-05	5.00E-05	0	8.50E-05	0	6.40E-05	1.40E-04	0	4.02E-04	2.50E-05	1.12E-04	0	0
0	8.60E-05	5.00E-05	0	5.20E-05	5.50E-05	2.60E-05	1.67E-04	0	7.21E-04	6.80E-05	1.02E-04	0	0
0	5.09E-04	4.83E-04	1.90E-05	4.10E-05	1.77E-04	3.04E-04	1.36E-04	2.70E-05	7.80E-05	0	4.59E-04	0	2.83E-04
0	0	0	0	0	0	0	0	0	0	0	0	0	0
0	0	0	0	0	0	0	0	0	0	0	0	0.008999	0
0	2.48E-04	2.07E-04	5.60E-05	4.19E-04	5.00E-05	9.00E-05	2.80E-05	0	0.001133	0	1.67E-04	0	2.80E-05
0	8.40E-05	8.30E-05	0	9.70E-05	0	3.70E-05	0	0	8.61E-04	2.60E-05	1.70E-05	4.20E-05	0
0	0	0	0	0	0	0	0	0	0	0	0	0	0
0.008028	0.017355	0.010944	0.027812	0.017046	0.008795	0.011313	0.014388	0.008739	0.044344	0.00685	0.019315	0.045188	0.009288

H127	H094	H110	H025	H089	H010	H114	H118	H005	H107	H034	H113	H040	H146
0.168155	0.165555	0.162434	0.160705	0.159389	0.156542	0.156468	0.142658	0.141571	0.13871	0.13365	0.132205	0.123141	0.119874
0.175725	0.302806	0.077512	0.091781	0.072742	0.192279	0.295731	0.166853	0.099612	0.136339	0.213616	0.176193	0.234064	0.192844
0.04966	0.023055	0.116157	0.055333	0.01302	0.120512	0.030113	0.061838	0.028259	0.094172	0.034623	0.085959	0.056927	0.022118
0.063677	0.090179	0.055023	0.067648	0.050399	0.043497	0.091193	0.041131	0.068851	0.042541	0.066252	0.038876	0.054372	0.010043
0.050305	0.075733	0.02632	0.018814	0.100267	0.009459	0.017351	0.01147	0.072855	0.013105	0.031515	0.019497	0.072401	0.020232
0.089644	0.033051	0.10566	0.09607	0.202405	0.041878	0.084601	0.094606	0.159547	0.043454	0.017632	0.037751	0.039461	0.176686
0.082868	0.067744	0.051633	0.049065	0.056574	0.116427	0.048592	0.105647	0.061616	0.051552	0.029577	0.124359	0.12121	0.010177
0.045968	0.024664	0.07691	0.118325	0.048542	0.022937	0.021071	0.133493	0.030951	0.066931	0.00739	0.080876	0.016716	0.200453
0.042749	0.020573	0.070767	0.082003	0.013339	0.041269	0.055421	0.053514	0.004939	0.081992	0.033944	0.026317	0.037379	0.005404
0.022698	0.027273	0.028158	0.010596	0.018867	0.021	0.04481	0.007474	0.041573	0.022667	0.029411	0.009934	0.02946	0.010895
0.008089	0.01421	0.025702	0.00482	0.004825	0.027893	0.022058	0.007522	0.01973	0.044397	0.005108	0.011533	0.011073	0.033319
0.030434	0.003822	0.022547	0.017107	0.096315	0.003222	0.015125	0.0103	0.068741	0.004563	0.002415	0.05607	0.016558	0.048934
0.006451	0.001328	0.026469	0.011198	9.05E-04	0.00597	0.002445	0.009977	0.008465	0.021013	0.002021	0.021052	0.004866	4.39E-04
0.011645	0.026055	0.004235	0.017349	0.019594	0.00928	0.004916	0.005913	0.011612	0.002493	0.067025	0.00877	0.025672	0.018072
0.015116	0.016119	0.005045	0.008294	0.020155	0.002558	0.009869	0.006939	0.018384	0.005477	0.009712	0.003868	0.00621	0.019184
0.015717	0.011248	0.007856	0.007661	0.012773	0.007814	0.011928	0.005421	0.023168	0.008477	0.00598	0.006431	0.00742	0.008363
0.013637	0.012919	0.017853	0.006811	0.001687	0.009724	0.003581	0.011286	0.007767	0.031302	0.00588	0.011889	0.004964	0.002519
3.59E-04	1.71E-04	0.004824	0.003471	2.04E-04	0	0.014581	7.69E-04	0	0.003349	9.90E-04	8.62E-04	0.017129	2.00E-05
0.014053	0.010028	0.006742	0.008193	0.011568	0.001626	1.81E-04	0.02845	0.001751	0.001485	0.009076	0.019636	0.003651	0.029769
0.001447	2.03E-04	0.008728	0.003426	2.20E-04	0.001791	0.004077	0.00714	7.33E-04	0.007679	4.22E-04	0.00211	0.00232	0.001197
1.87E-04	1.40E-04	0.001445	4.64E-04	1.13E-04	0.020272	1.68E-04	0.006421	4.80E-05	0.013014	0.001521	0.002746	2.77E-04	0.002635
0.011527	0.004541	0.00355	0.006575	0.004912	0.002755	0.004552	0.00408	0.013681	0.003301	0.004757	0.005457	0.004813	0.00536
9.70E-04	4.97E-04	0.005867	7.13E-04	2.70E-04	0.034694	8.85E-04	0.002276	4.77E-04	0.037066	1.30E-04	0.005513	0.003798	2.42E-04
6.63E-04	0.010364	3.17E-04	7.10E-05	0.001163	0.001491	0.003931	4.99E-04	2.47E-04	9.54E-04	0.00546	0.003813	0.021275	2.20E-05
0.005178	0.005206	0.004029	0.002148	0.00182	1.53E-04	0.008115	0.001251	0.023484	0.002659	0.003873	9.08E-04	0.002078	1.41E-04
0.009502	0.00457	7.17E-04	0.001489	0.002635	9.62E-04	0.002188	7.62E-04	0.002062	0.002844	0.002391	9.68E-04	0.010483	0.003969
0.005519	0.002033	0.002637	0.004999	0.003596	0.004087	0.002745	0.00603	0.006423	0.001274	0.001568	0.003881	0.001661	0.006264
0	0	0	0.005951	3.80E-05	1.18E-04	2.30E-05	0	0	0	0	0	0	0.002295
0.005365	0.013059	0.001165	0.001753	0.003339	0.003381	0.002382	0.005534	0.023414	0.00307	0.004202	0.002632	0.005015	0.002386
0.004016	0.003752	0.002384	0.005381	0.004245	0.001033	0.001671	0.00388	0.012488	0.001165	0.012791	0.004588	0.004575	0.002005
0.009122	0.005805	0.010083	9.86E-04	0.001369	3.63E-04	0.002132	0.006449	6.90E-05	0.002764	0.001737	0.015481	0.009471	8.26E-04
9.20E-05	0	8.96E-04	8.30E-05	5.10E-05	0.012502	5.60E-05	0.003535	4.60E-05	0.010458	6.00E-05	0.002025	2.12E-04	0.001779
0.003209	0.002994	3.50E-04	1.85E-04	0.00175	0.001249	0.001537	1.06E-04	0.005935	8.40E-05	0.00917	0.001131	0.002688	0.001269
8.80E-05	4.67E-04	0.008781	2.71E-04	0.002732	7.32E-04	8.16E-04	0.001784	6.00E-05	0.0013	3.66E-04	0.00604	0.00267	3.65E-04
2.80E-05	3.70E-05	1.72E-04	6.40E-05	0	0.00134	4.70E-05	0	6.90E-05	0.001283	0	9.80E-05	3.49E-04	0
4.20E-05	0	3.01E-04	0.024734	0	0.001242	0	4.09E-04	3.50E-05	8.22E-04	0.038432	1.06E-04	8.20E-05	2.02E-04
0.003064	6.14E-04	0.004713	0.001302	0.014804	4.01E-04	0.001313	0.003047	0.017594	0.003825	7.84E-04	9.39E-04	8.69E-04	0.001937
0.002329	0.002798	7.63E-04	0.001335	0.00178	0.002937	0.001526	0.003209	0.002042	5.79E-04	0.002031	0.001294	0.00258	0.00794
0.001374	0.00323	7.77E-04	6.51E-04	0.001221	0.001379	0.011094	6.24E-04	0.003702	0.001008	0.003077	0.001288	0.004904	0
9.63E-04	6.29E-04	0.001061	0.006174	0.001442	1.99E-04	2.54E-04	0.001338	9.02E-04	5.46E-04	0.001065	0.00123	9.83E-04	0
3.90E-05	2.70E-05	3.73E-04	0.002053	0	0.002225	2.10E-05	6.20E-04	1.50E-05	0.002138	0.046067	5.47E-04	9.80E-05	2.14E-04
0.001079	6.30E-04	5.93E-04	4.52E-04	3.24E-04	9.20E-05	5.59E-04	4.02E-04	8.60E-05	1.06E-04	3.17E-04	0.003856	0.002689	4.24E-04
0.003521	1.69E-04	0.001492	0.02996	6.42E-04	0.003619	2.21E-04	0.00318	2.60E-05	0.002049	0.004559	0.008708	0.002142	0.012383
0	0	0.001174	2.56E-04	1.89E-04	0	5.04E-04	2.94E-04	0	0	8.20E-05	0.005616	1.21E-04	0
0	0	9.15E-04	4.50E-05	0	0.001619	9.90E-05	8.97E-04	1.80E-05	0.01119	0	0.001839	1.74E-04	0
0.00516	2.16E-04	0.003837	0.006578	0.002483	0.001437	9.10E-05	0.001356	0.001037	0.001256	2.33E-04	0.001927	0.001696	4.90E-05
0.002609	1.35E-04	0.002243	0.002155	0.009844	5.90E-05	9.70E-05	3.76E-04	0.005054	0	6.70E-05	7.54E-04	4.11E-04	7.70E-05
8.70E-05	0	3.92E-04	4.80E-05	0	0.006021	7.30E-05	0.001533	2.00E-05	0.004239	3.70E-05	9.89E-04	1.25E-04	8.07E-04
4.20E-05	0	1.76E-04	0.002153	0	8.51E-04	0	2.12E-04	1.90E-05	7.09E-04	0.024551	5.30E-05	7.20E-05	2.09E-04
3.04E-04	1.65E-04	0.004513	5.32E-04	1.28E-04	0	0.001281	4.05E-04	2.60E-05	7.36E-04	2.98E-04	0.005404	1.83E-04	0
1.30E-04	6.70E-05	0.003151	2.17E-04	4.04E-04	0.00102	0.002034	5.00E-05	7.40E-05	4.74E-04	0	3.81E-04	2.62E-04	0
0.001053	5.54E-04	0.004083	0.004402	0.001478	3.57E-04	0.001086	0.001341	0.001737	0.003428	7.50E-04	0.002434	0.002163	0.001102
3.29E-04	9.62E-04	8.94E-04	0.001829	0.003239	1.37E-04	7.50E-05	9.40E-04	2.33E-04	0	3.88E-04	8.40E-05	0.001488	0
3.12E-04	3.33E-04	9.83E-04	0.001768	0.003405	6.43E-04	2.61E-04	2.59E-04	6.43E-04	0.006114	1.00E-04	3.74E-04	0.004157	0
4.00E-05	2.20E-04	0	1.44E-04	2.98E-04	0	7.10E-05	2.50E-05	4.40E-05	0	0	0	0	0

9.70E-05	1.50E-04	5.39E-04	9.33E-04	0.00166	0	0.005968	1.43E-04	9.00E-05	3.50E-04	2.87E-04	8.08E-04	8.46E-04	2.78E-04
2.26E-04	0	5.95E-04	4.50E-05	0	0.0037	5.80E-05	4.68E-04	2.90E-05	0.005252	2.20E-05	0.002831	2.07E-04	0
5.00E-05	0	1.01E-04	6.60E-05	0	7.55E-04	0.00141	0	7.40E-05	0	0	1.17E-04	1.40E-04	0
0	0	6.42E-04	0	0	0.001026	2.20E-05	4.84E-04	1.40E-05	0.004192	0	7.84E-04	0	0
0	0	2.20E-04	1.30E-05	0	0.001479	0	5.57E-04	2.20E-05	0.001187	0	9.39E-04	1.51E-04	2.66E-04
1.71E-04	0	0	0	0	0	0	0	0	0	0	0	0	0
5.20E-05	0	2.71E-04	0	0	0.002909	0	6.48E-04	0	0.001639	0	3.80E-04	4.00E-05	3.36E-04
1.97E-04	1.94E-04	0	2.40E-05	3.60E-05	0	6.20E-05	0	1.07E-04	0	0	0	0	0
0	5.82E-04	2.04E-04	1.30E-05	1.41E-04	0	6.90E-05	5.70E-05	0	5.23E-04	2.40E-05	0	0.001614	0
4.30E-05	0	1.38E-04	1.50E-05	0	0.002339	3.00E-05	6.99E-04	0	0.002371	0	2.74E-04	0	3.01E-04
0	0	0	4.31E-04	0	0	0	0	0	0	0.010544	0	0	0
0	0	1.26E-04	1.90E-05	0.00191	0.001876	0	3.86E-04	0	0.001845	0	2.52E-04	7.90E-05	1.83E-04
0	2.50E-05	0	0.001508	0	2.31E-04	5.20E-05	6.60E-05	0	1.82E-04	0.003943	1.26E-04	2.40E-05	2.50E-05
0	0	0	2.80E-05	7.50E-05	0.001066	0	3.92E-04	0	0.001186	0	7.72E-04	3.50E-05	2.51E-04
7.40E-05	0	5.50E-05	1.90E-05	0	0.001583	1.70E-05	3.09E-04	0	6.36E-04	0	4.32E-04	0	1.06E-04
0	4.03E-04	1.86E-04	2.00E-05	0	1.03E-04	4.10E-05	6.60E-05	0	0	1.29E-04	0.001011	0	1.05E-04
0	0	5.00E-05	0.002889	0	0	0	0	0	0	0.013024	0	0	0
0	0	0	5.20E-05	0	0	0	0	0	0	0.026348	0	0	0
4.70E-05	3.90E-05	3.56E-04	2.60E-05	0	0.001	8.20E-05	2.00E-05	2.50E-05	5.18E-04	0	1.14E-04	2.02E-04	0
3.80E-05	0	2.05E-04	0	4.30E-05	5.03E-04	2.80E-05	2.47E-04	0	8.20E-04	0	5.75E-04	0	0
0	0	0	0	0	6.60E-05	0	0	0	6.20E-05	0	2.70E-05	0	0
0.012665	0.007657	0.02091	0.037308	0.022631	0.036316	0.006141	0.019933	0.007704	0.037084	0.068576	0.023266	0.017104	0.012705

H131	H141	H029	H102	H122	H009	H104	H021	H092	H051	H059	H078	H035	H076
0.11938	0.111941	0.10617	0.100837	0.100495	0.099897	0.098795	0.09748	0.095907	0.09159	0.08992	0.088421	0.087771	0.086075
0.144967	0.241613	0.170335	0.05537	0.108107	0.118788	0.08663	0.203384	0.16936	0.105669	0.146688	0.247447	0.177118	0.187771
0.089222	0.082367	0.067951	0.039765	0.163916	0.032761	0.097115	0.06477	0.070033	0.09227	0.036955	0.037035	0.050402	0.009223
0.047959	0.130257	0.076709	0.017216	0.031596	0.044561	0.026649	0.064023	0.06494	0.044775	0.016947	0.081323	0.04992	0.055368
0.197821	0.070088	0.016489	0.024864	0.014035	0.044368	0.006641	0.034765	0.033659	0.008673	0.002507	0.050392	0.008731	0.27655
0.036385	0.040061	0.019749	0.134836	0.059419	0.174969	0.058494	0.106357	0.116223	0.080839	0.029516	0.086687	0.113835	0.063668
0.043232	0.031038	0.110055	0.107406	0.042734	0.1018	0.10839	0.049378	0.150554	0.153463	0.005988	0.099361	0.105451	0.076983
0.032828	0.049357	0.039776	0.062557	0.022399	0.095035	0.063819	0.096483	0.061063	0.083413	0.050016	0.037033	0.166004	0.019958
0.018566	0.037773	0.053307	0.057242	0.302842	0.023597	0.068664	0.010118	0.046342	0.115285	0.031161	0.032465	0.072993	0.014865
0.023841	0.045852	0.017022	0.006302	0.008193	0.008808	0.018275	0.017013	0.006375	0.011619	0.010586	0.01731	0.009678	0.014924
0.010308	0.029848	0.011795	0.00592	0.011298	0.005169	0.010757	0.009097	0.003341	0.005577	0.006292	0.003215	0.005122	0.003597
0.004452	0.004408	0.002198	0.063727	0.006726	0.05586	0.013151	0.014677	0.014119	0.017165	8.40E-05	0.022659	0.003457	0.007711
0.004098	0.003284	0.005723	0.005065	0.009333	0.003945	0.07229	0.007103	0.014125	0.00762	0.005425	0.004472	0.006296	0.001184
0.015432	0.001566	0.011222	0.006581	0.003123	0.013313	0.01302	0.011579	0.010713	0.007535	8.20E-05	0.029734	0.014341	0.004852
0.005949	0.009086	0.002804	0.006454	0.003675	0.006989	0.003741	0.007492	0.006619	0.005767	0.0057	0.014912	0.006449	0.015628
0.009962	0.003973	0.010587	0.020161	0.007998	0.018619	0.00287	0.019676	0.008086	0.012284	0.001257	0.010129	0.005117	0.025349
0.004481	0.010993	0.004837	0.003746	0.003514	0.003339	0.003281	0.003937	0.007563	0.003456	0.005917	0.006157	0.009822	0.001654
3.62E-04	0.001407	1.18E-04	0.002176	0.004518	1.18E-04	0.074788	0.003751	2.25E-04	0.004559	0	8.53E-04	2.57E-04	9.88E-04
0.014001	1.95E-04	9.88E-04	0.008702	0.004017	0.015679	0.00842	0.004602	0.01085	0.009578	0.004412	0.044848	0.011761	0.023782
0.002813	0.002728	6.55E-04	0.001014	0.001376	0.003291	0.020541	0.001395	5.25E-04	0.005829	0	3.80E-04	3.17E-04	1.09E-04
0.001069	3.80E-05	0.003044	1.56E-04	0.001013	0.011218	0.008441	2.58E-04	8.30E-05	0.001469	0.099283	7.40E-05	0.001313	4.80E-05
0.002675	0.013945	0.004318	0.00135	0.003196	0.010571	0.002512	0.0061	0.003869	0.002794	0.002773	0.006612	0.006225	0.008194
0.007151	0.00236	0.002898	0.00105	0.008702	0.004832	0.045344	0.001738	0.001221	0.001863	0.019228	1.07E-04	0.001769	3.41E-04
0.005561	0.003551	0.008254	4.21E-04	0.003747	6.00E-05	2.60E-05	0.003658	8.94E-04	5.90E-04	5.82E-04	0.0019	1.70E-05	0.013816
0.00407	0.005108	0.002998	0.00389	0.003155	0.002102	0.004266	0.004759	0.001503	0.00192	0.002051	0.001638	5.79E-04	0.005455
0.024692	0.002509	0.002133	7.66E-04	6.47E-04	0.00204	3.72E-04	0.00159	0.002045	6.45E-04	0	0.009291	3.74E-04	0.015962
0.00147	0.005191	0.001711	0.002313	0.003214	0.004	0.002441	0.004016	0.009345	0.004965	1.78E-04	0.006583	0.011339	9.78E-04
0	0	0	0.193277	0.013631	0	6.78E-04	2.50E-05	0	3.33E-04	0	0	0	0
0.001941	0.004842	0.002399	9.35E-04	0.001627	0.004013	0.003022	0.00598	0.009886	0.003503	0.004092	0.002807	0.005666	0.007281
0.005575	8.33E-04	0.011985	0.006995	0.00258	0.006134	6.51E-04	0.003429	0.002588	0.004442	0.001882	0.005237	2.55E-04	0.002995
0.004714	9.18E-04	0.002247	0.006138	0.013542	0.001996	9.99E-04	0.00773	0.012842	0.005941	8.90E-05	0.001868	0.003575	0.003386
7.95E-04	0	0.001681	0	3.97E-04	0.00703	0.004523	1.35E-04	1.36E-04	0.001028	0.063046	3.50E-05	7.65E-04	2.40E-05
0.004808	0.010247	0.004273	3.40E-05	0.00159	0.001134	1.94E-04	1.73E-04	0.00192	6.13E-04	0	0.006305	0	0.02053
0.003855	0.011001	7.01E-04	5.37E-04	0.001601	0.001234	7.20E-04	0.018576	2.78E-04	6.10E-04	0	3.90E-05	6.31E-04	2.92E-04
2.29E-04	1.83E-04	1.47E-04	6.80E-05	3.01E-04	2.90E-05	8.11E-04	2.69E-04	0	1.12E-04	0	0	1.62E-04	0
6.60E-05	0	0.077401	0	7.40E-05	7.47E-04	3.08E-04	2.20E-05	0	1.47E-04	0.004968	0	3.60E-05	2.60E-05
0.005013	0.00114	7.58E-04	0.005932	0.001223	0.005084	0.001149	0.004359	0.003309	0.003133	0	0.001112	0.002039	0.005929
0.001832	0.003517	0.002614	7.84E-04	0.002275	0.00178	7.42E-04	0.002062	0.004529	0.004186	0	0.002327	0.008504	0.001784
0.006202	0.002684	0.002589	0.001053	7.55E-04	0.001147	1.99E-04	0.00415	0.003306	0.002547	4.90E-04	0.001724	6.75E-04	0.002016
0.005245	9.50E-04	0.002026	0.002305	0.002706	4.72E-04	0.002509	0.008998	0.004606	0.006064	4.31E-04	0.002402	0.003379	8.51E-04
1.41E-04	9.00E-05	0.023048	0.003689	8.30E-05	0.002089	9.13E-04	1.98E-04	3.80E-05	3.86E-04	0.012887	4.30E-05	1.60E-04	3.90E-05
3.97E-04	2.23E-04	6.16E-04	0.001801	5.40E-04	0.002679	3.38E-04	7.87E-04	4.44E-04	0.001851	0	0.010097	2.26E-04	7.75E-04
0.002227	0.002505	7.74E-04	0.001423	1.16E-04	0.002991	0.003187	0.001148	0.012919	0.004141	0	0.005532	3.44E-04	0.002223
2.35E-04	3.50E-05	0	0.001145	0	4.08E-04	4.60E-04	1.98E-04	5.43E-04	0.001585	0	3.90E-05	0	0
8.01E-04	3.20E-05	3.29E-04	7.60E-05	9.21E-04	3.57E-04	0.003483	1.70E-05	0	5.26E-04	0.059944	0	8.00E-05	2.90E-05
0.002525	3.32E-04	4.85E-04	0.004706	0.001249	0.003532	0.002272	0.003748	0.006927	0.007682	0.004843	3.05E-04	0.012151	0.001456
1.65E-04	2.40E-04	5.50E-05	0.005524	9.24E-04	0.008989	0.001404	0.003673	9.39E-04	0.001828	0	6.02E-04	0.00169	0.002509
3.18E-04	0	7.93E-04	0	1.82E-04	0.0035	0.002006	4.40E-05	0	5.39E-04	0.028393	0	0.003072	0
5.70E-05	0	0.021879	4.60E-05	4.40E-05	6.39E-04	3.49E-04	0	0	8.40E-05	0.004631	0	7.40E-05	0
2.06E-04	3.40E-05	1.79E-04	5.62E-04	6.20E-05	4.34E-04	0	7.52E-04	0.00278	0.00244	0	5.13E-04	5.64E-04	5.00E-05
0.005202	0.00706	3.58E-04	7.10E-05	1.72E-04	1.53E-04	0	2.40E-04	1.22E-04	3.00E-05	0	1.32E-04	4.30E-05	0.001669
0.001106	3.73E-04	0.001566	0.003416	0.001173	0.001587	0.004677	4.86E-04	0.001379	8.74E-04	0.001059	0.001923	7.01E-04	5.47E-04
0.00212	2.60E-04	9.70E-04	0.003352	0.00207	4.22E-04	6.81E-04	0.013985	0.002037	0.00335	0	8.04E-04	0.005187	3.44E-04
0.005966	5.79E-04	0.001374	4.72E-04	5.04E-04	2.20E-05	6.00E-04	0.003596	0.001715	0.002529	6.71E-04	2.45E-04	0.001829	6.01E-04
0	5.15E-04	0	4.90E-05	0	0	0	2.50E-05	3.55E-04	0	0	9.47E-04	1.79E-04	0

0.002294	5.02E-04	7.72E-04	2.66E-04	2.91E-04	1.08E-04	2.73E-04	0.001424	3.46E-04	1.82E-04	0	8.70E-05	7.00E-04	8.90E-05
7.34E-04	8.10E-05	3.93E-04	1.15E-04	9.52E-04	0.001164	0.003472	2.08E-04	3.10E-05	8.56E-04	0.0189	0	1.37E-04	2.50E-05
3.38E-04	4.00E-04	8.50E-05	3.20E-05	2.60E-05	0	0	7.30E-05	0	2.20E-05	0	0	2.00E-05	2.60E-05
3.92E-04	4.10E-05	4.97E-04	0	5.86E-04	6.70E-04	0.002381	2.50E-05	0	3.47E-04	0.021505	0	0	2.20E-05
0.002031	0	3.55E-04	0	1.23E-04	0.001576	0.001057	2.30E-05	0	2.58E-04	0.011109	4.10E-05	2.47E-04	2.00E-05
0	0	0	0	0	0	0	0.021526	0	0.023639	0	0	0	0
1.52E-04	0	2.20E-04	0	5.10E-05	0.001338	0.001269	5.20E-05	0	1.11E-04	0.0106	0	0.001588	0
0	5.60E-05	0	4.20E-05	0	0	0	2.10E-05	1.24E-04	0	0	2.99E-04	1.45E-04	0
0.02137	5.30E-05	3.60E-05	0	0	0	0	2.01E-04	0	0	0	1.45E-04	1.60E-05	0.00112
1.54E-04	0	3.71E-04	0	8.30E-05	9.32E-04	9.01E-04	6.10E-05	0	1.86E-04	0.009004	0	3.70E-05	0
0	0	0.002906	0	0	4.90E-05	0	0	0	0	0	0	0	0
1.05E-04	0	1.58E-04	0	8.80E-05	0.001215	7.30E-04	5.89E-04	3.60E-05	7.97E-04	0.007213	0	0	0
1.40E-05	0	0.003279	0	5.80E-05	5.60E-05	1.43E-04	2.50E-05	4.70E-05	0	6.46E-04	0	1.90E-05	2.70E-05
5.40E-05	0	1.91E-04	0	3.60E-05	4.79E-04	3.13E-04	1.80E-05	0	5.70E-05	0.00571	0	0	0
7.50E-05	0	3.51E-04	0	5.00E-05	8.74E-04	4.55E-04	6.60E-05	0	8.60E-05	0.005857	4.20E-05	1.70E-04	0
0	0	2.70E-05	2.40E-04	0	5.43E-04	1.77E-04	4.80E-05	0	2.56E-04	0	1.62E-04	0	1.12E-04
0	0	0.012023	0	0	0	0	0	0	2.20E-05	0	0	0	0
0	0	9.94E-04	0	0	0	0	0	0	0	0	0	0	0
3.47E-04	0	1.70E-05	3.90E-05	4.43E-04	1.78E-04	0.001384	0	0	3.40E-05	0	4.40E-05	1.20E-05	2.50E-05
7.30E-05	0	5.60E-05	0	3.24E-04	4.90E-04	8.63E-04	2.50E-05	0	2.39E-04	0.00597	0	0	0
0.012106	0	0	0	0	0	0	1.23E-04	0	0	0	0	0	0
0.025273	0.009737	0.062176	0.014989	0.013559	0.025997	0.029974	0.041488	0.016236	0.031192	0.142512	0.013104	0.018464	0.008145

H003	H065	H099	H036	H060	H058	H098	H071	H120	H126	H091	H116	H066	H142
0.074233	0.072723	0.064873	0.064307	0.057949	0.057738	0.053695	0.048119	0.046864	0.046251	0.045224	0.044149	0.04124	0.041071
0.173151	0.089012	0.163244	0.378238	0.263805	0.190834	0.179211	0.256932	0.185267	0.228549	0.20674	0.22174	0.115703	0.166961
0.16245	0.111145	0.068425	0.032536	0.048608	0.03305	0.099117	0.048472	0.076538	0.075471	0.079026	0.010106	0.271903	0.067573
0.039297	0.053297	0.03253	0.125776	0.042168	0.032244	0.087582	0.080228	0.044496	0.102461	0.190473	0.103716	0.04857	0.239383
0.013415	0.011129	0.032192	0.038396	0.035559	0.039371	0.073266	0.108192	0.006021	0.026966	0.037864	0.126458	0.017642	0.076468
0.058517	0.059256	0.037914	0.095947	0.033044	0.03874	0.030769	0.038293	0.030526	0.080636	0.044333	0.099478	0.044874	0.054217
0.074396	0.168717	0.231133	0.049861	0.179217	0.018596	0.127692	0.12309	0.081579	0.076065	0.064795	0.085139	0.038311	0.044487
0.060066	0.067093	0.025398	0.025807	0.020176	0.0238	0.009047	0.036319	0.013574	0.076315	0.039003	0.029385	0.049127	0.031448
0.100679	0.177465	0.04888	0.027137	0.042882	0.016661	0.12161	0.013396	0.133697	0.033342	0.021679	0.012505	0.063967	0.047271
0.023884	0.017583	0.014173	0.022865	0.025891	0.02261	0.018255	0.009327	0.017255	0.027759	0.051268	0.031429	0.013681	0.044374
0.03306	0.015645	0.003713	0.009156	0.013784	0.011524	0.016301	0.007257	0.0394	0.005898	0.002548	0.002416	0.025821	0.018533
0.006773	0.014304	0.010656	0.005999	0.005996	0.004357	0.002548	0.003842	0.003287	0.009882	0.013718	0.023959	0.003101	0.015349
0.01428	0.010411	0.006285	0.006005	0.001161	0	4.83E-04	0.001498	0.00419	0.00511	0.004788	0.003118	0.005665	0.009997
6.33E-04	0.003166	0.012488	0.004227	0.003334	0.004362	0.009809	0.012048	8.91E-04	0.006122	0.005584	0.027179	0.001861	0.014668
0.003024	0.002429	0.001863	0.020381	0.005941	0.003042	0.006596	0.017311	0.004081	0.014051	0.004005	0.019578	0.001912	0.012098
0.004475	0.00468	0.030809	0.00558	0.016919	0.006855	0.019103	0.027122	0.004945	0.010159	0.018767	0.00739	0.006889	0.004747
0.00511	0.009128	0.002826	0.004142	0.004004	0.008553	0.002925	0.001255	0.005423	0.005439	0.004646	0.002127	0.006928	0.01129
0.05713	0.005277	0.003104	1.74E-04	0.002724	0	0.01027	0.016972	0.232557	0.001362	0	1.50E-05	0.00296	0.002222
1.68E-04	0.007186	0.011511	0.007268	0.001085	0.003079	0.051074	0.02666	8.73E-04	0.010793	0.00169	0.001985	6.91E-04	0
0.005067	0.009567	0.018433	0.002019	8.94E-04	3.93E-04	7.66E-04	3.19E-04	6.00E-04	7.37E-04	0.003037	6.10E-05	0.001766	0.02078
0.009185	0.002125	0.003702	0	0.017302	0.098958	7.94E-04	0.002976	0.001598	4.22E-04	0.003076	8.90E-05	0.013201	0
0.003801	0.002225	0.003025	0.006483	0.00565	0.004478	0.006614	0.006014	0.003365	0.004024	0.003274	0.007654	0.003149	0.006567
0.008138	0.00927	0.05066	0	0.012265	0.011747	0.006891	5.47E-04	0.005473	0.002539	0.010891	3.66E-04	0.054544	0.002412
5.43E-04	0.001483	0.008788	0.009568	0.008001	0.001901	0.008819	0.018306	0.003151	0.001095	0.002579	0.00389	0.001482	0.005974
0.003236	0.001047	3.15E-04	0.010729	0.007315	0.004524	0.004223	0.005143	0.002711	0.002786	0.001404	0.010779	3.10E-04	0.005396
5.89E-04	2.57E-04	0.007403	2.35E-04	0.00218	0.00197	0.006909	0.013268	6.85E-04	0.002032	0.006219	0.012568	0.001294	0.004892
0.003667	9.70E-04	0.002513	0.006202	0.004131	0.001764	0.00135	0.004582	0.002375	0.004823	0.003066	0.005961	0.001022	0.001242
0	3.63E-04	2.46E-04	0	3.60E-05	0	0	2.40E-04	0	0	0	0	0	0
0.002198	0.002076	0.003725	0.004995	0.002555	0.001866	0.003118	0.011788	0.002493	0.006939	0.004506	0.007664	7.54E-04	0.003405
6.67E-04	0.00256	0.014709	0.009473	0.001488	0.003643	8.50E-04	0.004662	0.001511	0.005523	6.18E-04	0.008846	0.002521	0.007699
0.00569	0.010277	0.012546	0.00288	0.006197	0.002492	0.001718	0.005324	0.007751	0.006185	0.003348	5.57E-04	0.009167	0.00339
0.004895	0.001331	0.00242	5.60E-05	0.010669	0.060968	6.02E-04	9.08E-04	0.00105	2.45E-04	0.002002	1.33E-04	0.008004	5.10E-05
0.001006	5.74E-04	0.00414	1.82E-04	0.003429	6.97E-04	0.009685	0.008254	0.00107	0.005143	9.35E-04	0.053977	0.001558	0.002006
0.001284	0.001923	7.72E-04	1.71E-04	0.000649	0.001017	5.73E-04	5.20E-04	7.77E-04	7.20E-04	0.004926	1.95E-04	0.007772	6.95E-04
6.75E-04	3.41E-04	0.002017	0	5.04E-04	0	1.58E-04	3.30E-05	1.16E-04	1.76E-04	5.86E-04	2.20E-05	0.003937	1.46E-04
4.37E-04	1.63E-04	3.23E-04	6.90E-05	0.001059	0.005105	3.50E-05	0	4.90E-05	0	2.57E-04	0	8.52E-04	2.40E-05
3.62E-04	9.74E-04	0.004336	0.002349	0.004819	0.00456	0.001046	0.001697	3.17E-04	0.003339	0.002576	0.006046	0.002773	0.001477
0.002023	4.01E-04	9.64E-04	0.004219	0.001525	0.001307	0.001355	0.005889	0.002356	0.006284	0.00093	0.004709	6.20E-04	0.003057
8.82E-04	8.05E-04	0.002486	0.001809	0.001073	3.08E-04	0.007691	0.003302	0.002531	0.004522	0.004223	0.004121	8.96E-04	0.003067
1.08E-04	0.0024	9.33E-04	2.34E-04	0.006673	0.003827	9.81E-04	0.002104	0.006332	0.039478	0.006175	8.76E-04	0.009377	3.06E-04
0.001044	2.02E-04	9.15E-04	7.10E-05	0.00266	0.010946	1.68E-04	3.13E-04	2.49E-04	2.63E-04	2.71E-04	1.60E-05	0.002015	5.80E-05
4.82E-04	9.90E-04	6.02E-04	3.58E-04	0	0	3.89E-04	2.85E-04	1.51E-04	1.85E-04	6.51E-04	1.43E-04	2.83E-04	0.003636
0	0.005523	2.50E-05	0	4.10E-04	0	0	0.002775	0	0.001155	0.002139	2.74E-04	8.51E-04	1.18E-04
0	0.00536	0.001801	1.61E-04	0	0	0	3.30E-05	0	8.10E-05	0	0	0	0
0.001044	0.00126	0.002073	1.80E-04	0.008705	0.023921	5.30E-04	0	9.82E-04	2.27E-04	9.92E-04	2.00E-05	0.007972	2.10E-05
0.001601	0.001321	0.001562	0.001709	0.002595	0	1.32E-04	0.001016	8.52E-04	0.005686	0.004773	3.61E-04	0	3.35E-04
5.48E-04	0.001224	6.94E-04	1.09E-04	3.50E-04	9.55E-04	3.60E-05	4.30E-05	8.50E-05	0.003868	4.13E-04	0.001639	0	1.86E-04
0.002151	7.33E-04	9.14E-04	1.23E-04	0.004385	0.021755	3.17E-04	3.86E-04	5.25E-04	2.40E-05	9.89E-04	1.30E-05	0.003208	1.90E-05
4.41E-04	5.90E-05	2.01E-04	0	0.001016	0.002502	1.52E-04	3.66E-04	5.50E-05	0	1.21E-04	0	4.21E-04	0
0.002469	0.001933	0.00156	5.76E-04	0	0.001386	0	6.10E-05	2.00E-05	9.43E-04	9.20E-05	0	0	1.64E-04
8.34E-04	4.61E-04	2.16E-04	0	0.0011	0.005064	1.25E-04	2.80E-05	3.00E-04	7.88E-04	0.012693	6.47E-04	0.00411	0.009133
0.001046	0.010264	0.001243	3.90E-04	2.11E-04	0	0.002224	2.55E-04	4.13E-04	0.002355	0.001745	8.59E-04	4.58E-04	0.001435
8.70E-05	3.11E-04	2.70E-05	0.001028	0.003215	1.02E-04	3.30E-05	7.34E-04	0.001506	0.004708	0.001419	5.28E-04	0.003462	2.00E-05
7.10E-05	4.97E-04	7.47E-04	1.35E-04	2.50E-04	0.001211	1.96E-04	0.002583	6.15E-04	8.87E-04	0.005877	7.19E-04	0.002887	2.00E-04
0	1.60E-05	0	3.57E-04	0	0	0	2.34E-04	8.40E-05	2.90E-05	0	2.90E-05	0	0.001179

4.81E-04	6.20E-05	7.31E-04	1.16E-04	7.86E-04	0.001619	0	2.77E-04	2.77E-04	4.68E-04	0.022927	4.30E-05	0	1.61E-04
0.001246	8.48E-04	0.002865	0	0.007715	0.007012	6.38E-04	3.50E-05	9.12E-04	1.61E-04	8.10E-04	0	0.007423	0
3.03E-04	1.90E-05	6.50E-05	5.70E-05	0	9.90E-05	4.20E-05	3.50E-05	6.61E-04	0	0.002768	2.00E-05	7.26E-04	5.21E-04
9.06E-04	4.63E-04	0.001706	0	0.005626	0.02132	4.24E-04	3.90E-05	6.27E-04	9.00E-05	4.30E-04	0	0.005952	0
7.98E-04	2.82E-04	2.91E-04	5.40E-05	0.001437	0.00981	6.90E-05	3.23E-04	1.34E-04	2.70E-05	9.27E-04	3.70E-05	0.001577	2.10E-05
0	0	0	0.004193	0	0	0	0	2.49E-04	8.90E-05	0	1.30E-05	0	0
0.001372	3.03E-04	3.23E-04	0	0.002226	0.010805	1.65E-04	0	2.20E-04	2.70E-05	3.81E-04	2.10E-05	0.001102	0
0	0	0	7.70E-05	0	0	0	0	0	0	0	8.70E-05	0	6.30E-05
0	0	0	0	0	0	4.03E-04	3.20E-05	0	4.70E-05	2.01E-04	1.51E-04	7.31E-04	0
0.001063	1.10E-04	2.36E-04	0	0.001854	0.010278	1.02E-04	0	1.96E-04	0	2.10E-04	0	0.001375	0
0	0	0	0	0	0	0	0	0	0	0	0	0	0
0.001068	1.36E-04	3.47E-04	0	0.001669	0.008638	5.40E-05	0	1.40E-04	2.47E-04	8.00E-05	1.60E-05	0.001257	0
8.70E-05	9.20E-05	0	0	9.00E-05	3.86E-04	4.20E-05	1.53E-04	1.89E-04	2.60E-05	1.49E-04	0	4.84E-04	0
3.41E-04	1.17E-04	1.34E-04	0	9.74E-04	0.005045	1.38E-04	6.60E-05	9.50E-05	1.70E-05	1.38E-04	0	9.22E-04	0
7.70E-04	1.49E-04	3.07E-04	0	0.001108	0.004749	6.60E-05	2.90E-05	1.47E-04	6.20E-05	2.20E-04	0	5.39E-04	0
0	1.21E-04	0	1.16E-04	0	0	3.70E-05	1.10E-04	0	6.56E-04	0	0	1.35E-04	0
0	0	0	0	0	0	0	0	0	0	0	0	0	0
0	0	0	0	0	0	0	0	0	0	0	0	0	0
3.58E-04	2.19E-04	0.001689	4.50E-05	4.86E-04	0	4.56E-04	0	1.22E-04	0	1.57E-04	0	0.021492	2.20E-05
3.72E-04	2.34E-04	1.47E-04	0	0.001113	0.001221	1.82E-04	3.90E-05	8.40E-05	0	1.43E-04	0	0.001674	0
0	0	0	0	0	4.79E-04	0	0	0	0	8.02E-04	0	0	3.40E-05
0.019823	0.015913	0.032106	0.00467	0.047358	0.123756	0.009349	0.017541	0.012335	0.037241	0.036703	0.013978	0.043099	0.007931

H139	H011	H008	H053	H100	H032	H073	H039	H079	H019	H130	H041	H125	ID...96
0.032768	0.026782	0.025357	0.021742	0.017473	0.014875	0.014747	0.012477	0.01223	0.011099	0.008956	0.008395	0.004286	Neisseria
0.120755	0.24922	0.422988	0.20971	0.327966	0.153314	0.356829	0.221384	0.364998	0.371572	0.398245	0.359613	0.285189	Prevotella
0.162873	0.081617	0.059741	0.011609	0.093932	0.046046	0.110028	0.045382	0.035469	0.0499	0.104895	0.010335	0.006316	Streptococc
0.256954	0.114663	0.103679	0.00545	0.125034	0.073577	0.107047	0.100162	0.182396	0.13266	0.172886	0.151409	0.0634	Veillonella
0.008147	0.015626	0.069994	0.002081	0.054538	0.091447	0.046029	0.087173	0.050313	0.042006	0.05206	0.147774	0.065704	Leptotrichia
0.017666	0.020032	0.018019	9.23E-04	0.023653	0.086468	0.019596	0.165585	0.055486	0.061179	0.013134	0.011699	0.004519	Fusobacteri
0.028843	0.098921	0.087014	0.002406	0.105068	0.04216	0.080563	0.086287	0.069292	0.125147	0.044226	0.036492	0.016911	Alloprevote
0.02049	0.014802	0.00467	0.006551	0.002108	0.0169	0.016547	0.066414	0.011881	0.005754	0.010496	0.001839	0.001707	Porphyromo
0.130926	0.014844	0.011085	0.004541	0.042906	0.010794	0.032837	0.024439	0.009212	0.014849	0.009816	0.003457	0.003771	Haemophilu
0.075492	0.030218	0.021047	5.92E-04	0.024088	0.033397	0.033634	0.019659	0.020225	0.024684	0.023573	0.039701	0.013023	Actinomyce
0.019654	0.027769	0.00232	3.51E-04	0.004476	0.004963	0.009075	0.002644	0.009043	0.009016	0.004132	0.001753	5.69E-04	Rothia
0.005331	0.006319	3.18E-04	3.55E-04	8.21E-04	0.019637	0.004741	0.012284	6.17E-04	0.00307	0.002079	4.11E-04	6.90E-05	Capnocytop
0.015797	0.001466	0.002045	3.51E-04	0.004329	0.003914	0.001543	0.005536	0.001219	0.002497	0.003033	3.22E-04	7.90E-05	Gemella
0.016042	4.58E-04	0.020668	3.26E-04	0.04667	0.006407	0.026897	0.009819	0.017629	0.008444	0.022089	0.043153	0.018762	TM7x
0.004509	0.001909	0.009261	1.06E-04	0.01328	0.011184	0.011655	0.015358	0.015665	0.015047	0.006322	0.017166	0.007369	Lachnoanae
0.004628	0.012987	0.012643	0	0.01351	0.011336	0.010112	0.007304	0.020671	0.023212	0.00337	0.004755	0.002317	Campyloba
0.008889	0.007122	0.002119	0	0.004559	0.001405	0.008708	0.006349	0.00445	0.005534	0.007492	0.001316	5.82E-04	Granulicate
0.005782	0.006548	0.002813	0	1.03E-04	0.001866	0.001171	1.47E-04	6.60E-05	6.66E-04	5.92E-04	3.20E-05	0	Actinobacil
1.47E-04	3.19E-04	2.95E-04	0.001653	0.002719	0.006778	3.60E-05	2.52E-04	0.016141	2.70E-04	3.30E-05	8.90E-05	2.19E-04	g__Abscon
0.011427	0.009872	7.91E-04	0	6.80E-04	0.002924	0.00205	3.80E-04	0	4.30E-04	0.001998	3.05E-04	0	Lautropia
3.12E-04	0.022654	4.29E-04	0.151285	0.001075	0.002817	1.90E-04	2.50E-05	1.10E-04	1.03E-04	4.80E-05	6.70E-05	0.115235	Bacteroides
0.004281	0.001525	0.00739	0	0.010413	0.002966	0.009483	0.007667	0.008021	0.008567	0.005615	0.00742	0.003947	Oribacteriu
0.003951	0.004061	8.97E-04	0.017083	0.003396	0.001571	9.85E-04	1.60E-04	1.38E-04	5.59E-04	6.28E-04	7.25E-04	0.006143	Pseudomona
0.004591	0.006983	0.033493	8.04E-04	0.008667	0.00171	0.009641	0.008348	0.032959	0.0163	0.012066	0.04702	0.02177	Megasphaer
0.001096	6.47E-04	0.007281	0.002055	0.015405	9.66E-04	0.005333	0.04148	0.012633	0.012136	0.00358	0.007037	0.003653	Stomatobac
3.46E-04	4.38E-04	0.00402	2.29E-04	0.007034	0.018768	0.006214	0.005246	0.003864	0.00441	0.012583	0.013986	0.004387	Selenomona
4.49E-04	0.001584	7.72E-04	0	0.002127	0.002287	0.001616	0.005769	0.004297	0.001625	5.09E-04	0.001606	0.001106	Peptostrepto
0	3.71E-04	0	0	0	2.60E-05	0	0	0	0	0	0	6.80E-05	Moraxella
0.002344	0.006997	0.011193	0	0.001832	0.003121	0.003137	0.00214	0.004047	0.009167	0.004924	0.009271	0.003862	Solobacteriu
0.002173	0.001067	0.005071	0.004002	0.002575	0.009664	0.006668	0.011616	0.003238	0.001427	0.001091	0.006449	0.005946	Clostridia_U
2.29E-04	0.001168	4.12E-04	1.18E-04	3.85E-04	0.009928	0.002872	9.27E-04	0.001358	4.01E-04	0.007481	2.27E-04	2.01E-04	Aggregatiba
3.70E-05	0.014931	1.76E-04	0.089917	5.05E-04	3.53E-04	2.00E-05	5.30E-05	8.30E-05	1.28E-04	0	6.10E-05	0.064617	Faecalibacte
0	0	0.013285	1.29E-04	0.011457	5.99E-04	0.007773	0.004901	0.004271	0.006846	0.010126	0.018266	0.008855	Lachnospira
6.20E-05	0.046813	4.54E-04	0	1.29E-04	0.059651	0.001515	4.96E-04	2.77E-04	4.74E-04	0.001478	3.26E-04	1.50E-04	Corynebacte
3.07E-04	3.09E-04	2.00E-05	0	1.31E-04	2.18E-04	1.05E-04	0	0	0	0	3.40E-05	0	Halomonas
0	0.001569	0	0.017348	2.80E-05	0.060334	0	1.05E-04	0	4.70E-05	0	0	0.005258	Muribacula
7.18E-04	0.0015	3.64E-04	0	5.98E-04	0.029053	5.24E-04	0.003481	0.001387	0.005544	3.81E-04	2.77E-04	1.52E-04	Tannerella
0.001634	0.00365	0.002709	1.11E-04	0.003496	0.001578	0.003413	0.001814	0.003214	0.003666	0.001168	0.004294	0.001924	[Eubacteriu
0.007978	0.005913	0.020647	0	0.002898	0.001087	0.005754	0.001306	0.006065	0.01023	0.006805	0.006945	0.002511	Atopobium
0.003476	0.001308	3.25E-04	0	3.67E-04	0.002932	0.00661	9.10E-04	5.73E-04	7.40E-04	0.002936	0	0	Treponema
4.10E-05	0.003054	5.80E-05	0.019546	1.43E-04	0.016	1.31E-04	1.60E-05	0	0	0	2.40E-05	0.013823	Lachnospira
4.75E-04	4.65E-04	9.40E-05	0	2.40E-05	6.20E-05	7.04E-04	2.20E-04	7.60E-05	1.12E-04	1.24E-04	9.00E-05	0	Neisseriace
0	1.63E-04	9.44E-04	0	0.001184	0.006509	0.002054	4.00E-05	0.003578	0.001348	0.010571	6.79E-04	7.60E-05	Saccharimo
0	7.37E-04	2.20E-05	0	0	2.40E-05	0	0	1.99E-04	0	4.00E-05	0	0	Abiotrophia
0	0.001418	9.90E-05	0.050871	4.15E-04	1.34E-04	3.80E-05	0	0	5.20E-05	0	0	0	Rhodococcu
8.30E-05	0.001021	4.50E-05	0	5.20E-05	9.10E-04	9.50E-04	4.78E-04	9.57E-04	6.37E-04	0.001048	7.60E-05	7.80E-05	Parvimonas
7.00E-05	0	6.40E-05	1.13E-04	1.75E-04	0.00736	1.96E-04	2.09E-04	0	4.80E-05	1.01E-04	1.03E-04	7.40E-05	Johnsonella
0	0.00829	1.50E-04	0.040672	2.28E-04	1.38E-04	0	0	0	1.90E-05	0	3.60E-04	0.035719	Fusicatenib
0	0.001037	0	0.005776	2.60E-05	0.011379	0	0	0	0	0	6.30E-05	0.005235	Lachnospira
0.001651	3.74E-04	5.47E-04	0	2.07E-04	0.001245	1.43E-04	0	1.10E-04	0	2.43E-04	0	0	Lactobacilla
6.30E-05	0.028385	3.00E-05	0	2.00E-04	5.00E-05	1.80E-04	5.81E-04	3.54E-04	1.50E-05	0.001448	0	0	F0332
7.74E-04	0.001187	7.44E-04	0	7.95E-04	0.001193	2.40E-04	5.26E-04	2.08E-04	1.91E-04	0.001365	6.50E-05	0	Bergeyella
0.001931	4.49E-04	4.30E-05	0	6.50E-05	3.43E-04	0.002508	6.11E-04	6.06E-04	1.89E-04	3.82E-04	0	0	Filifactor
2.90E-05	4.70E-04	0.001525	4.53E-04	2.50E-05	0.019551	0.001669	9.70E-04	0.001842	6.57E-04	0.002002	0.004474	0.002538	Dialister
0.001533	0	0	0	0	0	6.42E-04	4.43E-04	3.56E-04	1.15E-04	5.04E-04	5.50E-05	1.49E-04	P5D1-392

5.20E-05	1.13E-04	3.90E-05	0	1.23E-04	0.009269	5.85E-04	2.70E-04	5.70E-05	2.30E-05	6.64E-04	1.58E-04	1.37E-04	Saccharimo
1.71E-04	0.001612	1.22E-04	0.024743	3.34E-04	2.49E-04	2.90E-05	2.90E-05	0	4.30E-05	0	2.20E-05	0	Acinetobact
7.87E-04	0.002152	1.50E-05	0	9.00E-05	0.001039	3.97E-04	1.34E-04	3.30E-05	1.50E-04	6.50E-05	4.10E-05	0	Kingella
0	0.002876	6.80E-05	0.029611	4.06E-04	1.64E-04	0	0	0	4.60E-05	0	0	0	Sphingomor
0	0.002456	7.35E-04	0.016153	1.86E-04	5.20E-05	3.30E-05	0	0	2.30E-05	3.90E-05	4.80E-05	0.011602	Bifidobacte
0	0.022632	0	0	0	0	0	0	0	0	0	0	0	Leptotrichia
0	0.00354	5.20E-05	0.016662	3.80E-05	1.04E-04	0	0	0	6.90E-05	4.10E-05	2.50E-05	0.01368	Megamonas
1.40E-05	0	0	0	0	0	2.70E-05	5.10E-05	0	0	1.23E-04	2.80E-05	0	Burkholderi
0	5.80E-05	0.002266	0	0.001599	0	0.002177	1.18E-04	0	0	0.00405	0.01901	0.008437	Selenomona
0	0.002517	5.30E-05	0.014566	1.00E-04	6.60E-05	0	0	0	0	0	0	0.013068	[Eubacteriu
0	0	0	0	0	0.001226	0	0	0	0	0	0	0	Helicobacte
0	0.002027	2.40E-05	0.010294	5.30E-05	2.50E-05	0	0	0	0	0	0	0.008062	Christensen
0	1.05E-04	0	4.76E-04	0	0.002714	0	0	0	0	1.08E-04	0	3.63E-04	Lactobacillu
2.70E-05	0.001577	3.30E-05	0.018777	2.10E-05	2.60E-05	2.20E-05	2.70E-05	0	0	0	0	0.004768	Phascolarct
0	0.001838	0	0.010369	4.10E-05	5.70E-05	0	2.90E-05	0	0	0	0	0.007414	Agathobact
0	0	0	0	0	8.40E-05	0	0	0	0	0	0	0	Gracilibacte
0	0	0	0	0	0.002216	0	7.60E-05	0	1.70E-05	0	0	6.70E-05	Prevotellace
0	0	0	0	0	9.64E-04	0	0	0	0	0	0	2.22E-04	Mucispirillu
2.23E-04	5.20E-05	1.50E-05	0	7.70E-05	4.90E-05	0	0	0	0	0	7.20E-05	0	Serratia
0	4.29E-04	6.90E-05	0.010038	1.80E-04	6.20E-05	2.10E-05	0	0	0	0	0	0	Acidovorax
0	7.30E-05	0	0	0	1.27E-04	0	0	0	0	3.68E-04	0	0	Propionibac
0.010972	0.043911	0.006339	0.179052	0.012782	0.067588	0.021556	0.009693	0.008086	0.00684	0.015868	0.01058	0.129911	Others

CC65	CC73	CC62	CC69	CC63	CC70	CC25	CC68	CC16	CC37	CC58	CC43	CC21	CC30
0.76896	0.712166	0.588361	0.581617	0.53836	0.488067	0.369455	0.363708	0.29854	0.253396	0.19576	0.18649	0.162127	0.141807
0.022522	0.033508	0.021991	0.079106	0.01582	0.112757	0.038868	0.012544	0.027723	0.059075	0.011941	0.006507	0.012298	0.050745
0.055051	0.010899	0.024237	0.031311	0.012657	0.027925	0.068849	0.366157	0.378677	0.221889	0.375883	0.172673	0.226931	0.152194
0.014371	0.018766	0.061172	0.041511	0.020623	0.11058	0.099727	0.06675	0.017243	0.131963	0.026157	0.30061	0.009071	0.192464
0.055568	0.052672	0.148817	0.004042	0.034344	0.03109	0.123741	0.059704	0.03328	0.017405	0.217451	3.38E-04	0.053236	0.280808
0.010812	0.042391	0.016534	0.104324	0.13533	0.03576	0.027109	0.00773	0.007419	0.012371	0.002737	6.57E-04	0.048074	0.006261
0.00146	0.001361	7.89E-04	0.001524	3.42E-04	9.87E-04	6.50E-04	0.001155	6.17E-04	6.25E-04	2.75E-04	1.35E-04	0.001637	0.004186
9.33E-04	0.003192	0.004958	0.05438	0.02101	0.020539	0.002305	0.005847	0.002278	0.019843	0.018953	0.003964	0.080374	0.001092
0.003558	0.051725	1.31E-04	0.004502	0.002743	0.002988	0.003254	0.0184	0.005518	0.006844	0.003063	2.13E-04	0.07035	0.004543
0.00559	0.004164	0.087244	0.004196	0.010516	0.003576	0.122201	0.00254	0.004505	0.084494	0.014562	0.004288	0.004655	0.072471
0.007715	0.004328	0.012071	0.039911	0.013982	0.020485	0.023616	0.006506	0.006337	0.032066	0.030073	0.001214	0.010611	0.009664
0.008144	0.003441	0.002563	0.00311	0.072033	0.002542	0.062626	0.003325	8.58E-04	0.006653	0.021126	0.006478	0.015858	0.013338
9.80E-04	0.007138	5.15E-04	8.49E-04	0.002594	0	0.00334	0.003358	0.102093	0.041794	0.012721	1.30E-05	0.062582	0.001569
0.002007	0.00171	4.12E-04	0.001367	0.001868	0	7.50E-04	0.00231	8.90E-05	0.002559	0.00143	1.00E-05	0.001262	0.005624
0.004812	0.008841	0.010999	0.011927	0.03242	0.004899	0.006938	0.008084	0.001853	0.006611	0.0019	3.50E-05	0.003918	0.006954
2.36E-04	0.005207	3.42E-04	2.14E-04	1.29E-04	0.048873	0.001065	0.001147	0.009752	0.001288	7.15E-04	8.47E-04	7.34E-04	0.003412
0.001557	0.006508	0.001277	0.001373	0.003129	0.023396	0.00345	0.001685	0.006004	0.013535	0.004179	0.020427	0.015261	0.002004
3.90E-05	0	0	6.40E-05	0	0	0	0.001277	1.00E-05	0	0	0	0.016482	0
0.001647	0	4.20E-05	5.80E-04	0.003066	0	2.70E-04	6.00E-05	0	0.002555	0	0	6.26E-04	1.47E-04
0.005534	0	5.62E-04	0.00228	0.003511	0.0069	0.001815	2.54E-04	2.80E-05	0.004029	0.018499	0.123697	0.022532	8.70E-05
0	0	0	0	0	0	0	0	0.002481	6.10E-05	0	0.001482	9.00E-05	0
0.001155	0.001009	0.002832	0.002783	0.016824	5.60E-05	0.001806	0.001996	0.001605	0.001409	0.001016	0	0.00526	0.001677
0	0	0	0	0	2.00E-05	0	0	7.20E-05	0	0	2.75E-04	6.80E-05	0
4.10E-05	9.80E-05	0	9.50E-05	0	0.002397	3.85E-04	0	9.61E-04	3.00E-04	1.88E-04	2.56E-04	0	2.02E-04
1.44E-04	8.97E-04	4.44E-04	0.002979	0.002696	7.42E-04	0.00177	2.67E-04	7.31E-04	0.002762	3.25E-04	1.10E-05	6.15E-04	0.002552
0.003051	1.02E-04	4.55E-04	2.31E-04	0.001162	0.005612	0.002023	0.009631	0.001795	0.003321	2.94E-04	8.00E-05	2.02E-04	0.011978
0.001105	0	3.02E-04	0.004546	0.006703	0.003011	0.001583	0.001069	0.005294	0.00486	1.09E-04	2.80E-05	0.003228	0.002097
0	0	0	0	5.90E-05	0	0	0	0	0	0	0	0	0
9.06E-04	0.002891	8.82E-04	0.001008	0.003393	0.00359	0.002563	1.31E-04	2.24E-04	0.00155	1.55E-04	3.00E-05	0.001352	0.003534
0.00388	1.78E-04	3.00E-04	9.11E-04	1.68E-04	0	0.001304	0.004095	5.44E-04	5.17E-04	7.00E-04	2.90E-05	5.28E-04	0.003349
0	0.003781	3.90E-05	0	0	0	0	4.98E-04	1.50E-05	0	0.002691	0	0.005909	4.00E-05
0	0	0	0	0	0	0	0	0.00093	0	0	6.09E-04	2.40E-05	0
0.002295	6.53E-04	0	0	8.60E-05	0	0	0.009738	9.50E-05	8.62E-04	0.001242	0	8.30E-05	3.90E-05
4.10E-05	1.44E-04	3.10E-05	1.12E-04	4.41E-04	0.023942	0.00404	0	0.002134	1.43E-04	0.003645	0	0.003752	1.39E-04
6.17E-04	0.003175	1.90E-04	6.60E-05	8.70E-05	0.003929	2.63E-04	0	0.026888	0.001362	7.54E-04	0.044794	0.071493	3.39E-04
0	0	0	0	0	0	0	0	0	0	0	0	0	0
8.92E-04	7.91E-04	1.99E-04	0.002525	0.008591	2.00E-05	2.52E-04	0	3.80E-04	1.32E-04	0.002814	5.90E-05	0.001516	6.23E-04
0.001426	0.001416	0.001323	0.002016	0.014619	2.70E-05	0.002571	9.91E-04	1.60E-04	0.002201	7.90E-04	0	3.96E-04	0.00268
1.76E-04	0.001325	2.30E-04	3.44E-04	1.10E-04	0.001185	3.83E-04	1.13E-04	0.00144	0.001256	7.14E-04	6.40E-05	2.49E-04	0.001584
0	5.00E-05	3.30E-05	0	7.20E-05	2.20E-05	0	1.27E-04	0.006024	2.99E-04	0	1.30E-05	7.70E-05	1.36E-04
0	0	0	3.70E-05	5.80E-05	0	2.48E-04	5.60E-05	2.63E-04	7.70E-05	0	7.30E-05	2.40E-05	0
0.004255	0.007984	1.07E-04	1.25E-04	0.002217	0.00397	6.45E-04	0	0.02231	0.001176	0.001303	0.013406	0.002229	7.93E-04
7.30E-04	0	0	0	0	0	0	9.72E-04	5.80E-05	0	1.53E-04	0	0	2.22E-04
3.67E-04	0.00236	3.50E-05	3.18E-04	0	0	0	0	2.60E-05	0.016199	4.98E-04	0.053655	0.040722	0
0	0	0	0	0	0	0	0	1.06E-04	0	0	3.18E-04	5.09E-04	0
0	1.36E-04	0	0.001144	5.58E-04	0	1.32E-04	6.70E-05	0.0033	0.001657	0	1.70E-05	0	0
0	3.51E-04	2.60E-04	7.31E-04	0.002328	0	8.03E-04	1.29E-04	0.001072	0.002138	5.10E-05	0	2.80E-04	4.57E-04
0	0	0	0	0	0	0	0	2.11E-04	0	0	1.20E-05	0	0
0	0	0	0	0	0	0	0	1.61E-04	0	0	1.50E-05	0	0
1.90E-05	1.80E-04	7.72E-04	8.76E-04	0	0	0	0.004532	0	0.018808	0.002988	0	0.019641	0
0	1.16E-04	0	5.60E-05	4.20E-05	0.002226	2.55E-04	0	6.83E-04	7.50E-05	3.29E-04	0	0.002995	2.70E-05
5.90E-05	0.001045	1.64E-04	1.89E-04	1.93E-04	3.30E-04	0	0	1.23E-04	2.98E-04	5.90E-04	0	0.002804	4.30E-05
0	0	2.21E-04	0.001064	3.65E-04	0	6.31E-04	0	0	7.53E-04	1.13E-04	0	0	1.10E-04
0	2.60E-05	1.02E-04	0	2.70E-05	0	0	0	2.24E-04	0	0	6.10E-05	0	0
7.78E-04	2.22E-04	0.001602	0.001581	3.22E-04	4.95E-04	0.005745	0.010063	1.80E-05	0.010698	0.005516	4.00E-05	0	0.009047

0	0	0	0	1.68E-04	0	0	0	2.60E-05	8.20E-05	0.008771	1.90E-05	2.10E-05	0
0	0	0	0	0	0	0	0	2.50E-05	0	0	1.80E-04	8.60E-05	0
2.04E-04	0	3.60E-05	3.70E-05	0	9.70E-05	0	0	4.20E-05	0	0	0.026317	5.33E-04	0
0	0	0	0	0	0	0	0	0	0	0	2.83E-04	1.60E-04	0
0	0	0	0	0	0	0	0	0.001187	0	0	4.28E-04	5.10E-05	0
0	0	0	0	0	0	0	0	0	0	0	0	0	0
0	0	0	0	0	0	0	0	1.00E-04	0	0	7.10E-05	0	0
0.001423	9.90E-05	0.00334	0.00437	5.25E-04	1.70E-05	0.008196	0.016177	0	0.003484	0.001863	0	0	0.003909
0	0	0	0	0	0	0	0	0	0	0	0	0	0
0	0	0	0	0	0	0	0	0	0	0	0	0	0
0	0	0	0	0	0	0	0	0	0	0	0	0	0
0	0	0	3.10E-05	0	0	0	0	6.00E-05	0	0	8.00E-05	0	0
0	0	4.00E-05	7.10E-05	0	0	1.23E-04	0	1.63E-04	0	0	5.10E-05	0	0
0	0	0	0	0	0	0	0	1.08E-04	0	0	4.10E-05	0	0
0	0	0	0	0	0	0	0	2.57E-04	0	0	9.50E-05	2.25E-04	0
5.10E-05	0	0	4.90E-05	0	0	0	0	0	0	0	0	4.09E-04	0
0	0	0	0	0	0	0	0	0	0	0	0	0	0
0	0	0	0	0	0	0	0	0	0	0	0	0	0
0	0	0	0	0	1.70E-05	0	0	2.70E-05	0	0	1.65E-04	8.30E-05	0
0	0	0	0	0	0	0	0	3.70E-05	0	0	1.70E-05	1.11E-04	0
0	0	0	0	0	2.00E-05	0	0	0	0	5.28E-04	0	0	0
0.004889	0.002954	0.003044	0.003517	0.013709	0.006911	0.00425	0.006807	0.014816	0.004525	0.004435	0.02833	0.015656	0.005053

CC36	CC28	CC44	CC71	CC52	CC72	CC34	CC27	CC67	CC12	CC57	CC45	CC24	CC60
0.137361	0.118725	0.094812	0.088889	0.079471	0.077624	0.07125	0.05704	0.040297	0.037202	0.03199	0.027911	0.026878	0.024765
0.031125	0.011597	0.206898	0.062176	0.026166	0.25178	0.152434	0.04186	0.095115	1.21E-04	0.036336	0.058974	0.409736	0.033424
0.225086	0.371889	0.198022	0.045098	0.173058	0.047573	0.134137	0.467579	0.656885	0.714943	0.296828	0.442777	0.036541	0.024965
0.069116	0.02011	0.076384	0.047184	0.046769	0.029091	0.021383	0.037124	0.032222	3.98E-04	0.048098	0.055463	0.146524	0.026422
0.026542	0.056396	0.192726	0.236417	0.092259	0.138139	0.05216	0.093491	0.03767	0.139306	0.054456	0.03915	0.11358	0.194866
0.059169	0.004896	0.019627	0.231498	0.016904	0.128023	0.0308	0.04033	0.002863	1.70E-05	0.041997	0.007525	0.051586	0.560584
0.004731	0	9.03E-04	8.11E-04	0.000649	0.009677	0.010911	0.001489	0.001018	4.70E-05	0.003977	0.002829	0.004726	6.65E-04
0.076047	0.014198	0.017707	0.002748	0.004948	0.012149	0.091319	0.036842	0.003367	1.18E-04	0.011703	0.058577	0.002609	0.024757
0.077918	0.00313	0.024913	0.136337	0.045198	0.131951	0.004399	0.008785	0.005083	0.062756	0.020891	0.030785	0.001924	6.17E-04
0.048895	0.006546	0.081953	0.004183	0.170728	0.013894	0.032903	0.012075	0.04061	0.003437	0.049452	0.011474	0.067678	0.003916
0.029631	0.249534	0.007655	0.032388	0.022829	0.020149	0.014476	0.019385	0.022809	0.022878	0.0437	0.023724	0.006825	9.28E-04
0.026384	0.013218	0.004874	0.003399	0.021422	0.013313	0.071893	0.004732	8.99E-04	2.10E-05	0.011907	0.042399	0.002569	0.026226
0.053076	0.058406	0.018656	0.028891	0.057782	0.013262	0.029859	0.043489	0.00996	0	0.044879	0.093253	0.001215	0.003215
0.006802	0.003953	0.008578	0.008014	7.34E-04	0.014786	0.06043	0.026546	0.002019	9.20E-05	0.002083	0.004281	0.006127	0.001191
7.84E-04	0.001975	0.006632	0.020722	0.008847	0.009724	0.010323	0.004111	0.002006	4.10E-05	0.002991	0.002584	0.011615	0.005798
0.003205	3.35E-04	0.001163	0.011081	0.007217	0.014767	6.50E-04	0.004617	5.67E-04	0	0.004745	0.002362	0.020699	7.23E-04
0.017247	0.008153	0.003609	0.011716	0.001624	0.006512	0.015139	0.025918	0.00374	0	0.004219	0.007711	0.01019	4.01E-04
0.043249	6.00E-05	0	1.10E-05	1.54E-04	0.008294	9.60E-05	3.22E-04	3.18E-04	0	0	3.13E-04	1.70E-05	0
1.68E-04	5.13E-04	0	0	2.01E-04	1.29E-04	0.007751	0.002094	1.97E-04	0	1.05E-04	0.00171	7.56E-04	1.17E-04
0.00401	0.001268	5.27E-04	0.001111	0.017963	0.001279	4.27E-04	0.003292	0.00109	0.01236	0.050373	0.004642	8.00E-05	4.87E-04
7.72E-04	0	0	0	0.002275	0	3.90E-05	2.20E-05	0	1.80E-05	0.003195	1.25E-04	2.10E-05	0
0.001113	0.00494	0.001468	0.002046	0.006394	0.003106	0.024185	0.004054	6.78E-04	0	0.003718	0.0043	0.005043	0.00518
8.40E-05	0	0	0	0.002454	0	0	0	0	2.07E-04	0	0	0	0
1.50E-05	0	0	0.001253	5.45E-04	8.84E-04	8.38E-04	7.50E-04	7.50E-05	0	0.003667	7.80E-04	0.009255	5.10E-05
4.57E-04	0.013387	0.001189	8.55E-04	0.003126	2.77E-04	0.0152	0.002332	2.66E-04	0	0.001867	0.001418	0.001448	0.012072
1.36E-04	5.03E-04	0.002	1.48E-04	0.003266	8.37E-04	0.002529	0.007205	0.006251	0	0.003121	0.003388	0.003152	0.002915
0.001064	0.007998	0.002374	0.0043	6.00E-05	0.005003	0.036364	0.001314	1.79E-04	6.20E-05	0.003551	0.003413	0.00593	0.004525
0	0	0	0	0	0	0	0	0	0	0	4.90E-05	0	0
0.001384	3.17E-04	5.04E-04	0.005636	1.42E-04	0.005311	0.005125	0.002528	5.87E-04	2.00E-05	0.001269	0.00157	0.027478	2.40E-04
0.001439	0.001157	3.12E-04	2.26E-04	0.001272	3.03E-04	0.010905	0.005256	0.002996	0	9.03E-04	0.002768	0.004033	9.50E-04
0.002849	1.09E-04	0	1.00E-05	5.37E-04	0	1.44E-04	0.004578	2.89E-04	0	0.00313	0.00331	1.84E-04	0
2.33E-04	0	1.92E-04	0	0.001002	0	0	0	0	0	2.71E-04	0	0	0
0	0	0	1.50E-05	2.60E-04	6.87E-04	0.011594	9.27E-04	0.001947	0	0	0.002536	0	0.001501
6.87E-04	4.56E-04	1.26E-04	1.17E-04	0.001297	3.72E-04	1.80E-05	9.64E-04	7.82E-04	0	0.062777	1.31E-04	6.22E-04	5.70E-05
0.023502	0.001221	0.001993	3.10E-05	0.044685	0.004864	1.80E-05	1.80E-05	0	2.53E-04	0.054377	0	1.78E-04	0
1.60E-05	0	0	0	7.60E-05	0	0	0	0	0	2.70E-05	0	0	0
6.21E-04	5.70E-05	0.002213	8.10E-05	0.001962	4.89E-04	0.020104	0.002131	4.95E-04	4.10E-05	0.002329	0.004122	5.19E-04	0.014031
4.55E-04	0.00105	0.002559	0.002425	5.15E-04	0.003356	0.008411	0.001366	3.07E-04	0	0.001309	0.002632	0.003336	0.002352
3.94E-04	0	4.99E-04	3.21E-04	3.65E-04	0.003027	0.005595	0.002097	0.001489	0	0.00156	0.001025	0.00399	2.32E-04
0.001745	3.50E-04	5.00E-05	1.54E-04	0.013134	0.001896	8.68E-04	0.002489	4.20E-05	0	7.63E-04	5.98E-04	6.67E-04	3.30E-04
5.70E-05	0	6.10E-05	0	0.001967	7.90E-05	1.07E-04	0	0	0	2.86E-04	0	0	0
6.35E-04	4.83E-04	0.00141	2.70E-05	0.004121	1.54E-04	2.20E-05	8.68E-04	1.84E-04	0	0.021103	0.00318	9.40E-05	0
0	0	0	0	0.002932	0.003264	0.00404	0.001373	8.16E-04	0	0.002287	0.001038	1.06E-04	0
7.08E-04	3.22E-04	0	0.002751	0.038298	0.006348	1.90E-05	9.48E-04	2.34E-04	0	6.80E-04	0.007164	0	0
0	0	0	0	3.79E-04	0	0	0	0	0	9.50E-05	0	0	5.50E-05
5.96E-04	7.00E-05	1.80E-04	1.50E-05	7.10E-05	1.22E-04	0.004011	1.09E-04	0	2.20E-05	0.004328	5.61E-04	2.60E-05	2.17E-04
7.07E-04	2.34E-04	2.33E-04	8.43E-04	0.017916	3.19E-04	0.016844	0.001698	7.20E-05	0	0.002185	0.001433	6.66E-04	0.005325
8.20E-05	0	0	0	1.80E-05	0	0	0	0	0	0	0	0	0
1.90E-05	0	0	0	5.00E-05	0	0	0	0	0	0	0	0	0
1.51E-04	0.001369	0	0	2.50E-05	0.002699	3.07E-04	0.004838	0.004151	0	0.002599	0.015144	0	0
2.61E-04	7.32E-04	6.20E-05	0	0.001958	3.60E-05	0	0	3.20E-05	1.40E-04	5.54E-04	0	1.34E-04	0
0.002784	7.00E-05	5.70E-05	0.00271	3.12E-04	0.001297	5.94E-04	0.001163	4.70E-05	0	0.001784	0.00319	3.38E-04	0
0.001277	0.001249	0	0	1.15E-04	5.78E-04	4.11E-04	8.62E-04	0	0	0.002171	0.001042	4.60E-05	0.003024
3.33E-04	2.63E-04	0	3.33E-04	0.0028	8.70E-05	1.25E-04	8.43E-04	2.89E-04	1.20E-05	0.006742	0	2.66E-04	2.24E-04
1.25E-04	0.005931	0.007837	4.60E-05	0	1.99E-04	9.33E-04	4.50E-05	0.011422	0	6.21E-04	0.002297	0.00175	0.001895

1.18E-04	6.20E-05	3.97E-04	6.70E-05	0.006878	2.88E-04	2.86E-04	0.007318	4.98E-04	0	0.005283	0.002056	0.001188	6.20E-05
1.30E-05	0	0	0	1.72E-04	0	0	0	0	2.30E-05	1.50E-04	0	0	0
2.69E-04	5.90E-05	5.10E-05	0	2.43E-04	5.30E-05	0	0	1.43E-04	0	0.00307	3.70E-05	1.80E-04	0
9.00E-06	0	0	0	5.24E-04	0	0	0	0	0	0	0	0	0
3.96E-04	0	1.24E-04	0	9.37E-04	0	0	0	0	1.19E-04	2.72E-04	0	0	0
0	1.11E-04	0	0	0	0	0	0	0	0	0	0	0	0
3.60E-05	0	0	0	1.19E-04	0	0	0	0	0	8.10E-05	0	0	0
9.80E-05	0.005788	0.00225	8.10E-05	0	3.10E-05	0.001279	2.20E-05	0.004779	2.82E-04	3.20E-05	0.001727	2.58E-04	7.89E-04
0	0	0	0	5.30E-05	0	0	0	0	0	8.90E-05	0	0	0
0	0	0	0	1.90E-05	0	0	0	0	0	0	0	0	0
0	0	0	0	0	0	0	0	0	0	0	0	0	0
0	0	0	0	8.10E-05	0	0	4.10E-05	0	0	0	0	0	4.81E-04
0	0	0	1.70E-05	3.11E-04	0	6.50E-05	0	3.10E-05	7.22E-04	1.51E-04	0	0	0
1.80E-05	0	0	0	2.30E-05	0	0	0	0	0	2.01E-04	0	0	0
1.63E-04	0	0	0	1.96E-04	0	0	0	0	0	9.80E-05	0	0	0
0	0	0	0	0	0	4.40E-05	3.70E-05	0	0	0	1.26E-04	0	0
0	0	0	0	0	0	0	0	0	0	0	0	0	0
0	0	0	0	0	0	0	0	0	0	0	0	0	0
2.00E-05	0	0	0	6.20E-05	2.80E-05	0	0	0	0	0	0	0	0
0	0	0	1.00E-05	6.30E-05	0	0	0	0	6.00E-05	2.10E-05	0	0	0
0	0	0	0	0	0	0	0	0	0	0	0	0	0
0.013613	0.00681	0.00622	0.002808	0.041067	0.01189	0.016236	0.010683	0.002184	0.004282	0.036553	0.010396	0.007217	0.009425

CC29	CC01	CC09	CC42	CC33	CC26	CC46	CC39	CC31	CC55	CC08	CC35	CC64	CC54
0.021805	0.009241	0.006702	0.005383	0.004746	0.004002	0.003966	0.003946	0.002735	0.002173	0.00174	0.0016	0.001595	0.001586
0.238261	0.236629	0.124763	0.02198	0.290797	0.009904	0.037424	0.027102	0.123239	0.023711	0.202327	0.876104	0.001923	0.178422
0.171381	0.003409	0.023542	0.111873	0.10655	0.673501	0.435454	0.585202	0.034425	0.040498	5.49E-04	0.005625	0.972593	0.03028
0.052025	0.184919	0.072217	0.397193	0.032255	0.081262	0.015399	0.023755	0.005722	0.289151	0.216593	0.008017	0.002358	0.162485
0.100637	0.525203	0.40795	0.007032	0.491161	0.054434	0.010792	0.052843	0.713589	2.08E-04	0.315524	0.001081	7.83E-04	0.175315
0.004365	8.76E-04	0.090906	3.33E-04	6.03E-04	0.003455	0.004938	0.008481	0.064431	5.05E-04	1.00E-04	4.45E-04	6.14E-04	0.001836
0.004544	0	8.25E-04	5.74E-04	7.44E-04	1.97E-04	0.001451	2.00E-04	6.07E-04	1.92E-04	0	9.99E-04	4.06E-04	0.002055
0.001515	7.35E-04	0.003447	0.384855	8.42E-04	1.73E-04	0.025073	7.38E-04	0.001849	0.091235	6.90E-05	8.90E-05	8.24E-04	7.45E-04
0.001014	8.74E-04	0.001747	0.0242	0.001577	0.018835	0.019492	0.013506	2.27E-04	1.39E-04	3.10E-05	6.31E-04	0.002075	1.87E-04
0.24982	0.012565	0.013552	1.36E-04	0.018416	0.00632	0.004357	0.006123	0.008155	0.248872	0.241367	0.091367	0.001075	0.256261
0.015031	0.002609	1.36E-04	0.021341	0.009023	0.001748	0.010233	0.024542	0.007338	0.171438	4.94E-04	0.001064	2.72E-04	0.008112
0.001003	1.42E-04	0.029724	7.00E-05	1.39E-04	1.15E-04	0.028639	0.005801	5.80E-05	4.00E-05	0	3.36E-04	1.19E-04	0.002197
0.034695	0	0.005601	1.39E-04	0.005968	0.0555	0.063504	0.2105	0.001778	0	1.48E-04	1.77E-04	0.004867	0.004571
8.94E-04	1.60E-04	0.014437	6.10E-05	7.68E-04	2.45E-04	0.043127	0.001691	7.24E-04	0	4.71E-04	3.47E-04	1.20E-04	0.007856
0.014924	0.002649	0.125127	9.16E-04	0.004111	0.002577	0.001256	0.003718	0.002947	4.50E-05	0.01098	8.90E-05	2.38E-04	0.029551
0.001411	0.001638	0.001898	0.001364	0.003121	0.001003	0.014852	0.005475	0.001016	0.011411	1.44E-04	5.66E-04	5.50E-05	0.009626
0.004798	8.80E-04	0.005165	8.19E-04	5.55E-04	0.008881	0.009177	1.80E-05	7.84E-04	0.079194	6.16E-04	0	2.65E-04	8.26E-04
2.10E-05	0	3.30E-05	0	0	0	1.09E-04	0.00105	0	0	0	0	2.10E-05	0
0	0	2.70E-05	0	1.24E-04	7.70E-05	0	0	5.90E-05	0	0	0	3.20E-05	0
8.20E-05	7.45E-04	1.21E-04	2.25E-04	2.04E-04	3.00E-05	0.155432	0	0	0.013539	0	8.90E-05	5.20E-05	1.04E-04
5.90E-05	0	0	0	0	0	5.02E-04	5.40E-05	0	0	0	9.60E-05	0	0
0.006131	0.001645	0.007607	0	3.32E-04	0.001127	0.004939	0.002458	0.001397	1.70E-05	2.12E-04	8.50E-05	8.40E-05	0.012698
0	0	0	0	0	0	1.07E-04	0	0	0	2.70E-05	0	0	0
2.74E-04	0.003857	0	2.54E-04	4.97E-04	1.77E-04	0.001406	0	3.20E-04	0.003682	0.002686	0.003521	1.10E-05	0.009561
8.45E-04	0.001647	0.024953	0	8.19E-04	0.002041	7.12E-04	6.59E-04	9.26E-04	0	0.003028	0	1.14E-04	0.012211
0.027752	0.004926	5.93E-04	0.004563	0.010919	1.16E-04	0.00998	0.001923	0.005227	0.001011	2.74E-04	7.14E-04	6.20E-05	0.012241
0.002291	1.51E-04	0.006689	0.001354	0	3.30E-05	0.00789	0.001547	0.001378	4.90E-05	2.70E-05	0	2.90E-05	7.15E-04
0	0	0	0	0	0	0	0	0	0	0	0	0	0
0.003622	0	0.003152	0.002605	2.01E-04	5.84E-04	0.001235	7.73E-04	6.59E-04	4.48E-04	8.77E-04	9.30E-05	1.78E-04	0.002016
0.008833	0	0	0	0.002552	1.03E-04	8.28E-04	3.33E-04	0.002335	2.40E-05	3.70E-05	0	2.11E-04	0.048268
1.70E-05	0	0	0	7.10E-05	1.43E-04	0	6.70E-05	0	0	0	0	4.30E-05	0
7.00E-05	0	0	0	0	0	1.01E-04	5.90E-05	0	0	0	0	0	0
0.002057	0	0.001567	0	0.001932	0	0	0	6.42E-04	0	0	0	0	0.018166
4.10E-05	0	0	6.11E-04	1.39E-04	4.20E-05	0	2.15E-04	0	0.007976	0	0	4.00E-05	4.60E-05
0.002451	1.53E-04	0.002982	5.71E-04	2.02E-04	4.35E-04	0	1.74E-04	2.71E-04	0.00739	2.40E-05	0	2.60E-05	9.60E-05
0	0	0	0	0	0	0	0	0	0	0	0	0	0
0.001448	7.46E-04	2.90E-05	0	2.51E-04	0	3.05E-04	3.80E-04	0.009106	0	0	0	7.90E-05	5.40E-05
0.011072	0.001076	0.006943	0	4.57E-04	5.06E-04	3.23E-04	2.30E-05	0.001686	0	1.22E-04	0.00107	1.63E-04	6.09E-04
0.001872	7.49E-04	0.001967	1.58E-04	4.44E-04	8.20E-05	0.00115	6.70E-05	0.001774	3.00E-05	0.001273	0.005286	2.50E-05	0.002336
1.88E-04	0	5.18E-04	0	6.40E-05	7.64E-04	0.014076	0.001736	2.50E-04	0	0	0	2.50E-05	5.00E-05
1.80E-05	0	0	0	0	3.28E-04	4.23E-04	4.03E-04	1.28E-04	0	0	0	0	0
8.50E-05	0	0	1.95E-04	6.40E-05	7.30E-05	0.031284	3.38E-04	5.50E-05	1.84E-04	0	0	0	0
0	0	0	0	6.20E-05	4.90E-05	0.00101	4.53E-04	0	0	0	0	0	0
2.62E-04	0	0	0	6.30E-05	0	7.35E-04	0	0	0.00118	0	0	2.10E-04	0
6.50E-05	0	2.90E-05	0	0	0	1.00E-04	1.21E-04	0	1.03E-04	2.60E-05	0	0	0
5.30E-05	0	0	0	0	0	8.23E-04	1.13E-04	0	2.90E-05	0	0	3.00E-05	4.50E-05
0	0	0.004516	8.30E-05	0	0	0	2.45E-04	0	0	0	0	0	4.50E-05
0	0	0	0	0	0	0	0	0	0	0	0	0	0
0	0	0	0	0	0	0	0	0	0	0	0	0	0
0	0	0	0	3.80E-04	0	0	0	3.18E-04	2.20E-05	0	0	0.004913	0
0.002869	0	3.50E-05	0	0	0.044582	0	6.61E-04	0	2.00E-05	0	0	0	1.02E-04
7.20E-05	1.49E-04	3.47E-04	0	6.00E-05	3.30E-05	0.00124	3.30E-04	0	0	0	0	8.00E-06	0
0	0	0	0	0	3.90E-05	0	5.60E-05	2.83E-04	0	0	0	5.30E-05	1.09E-04
7.00E-05	1.32E-04	2.80E-05	0	1.90E-04	0	0.01106	8.24E-04	3.66E-04	0	0	0	0	3.93E-04
0.002052	6.07E-04	2.93E-04	0.009811	0.004402	0.020939	0	0	7.06E-04	0.002342	3.10E-05	7.50E-05	0.002584	0.002869

9.00E-05	0	0	0	1.24E-04	0	0.004369	0.001016	1.76E-04	5.40E-05	0	0	1.81E-04	2.10E-04
0	0	0	0	0	0	4.87E-04	0	0	2.30E-05	0	0	0	0
0	0	0	0	0	4.40E-05	6.08E-04	1.30E-04	0	3.10E-05	0	9.20E-05	9.00E-06	0
0	0	0	0	0	0	0	0	0	0	0	0	0	0
6.40E-05	0	0	0	0	0	1.14E-04	0	0	0	0	0	0	0
0	0	0	0	0	0	0	0	0	0	0	0	0	0
2.20E-05	0	0	0	0	0	0	0	0	0	0	0	0	0
2.72E-04	1.55E-04	0	1.20E-04	5.52E-04	0.001367	0	0	1.82E-04	0	0	0	3.09E-04	0
0	0	0	0	0	0	0	2.20E-05	0	0	0	0	0	1.63E-04
0	0	0	0	0	0	0	0	0	0	0	0	0	0
0	0	0	0	0	0	0	0	0	0	0	0	0	0
0	0	0	0	0	0	0	0	0	0	0	0	0	0
4.80E-05	0	0	6.32E-04	0	1.23E-04	0	2.10E-05	0	0	6.70E-05	0	4.10E-05	0
0	0	0	0	0	0	0	0	0	0	0	0	0	0
6.70E-05	0	0	0	0	0	1.00E-04	0	0	0	0	0	0	0
0	0	0	0	0	0	0	0	0	0	0	0	0	0
0	0	0	0	0	0	0	0	0	0	0	0	0	0
0	0	0	0	0	0	0	0	0	0	0	0	0	0
3.70E-05	0	0	0	0	0	0	0	0	5.00E-05	0	0	0	0
1.70E-05	0	0	0	0	0	1.06E-04	0	0	0	0	0	0	0
0	0	0	0	0	0	0	0	0	0	0	0	0	0
0.006588	7.33E-04	0.009832	5.49E-04	0.003519	0.004011	0.019312	0.010108	0.002132	0.002984	1.36E-04	3.42E-04	2.85E-04	0.004982

CC17	CC20	CC48	CC02	CC05	CC06
0.001421	9.82E-04	7.42E-04	5.11E-04	1.21E-04	1.11E-04
0.240161	0.069864	0.149546	0.268974	0.597858	0.333565
0.019035	0.040896	0.005209	0.031166	0.021588	0.08799
0.1539	0.004336	0.309515	0.180312	0.171448	0.289009
0.232437	0.065683	3.09E-04	0.055463	0.026697	0.077827
0.005599	0.003598	1.30E-04	0.030431	1.82E-04	1.90E-05
7.96E-04	6.03E-04	6.50E-05	0.015444	0.005774	0.002611
7.24E-04	0.001236	0.30678	7.50E-05	0.003117	3.00E-05
0.002857	1.21E-04	2.02E-04	7.50E-05	7.69E-04	1.60E-05
0.035588	0.60363	0.020239	0.018512	0.001063	0.003104
6.15E-04	0.037248	0.012633	0.187626	0.002132	0.020399
3.77E-04	0.002475	8.70E-05	0.001227	0.009256	7.20E-04
0.003039	2.30E-05	4.30E-05	2.40E-05	3.99E-04	0.002712
0.003575	0.018921	0	0	0.073581	0.044648
0.011601	0.002558	1.32E-04	0.072212	0.028098	0.01572
0.014193	5.96E-04	0.080258	0.001183	0.003743	5.08E-04
6.99E-04	0.003224	1.12E-04	0.051445	2.71E-04	0.027254
0	0	1.90E-05	0	0	0
0	0	2.50E-05	0	0.006781	1.00E-05
0.003866	1.80E-05	0	3.80E-04	0	0
0.034319	1.55E-04	1.21E-04	1.27E-04	0	1.80E-05
0.01207	0.002176	2.30E-05	0.031987	2.08E-04	0.030473
0	3.15E-04	1.43E-04	0	0	2.30E-05
0.021633	0.001739	0.001928	0.008913	0.006791	0.006812
0.005026	0.008902	2.00E-05	1.20E-04	3.24E-04	0.037143
0.025664	4.64E-04	0.001873	0.002892	0	3.54E-04
0.006854	0.050899	4.70E-05	0.007481	0.003254	0.002055
0	0	0	0	0	0
0.005313	0.015693	0	0.003217	0.001132	0.001461
0.013009	2.00E-05	5.90E-05	9.20E-05	0	4.50E-04
0	0	0	0	0	0
0.014121	2.42E-04	3.70E-05	1.24E-04	0	0
0.003937	1.90E-05	0	2.00E-05	0	0
2.80E-05	0.003102	0	0	0	0
0.01011	0.0198	0.029702	0.004329	5.90E-05	0
2.43E-04	0	0	2.00E-05	0	0
2.20E-05	0.004909	6.50E-05	0.001162	0.009603	0
0.005307	0.010149	2.60E-05	0.005678	0.013295	9.50E-05
0.011484	0.009727	2.50E-05	0.010496	1.25E-04	9.71E-04
0.002631	1.49E-04	0	0	0	3.70E-05
0.003793	7.70E-05	0	0	0	3.70E-05
1.39E-04	0	0.008599	0	0	1.47E-04
4.04E-04	7.10E-05	2.40E-05	0	0	9.60E-05
0	4.10E-05	2.20E-05	5.85E-04	0	0
1.08E-04	1.58E-04	1.86E-04	2.10E-05	0	2.30E-05
1.80E-05	8.14E-04	0	9.00E-05	0	0
6.70E-05	1.72E-04	0	0	0	4.00E-05
0.002065	7.40E-05	0	0	0	0
9.87E-04	1.70E-05	0	0	0	0
0	0	0	0	0	0
4.10E-05	4.30E-05	7.60E-05	0	0	1.40E-05
2.00E-05	0	0	5.15E-04	2.90E-05	1.90E-05
0	1.70E-04	0	0	0	0
0.004219	3.50E-05	0	2.30E-05	0	4.87E-04
6.10E-05	6.20E-05	0	2.11E-04	3.07E-04	8.45E-04

3.72E-04	4.35E-04	5.90E-05	0	0	0
1.40E-05	0	1.03E-04	0	0	0
1.90E-05	0	0.063661	0	0	0
2.50E-05	1.70E-05	0	0	0	0
0.008508	1.53E-04	0	0	0	0
0	0	0	0	0	0
0.001671	0	0	3.50E-05	0	0
0	0	0	0	0	0
0	0	0	0	0	0
2.41E-04	0	0	0	0	0
1.90E-05	0	0	0	0	0
7.61E-04	0	0	0	0	0
9.68E-04	0	0	0	0	1.80E-05
0.001098	5.30E-05	0	1.60E-05	0	0
0.003853	2.10E-05	0	9.90E-05	0	0
0	0	0	0	0	0
2.30E-05	0	0	0	0	0
0	0	0	0	0	0
4.50E-05	1.63E-04	1.90E-05	2.50E-05	0	0
0	0	0	0	0	0
0	0	2.10E-05	0	0	0
0.068207	0.012952	0.007115	0.006662	0.011995	0.012129

Table S4. The different degree of phylum level (p value) between the healthy controls (n=94) and confirmed cases (n=48)												
ID	H.median	H.mean	H.se	CC.median	CC.mean	CC.se	p-value	z-score	Sig_mark	q-value	fixp	fixps
Firmicutes	0.2202(0.1702,0.2695)	0.225054457	0.0079561	0.3618(0.1836,0.5026)	0.37226383	0.03266346	1.33E-04	-3.8209578	***	2.99E-04	0.0001	***0.0001
Bacteroidota	0.3198(0.2633,0.3926)	0.323659096	0.00926198	0.115(0.0596,0.2405)	0.17182904	0.0247922	5.53E-12	-6.8912522	***	9.95E-11	<0.0001	***<0.0001
Proteobacteria	0.2517(0.142,0.3485)	0.25831167	0.01546552	0.0925(0.0238,0.2772)	0.18377202	0.03101321	4.33E-04	-3.5191707	***	8.66E-04	0.0004	***0.0004
Patescibacteria	0.0235(0.0128,0.0348)	0.025461904	0.00177392	0.0037(0.0005,0.0101)	0.01090604	0.00279398	5.37E-10	-6.2079782	***	4.83E-09	<0.0001	***<0.0001
Campilobacterota	0.0079(0.005,0.0127)	0.010297479	8.13E-04	0.0013(0.0006,0.0053)	0.00625492	0.00196262	1.38E-08	-5.6753789	***	6.23E-08	<0.0001	***<0.0001
Spirochaetota	0.0014(0.0005,0.0035)	0.002684872	4.76E-04	<0.0001(<0.0001,0.0006)	0.00106717	4.10E-04	1.18E-08	-5.7031871	***	6.23E-08	<0.0001	***<0.0001
Synergistota	0.0001(<0.0001,0.0004)	4.32E-04	9.18E-05	<0.0001(<0.0001,<0.0001)	1.67E-04	8.55E-05	3.13E-07	-5.115618	***	1.13E-06	<0.0001	***<0.0001
Desulfobacterota	<0.0001(<0.0001,0.0002)	4.48E-04	1.21E-04	<0.0001(<0.0001,<0.0001)	2.25E-05	1.01E-05	8.45E-07	-4.9245726	***	2.17E-06	<0.0001	***<0.0001
Deferribacterota	<0.0001(<0.0001,<0.0001)	4.09E-04	2.95E-04	<0.0001(<0.0001,<0.0001)	0	0	0.0387582	-2.0667449	*	0.063423	0.0388	* 0.0388
Verrucomicrobiota	<0.0001(<0.0001,<0.0001)	7.44E-05	2.52E-05	<0.0001(<0.0001,<0.0001)	8.54E-06	5.30E-06	0.0013954	-3.1955986	**	0.002512	0.0014	** 0.0014
Bacteria_unclassified	<0.0001(<0.0001,<0.0001)	8.40E-07	4.80E-07	<0.0001(<0.0001,<0.0001)	5.23E-05	2.09E-05	5.84E-07	-4.9963045	***	1.75E-06	<0.0001	***<0.0001

Table S4. The different degree of genus level (p value) between the healthy controls (n=94) and confirmed cases (n=48)

ID	H.median	H.mean	H.se	CC.median	CC.mean	CC.se	p-value	z-score	Sig_mark	q-value	fixp	fixps
Neisseria	0.1421(0.0644,0.2686)	0.169761053	0.013085052	0.0346(0.0036,0.1682)	0.138294521	0.029863	3.12E-04	-3.604871806	***	8.36E-04	0.0003	***0.0003
Prevotella	0.165(0.1041,0.2111)	0.168590851	0.009107967	0.0549(0.0234,0.1844)	0.124493771	0.024038	3.80E-05	-4.119212797	***	1.32E-04	<0.0001	***<0.0001
Streptococcus	0.0522(0.0339,0.0851)	0.065783394	0.004755614	0.0973(0.0297,0.3142)	0.195030833	0.033191	0.048742	-1.970845492	*	0.07469	0.0487	* 0.0487
Leptotrichia	0.0257(0.0158,0.0496)	0.039801809	0.004334475	0.056(0.0327,0.1554)	0.122804625	0.021829	5.75E-05	-4.022986324	***	1.82E-04	<0.0001	***<0.0001
Fusobacterium	0.059(0.0374,0.0934)	0.06736984	0.004523445	0.0076(0.0016,0.0407)	0.039066146	0.012837	1.88E-09	-6.007999788	***	2.06E-08	<0.0001	***<0.0001
Alloprevotella	0.0611(0.0369,0.0978)	0.068396457	0.004422687	0.0008(0.0005,0.0017)	0.002005479	4.39E-04	6.36E-22	-9.623582422	***	1.96E-19	<0.0001	***<0.0001
Porphyromonas	0.0458(0.0206,0.0725)	0.050024957	0.003820588	0.0034(0.0009,0.0207)	0.02960225	0.010328	1.94E-09	-6.003004069	***	2.06E-08	<0.0001	***<0.0001
Haemophilus	0.0409(0.0224,0.0682)	0.051747766	0.004688509	0.0032(0.0007,0.019)	0.017106125	0.004506	2.32E-12	-7.01349812	***	6.51E-11	<0.0001	***<0.0001
Capnocytophaga	0.011(0.0044,0.0192)	0.017410543	0.002076938	0.0034(0.0006,0.0133)	0.011414083	0.00259	7.49E-04	-3.370806467	***	0.00186	0.0007	***0.0007
TM7x	0.0078(0.0037,0.0145)	0.010800394	0.001098544	0.0017(0.0004,0.0063)	0.00788875	0.00229	4.24E-06	-4.599424234	***	1.95E-05	<0.0001	***<0.0001
Campylobacter	0.0078(0.0049,0.0122)	0.009667415	6.90E-04	0.0013(0.0006,0.0053)	0.006253188	0.001963	2.55E-08	-5.569726047	***	2.07E-07	<0.0001	***<0.0001
Granulicatella	0.0057(0.0038,0.0087)	0.007321681	5.87E-04	0.0037(0.0008,0.0094)	0.008644729	0.002049	0.034036	-2.11964854	*	0.05606	0.034	* 0.034
Actinobacillus	0.0015(0.0002,0.0048)	0.010110149	0.003220446	<0.0001(<0.0001,<0.0001)	0.001499146	9.66E-04	1.50E-11	-6.748168495	***	3.29E-10	<0.0001	***<0.0001
g__Absconditabacteriales_	0.0064(0.0012,0.0109)	0.008242298	9.96E-04	<0.0001(<0.0001,0.0002)	6.22E-04	2.26E-04	8.80E-15	-7.755526394	***	3.87E-13	<0.0001	***<0.0001
Bacteroides	0.0005(0.0001,0.0023)	0.007102128	0.002468277	<0.0001(<0.0001,<0.0001)	9.59E-04	7.17E-04	7.49E-11	-6.510577394	***	1.28E-09	<0.0001	***<0.0001
Oribacterium	0.0041(0.0028,0.0061)	0.004629426	2.79E-04	0.0019(0.0009,0.005)	0.004610875	0.00105	5.51E-04	-3.454419889	***	0.00139	0.0006	***0.0006
Pseudomonas	0.0016(0.0005,0.0053)	0.005926404	0.001207174	<0.0001(<0.0001,<0.0001)	7.91E-05	5.15E-05	1.05E-19	-9.083472302	***	1.62E-17	<0.0001	***<0.0001
Megasphaera	0.0015(0.0004,0.0055)	0.004677713	8.02E-04	0.0004(<0.0001,0.002)	0.002022875	5.58E-04	0.002021	-3.087145668	**	0.00448	0.002	** 0.002
Stomatobaculum	0.0022(0.001,0.005)	0.003876809	5.56E-04	0.0009(0.0003,0.0027)	0.003548313	0.001007	0.003528	-2.917576433	**	0.00734	0.0035	** 0.0035
Moraxella	<0.0001(<0.0001,<0.0001)	0.005172415	0.00268073	<0.0001(<0.0001,<0.0001)	2.25E-06	1.58E-06	4.41E-05	-4.084999519	***	1.49E-04	<0.0001	***<0.0001
Solobacterium	0.003(0.0022,0.0045)	0.003863372	3.29E-04	0.0012(0.0003,0.003)	0.002449563	6.49E-04	1.09E-06	-4.875407272	***	5.95E-06	<0.0001	***<0.0001
Clostridia_UCG-014	0.0029(0.0015,0.005)	0.003760894	3.16E-04	0.0005(<0.0001,0.0024)	0.002628688	0.001051	1.04E-07	-5.320052225	***	7.43E-07	<0.0001	***<0.0001
Aggregatibacter	0.0029(0.0008,0.0069)	0.004317691	4.45E-04	<0.0001(<0.0001,0.0001)	5.93E-04	2.00E-04	5.77E-15	-7.808917594	***	2.96E-13	<0.0001	***<0.0001
Faecalibacterium	0.0002(<0.0001,0.001)	0.004224266	0.001479948	<0.0001(<0.0001,<0.0001)	3.75E-04	2.94E-04	1.83E-09	-6.01180427	***	2.06E-08	<0.0001	***<0.0001
Lachnospiraceae_uncultured	0.0012(0.0002,0.0042)	0.003575043	7.10E-04	<0.0001(<0.0001,0.001)	0.001310417	4.82E-04	4.10E-06	-4.60637879	***	1.91E-05	<0.0001	***<0.0001
Corynebacterium	0.0007(0.0004,0.0016)	0.002868426	8.31E-04	0.0001(<0.0001,0.0006)	0.002483542	0.001388	3.64E-06	-4.630727875	***	1.75E-05	<0.0001	***<0.0001
Halomonas	<0.0001(<0.0001,0.0002)	2.55E-04	6.06E-05	0.0003(<0.0001,0.004)	0.007580667	0.002317	5.73E-05	-4.023690104	***	1.82E-04	<0.0001	***<0.0001
Muribaculaceae	<0.0001(<0.0001,0.0003)	0.004072617	0.001402026	<0.0001(<0.0001,<0.0001)	7.96E-06	5.29E-06	7.35E-10	-6.158422985	***	1.03E-08	<0.0001	***<0.0001
Tannerella	0.0017(0.0008,0.0033)	0.002695223	3.93E-04	0.0004(<0.0001,0.002)	0.002003083	5.73E-04	6.15E-05	-4.007100503	***	1.93E-04	<0.0001	***<0.0001
[Eubacterium]_nodatum_group	0.002(0.0013,0.003)	0.002333032	1.62E-04	0.0012(0.0003,0.0026)	0.002485188	5.10E-04	0.007743	-2.663082232	**	0.01532	0.0077	** 0.0077
Atopobium	0.0014(0.0008,0.003)	0.002436968	3.01E-04	0.0007(0.0002,0.0016)	0.001662667	3.82E-04	0.00156	-3.163323719	**	0.00348	0.0016	** 0.0016
Treponema	0.0013(0.0005,0.0035)	0.002680266	4.76E-04	<0.0001(<0.0001,0.0006)	0.001050563	4.11E-04	5.64E-09	-5.827009789	***	5.27E-08	<0.0001	***<0.0001
Lachnospiraceae_unclassified	0.0001(<0.0001,0.0005)	0.002976691	8.79E-04	<0.0001(<0.0001,<0.0001)	1.79E-04	8.79E-05	5.47E-07	-5.009157126	***	3.24E-06	<0.0001	***<0.0001
Saccharimonadales	0.0012(0.0002,0.0032)	0.00254934	4.17E-04	<0.0001(<0.0001,0.0003)	4.20E-04	1.31E-04	9.09E-10	-6.12458647	***	1.22E-08	<0.0001	***<0.0001
Rhodococcus	<0.0001(<0.0001,0.0006)	0.00209517	8.74E-04	<0.0001(<0.0001,<0.0001)	5.00E-05	1.52E-05	1.52E-05	-4.325846671	***	5.92E-05	<0.0001	***<0.0001
Parvimonas	0.0012(0.0005,0.0024)	0.001871883	2.18E-04	<0.0001(<0.0001,0.0001)	4.03E-04	1.40E-04	6.97E-11	-6.521288901	***	1.26E-09	<0.0001	***<0.0001
Johnsonella	0.0006(<0.0001,0.0017)	0.001378255	2.05E-04	0.0002(<0.0001,0.0007)	0.001296729	5.15E-04	0.003029	-2.964739446	**	0.00643	0.003	** 0.003
Fusicatenibacter	<0.0001(<0.0001,0.0005)	0.001944043	6.81E-04	<0.0001(<0.0001,<0.0001)	5.13E-05	4.31E-05	2.21E-10	-6.345705599	***	3.41E-09	<0.0001	***<0.0001

Lachnospiraceae_NK4A136_group	<0.0001(<0.0001,0.0002)	0.001862128	6.63E-04	<0.0001(<0.0001,<0.0001)	2.60E-05	2.07E-05	9.65E-08	-5.333133427	***	7.08E-07	<0.0001	***<0.0001
Lactobacillales_unclassified	0.0002(<0.0001,0.0007)	9.23E-04	2.35E-04	<0.0001(<0.0001,0.0008)	0.00176525	6.42E-04	0.026445	-2.219611311	*	0.04451	0.0264	* 0.0264
F0332	0.0002(<0.0001,0.0005)	0.001143372	3.56E-04	<0.0001(<0.0001,0.0002)	0.001232417	9.28E-04	4.01E-04	-3.539394506	***	0.00106	0.0004	***0.0004
Bergeyella	0.0011(0.0005,0.0018)	0.001459298	1.55E-04	<0.0001(<0.0001,0.0004)	4.79E-04	1.20E-04	1.37E-09	-6.059206237	***	1.62E-08	<0.0001	***<0.0001
Filifactor	0.0007(0.0002,0.0019)	0.001475734	2.36E-04	<0.0001(<0.0001,0.0003)	3.07E-04	8.76E-05	1.82E-08	-5.627843298	***	1.52E-07	<0.0001	***<0.0001
Dialister	0.0006(0.0002,0.0015)	0.001279957	2.42E-04	<0.0001(<0.0001,0.0003)	6.37E-04	2.80E-04	1.31E-08	-5.68532837	***	1.12E-07	<0.0001	***<0.0001
P5D1-392	<0.0001(<0.0001,0.0002)	1.61E-04	3.06E-05	0.0007(<0.0001,0.0027)	0.002654688	6.14E-04	1.12E-08	-5.711994659	***	1.01E-07	<0.0001	***<0.0001
Saccharimonadaceae	0.0003(<0.0001,0.0006)	0.00102933	3.54E-04	<0.0001(<0.0001,0.0003)	8.47E-04	2.96E-04	0.00239	-3.036953253	**	0.00522	0.0024	** 0.0024
Acinetobacter	0.0001(<0.0001,0.0007)	0.001232989	3.64E-04	<0.0001(<0.0001,<0.0001)	2.66E-05	1.17E-05	6.65E-11	-6.528285878	***	1.26E-09	<0.0001	***<0.0001
Sphingomonas	<0.0001(<0.0001,0.0004)	0.001183681	4.51E-04	<0.0001(<0.0001,<0.0001)	2.12E-05	1.26E-05	2.63E-07	-5.148471702	***	1.72E-06	<0.0001	***<0.0001
Bifidobacterium	<0.0001(<0.0001,0.0003)	7.76E-04	2.59E-04	<0.0001(<0.0001,<0.0001)	2.57E-04	1.79E-04	6.41E-06	-4.512431283	***	2.67E-05	<0.0001	***<0.0001
Leptotrichiaceae_uncultured	<0.0001(<0.0001,<0.0001)	8.48E-04	4.17E-04	<0.0001(<0.0001,<0.0001)	2.31E-06	2.31E-06	0.035812	-2.099058837	*	0.05836	0.0358	* 0.0358
Megamonas	<0.0001(<0.0001,0.0002)	8.13E-04	2.77E-04	<0.0001(<0.0001,<0.0001)	4.45E-05	3.48E-05	2.14E-07	-5.186417065	***	1.44E-06	<0.0001	***<0.0001
Burkholderiales_unclassified	<0.0001(<0.0001,0.0001)	1.17E-04	2.21E-05	0.0001(<0.0001,0.0014)	0.001328667	4.09E-04	0.001292	-3.217859975	**	0.00301	0.0013	** 0.0013
Selenomonadaceae_uncultured	<0.0001(<0.0001,0.0001)	7.16E-04	3.17E-04	<0.0001(<0.0001,<0.0001)	6.81E-06	3.97E-06	4.63E-06	-4.580941483	***	2.10E-05	<0.0001	***<0.0001
[Eubacterium]_ruminantium_group	<0.0001(<0.0001,0.0002)	7.14E-04	2.51E-04	<0.0001(<0.0001,<0.0001)	5.42E-06	5.03E-06	4.35E-09	-5.870423544	***	4.18E-08	<0.0001	***<0.0001
Christensenellaceae_R-7_group	<0.0001(<0.0001,0.0002)	5.87E-04	1.82E-04	<0.0001(<0.0001,<0.0001)	3.20E-05	1.86E-05	4.25E-07	-5.057511269	***	2.67E-06	<0.0001	***<0.0001
Phascolarctobacterium	<0.0001(<0.0001,0.0001)	4.86E-04	2.19E-04	<0.0001(<0.0001,<0.0001)	3.25E-05	2.32E-05	4.97E-06	-4.566172336	***	2.19E-05	<0.0001	***<0.0001
Agathobacter	<0.0001(<0.0001,0.0001)	4.40E-04	1.56E-04	<0.0001(<0.0001,<0.0001)	1.08E-04	8.02E-05	4.30E-04	-3.520849724	***	0.00112	0.0004	***0.0004
Gracilibacteria	<0.0001(<0.0001,0.0002)	4.63E-04	2.52E-04	<0.0001(<0.0001,<0.0001)	1.49E-05	8.95E-06	3.85E-07	-5.076174997	***	2.47E-06	<0.0001	***<0.0001
Prevotellaceae_NK3B31_group	<0.0001(<0.0001,<0.0001)	4.26E-04	2.02E-04	<0.0001(<0.0001,<0.0001)	4.79E-07	4.79E-07	0.049209	-1.966778273	*	0.07503	0.0492	* 0.0492
Mucispirillum	<0.0001(<0.0001,<0.0001)	4.09E-04	2.95E-04	<0.0001(<0.0001,<0.0001)	0	0	0.038758	-2.06674489	*	0.06091	0.0388	* 0.0388
Serratia	<0.0001(<0.0001,0.0002)	3.75E-04	2.29E-04	<0.0001(<0.0001,<0.0001)	1.54E-05	5.25E-06	5.75E-06	-4.535467808	***	2.43E-05	<0.0001	***<0.0001
Acidovorax	<0.0001(<0.0001,<0.0001)	3.02E-04	1.25E-04	<0.0001(<0.0001,<0.0001)	9.21E-06	3.62E-06	3.85E-05	-4.116538283	***	1.32E-04	<0.0001	***<0.0001

Table S5. The relative abundance and distribution of the key 57 OTUs										
	H002	H003	H004	H005	H008	H009	H010	H011	H013	H015
OTU796 (Rothia)	0	0	0	0.000422	0	0	0	0	0.00012	0
OTU813 (Rothia)	0	0	0	4.98E-05	0	0	0	0	0	0
OTU744 (Leptotrichia)	0.000111	0	0	0.001054	5.81E-05	3.28E-05	0	0	0.001946	0.001853
OTU748 (Leptotrichia)	0	0	0	0.00016	0	0	0	0	0	0.000113
OTU762 (Streptococcus)	0.000139	0	0	0.000268	0	0	0.001438	0	0.00157	0.000221
OTU658 (P5D1-392)	0	0	0	0.000115	0	0	0	0	0.00036	0.000554
OTU599 (Burkholderiales_unclassified)	0	0	0	0.000281	0	0	0	0	0.001129	0.000272
OTU734 (Streptococcus)	0	0	0	0	0	0	0	0	0.000109	0.000376
OTU34 (Halomonas)	0.000353	0.001022	0.000122	0.000181	6.84E-05	5.28E-05	0.002608	0.000719	0	4.65E-05
OTU162 (Rhizobiaceae_unclassified)	0	0	0	0	0	0	0	0	0	0
OTU138 (Pelagibacterium)	0	0	0	0	0	0	0	0	0	0
OTU123 (Xanthomonadaceae_unclassified)	0	0	0	0	0	0	0	0	0	0
OTU12 (Gemella)	0.002162	0.02162	0.001775	0.022205	0.006993	0.007181	0.01162	0.003409	0.004386	0.003972
OTU1 (Streptococcus)	0.092913	0.225843	0.028655	0.068001	0.093629	0.052115	0.183993	0.140543	0.079217	0.054509
OTU84 (Streptococcus)	0.009296	0.017379	0.003094	0.003334	0.018318	0.004425	0.019653	0.017191	0.005694	0.006209
OTU104 (Catonella)	0.00456	0.000592	0.002873	0.006251	0.002585	0.008038	0.0014	0.004881	0.002052	0.002604
OTU4 (Prevotella)	0.116394	0.136927	0.073231	0.164448	0.319775	0.041436	0.110746	0.259281	0.197486	0.092929
OTU523 (Prevotella)	0.007474	0.010884	0.005812	0.011922	0.019228	0.002133	0.007801	0.017684	0.011093	0.004641
OTU15 (Campylobacter)	0.016865	0.004834	0.004931	0.002762	0.040767	0.010113	0.010476	0.014345	0.004094	0.011993
OTU134 (Prevotella)	0.005874	0.003201	0.00304	0.002917	0.003994	0.006134	0.002238	0.005983	0.01027	0.005914
OTU45 (Alloprevotella)	0.042126	0.003064	0.011309	0.033518	0.001621	0.047838	0.011145	0.00163	0.036123	0.013033
OTU10 (Alloprevotella)	0.194623	0.091783	0.123736	0.117943	0.198567	0.106196	0.201187	0.211076	0.161106	0.114955
OTU71 (Alloprevotella)	0.01435	0.017054	0.000869	0.008111	0.09591	0.002122	0.013771	0.005437	0.001235	0.005252
OTU64 (Clostridia_UCG-014)	0.002957	0.000232	0.000703	0.008357	0.01335	0.004137	0.00117	0.000712	0.0079	0.00219
OTU76 (Candidatus_Saccharimonas)	0.001738	0.000236	0.000613	0.003171	0.004179	0.001462	0.004845	0.000284	0.00483	0.006165
OTU46 (g__Absconditabacteriales_)	0.007449	0	0.017619	0.001532	0.000684	0.019434	0.002667	0.000251	0.001952	0.017778
OTU42 (g__Absconditabacteriales_)	0.020868	0.000254	0.003433	0.003061	0.000325	0.009104	0.000498	0.000491	0.006573	0.006397
OTU67 (Alloprevotella)	0.003878	0.000451	0.016636	0.001834	0.001228	0.023673	0.00028	0.00925	0.009144	0.019743
OTU57 (Parvimonas)	0.0029	0.002424	0.002632	0.00272	0.000154	0.006429	0.002797	0.002318	0.006821	0.001158
OTU26 (Prevotella)	0.018524	0.022179	0.054855	0.001611	0.041167	0.053698	0.027832	0.025767	0.012016	0.038064
OTU101 (Porphyromonas)	0.004209	0.002141	0.003782	0.001288	0.000568	0.003877	0.000975	0.001225	0.000931	0.003222
OTU9 (Porphyromonas)	0.080043	0.072744	0.182632	0.06323	0.010611	0.137639	0.034803	0.00959	0.032261	0.144234
OTU380 (Porphyromonas)	0.039682	0.014094	0.020076	0.006222	0.003344	0.021323	0.002972	0.001818	0.002984	0.020894
OTU2 (Fusobacterium)	0.101099	0.08244	0.183892	0.389006	0.055598	0.298083	0.03955	0.023635	0.250056	0.266542
OTU678 (Neisseria)	0.000119	0.00468	0.0379	0.019739	0.005358	0.002909	0.007969	0.000144	0.002961	0.029248
OTU365 (Fusobacterium)	0.027671	0.006092	0.012432	0.029502	0.006018	0.020311	0.041964	0.022654	0.024933	0.018489
OTU75 (Porphyromonas)	0.020444	0.00111	0.015174	0.006366	0.000865	0.007244	0.004045	0.007467	0.043621	0.000824
OTU169 (Treponema)	0.001287	0	0.000146	6.82E-05	5.47E-05	4.91E-05	0	0.000214	0.000248	0
OTU29 (Porphyromonas)	0.013277	0	0.001292	0.003835	0	0.001134	0	0.005018	0.009838	0.00037
OTU103 (Tannerella)	0.00161	0	0.000161	0.000365	8.89E-05	3.28E-05	0.000352	0	0.000862	0
OTU87 (Corynebacterium)	0.001149	0.001394	0.000116	6.03E-05	0.000472	0.000144	0.00109	0.00998	0	0.005292
OTU172 (Prevotella)	0.002963	0.000304	0.000188	0.000236	0.000479	0	0.001767	0.001249	0.000809	0.000166
OTU114 (Prevotella)	0.007911	0.000248	0.000131	0.000351	0	0.000231	0.001429	0.001625	0.001829	0.000257
OTU119 (Prevotella)	0.000416	0.000276	6.25E-05	0.000354	0.000291	0	0.007914	0.000507	0.000337	0.000272
OTU131 (Pseudomonas)	0.000633	0.000821	0.00028	0	7.18E-05	0.001118	0.005049	0.000156	0	0
OTU31 (Pseudomonas)	0.00629	0.010636	0.001647	0.001154	0.002086	0.006314	0.055054	0.009053	0	0.000201
OTU129 (Acinetobacter)	0.00044	0.001886	0.000301	7.61E-05	0.000417	0.002119	0.007165	0.003748	0.000148	4.43E-05
OTU126 ([Eubacterium]_ruminantium_group)	0.000462	0.001609	7.44E-05	0	0.000181	0.001696	0.004553	0.005853	0	6.20E-05
OTU252 (Prevotella)	0.004068	0.013249	0.000569	0.000233	0.000619	0.018708	0.036839	0.053151	0	4.65E-05
OTU85 (Prevotella)	0.001149	0.005113	0.00025	5.51E-05	0.000605	0.006842	0.01281	0.01727	0	6.20E-05
OTU82 (Faecalibacterium)	0.002816	0.007411	0.000503	0.000121	0.000602	0.012796	0.024335	0.03472	0	0.000299
OTU69 (Fusicatenibacter)	0.00104	0.003257	0.000152	5.25E-05	0.000513	0.006371	0.01172	0.019277	0	0
OTU39 (Haemophilus)	0.003482	0.039233	0.000342	4.98E-05	0.001443	0.004652	0.001261	0.006367	0.001486	0.000819
OTU62 (Aggregatibacter)	0.006409	0.002197	0.000402	5.77E-05	0.000386	0.002963	0.000707	0.000607	0.001138	0.000531
OTU171 (Aggregatibacter)	0.003742	0.006418	0.000381	0.000123	0.001022	0.00067	0	0.002109	0.000555	0.000999
OTU5 (Haemophilus)	0.097856	0.07617	0.180524	0.011227	0.036086	0.036807	0.07751	0.026084	0.053501	0.095584
OTU20 (Actinobacillus)	0.00418	0.086496	0.000649	0	0.009619	0.000215	0	0.015226	0.004275	0.000598

H017	H019	H020	H021	H023	H024	H025	H027	H028	H029	H032	H034	H035	H036
0	5.21E-05	0	0	0	0	0	0	0	0	0	0.00011	0	0.000378
0	0	0	0	0	0	0	0	0	0	0	0	0	0
0.000339	0.00036	0.001301	0.000139	0.002141	0.003088	6.29E-05	0.000232	0.000129	0	0.001	0	0	0.000882
0	0	0	0	0	0.000458	0	0	0	0	0	0	0	0
0.001088	0.000258	0.00013	0	0.000376	0.003617	0.001807	0	0.000587	0	0	0	0.000381	0.001278
0.000381	0.0003	0	5.33E-05	7.48E-05	0.000809	0.000302	0	0	0	0	0	0.000265	0.000696
0.001949	0	0	4.48E-05	0.000176	0.00301	5.03E-05	0	0	0	0	0	0.000215	0.00015
0.000451	0	0	0	0	0	0	0	0	0	0	0	0.000255	0
0.000119	0	0.000278	0.000574	0	0	0.000134	0.000549	0	0.000395	0.000645	0	0.00024	0
0	0	0	0	0	0	0	0	0	0	0	0	0	0
0	0	0	0	0	0	0	0	0	0	0	0	0	0
0	0	0	0	0	0	0	0	0	0	0	0	0	0
0.009468	0.006509	0.012811	0.015155	0.015026	0.003855	0.02348	0.006471	0.006879	0.015386	0.011578	0.007931	0.009334	0.011714
0.072009	0.046601	0.118327	0.127305	0.095203	0.051217	0.049093	0.092182	0.052485	0.1055	0.117106	0.087343	0.067565	0.054481
0.004444	0.009979	0.0097	0.009565	0.006255	0.006955	0.010486	0.009415	0.00326	0.020376	0.010486	0.014787	0.004824	0.006404
0.002785	0.005774	0.001026	0.008131	0.0016	0.003064	0.007026	0.001354	0.003375	0.002151	0.001494	0.005019	0.003153	0.001297
0.053342	0.26801	0.097066	0.177898	0.192507	0.318871	0.063267	0.03628	0.238386	0.154482	0.013613	0.388493	0.097926	0.437658
0.003083	0.017588	0.006326	0.010323	0.008705	0.015016	0.003875	0.001219	0.011641	0.011147	0.001254	0.018935	0.007925	0.029052
0.001183	0.04731	0.006215	0.003879	0.009387	0.013136	0.012939	0.017675	0.035019	0.027114	0.00405	0.021705	0.002059	0.007512
0.00387	0.010672	0.005546	0.002432	0.006455	0.011107	0.005171	0.000391	0.003228	0.001277	0.000544	0.002492	0.000566	0.003127
0.007292	0.03181	0.013869	0.026212	0.016597	0.000952	0.010228	0.000664	0.034399	0.007041	0.022257	0.023801	0.008352	0.010485
0.061302	0.242646	0.215518	0.059624	0.103003	0.277643	0.072809	0.088537	0.092938	0.237361	0.066108	0.05297	0.120474	0.077874
0.001148	0.049966	0.01288	0.00881	0.023735	0.000494	0.000503	0.014977	0.071054	0.03383	0.011773	0.025575	0.000308	0.003152
0.001008	0.001413	0.00455	0.004056	0.002663	0	0.006037	0.000829	0.002867	0.000406	0.012329	0.012522	8.75E-05	0.005048
0.000826	0.00263	0.003342	0.003932	0.001772	0.000666	0.001271	0.000546	0.003601	0.000597	0.003357	0.004458	0.001909	0.002339
0.00845	0.000644	0.00439	0.005212	0.006926	0.004034	0.007435	0.11366	0.023355	0.002145	0.001325	0.033282	0.013643	0.013832
0.000759	6.00E-05	0.0012	0.004607	0.013897	0	0.009744	0.001876	9.40E-05	0.000511	0.018725	0.002335	0.003792	0.000345
0.010255	0.001408	0.002469	0.009638	0.04123	0.008864	0.016611	0	0.003263	0.000296	0.019707	0.005419	0.025681	0.003642
0.00802	0.00166	0.005135	0.007713	0.007239	0	0.013539	0	0.002291	0.001304	0.002603	0.000914	0.017985	0.003334
0.038079	0.035603	0.062014	0.0098	0.024192	0.032845	0.041005	0.114903	0.049303	0.029813	0.011903	0.037489	0.062919	0.027204
0.005924	0.000308	0.003502	0.004664	0.015257	0.006913	0.007374	0.003389	0.002852	0.002145	0.021091	0.000624	0.006665	0.002721
0.120722	0.004961	0.075848	0.031032	0.08301	0.004682	0.15102	0.208204	0.083811	0.074975	0.009945	0.016082	0.128603	0.027411
0.034906	0.000646	0.014092	0.004707	0.016692	0.001624	0.063319	0.002894	0.011902	0.006283	0.000701	0.001864	0.040053	0.010637
0.134217	0.145761	0.149168	0.177723	0.135942	0.004527	0.174777	0.060777	0.101397	0.037596	0.198519	0.058998	0.096838	0.167563
0.128738	0.000441	0.031259	0.002558	0.007008	0.145946	0.000461	0.029206	0.015077	0.004441	0.001958	0.009819	0.000735	0.006289
0.044147	0.01368	0.019621	0.047497	0.020519	0.003272	0.026009	0.004191	0.008377	0.01544	0.053302	0.010195	0.071861	0.019601
0.026634	0.001595	0.003067	0.03771	0.016082	0.001541	0.009807	0.000125	0.00712	0.001449	0.003289	0.005588	0.035003	0.001215
0.001645	0.000117	0	0.000988	0	0	0.000411	0	0	0	0.000849	0.00042	0	0.000123
0.029342	0.006465	0.012405	0.107729	0.003669	0.001077	0.015009	0.000101	0.001351	0.019914	0.002328	0.004293	0.034808	0.005364
0.000857	0.000211	0.002236	0.003636	0.00028	0	0.001503	0	0.0002	0.001078	0.000388	0.000958	0.001976	0.000125
0.023713	0.001006	0.001658	0.03462	0.000836	0.006056	0.00021	0.001462	0.001269	0.000347	0.167047	0.000518	0.000461	0.00022
0.003877	0.000279	0.001193	0.002296	0.001052	0.000268	0.001472	0.000657	0.000761	0.007818	0.011933	0.000255	0.002083	0.000484
0.00451	0	0.001945	0.009298	0.0028	0.000756	0.00672	0.000236	0.000884	0.004165	0.098336	0.000471	0.001324	0
0.005654	0	0.000145	0.00226	0.001065	0.000482	0.00311	0.000933	0.000928	0.003022	0.030347	0	0.001278	0
0	0.000149	5.90E-05	0.000241	9.02E-05	0	0.000327	0.000616	0.000129	0.000492	0.000388	0	7.12E-05	0
0.000493	0.001061	0.003421	0.002697	0.000634	0.000202	0.001021	0.005925	0.001081	0.006326	0.003863	0.00051	0.002234	0
0	0.000112	0.000814	0.000444	0.000165	0	9.44E-05	0.006805	0	0.001057	0.000737	8.63E-05	0.000203	0
0	0	0.000394	0.00013	9.90E-05	0	3.15E-05	0.002469	0	0.000997	0.000195	0	5.49E-05	0
0.000101	0.000683	0.002622	0.000683	0	0	0.000149	0.021448	0	0.005829	0.001624	0	0.004447	0.000217
0	0.000167	0.000674	0.00039	0	0	0.00017	0.005845	0	0.001234	0.000231	0	0.003425	0
0.000112	0.000334	0.002272	0.000288	0.000218	0.000149	0.000174	0.015516	9.69E-05	0.004519	0.001044	0.000235	0.001134	0.000109
0.000105	4.95E-05	0.000954	9.39E-05	7.48E-05	0.00022	0.000101	0.007017	0	0.002132	0.000408	0.000145	0.004554	0.00024
0.000504	0.000454	0.003748	0.003023	0.015001	0	0.005605	0.000559	0.001939	0.003861	0.015406	0.000883	0.005681	0.003063
0.004503	0.000287	0.001702	0.002095	0.017984	0.003486	0.000717	0.000718	0.001439	0.000828	0.011886	0.005545	0.001168	0.000784
0.006305	0.000759	0.006822	0.003653	0.001395	0.012202	0.00135	0.000478	0.00074	0.005213	0.017482	0.001271	0.004132	0.004834
0.130981	0.037488	0.070147	0.016438	0.063738	0.040431	0.164903	0.117464	0.078612	0.137391	0.009324	0.131774	0.100965	0.046776
0.00036	0.001736	0.006136	0.008003	0.017231	0.006467	0.007278	0.001206	0.041875	0.000317	0.00552	0.003885	0.000381	0.000339

H039	H040	H041	H044	H048	H049	H051	H053	H057	H058	H059	H060	H061	H062
0.000181	0	0	0	0.000455	0	0	0	0	0	0	0	0	0
0	0	0	0	0	0	0	0	0	0	0	0	0	0
0.010284	0.000253	0.00905	0	0	0.000566	0	0	0.000182	0	0	0	0.000906	6.30E-05
0.00098	0	0.000499	0	0	0	0	0	0	0	0	0	0.000395	0
0.001298	0	0.000626	0	0.000457	0.000613	0	0.00238	5.70E-05	0	0.000586	0.000298	0.001934	0
0.000952	0	0.000499	0	0.000151	0	0	0	0	0	0	0	0.003173	0
0.00011	0	0.000254	0	0.001569	0	0	0	0	0	0	0	0.001594	0
0.000243	0	0	0	0	0	0	0	0	0	0	0	0.000873	0
0	0.00097	0.000308	0.00031	0.000583	0.00042	0.000183	0	0.000209	0	0	0.001081	0	0.003977
0	0	0	0	0	0	0	0	0	0	0	0	0	0
0	0	0	0	0	0	0	0	0	0	0	0	0	0
0	0	0	0	0	0	0	0	0	0	0	0	0	0
0.011898	0.013524	0.00292	0.016292	0.023665	0.020191	0.012426	0.000878	0.007934	0	0.012514	0.00249	0.014877	0.015832
0.0837	0.132551	0.073875	0.090927	0.106684	0.06778	0.136082	0.018317	0.076749	0.069786	0.064381	0.078864	0.104526	0.131852
0.008672	0.010761	0.011443	0.016881	0.009817	0.007005	0.012055	0.001705	0.008627	0.009287	0.005075	0.012885	0.006224	0.027829
0.004124	0.001442	0.006075	0.003132	0.000614	0.001281	0.003302	0	0.00662	0.000507	0.002786	0.004799	0.001735	0.006268
0.061928	0.086797	0.253371	0.301021	0.070718	0.066704	0.068559	0.008756	0.306397	0.057646	0.016975	0.107064	0.084589	0.103645
0.005799	0.007621	0.011942	0.026893	0.005722	0.003882	0.00466	0	0.022939	0.005014	0.000724	0.008384	0.006284	0.008759
0.013805	0.012893	0.041421	0.009511	0.002879	0.011172	0.00262	0	0.081511	0.011034	0.002899	0.021553	0.003889	0.006882
0.009177	0.004886	0.003618	0.002114	0.002191	0.00447	0.002293	0	0.020842	0.001027	0	0.003778	0.009512	0.00329
0.008064	0.059664	0.048848	0.001855	0.003572	0.001286	0.023731	0.000288	0.000921	0.00246	0.000842	0.049905	0.011322	0.005446
0.159282	0.134686	0.273066	0.018537	0.011843	0.036333	0.176529	0.001223	0.047424	0.037166	0.011711	0.297642	0.062939	0.039025
0.014607	0.13672	0.006737	0.000545	0.021016	0.000698	0.005132	0	0.000206	0.00492	0	0.029499	0.001102	0.001422
0.01808	0.003666	0.037985	0.00239	0.000983	0.000368	0.001503	0	0.008945	0.000238	0	0.001055	0.002948	0.001283
0.0032	0.007935	0.032907	0.000616	0.001118	0.004004	0.001931	0.000918	0.002102	0.001492	0.003665	0.003193	0.001537	0.000455
0.00037	0.006756	0.000807	0.000357	0.00192	0.021117	0.00851	0.004133	0.035912	0.007619	0.00958	0.001094	0.00508	0.005363
0.000172	0.003391	0	0.000441	0.001786	0.014092	0.007108	0	0.026745	0	0.000597	0.001233	0.004983	0.001416
3.01E-05	0.001601	0.001152	0.000224	0.001786	0.015181	0.042479	0	0	0.000267	0.00092	0.004831	0.009146	0.019932
0.001027	0.004714	0.000689	0.002237	0.004007	0.012323	0.01247	0	0.003194	0	0.011171	0.005565	0.001541	0.003945
0.019582	0.020113	0.007844	0.007747	0.068979	0.03096	0.020176	0	0.025365	0.011217	0.012862	0.000943	0.015027	0.022934
0.004079	0.00209	0.001306	0.000522	0.004525	0.001201	0.004139	0	0.000608	0.000772	0.002069	0.000705	0.008822	0.001483
0.094212	0.017334	0.01012	0.012404	0.06131	0.07196	0.062766	0.014644	0.018308	0.043608	0.099842	0.006521	0.118951	0.080351
0.04086	0.004991	0.000889	0.003452	0.015563	0.005488	0.011111	0.000278	0.004167	0.008874	0.013275	0.000459	0.028033	0.012017
0.319209	0.06704	0.089118	0.283992	0.076108	0.136009	0.098868	0.00202	0.156424	0.072424	0.062737	0.052962	0.327523	0.100436
0	0.0051	0.000834	0.001991	0.033827	0.010268	0.005466	0.002913	0.002716	0.000787	0.000397	0.002745	0	0.026213
0.036564	0.04253	0.016966	0.025451	0.04094	0.11117	0.029101	0	0.01971	0.023442	0.005347	0.017894	0.045126	0.041706
0.002947	0.016583	0.002784	0.002821	0.073991	0.014705	0.020264	0.000295	0.001559	0.005642	0.000185	0.004184	0.096439	0.012677
0.000142	0.000178	0	0.000424	0.000668	0.00032	0.000837	0	0	0	0	0	0.000238	0.000396
0	0.002776	0.000299	0.007227	0.017617	0.155667	0.030492	0.001163	0.004365	0	0	0.030308	0.010118	0.011486
0.000355	0.000609	0.000245	0.00019	0.00295	0.015323	0.001965	0	0.000736	0	0	0.0068	0.001281	0.002286
0.000866	0.004424	0	0.000865	0.002016	0.001144	0.000168	0	0.002515	0.002034	0	0.000667	0	0.004593
0.003525	0.001098	0.003709	0.000811	0.001096	0.000908	0.000995	0	0.002442	0.003794	0	0.002685	0.000767	0.001499
0.003048	0.000447	0.000245	0.000111	0.000907	0.000135	0.004479	0	0.001823	0.005216	0	0.004621	0.000214	0.000162
0.001178	0.002518	0.000916	0.000552	0.000214	0.000501	0.002956	0	0.001393	0	0	0.001585	0.000269	0.001114
0	0.000778	0.000635	7.39E-05	0.000205	0.000836	0.000201	0.000598	0.000212	0	0	0.002031	0.000205	0.011284
0.000234	0.008938	0.005404	0.002264	0.002065	0.007245	0.00251	0.039175	0.001698	0.023635	0.038625	0.022642	0.000392	0.083629
6.23E-05	0.000575	0.000199	0.000256	0.000167	0.000838	0.001396	0.061866	0.000511	0.017352	0.043596	0.016543	0	0.011561
0	0	0	5.91E-05	0	0.000225	0.000303	0.03642	0	0.025434	0.020769	0.003976	0	0.002264
0.000155	0.000867	0.002086	0.000473	0.000359	0.002077	0.001846	0.340978	0.000739	0.220678	0.199291	0.031957	0	0.018299
4.94E-05	0.000214	0.000798	5.42E-05	0	0.000378	0.000762	0.124071	0.00038	0.074523	0.075419	0.011946	9.48E-05	0.006954
0.000114	0.000589	0.000553	0.000355	0	0.001136	0.001676	0.224822	0.000646	0.150872	0.145426	0.022878	0	0.014414
0	0.000347	0.003264	7.89E-05	0	0.000483	0.000879	0.101693	0.000478	0.053835	0.065493	0.009403	0	0.004944
0.000361	0.003302	0	0.002099	0.091967	0.0001	0.031067	0	0.01149	0	0.004053	0.000901	0	0.000464
0.000913	0.013387	0.000907	0.000505	0.008921	0.00024	0.006205	0.000295	0.00088	0.000728	0.000205	0.004271	0	0.006103
0.001079	0.012934	0.001152	0.007018	0.013306	0.000158	0.001958	0	0.000657	0.005439	0	0.009017	0	0.00365
0.052166	0.091802	0.031347	0.142204	0.208602	0.140989	0.128408	0.010171	0.076239	0.041229	0.065984	0.090306	0.005232	0.11185
0.000316	0.047605	0.00029	0.001713	0.000164	5.01E-05	0.007434	0	0.006421	0	0	0.005841	0.000157	0.018546

H063	H064	H065	H066	H067	H070	H071	H073	H074	H075	H076	H078	H079	H083
0	0	0	0	8.77E-05	0.000351	0	0.000488	0	0	0	0.000159	0	8.16E-05
0	0	0	0	0	0	0	0	0	0	0	0	0	0
0.000141	0.000412	6.21E-05	5.93E-05	7.87E-05	0.001738	0.000711	0.000741	0	0	0.004609	0.002824	0.000722	0.000343
0	0	0	0	0	0.002145	0.000762	0.000677	0	0	0.000102	0.001039	0.000433	0
0	0.001008	0	0	7.20E-05	0.000619	0.000391	0.002251	6.29E-05	0	0.00022	0.002253	0.000953	0.00042
0	0.000364	2.36E-05	0	0.000196	0.000471	0.000623	0.001789	5.64E-05	0	0	0.002182	0.001142	0.000394
0	0.001133	0	0	0.000272	0.000855	0	7.52E-05	0.000141	0	0	0.000689	0	0.000606
0	6.74E-05	0	0	0	0	0	0	6.94E-05	0	0	0.000419	0	0.000235
0.000394	0.000217	0.000504	0.006311	0.000238	7.68E-05	8.79E-05	0.000293	0	0.00566	0	0	0	0.000131
0	0	0	0	0	0	0	0	0	0	0	0	0	0
0	0	0	0	0	0	0	0	0	0	0	0	0	0
0	0	0	0	0	0	0	0	0	0	0	0	0	0
0.016773	0.004396	0.015383	0.00908	0.00562	0.061731	0.00399	0.004299	0.006789	0.052917	0.00401	0.010302	0.003912	0.013491
0.181987	0.10075	0.144721	0.382647	0.051674	0.257795	0.106659	0.215655	0.022779	0.133726	0.026326	0.063693	0.081379	0.102054
0.034165	0.009647	0.017602	0.039414	0.004066	0.021661	0.009292	0.024545	0.002125	0.013666	0.002218	0.003981	0.009508	0.007483
0.002223	0.00103	0.000801	0.000803	0.004516	0.001718	0.00281	0.003669	0.001145	0.00292	0.002818	0.003506	0.003876	0.002207
0.007135	0.079964	0.064475	0.022743	0.219493	0.077367	0.062911	0.221776	0.115571	0.031786	0.125481	0.155075	0.237117	0.155909
0.000838	0.007559	0.003212	0.001646	0.016429	0.003072	0.005052	0.01337	0.010438	0.002726	0.009438	0.008632	0.013317	0.013157
0.008664	0.007469	0.003481	0.004602	0.005982	0.019285	0.066005	0.020193	0.000967	0.002141	0.076188	0.01643	0.064067	0.002782
0.000179	0.003597	0.000263	0	0.007343	0.001029	0.014134	0.003349	0.002587	0.001154	0.00362	0.007086	0.010403	0.004034
0.000609	0.000549	0.004003	0.008914	0.011438	0.000412	0.035224	0.0358	0.002532	0.003442	0.02571	0.031642	0.038225	0.003999
0.007647	0.074239	0.111114	0.04088	0.098581	0.020215	0.246495	0.034109	0.11811	0.039231	0.119169	0.123698	0.174905	0.034941
0.000291	0.00015	0.001501	0.010363	0.006383	0.019695	0.031748	0.149702	0.000557	0.000444	0.093679	0.056079	0.007063	0.00455
0.000309	0.001325	0.001383	0	0.000281	0.005002	0.008112	0.011707	0.000733	0.000437	0.004379	0.006335	0.006469	0.002686
0.000911	0.000499	0.000297	0.00142	0.00063	0.002048	0.001313	0.007767	0.000512	0.000144	0.001957	0.004059	0.004794	0.002533
0.01769	0	0.008212	0.000234	0	0.000299	0.06441	5.57E-05	0.016294	0.018099	0.012327	0.070591	0.051796	0.010379
0.001288	0.000299	0.002405	0.000874	0.001167	0.000307	0.006591	4.46E-05	0.002088	0.000568	0.06821	0.032727	0	0.002392
0.036588	0.00256	0.131967	0	0.012023	0.000228	0.008304	0.003747	0.004761	0.01293	0.01592	0.012611	0.000812	0.02925
0.003557	0.009009	0.001952	0	0.00155	9.47E-05	0.002706	0.002647	0.006457	0.001347	0.004931	0.000703	0.003071	0.003989
0.014188	0.098884	0.021699	0.00889	0.084718	0.010746	0.005678	0.012211	0.022053	0.037777	0.027417	0.018151	0.008529	0.045407
0.005518	0.000309	0.002744	0.000147	0.000634	0.002178	0.000956	0	0.001587	0.00424	0.005205	0.001412	0.003052	0.004187
0.138343	0.011147	0.068043	0.004908	0.020229	0.029125	0.005436	0.000468	0.13335	0.112778	0.03952	0.026834	0.020168	0.127829
0.026692	0.003141	0.012587	0.001378	0.004655	0.008593	0.001129	0	0.002166	0.015744	0.003877	0.015456	0.004399	0.023209
0.095252	0.254148	0.074955	0.022056	0.217209	0.043119	0.073513	0.03517	0.301261	0.059498	0.190253	0.185516	0.139266	0.145634
0.070403	0.061019	0	0.001207	0.001255	0.000118	0.000429	0.002059	0.043377	0.021423	0.018757	0.00029	0.00121	0.030105
0.009367	0.028231	0.009149	0.049821	0.03532	0.016408	0.028038	0.018978	0.022488	0.012185	0.025161	0.014113	0.03869	0.020668
0.007891	0.024763	0.002666	0.012985	0.021489	0.000604	0.076051	0.036466	0.017584	0.006171	0.013471	0.034291	0.006148	0.006696
0	0.000826	0.00048	0.000313	0.000101	0	0.000173	0.004265	0	0.001304	0	0.001055	0.000738	0.001766
0.048824	0.007152	0.002405	0.059012	0.000153	0	0.007076	0.008486	0.002493	0.006972	0	0.004829	0.003238	0.006869
0.001329	0.000798	0	0.004334	0.000947	0.000653	0.001294	0.000571	0.001401	0.000229	0	0.00062	0.000221	0.000563
0.012507	0.000324	0.001717	0.001294	0.000753	0.001001	0.000487	0.002064	0	0.000754	0.000989	0	0.000279	0.001417
0	0.000514	0.000663	0.017127	0.000279	0.000753	0.008038	0.003789	8.24E-05	0.00187	0.001195	0.001002	0.009325	0.001132
0	5.74E-05	0.000395	0.028636	0.002766	0.001753	0.007974	0.011556	0.000241	0.005423	0.005777	0.000663	0.013612	0.000903
3.82E-05	0.000122	0.000108	0.013915	0.001178	0.000988	0.007444	0.001741	0	0.000521	0.000891	0.00021	0.002898	0.000661
0.000597	0.000145	0.000928	0.00666	0.000418	0	7.46E-05	0.000212	0	0.006087	0	0	0	0.000259
0.007115	0.001265	0.011853	0.069971	0.001262	0.002086	0.001177	0.001998	0.000241	0.065142	0.000969	0.000246	0.000443	0.001691
0.000714	0.000259	0.001207	0.011754	0	0.000225	9.32E-05	8.08E-05	0	0.00418	8.47E-05	0	0	0.000151
0.000132	0	0.000163	0.002204	0	9.22E-05	0	0	0	0.000189	0	0	0	0.000104
0.001197	5.99E-05	0.002126	0.018763	0.000245	0	0.000397	8.92E-05	0	0.001539	4.74E-05	0.000108	0	0.000186
0.000447	8.98E-05	0.000891	0.006222	0.000155	0	0	0	0	0.000314	7.11E-05	0	0	0.000145
0.00065	0.000115	0.001967	0.01283	9.67E-05	0.000184	0.002418	5.57E-05	0	0.000785	8.13E-05	8.06E-05	0.000266	0.000691
6.76E-05	0	0.001083	0.005142	0	0	0.001028	0	0	0.000442	0	0	0	0.000186
0.000367	0.000631	0.069654	0.00264	0.000153	0.006461	0.003867	0.002647	0.002864	0.016771	0.000623	0.005978	0.002988	0.006327
0.017901	8.48E-05	0.008667	0.00063	0.000349	0.000701	0.009944	0.006352	0.001438	0.000369	0.005879	0.003248	0.002606	0.001171
0.005809	0.000706	0.006497	0.009471	0.001199	0.007531	0.004235	0.001649	0.000425	0.002951	0.005588	0.001055	0.001752	0.020789
0.205742	0.179919	0.176193	0.092955	0.146175	0.368374	0.029519	0.087082	0.131696	0.153751	0.049489	0.066163	0.025993	0.153027
0.00752	0.019047	0.007797	0.004745	0.010141	9.22E-05	0.0452	0.003262	0.000479	0.137252	0.003346	0.001965	0.000212	0.000182

H084	H086	H088	H089	H090	H091	H092	H093	H094	H096	H097	H098	H099	H100
0	5.74E-05	0	7.87E-05	0.001256	0	0	6.20E-05	0.000677	0	0.001019	0	0	0
0	0	0	0	0	0	0	0	0.000131	0	0	0	0	0
2.99E-05	0.000203	5.97E-05	0.006718	0.003834	0.001088	0.000284	0.001122	0.003735	4.77E-05	0.003048	0.000104	8.33E-05	7.19E-05
0	0	0.000203	8.63E-05	0	0	0.000147	0.000618	0.000459	0	0.000566	3.27E-05	0	0
0	5.74E-05	0.001425	0.000569	0.003804	0	0.000147	0.001888	0.001275	4.22E-05	0.000774	0	0	8.52E-05
0	0.000499	0.001179	0.000757	0.000638	0	0.000612	0.001888	0.000668	0	0.000412	0	0	0
0	0.000351	0.001117	9.14E-05	0.002612	0	0.000214	0.002288	0.000589	0	0.001189	0	0	0
0	0	0.000392	0.000185	0	0	0.000119	0.000344	0	0	0.000692	0	0	0
0.000549	0.0004	0.000486	0	0	0.001436	0	0	0.000112	0.000879	0	0.000323	0.003361	0.000349
0	0	0	0	0	0	0	0	0	0	0	0	0	0
0	0	0	0	0	0	0	0	0	0	0	0	0	0
0	0	0	0	0	0	0	0	0	0	0	0	0	0
0.022187	0.007813	0.015457	0.002298	0.000827	0.011737	0.024346	0.004982	0.004033	0.019354	0.001856	0.000986	0.010473	0.011524
0.218662	0.082801	0.205923	0.027487	0.082349	0.110023	0.106931	0.118048	0.051632	0.210092	0.037739	0.159418	0.091412	0.170711
0.020616	0.006565	0.015026	0.002455	0.009725	0.013006	0.009604	0.008385	0.005181	0.014738	0.003426	0.017115	0.019822	0.019198
0.000514	0.005874	0.002103	0.005535	0.001608	0.001968	0.004069	0.001006	0.001193	0.002187	0.001425	0.003464	0.004692	0.008782
0.04133	0.189674	0.090579	0.066968	0.132612	0.251307	0.101683	0.212192	0.338719	0.019205	0.165292	0.088072	0.115699	0.242081
0.004246	0.012822	0.005579	0.003392	0.009456	0.015139	0.007453	0.011409	0.02321	0.001346	0.011126	0.007173	0.005919	0.015101
0.001292	0.004512	0.005943	0.002988	0.075215	0.012055	0.007604	0.001172	0.031425	0.003103	0.015709	0.038287	0.02794	0.034118
0.001061	0.004653	0.00232	0.001983	0.011317	0.001792	0.002134	0.001832	0.004835	0.000523	0.007487	0.002371	0.002401	0.007225
0.004676	0.04081	0.004632	0.024432	0.02923	0.030369	0.02387	0.00133	0.001142	0.036906	0.026159	0.000276	0.019689	0.018391
0.024332	0.11431	0.118108	0.102054	0.188066	0.111824	0.199536	0.061979	0.136609	0.025104	0.112571	0.081704	0.082949	0.15258
0.006749	0.001652	0.0094	0.00278	0.112587	0.009278	0.012739	0.010859	0.063212	0.007216	0.038232	0.152277	0.277756	0.093065
0.000656	0.006733	0.00186	0.006373	0	0.000277	0.002416	0.00255	0.006951	0.002122	0.000271	0.001411	0.003028	0.002553
0.000297	0.00144	0.003037	0.001252	0.000214	0.001878	0.003054	0.001682	0.006784	0.001493	0.001217	0.001095	0.003164	0.002643
0.006011	0.011607	0.000281	0.011329	0	0.003782	0.007189	0.013967	0.029436	0.037378	0.022067	0.078896	0.010314	0.004528
0.001596	0.010558	5.12E-05	0.018042	0	0.00036	0.011512	0.002206	0.001017	0.002611	0.017226	0.025418	0.008866	0.00271
0.013802	0.014023	0.002267	0.003859	0.000202	0.007175	0.019171	0.011151	0.002044	0.019113	0.007537	0.026541	0.004487	0.015567
0.002022	0.018888	0.008969	0.006304	0	0.0117	0.011939	0.00117	0.000656	0.003889	0.001233	0.00027	0.002603	0.000138
0.007389	0.020904	0.00814	0.013573	0.013693	0.049221	0.031452	0.02602	0.021115	0.02011	0.046053	0.014481	0.006677	0.012189
0.002298	0.002635	0.001077	0.00375	0	0.002312	0.002822	0.004342	0.009609	0.0017	0.001677	0.000496	0.003624	0.000423
0.059437	0.065983	0.018657	0.057556	0.002742	0.061159	0.036964	0.170735	0.032449	0.064879	0.082067	0.010028	0.013199	0.003027
0.016107	0.024133	0.00432	0.028975	0.001873	0.006052	0.006322	0.034163	0.016918	0.007105	0.015263	0.000976	0.002743	0.001669
0.086043	0.141662	0.050918	0.449428	0.030393	0.054928	0.15753	0.132665	0.075644	0.097526	0.173666	0.057404	0.035245	0.053929
0.003824	0.030265	0.024526	0.008224	0.005148	0.00543	0.0049	0.028698	0.003456	0.005743	0.000123	0.001577	0.002154	0.000474
0.030092	0.034993	0.082249	0.064304	0.006773	0.053644	0.04279	0.012295	0.024653	0.049229	0.0166	0.005331	0.02793	0.006855
0.003803	0.04335	0.03559	0.026116	0.000672	0.023373	0.025588	0.001372	0.006632	0.013787	0.014697	0.005851	0.010319	0.000269
0.000843	0.001003	0	8.89E-05	0	0.000885	0.001786	0	0.000501	0.000362	0	0	0.000298	0
0.02359	0.034518	0.134025	0.003242	0.001268	0	0.024997	0.001578	0.008196	0.002339	0.000981	5.51E-05	0	0
0.003875	0.005375	0.004035	0.000462	0	0.001883	0.00263	0.000414	0.000465	0.000145	0.000557	0.000118	0.000397	0.000189
0.000157	0.002392	0.001847	0.006253	0.002276	0.011403	0.000284	0.00021	0.001108	0.000165	0	0.00011	0.000365	5.32E-05
0.00066	0.000137	0.004103	0.00032	0.001419	0.006636	0.002977	0.00107	0.001209	0.001217	0.000264	0.000176	0.001371	7.19E-05
0.000162	0.000124	0.002779	0.000353	0.001495	0.02199	0.000539	0.001352	0.00174	0.000628	0	0	0.002131	0.000186
0.003013	0.000351	0.002002	0.000955	0.000857	0.001919	0.000481	0.000228	0.001184	0.008605	0.000409	0.000214	0.003834	0.000823
0.001107	0.000197	0.000254	0	0.000189	0.002314	0.00026	0	0.000319	0.001015	0	0.001172	0.007007	0.001006
0.009279	0.001692	0.003881	0.000587	0.00042	0.021672	0.001496	0.000398	0.001093	0.012081	0.000425	0.011542	0.069174	0.007041
0.000815	0.000108	0	0	0	0.00176	5.34E-05	7.60E-05	0	0.001434	0	0.001303	0.004774	0.000889
0.000176	0	0	0	0	0.000515	0	0	0	0.000257	0	0.000208	0.000393	0.000266
0.001552	0.000126	0.000134	0	0.000626	0.006974	0.000145	0.000554	0.000106	0.001695	0.000129	0.00214	0.005985	0.001903
0.000781	6.85E-05	0	0	0	0.003143	5.86E-05	0.000118	0	0.00054	0.00012	0.000723	0.001773	0.000804
0.001542	5.96E-05	0	0.000129	0	0.004907	0.000234	0.000196	0	0.001871	0	0.00123	0.004032	0.001344
0.000454	5.08E-05	6.82E-05	0	0	0.002424	0	0	0	0.000604	0	0.000647	0.001523	0.000607
0.012331	0.001095	0.015098	0.00019	0.001625	0.001772	0.01377	0.001806	0.002211	0.004111	0.001733	0.012937	0.026902	0.045474
0.002233	0.000872	0.002909	0.00214	0.032732	0.000939	0.019654	0.001552	0.009284	0.000406	0.024879	0.001381	0.012407	0.000122
0.004357	0.004759	0.007379	0.001335	0.020307	0.007268	0.00248	0.000134	0.008345	0.002143	0.006074	0.002128	0.008498	0.000902
0.111838	0.045682	0.052166	0.033484	0.155254	0.049283	0.062626	0.102815	0.057518	0.286794	0.13119	0.161562	0.047414	0.057788
0.241616	0.001164	0.047447	0.000518	0.054685	0	0.000388	0.003084	0.000519	0.006162	0.004822	0.020975	0.005172	0.000274

H102	H104	H107	H108	H109	H110	H113	H114	H116	H118	H120	H122	H123	H124
8.80E-05	0	0	0	4.50E-05	0	0	0.00051	0	0	0	0	0	0
0	0	0	0	0	0	0	9.22E-05	0	0	0	0	0	0
0.00035	0	0.000375	6.20E-05	0	0.000328	0.000201	0.000141	0.013196	0	0	9.90E-05	0.0007	0.000354
0	0	0	0	3.72E-05	0	0	0	0	0	0	3.79E-05	9.08E-05	0
0	4.80E-05	0.000326	4.56E-05	0.000855	0	0	0.000209	0.000395	0	0.000124	0	0.000236	3.63E-05
0.000108	0	0	0	0.000781	0	0	0.000193	7.84E-05	3.99E-05	0.000119	0	7.06E-05	0
9.24E-05	0	0	0	0.000763	0	0	0.000168	0.000235	0	0	0	0	5.36E-05
0	0	0	0	0.000155	0	0	0	0.000135	0	0	0	0	0
0.00015	0.001216	0.002507	0.000376	7.05E-05	0.000334	0.000199	0.000127	5.95E-05	0	0.000165	0.000438	0.000486	0.000432
0	0	0	0	0	0	0	0	0	0	0	0	0	0
0	0	0	0	0	0	0	0	0	0	0	0	0	0
0	0	0	0	0	0	0	0	0	0	0	0	0	0
0.011145	0.108412	0.041055	0.01932	0.024941	0.051352	0.042795	0.006632	0.008426	0.015939	0.005953	0.013589	0.008538	0.021028
0.082259	0.115437	0.106732	0.193816	0.129574	0.184018	0.150168	0.050837	0.020561	0.089458	0.086764	0.223122	0.067964	0.224209
0.004751	0.023823	0.019636	0.012517	0.010989	0.016789	0.016675	0.012059	0.00133	0.007785	0.007742	0.013484	0.008755	0.018124
0.003512	0.001486	0.000492	0.003533	0.001454	0.000859	0.003681	0.004454	0.005154	0.002505	0.000722	0.001556	0.000541	0.002554
0.031628	0.06522	0.073782	0.074306	0.112465	0.034308	0.098501	0.117772	0.262446	0.040845	0.131287	0.06573	0.036097	0.080407
0.001384	0.006561	0.006387	0.007483	0.00923	0.002307	0.007137	0.008039	0.020898	0.002898	0.009031	0.004034	0.00429	0.006412
0.004656	0.002501	0.013335	0.011489	0.000675	0.006876	0.008963	0.030633	0.002965	0.007224	0.004484	0.003138	0.012067	0.011234
0.001285	0.001341	0.000729	0.004069	0.002239	0.001694	0.001338	0.002352	0.011994	0.002133	0.000402	0.001227	0.002469	0.003191
0.028694	0.006038	0.001573	0.031648	0.023452	0.012327	0.002683	0.004527	0.042737	0.007298	0.001378	0.014531	0.025272	0.022705
0.083513	0.14838	0.08068	0.147003	0.152904	0.041944	0.1976	0.034598	0.102333	0.147976	0.093202	0.03244	0.127426	0.159989
0.000537	0.002567	0.002304	0.005516	0.001712	0.006288	0.022345	0.087676	0.077724	0.011837	0.018056	0.006202	0.01929	0.033186
0.003633	0.000306	0.000457	0.000361	0.001325	0.002295	0.003671	0.003418	0.013623	0.004314	0.001265	0.003312	0.001446	0.005499
0.005151	0.001068	0.005242	0.001991	0.000679	0.002185	0.002297	0.003458	0.008472	0.001578	0.000425	0.001757	0.000387	0.001746
0.016669	0.009259	0.002901	0.013335	0.025667	0.009254	0.03321	0.000217	0.002616	0.040223	0.001202	0.003695	0.040001	0.002837
0.00248	0.003368	0	0.007582	0.004715	0.003826	0.006706	0.000274	0.002748	0.005229	3.84E-05	0.002153	0.005377	0.001523
0.106904	0.004986	0.01567	0.039056	0.049158	0.038479	0.030088	0.004852	0.005064	0.001208	0.003167	0.008654	0.013021	0.03177
0.010355	0.003407	0.002454	0.002328	0.006433	0.007444	0.003917	0.000247	0.000976	0.002166	0.001211	0.001819	0.003273	0.001626
0.020839	0.010555	0.06691	0.052277	0.041856	0.045543	0.040254	0.144939	0.007213	0.127744	0.018546	0.005045	0.02531	0.033471
0.006921	0.002038	0.001473	0.003994	0.005877	0.01475	0.010815	0.003171	0.001494	0.003646	0.000733	0.005116	0.001069	0.005044
0.096218	0.059437	0.109827	0.069018	0.08718	0.102145	0.103223	0.037042	0.032749	0.16239	0.010837	0.012226	0.029751	0.043712
0.011205	0.01718	0.014738	0.024476	0.02846	0.016192	0.01062	0.002143	0.012196	0.025178	0.002431	0.001826	0.006192	0.007146
0.279187	0.075794	0.067662	0.058557	0.127095	0.174744	0.06141	0.209424	0.249193	0.135398	0.029943	0.068477	0.104103	0.056381
0.010459	0.00169	0.001299	0.000749	0.021052	0.000239	0.000455	0.000296	0.000143	0.002727	0.001321	0.005961	0.013963	0.0026
0.0171	0.011884	0.017147	0.009667	0.01466	0.020183	0.014295	0.019957	0.019647	0.015479	0.01343	0.017911	0.01427	0.01983
0.015821	0.002224	0.000545	0.009091	0.011658	0.002961	0.003551	0.001513	0.031568	0.015275	0.004368	0.004873	0.013703	0.005023
0.000112	0	0	0.00076	0.000211	0.000351	0	0	0.000127	0	0.003107	3.79E-05	4.84E-05	0.001515
0.00024	0.004613	0.00236	0	0	0.005593	0.000565	0	0.000378	0	0.000274	0.004378	0.006579	0.009023
0.000618	0.000876	0.001655	0.000383	0.000523	0.000487	0.000344	0.000567	0	0.000721	0.00028	0.00015	3.43E-05	0.000809
0.000849	0.000508	0.00254	0.001072	0.002147	0.012366	0.005911	0.001435	0.000327	0.002077	0.000406	0.00161	0.00025	0.001106
0.000165	0.001158	0.005023	0.000798	0.000767	0.003857	0.001846	0.006775	0.002119	0.000192	0.001405	0.000866	0.000288	0.000991
7.92E-05	0.000666	0.010111	0.000461	0.000454	0.001925	0.001053	0.000252	0.001684	0.00026	0.002185	0.000689	0.000159	0.002877
8.36E-05	0.000337	0.010101	0.000474	0.000777	0.000892	0.000547	0.000464	0.000397	0.000593	0.000425	0.001021	0.000141	0.003617
0.000152	0.007233	0.005041	0.000427	0.000186	0.00096	0.000506	0.000168	0.000116	0.000267	0.000281	0.000699	0.000805	0.000415
0.001888	0.053282	0.060642	0.005254	0.001433	0.007103	0.00879	0.00217	0.00077	0.00277	0.006689	0.010591	0.0114	0.0051
0.000253	0.005121	0.010261	0.00045	7.63E-05	0.001154	0.003181	0.000157	0	0.000748	0.001296	0.001386	0.001257	0.001119
0	0.001351	0.004632	0.000155	8.02E-05	0.000268	0.000557	8.14E-05	0	0.001117	0.000278	0.000121	0.000377	0.00014
0.000244	0.009772	0.025407	0.00058	2.94E-05	0.002918	0.006243	3.25E-05	0.00027	0.009447	0.002273	0.001035	0.003413	0.002248
7.92E-05	0.00287	0.005906	0.000385	4.89E-05	0.000945	0.002049	0	0.000108	0.00358	0.000527	0.000208	0.001222	0.000639
0	0.006783	0.020433	0.000709	0.000145	0.001738	0.004116	0.000152	0.000359	0.005648	0.001492	0.000578	0.00168	0.001626
0	0.003008	0.008282	0.000129	0	0.000761	0.00201	0.000198	3.51E-05	0.002449	0.000746	0.000265	0.000801	0.000576
0.007506	0.001075	0.000207	0.003757	0.00109	0.017664	0.005981	5.70E-05	0.000203	0.006876	0.001972	0.032075	0.027956	0.016217
0.011839	0.000724	0.000928	0.017198	0.001611	0.015208	0.022091	0.001359	0.001203	0.000714	0.00026	0.002794	0.012063	0.029821
0.001668	0.000774	0.004472	0.002313	0.002855	0.004354	0.009379	0.004424	0.000303	0.009589	0.010753	0.016923	0.00215	0.014017
0.112341	0.10146	0.159216	0.152054	0.079581	0.112433	0.046277	0.15019	0.033189	0.073231	0.186571	0.395541	0.135335	0.09933
0.004788	0.112159	0.006543	0.008973	0.009828	0.009359	0.001752	0.039548	4.05E-05	0.001229	0.33043	0.006578	0.207883	0.006691

H125	H126	H127	H130	H131	H133	H137	H139	H141	H142	H146	H147	H149	H150
0	0	8.40E-05	0.000341	0	0	6.41E-05	6.05E-05	6.26E-05	0.000538	0	0.000228	0.000302	0.000878
0	0	0	0	0	0	0	0	0	0	0	0	6.87E-05	0.000289
0.00151	0	0	0.002259	0	5.20E-05	0.000779	3.55E-05	0.000865	0.003749	0	0.000433	0.000241	0.000753
0	0	0	0.00194	0	0	0.000274	3.34E-05	0.00016	0.000469	0	0	0.000231	0.000259
0	0.000159	0.000446	0.009007	0.000362	0.000473	0.000222	0.003211	0.003664	0.004902	0.00022	0.000174	0.001365	0.002607
0.000509	5.48E-05	8.62E-05	0.002263	0	0	0.000434	0.003199	0.001401	0.003665	0	0.000833	0.00082	0.001088
0	0	0.000424	0.000552	0	0	0.000809	2.92E-05	0.000152	0.000196	0	0.000811	0.001924	0.000731
0	0	0.00011	0	0	0	0.000123	0.000236	0.000348	5.60E-05	0	0.000747	0.000323	9.27E-05
0	0.000332	6.03E-05	0	0.000733	0.000102	0	0.000641	0.000498	0.000454	0	0	0.00033	0
0	0	0	0	0	0	0	0	0	0	0	0	0	0
0	0	0	0	0	0	0	0	0	0	0	0	0	0
0	0	0	0	0	0	0	0	0	0	0	0	0	0
0.00027	0.009653	0.013895	0.013619	0.013117	0.035607	0.010142	0.03296	0.008933	0.031076	0.000709	0.005461	0.014808	0.006465
0.016257	0.122094	0.075613	0.181873	0.189678	0.329143	0.080133	0.271096	0.136918	0.153722	0.028163	0.06443	0.296166	0.140578
0.001776	0.011911	0.007864	0.037457	0.015534	0.036825	0.007237	0.053813	0.027984	0.022565	0.002658	0.005332	0.04593	0.014681
0.001755	0.009058	0.006395	0.001666	0.002599	0.001397	0.003991	0.001953	0.005068	0.001828	0.001416	0.003672	0.000868	0.002759
0.040676	0.20452	0.128956	0.279588	0.107596	0.03501	0.096079	0.171019	0.215592	0.156775	0.037272	0.095535	0.038346	0.200845
0.002309	0.022134	0.008952	0.016825	0.005304	0.002671	0.005646	0.015012	0.014471	0.010498	0.002926	0.00844	0.002776	0.01533
0.007391	0.005631	0.025591	0.011818	0.007996	0.002582	0.005207	0.006433	0.008987	0.005462	0.012815	0.001844	0.001214	0.015292
0.000523	0.002514	0.005833	0.000516	0.00243	0.001217	0.003917	0.002326	0.004646	0.002568	0.003325	0.003058	0.000144	0.002792
0.006872	0.007847	0.006718	0.011347	0.030454	0.00105	0.005281	3.55E-05	0.004086	0.0011	0.00149	0.028435	0.001026	0.005025
0.048231	0.106813	0.101554	0.020749	0.045485	0.043322	0.002891	0.059323	0.072686	0.135531	0.011946	0.065777	0.014343	0.144742
0.000458	0.016475	0.012465	0.161618	0.061144	3.71E-05	0.029157	0.000463	0.004151	0.000706	0.000796	0.001292	0.001143	0.021127
0.007742	0.006234	0.003931	0.002016	0.00718	0.000356	0.002282	0.003724	0.002054	0.01466	0.002549	0.003805	0.001448	0.003365
0.005724	0.001534	0.006727	0.000647	0.005272	0.002849	0.002225	0.001258	0.003302	0.004514	0.006352	0.005214	0.000605	0.009634
0.000748	0.016186	0.010664	0.000148	0.019974	0.012438	0.008934	0.000307	0.000272	0	0.031219	0.006137	0.00347	0.014264
0	0.004203	0.019605	0	0.024843	0.017882	0.011832	0	0.000258	0	0.016832	0.003493	0.000247	0.006683
0.000724	0.012201	0.057091	0.003781	0.000976	0.007444	0.043914	0	0.002677	0	0.002124	0.00967	0.00219	0.001194
0.000266	0.010741	0.011114	0.004706	0.008082	0.003723	0.00608	0.000173	0.000903	0.001041	7.91E-05	0.00533	0.003111	0.000725
0.001482	0.012761	0.072918	0.014597	0.01716	0.078961	0.010256	0.014318	0.086629	0.013594	0.205312	0.013915	0.002428	0.047113
0	0.002478	0.001958	0.006946	0.00273	0.011024	0.008633	0.000286	0.004401	0.003438	0.00393	0.002567	0.002265	0.001153
0.003449	0.053748	0.065978	0.011912	0.057828	0.12421	0.101585	0.019173	0.112587	0.068401	0.245315	0.11169	0.085274	0.089061
0.001281	0.010292	0.018543	0.001845	0.022295	0.032055	0.053406	0.003691	0.011637	0.019652	0.032274	0.014834	0.00304	0.00914
0.013203	0.109366	0.180301	0.022208	0.055338	0.084492	0.251738	0.011334	0.087024	0.127995	0.2633	0.277644	0.082024	0.105059
0.000533	0.007409	0.017022	0.001931	0.000314	0.015079	0.045863	0.001317	0.004953	0.003022	0.005532	0.056123	0.04489	0.053982
0.001957	0.042961	0.012783	0.036765	0.06102	0.018154	0.023107	0.024977	0.021802	0.040542	0.021894	0.03225	0.041683	0.011158
0.0011	0.046222	0.008532	0.017152	0.020275	0.014701	0.018344	0.006746	0.003544	0.004921	0.040519	0.047279	0.018123	0.001284
0	0.019338	0	0.001235	0.000378	6.68E-05	0.00059	0.000561	0.000381	0.000152	0	0.000921	0.000719	0
0	0.029275	0.002055	0.005402	7.68E-05	0.000132	0.004001	0.01265	0	0.001346	0	0.008335	0	0.000578
0	0.001891	0.000166	0.000382	0.002045	0.001193	0.000683	0.001498	0.000112	0	0	0.000107	0.005481	6.54E-05
0	0.000948	9.26E-05	0.004593	0.011933	0.003393	0.000402	3.96E-05	0.027225	0.00157	0.000116	0.000528	0.001677	8.45E-05
0	0.00357	0.000286	0.0085	0.009917	0.001754	0.000345	0.000309	0.002587	0.002506	0.000102	0.00207	0.000637	0.000444
0.000546	0.008569	0.00089	0.016735	0.080254	7.05E-05	0.000696	0	0.002187	0.000224	0	0.000909	0.000616	0.000327
0	0.001099	0.000536	0.00269	0.005057	0.000388	0.000301	0	0.000696	0.000174	0	0.000814	5.73E-05	9.00E-05
0.000242	0.000402	0.000439	0.000355	0.001204	0.000906	5.67E-05	0.000981	0.000547	0.000538	0	0	0.000268	8.73E-05
0.018859	0.00355	0.001475	0.001747	0.018412	0.004625	0.000925	0.005709	0.004809	0.006024	0.00031	0.000402	0.003211	0.000548
0	0.000304	0.000487	0	0.002087	0.000217	7.65E-05	0.000357	0.00022	0	0	0	0.000241	0.000164
0.044631	0	9.26E-05	0	0.000493	0.00041	0	0	0	0	0.000486	0	0	0
0.307897	0.000595	7.54E-05	0.000355	0.003838	0.003925	0	5.63E-05	0.000106	0.001107	0.003169	0	7.10E-05	6.82E-05
0.105785	0.000168	7.32E-05	0	0.000759	0.00226	5.43E-05	0	3.54E-05	0.000298	0.000933	0	0	0
0.220685	0.000463	0.000198	0	0.002545	0.003335	0	7.72E-05	0	0.000159	0.002872	0.000121	7.33E-05	0
0.12199	4.53E-05	0.000187	0	0.001018	0.001852	0	0	0	5.91E-05	0.001303	0.000112	0	0
0	0.014038	0.002031	0.002892	0.003057	0.00283	0.014368	0.004951	0.002443	0.000146	4.04E-05	0.000745	0.003967	0.000346
0.000437	0.007337	0.018058	0.029999	0.015016	0.003983	0.01436	0	0.000264	0.003308	0.001259	0.00099	0.006095	0.000237
0.000249	0.004347	0.00159	0.003592	7.36E-05	0.006542	0.006156	0.000478	0.002233	0.007231	7.42E-05	0.005935	0.010369	0.00075
0.011933	0.045955	0.08828	0.040775	0.05496	0.043879	0.101752	0.251087	0.099612	0.130613	0.008339	0.093891	0.209107	0.066061
0	0.002573	0.000773	0.002658	0.001159	0.005378	0.014651	0.012064	0.003827	0.006907	3.23E-05	0.007667	0.044244	0

CC01	CC02	CC05	CC06	CC08	CC09	CC12	CC16	CC17	CC20	CC21	CC24	CC25	CC26
0.008117	0.002123	0	0.000219	0.02026	0	2.44E-05	0	0	0.011635	0	0.000915	0.054964	0
0.002869	0	0.000114	0.000423	0.003588	0	0	0	0	0.003909	0	0.000533	0.005057	6.76E-05
0	0	0	0.000241	0.61148	0.155265	0.000789	0.000203	0.015843	0	0.000168	0.021456	0.024816	0.002205
0	0	0	0.000139	0.037037	0.002043	0.001223	0	0.000194	0	0	0.001376	0.003995	0.002481
0.011138	0.001455	0.002652	0.00754	0.035032	0.004999	0.00197	0	0.008058	0.000753	2.99E-05	0.004564	0.026744	0.018558
0.011458	0.00083	0.000997	0.004522	0.003271	0.001415	0	3.30E-05	0.000537	0.000631	0	0.003885	0.036547	0.027223
0.002926	0	0	0	0	0	0.000362	0	0	0	0	0.000573	0.052139	0.001777
0	0	0.000179	0	0	0.001092	0	0	0	0.000407	0	0.001661	0.016705	0.001998
0.002888	0.017023	0.000192	0	0.002532	0.014403	0.000324	0.04923	0.089032	0.201545	0.118692	0.000395	0.001673	0.000566
0	0.000842	0	0	0	0.001082	0	0.001415	0.004386	0.016073	0.003251	7.55E-05	0	4.16E-05
0	0.002005	0	0	0	0.002415	5.51E-05	0.0026	0.004077	0.026751	0.007472	7.77E-05	0	0
0.002643	0.000613	0	0	0	0.000584	0	0.001868	0.003161	0.016398	0.005269	8.21E-05	0	5.33E-05
0	9.44E-05	0.001295	0.014513	0.015617	0.027053	0	0.186924	0.026762	0.000234	0.103898	0.002697	0.021248	0.072156
0.039151	0.110005	0.062055	0.259856	0.016039	0.095798	0.870741	0.569599	0.134596	0.260462	0.328249	0.064247	0.290698	0.790654
0.011251	0.010829	0.004935	0.193836	0.003799	0.011259	0.043375	0.123581	0.020633	0.086278	0.044608	0.007037	0.015509	0.049361
0.002794	0.003213	0	0.000551	0.003377	0.000782	0	0	0.011633	0.010719	0.003807	0.003219	0.003295	4.81E-05
0.757088	0.599498	0.815193	0.453398	0.177377	0.181372	0.000131	0.011084	0.104302	0.215114	0.008138	0.648358	0.150553	0.003783
0.056046	0.061697	0.040395	0.041216	0.013717	0.015253	0	0.000295	0.012188	0.017681	0.001028	0.053311	0.009014	0.000137
0.030921	0.004652	0.011616	0.001541	0.015195	0.000546	0	0.001448	0.102928	0.0051	0.001185	0.037232	0.00437	0.000363
0	0.001313	0.006071	0.00054	0	0.001024	0	2.93E-05	0.006887	0.012887	0.000307	0.003397	0.001527	4.42E-05
0	0.016303	0.00996	0.000883	0	0	6.03E-05	7.51E-05	0.004465	0.000519	0	0.00099	0.001616	5.46E-05
0	0.043558	0.008035	0.011516	0	0.003985	0	0.000597	0.000784	0.004764	0.000943	0.008616	0.001699	0.000202
0	0.00059	0	0.001573	0	0	0	4.76E-05	0.000537	0	0	4.66E-05	0	0
0	0.000362	0	0.002408	0	0	0	0.000104	0.003901	0	0.000762	0.002955	0	0
0	0	0	0.000583	0	0.010848	0	0.000388	0.024799	0.000163	0.000584	0.001652	0	0
0	0	0	5.35E-05	0	0	0	0	0	0	0.000897	0.001641	0.001718	0
0	0	0.022014	0	0	0.00013	0	0	0	0	0.000143	3.77E-05	0	0.0001
0	0	0	0	0	0	0	0.00041	0	0.000855	0.001738	0.000335	0.000821	0
0	0.000354	0	0	0	0	2.82E-05	0.006042	0.000159	0.008286	0	5.77E-05	0.00084	0
0.008268	0.00129	0.000838	0	0.01129	0.002135	0	0.000628	0.010118	0.040859	0.004459	0.001518	0.008168	0.000122
0	0	0	0	0.003693	0.000324	0	2.56E-05	0	0.000611	0.005271	0	0.000808	0
0.013875	7.47E-05	0.002263	0.000161	0.003588	0.010148	0.000122	0.000936	0.001383	0.00909	0.107924	0.000182	0.004765	0.000118
0	6.29E-05	0.000117	0	0	0.001521	0	0.00013	0	0.001333	0.011709	8.66E-05	0.000827	0
0.016536	0.109392	0.000237	0.000102	0.00306	0.411449	2.18E-05	0.011317	0.010902	0.033224	0.072802	0.102238	0.157824	0.004253
0.005531	0	5.84E-05	0	0.006437	0.00184	0	0	0	0.000132	0.006538	0.002715	0.060906	5.46E-05
0	0.010271	0.000354	0	0.007492	0.027628	0	0.002219	0.036625	0.003237	0.006973	0.012197	0.013836	0.000239
0	8.65E-05	0.007739	0	0	0.004656	2.95E-05	0.002474	0.004993	0.000407	0	0.005295	0.006616	0.000107
0	0	0	0	0	0	0	0	0	0.000489	0	0	0	0
0	7.08E-05	0	0	0	0	0	0.000606	0	0	0	4.00E-05	0	0
0	0	0	0	0	0	5.26E-05	0	0	0.000224	0	0	0	0
0	0	0	0	0	0	0	0.003266	0	0	0.000226	0.000238	0	0
0	0.000425	0	0.00099	0	0.000169	0	0.007351	0.003954	0.001944	0	6.88E-05	0	0
0	0	0.000107	0.002986	0	0.000184	0	0.000231	0.02361	0	0	0	0	0
0	0	9.09E-05	0	0	0	0	2.01E-05	0.001022	0	0	0	0	0
0	0	0	0.000123	0.002849	0	0.000181	5.31E-05	0	0	0	0	0	0
0	0	0	0	0	0	0	0	0	0.001252	7.47E-05	0	0	0
0	0	0	0	0	0	2.95E-05	4.58E-05	0.000123	0	0.000143	0	0	0
0	0	0	0	0	0	0	0	0.002122	0	0	0	0	0
0	0.000185	0	0	0	0	0	0.002029	0.129814	0.001171	4.81E-05	0	0	0
0	0	0	0	0	0	0	0.000458	0.027775	0.000417	0	4.88E-05	0	0
0	0.000488	0	0	0	0	0	0.001703	0.124354	0.002463	3.98E-05	0	0	0
0	0	0	0	0	0	0	0.000386	0.018185	0.000753	0	0	0	0
0	0	0	0	0	0	0	0	0	0	0.000772	0	0	0.000203
0	0	0	0	0	0	0	2.75E-05	0	0	0.00012	0.000124	0	0.000186
0	0	0	0	0	0	0	0	0	0	0.009691	0.000284	0	0
0.016499	0.000295	0.002496	8.56E-05	0.003271	0.008438	0.080481	0.010103	0.02516	0.001232	0.114679	0.003505	0.020701	0.022847
0	0	0	0	0	0.000159	0	1.83E-05	0	0	0.027363	3.77E-05	0	0

CC27	CC28	CC29	CC30	CC31	CC33	CC34	CC35	CC36	CC37	CC39	CC42	CC43	CC44
0	0.010422	0.014826	0.005188	0.005367	0.003615	0.001078	0.000982	0	0.016732	0	0.000151	0	0.004636
0	0	0.000577	0.002377	0.000773	0.003172	0.000165	0	2.87E-05	0.000408	0	0	0	0.000433
0.007905	0.015079	0.043231	0.158718	0.11481	0.053996	0.003745	0	0.000155	0.003043	3.38E-05	0.000161	5.64E-05	0.051966
0.000577	0.008987	0.017313	0.012822	0.072535	0.128733	0.002311	0	0	0.005917	0	0.001323	0	0.020369
0.000306	0.008047	0.011224	0.028776	0.00352	0.021803	0.001771	0.000495	9.12E-05	0.021561	0.000104	0.02362	9.54E-05	0.013055
7.37E-05	0.012589	0.010027	0.033818	0.002873	0.028675	0.002334	0.000103	0.000211	0.029276	0	0.018123	0.000173	0.015582
3.60E-05	0.012286	0.001329	0.014612	0.000741	0.003596	0.0032	0	0.000166	0.009863	0	0.000222	0	0.004473
6.06E-05	0.004659	0.000161	0.008676	0.002621	0.010416	0.000703	0	2.70E-05	0.013632	0	0.00358	0	0.004939
2.95E-05	0.002592	0.011977	0.001267	0.001103	0.001316	4.50E-05	0	0.039694	0.003856	0.00021	0.001055	0.194289	0.003962
0	0	0.001691	0	0	0	0	0	0.001654	0	8.68E-05	0.000122	0.007048	0.000358
0	0.000129	0.001114	0	0	0	0	0	0.002817	0	0.000232	0	0.009286	0.000209
0	0.000136	0.0015	0.000176	0	0	0	0	0.002332	0.000433	0.00027	0	0.009226	0
0.07125	0.123972	0.169539	0.005865	0.007235	0.038877	0.074695	0.000242	0.089644	0.118321	0.25385	0.000257	5.64E-05	0.037092
0.70682	0.688617	0.3724	0.464733	0.119075	0.557654	0.277075	0.00399	0.329522	0.487955	0.654735	0.164668	0.669904	0.328247
0.035341	0.04838	0.040617	0.037093	0.00761	0.041346	0.016751	0.003086	0.046562	0.036707	0.05039	0.011885	0.078411	0.031044
0.001416	0.001431	0.00971	0.003361	0.000439	0.000899	0.002667	0.000112	0.001559	0.001656	0.000163	0	0	0.001618
0.014483	0.00027	0.198336	0.130828	0.362372	0.052381	0.156715	0.918994	0.025804	0.104118	0.000603	0.003414	0.001739	0.317393
0.00088	0.000144	0.012896	0.007824	0.022629	0.001511	0.009191	0.068413	0.003535	0.00744	0	0.000693	9.54E-05	0.029095
0.002058	0.000569	0.006289	0.009786	0.002568	0.019106	0.000956	0.000643	0.002893	0.00128	0.003523	0.001572	0.000742	0.001374
0.000775	0.000129	0.00192	0.001163	0.00022	0.00301	0.013929	0.00012	8.11E-05	0.000221	0	0.000236	5.64E-05	0.000233
0.001049	0	0	0.004904	0.000537	0.004006	0.004083	0.001107	0.00217	0.000212	0.000118	0	0	0.000972
0.001101	0	0.020719	0.009465	0.001701	0.00084	0.014932	0.000122	0.003947	0.001183	0.000123	0	0.00016	0.000513
4.75E-05	0	0	0	0	0	0.000473	0	0	0.000198	0	0	0	0.000223
0.000967	0.001611	0.010824	0.001293	0.000248	0.005889	0.005386	0	0.000473	0.00066	0.000207	0	0	0.000489
0.001438	0	0.003005	0.000875	0.00026	0.000449	0.014162	0	0.000255	0.000807	0.000408	0	8.67E-05	0.000471
0.000796	0.000121	0	0.000434	0.00024	0.000808	0.01198	0	0.000135	0.006342	0	0	0	0
0.002634	0.000968	0	0.000116	0	0	0.00741	0	0.000149	0.000892	0	0	0	0
9.50E-05	0	0	0	0	0	0.00455	0	0.001491	0.000176	0	0.00106	0	0
0.000179	0.000149	0.000259	0	0	0	0.010034	0	0.001007	0.004691	0.000136	0	7.37E-05	0.000358
0.003221	0.000261	0.004295	0.001544	0.000501	0.000384	0.042482	0	0.001713	0.003612	0.00462	0.014554	6.94E-05	0.006311
0.007669	0.001167	0.000112	0.00012	0	0	0.005741	0	0.002772	0.001192	0.000169	0.017775	0.000577	0.000101
0.034215	0.022457	0.006108	0.002572	0.001082	0.001283	0.16043	0.000122	0.110204	0.041455	9.17E-05	0.615547	0.012722	0.02456
0.008739	0.003173	0.000308	0.000318	0	0.001179	0.030575	0	0.00932	0.002975	3.86E-05	0.074668	0.003895	0.000594
0.054352	0.005924	0.019253	0.021651	0.195398	0.003928	0.070438	0.000608	0.077193	0.027693	0.0068	0.000615	0.001054	0.03681
0.002346	0.012005	0.000137	0.010171	0	0	9.51E-05	0	0.013427	0.009541	7.12E-05	0	5.20E-05	0
0.011446	0.004468	0.002077	0.001753	0.065421	0	0.006612	0	0.022485	0.007129	0.003427	0	0.001752	0.002213
0.001812	0.002443	0.000586	0.000759	0.005473	0.001629	0.03127	0	0.002954	0.009937	0.00047	0	0	0.009836
0.000197	0	0	0	0	0	0	0	9.12E-05	0	0	0	0	0
0.00172	0	0	0	0.000281	0.001394	6.25E-05	0	0	0	0	0	0	0.000113
0.000831	0	0	0	0.000476	0	0.000268	0	5.91E-05	0	0.000283	0	0.0002	0
0.000352	0.00028	0.000137	0	0	0	0	0	0.000145	0	8.32E-05	0	0	0.000125
0.000557	0	8.80E-05	0.000135	0.000753	0.000391	0.000323	0	0.000674	0.000385	0.000885	0	0	0.000459
6.88E-05	0	0	0	0	0.000886	0	0	9.29E-05	0	8.20E-05	0	0	0
0.000408	0	0	0.000142	0.000216	0.00041	5.25E-05	0	0.00015	0	0.00015	0	0	0
0	0	0	0	0	0	0	0	5.40E-05	0	0	0	0.000134	0
0	0	0	0	0	0	0	0	0	0	0	0	0.000104	0
0	0	0	0	0	0	0	0	2.20E-05	0	0	0	0.000781	0
0	0	0	0	0	0	0	0	0	0	0	0	0	0
3.60E-05	0	0	0	0	0	0	0	0.000542	0	4.10E-05	0	0.002923	0
0	0	0	0	0	0	0	0	0	0	0	0	0.00062	0
0	0	0.000342	0	0	0	0	0	0.000394	0	7.12E-05	0	0.002641	0.000382
0	0	0	0	0	0	0	0	0.000138	0	0	0	5.20E-05	0
0.00109	0	0	0	0	0	0.000108	0	0.00984	0.000439	2.29E-05	0	0	0
0.003206	0	0	0.00015	0	0.000463	0.000225	0	0.00128	0	0	0	0	0
0.004294	0.000231	8.31E-05	0	0	0	0.000135	0	0.003532	0	8.08E-05	0	0	0
0.012596	0.006177	0.004887	0.016518	0.000924	0.005954	0.010604	0.000863	0.113466	0.018062	0.016155	0.044701	0.000924	0.049391
0.000528	0.000127	0.000103	0	0	0	0.00024	0	0.073047	0	0.001266	0	0	0

CC45	CC46	CC48	CC52	CC54	CC55	CC57	CC58	CC60	CC62	CC63	CC64	CC65	CC67
0.000182	0	0	0	0.019393	0.052165	3.73E-05	0.000401	0.000149	0.034177	0.002008	9.30E-06	0.001187	0.004904
0	0	0	0	0.000871	0.027829	0	0	8.70E-05	0	0	0	0	0.000229
0.002313	0.000172	0	0.000487	0.099629	0.000123	7.46E-05	0.001325	0.062727	0.14741	0.012015	3.51E-05	0.021828	0.001391
0.001332	0	0	0	0.012503	0	5.69E-05	0.000382	0.003172	0.023955	0.000323	0.000256	0.015134	0.008405
0.003928	0.000559	0	0	0.022012	0.012997	0.002615	0.015186	0.002669	0.018016	0.001585	0.004134	0.013574	0.020227
0.003579	0	0	0	0.01602	0.012032	0.001219	0.013407	0.002795	0.01607	0.001679	0.002619	0.010374	0.015238
0.002736	0	0	0	0	0	6.28E-05	0.004528	0.001164	0.033505	0.002738	0.000319	0.018974	0.006376
0.001382	0.000195	0	0	0.010012	0.001089	0.000188	0.00228	0.000947	0.005347	0.000302	0.000221	0.001293	0.002818
0	0	0.073007	0.123721	0.000536	0.037965	0.106753	0.001833	0	0.001906	0.000454	2.69E-05	0.008227	0
0	0	0.002038	0.00549	0	0.00373	0.004162	0.000148	0	0.000351	0.000125	1.03E-05	0.00128	5.20E-05
0	0	0.003601	0.006579	0.000274	0.005091	0.006255	0	0	0	0	0	0	0
0	0	0.002827	0.005463	0	0.003432	0.008181	0.000143	0	0	0.000271	0	0	0
0.147746	0.112374	0.000106	0.159983	0.025524	0	0.088107	0.030919	0.004741	0.005166	0.013527	0.005027	0.013067	0.013287
0.618226	0.578514	0.006329	0.421911	0.099545	0.181014	0.492491	0.764221	0.02829	0.158224	0.052648	0.936014	0.484379	0.779373
0.02942	0.186338	0.006474	0.056881	0.014021	0.010562	0.067491	0.075857	0.001933	0.00973	0.004855	0.04611	0.080563	0.039331
0.000428	0.000342	4.92E-05	0.00337	0.005316	0	0.003693	0.00246	0.004538	0.012479	0.006774	9.30E-06	0.012454	0.000156
0.030236	0.002897	0.000809	0.004253	0.519762	0.000714	0.019059	0.014333	0.019122	0.145504	0.018106	0.00117	0.055282	0.082262
0.001578	0	0	0.00044	0.041696	0.000144	0.0015	0.00087	0.001618	0.009038	0.001366	2.69E-05	0.003973	0.00569
0.001006	0.000536	0.148736	0.004712	0.052568	0.05778	0.00105	0.000817	0.000462	0.001154	0.000329	1.96E-05	0.003147	0.000656
0.001787	0	0.000118	0	0.00029	0	0.000218	0.000255	0.000159	0	0.001173	0	0.003613	0.000211
0.002104	0.002215	0	0.00147	0.001134	0	0.005375	0.000406	0.000177	0	0.001006	0	0	9.34E-05
0.000526	0	0	0	0.009833	0.000986	0.000968	0.000262	0.000571	0.004354	0.000777	9.71E-05	0.018441	0.000852
0	0.000186	0	0.000119	0	0	0.00041	0	0	0	0	0	0	0.000366
0.000689	0	0	0.000305	0.02677	0.000123	0.000872	0.001701	0.000155	0.002638	0.000662	2.58E-05	0.000627	0.000456
0.001768	0.001794	0	0.001088	0.005266	0.000144	0.00137	0.000887	0.0004	0	0.000485	2.07E-05	0.000267	0.000108
0.002442	0	6.14E-05	0.000321	0	0	0.000206	0	8.26E-05	0	0.014481	2.07E-05	0.000227	9.87E-05
0.000268	0	0	0.000235	0	0	0	0	9.00E-05	0.000421	0.001507	1.24E-05	0.021734	0.000164
0.00157	0.000166	0.00016	0	0	0	0.001054	0	0.000233	0.001585	0	0.000297	0	4.67E-05
0.000889	0.001256	0	0.000197	0.000251	0.000149	0.008497	0	0.000246	0	0.00291	3.10E-05	0	0
0.003156	0	5.16E-05	0.000371	0.001776	0.119099	0.000255	0.000559	0	0.001926	0.010085	0.000148	0.005374	0.001446
0.01376	0.000711	0.019541	0.002414	0	0.014015	0.006058	0.00297	0.000498	0.000441	0.002607	1.96E-05	0.000373	0.00049
0.047491	0	0.593393	0.003862	0	0.35347	0.000605	0.034018	0.020598	0.033043	0.065654	0.000195	0.003693	0.002714
0.004127	0	0.141131	0.000496	0.000307	0.101072	0.00012	0.000408	0.001644	0.01288	0.008015	5.16E-05	0.00232	0.000971
0.007733	0.003497	0.00032	0.032785	0.009085	0.002445	0.042146	0.006123	0.76374	0.152716	0.662747	0.000392	0.133272	0.003545
0.000604	0	8.36E-05	0.00026	0	0.000123	0	0.002494	0	0.149747	0.011118	0	0.006267	0.000132
0.004189	0.005241	0	0.013536	0.001167	0.000149	0.038642	0.00053	0.062311	0.013141	0.042964	0.000242	0.01052	0.000275
0.011989	0.043492	0	0.001708	0.003211	0.000149	0.014294	0	0.0119	0.003371	0.032676	0.000187	0.000267	3.87E-05
0	0	0	0.002813	0	0	0	0	0	0	0.000162	0	0	0
0	0	0	0.004577	0	0	0.000324	0	0.001544	0	0.000261	1.14E-05	0.000307	0
0.000375	0	0	0.00147	0	0	0	0	0.000159	0	0.005569	3.31E-05	0	0
6.34E-05	0	0	0.002356	0	0.00036	0.001368	0.006796	8.41E-05	0	0.001747	1.24E-05	0	9.61E-05
0.00218	0.009104	0	0.000653	0.000184	0.000216	0.017594	0	8.55E-05	0	0.000136	1.55E-05	0	0
6.02E-05	0.012518	0	0.002904	0	0	0.007236	0.000139	0	0	0	0	0	0
6.97E-05	0.001977	0	0.000598	0	0	0.001198	0	0	0	0	3.31E-05	0	0
0	0.000189	4.67E-05	0.001642	0	0	0	0	0	0	0	0	0	0
0	0	0.000231	0.000468	0	0	0	0	0	0	0	0	0	0
0	0.000862	0.000253	0.000424	0	0.000118	0.000294	0	0	0	0	0	0	0
0	0	0	5.26E-05	0	0	0	0	0	0	0	0	0	0
0	0	0	0.002688	0	0	0.000214	0	0	0	0	0	0	0
0	0	0	0.000952	0	0	4.91E-05	0	0	0	0	0	0	0
0	0.000179	9.09E-05	0.002774	0	0	0.000532	0	0	0	0	0	0	0
0	0	0	4.98E-05	0	0	0	0	0	0	0	0	0	0
0.011251	0	0	0.003948	0	0	0.001808	0.000972	0	0	0	0.000336	0	0.000371
0.003677	0	0	0	0	0	0.001843	0	0	0	0	0	0	0.000113
0.001567	0	0	0.001487	0	0	0.004301	0.006541	0	0.000391	0	4.44E-05	0	0.000272
0.031076	0.034492	0.000497	0.117259	0.001044	0.000714	0.03915	0.005831	0.00091	0.001314	0.014153	0.001744	0.047442	0.00632
0.000496	0.000193	4.67E-05	0.000426	0	0	0	0	0	0	0	2.17E-05	0.00052	0.000424

CC68	CC69	CC70	CC71	CC72	CC73
0.000357	0.004277	0	8.01E-05	0	0
0	0.000251	0	7.01E-05	0.000108	0
0.003757	0.000135	0.001041	0.009629	0.002972	0.00688
0.003098	0.000109	0.000478	0	4.24E-05	0
0.034599	0.003724	0.009748	6.61E-05	0.000392	0.000915
0.026271	0.005755	0.00353	9.21E-05	0.000384	0.001286
0.042836	0.015908	0.000121	0.000162	5.98E-05	0.000573
0.002396	0.00277	0.000193	0	0	0.000139
0	0.00024	0.028019	6.21E-05	0.009381	0.018388
0	0	0.001177	0	0.000501	0.000724
0	0	0.001391	0	0.000764	0.000903
0.000199	0	0.002681	0	0.000912	0.002201
0.008892	0.003091	0	0.057849	0.025579	0.04134
0.719446	0.084429	0.17473	0.083226	0.08139	0.053855
0.036317	0.004266	0.009071	0.00682	0.00735	0.003869
0.000691	0.004805	0	0.000318	0.000837	0.000498
0.024875	0.21564	0.041297	0.068331	0.251845	0.110843
0.001568	0.016086	0.001462	0.003907	0.016365	0.010019
0.003037	0.00039	0.236115	0.002425	0.008685	0.024811
0.000699	9.46E-05	0.006953	0.020229	0.009642	0.000649
0.00135	0.001012	0	6.21E-05	0.000102	0
0	0.001143	0.003758	0	0.001244	0.006156
0	0	0	0	0.003879	0
0.001764	0.000692	0	2.80E-05	0.000392	0.000145
0.000196	0.000561	0	0	0.002511	0
0	0.00158	0	0	0.000249	0
0.000159	0.000531	0	0	0	0
0.001549	0.003254	0.00328	0.001562	0.013305	0.001581
0.000177	0.004164	0	3.00E-05	0.000235	0.000788
0.001872	0.008857	0.051217	0.000242	0.024127	0.021695
0.000654	0.007237	0.004607	0.00015	0.001454	0.00029
0.009996	0.106115	0.113337	0.004671	0.011806	0.016384
0.001554	0.07214	0.025587	0.000663	0.003051	0.001535
0.019799	0.357408	0.247739	0.441018	0.231406	0.231069
0.001364	0.024881	0.000128	0.002561	0.003981	0.113548
0.00067	0.021572	0.007274	0.022478	0.015519	0.014438
0.001006	0.00992	7.13E-05	0	0.001277	0
0	0	0	0	0	0
0	0.000488	0	0	0	0.000104
0	0	0	0	0.000145	0
0	0.000189	0	0.000234	0	0.000643
0	0	0	6.41E-05	0.001103	0
0	0	0.000264	0	5.40E-05	0
0	0	0.00328	0	5.40E-05	0.000191
0	0	0.000143	0	0	0
0	0	0	0	0	0
0	0	0	0	0	0
0	0	0	0	0	0
0	0	0	0	0	0
0	0	0	0	0	0
0	0	0	0	0	0
0	0	0	0	0	0
0.014156	0.000251	0	1.80E-05	0.009746	0.035867
0.001319	0	0	2.00E-05	0	0
0	0	0	0	0	0.021898
0.029994	0.015799	0.021308	0.272909	0.241152	0.255776
0.003381	0.000233	0	2.20E-05	0.015997	0

Supplementary table S6. The cladogram of oropharyngeal microbial structure and their predominant bacteria between CCs and Hs	
Biomaker_names	Logarithm value
d__Bacteria.p__Synergistota.c__Synergistia.o__Synergistales.f__Synergistaceae.g__Fretibacterium	2.632274171
d__Bacteria.p__Proteobacteria.c__Gammaproteobacteria.o__Oceanospirillales.f__Halomonadaceae.g__Halomonas	3.8797074
d__Bacteria.p__Patescibacteria.c__Saccharimonadia.o__Saccharimonadales.f__Saccharimonadales.g__Saccharimonadales	3.406427833
d__Bacteria.p__Actinobacteriota.c__Coriobacteriia.o__Coriobacteriales.f__Atopobiaceae.g__Olsenella	2.162704196
d__Bacteria.p__Firmicutes.c__Negativicutes.o__Veillonellales_Selenomonadales.f__Veillonellaceae.g__Anaeroglobus	2.188659265
d__Bacteria.p__Firmicutes.c__Clostridia.o__Peptostreptococcales_Tissierellales.f__Anaerovoracaceae.g__Anaerovoracaceae_Family_XIII_UCG_001	2.121238445
d__Bacteria.p__Proteobacteria.c__Alphaproteobacteria.o__Caulobacterales.f__Caulobacteraceae	2.050206929
d__Bacteria.p__Bacteroidota.c__Bacteroidia.o__Bacteroidales.f__Bacteroidaceae.g__Bacteroides	3.851388474
d__Bacteria.p__Firmicutes.c__Clostridia.o__Oscillospirales.f__Oscillospiraceae	3.252406272
d__Bacteria.p__Proteobacteria.c__Gammaproteobacteria.o__Cardiobacteriales.f__Cardiobacteriaceae.g__Cardiobacterium	2.689105282
d__Bacteria.p__Proteobacteria.c__Gammaproteobacteria.o__Burkholderiales.f__Alcaligenaceae	2.588068969
d__Bacteria.p__Firmicutes.c__Clostridia.o__Peptostreptococcales_Tissierellales.f__Anaerovoracaceae.g__Mogibacterium	2.075741042
d__Bacteria.p__Patescibacteria.c__Gracilibacteria.o__Gracilibacteria.f__Gracilibacteria	2.665979957
d__Bacteria.p__Actinobacteriota.c__Coriobacteriia.o__Coriobacteriales.f__Atopobiaceae.g__Atopobium	3.386849842
d__Bacteria.p__Firmicutes.c__Bacilli	5.375146458
d__Bacteria.p__Actinobacteriota.c__Coriobacteriia.o__Coriobacteriales	3.420666635
d__Bacteria.p__Campilobacterota.c__Campylobacteria.o__Campylobacterales.f__Campylobacteraceae.g__Campylobacter	3.985310357
d__Bacteria.p__Bacteroidota.c__Bacteroidia.o__Bacteroidales.f__Prevotellaceae.g__Prevotellaceae_unclassified	2.383586207
d__Bacteria.p__Firmicutes.c__Bacilli.o__RF39.f__RF39	2.429683573
d__Bacteria.p__Firmicutes.c__Clostridia.o__Lachnospirales	4.551732436
d__Bacteria.p__Proteobacteria.c__Gammaproteobacteria.o__Xanthomonadales	2.630660894
d__Bacteria.p__Firmicutes.c__Negativicutes.o__Acidaminococcales.f__Acidaminococcaceae.g__Phascolarctobacterium	2.687054354
d__Bacteria.p__Proteobacteria.c__Gammaproteobacteria.o__Burkholderiales.f__Neisseriaceae.g__Alysiella	2.289797616
d__Bacteria.p__Firmicutes.c__Clostridia.o__Clostridia_vadinBB60_group.f__Clostridia_vadinBB60_group	2.672176492
d__Bacteria.p__Firmicutes.c__Clostridia.o__Christensenellales.f__Christensenellaceae.g__Christensenellaceae_R_7_group	2.768921357
d__Bacteria.p__Bacteroidota.c__Bacteroidia.o__Bacteroidales	5.48322266
d__Bacteria.p__Firmicutes.c__Clostridia.o__Peptostreptococcales_Tissierellales.f__Anaerovoracaceae	3.542548659
d__Bacteria.p__Proteobacteria	5.412144028
d__Bacteria.p__Spirochaetota.c__Spirochaetia.o__Spirochaetales.f__Spirochaetaceae	3.428923641
d__Bacteria.p__Campilobacterota.c__Campylobacteria.o__Campylobacterales	4.012730903
d__Bacteria.p__Bacteroidota.c__Bacteroidia.o__Flavobacteriales	4.280226389
d__Bacteria.p__Actinobacteriota.c__Actinobacteria.o__Actinomycetales.f__Actinomycetaceae.g__F0332	3.090757563
d__Bacteria.p__Proteobacteria.c__Gammaproteobacteria.o__Pseudomonadales.f__Moraxellaceae.g__Acinetobacter	3.09095933
d__Bacteria.p__Patescibacteria	4.405890881
d__Bacteria.p__Bacteroidota.c__Bacteroidia.o__Bacteroidales.f__Rikenellaceae.g__Alistipes	2.794984633
d__Bacteria.p__Patescibacteria.c__Saccharimonadia.o__Saccharimonadales.f__Saccharimonadaceae.g__Saccharimonadaceae_unclassified	2.992158534
d__Bacteria.p__Firmicutes.c__Bacilli.o__Lactobacillales.f__Streptococcaceae	5.290103276
d__Bacteria.p__Firmicutes.c__Clostridia.o__Peptostreptococcales_Tissierellales.f__Anaerovoracaceae.g___Eubacterium__brachy_group	2.296711856
d__Bacteria.p__Actinobacteriota.c__Actinobacteria.o__Propionibacteriales	2.473518029
d__Bacteria.p__Proteobacteria.c__Gammaproteobacteria.o__Burkholderiales.f__Comamonadaceae.g__Comamonas	2.354190204
d__Bacteria.p__Bacteroidota.c__Bacteroidia.o__Bacteroidales.f__Tannerellaceae.g__Tannerella	3.430594769
d__Bacteria.p__Patescibacteria.c__Saccharimonadia.o__Saccharimonadales.f__Saccharimonadales	3.406427833
d__Bacteria.p__Spirochaetota.c__Spirochaetia.o__Spirochaetales	3.428923641
d__Bacteria.p__Firmicutes.c__Bacilli.o__Lactobacillales.f__Lactobacillales_unclassified	3.24680622
d__Bacteria.p__Firmicutes.c__Clostridia.o__Oscillospirales.f__Ruminococcaceae.g__Faecalibacterium	3.625751253
d__Bacteria.p__Proteobacteria.c__Gammaproteobacteria.o__Burkholderiales.f__Neisseriaceae	5.234468118
d__Bacteria.p__Spirochaetota.c__Spirochaetia	3.428923641
d__Bacteria.p__Firmicutes.c__Clostridia.o__Peptostreptococcales_Tissierellales	4.017174586
d__Bacteria.p__Proteobacteria.c__Gammaproteobacteria.o__Xanthomonadales.f__Xanthomonadaceae.g__Xanthomonadaceae_unclassified	2.627002939
d__Bacteria.p__Patescibacteria.c__Saccharimonadia.o__Saccharimonadales.f__Saccharimonadaceae.g__Saccharimonadaceae	3.012554541
d__Bacteria.p__Firmicutes.c__Clostridia.o__Lachnospirales.f__Lachnospiraceae.g__Blautia	2.402864785
d__Bacteria.p__Firmicutes.c__Clostridia.o__Oscillospirales.f__Ruminococcaceae.g__Subdoligranulum	2.605006869
d__Bacteria.p__Firmicutes.c__Clostridia.o__Lachnospirales.f__Lachnospiraceae.g__Lachnoclostridium	2.563493709
d__Bacteria.p__Firmicutes.c__Clostridia.o__Peptostreptococcales_Tissierellales.f__Anaerovoracaceae.g___Eubacterium__saphenum_group	2.3649699
d__Bacteria.p__Proteobacteria.c__Gammaproteobacteria.o__Burkholderiales.f__Sutterellaceae	2.571832679
d__Bacteria.p__Proteobacteria.c__Gammaproteobacteria.o__Enterobacterales.f__Yersiniaceae	2.574018947

d__Bacteria.p__Patescibacteria.c__Saccharimonadia.o__Saccharimonadales	4.223034233
d__Bacteria.p__Patescibacteria.c__Gracilibacteria.o__Absconditabacteriales__SR1_.f__Absconditabacteriales__SR1_.g__Absconditabacteriales__SR1_	3.916048306
d__Bacteria.p__Firmicutes.c__Bacilli.o__Lactobacillales.f__P5D1_392.g__P5D1_392	3.424013405
d__Bacteria.p__Bacteroidota.c__Bacteroidia.o__Bacteroidales.f__Rikenellaceae	2.995765846
d__Bacteria.p__Proteobacteria.c__Gammaproteobacteria.o__Pasteurellales.f__Pasteurellaceae.g__Haemophilus	4.713891605
d__Bacteria.p__Bacteroidota.c__Bacteroidia.o__Bacteroidales.f__Prevotellaceae.g__Prevotella	5.226834003
d__Bacteria.p__Proteobacteria.c__Alphaproteobacteria.o__Sphingomonadales.f__Sphingomonadaceae	3.082728092
d__Bacteria.p__Spirochaetota	3.428923641
d__Bacteria.p__Firmicutes.c__Clostridia.o__Lachnospirales.f__Lachnospiraceae.g__Agathobacter	2.64362065
d__Bacteria.p__Proteobacteria.c__Gammaproteobacteria.o__Pasteurellales	4.821942858
d__Bacteria.p__Bacteroidota.c__Bacteroidia	5.510087816
d__Bacteria.p__Proteobacteria.c__Alphaproteobacteria.o__Rhizobiales.f__Rhizobiaceae.g__Rhizobiaceae_unclassified	2.496381824
d__Bacteria.p__Firmicutes.c__Bacilli.o__Erysipelotrichales.f__Erysipelatoclostridiaceae	2.134522973
d__Bacteria.p__Proteobacteria.c__Gammaproteobacteria.o__Burkholderiales.f__Alcaligenaceae.g__Achromobacter	2.372030147
d__Bacteria.p__Firmicutes.c__Negativicutes.o__Acidaminococcales	2.687054354
d__Bacteria.p__Firmicutes.c__Clostridia.o__Lachnospirales.f__Lachnospiraceae.g__Oribacterium	3.665527103
d__Bacteria.p__Fusobacteriota.c__Fusobacteriia.o__Fusobacteriales.f__Fusobacteriaceae	4.828465519
d__Bacteria.p__Actinobacteriota.c__Actinobacteria.o__Corynebacteriales.f__Nocardiaceae.g__Rhodococcus	3.321219311
d__Bacteria.p__Proteobacteria.c__Alphaproteobacteria.o__Sphingomonadales.f__Sphingomonadaceae.g__Sphingomonas	3.073234622
d__Bacteria.p__Bacteroidota.c__Bacteroidia.o__Bacteroidales.f__Tannerellaceae	3.487183521
d__Bacteria.p__Bacteroidota.c__Bacteroidia.o__Sphingobacteriales.f__Lentimicrobiaceae	2.502383531
d__Bacteria.p__Actinobacteriota.c__Actinobacteria.o__Corynebacteriales.f__Corynebacteriaceae	3.473272604
d__Bacteria.p__Synergistota.c__Synergistia.o__Synergistales	2.635259098
d__Bacteria.p__Bacteroidota	5.510087816
d__Bacteria.p__Bacteroidota.c__Bacteroidia.o__Bacteroidales.f__Porphyromonadaceae.g__Porphyromonas	4.699186728
d__Bacteria.p__Bacteroidota.c__Bacteroidia.o__Bacteroidales.f__Prevotellaceae.g__Alloprevotella	4.835033608
d__Bacteria.p__Firmicutes.c__Bacilli.o__Mycoplasmatales.f__Mycoplasmataceae.g__Mycoplasma	2.674949042
d__Bacteria.p__Firmicutes.c__Clostridia.o__Lachnospirales.f__Lachnospiraceae.g__Johnsonella	3.139329677
d__Bacteria.p__Firmicutes.c__Clostridia.o__Lachnospirales.f__Lachnospiraceae.g___Ruminococcus__torques_group	2.264114186
d__Bacteria.p__Synergistota.c__Synergistia.o__Synergistales.f__Synergistaceae	2.635259098
d__Bacteria.p__Proteobacteria.c__Alphaproteobacteria.o__Caulobacterales.f__Caulobacteraceae.g__Brevundimonas	1.891916868
d__Bacteria.p__Synergistota	2.635259098
d__Bacteria.p__Firmicutes.c__Clostridia.o__Lachnospirales.f__Lachnospiraceae.g__Lachnospiraceae_ND3007_group	2.46693069
d__Bacteria.p__Fusobacteriota.c__Fusobacteriia.o__Fusobacteriales.f__Leptotrichiaceae	5.08932294
d__Bacteria.p__Proteobacteria.c__Gammaproteobacteria.o__Enterobacterales.f__Enterobacteriaceae.g__Escherichia_Shigella	2.345665651
d__Bacteria.p__Bacteroidota.c__Bacteroidia.o__Flavobacteriales.f__Flavobacteriaceae.g__Capnocytophaga	4.240812305
d__Bacteria.p__Proteobacteria.c__Gammaproteobacteria.o__Pseudomonadales	4.091970622
d__Bacteria.p__Firmicutes.c__Negativicutes.o__Veillonellales_Selenomonadales.f__Selenomonadaceae.g__Megamonas	2.90995982
d__Bacteria.p__Synergistota.c__Synergistia	2.635259098
d__Bacteria.p__Firmicutes.c__Clostridia.o__Peptococcales	2.539276348
d__Bacteria.p__Bacteroidota.c__Bacteroidia.o__Bacteroidales.f__Porphyromonadaceae	4.699186728
d__Bacteria.p__Bacteroidota.c__Bacteroidia.o__Flavobacteriales.f__Flavobacteriaceae	4.240990063
d__Bacteria.p__Desulfobacterota	2.651030435
d__Bacteria.p__Firmicutes.c__Clostridia.o__Oscillospirales.f__Oscillospiraceae.g__UCG_002	2.666358636
d__Bacteria.p__Firmicutes.c__Clostridia.o__Oscillospirales.f___Eubacterium__coprostanoligenes_group	2.613886785
d__Bacteria.p__Bacteroidota.c__Bacteroidia.o__Bacteroidales.f__Paludibacteraceae	2.847946573
d__Bacteria.p__Bacteroidota.c__Bacteroidia.o__Bacteroidales.f__Tannerellaceae.g__Parabacteroides	2.574142137
d__Bacteria.p__Actinobacteriota.c__Actinobacteria.o__Propionibacteriales.f__Propionibacteriaceae	2.473518029
d__Bacteria.p__Firmicutes.c__Clostridia.o__Oscillospirales.f__Ruminococcaceae	3.757072013
d__Bacteria.p__Proteobacteria.c__Alphaproteobacteria.o__Sphingomonadales	3.082728092
d__Bacteria.p__Firmicutes.c__Clostridia.o__Peptostreptococcales_Tissierellales.f__Peptostreptococcaceae.g___Eubacterium__yurii_group	2.326923879
d__Bacteria.p__Proteobacteria.c__Gammaproteobacteria.o__Burkholderiales.f__Sutterellaceae.g__Parasutterella	2.306675926
d__Bacteria.p__Firmicutes.c__Clostridia.o__Lachnospirales.f__Lachnospiraceae.g__Stomatobaculum	3.58847435
d__Bacteria.p__Bacteroidota.c__Bacteroidia.o__Flavobacteriales.f__Weeksellaceae	3.216659936
d__Bacteria.p__Campilobacterota.c__Campylobacteria.o__Campylobacterales.f__Campylobacteraceae	3.985310357
d__Bacteria.p__Proteobacteria.c__Gammaproteobacteria.o__Cardiobacteriales	2.689105282
d__Bacteria.p__Firmicutes.c__Bacilli.o__Erysipelotrichales	3.611470199
d__Bacteria.p__Proteobacteria.c__Gammaproteobacteria.o__Pasteurellales.f__Pasteurellaceae.g__Aggregatibacter	3.635251608

d__Bacteria.p__Fusobacteriota.c__Fusobacteriia.o__Fusobacteriales.f__Fusobacteriaceae.g__Fusobacterium	4.828465519
d__Bacteria.p__Patescibacteria.c__Gracilibacteria	3.941992212
d__Bacteria.p__Campilobacterota.c__Campylobacteria	4.012730903
d__Bacteria.p__Firmicutes.c__Clostridia.o__Lachnospirales.f__Lachnospiraceae.g__Fusicatenibacter	3.288705767
d__Bacteria.p__Actinobacteriota.c__Coriobacteriia.o__Coriobacteriales.f__Atopobiaceae	3.412026018
d__Bacteria.p__Bacteroidota.c__Bacteroidia.o__Bacteroidales.f__Prevotellaceae	5.376521861
d__Bacteria.p__Actinobacteriota.c__Actinobacteria.o__Bifidobacteriales.f__Bifidobacteriaceae	2.989568146
d__Bacteria.p__Bacteroidota.c__Bacteroidia.o__Flavobacteriales.f__Weeksellaceae.g__Bergeyella	3.164143949
d__Bacteria.p__Firmicutes.c__Bacilli.o__Lactobacillales	5.32602313
d__Bacteria.p__Firmicutes.c__Clostridia.o__Clostridia_vadinBB60_group	2.672176492
d__Bacteria.p__Proteobacteria.c__Gammaproteobacteria.o__Pasteurellales.f__Pasteurellaceae	4.821942858
d__Bacteria.p__Proteobacteria.c__Gammaproteobacteria.o__Enterobacterales.f__Yersiniaceae.g__Serratia	2.574018947
d__Bacteria.p__Firmicutes.c__Clostridia.o__Oscillospirales.f___Eubacterium__coprostanoligenes_group.g___Eubacterium__coprostanoligenes_group	2.613886785
d__Bacteria.p__Patescibacteria.c__Saccharimonadia	4.223034233
d__Bacteria.p__Actinobacteriota.c__Actinobacteria.o__Corynebacteriales.f__Corynebacteriaceae.g__Corynebacterium	3.45764358
d__Bacteria.p__Actinobacteriota.c__Actinobacteria.o__Bifidobacteriales	2.989568146
d__Bacteria.p__Firmicutes.c__Bacilli.o__Erysipelotrichales.f__Erysipelotrichaceae	3.59674094
d__Bacteria.p__Firmicutes.c__Bacilli.o__RF39	2.429683573
d__Bacteria.p__Proteobacteria.c__Gammaproteobacteria.o__Pseudomonadales.f__Pseudomonadaceae	3.772791273
d__Bacteria.p__Firmicutes.c__Bacilli.o__Lactobacillales.f__Lactobacillales_unclassified.g__Lactobacillales_unclassified	3.24680622
d__Bacteria.p__Firmicutes.c__Bacilli.o__Lactobacillales.f__Streptococcaceae.g__Streptococcus	5.290103276
d__Bacteria.p__Proteobacteria.c__Gammaproteobacteria.o__Burkholderiales.f__Neisseriaceae.g__Neisseria	5.229838061
d__Bacteria.p__Actinobacteriota.c__Actinobacteria.o__Bifidobacteriales.f__Bifidobacteriaceae.g__Bifidobacterium	2.889980781
d__Bacteria.p__Patescibacteria.c__Gracilibacteria.o__Absconditabacteriales__SR1_	3.916048306
d__Bacteria.p__Proteobacteria.c__Gammaproteobacteria.o__Cardiobacteriales.f__Cardiobacteriaceae	2.689105282
d__Bacteria.p__Firmicutes.c__Clostridia.o__Christensenellales	2.768921357
d__Bacteria.p__Firmicutes.c__Clostridia.o__Peptostreptococcales_Tissierellales.f__Peptostreptococcaceae.g__Peptostreptococcaceae_unclassified	2.027306224
d__Bacteria.p__Firmicutes.c__Clostridia.o__Lachnospirales.f__Lachnospiraceae.g__Butyrivibrio	2.611833903
d__Bacteria.p__Firmicutes.c__Clostridia.o__Clostridia_vadinBB60_group.f__Clostridia_vadinBB60_group.g__Clostridia_vadinBB60_group	2.672176492
d__Bacteria.p__Firmicutes.c__Clostridia.o__Peptostreptococcales_Tissierellales.f__Peptostreptococcales_Tissierellales	3.290086733
d__Bacteria.p__Proteobacteria.c__Gammaproteobacteria.o__Burkholderiales.f__Comamonadaceae.g__Acidovorax	2.480664282
d__Bacteria.p__Firmicutes.c__Clostridia.o__Clostridia_UCG_014	3.575291049
d__Bacteria.p__Proteobacteria.c__Gammaproteobacteria.o__Burkholderiales	5.247072604
d__Bacteria.p__Firmicutes.c__Clostridia.o__Lachnospirales.f__Lachnospiraceae.g__Roseburia	2.559741133
d__Bacteria.p__Firmicutes.c__Clostridia.o__Peptostreptococcales_Tissierellales.f__Anaerovoracaceae.g___Eubacterium__nodatum_group	3.395359161
d__Bacteria.p__Firmicutes.c__Bacilli.o__Mycoplasmatales.f__Mycoplasmataceae	2.674949042
d__Bacteria.p__Patescibacteria.c__Gracilibacteria.o__Gracilibacteria	2.665979957
d__Bacteria.p__Proteobacteria.c__Gammaproteobacteria.o__Pseudomonadales.f__Moraxellaceae	3.808361838
d__Bacteria.p__Spirochaetota.c__Spirochaetia.o__Spirochaetales.f__Spirochaetaceae.g__Treponema	3.42817789
d__Bacteria.p__Proteobacteria.c__Gammaproteobacteria.o__Oceanospirillales	3.8797074
d__Bacteria.p__Firmicutes.c__Clostridia.o__Peptococcales.f__Peptococcaceae.g__Peptococcus	2.512409029
d__Bacteria.p__Firmicutes.c__Clostridia.o__Lachnospirales.f__Lachnospiraceae.g__Lachnospira	2.629583091
d__Bacteria.p__Firmicutes.c__Bacilli.o__Lactobacillales.f__P5D1_392	3.424013405
d__Bacteria.p__Patescibacteria.c__Gracilibacteria.o__Absconditabacteriales__SR1_.f__Absconditabacteriales__SR1_	3.916048306
d__Bacteria.p__Firmicutes	5.570850845
d__Bacteria.p__Firmicutes.c__Bacilli.o__Erysipelotrichales.f__Erysipelotrichaceae.g__Solobacterium	3.586966566
d__Bacteria.p__Firmicutes.c__Clostridia.o__Peptococcales.f__Peptococcaceae	2.539276348
d__Bacteria.p__Firmicutes.c__Clostridia.o__Peptostreptococcales_Tissierellales.f__Peptostreptococcaceae	3.695951821
d__Bacteria.p__Bacteroidota.c__Bacteroidia.o__Bacteroidales.f__Muribaculaceae	3.613542702
d__Bacteria.p__Proteobacteria.c__Gammaproteobacteria.o__Burkholderiales.f__Alcaligenaceae.g__Alcaligenes	2.181265684
d__Bacteria.p__Firmicutes.c__Bacilli.o__Lactobacillales.f__Carnobacteriaceae	3.936871737
d__Bacteria.p__Proteobacteria.c__Gammaproteobacteria.o__Burkholderiales.f__Comamonadaceae	2.832033048
d__Bacteria.p__Patescibacteria.c__Saccharimonadia.o__Saccharimonadales.f__Saccharimonadaceae.g__Candidatus_Saccharimonas	3.127797269
d__Bacteria.p__Firmicutes.c__Clostridia.o__Oscillospirales	3.906187699
d__Bacteria.p__Patescibacteria.c__Saccharimonadia.o__Saccharimonadales.f__Saccharimonadaceae.g__TM7x	4.033439584
d__Bacteria.p__Proteobacteria.c__Alphaproteobacteria.o__Caulobacterales	2.050206929
d__Bacteria.p__Firmicutes.c__Clostridia.o__Clostridia_UCG_014.f__Clostridia_UCG_014	3.575291049
d__Bacteria.p__Firmicutes.c__Bacilli.o__Lactobacillales.f__Carnobacteriaceae.g__Granulicatella	3.936751392

d__Bacteria.p__Proteobacteria.c__Gammaproteobacteria.o__Enterobacterales	2.837460828
d__Bacteria.p__Firmicutes.c__Negativicutes.o__Veillonellales_Selenomonadales.f__Veillonellaceae.g__Megasphaera	3.670033551
d__Bacteria.p__Firmicutes.c__Clostridia.o__Lachnospirales.f__Lachnospiraceae.g__Catonella	3.125647352
d__Bacteria.p__Bacteroidota.c__Bacteroidia.o__Bacteroidales.f__Paludibacteraceae.g__F0058	2.847946573
d__Bacteria.p__Actinobacteriota.c__Coriobacteriia	3.420666635
d__Bacteria.p__Proteobacteria.c__Gammaproteobacteria.o__Enterobacterales.f__Enterobacteriaceae	2.49527856
d__Bacteria.p__Firmicutes.c__Clostridia.o__Lachnospirales.f__Defluviitaleaceae.g__Defluviitaleaceae_UCG_011	2.28192745
d__Bacteria.p__Firmicutes.c__Clostridia	4.77368952
d__Bacteria.p__Proteobacteria.c__Gammaproteobacteria.o__Xanthomonadales.f__Xanthomonadaceae	2.630660894
d__Bacteria.p__Patescibacteria.c__Saccharimonadia.o__Saccharimonadales.f__Saccharimonadaceae	4.150878212
d__Bacteria.p__Firmicutes.c__Clostridia.o__Oscillospirales.f__Ruminococcaceae.g__Ruminococcus	2.639613631
d__Bacteria.p__Proteobacteria.c__Gammaproteobacteria.o__Burkholderiales.f__Burkholderiales_unclassified	3.12341604
d__Bacteria.p__Firmicutes.c__Bacilli.o__Mycoplasmatales	2.674949042
d__Bacteria.p__Proteobacteria.c__Alphaproteobacteria.o__Rhizobiales.f__Rhizobiaceae	2.52695818
d__Bacteria.p__Firmicutes.c__Bacilli.o__RF39.f__RF39.g__RF39	2.429683573
d__Bacteria.p__Bacteroidota.c__Bacteroidia.o__Bacteroidales.f__Prevotellaceae.g__Prevotellaceae_uncultured	1.9098518
d__Bacteria.p__Firmicutes.c__Clostridia.o__Lachnospirales.f__Defluviitaleaceae	2.28192745
d__Bacteria.p__Actinobacteriota.c__Actinobacteria.o__Corynebacteriales.f__Nocardiaceae	3.321219311
d__Bacteria.p__Firmicutes.c__Clostridia.o__Lachnospirales.f__Lachnospiraceae.g___Eubacterium__eligens_group	2.681000538
d__Bacteria.p__Bacteroidota.c__Bacteroidia.o__Bacteroidales.f__Rikenellaceae.g__Rikenellaceae_RC9_gut_group	2.50970287
d__Bacteria.p__Bacteroidota.c__Bacteroidia.o__Bacteroidales.f__Muribaculaceae.g__Muribaculaceae	3.609873572
d__Bacteria.p__Proteobacteria.c__Alphaproteobacteria.o__Rhizobiales	2.937047486
d__Bacteria.p__Campilobacterota	4.012730903
d__Bacteria.p__Firmicutes.c__Clostridia.o__Lachnospirales.f__Lachnospiraceae.g__Lachnospiraceae_uncultured	3.553281216
d__Bacteria.p__Actinobacteriota.c__Actinobacteria.o__Corynebacteriales	3.709854306
d__Bacteria.p__Firmicutes.c__Clostridia.o__Peptostreptococcales_Tissierellales.f__Peptostreptococcaceae.g__Filifactor	3.169008096
d__Bacteria.p__Firmicutes.c__Clostridia.o__Lachnospirales.f__Lachnospiraceae.g__Lachnospiraceae_NK4A136_group	3.270009451
d__Bacteria.p__Bacteroidota.c__Bacteroidia.o__Sphingobacteriales.f__Lentimicrobiaceae.g__Lentimicrobium	2.502383531
d__Bacteria.p__Firmicutes.c__Clostridia.o__Peptostreptococcales_Tissierellales.f__Peptostreptococcales_Tissierellales.g__Parvimonas	3.272278695
d__Bacteria.p__Proteobacteria.c__Gammaproteobacteria.o__Pasteurellales.f__Pasteurellaceae.g__Actinobacillus	4.004757553
d__Bacteria.p__Firmicutes.c__Clostridia.o__Lachnospirales.f__Lachnospiraceae.g___Eubacterium__ruminantium_group	2.853536412
d__Bacteria.p__Proteobacteria.c__Gammaproteobacteria.o__Oceanospirillales.f__Halomonadaceae	3.8797074
d__Bacteria.p__Bacteroidota.c__Bacteroidia.o__Bacteroidales.f__Bacteroidaceae	3.851388474
d__Bacteria.p__Proteobacteria.c__Gammaproteobacteria.o__Pseudomonadales.f__Pseudomonadaceae.g__Pseudomonas	3.772791273
d__Bacteria.p__Firmicutes.c__Clostridia.o__Lachnospirales.f__Lachnospiraceae.g__Lachnospiraceae_unclassified	3.473733826
d__Bacteria.p__Patescibacteria.c__Gracilibacteria.o__Gracilibacteria.f__Gracilibacteria.g__Gracilibacteria	2.665979957
d__Bacteria.p__Fusobacteriota.c__Fusobacteriia.o__Fusobacteriales.f__Leptotrichiaceae.g__Leptotrichia	5.089214723
d__Bacteria.p__Bacteroidota.c__Bacteroidia.o__Sphingobacteriales	2.521763858
d__Bacteria.p__Firmicutes.c__Clostridia.o__Christensenellales.f__Christensenellaceae	2.768921357
d__Bacteria.p__Firmicutes.c__Clostridia.o__Peptostreptococcales_Tissierellales.f__Anaerovoracaceae.g__Amnipila	2.593604175
d__Bacteria.p__Firmicutes.c__Negativicutes.o__Veillonellales_Selenomonadales.f__Veillonellaceae.g__Dialister	3.107195531
d__Bacteria.p__Proteobacteria.c__Gammaproteobacteria.o__Burkholderiales.f__Burkholderiales_unclassified.g__Burkholderiales_unclassified	3.12341604
d__Bacteria.p__Firmicutes.c__Negativicutes.o__Acidaminococcales.f__Acidaminococcaceae	2.687054354
d__Bacteria.p__Firmicutes.c__Clostridia.o__Clostridia_UCG_014.f__Clostridia_UCG_014.g__Clostridia_UCG_014	3.575291049
d__Bacteria.p__Firmicutes.c__Clostridia.o__Lachnospirales.f__Lachnospiraceae	4.549392799
d__Bacteria.p__Proteobacteria.c__Gammaproteobacteria	5.409524578

Groups	LDA_value	P_value
H	2.544195561	5.86E-08
CC	3.62219215	5.68E-05
H	3.058205805	8.97E-10
H	3.333556801	1.03E-06
H	3.310887846	2.28E-05
H	3.628776555	0.030672536
H	3.213266949	1.07E-06
H	3.454299106	7.38E-11
H	2.954745837	2.24E-09
CC	2.73851522	0.000459512
H	2.716438412	3.40E-16
H	3.789740044	1.36E-05
H	2.820651468	3.80E-07
H	2.672273405	0.001548273
CC	4.882008611	0.015364351
H	2.705730019	0.000454646
H	3.258550038	2.52E-08
H	3.18734884	1.32E-09
H	2.837450031	5.12E-12
H	3.790152613	9.09E-06
CC	3.118007819	0.003923325
H	2.578202657	4.91E-06
H	3.356073553	2.49E-09
H	2.830689641	5.63E-15
H	2.661358308	4.20E-07
H	4.882860876	4.88E-10
H	2.729638418	0.000473422
H	4.536871524	0.000500929
H	2.888313052	1.16E-08
H	3.308841817	1.37E-08
H	3.567880722	0.000201239
CC	2.881590757	0.000397763
H	2.836493974	6.55E-11
H	3.872165377	5.29E-10
H	2.704047237	3.12E-05
H	2.871360001	5.02E-06
CC	4.81369307	0.048740679
H	3.625716289	6.73E-10
H	2.670087398	0.001078223
H	3.194897835	2.81E-06
H	2.726296803	6.09E-05
H	3.054895289	8.97E-10
H	2.888313052	1.16E-08
CC	2.736866641	0.026295925
H	3.251538362	1.81E-09
H	4.288894245	0.000857202
H	2.888313052	1.16E-08
H	3.120578555	0.000263827
CC	3.086019822	5.55E-15
H	2.597258712	0.00237272
H	2.76316896	7.18E-06
H	2.56135264	0.000142744
H	2.755515167	2.19E-05
H	3.193651774	1.59E-05
H	2.609215303	6.23E-07
H	2.891704774	5.69E-06

H	3.544014821	6.05E-07
H	3.595724573	8.65E-15
CC	3.092632107	1.10E-08
H	2.784424053	3.60E-12
H	4.211344374	4.16E-11
H	4.422466795	5.13E-05
H	2.667865243	7.05E-06
H	2.888313052	1.16E-08
H	2.536754336	0.000426367
H	4.34659401	9.94E-12
H	4.902962913	8.08E-11
CC	2.9904862	1.32E-15
H	3.092811926	6.10E-06
H	2.902582031	1.41E-14
H	2.575609057	4.91E-06
H	2.87925204	0.000547084
H	4.130065522	8.55E-09
H	2.988942843	1.50E-05
H	2.779680807	2.59E-07
H	2.788887573	1.13E-05
H	2.938498532	7.23E-12
H	2.887043344	6.40E-07
H	2.520516855	3.09E-07
H	4.902962913	8.08E-11
H	4.062534396	8.77E-09
H	4.528413932	6.23E-22
H	2.909726627	1.16E-07
H	2.686971723	0.003008185
H	3.037193131	1.83E-06
H	2.517565241	3.09E-07
H	3.509268948	3.32E-06
H	2.513054847	3.09E-07
H	2.972462541	5.94E-07
CC	4.55429267	0.000117568
H	2.627404946	1.69E-05
H	3.513865391	0.000844008
H	3.774059618	1.35E-19
H	2.704818322	2.12E-07
H	2.524963632	3.09E-07
H	3.271147419	3.78E-15
H	4.062534396	8.77E-09
H	3.514285968	0.000793093
H	2.778450186	8.35E-07
H	2.56309021	1.25E-06
H	2.614228064	3.90E-06
H	2.530809476	6.59E-05
H	2.599692515	8.04E-06
H	2.733335271	0.001078223
H	3.365654982	3.63E-09
H	2.667712409	7.05E-06
H	3.432696887	0.002519575
H	2.800118826	1.89E-06
H	2.865673096	0.003503316
H	2.912028414	6.62E-11
H	3.258550038	2.52E-08
CC	2.669246192	0.000319886
H	2.941219314	1.30E-07
H	3.313474492	5.67E-15

H	4.130065522	8.55E-09
H	3.622295557	2.18E-15
H	3.308841817	1.37E-08
H	2.983979591	2.18E-10
H	2.70765682	0.000799227
H	4.770508277	6.45E-09
H	2.669972992	0.001387025
H	2.851108484	1.35E-09
CC	4.83958285	0.015364351
H	2.830241018	5.63E-15
H	4.34659401	9.94E-12
H	2.87879635	5.69E-06
H	2.608770215	3.90E-06
H	3.544014821	6.05E-07
H	2.884298581	3.61E-06
H	2.671781021	0.001387025
H	2.91197237	5.06E-07
H	2.828774259	5.12E-12
H	3.452193577	1.03E-19
CC	2.765089044	0.026295925
CC	4.813714788	0.048740679
H	4.307161799	0.000367762
H	2.641660344	6.34E-06
H	3.595724573	8.65E-15
CC	2.669246192	0.000319886
H	2.665608265	4.20E-07
H	3.414616455	4.44E-05
CC	3.045200027	5.08E-05
H	2.83107319	5.63E-15
H	2.958960304	2.62E-11
H	2.892408778	3.81E-05
H	2.865246069	1.03E-07
H	4.241418575	0.003361795
H	2.903664989	1.26E-09
CC	2.692755743	0.007693372
H	2.916430855	1.16E-07
H	2.818272031	3.80E-07
H	3.497761469	7.72E-13
H	2.892759218	5.57E-09
CC	3.62219215	5.68E-05
H	3.208661674	4.59E-13
H	2.514573359	3.02E-05
CC	3.092632107	1.10E-08
H	3.595724573	8.65E-15
CC	4.878685629	0.000131777
H	2.89467189	1.07E-06
H	3.24866255	3.78E-15
H	2.867650744	0.002466342
H	3.34179421	7.24E-10
H	3.194547814	1.18E-10
CC	3.069973981	0.034585397
H	2.663628627	5.38E-07
H	3.045584342	1.79E-11
H	3.529162819	1.78E-10
H	3.291341281	4.19E-06
H	3.232293068	0.010065566
H	2.865246069	1.03E-07
CC	3.06978808	0.03385413

H	2.562745625	1.09E-07
H	3.158989106	0.002006267
H	2.88705667	5.30E-06
H	2.530809476	6.59E-05
H	2.705730019	0.000454646
H	2.703460695	4.43E-06
H	3.189777449	4.81E-06
H	4.066526893	9.84E-07
CC	3.1163832	0.003923325
H	3.409819445	4.85E-06
H	2.529452988	0.000203744
CC	2.935619565	0.001281256
H	2.905198728	1.16E-07
CC	2.984033146	1.32E-15
H	2.811020189	5.12E-12
H	3.639180776	0.001309513
H	3.180550787	4.81E-06
H	2.989237283	1.50E-05
H	2.504748066	5.26E-05
H	3.161003754	3.10E-09
H	3.338016943	7.24E-10
CC	2.885570405	3.60E-07
H	3.308841817	1.37E-08
H	3.148496938	4.05E-06
H	3.088412139	1.78E-07
H	2.97928156	1.80E-08
H	3.018367927	9.53E-08
H	2.941886565	7.23E-12
H	2.950764496	6.87E-11
H	3.609263759	1.47E-11
H	2.702657753	4.28E-09
CC	3.62219215	5.68E-05
H	3.453972093	7.38E-11
H	3.452877735	1.03E-19
H	3.180500264	5.41E-07
H	2.810752448	3.80E-07
CC	4.560091457	7.53E-05
H	2.846115772	4.72E-12
H	2.661018746	4.20E-07
H	2.920589876	0.000799992
H	2.679523278	1.29E-08
CC	2.928104398	0.001281256
H	2.577910193	4.91E-06
H	2.865246069	1.03E-07
H	3.785757013	1.01E-05
H	4.53772341	0.000517301

Supplementary table S7. The corresponding LDA value and p value of microbial community gene function for samples in the discovery cohort				
Biomaker_names	Logarithm value	Groups	LDA_value	P_value
L1_Metabolism.L2_Glycan_biosynthesis_and_metabolism.L3_Glycosylphosphatidylinositol_GPI_anchor_biosynthesis	0			-
L1_Metabolism.L2_Lipid_metabolism.L3_Sphingolipid_metabolism	3.424984742			-
L1_Metabolism.L2_Metabolism_of_cofactors_and_vitamins.L3_Riboflavin_metabolism	4.048603752			-
L1_Metabolism.L2_Nucleotide_metabolism.L3_Purine_metabolism	3.994368667			-
L1_Metabolism.L2_Amino_acid_metabolism.L3_Cysteine_and_methionine_metabolism	4.141796991			-
L1_Metabolism.L2_Lipid_metabolism.L3_Fatty_acid_degradation	3.577130545			-
L1_Environmental_Information_Processing.L2_Membrane_transport.L3_Bacterial_secretion_system	4.026748259			-
L1_Metabolism.L2_Metabolism_of_cofactors_and_vitamins.L3_Porphyrin_and_chlorophyll_metabolism	3.900534524			-
L1_Metabolism.L2_Biosynthesis_of_other_secondary_metabolites.L3_Penicillin_and_cephalosporin_biosynthesis	2.736786849			-
L1_Metabolism.L2_Xenobiotics_biodegradation_and_metabolism.L3_Drug_metabolism_other_enzymes	4.111133221			-
L1_Cellular_Processes.L2_Cell_growth_and_death.L3_Apoptosis	2.761205409			-
L1_Metabolism.L2_Metabolism_of_terpenoids_and_polyketides.L3_Biosynthesis_of_siderophore_group_nonribosomal_peptides	2.9341534			-
L1_Metabolism.L2_Amino_acid_metabolism.L3_Histidine_metabolism	3.979603121			-
L1_Metabolism.L2_Amino_acid_metabolism.L3_Valine_leucine_and_isoleucine_degradation	3.600547785			-
L1_Metabolism.L2_Metabolism_of_terpenoids_and_polyketides.L3_Limonene_and_pinene_degradation	2.861655126			-
L1_Metabolism.L2_Amino_acid_metabolism.L3_Glycine_serine_and_threonine_metabolism	4.054501045			-
L1_Metabolism.L2_Metabolism_of_terpenoids_and_polyketides.L3_Terpenoid_backbone_biosynthesis	4.151101569			-
L1_Metabolism.L2_Glycan_biosynthesis_and_metabolism.L3_N_Glycan_biosynthesis	2.842134376			-
L1_Metabolism.L2_Carbohydrate_metabolism.L3_Propanoate_metabolism	3.829394769			-
L1_Metabolism.L2_Lipid_metabolism.L3_Biosynthesis_of_unsaturated_fatty_acids	3.540351519			-
L1_Metabolism.L2_Biosynthesis_of_other_secondary_metabolites.L3_Betalain_biosynthesis	1.53208283			-
L1_Genetic_Information_Processing.L2_Translation.L3_Aminoacyl_tRNA_biosynthesis	4.274631072			-
L1_Cellular_Processes.L2_Cell_growth_and_death.L3_Cell_cycle_Caulobacter	4.224830759			-
L1_Metabolism.L2_Xenobiotics_biodegradation_and_metabolism.L3_Xylene_degradation	2.467195301			-
L1_Metabolism.L2_Xenobiotics_biodegradation_and_metabolism.L3_Chlorocyclohexane_and_chlorobenzene_degradation	2.414770755			-
L1_Metabolism.L2_Metabolism_of_cofactors_and_vitamins.L3_Nicotinate_and_nicotinamide_metabolism	4.095193168			-
L1_Metabolism.L2_Metabolism_of_other_amino_acids.L3_D_Alanine_metabolism	4.298644744	H	2.809955	0.0028
L1_Metabolism.L2_Xenobiotics_biodegradation_and_metabolism.L3_Nitrotoluene_degradation	3.271989313			-
L1_Metabolism.L2_Lipid_metabolism.L3_Steroid_hormone_biosynthesis	2.25215489			-
L1_Metabolism.L2_Xenobiotics_biodegradation_and_metabolism.L3_Caprolactam_degradation	2.507657618			-
L1_Metabolism.L2_Metabolism_of_terpenoids_and_polyketides.L3_Biosynthesis_of_type_II_polyketide_products	0			-
L1_Metabolism.L2_Metabolism_of_other_amino_acids.L3_Taurine_and_hypotaurine_metabolism	3.888381097			-
L1_Cellular_Processes.L2_Cellular_community_eukaryotes.L3_Focal_adhesion	0			-
L1_Metabolism.L2_Metabolism_of_terpenoids_and_polyketides.L3_Zeatin_biosynthesis	3.909430499			-
L1_Genetic_Information_Processing.L2_Replication_and_repair.L3_DNA_replication	4.143179904			-
L1_Metabolism.L2_Biosynthesis_of_other_secondary_metabolites.L3_Flavonoid_biosynthesis	2.562208652			-
L1_Genetic_Information_Processing.L2_Translation.L3_Ribosome_biogenesis_in_eukaryotes	2.896791303			-
L1_Environmental_Information_Processing.L2_Signal_transduction.L3_Two_component_system	3.398596432			-
L1_Human_Diseases.L2_Infectious_diseases__Parasitic.L3_Toxoplasmosis	0.723306361			-
L1_Metabolism.L2_Nucleotide_metabolism.L3_Pyrimidine_metabolism	4.112468778			-
L1_Metabolism.L2_Amino_acid_metabolism.L3_Arginine_and_proline_metabolism	3.792266305			-
L1_Metabolism.L2_Carbohydrate_metabolism.L3_Pyruvate_metabolism	4.092249337			-
L1_Metabolism.L2_Metabolism_of_cofactors_and_vitamins.L3_Folate_biosynthesis	4.195017053	CC	2.615679	0.0002
L1_Metabolism.L2_Amino_acid_metabolism.L3_Valine_leucine_and_isoleucine_biosynthesis	4.330181005	H	2.641098	0.001
L1_Metabolism.L2_Xenobiotics_biodegradation_and_metabolism.L3_Fluorobenzoate_degradation	2.088919189			-
L1_Cellular_Processes.L2_Transport_and_catabolism.L3_Endocytosis	0			-
L1_Metabolism.L2_Xenobiotics_biodegradation_and_metabolism.L3_Dioxin_degradation	3.181465735			-
L1_Metabolism.L2_Amino_acid_metabolism.L3_Tyrosine_metabolism	3.499635799			-
L1_Human_Diseases.L2_Infectious_diseases__Bacterial.L3_Vibrio_cholerae_infection	2.344361963			-
L1_Human_Diseases.L2_Infectious_diseases__Bacterial.L3_Bacterial_invasion_of_epithelial_cells	1.605443406			-
L1_Metabolism.L2_Xenobiotics_biodegradation_and_metabolism.L3_Styrene_degradation	3.236607167			-
L1_Metabolism.L2_Metabolism_of_terpenoids_and_polyketides.L3_Geraniol_degradation	3.162760657			-
L1_Organismal_Systems.L2_Endocrine_system.L3_Insulin_signaling_pathway	2.963224537			-
L1_Metabolism.L2_Xenobiotics_biodegradation_and_metabolism.L3_Metabolism_of_xenobiotics_by_cytochrome_P450	3.259586523	CC	2.606198	0.0006
L1_Metabolism.L2_Glycan_biosynthesis_and_metabolism.L3_Lipopolysaccharide_biosynthesis	4.269012715	CC	2.963771	0.0376

L1_Metabolism.L2_Biosynthesis_of_other_secondary_metabolites.L3_Tropane_piperidine_and_pyridine_alkaloid_biosynthesis	3.671078792			-
L1_Metabolism.L2_Metabolism_of_cofactors_and_vitamins.L3_One_carbon_pool_by_folate	4.302182726			-
L1_Metabolism.L2_Energy_metabolism.L3_Methane_metabolism	3.654051011			-
L1_Genetic_Information_Processing.L2_Replication_and_repair.L3_Homologous_recombination	4.238217884			-
L1_Organismal_Systems.L2_Immune_system.L3_NOD_like_receptor_signaling_pathway	2.847595937			-
L1_Metabolism.L2_Metabolism_of_other_amino_acids.L3_D_Arginine_and_D_ornithine_metabolism	3.46112072			-
L1_Genetic_Information_Processing.L2_Replication_and_repair.L3_Base_excision_repair	4.030186007			-
L1_Human_Diseases.L2_Immune_diseases.L3_Systemic_lupus_erythematosus	2.285430197			-
L1_Metabolism.L2_Metabolism_of_terpenoids_and_polyketides.L3_Sesquiterpenoid_and_triterpenoid_biosynthesis	0			-
L1_Metabolism.L2_Carbohydrate_metabolism.L3_Pentose_phosphate_pathway	4.200880392	H	2.582621	0.0006
L1_Metabolism.L2_Biosynthesis_of_other_secondary_metabolites.L3_Isoflavonoid_biosynthesis	0			-
L1_Metabolism.L2_Amino_acid_metabolism.L3_Phenylalanine_metabolism	3.451162453			-
L1_Metabolism.L2_Energy_metabolism.L3_Photosynthesis_antenna_proteins	0.509686311			-
L1_Metabolism.L2_Metabolism_of_cofactors_and_vitamins.L3_Biotin_metabolism	4.189983918	CC	2.763653	0.013
L1_Genetic_Information_Processing.L2_Folding_sorting_and_degradation.L3_Protein_processing_in_endoplasmic_reticulum	2.462317189			-
L1_Genetic_Information_Processing.L2_Folding_sorting_and_degradation.L3_Proteasome	1.852245449			-
L1_Metabolism.L2_Amino_acid_metabolism.L3_Phenylalanine_tyrosine_and_tryptophan_biosynthesis	4.11120093			-
L1_Metabolism.L2_Carbohydrate_metabolism.L3_C5_Branched_dibasic_acid_metabolism	4.224871894			-
L1_Metabolism.L2_Metabolism_of_terpenoids_and_polyketides.L3_Carotenoid_biosynthesis	2.495921848			-
L1_Metabolism.L2_Carbohydrate_metabolism.L3_Amino_sugar_and_nucleotide_sugar_metabolism	4.014186316			-
L1_Organismal_Systems.L2_Digestive_system.L3_Protein_digestion_and_absorption	2.832987533			-
L1_Metabolism.L2_Energy_metabolism.L3_Sulfur_metabolism	4.008208093			-
L1_Genetic_Information_Processing.L2_Translation.L3_RNA_transport	2.681058872			-
L1_Metabolism.L2_Carbohydrate_metabolism.L3_Butanoate_metabolism	3.842622393			-
L1_Metabolism.L2_Xenobiotics_biodegradation_and_metabolism.L3_Aminobenzoate_degradation	3.195090236			-
L1_Metabolism.L2_Energy_metabolism.L3_Nitrogen_metabolism	3.841114914			-
L1_Metabolism.L2_Metabolism_of_other_amino_acids.L3_Glutathione_metabolism	3.903322386			-
L1_Metabolism.L2_Energy_metabolism.L3_Carbon_fixation_in_photosynthetic_organisms	4.237736612			-
L1_Human_Diseases.L2_Infectious_diseases__Bacterial.L3_Staphylococcus_aureus_infection	2.66471718			-
L1_Metabolism.L2_Metabolism_of_cofactors_and_vitamins.L3_Vitamin_B6_metabolism	4.08864834			-
L1_Metabolism.L2_Xenobiotics_biodegradation_and_metabolism.L3_Bisphenol_degradation	2.915728117			-
L1_Metabolism.L2_Carbohydrate_metabolism.L3_Inositol_phosphate_metabolism	3.347967523			-
L1_Metabolism.L2_Metabolism_of_other_amino_acids.L3_D_Glutamine_and_D_glutamate_metabolism	4.399546844			-
L1_Cellular_Processes.L2_Cell_growth_and_death.L3_Meiosis_yeast	1.364913779			-
L1_Environmental_Information_Processing.L2_Membrane_transport.L3_ABC_transporters	3.861135632			-
L1_Cellular_Processes.L2_Cellular_community_prokaryotes.L3_Biofilm_formation_Vibrio_cholerae	3.297137674			-
L1_Metabolism.L2_Lipid_metabolism.L3_Linoleic_acid_metabolism	3.198954173			-
L1_Metabolism.L2_Glycan_biosynthesis_and_metabolism.L3_Peptidoglycan_biosynthesis	4.317092469			-
L1_Genetic_Information_Processing.L2_Folding_sorting_and_degradation.L3_Protein_export	4.207464598			-
L1_Metabolism.L2_Glycan_biosynthesis_and_metabolism.L3_Other_types_of_O_glycan_biosynthesis	1.268640971			-
L1_Metabolism.L2_Carbohydrate_metabolism.L3_Galactose_metabolism	3.913464031	H	2.675253	0.013
L1_Metabolism.L2_Xenobiotics_biodegradation_and_metabolism.L3_Toluene_degradation	3.328403238			-
L1_Genetic_Information_Processing.L2_Folding_sorting_and_degradation.L3_Sulfur_relay_system	3.973030706			-
L1_Metabolism.L2_Metabolism_of_terpenoids_and_polyketides.L3_Biosynthesis_of_vancomycin_group_antibiotics	4.357672237	CC	2.937801	0.0118
L1_Human_Diseases.L2_Cardiovascular_diseases.L3_Hypertrophic_cardiomyopathy_HCM	0.533754533			-
L1_Metabolism.L2_Glycan_biosynthesis_and_metabolism.L3_Glycosphingolipid_biosynthesis_lacto_and_neolacto_series	0			-
L1_Genetic_Information_Processing.L2_Transcription.L3_RNA_polymerase	3.913898664			-
L1_Metabolism.L2_Amino_acid_metabolism.L3_Alanine_aspartate_and_glutamate_metabolism	4.186476829			-
L1_Metabolism.L2_Xenobiotics_biodegradation_and_metabolism.L3_Benzoate_degradation	3.150901577			-
L1_Human_Diseases.L2_Infectious_diseases__Bacterial.L3_Shigellosis	0			-
L1_Metabolism.L2_Carbohydrate_metabolism.L3_Glycolysis_Gluconeogenesis	4.070652519			-
L1_Metabolism.L2_Amino_acid_metabolism.L3_Lysine_biosynthesis	4.191698918			-
L1_Metabolism.L2_Lipid_metabolism.L3_Synthesis_and_degradation_of_ketone_bodies	3.559495658	H	2.705307	0.0016
L1_Human_Diseases.L2_Infectious_diseases__Parasitic.L3_Chagas_disease_American_trypanosomiasis	1.211584122			-
L1_Metabolism.L2_Energy_metabolism.L3_Oxidative_phosphorylation	3.734662689			-
L1_Metabolism.L2_Metabolism_of_cofactors_and_vitamins.L3_Ubiquinone_and_other_terpenoid_quinone_biosynthesis	3.790157976			-
L1_Organismal_Systems.L2_Endocrine_system.L3_Renin_angiotensin_system	0			-
L1_Metabolism.L2_Energy_metabolism.L3_Carbon_fixation_pathways_in_prokaryotes	4.079524471			-

L1_Metabolism.L2_Xenobiotics_biodegradation_and_metabolism.L3_Ethylbenzene_degradation	3.141149048			-
L1_Human_Diseases.L2_Infectious_diseases__Parasitic.L3_African_trypanosomiasis	1.91843405			-
L1_Metabolism.L2_Metabolism_of_other_amino_acids.L3_beta_Alanine_metabolism	3.683322678			-
L1_Metabolism.L2_Lipid_metabolism.L3_Glycerophospholipid_metabolism	3.825801111			-
L1_Metabolism.L2_Carbohydrate_metabolism.L3_Fructose_and_mannose_metabolism	3.91976181			-
L1_Metabolism.L2_Lipid_metabolism.L3_Secondary_bile_acid_biosynthesis	2.98735526			-
L1_Metabolism.L2_Metabolism_of_other_amino_acids.L3_Cyanoamino_acid_metabolism	3.758808352	CC	2.924545	0.0034
L1_Genetic_Information_Processing.L2_Transcription.L3_Spliceosome	1.706480795			-
L1_Metabolism.L2_Lipid_metabolism.L3_Glycerolipid_metabolism	3.602793709			-
L1_Metabolism.L2_Metabolism_of_other_amino_acids.L3_Phosphonate_and_phosphinate_metabolism	3.291016964			-
L1_Genetic_Information_Processing.L2_Replication_and_repair.L3_Non_homologous_end_joining	1.659959289			-
L1_Metabolism.L2_Lipid_metabolism.L3_Primary_bile_acid_biosynthesis	2.10784858			-
L1_Metabolism.L2_Biosynthesis_of_other_secondary_metabolites.L3_Streptomycin_biosynthesis	4.193578543			-
L1_Metabolism.L2_Carbohydrate_metabolism.L3_Glyoxylate_and_dicarboxylate_metabolism	3.750259745			-
L1_Organismal_Systems.L2_Excretory_system.L3_Vasopressin_regulated_water_reabsorption	0			-
L1_Cellular_Processes.L2_Cell_motility.L3_Flagellar_assembly	3.29252061			-
L1_Metabolism.L2_Xenobiotics_biodegradation_and_metabolism.L3_Naphthalene_degradation	2.390523827			-
L1_Metabolism.L2_Carbohydrate_metabolism.L3_Ascorbate_and_aldarate_metabolism	3.402146255			-
L1_Metabolism.L2_Xenobiotics_biodegradation_and_metabolism.L3_Chloroalkane_and_chloroalkene_degradation	3.411630747			-
L1_Cellular_Processes.L2_Cell_motility.L3_Bacterial_chemotaxis	3.610892368			-
L1_Environmental_Information_Processing.L2_Membrane_transport.L3_Phosphotransferase_system_PTS	3.702208516			-
L1_Metabolism.L2_Carbohydrate_metabolism.L3_Citrate_cycle_TCA_cycle	4.075022678			-
L1_Metabolism.L2_Metabolism_of_cofactors_and_vitamins.L3_Pantothenate_and_CoA_biosynthesis	4.249881642			-
L1_Metabolism.L2_Lipid_metabolism.L3_Fatty_acid_biosynthesis	4.26604509			-
L1_Cellular_Processes.L2_Transport_and_catabolism.L3_Peroxisome	3.44000292			-
L1_Genetic_Information_Processing.L2_Replication_and_repair.L3_Nucleotide_excision_repair	3.929651114			-
L1_Metabolism.L2_Lipid_metabolism.L3_Steroid_biosynthesis	1.395944208			-
L1_Metabolism.L2_Glycan_biosynthesis_and_metabolism.L3_Glycosaminoglycan_degradation	3.605634766			-
L1_Metabolism.L2_Amino_acid_metabolism.L3_Tryptophan_metabolism	3.341828701			-
L1_Metabolism.L2_Amino_acid_metabolism.L3_Lysine_degradation	3.244733287			-
L1_Metabolism.L2_Metabolism_of_other_amino_acids.L3_Selenocompound_metabolism	4.074328487			-
L1_Human_Diseases.L2_Infectious_diseases__Bacterial.L3_Epithelial_cell_signaling_in_Helicobacter_pylori_infection	3.184145184			-
L1_Metabolism.L2_Xenobiotics_biodegradation_and_metabolism.L3_Polycyclic_aromatic_hydrocarbon_degradation	1.582526844			-
L1_Metabolism.L2_Metabolism_of_cofactors_and_vitamins.L3_Lipoic_acid_metabolism	4.130742797			-
L1_Metabolism.L2_Carbohydrate_metabolism.L3_Starch_and_sucrose_metabolism	3.901137421			-
L1_Genetic_Information_Processing.L2_Translation.L3_Ribosome	4.25999274			-
L1_Genetic_Information_Processing.L2_Folding_sorting_and_degradation.L3_RNA_degradation	3.828354083			-
L1_Metabolism.L2_Metabolism_of_cofactors_and_vitamins.L3_Retinol_metabolism	2.972164085			-
L1_Genetic_Information_Processing.L2_Replication_and_repair.L3_Mismatch_repair	4.278454094			-
L1_Metabolism.L2_Xenobiotics_biodegradation_and_metabolism.L3_Atrazine_degradation	2.428096345			-
L1_Organismal_Systems.L2_Environmental_adaptation.L3_Plant_pathogen_interaction	3.227654141			-
L1_Metabolism.L2_Metabolism_of_terpenoids_and_polyketides.L3_Biosynthesis_of_ansamycins	4.615876441	H	3.365285	0.009
L1_Metabolism.L2_Metabolism_of_terpenoids_and_polyketides.L3_Biosynthesis_of_type_II_polyketide_backbone	0			-
L1_Metabolism.L2_Energy_metabolism.L3_Photosynthesis	3.691097017	CC	2.837926	0.0433
L1_Metabolism.L2_Glycan_biosynthesis_and_metabolism.L3_Various_types_of_N_glycan_biosynthesis	0			-
L1_Metabolism.L2_Glycan_biosynthesis_and_metabolism.L3_Other_glycan_degradation	4.041557513			-
L1_Metabolism.L2_Metabolism_of_cofactors_and_vitamins.L3_Thiamine_metabolism	4.162356363			-
L1_Human_Diseases.L2_Infectious_diseases__Parasitic.L3_Amoebiasis	1.922963303			-
L1_Metabolism.L2_Carbohydrate_metabolism.L3_Pentose_and_glucuronate_interconversions	3.545603452			-

Supplementary Table S8. the corresponding output value and POD valueof each optimal microbial marker in the discovery cohort										
rownames(Data)	OTU10 (Alloprevotella)	OTU26 (Prevotella)	OTU71 (Alloprevotella)	OTU29 (Porphyromonas)	OTU5 (Haemophilus)	OTU67 (Alloprevotella)	OTU252 (Prevotella)	OTU34 (Halomonas)	group	POD(H)	POD(CC)
H002	0.071665	0.006821	0.005284	0.004889	0.036033	0.001428	0.001498	1.30E-04	H	1	0
H003	0.060622	0.014649	0.011264	0	0.05031	2.98E-04	0.008751	6.75E-04	H	0.99719888	0.00280112
H004	0.041554	0.018422	2.92E-04	4.34E-04	0.060625	0.005587	1.91E-04	4.10E-05	H	0.994708995	0.005291005
H005	0.044963	6.14E-04	0.003092	0.001462	0.00428	6.99E-04	8.90E-05	6.90E-05	H	0.881081081	0.118918919
H008	0.058069	0.012039	0.028048	0	0.010553	3.59E-04	1.81E-04	2.00E-05	H	0.989041096	0.010958904
H009	0.058344	0.029502	0.001166	6.23E-04	0.020222	0.013006	0.010278	2.90E-05	H	1	0
H010	0.10336	0.014299	0.007075	0	0.039821	1.44E-04	0.018926	0.00134	H	0.988571429	0.011428571
H011	0.090771	0.011081	0.002338	0.002158	0.011217	0.003978	0.022857	3.09E-04	H	1	0
H013	0.057774	0.004309	4.43E-04	0.003528	0.019186	0.003279	0	0	H	0.986772487	0.013227513
H015	0.051915	0.01719	0.002372	1.67E-04	0.043167	0.008916	2.10E-05	2.10E-05	H	0.982905983	0.017094017
H017	0.01752	0.010883	3.28E-04	0.008386	0.037434	0.002931	2.90E-05	3.40E-05	H	0.994609164	0.005390836
H019	0.093084	0.013658	0.019168	0.00248	0.014381	5.40E-04	2.62E-04	0	H	1	0
H020	0.08763	0.025215	0.005237	0.005044	0.028522	0.001004	0.001066	1.13E-04	H	0.997093023	0.002906977
H021	0.027945	0.004593	0.004129	0.050491	0.007704	0.004517	3.20E-04	2.69E-04	H	0.997245179	0.002754821
H023	0.0468	0.010992	0.010784	0.001667	0.02896	0.018733	0	0	H	1	0
H024	0.046669	0.005521	8.30E-05	1.81E-04	0.006796	0.00149	0	0	H	0.712365591	0.287634409
H025	0.034724	0.019556	2.40E-04	0.007158	0.078645	0.007922	7.10E-05	6.40E-05	H	1	0
H027	0.026283	0.03411	0.004446	3.00E-05	0.03487	0	0.006367	1.63E-04	H	0.95890411	0.04109589
H028	0.03164	0.016785	0.02419	4.60E-04	0.026763	0.001111	0	0	H	0.939153439	0.060846561
H029	0.088287	0.011089	0.012583	0.007407	0.051103	1.10E-04	0.002168	1.47E-04	H	0.997175141	0.002824859
H032	0.022348	0.004024	0.00398	7.87E-04	0.003152	0.006662	5.49E-04	2.18E-04	H	0.89258312	0.10741688
H034	0.013498	0.009553	0.006517	0.001094	0.033579	0.001381	0	0	H	0.965517241	0.034482759
H035	0.081266	0.042442	2.08E-04	0.02348	0.068106	0.017323	0.003	1.62E-04	H	0.979643766	0.020356234
H036	0.039921	0.013946	0.001616	0.00275	0.023979	0.001867	1.11E-04	0	H	1	0
H039	0.074109	0.009111	0.006796	0	0.024271	1.40E-05	7.20E-05	0	H	0.959537572	0.040462428
H040	0.048462	0.007237	0.049194	9.99E-04	0.033032	5.76E-04	3.12E-04	3.49E-04	H	1	0
H041	0.030114	8.65E-04	7.43E-04	3.30E-05	0.003457	1.27E-04	2.30E-04	3.40E-05	H	0.802197802	0.197802198
H044	0.007523	0.003144	2.21E-04	0.002933	0.057711	9.10E-05	1.92E-04	1.26E-04	H	0.869942197	0.130057803
H048	0.004324	0.025184	0.007673	0.006432	0.07616	6.52E-04	1.31E-04	2.13E-04	H	0.865168539	0.134831461
H049	0.014518	0.012371	2.79E-04	0.062202	0.056337	0.006066	8.30E-04	1.68E-04	H	0.994186047	0.005813953
H051	0.108256	0.012373	0.003147	0.018699	0.078746	0.02605	0.001132	1.12E-04	H	1	0
H053	4.89E-04	0	0	4.65E-04	0.004068	0	0.136373	0	H	0.157622739	0.842377261
H057	0.017459	0.009338	7.60E-05	0.001607	0.028067	0	2.72E-04	7.70E-05	H	0.798365123	0.201634877
H058	0.015019	0.004533	0.001988	0	0.016661	1.08E-04	0.089177	0	H	0.983516484	0.016483516
H059	0.005077	0.005576	0	0	0.028606	3.99E-04	0.086398	0	H	0.597368421	0.402631579
H060	0.138805	4.40E-04	0.013757	0.014134	0.042114	0.002253	0.014903	5.04E-04	H	0.917737789	0.082262211
H061	0.028545	0.006815	5.00E-04	0.004589	0.002373	0.004148	0	0	H	0.820855615	0.179144385
H062	0.017341	0.010191	6.32E-04	0.005104	0.049701	0.008857	0.008131	0.001767	H	0.986376022	0.013623978
H063	0.002601	0.004826	9.90E-05	0.016607	0.069981	0.012445	4.07E-04	1.34E-04	H	0.634920635	0.365079365
H064	0.029758	0.039637	6.00E-05	0.002867	0.072119	0.001026	2.40E-05	8.70E-05	H	0.863157895	0.136842105
H065	0.075201	0.014686	0.001016	0.001628	0.119246	0.089314	0.001439	3.41E-04	H	0.982005141	0.017994859
H066	0.025504	0.005546	0.006465	0.036816	0.057992	0	0.011706	0.003937	H	0.864864865	0.135135135
H067	0.043834	0.03767	0.002838	6.80E-05	0.064997	0.005346	1.09E-04	1.06E-04	H	0.997333333	0.002666667
H070	0.007897	0.004198	0.007694	0	0.143909	8.90E-05	0	3.00E-05	H	0.725212465	0.274787535
H071	0.092555	0.002132	0.011921	0.002657	0.011084	0.003118	1.49E-04	3.30E-05	H	0.989041096	0.010958904
H073	0.012243	0.004383	0.053734	0.003046	0.031257	0.001345	3.20E-05	1.05E-04	H	0.99734748	0.00265252
H074	0.054474	0.010171	2.57E-04	0.00115	0.06074	0.002196	0	0	H	0.965517241	0.034482759
H075	0.020393	0.019637	2.31E-04	0.003624	0.079922	0.006721	8.00E-04	0.002942	H	0.9375	0.0625
H076	0.03519	0.008096	0.027663	0	0.014614	0.004701	1.40E-05	0	H	0.97721519	0.02278481
H078	0.053695	0.007879	0.024343	0.002096	0.02872	0.005474	4.70E-05	0	H	1	0
H079	0.054505	0.002658	0.002201	0.001009	0.0081	2.53E-04	0	0	H	0.882022472	0.117977528
H083	0.017132	0.022264	0.002231	0.003368	0.075032	0.014342	9.10E-05	6.40E-05	H	1	0
H084	0.013827	0.004199	0.003835	0.013405	0.063553	0.007843	8.82E-04	3.12E-04	H	1	0
H086	0.051763	0.009466	7.48E-04	0.015631	0.020686	0.00635	5.70E-05	1.81E-04	H	1	0
H088	0.055384	0.003817	0.004408	0.062848	0.024462	0.001063	6.30E-05	2.28E-04	H	0.991253644	0.008746356

H089	0.040195	0.005346	0.001095	0.001277	0.013188	0.00152	0	0	H	0.994350282	0.005649718
H090	0.044787	0.003261	0.026812	3.02E-04	0.036973	4.80E-05	1.49E-04	0	H	0.95264624	0.04735376
H091	0.045619	0.02008	0.003785	0	0.020105	0.002927	0.002845	5.86E-04	H	0.989361702	0.010638298
H092	0.115768	0.018248	0.007391	0.014503	0.036335	0.011123	8.40E-05	0	H	1	0
H093	0.030992	0.013011	0.00543	7.89E-04	0.051412	0.005576	2.77E-04	0	H	1	0
H094	0.044984	0.006953	0.020815	0.002699	0.01894	6.73E-04	3.50E-05	3.70E-05	H	0.994397759	0.005602241
H096	0.013673	0.010953	0.00393	0.001274	0.156204	0.01041	9.23E-04	4.79E-04	H	0.977961433	0.022038567
H097	0.035786	0.01464	0.012154	3.12E-04	0.041705	0.002396	4.10E-05	0	H	1	0
H098	0.040004	0.00709	0.074558	2.70E-05	0.079104	0.012995	0.001048	1.58E-04	H	0.991620112	0.008379888
H099	0.049781	0.004007	0.166693	0	0.028455	0.002693	0.003592	0.002017	H	0.960212202	0.039787798
H100	0.057319	0.004579	0.034961	0	0.021709	0.005848	7.15E-04	1.31E-04	H	0.997229917	0.002770083
H102	0.037952	0.00947	2.44E-04	1.09E-04	0.051053	0.048582	1.11E-04	6.80E-05	H	0.986149584	0.013850416
H104	0.098941	0.007038	0.001712	0.003076	0.067654	0.003325	0.006516	8.11E-04	H	1	0
H107	0.041294	0.034246	0.001179	0.001208	0.08149	0.00802	0.013004	0.001283	H	1	0
H108	0.080637	0.028676	0.003026	0	0.083408	0.021424	3.18E-04	2.06E-04	H	0.997340426	0.002659574
H109	0.078129	0.021387	8.75E-04	0	0.040663	0.025118	1.50E-05	3.60E-05	H	0.974431818	0.025568182
H110	0.02162	0.023475	0.003241	0.002883	0.057953	0.019834	0.001504	1.72E-04	H	1	0
H113	0.097204	0.019802	0.010992	2.78E-04	0.022765	0.014801	0.003071	9.80E-05	H	1	0
H114	0.012756	0.053437	0.032325	0	0.055373	0.001789	1.20E-05	4.70E-05	H	0.977653631	0.022346369
H116	0.037866	0.002669	0.02876	1.40E-04	0.012281	0.001874	1.00E-04	2.20E-05	H	0.964769648	0.035230352
H118	0.092623	0.079959	0.007409	0	0.045838	7.56E-04	0.005913	0	H	1	0
H120	0.065596	0.013053	0.012708	1.93E-04	0.131309	0.002229	0.0016	1.16E-04	H	0.976	0.024
H122	0.022281	0.003465	0.00426	0.003007	0.271669	0.005944	7.11E-04	3.01E-04	H	0.994778068	0.005221932
H123	0.063179	0.012549	0.009564	0.003262	0.0671	0.006456	0.001692	2.41E-04	H	1	0
H124	0.083639	0.017498	0.017349	0.004717	0.051928	0.016609	0.001175	2.26E-04	H	0.997282609	0.002717391
H125	0.014122	4.34E-04	1.34E-04	0	0.003494	2.12E-04	0.090153	0	H	0.595800525	0.404199475
H126	0.056543	0.006755	0.008721	0.015497	0.024327	0.006459	3.15E-04	1.76E-04	H	1	0
H127	0.047149	0.033854	0.005787	9.54E-04	0.040986	0.026506	3.50E-05	2.80E-05	H	1	0
H130	0.004621	0.003251	0.035994	0.001203	0.009081	8.42E-04	7.90E-05	0	H	0.855555556	0.144444444
H131	0.01421	0.005361	0.019102	2.40E-05	0.01717	3.05E-04	0.001199	2.29E-04	H	0.969863014	0.030136986
H133	0.023343	0.042546	2.00E-05	7.10E-05	0.023643	0.004011	0.002115	5.50E-05	H	0.766666667	0.233333333
H137	0.001172	0.004158	0.011821	0.001622	0.041253	0.017804	0	0	H	0.467391304	0.532608696
H139	0.028432	0.006862	2.22E-04	0.006063	0.120339	0	2.70E-05	3.07E-04	H	0.909574468	0.090425532
H141	0.026722	0.031848	0.001526	0	0.036621	9.84E-04	3.90E-05	1.83E-04	H	0.983286908	0.016713092
H142	0.043599	0.004373	2.27E-04	4.33E-04	0.042017	0	3.56E-04	1.46E-04	H	0.962962963	0.037037037
H146	0.007401	0.127197	4.93E-04	0	0.005166	0.001316	0.001963	0	H	0.799450549	0.200549451
H147	0.027643	0.005848	5.43E-04	0.003503	0.039458	0.004064	0	0	H	0.992	0.008
H149	0.006262	0.00106	4.99E-04	0	0.091292	9.56E-04	3.10E-05	1.44E-04	H	0.663101604	0.336898396
H150	0.053082	0.017278	0.007748	2.12E-04	0.024227	4.38E-04	2.50E-05	0	H	0.989071038	0.010928962
CC01	0	4.38E-04	0	0	8.74E-04	0	0	1.53E-04	CC	0.002793296	0.997206704
CC02	0.011077	3.28E-04	1.50E-04	1.80E-05	7.50E-05	0	4.70E-05	0.004329	CC	0.558746736	0.441253264
CC05	0.002475	2.58E-04	0	0	7.69E-04	0	0	5.90E-05	CC	0.011527378	0.988472622
CC06	0.002152	0	2.94E-04	0	1.60E-05	0	0	0	CC	0.146341463	0.853658537
CC08	0	1.07E-04	0	0	3.10E-05	0	0	2.40E-05	CC	0.005319149	0.994680851
CC09	8.25E-04	4.42E-04	0	0	0.001747	0	0	0.002982	CC	0.002724796	0.997275204
CC12	0	0	0	0	0.062756	0	0	2.53E-04	CC	0.023872679	0.976127321
CC16	3.26E-04	3.43E-04	2.60E-05	3.31E-04	0.005518	2.24E-04	0.001108	0.026888	CC	0.079081633	0.920918367
CC17	8.90E-05	0.001149	6.10E-05	0	0.002857	0	0.014741	0.01011	CC	0.122015915	0.877984085
CC20	4.68E-04	0.004014	0	0	1.21E-04	8.40E-05	1.15E-04	0.0198	CC	0.051282051	0.948717949
CC21	5.68E-04	0.002686	0	0	0.069076	0.001047	2.90E-05	0.071493	CC	0.102040816	0.897959184
CC24	0.003881	6.84E-04	2.10E-05	1.80E-05	0.001579	1.51E-04	0	1.78E-04	CC	0.08839779	0.91160221
CC25	2.67E-04	0.001284	0	0	0.003254	1.29E-04	0	2.63E-04	CC	0.002949853	0.997050147
CC26	1.55E-04	9.40E-05	0	0	0.017573	0	0	4.35E-04	CC	0.002739726	0.997260274
CC27	6.72E-04	0.001966	2.90E-05	0.00105	0.007688	5.80E-05	2.20E-05	1.80E-05	CC	0.235751295	0.764248705
CC28	0	1.23E-04	0	0	0.00291	0	0	0.001221	CC	0	1
CC29	0.00424	8.79E-04	0	0	0.001	0	0	0.002451	CC	0.103448276	0.896551724
CC30	0.002532	4.13E-04	0	0	0.004419	0	0	3.39E-04	CC	0.024793388	0.975206612
CC31	4.18E-04	1.23E-04	0	6.90E-05	2.27E-04	0	0	2.71E-04	CC	0.011204482	0.988795518

CC33	1.29E-04	5.90E-05	0	2.14E-04	9.14E-04	0	0	2.02E-04	CC	0.03038674	0.96961326
CC34	0.005969	0.016982	1.89E-04	2.50E-05	0.004239	0.001819	0	1.80E-05	CC	0.783625731	0.216374269
CC35	8.90E-05	0	0	0	6.31E-04	0	0	0	CC	0.03968254	0.96031746
CC36	0.002337	0.001014	0	0	0.06718	8.83E-04	3.21E-04	0.023502	CC	0.152173913	0.847826087
CC37	4.18E-04	0.001276	7.00E-05	0	0.00638	6.20E-05	0	0.001362	CC	0.03021148	0.96978852
CC39	1.02E-04	0.003831	0	0	0.013396	0	3.40E-05	1.74E-04	CC	0.161016949	0.838983051
CC42	0	0.007879	0	0	0.0242	5.74E-04	0	5.71E-04	CC	0.143222506	0.856777494
CC43	3.70E-05	1.60E-05	0	0	2.13E-04	0	6.74E-04	0.044794	CC	0.019178082	0.980821918
CC44	2.58E-04	0.003174	1.12E-04	5.70E-05	0.024842	0	0	0.001993	CC	0.166666667	0.833333333
CC45	3.32E-04	0.001992	0	0	0.019614	9.91E-04	0	0	CC	0.080645161	0.919354839
CC46	0	0	1.05E-04	0	0.019492	9.40E-05	0	0	CC	0.094182825	0.905817175
CC48	0	2.10E-05	0	0	2.02E-04	6.50E-05	0	0.029702	CC	0	1
CC52	0	1.34E-04	4.30E-05	0.001653	0.042351	0	9.71E-04	0.044685	CC	0.191011236	0.808988764
CC54	0.001761	3.18E-04	0	0	1.87E-04	0	0	9.60E-05	CC	0.00265252	0.99734748
CC55	1.92E-04	0.023183	0	0	1.39E-04	0	0	0.00739	CC	0.049723757	0.950276243
CC57	4.93E-04	1.30E-04	2.09E-04	1.65E-04	0.019942	5.37E-04	1.09E-04	0.054377	CC	0.192200557	0.807799443
CC58	1.08E-04	2.30E-04	0	0	0.002399	0	0	7.54E-04	CC	0	1
CC60	3.87E-04	0	0	0.001047	6.17E-04	1.58E-04	0	0	CC	0.229110512	0.770889488
CC62	4.34E-04	1.92E-04	0	0	1.31E-04	1.58E-04	0	1.90E-04	CC	0.002762431	0.997237569
CC63	1.49E-04	0.001934	0	5.00E-05	0.002714	0	0	8.70E-05	CC	0.00802139	0.99197861
CC64	9.40E-05	1.43E-04	0	1.10E-05	0.001689	2.88E-04	0	2.60E-05	CC	0.005540166	0.994459834
CC65	0.001383	4.03E-04	0	2.30E-05	0.003558	0	0	6.17E-04	CC	0.021857923	0.978142077
CC67	6.39E-04	0.001084	2.74E-04	0	0.004737	3.50E-05	0	0	CC	0.180628272	0.819371728
CC68	0	7.07E-04	0	0	0.011327	5.85E-04	0	0	CC	0.050531915	0.949468085
CC69	3.14E-04	0.002433	0	1.34E-04	0.00434	8.94E-04	0	6.60E-05	CC	0.10982659	0.89017341
CC70	5.27E-04	0.007182	0	0	0.002988	4.60E-04	0	0.003929	CC	0.058171745	0.941828255
CC71	0	1.21E-04	0	0	0.136297	7.80E-04	0	3.10E-05	CC	0.062337662	0.937662338
CC72	6.45E-04	0.012509	0.002011	0	0.12503	0.006898	0	0.004864	CC	0.465875371	0.534124629
CC73	0.001063	0.003746	0	1.80E-05	0.044164	2.73E-04	0	0.003175	CC	0.139130435	0.860869565

Supplementary Table S9. the corresponding output value and POD valueof each optimal microbial marker in the validation cohort										
SampleID	OTU5 (Haemophilus)	OTU10 (Alloprevotella)	OTU26 (Prevotella)	OTU29 (Porphyromonas)	OTU34 (Halomonas)	OTU67 (Alloprevotella)	OTU71 (Alloprevotella)	OTU252 (Prevotella)	POD(H)	POD(CC)
H006	0.025335	0.005892	0.002772	0.011426	2.60E-04	0.00368	0.001602	0.001118	0.935	0.065
H014	0.010686	0.039625	0.004085	2.82E-04	0	8.72E-04	0.003012	8.60E-05	0.998	0.002
H016	0.00554	0.034345	0.004225	0.002536	0	0	5.98E-04	3.10E-05	0.919	0.081
H018	0.061137	0.039745	0.036134	4.00E-05	3.40E-05	0.032793	0.001889	0.001057	0.998	0.002
H026	0.008158	0.148932	0.00309	1.73E-04	2.10E-05	3.29E-04	0.014516	0.001448	0.992	0.008
H030	0.024287	0.069639	0.001646	0.017919	2.80E-05	0.002521	0.002825	3.10E-05	0.968	0.032
H031	0.029449	0.012357	0.009768	0.002557	3.60E-04	0.021889	0.007456	3.57E-04	1	0
H033	0.010302	0.008737	0.001739	0.00197	8.80E-05	0.004627	2.28E-04	0	0.799	0.201
H038	0.004819	0.044032	0.003322	4.52E-04	0	0.001802	0.016648	0	0.943	0.057
H042	0.0108	0.023244	4.22E-04	0.003282	2.30E-05	5.50E-05	0.038952	2.30E-05	0.861	0.139
H043	0.04826	0.063016	0.053227	0.002588	1.01E-04	0.009466	0.003225	7.60E-05	1	0
H045	0.00748	0.001736	0	0.011787	5.59E-04	8.52E-04	1.57E-04	3.82E-04	0.372	0.628
H046	0.050842	0.02173	0.014105	0.003963	7.30E-05	0.001202	0.001431	6.00E-05	1	0
H047	0.034881	0.025654	0.022395	0	2.32E-04	0	5.42E-04	0.005772	0.965	0.035
H050	0.030476	0.057304	0.003737	0.024151	2.00E-04	6.33E-04	0.01501	1.13E-04	0.996	0.004
H052	0.102663	0.021537	0.002881	3.09E-04	4.00E-05	0.021094	8.80E-05	0.001229	0.881	0.119
H054	0.014104	0.01654	0.006403	0.027841	5.78E-04	0.007915	0.0013	0.024921	0.996	0.004
H055	0.022427	0	0	7.90E-05	8.40E-05	0.036755	0	0.045874	0.297	0.703
H056	8.41E-04	0.070254	4.80E-05	0.024943	1.96E-04	0.001424	0.002015	2.12E-04	0.742	0.258
H068	0.016413	0.043169	0.00895	2.60E-05	2.71E-04	0.009747	3.20E-04	5.82E-04	0.972	0.028
H069	0.041748	0.033679	0.007827	3.93E-04	4.90E-05	0	0.005714	0	0.931	0.069
H072	0.020162	0.036445	0.00668	0.001007	0	0.02689	0.004749	3.80E-05	1	0
H077	0.027445	0.044523	0.002108	0.002204	0	0.004803	6.13E-04	0	0.952	0.048
H080	0.054068	0.066962	9.86E-04	0.004483	1.06E-04	0.00403	0.001282	0	0.915	0.085
H081	0.064643	0.028831	0.082044	0	3.27E-04	0	2.58E-04	0.001296	0.941	0.059
H082	0.019533	0.076179	0.020489	0.001416	4.30E-05	0.011001	0.00115	1.03E-04	1	0
H085	0.001773	0.004118	5.85E-04	0.002864	1.38E-04	2.11E-04	0.006514	8.70E-05	0.631	0.369
H087	0.042178	0.081098	0.018317	2.80E-04	2.40E-05	0.00246	4.40E-05	1.26E-04	0.874	0.126
H095	0.045585	0.011526	0.023139	0.001077	5.60E-05	0.023381	0.009942	5.61E-04	0.994	0.006
H101	0.084984	0.01034	0.018187	0.005655	1.97E-04	0.001331	2.68E-04	3.69E-04	0.99	0.01
H103	0.013926	0.023039	0.004497	0.019602	1.66E-04	0.00211	0.001485	4.00E-05	0.996	0.004
H105	0.114463	0.058853	0.031395	0.001819	0.001205	0.035106	4.82E-04	0.009045	0.992	0.008
H106	0.02148	0.038039	0.011865	0.071559	4.20E-05	0.008127	0.003826	2.60E-04	1	0
H111	0.062478	0.190903	0.076171	0.003396	1.09E-04	0.00473	0.001063	2.90E-04	1	0
H112	0.021811	0.112873	0.010143	0.003402	7.60E-05	0.00538	0.057106	2.55E-04	1	0
H115	0.072655	0.011676	0.002603	0	4.40E-05	0	0	4.80E-05	0.567	0.433
H117	0.010371	0.02263	0.004059	6.73E-04	2.80E-05	5.04E-04	2.70E-04	2.90E-05	0.974	0.026
H119	0.068675	0.034558	0.011063	0.01247	3.91E-04	0.032529	0.006438	4.36E-04	1	0
H121	0.0662	0.031032	0.022103	0.004348	4.50E-05	0.006434	8.20E-05	0.001721	0.976	0.024
H129	0.01571	0.029295	0.00857	0.006538	3.40E-05	0.00119	0.007544	5.80E-05	1	0
H132	0.127956	0.026791	0.012135	0.002527	2.57E-04	0.023739	0	2.21E-04	0.821	0.179
H135	0.016197	0.019489	0.007338	3.91E-04	0	3.72E-04	0.008252	0	0.968	0.032
H136	0.013114	0.031896	0.016584	0	9.60E-05	8.25E-04	0.004075	0	0.881	0.119
H138	0.039681	0.017895	9.90E-04	0	5.90E-05	2.47E-04	0.022534	0	0.817	0.183
H140	0.152457	0.009239	0.012063	0.018526	0.001072	0.024626	1.53E-04	3.00E-05	0.891	0.109
H143	0.02735	0.032836	0.010043	0.115912	8.40E-05	0.008533	0.004813	8.70E-05	1	0
CC03	0.099779	0	1.95E-04	0.010451	0.032827	0	0	1.30E-05	0.128	0.872
CC04	0.010281	1.72E-04	7.04E-04	0	0	0	0	0	0.025	0.975
CC07	1.23E-04	0	8.40E-05	0	0	4.60E-05	4.40E-05	0	0.009	0.991
CC10	0.005024	0.020021	0.049762	0	0.188121	0.001342	2.70E-05	4.20E-04	0.619	0.381
CC11	1.42E-04	4.70E-05	0.002193	0	7.71E-04	3.10E-05	0	0	0.002	0.998
CC13	0.005559	0	0.001854	4.90E-05	0.00163	3.23E-04	0	5.40E-05	0.064	0.936
CC15	3.60E-05	5.60E-05	0.097106	0	0	0	0	0	0.089	0.911
CC18	0.001214	1.56E-04	5.70E-05	2.46E-04	0.006885	0	2.31E-04	0	0.083	0.917
CC19	8.20E-05	0	0	0	7.90E-05	0	0	0	0.008	0.992

CC22	0.001878	2.89E-04	0.002572	0	0	0	0	0	0.024	0.976
CC23	3.04E-04	2.39E-04	6.16E-04	0	0	0	0	0	0.013	0.987
CC32	4.41E-04	1.38E-04	1.54E-04	0	1.45E-04	0	0	0	0.001	0.999
CC38	2.13E-04	0	7.00E-05	0	7.70E-05	0	0	0	0	1
CC40	0.015961	0.001096	0.011366	0	0.030382	1.28E-04	0	0	0.07	0.93
CC41	0.001319	0.001295	0	0	0	0	2.44E-04	0	0.061	0.939
CC47	9.90E-05	9.10E-05	0	0	0	0	9.20E-05	0	0.02	0.98
CC49	6.44E-04	2.22E-04	8.88E-04	0	1.13E-04	0	0	0	0	1
CC50	5.24E-04	0	0	0	0	0	0	0	0.018	0.982
CC51	0.024989	9.00E-05	4.70E-05	0	0.008301	3.10E-05	2.70E-05	2.90E-05	0.022	0.978
CC53	1.93E-04	4.40E-05	3.96E-04	0	0.00138	0	0	0	0	1
CC56	6.75E-04	2.74E-04	4.50E-05	0	4.80E-05	3.80E-05	0	0	0.002	0.998
CC59	0.027355	0.005104	0.004374	0	0.005696	0	3.80E-05	0	0.363	0.637
CC61	0.010136	0.001146	8.00E-05	2.30E-05	0.015732	0	0	0	0.012	0.988
CC66	7.37E-04	0.002779	0.001499	6.36E-04	4.50E-05	8.40E-05	2.80E-05	0	0.127	0.873
CC74	2.60E-05	0.020825	0.017794	0	1.20E-05	8.70E-05	6.60E-04	0	0.666	0.334

Supplementary Table S10. the correSConding output value and POD valueof each optimal microbial marker in the in the SC (n=36) and H(n=46) groups.

SampleID	OTU5 (Haemophilus)	OTU10 (Alloprevotella)	OTU26 (Prevotella)	OTU29 (Porphyromonas)	OTU34 (Halomonas)	OTU67 (Alloprevotella)	OTU71 (Alloprevotella)	OTU252 (Prevotella)	POD(H)	POD(SC)
H006	0.02494	0.005897	0.002763	0.01125	2.44E-04	0.003708	0.001586	0.001129	0.936	0.064
H014	0.010548	0.039656	0.003981	3.03E-04	0	8.79E-04	0.003029	9.60E-05	0.998	0.002
H016	0.005602	0.034508	0.004291	0.00248	0	0	5.53E-04	3.40E-05	0.921	0.079
H018	0.061794	0.03997	0.035719	3.40E-05	3.40E-05	0.032897	0.001923	0.001049	0.998	0.002
H026	0.008169	0.148829	0.003238	1.79E-04	2.70E-05	3.33E-04	0.014713	0.001471	0.994	0.006
H030	0.024129	0.070329	0.001632	0.01749	1.90E-05	0.00249	0.002823	4.20E-05	0.965	0.035
H031	0.029174	0.012376	0.009729	0.0027	3.55E-04	0.021756	0.007571	3.50E-04	1	0
H033	0.010309	0.008738	0.001738	0.0019	8.00E-05	0.004723	2.11E-04	0	0.734	0.266
H038	0.004796	0.043831	0.003412	4.40E-04	0	0.00184	0.016628	0	0.943	0.057
H042	0.010689	0.023417	4.72E-04	0.00337	3.10E-05	5.30E-05	0.038857	1.50E-05	0.87	0.13
H043	0.0485	0.062772	0.053347	0.00254	7.80E-05	0.009547	0.003286	6.50E-05	1	0
H045	0.00753	0.001744	0	0.01193	5.59E-04	9.24E-04	1.95E-04	4.19E-04	0.386	0.614
H046	0.050906	0.02165	0.013956	0.0041	5.80E-05	0.001163	0.001464	6.70E-05	1	0
H047	0.034629	0.025729	0.022251	0	2.80E-04	0	5.16E-04	0.005729	0.963	0.037
H050	0.030332	0.057696	0.00371	0.02393	1.75E-04	6.79E-04	0.015142	1.14E-04	0.996	0.004
H052	0.103024	0.022	0.002819	3.13E-04	3.80E-05	0.021099	8.70E-05	0.001205	0.879	0.121
H054	0.014181	0.016759	0.006383	0.02769	5.88E-04	0.008044	0.001258	0.024623	0.997	0.003
H055	0.022311	0	0	8.60E-05	7.90E-05	0.03683	0	0.045607	0.293	0.707
H056	9.33E-04	0.070131	5.50E-05	0.02493	1.85E-04	0.00145	0.002018	2.18E-04	0.742	0.258
H068	0.016681	0.043253	0.008941	3.80E-05	2.63E-04	0.009907	3.06E-04	5.99E-04	0.983	0.017
H069	0.041517	0.033259	0.007759	3.86E-04	3.60E-05	0	0.005608	0	0.931	0.069
H072	0.02007	0.036571	0.006669	0.00105	0	0.026771	0.004726	3.90E-05	1	0
H077	0.027036	0.044522	0.002071	0.00215	0	0.004685	6.73E-04	0	0.949	0.051
H080	0.054242	0.066895	9.77E-04	0.00461	1.05E-04	0.003855	0.001271	0	0.915	0.085
H081	0.064731	0.028841	0.082122	0	3.59E-04	0	2.71E-04	0.001313	0.938	0.062
H082	0.019448	0.076093	0.020501	0.00148	5.10E-05	0.010885	0.001145	9.00E-05	1	0
H085	0.001903	0.004208	6.11E-04	0.00286	1.43E-04	2.13E-04	0.006572	8.00E-05	0.632	0.368
H087	0.042176	0.081204	0.018355	2.85E-04	2.80E-05	0.002593	5.00E-05	1.16E-04	0.889	0.111
H095	0.045726	0.011381	0.022788	0.00111	6.10E-05	0.023076	0.009942	5.83E-04	0.994	0.006
H101	0.085557	0.010443	0.018227	0.00587	1.89E-04	0.001382	2.84E-04	3.68E-04	0.991	0.009
H103	0.013931	0.022714	0.004602	0.01957	1.88E-04	0.002177	0.001492	5.00E-05	0.996	0.004
H105	0.114719	0.058608	0.031405	0.00187	0.001238	0.035216	4.83E-04	0.008984	0.992	0.008
H106	0.02143	0.037794	0.011999	0.07137	3.10E-05	0.008041	0.004013	2.88E-04	1	0
H111	0.062386	0.191038	0.075805	0.0034	1.10E-04	0.00469	0.00108	2.84E-04	1	0
H112	0.022003	0.113452	0.010024	0.0035	9.80E-05	0.005495	0.056831	2.96E-04	1	0
H115	0.072928	0.011631	0.002532	0	4.20E-05	0	0	5.20E-05	0.546	0.454
H117	0.010267	0.022254	0.003946	6.29E-04	2.90E-05	4.81E-04	2.98E-04	3.30E-05	0.972	0.028
H119	0.067987	0.034588	0.011457	0.01225	3.88E-04	0.03212	0.006484	4.11E-04	1	0
H121	0.066062	0.031146	0.022248	0.0042	5.10E-05	0.006497	6.00E-05	0.001716	0.961	0.039
H129	0.015757	0.029164	0.008545	0.00665	2.90E-05	0.001142	0.007546	6.40E-05	1	0
H132	0.127402	0.026997	0.012314	0.0025	2.68E-04	0.023674	0	2.14E-04	0.82	0.18
H135	0.01611	0.019672	0.007281	3.90E-04	0	3.80E-04	0.008149	0	0.968	0.032
H136	0.013211	0.032018	0.016746	0	8.30E-05	8.49E-04	0.004003	0	0.882	0.118
H138	0.039867	0.017692	0.001023	0	5.70E-05	2.29E-04	0.022446	0	0.817	0.183
H140	0.153742	0.009267	0.012075	0.01851	0.001122	0.024807	1.39E-04	3.40E-05	0.898	0.102
H143	0.027558	0.032442	0.010157	0.11628	8.00E-05	0.008482	0.004635	9.40E-05	1	0
SC01	0	0.002882	0.004998	8.80E-05	5.52E-04	0	8.40E-05	0	0.224	0.776
SC02	0.050138	1.51E-04	0	0	0.00805	8.90E-05	0	0	0.008	0.992
SC03	0.030727	4.16E-04	0.037825	0	3.71E-04	5.90E-05	1.82E-04	0	0.179	0.821
SC04	0.016671	0.005919	5.15E-04	1.17E-04	5.09E-04	0	0	0	0.307	0.693
SC05	0.008697	0.001198	0.002188	0	3.70E-05	0	0	0	0.043	0.957
SC06	0.032839	0.007596	0.00352	0	0.376659	0.00485	0	5.00E-05	0.498	0.502
SC07	0.051337	0.0011	8.20E-05	0	0.002596	2.39E-04	0	0	0.011	0.989
SC08	0.036345	9.12E-04	0.010763	0	0.019049	6.68E-04	0	0	0.093	0.907
SC09	0.01888	0.009166	0.005236	0	0.026735	0.001671	0.007779	0	0.704	0.296
SC10	1.03E-04	9.13E-04	9.94E-04	0	0.009145	2.46E-04	0	0	0.003	0.997
SC11	0.015867	0.001197	0.01225	9.90E-05	0.022518	4.26E-04	2.20E-05	0	0.158	0.842
SC12	0.012805	9.93E-04	0.002793	0	0	0	6.50E-05	0	0.127	0.873
SC13	0.026613	2.29E-04	0.003805	0	0.003782	0.001907	0	0	0.063	0.937
SC14	0.016544	2.48E-04	0.00114	0	0.03635	5.46E-04	0	0	0.001	0.999
SC15	0.019523	9.50E-04	0.012488	0	2.42E-04	0	0	0	0.108	0.892
SC16	1.02E-04	1.20E-04	5.90E-05	0	0.001569	9.30E-05	0	0	0	1
SC17	0.002585	0.002658	0.001173	3.24E-04	6.10E-05	1.16E-04	0.001844	0	0.385	0.615
SC18	0.017317	7.06E-04	9.83E-04	6.79E-04	0.015286	2.45E-04	2.14E-04	0	0.179	0.821
SC19	0.010458	0.020077	2.82E-04	0	9.45E-04	0.034679	2.70E-05	0	0.539	0.461
SC20	0.051541	1.73E-04	6.19E-04	0	5.15E-04	0.007975	0	0	0.095	0.905
SC21	0.010624	6.40E-05	0.001517	0	3.31E-04	0.00165	0	0	0.065	0.935
SC22	0.014461	8.78E-04	0.008055	0	0.001395	8.30E-05	5.90E-05	0	0.126	0.874
SC23	0.048933	0.003893	0.001337	0	0	0.001114	0	0	0.21	0.79
SC24	0.156023	0.003124	0.010545	0	0	6.52E-04	0	0	0.357	0.643
SC25	0.009901	0.003111	0.00146	0.00588	0.031659	7.06E-04	8.30E-05	0	0.288	0.712
SC26	0.015818	1.49E-04	0.006246	0	7.11E-04	9.10E-05	0	0	0.073	0.927
SC27	0.019062	0.044221	5.16E-04	0	4.12E-04	0	0	0	0.516	0.484
SC28	0.012866	0.013502	0.016746	1.03E-04	6.70E-05	0.005797	0	0	0.637	0.363
SC29	1.60E-05	3.08E-04	8.48E-04	0	1.11E-04	0	0	7.40E-05	0.042	0.958

SC30	0.022841	6.30E-05	0.001888	5.19E-04	0.006572	2.30E-05	3.20E-05	0	0.082	0.918
SC31	0	3.10E-05	0.004075	0	0.05222	3.10E-05	0	1.16E-04	0.024	0.976
SC32	0.019692	5.30E-05	0.003142	1.13E-04	0.025805	0	1.20E-05	0	0.034	0.966
SC33	0.007923	1.90E-05	8.92E-04	0	0.004746	0	0	0	0	1
SC34	2.70E-04	1.96E-04	4.75E-04	2.70E-05	0.009324	7.40E-05	0	2.60E-05	0.011	0.989
SC35	0.001144	6.10E-05	4.43E-04	0	0.03491	3.94E-04	6.10E-05	0	0.011	0.989
SC36	0.105842	8.90E-05	0.001824	0	4.64E-04	0.064502	0	0	0.147	0.853

Table S11. The levels of Ig-G antibody		
IgG levels	Number	Group
0.78	H-001	H
1.56	H-002	H
0.74	H-003	H
0.37	H-004	H
0.38	H-005	H
2.31	H-006	H
10.13	GY-704	SCR
11.39	GY-701	SCR
18.75	GY-712	SCR
25.48	GY-702	SCR
47.26	GY-703	SCR
23.75	GY-713	SCR
18.86	GY-714	SCR
41.96	GY-715	SCR
54.28	GY-716	SCR
313.62	GY-718	SCR
13.03	GY-719	SCR
17.21	GY-709	SCR
29.31	GY-721	SCR
13.86	GY-726	SCR
341.49	GY-727	SCR
39.75	GY-728	SCR
23.57	GY-708	SCR
15.64	GY-731	SCR
10.11	GY-724	SCR
15.26	GY-717	SCR
57.27	GY-732	SCR
31.58	GY-734	SCR
19.64	GY-736	SCR
21.29	GY-720	SCR
25.16	GY-725	SCR
48.14	GY-710	SCR
11.27	GY-729	SCR
22.74	GY-711	SCR
87.76	GY-737	SCR
308.06	GY-730	SCR
43.96	GY-723	SCR
45.64	GY-705	SCR
37.46	GY-733	SCR
21.57	GY-735	SCR
327.93	GY-706	SCR
13.67	GY-707	SCR
451.37	GY-801	CCR
214.03	GY-802	CCR
524.97	GY-803	CCR
282.51	GY-804	CCR
973.87	GY-805	CCR
848.58	GY-806	CCR
147.04	GY-807	CCR
112.88	GY-808	CCR
420.79	GY-809	CCR
188.59	GY-810	CCR
230.16	GY-811	CCR
336.60	GY-813	CCR
254.92	GY-814	CCR

247.17	GY-815	CCR
201.13	GY-816	CCR
122.54	GY-817	CCR
446.07	GY-818	CCR
448.21	GY-819	CCR
279.65	GY-820	CCR
312.12	GY-821	CCR
225.38	GY-822	CCR

Supplementary table S12. The detailed values of oral microbial diversity index and observed OTUs among CC (n=73), SC (n=36), and H (n=140) groups.								
sample	reads	label	ace	chao	shannon	simpson	coverage	observed_otus
CC01	7455	0.97	135.608	147.5	2.524982	0.160715	0.995842	101
CC02	44451	0.97	262.705	237.0667	2.849096	0.099143	0.998943	165
CC03	48453	0.97	230.535	224.12	2.341045	0.195993	0.999113	188
CC04	34734	0.97	192.784	206.9286	2.769604	0.107754	0.998877	154
CC05	31376	0.97	126.918	133.1429	2.582905	0.13304	0.999363	106
CC06	54380	0.97	173.429	170.2353	3.030465	0.07619	0.999559	154
CC07	25172	0.97	159.026	154.3125	2.248837	0.264487	0.998967	134
CC08	36229	0.97	189.743	159.5833	2.179127	0.173118	0.999034	110
CC09	29623	0.97	241.633	228	3.156719	0.078491	0.99865	168
CC10	35071	0.97	279.369	284.913	3.429069	0.064269	0.998489	225
CC11	27951	0.97	219.866	190.8333	2.489673	0.156193	0.998497	143
CC12	47479	0.97	272.714	212.4	1.342442	0.467968	0.998863	117
CC13	19591	0.97	291.683	243	2.391576	0.220811	0.997091	167
CC15	22672	0.97	170.484	110	2.506587	0.107933	0.998941	64
CC16	65473	0.97	393.698	408.4286	2.477593	0.185261	0.998839	327
CC17	45610	0.97	485.689	490.0227	3.884058	0.042297	0.998268	420
CC18	113725	0.97	344.157	358.2727	3.34938	0.075944	0.999578	307
CC19	12117	0.97	91.0936	93.33333	1.046064	0.627054	0.998267	70
CC20	52561	0.97	367.719	316.12	2.557275	0.221046	0.998706	225
CC21	42544	0.97	230.358	264.5455	3.22275	0.073994	0.999036	190
CC22	8304	0.97	180.691	184.1538	3.443264	0.064305	0.995906	141
CC23	16827	0.97	188.34	179.3913	2.434734	0.181119	0.997979	155
CC24	53682	0.97	293.158	293.12	3.201019	0.105425	0.999106	248
CC25	8343	0.97	226.185	218.6364	3.438151	0.064971	0.995206	195
CC26	26851	0.97	351.889	294.8889	2.118671	0.351655	0.997505	213
CC27	48813	0.97	319.578	323.5556	3.007876	0.166827	0.998996	280
CC28	16935	0.97	252.228	250.913	2.779329	0.157222	0.99687	191
CC29	55341	0.97	320.327	323.5652	3.415581	0.070506	0.999006	259
CC30	25987	0.97	254.251	250.037	3.444605	0.059912	0.998653	228
CC31	17336	0.97	208.825	210.0476	1.939938	0.398615	0.997635	171
CC32	13496	0.97	160.406	159.1429	1.893053	0.331	0.997703	137
CC33	16698	0.97	215.628	227.5556	2.338563	0.235778	0.997365	175
CC34	43841	0.97	317.793	335.0455	3.870457	0.035621	0.998768	270
CC35	11676	0.97	153.739	140.5	1.504863	0.449136	0.997345	94
CC36	64763	0.97	433.069	424.1364	3.449841	0.064589	0.998688	343
CC37	14751	0.97	252.834	254.12	3.516755	0.069812	0.997424	226
CC38	13135	0.97	213.888	153.9286	1.874375	0.237637	0.997031	101
CC39	32572	0.97	236.159	237.3846	1.888748	0.342543	0.998649	201
CC40	60581	0.97	240.275	195.625	2.983687	0.120696	0.999389	154
CC41	8255	0.97	229.281	275	2.934521	0.124152	0.993701	173
CC42	14069	0.97	139.187	120.8125	2.401164	0.152428	0.998365	105
CC43	76210	0.97	286.901	280.2286	2.655941	0.105055	0.999239	233
CC44	18457	0.97	281.515	290.1364	3.525344	0.068021	0.997454	241
CC45	23573	0.97	320.827	332.2917	3.166044	0.159358	0.997497	261
CC46	9778	0.97	222.136	214.6	2.806427	0.147233	0.995398	175
CC47	10638	0.97	196.432	160.5882	1.758022	0.338065	0.996334	117
CC48	49094	0.97	177.551	168.1364	2.511787	0.111266	0.999144	129
CC49	17850	0.97	159.54	162.0833	2.823122	0.13279	0.998543	135
CC50	13881	0.97	153.605	122.1	1.538265	0.435624	0.998055	87
CC51	41266	0.97	254.65	243.6364	2.15015	0.259824	0.998764	205
CC52	47153	0.97	406.804	405.0714	3.724811	0.050097	0.998855	371
CC53	47576	0.97	281.572	252.3529	2.606173	0.182189	0.998991	186
CC54	20934	0.97	239.769	221.2857	3.458245	0.059758	0.998089	199
CC55	43493	0.97	168.931	181.5455	2.875555	0.081591	0.999149	121
CC56	24012	0.97	212.63	192.4	3.097301	0.079944	0.998459	148
CC57	37653	0.97	419.01	421.1628	3.641364	0.07791	0.998141	365
CC58	19236	0.97	257.303	276.5882	3.052715	0.123426	0.997557	213
CC59	51820	0.97	268.646	261.9375	2.981119	0.096204	0.999132	231
CC60	18762	0.97	231.801	230.3704	2.577832	0.254973	0.997815	200
CC61	51264	0.97	223.914	212.0333	2.846164	0.09038	0.999083	176
CC62	26361	0.97	218.952	229.1765	2.506215	0.178341	0.998596	190
CC63	34039	0.97	244.584	237	2.759314	0.154714	0.998825	211
CC64	84521	0.97	275.777	226.4	0.581201	0.813159	0.999349	167
CC65	42728	0.97	212.46	206.125	1.856474	0.424501	0.999298	188
CC66	40352	0.97	318.536	322	3.219454	0.087534	0.998587	265
CC67	30156	0.97	262.215	265.4167	2.434043	0.307891	0.998541	226
CC68	15742	0.97	219.654	230.1667	2.740964	0.147758	0.997268	180
CC69	30088	0.97	240.154	226.5517	2.711422	0.134943	0.998704	201
CC70	48825	0.97	264.104	244.0667	2.665516	0.207362	0.999099	181
CC71	65325	0.97	207.556	209	2.92801	0.100844	0.999388	170
CC72	35789	0.97	283.972	298.3333	3.498472	0.062326	0.998603	240
CC73	35906	0.97	220.872	213.4444	2.273064	0.238135	0.998914	186
CC74	73418	0.97	171.979	210.2	2.954036	0.120552	0.999673	155
SC01	26648	0.97	233.011	246.0667	2.918566	0.15492	0.998349	183
SC02	39458	0.97	304.18	252.5652	2.902501	0.095814	0.998606	188
SC03	27989	0.97	325.323	310.5263	3.471199	0.07424	0.997999	270

SC04	31489	0.97	263.648	270	3.350013	0.074105	0.998476	223	
SC05	28731	0.97	224.515	204.7143	1.520976	0.469944	0.998434	134	
SC06	65617	0.97	210.957	211.4737	2.793536	0.162908	0.999589	193	
SC07	24848	0.97	210.234	219.125	2.990125	0.088547	0.998229	160	
SC08	38019	0.97	383.357	379.3947	3.724552	0.044648	0.998133	314	
SC09	32837	0.97	274.289	278.875	3.539166	0.057331	0.998721	243	
SC10	29086	0.97	177.505	177.25	2.873281	0.140802	0.999175	160	
SC11	78036	0.97	426.63	418.5957	3.821947	0.041186	0.999295	387	
SC12	17233	0.97	172.764	165.375	2.143685	0.302378	0.998201	146	
SC13	33387	0.97	347.388	355.8889	3.578498	0.054449	0.997993	274	
SC14	32459	0.97	352.434	370.3846	3.650344	0.065759	0.998121	300	
SC15	14771	0.97	262.546	266.04	3.442031	0.087543	0.996683	219	
SC16	32130	0.97	162.049	134.4615	1.878379	0.331453	0.999066	101	
SC17	47136	0.97	345.236	351.6897	3.776821	0.048219	0.999024	316	
SC18	36901	0.97	392.519	380.7179	3.363405	0.101914	0.998076	317	
SC19	37975	0.97	284.155	293.0435	3.427103	0.065895	0.998736	244	
SC20	25013	0.97	263.897	272	3.491889	0.057914	0.998281	229	
SC21	32058	0.97	153.058	146.8125	2.253267	0.26814	0.999283	131	
SC22	29700	0.97	262.346	244.8824	2.783632	0.165372	0.998451	184	
SC23	25753	0.97	246.302	240.25	3.393229	0.060079	0.99833	208	
SC24	43010	0.97	244.425	255.5556	3.318728	0.070518	0.999047	210	
SC25	48047	0.97	371.445	382.3333	4.037787	0.043611	0.999043	344	
SC26	36399	0.97	273.958	271.5333	3.122191	0.11065	0.998791	240	
SC27	31789	0.97	301.985	266.5556	3.29822	0.091428	0.998459	223	
SC28	29905	0.97	305.079	296	3.332877	0.101211	0.998127	241	
SC29	39693	0.97	160.967	152.8824	2.120597	0.295816	0.999269	129	
SC30	28121	0.97	338.927	335.5676	3.471624	0.090234	0.998435	310	
SC31	40581	0.97	257.218	190.3333	2.76832	0.109273	0.99899	122	
SC32	57303	0.97	382.054	391.2632	3.243782	0.083302	0.998744	324	
SC33	46286	0.97	317.719	268.7727	2.356783	0.195008	0.998725	191	
SC34	29757	0.97	291.189	282.1	3.496076	0.051985	0.998051	227	
SC35	54487	0.97	289.358	290.4063	2.930003	0.117401	0.998991	244	
SC36	21175	0.97	257.721	256.1154	3.080699	0.084339	0.997403	199	
H002	44182	0.97	418.714	416	3.699402	0.057874	0.998302	341	
H003	25256	0.97	344.936	339.3415	3.676188	0.052229	0.997941	307	
H004	46291	0.97	380.907	328.129	3.083469	0.085105	0.998401	241	
H005	46124	0.97	355.862	372.5556	3.494152	0.058567	0.998352	267	
H006	33928	0.97	459.301	467	3.801187	0.059782	0.997613	386	
H008	50924	0.97	403.438	360.2222	3.417499	0.053912	0.998468	249	
H009	40078	0.97	415.332	422.4103	3.914814	0.046382	0.998303	364	
H010	33172	0.97	408.103	407.3171	3.882001	0.043362	0.998252	367	
H011	32252	0.97	446.004	444.4667	4.143309	0.03684	0.998233	409	
H013	23478	0.97	311.297	321.25	3.302177	0.089915	0.997402	245	
H014	23771	0.97	254.276	250	3.393336	0.059212	0.997897	201	
H015	39861	0.97	359.012	340.0556	3.526607	0.058624	0.99852	245	
H016	30321	0.97	303.89	294.0345	3.34806	0.074098	0.99812	239	
H017	29082	0.97	345.604	359.3571	2.65418	0.250202	0.997902	294	
H018	29803	0.97	437.816	369.6512	3.594266	0.052852	0.997349	298	
H019	47629	0.97	372.11	317.7	3.488568	0.051559	0.998593	244	
H020	52408	0.97	379.994	375.8	3.649874	0.053269	0.998779	331	
H021	44461	0.97	440.36	437.5	4.1995	0.031625	0.998695	394	
H023	27682	0.97	354.837	347	3.805607	0.040967	0.997544	280	
H024	23929	0.97	440.036	483.7308	3.361027	0.145198	0.996866	377	
H025	49918	0.97	608.646	621.7213	3.896896	0.044358	0.997836	527	
H026	41157	0.97	535.015	527.2273	3.864113	0.052862	0.997716	461	
H027	26320	0.97	521.341	528.7255	3.933518	0.062305	0.996847	462	
H028	25712	0.97	419.29	455.1071	4.129717	0.031324	0.997083	356	
H029	33946	0.97	589.232	593.9828	4.438287	0.026878	0.997201	517	
H030	46722	0.97	604.197	621.1702	4.375987	0.02844	0.998202	547	
H031	45772	0.97	566.718	563.1719	3.949698	0.062979	0.998187	510	
H032	36906	0.97	541.295	547.7333	4.623533	0.020207	0.997887	481	
H033	23635	0.97	436.717	439.2195	4.15068	0.042533	0.997081	382	
H034	33433	0.97	488.732	526.7813	4.245549	0.030613	0.997547	423	
H035	50620	0.97	440.124	431.9783	3.700649	0.042183	0.99836	358	
H036	17126	0.97	289.937	271.875	3.446191	0.076332	0.996672	222	
H038	34885	0.97	324.08	364.55	3.624085	0.047539	0.998223	270	
H039	38270	0.97	374.103	364.9231	3.652344	0.049028	0.998171	303	
H040	27833	0.97	322.345	325	3.979503	0.031803	0.998096	272	
H041	33245	0.97	269.247	293.5	3.36575	0.057955	0.998376	214	
H042	39412	0.97	361.941	375.6	3.300093	0.081121	0.998148	288	
H043	43817	0.97	327.286	337.24	3.603384	0.051772	0.998768	280	
H044	36188	0.97	467.472	407.8333	3.384362	0.063624	0.99743	289	
H045	31363	0.97	403.314	399.3171	3.853847	0.046701	0.997896	347	
H046	29779	0.97	286.631	285.3333	3.061827	0.09883	0.998119	234	
H047	26875	0.97	436.087	427.32	3.603722	0.05671	0.996688	349	
H048	34909	0.97	367.018	371.0286	3.40195	0.072673	0.998023	304	
H049	41981	0.97	380.681	383.303	3.658843	0.052274	0.998309	308	
H050	45510	0.97	452.463	402.1622	4.000684	0.033242	0.99844	335	
H051	56479	0.97	500.79	508	3.949736	0.041336	0.998495	423	
H052	44502	0.97	381.8	394.6176	3.019068	0.115406	0.998315	313	

H053	8717	0.97	309.071	333.3913	4.037103	0.04129	0.993232	259	
H054	13490	0.97	435.494	444.3714	4.808713	0.017356	0.996071	405	
H055	13119	0.97	341.252	358.3913	4.274174	0.029492	0.995655	289	
H056	34856	0.97	375.302	378.0294	3.614478	0.052626	0.997992	307	
H057	40713	0.97	341.246	342.0333	3.526476	0.067303	0.998477	279	
H058	10079	0.97	368.96	389	4.544121	0.023236	0.994543	334	
H059	12286	0.97	311.855	319.84	4.235218	0.029548	0.996419	282	
H060	29501	0.97	412.014	422.2	4.061236	0.042815	0.998339	383	
H061	23259	0.97	309.825	303.2286	3.299347	0.084408	0.997506	256	
H062	44382	0.97	452.62	462.3636	4.074878	0.041091	0.998783	419	
H063	45742	0.97	348.873	363.8889	3.077113	0.143506	0.998492	277	
H064	38339	0.97	372.879	385.5172	3.112837	0.106887	0.997861	271	
H065	52089	0.97	406.936	411.6923	3.723511	0.048696	0.998771	360	
H066	26537	0.97	396.481	408.3333	3.949891	0.070075	0.998267	370	
H067	27830	0.97	311.578	306.8485	3.2147	0.088234	0.99788	255	
H068	37090	0.97	392.969	403	2.897433	0.173494	0.997924	326	
H069	26283	0.97	335.185	354.4	3.394113	0.09502	0.997299	255	
H070	26930	0.97	330.703	336.9643	3.310997	0.082436	0.997512	258	
H071	30209	0.97	435.281	439.2174	3.904785	0.035978	0.997087	356	
H072	27873	0.97	335.972	338.0938	3.813318	0.05264	0.997776	279	
H073	36445	0.97	396.377	405.1765	3.840216	0.04214	0.998216	344	
H074	31459	0.97	299.52	290.0333	2.976409	0.123787	0.998029	227	
H075	37042	0.97	372.246	371.6923	3.362193	0.072541	0.998272	320	
H076	40083	0.97	436.306	394.8966	3.587233	0.070594	0.998079	294	
H077	23011	0.97	327.685	329.2759	2.850233	0.17234	0.996958	246	
H078	25401	0.97	344.552	352.1	3.876766	0.039019	0.99752	287	
H079	27934	0.97	301.749	297.1034	3.631062	0.047899	0.997995	244	
H080	26245	0.97	433.002	380.4375	3.643722	0.048642	0.997066	289	
H081	25087	0.97	355.891	342.5	3.369157	0.065979	0.99721	290	
H082	42683	0.97	374.295	363.3846	3.696568	0.053222	0.99829	296	
H083	44640	0.97	413.799	421.0263	3.500399	0.067822	0.99832	348	
H084	40666	0.97	388.421	402.303	3.222985	0.095712	0.998254	327	
H085	35737	0.97	333.607	332.1154	3.977446	0.033302	0.998461	275	
H086	35797	0.97	361.596	367.3125	3.62004	0.068939	0.998324	312	
H087	44050	0.97	353.82	327.4444	3.090671	0.092125	0.998502	248	
H088	39983	0.97	380.437	383.5294	3.611452	0.068028	0.998674	343	
H089	26372	0.97	350.856	383.1818	3.622669	0.065463	0.997687	300	
H090	21110	0.97	274.731	275.0357	3.533686	0.052846	0.997395	222	
H091	27107	0.97	416.521	417.1538	4.217094	0.030342	0.997455	357	
H092	24318	0.97	363.402	391.5769	3.847173	0.042134	0.99708	296	
H093	31004	0.97	321.178	320.125	3.393301	0.07095	0.998194	272	
H094	29782	0.97	330.54	360.9545	3.729384	0.044471	0.997918	275	
H095	33111	0.97	482.117	438.0968	3.781852	0.042573	0.997252	306	
H096	39006	0.97	417.023	413.3415	3.541915	0.065407	0.998154	351	
H097	23754	0.97	314.858	318.0333	3.393218	0.095713	0.99739	255	
H098	50077	0.97	311.686	314.6774	3.698655	0.038649	0.998882	265	
H099	39062	0.97	409.711	415.303	3.995593	0.047114	0.998413	358	
H100	38412	0.97	324.289	322.1935	3.544484	0.047589	0.998282	253	
H101	44812	0.97	348.523	348.0938	2.965165	0.131932	0.998505	279	
H102	25711	0.97	295.878	299.0385	3.479141	0.071852	0.998016	250	
H103	27776	0.97	356.688	340.1667	3.718208	0.063718	0.99784	291	
H104	37727	0.97	398.27	398	3.796227	0.044463	0.998622	364	
H105	32470	0.97	416.429	421.3636	4.064943	0.038265	0.998337	378	
H106	47327	0.97	445.156	450.0789	3.857419	0.045856	0.998331	369	
H107	38798	0.97	415.287	416.2778	4.062118	0.03387	0.998634	378	
H108	33537	0.97	360.304	360.7568	3.555296	0.061401	0.998002	301	
H109	43593	0.97	378.784	389.6563	3.453687	0.069093	0.998371	312	
H110	40098	0.97	446.003	433.5625	3.948802	0.042391	0.998055	371	
H111	41856	0.97	356.16	376.4643	3.288216	0.077505	0.998232	280	
H112	34330	0.97	342.403	358.6154	3.787234	0.043023	0.998019	271	
H113	42796	0.97	403.284	404.6923	4.042916	0.036855	0.998738	368	
H114	65401	0.97	367.055	373	3.60635	0.047148	0.998991	308	
H115	22659	0.97	297.482	260.625	3.549059	0.044281	0.997617	201	
H116	54448	0.97	375.614	398.1379	3.751188	0.042729	0.998678	310	
H117	31384	0.97	320.087	313.4688	3.410102	0.074112	0.99812	260	
H118	35534	0.97	383.349	388.0769	3.686999	0.048241	0.998227	338	
H119	44373	0.97	487.127	482.5849	3.701022	0.061269	0.998039	412	
H120	34403	0.97	392.993	409.9412	3.276819	0.092451	0.997675	317	
H121	41867	0.97	455.626	443.5645	3.118505	0.141777	0.998041	390	
H122	47318	0.97	442.627	426.1957	3.312569	0.106497	0.998267	354	
H123	37402	0.97	441.757	445.878	3.72312	0.0508	0.997567	346	
H124	38914	0.97	512.229	476.3714	3.876637	0.047911	0.997739	367	
H125	13782	0.97	385.463	402.8333	4.247319	0.028965	0.994848	320	
H126	41427	0.97	421.652	425.0857	4.163017	0.033719	0.998359	360	
H127	24349	0.97	503.061	378.3714	3.828937	0.037888	0.996386	269	
H129	30574	0.97	505.67	519.0227	4.163402	0.033715	0.996795	411	
H130	26281	0.97	337.651	326.7174	3.630579	0.056549	0.998059	299	
H131	49287	0.97	407.459	400.5	4.044654	0.042099	0.998864	362	
H132	26742	0.97	343.336	336.625	3.459161	0.071901	0.997532	283	
H133	33174	0.97	357.812	380.2	3.505306	0.070095	0.99792	302	

H135	24873	0.97	269.392	267.3704	3.823605	0.041312	0.998352	237	
H136	33568	0.97	293.957	312.9	3.730852	0.040577	0.998421	244	
H137	35792	0.97	358.689	395.5	3.356914	0.101934	0.997988	289	
H138	45762	0.97	375.291	403.7778	3.551026	0.057076	0.998361	301	
H139	51560	0.97	319.685	355.3333	3.396349	0.064285	0.998836	257	
H140	52568	0.97	348.28	369	3.910153	0.048043	0.999144	314	
H141	51274	0.97	367.746	368.0278	3.976094	0.029779	0.999025	334	
H142	41740	0.97	346.227	359.4	3.90542	0.04003	0.998682	300	
H143	24063	0.97	351.815	338.4667	3.841079	0.043106	0.997631	303	
H146	41051	0.97	384.942	353.12	3.313343	0.073508	0.998222	248	
H147	22931	0.97	309.349	299.0263	3.070559	0.141815	0.997427	254	
H149	31417	0.97	371.937	384.4839	3.122366	0.109518	0.997836	311	
H150	32212	0.97	287.434	285.6774	3.439678	0.066554	0.998262	236	

Table S3 . The abundance and composition at the phylum level of each sample

ID	CC19	CC35	CC50	CC74	CC05	CC53	CC48	CC24	CC49	CC42	CC22	CC34
Bacteroidota	0.921853	0.873214	0.864062	0.690353	0.631806	0.519472	0.456594	0.433992	0.418733	0.41641	0.399973	0.352449
Firmicutes	0.014768	0.021146	0.023426	0.196728	0.249519	0.2242	0.319428	0.267657	0.4059	0.518243	0.389522	0.351474
Proteobacteria	0.00989	0.002372	0.001428	0.001297	0.001059	0.002482	0.108138	0.028773	0.001811	0.030897	0.027822	0.077244
Fusobacteriota	0.051388	0.001554	7.82E-04	0.002572	0.026317	0.140642	4.26E-04	0.160437	0.031742	0.008032	0.027323	0.081686
Actinobacteriota	0.001001	0.09973	0.108966	0.092706	0.003416	0.110429	0.033683	0.079484	0.139773	0.0222	0.147428	0.053422
Patescibacteria	0	4.25E-04	7.09E-04	0.005671	0.084137	9.00E-05	1.10E-04	0.00925	8.61E-04	7.30E-05	0.005246	0.08146
Campilobacterota	0.0011	5.32E-04	2.73E-04	0.010452	0.003707	0.002067	0.08024	0.019561	4.57E-04	0.001364	0	6.63E-04
Spirochaetota	0	0	0	0	0	3.77E-04	5.90E-05	6.10E-04	0	0	0	8.70E-04
Bacteria_unclassified	0	0.001027	3.54E-04	2.21E-04	3.90E-05	2.21E-04	0	2.36E-04	7.23E-04	0.002781	0.002686	6.67E-04
Synergistota	0	0	0	0	0	2.00E-05	8.00E-05	0	0	0	0	4.30E-05
Desulfobacterota	0	0	0	0	0	0	0	0	0	0	0	0
Cyanobacteria	0	0	0	0	0	0	0.001229	0	0	0	0	2.20E-05
Deferribacterota	0	0	0	0	0	0	0	0	0	0	0	0
Verrucomicrobiota	0	0	0	0	0	0	0	0	0	0	0	0
Deinococcota	0	0	0	0	0	0	0	0	0	0	0	0
Chloroflexi	0	0	0	0	0	0	1.30E-05	0	0	0	0	0
Elusimicrobiota	0	0	0	0	0	0	0	0	0	0	0	0
Planctomycetota	0	0	0	0	0	0	0	0	0	0	0	0

CC06	CC47	CC23	CC33	CC56	CC66	CC72	CC02	CC17	CC01	CC29	CC40	CC41
0.338354	0.333713	0.326473	0.316854	0.315027	0.313392	0.290189	0.290114	0.283298	0.262959	0.260744	0.244023	0.241712
0.506251	0.069915	0.074038	0.169179	0.26671	0.505618	0.137359	0.400847	0.370691	0.215858	0.338671	0.222987	0.131367
5.62E-04	0.006377	0.504283	0.00754	0.001747	0.039708	0.230602	0.006158	0.02638	0.011473	0.025192	0.40543	0.422125
0.077087	0.56602	0.071604	0.473309	0.21581	0.053006	0.266238	0.084911	0.23642	0.491865	0.102983	0.049642	0.171937
0.027887	0.022633	0.022696	0.027924	0.19845	0.068962	0.036974	0.216533	0.057496	0.015924	0.268471	0.048174	0.029936
0.048848	7.46E-04	1.18E-04	0.001157	0.00182	0.015969	0.02146	0	0.007574	1.41E-04	0.00162	0.002381	0.001082
5.51E-04	4.96E-04	3.59E-04	0.002941	4.80E-05	0.002173	0.01504	0.001161	0.014136	0.001503	0.001288	0.027255	0.001475
3.60E-05	0	0	5.20E-05	0	9.10E-05	0.001853	0	0.002643	0	1.86E-04	0	0
2.23E-04	1.00E-04	4.29E-04	9.83E-04	3.88E-04	9.63E-04	2.85E-04	2.30E-04	0	2.77E-04	8.45E-04	0	3.66E-04
0	0	0	6.10E-05	0	9.30E-05	0	2.10E-05	4.00E-05	0	0	0	0
0	0	0	0	0	2.50E-05	0	0	4.21E-04	0	0	0	0
2.01E-04	0	0	0	0	0	0	2.50E-05	7.84E-04	0	0	2.00E-05	0
0	0	0	0	0	0	0	0	0	0	0	0	0
0	0	0	0	0	0	0	0	1.17E-04	0	0	0	0
0	0	0	0	0	0	0	0	0	0	0	8.80E-05	0
0	0	0	0	0	0	0	0	0	0	0	0	0
0	0	0	0	0	0	0	0	0	0	0	0	0
0	0	0	0	0	0	0	0	0	0	0	0	0

CC44	CC10	CC18	CC61	CC08	CC54	CC13	CC45	CC09	CC69	CC31	CC36	CC70
0.241059	0.220782	0.200461	0.199956	0.199607	0.182296	0.176666	0.169348	0.162083	0.150511	0.147885	0.142954	0.137917
0.316538	0.311402	0.544027	0.567355	0.229322	0.343802	0.793284	0.655691	0.298482	0.108681	0.066801	0.38125	0.186543
0.12563	0.298103	0.011291	0.144729	0.001843	0.002101	0.009209	0.076863	0.012454	0.58166	0.003399	0.294753	0.506215
0.205862	0.021313	0.016916	0.011648	0.328386	0.175267	0.00335	0.047069	0.490728	0.111105	0.760612	0.086162	0.067989
0.099151	0.138582	0.203786	0.056741	0.240238	0.277798	0.015719	0.03678	0.015614	0.043327	0.018071	0.080496	0.051263
0.009329	3.76E-04	0.007024	6.00E-05	4.75E-04	0.009165	9.70E-05	0.01046	0.01815	0.002328	9.97E-04	0.008532	0
0.001159	0.007767	0.003044	0.017476	1.29E-04	0.009002	5.67E-04	0.002383	0.001854	1.88E-04	9.27E-04	0.003262	0.048493
4.50E-05	0	0.008659	2.48E-04	0	5.30E-05	0	5.67E-04	5.71E-04	0	1.49E-04	0.001829	5.50E-05
0.001175	2.50E-05	0.003872	1.80E-05	0	5.16E-04	0.001014	7.56E-04	6.40E-05	0.0022	0.001159	1.18E-04	1.68E-04
0	2.90E-05	3.49E-04	2.20E-05	0	0	4.80E-05	8.30E-05	0	0	0	6.01E-04	0
0	4.70E-05	7.60E-05	0	0	0	0	0	0	0	0	1.40E-05	0
5.20E-05	1.03E-04	4.54E-04	0.001747	0	0	4.60E-05	0	0	0	0	1.50E-05	0.001357
0	0	0	0	0	0	0	0	0	0	0	0	0
0	0	0	0	0	0	0	0	0	0	0	0	0
0	0.001437	0	0	0	0	0	0	0	0	0	0	0
0	3.40E-05	1.80E-05	0	0	0	0	0	0	0	0	1.40E-05	0
0	0	2.30E-05	0	0	0	0	0	0	0	0	0	0
0	0	0	0	0	0	0	0	0	0	0	0	0

CC63	CC59	CC21	CC55	CC32	CC25	CC04	CC67	CC60	CC15	CC46	CC27	CC37
0.125488	0.120648	0.11492	0.110604	0.107462	0.106685	0.104568	0.104535	0.102859	0.10205	0.094827	0.09172	0.09149
0.126491	0.452282	0.392447	0.410301	0.04317	0.202289	0.623505	0.714336	0.113704	0.611093	0.575976	0.61675	0.467612
0.543555	0.252032	0.36411	0.025078	0.794214	0.380398	0.010805	0.052226	0.027913	0.006962	0.213953	0.075216	0.268461
0.173679	0.098101	0.101767	7.01E-04	0.049641	0.142144	0.234067	0.039041	0.746155	0.035479	0.015621	0.134845	0.030206
0.024862	0.059715	0.022997	0.440893	0.003378	0.165659	0.02426	0.078974	0.006053	0.236854	0.016924	0.034152	0.131426
0.005442	0.005009	0.002845	6.10E-05	0.001162	0.001162	0.001054	0.003452	0.001796	0	0.052738	0.039836	0.006167
1.10E-04	0.009843	7.23E-04	0.01097	1.47E-04	9.64E-04	0.001384	5.71E-04	6.90E-04	0.007317	0.014975	0.004742	0.001238
4.70E-05	0.001964	8.00E-05	0	0	0	4.00E-05	2.80E-05	3.86E-04	0	0.013663	0.002479	3.32E-04
2.51E-04	1.56E-04	0	0.001392	7.48E-04	6.99E-04	3.17E-04	0.006837	3.25E-04	0	0	8.70E-05	0.002927
4.90E-05	1.66E-04	0	0	0	0	0	0	1.19E-04	0	5.17E-04	9.10E-05	0
2.60E-05	0	0	0	0	0	0	0	0	0	0	0	1.41E-04
0	8.40E-05	1.11E-04	0	7.80E-05	0	0	0	0	2.45E-04	8.06E-04	8.20E-05	0
0	0	0	0	0	0	0	0	0	0	0	0	0
0	0	0	0	0	0	0	0	0	0	0	0	0
0	0	0	0	0	0	0	0	0	0	0	0	0
0	0	0	0	0	0	0	0	0	0	0	0	0
0	0	0	0	0	0	0	0	0	0	0	0	0
0	0	0	0	0	0	0	0	0	0	0	0	0

CC20	CC30	CC57	CC71	CC52	CC38	CC58	CC73	CC28	CC39	CC65	CC16	CC62
0.079142	0.075524	0.072782	0.072027	0.061072	0.056779	0.054398	0.043574	0.037912	0.03603	0.035886	0.035027	0.034775
0.144917	0.391522	0.450165	0.175333	0.375707	0.908252	0.414019	0.061818	0.501516	0.835837	0.095362	0.531666	0.103903
0.028545	0.147395	0.201596	0.228525	0.2079	0.001007	0.241567	0.780261	0.12839	0.019838	0.784173	0.358256	0.583046
0.067357	0.281668	0.096636	0.467851	0.109023	0.001617	0.227464	0.096746	0.062563	0.062122	0.066081	0.03988	0.170231
0.657619	0.092812	0.161464	0.036539	0.202202	0.032126	0.049149	0.010474	0.251326	0.032666	0.013706	0.017692	0.106721
0.021246	0.006469	0.010691	0.008532	0.011352	1.44E-04	0.010242	0.001829	0.004598	0.003556	0.004264	3.68E-04	5.73E-04
5.91E-04	0.003314	0.004602	0.010999	0.006945	0	6.62E-04	0.005156	3.34E-04	0.005491	2.02E-04	0.009846	3.21E-04
4.01E-04	1.61E-04	0.001276	1.34E-04	0.013007	0	0	5.00E-05	3.61E-04	0.001836	0	0.006074	3.40E-05
1.31E-04	0.001096	2.20E-04	6.00E-05	0	7.50E-05	0.002499	9.20E-05	0.012878	6.40E-05	3.26E-04	0	3.96E-04
0	3.90E-05	2.94E-04	0	0.003461	0	0	0	0	0.002253	0	1.16E-04	0
0	0	0	0	5.90E-05	0	0	0	6.30E-05	3.07E-04	0	3.20E-05	0
5.10E-05	0	3.00E-05	0	0.009272	0	0	0	5.90E-05	0	0	9.00E-04	0
0	0	0	0	0	0	0	0	0	0	0	0	0
0	0	2.44E-04	0	0	0	0	0	0	0	0	1.20E-05	0
0	0	0	0	0	0	0	0	0	0	0	5.50E-05	0
0	0	0	0	0	0	0	0	0	0	0	0	0
0	0	0	0	0	0	0	0	0	0	0	7.60E-05	0
0	0	0	0	0	0	0	0	0	0	0	0	0

CC11	CC68	CC03	CC43	CC07	CC26	CC51	CC64	CC12	ID...75	SC29	SC27	SC01
0.033308	0.024505	0.021677	0.019264	0.017214	0.011802	0.011603	0.003841	4.91E-04	Bacteroidota	0.642169	0.588912	0.58714
0.502566	0.490069	0.251949	0.551057	0.071974	0.842951	0.747773	0.986156	0.715048	Firmicutes	0.168359	0.268909	0.209807
0.001523	0.398277	0.656497	0.420238	0.087471	0.023441	0.08925	0.005044	0.114399	Proteobacteria	9.94E-04	0.051676	0.049565
0.039878	0.069469	0.018683	9.86E-04	0.601499	0.062671	0.049523	0.001424	0.140492	Fusobacteriota	0.133317	0.036733	0.054767
0.411446	0.011285	0.028801	0.006814	0.220511	0.056043	0.098239	0.002731	0.026673	Actinobacteriota	0.020645	0.025835	0.044362
0	0.003271	2.08E-04	4.40E-05	0.001004	4.37E-04	1.63E-04	3.54E-04	1.28E-04	Patescibacteria	0.032717	0.012372	0.053543
1.05E-04	0.001134	0.007067	8.10E-04	1.55E-04	0.001015	7.94E-04	6.90E-05	0	Campilobacterota	0.00175	0.006403	7.30E-05
0	1.33E-04	0.003218	1.50E-05	0	7.46E-04	0.002228	4.00E-05	0	Spirochaetota	0	0.008868	0
0.010514	0.00172	0	0	1.72E-04	7.08E-04	7.10E-05	3.41E-04	6.26E-04	Bacteria_unclassified	0	0	7.01E-04
0	0	2.20E-05	8.10E-05	0	1.45E-04	2.50E-05	0	4.70E-05	Synergistota	0	2.31E-04	4.20E-05
0	0	0	6.00E-06	0	4.10E-05	0	0	0	Desulfobacterota	0	6.10E-05	0
6.60E-04	1.37E-04	0.011878	6.05E-04	0	0	3.31E-04	0	0.001639	Cyanobacteria	4.90E-05	0	0
0	0	0	0	0	0	0	0	0	Deferribacterota	0	0	0
0	0	0	5.00E-05	0	0	0	0	0	Verrucomicrobiota	0	0	0
0	0	0	0	0	0	0	0	4.57E-04	Deinococcota	0	0	0
0	0	0	1.40E-05	0	0	0	0	0	Chloroflexi	0	0	0
0	0	0	1.60E-05	0	0	0	0	0	Elusimicrobiota	0	0	0
0	0	0	0	0	0	0	0	0	Planctomycetota	0	0	0

SC28	SC22	SC26	SC23	SC03	SC19	SC04	SC17	SC25	SC09	SC08	SC07	SC15
0.526802	0.50746	0.465444	0.445869	0.431008	0.420386	0.370219	0.340425	0.329093	0.319407	0.293641	0.25235	0.225179
0.328188	0.152709	0.251026	0.275459	0.268974	0.094856	0.293595	0.258849	0.210612	0.286321	0.307301	0.128943	0.248433
0.031895	0.03551	0.066421	0.095501	0.16174	0.027479	0.130264	0.018678	0.089701	0.216477	0.11971	0.262333	0.334903
0.035859	0.228976	0.199023	0.166707	0.058192	0.40167	0.110676	0.266504	0.213496	0.113897	0.115227	0.32326	0.114329
0.052709	0.055291	0.010139	0.005937	0.068658	0.017884	0.067993	0.05789	0.045114	0.049511	0.150337	0.031517	0.046631
0.01889	0.019038	0.001691	0.00107	0.006351	0.016346	0.018177	0.050919	0.03755	0.010059	0.011217	2.44E-04	0.02399
0.004535	8.85E-04	0.00513	0.008072	0.002736	0.009655	0.008079	0.005497	0.025686	0.003442	6.14E-04	9.48E-04	0.004177
6.51E-04	0	7.25E-04	6.08E-04	2.64E-04	0.0116	1.71E-04	5.28E-04	0.03959	6.16E-04	1.20E-04	1.17E-04	6.02E-04
3.07E-04	1.31E-04	3.73E-04	7.36E-04	0.001973	3.20E-05	7.64E-04	2.35E-04	5.50E-05	2.70E-04	0.001516	2.45E-04	0.001756
1.28E-04	0	0	4.10E-05	3.10E-05	5.70E-05	3.50E-05	3.75E-04	0.006104	0	8.00E-05	0	0
3.60E-05	0	0	0	0	0	0	1.00E-04	0.002883	0	1.87E-04	0	0
0	0	0	0	0	3.50E-05	2.70E-05	0	4.30E-05	0	0	0	0
0	0	0	0	0	0	0	0	0	0	0	0	0
0	0	0	0	7.30E-05	0	0	0	0	0	0	4.30E-05	0
0	0	0	0	0	0	0	0	0	0	0	0	0
0	0	2.80E-05	0	0	0	0	0	7.30E-05	0	5.00E-05	0	0
0	0	0	0	0	0	0	0	0	0	0	0	0
0	0	0	0	0	0	0	0	0	0	0	0	0

SC06	SC36	SC20	SC11	SC34	SC35	SC13	SC32	SC12	SC24	SC10	SC14	SC30
0.217926	0.20959	0.20498	0.183721	0.17712	0.173458	0.165833	0.147688	0.144067	0.133929	0.102173	0.096317	0.083641
0.129494	0.458129	0.274189	0.321798	0.470236	0.483262	0.292642	0.173681	0.136458	0.105914	0.594635	0.422609	0.483859
0.569677	0.141992	0.250578	0.101165	0.01488	0.07313	0.079435	0.252323	0.686476	0.556022	0.027478	0.339807	0.282506
0.070226	0.170242	0.242076	0.243214	0.128998	0.097347	0.410833	0.382375	0.005949	0.115963	0.200221	0.071265	0.085117
0.008953	0.011815	0.013767	0.12952	0.093708	0.167414	0.040196	0.026571	0.021945	0.063417	0.040082	0.056669	0.040571
0.001947	0.005394	0.006386	0.006334	0.114028	0.001281	0.002859	0.009665	0.002756	0.022724	0.033704	0.007268	0.010889
0.001662	0.001824	0.005347	0.009744	2.50E-05	5.04E-04	0.005955	0.005733	0.001763	0.00171	0.001034	0.004774	0.001504
1.15E-04	7.88E-04	0.002172	0.001411	2.80E-04	6.50E-05	0.001403	0.001406	5.70E-05	8.20E-05	1.04E-04	0.001108	0.005163
0	1.78E-04	5.05E-04	0.001093	5.68E-04	0.003498	6.78E-04	1.30E-04	5.29E-04	2.39E-04	5.35E-04	1.83E-04	5.24E-04
0	4.80E-05	0	3.67E-04	3.90E-05	0	1.66E-04	5.20E-05	0	0	0	0	0.003487
0	0	0	5.97E-04	0	0	0	1.70E-05	0	0	0	0	0.002739
0	0	0	0.00102	9.40E-05	2.20E-05	0	4.90E-05	0	0	3.40E-05	0	0
0	0	0	0	0	0	0	0	0	0	0	0	0
0	0	0	0	2.40E-05	1.90E-05	0	2.85E-04	0	0	0	0	0
0	0	0	0	0	0	0	0	0	0	0	0	0
0	0	0	1.60E-05	0	0	0	2.50E-05	0	0	0	0	0
0	0	0	0	0	0	0	0	0	0	0	0	0
0	0	0	0	0	0	0	0	0	0	0	0	0

SC31	SC18	SC05	SC02	SC33	SC16	SC21	ID...112	H042	H008	H019	H026	H060
0.079596	0.078966	0.078925	0.078186	0.076564	0.062597	0.062246	Bacteroidota	0.546506	0.516332	0.511812	0.504682	0.500047
0.53473	0.508753	0.037077	0.160492	0.448918	0.77758	0.771726	Firmicutes	0.292866	0.269977	0.276012	0.216853	0.199169
0.234843	0.240235	0.841345	0.158932	0.329953	0.002438	0.070557	Proteobacteria	0.02365	0.042386	0.028586	0.085732	0.144623
0.106256	0.131884	0.040538	0.586173	0.033996	2.29E-04	0.00174	Fusobacteriota	0.045164	0.087664	0.103299	0.081095	0.068718
0.043891	0.02908	0.001635	0.014007	0.103236	0.146489	0.086453	Actinobacteriota	0.057222	0.045484	0.044156	0.031973	0.05407
2.60E-05	0.004886	1.17E-04	2.20E-04	4.71E-04	3.60E-05	3.39E-04	Patescibacteria	0.017684	0.025031	0.012106	0.037209	0.007225
2.13E-04	0.001794	2.06E-04	0.001688	1.14E-04	0.001672	1.37E-04	Campilobacterota	0.015448	0.01277	0.023361	0.040304	0.016549
0	0.003001	5.10E-05	2.31E-04	2.78E-04	0	0	Spirochaetota	0.001238	3.19E-04	6.45E-04	6.32E-04	0.006695
0	2.56E-04	7.00E-05	7.10E-05	0.006183	0.008928	0.006802	Bacteria_unclassified	4.80E-05	0	2.30E-05	0	0
0	7.63E-04	0	0	1.18E-04	0	0	Synergistota	1.24E-04	1.90E-05	0	0	7.39E-04
2.30E-05	3.20E-04	3.60E-05	0	0	0	0	Desulfobacterota	2.30E-05	0	0	0.001345	0.00108
4.22E-04	0	0	0	1.69E-04	0	0	Cyanobacteria	0	0	0	8.30E-05	1.08E-04
0	0	0	0	0	0	0	Deferribacterota	2.70E-05	0	0	1.90E-05	0
0	6.20E-05	0	0	0	3.10E-05	0	Verrucomicrobiota	0	1.80E-05	0	7.30E-05	4.60E-04
0	0	0	0	0	0	0	Deinococcota	0	0	0	0	0
0	0	0	0	0	0	0	Chloroflexi	0	0	0	0	5.70E-05
0	0	0	0	0	0	0	Elusimicrobiota	0	0	0	0	0
0	0	0	0	0	0	0	Planctomycetota	0	0	0	0	4.60E-04

H112	H038	H056	H036	H073	H130	H146	H111	H035	H079	H113	H099	H106
0.490049	0.479683	0.478761	0.470398	0.463831	0.46382	0.459739	0.457614	0.457496	0.451127	0.446428	0.442603	0.440686
0.152914	0.208163	0.382462	0.244157	0.310908	0.355892	0.094322	0.184325	0.195689	0.321211	0.216618	0.181717	0.168739
0.156273	0.097417	0.010276	0.095981	0.057402	0.029692	0.129856	0.241468	0.168399	0.023133	0.195243	0.21858	0.201139
0.097798	0.126968	0.030088	0.136084	0.065351	0.064949	0.195881	0.062329	0.122693	0.105658	0.05705	0.069591	0.114041
0.047069	0.048492	0.075121	0.03405	0.050584	0.044956	0.044743	0.023845	0.016763	0.036639	0.035253	0.027344	0.024297
0.035142	0.030094	0.004429	0.013425	0.034308	0.034216	0.066904	0.026148	0.029743	0.040726	0.041476	0.027858	0.023071
0.019152	0.008378	0.010729	0.005488	0.010023	0.003309	0.008483	0.001831	0.00514	0.020756	0.006276	0.030779	0.017906
0.001331	6.48E-04	0.007453	2.42E-04	0.006492	0.002775	0	0.002213	0.003406	5.45E-04	0.001229	9.58E-04	0.009038
0	3.10E-05	0	5.20E-05	2.32E-04	7.30E-05	0	0	8.70E-05	2.50E-05	0	0	0
2.16E-04	1.26E-04	3.59E-04	5.70E-05	8.14E-04	3.18E-04	0	1.55E-04	5.49E-04	1.80E-04	0	9.80E-05	4.80E-04
2.20E-05	0	2.35E-04	6.60E-05	2.80E-05	0	3.10E-05	7.20E-05	3.50E-05	0	6.90E-05	0	3.17E-04
0	0	8.70E-05	0	0	0	0	0	0	0	3.27E-04	4.11E-04	0
0	0	0	0	0	0	0	0	0	0	0	0	0
3.40E-05	0	0	0	0	0	4.10E-05	0	0	0	3.10E-05	6.10E-05	1.60E-05
0	0	0	0	0	0	0	0	0	0	0	0	0
0	0	0	0	2.70E-05	0	0	0	0	0	0	0	1.50E-05
0	0	0	0	0	0	0	0	0	0	0	0	2.55E-04
0	0	0	0	0	0	0	0	0	0	0	0	0

H100	H125	H118	H071	H016	H029	H043	H103	H078	H030	H081	H143	H053
0.438478	0.436224	0.432748	0.429933	0.429421	0.427958	0.42748	0.425456	0.417197	0.410327	0.409268	0.407551	0.404875
0.313098	0.414038	0.180066	0.243989	0.295072	0.296156	0.253226	0.235738	0.195041	0.283069	0.170141	0.232256	0.38549
0.067327	0.023857	0.215612	0.084977	0.04848	0.167154	0.139252	0.100855	0.133218	0.090153	0.253368	0.123359	0.12694
0.078656	0.070044	0.106572	0.147451	0.172578	0.035893	0.124544	0.146932	0.135832	0.09322	0.110095	0.167659	0.003003
0.0326	0.028126	0.018754	0.020734	0.020573	0.03364	0.03361	0.035129	0.022896	0.079503	0.046697	0.021488	0.074938
0.055689	0.021976	0.039385	0.043359	0.024087	0.018169	0.008588	0.042001	0.08294	0.02446	0.005159	0.024834	0.002409
0.013671	0.00222	0.005327	0.027274	0.008187	0.013291	0.010136	0.005473	0.010084	0.011773	0.004677	0.003808	0
4.24E-04	0	0.001302	0.002097	0.001449	0.001982	0.002429	0.006339	0.002383	0.003896	3.59E-04	0.007333	0
0	0	0	0	5.80E-05	0	0	7.00E-05	1.18E-04	8.10E-05	0	1.86E-04	0
5.70E-05	0	1.05E-04	1.86E-04	6.70E-05	7.45E-04	3.76E-04	0.001367	2.91E-04	0.001525	0	0.008882	0
0	0.001389	4.30E-05	0	2.80E-05	0.003507	9.10E-05	5.68E-04	0	0.001004	3.70E-05	0.002351	0.001774
0	6.50E-04	0	0	0	4.14E-04	2.68E-04	0	0	9.28E-04	1.61E-04	0	1.14E-04
0	2.04E-04	0	0	0	0.001031	0	0	0	1.70E-05	0	0	0
0	0.001272	8.60E-05	0	0	2.80E-05	0	0	0	4.40E-05	3.80E-05	0	4.57E-04
0	0	0	0	0	0	0	0	0	0	0	0	0
0	0	0	0	0	3.20E-05	0	7.20E-05	0	0	0	2.93E-04	0
0	0	0	0	0	0	0	0	0	0	0	0	0
0	0	0	0	0	0	0	0	0	0	0	0	0

H094	H092	H041	H011	H126	H018	H040	H039	H009	H114	H080	H021	H105
0.404729	0.402828	0.401712	0.401447	0.400387	0.394644	0.394044	0.39256	0.391925	0.386043	0.382323	0.377446	0.3764
0.197276	0.22308	0.320728	0.300677	0.284056	0.169747	0.198584	0.270083	0.174967	0.171305	0.29463	0.228056	0.21866
0.19223	0.15896	0.013435	0.077027	0.091859	0.251006	0.199554	0.038379	0.140801	0.234813	0.207728	0.128066	0.269351
0.108712	0.149432	0.160239	0.058332	0.107858	0.115921	0.11157	0.251996	0.219141	0.101296	0.043105	0.163081	0.076789
0.044956	0.013398	0.048492	0.145928	0.040537	0.024913	0.049603	0.025264	0.018669	0.081309	0.047863	0.050627	0.03723
0.040256	0.037262	0.05061	0.001578	0.020789	0.036986	0.037925	0.012478	0.034792	0.013121	0.01387	0.022167	0.006077
0.011032	0.008232	0.004784	0.012827	0.010683	0.005479	0.007409	0.007313	0.018928	0.011828	0.007263	0.019804	0.006921
5.92E-04	0.004738	0	0.00139	0.039131	0.001045	8.90E-04	9.01E-04	4.30E-04	2.27E-04	0.00314	0.009312	0.006804
1.00E-04	0	0	0	0	0	0	7.20E-05	0	0	4.20E-05	0	0
1.17E-04	0.001513	0	2.90E-05	0.004147	3.70E-05	3.93E-04	7.23E-04	0	4.70E-05	3.60E-05	7.38E-04	0.001214
0	5.12E-04	0	2.96E-04	4.51E-04	4.10E-05	2.80E-05	2.31E-04	8.10E-05	1.10E-05	0	4.99E-04	1.74E-04
0	0	0	7.60E-05	7.70E-05	7.10E-05	0	0	5.20E-05	0	0	0	2.90E-04
0	0	0	0	0	0	0	0	0	0	0	0	0
0	0	0	3.93E-04	0	7.10E-05	0	0	2.14E-04	0	0	0	6.50E-05
0	0	0	0	0	3.90E-05	0	0	0	0	0	0	2.50E-05
0	4.50E-05	0	0	2.50E-05	0	0	0	0	0	0	0	0
0	0	0	0	0	0	0	0	0	0	0	2.04E-04	0
0	0	0	0	0	0	0	0	0	0	0	0	0

H051	H023	H116	H138	H095	H136	H129	H010	H058	H093	H050	H065	H054
0.368989	0.368819	0.367916	0.366506	0.365525	0.365374	0.362936	0.359817	0.358051	0.356857	0.354495	0.353437	0.352816
0.221357	0.168511	0.265468	0.215404	0.227407	0.372097	0.28185	0.257722	0.336644	0.174481	0.29087	0.217701	0.348023
0.230205	0.312841	0.057868	0.258295	0.164832	0.118215	0.132921	0.248821	0.12807	0.32749	0.174717	0.290669	0.104507
0.11432	0.095467	0.226159	0.075343	0.146579	0.062638	0.125325	0.050788	0.077142	0.096557	0.061823	0.075715	0.109093
0.021848	0.025322	0.039236	0.056063	0.026217	0.042161	0.059601	0.05569	0.076515	0.018751	0.080911	0.038016	0.041932
0.023986	0.018383	0.034848	0.020483	0.054741	0.020783	0.025106	0.018144	0.010101	0.020502	0.015782	0.017157	0.01066
0.012141	0.008928	0.007546	0.00736	0.012481	0.017885	0.009636	0.007806	0.007054	0.004619	0.007657	0.004625	0.016397
0.00591	0.001527	9.39E-04	3.18E-04	0.002048	5.90E-04	0.001939	2.01E-04	0.003768	4.18E-04	0.011048	0.002456	0.009697
0	0	2.00E-05	1.41E-04	0	2.57E-04	6.90E-05	0	0	2.58E-04	0	0	0
8.10E-04	2.02E-04	0	6.60E-05	5.60E-05	0	2.73E-04	0	0	3.70E-05	0.002376	1.00E-04	0.004862
2.38E-04	0	0	2.10E-05	2.10E-05	0	1.60E-04	4.91E-04	0.001173	3.00E-05	1.14E-04	1.40E-05	0.001114
2.20E-05	0	0	0	3.00E-05	0	1.52E-04	1.44E-04	4.96E-04	0	1.79E-04	0	3.06E-04
0	0	0	0	0	0	0	0	0	0	0	0	0
1.70E-05	0	0	0	6.30E-05	0	0	2.91E-04	9.86E-04	0	0	1.10E-04	5.93E-04
0	0	0	0	0	0	0	8.50E-05	0	0	0	0	0
0	0	0	0	0	0	0	0	0	0	2.80E-05	0	0
1.57E-04	0	0	0	0	0	3.20E-05	0	0	0	0	0	0
0	0	0	0	0	0	0	0	0	0	0	0	0

H032	H091	H127	H082	H028	H086	H141	H013	H072	H090	H003	H085	H098
0.341562	0.338604	0.338555	0.337168	0.332531	0.331664	0.33089	0.330752	0.328127	0.328087	0.326878	0.323963	0.323498
0.278085	0.33873	0.212268	0.139871	0.270202	0.164073	0.292296	0.15591	0.166143	0.187473	0.271844	0.431463	0.247769
0.048452	0.094945	0.225246	0.246177	0.21748	0.325461	0.158398	0.357491	0.198538	0.294129	0.258397	0.0067	0.198648
0.178288	0.082242	0.140715	0.190783	0.066161	0.102761	0.109892	0.125776	0.248141	0.11032	0.07199	0.070652	0.103535
0.103008	0.083781	0.032401	0.045728	0.024586	0.025702	0.097332	0.016163	0.012977	0.029825	0.064545	0.142495	0.044058
0.031521	0.036472	0.03408	0.028672	0.02614	0.030699	0.006098	0.008554	0.028943	0.031468	0.001652	0.008621	0.062277
0.012654	0.018693	0.015604	0.010721	0.050569	0.013587	0.003831	0.00455	0.014704	0.01842	0.004419	0.011978	0.019176
0.002904	0.006165	9.24E-04	7.66E-04	3.01E-04	0.004177	9.05E-04	6.83E-04	0.002171	1.91E-04	1.17E-04	0.003922	9.72E-04
0	0	0	0	0	0	1.39E-04	3.60E-05	0	3.90E-05	0	0	0
1.02E-04	4.90E-05	1.20E-04	9.00E-05	3.20E-05	0.001664	1.40E-04	8.50E-05	1.40E-04	4.80E-05	0	2.06E-04	0
0.00245	0	0	2.40E-05	0.002784	1.90E-04	7.90E-05	0	1.16E-04	0	4.30E-05	0	0
3.00E-05	2.80E-04	8.70E-05	0	1.57E-04	0	0	0	0	0	3.20E-05	0	6.70E-05
9.18E-04	0	0	0	0.009057	0	0	0	0	0	0	0	0
0	0	0	0	0	0	0	0	0	0	8.30E-05	0	0
2.60E-05	0	0	0	0	0	0	0	0	0	0	0	0
0	3.90E-05	0	0	0	2.20E-05	0	0	0	0	0	0	0
0	0	0	0	0	0	0	0	0	0	0	0	0
0	0	0	0	0	0	0	0	0	0	0	0	0

H074	H046	H059	H055	H150	H034	H025	H124	H033	H015	H109	H014	H047
0.322669	0.320539	0.320443	0.319634	0.319133	0.319109	0.317271	0.314144	0.313848	0.313544	0.313516	0.311657	0.309688
0.073136	0.134099	0.337931	0.385149	0.237685	0.28365	0.215353	0.263015	0.159756	0.194038	0.188923	0.196894	0.227075
0.38164	0.380428	0.205073	0.177919	0.292551	0.169998	0.260087	0.296078	0.401793	0.254596	0.350375	0.190607	0.297897
0.177868	0.116348	0.03191	0.007702	0.058811	0.048784	0.115329	0.061338	0.068225	0.175734	0.088201	0.190616	0.113037
0.010237	0.016286	0.093962	0.104811	0.056538	0.037973	0.017093	0.031751	0.017428	0.02166	0.030508	0.034449	0.017709
0.023028	0.028664	0.006282	0	0.028784	0.08922	0.059665	0.01314	0.017725	0.028327	0.025076	0.045402	0.026435
0.009467	0.002058	0.001238	0.001944	0.006083	0.016572	0.008103	0.012788	0.014743	0.011861	0.002689	0.030296	0.004859
0.001766	0.001236	4.37E-04	0	2.00E-04	9.67E-04	0.006185	0.007088	0.001231	1.94E-04	5.03E-04	3.90E-05	0.001325
0	0	0	0	1.34E-04	0	0	0	0	1.50E-05	7.30E-05	4.00E-05	0
1.89E-04	2.95E-04	0	0	4.80E-05	1.62E-04	2.12E-04	4.66E-04	2.16E-04	0	1.08E-04	0	0.001851
0	4.70E-05	6.84E-04	0.001405	0	0.007197	4.61E-04	1.47E-04	0.004114	0	0	0	4.20E-05
0	0	3.07E-04	3.76E-04	0	1.93E-04	6.00E-05	0	7.65E-04	3.10E-05	2.80E-05	0	3.90E-05
0	0	0	0	0	0.026175	4.30E-05	0	1.56E-04	0	0	0	0
0	0	0.001733	0.00106	0	0	1.38E-04	2.20E-05	0	0	0	0	0
0	0	0	0	0	0	0	2.30E-05	0	0	0	0	0
0	0	0	0	0	0	0	0	0	0	0	0	4.30E-05
0	0	0	0	3.30E-05	0	0	0	0	0	0	0	0
0	0	0	0	0	0	0	0	0	0	0	0	0

H135	H108	H076	H088	H117	H102	H089	H027	H083	H104	H002	H120	H061
0.306606	0.305294	0.303324	0.300383	0.299365	0.299216	0.298045	0.297021	0.295907	0.289925	0.289585	0.287926	0.286512
0.290983	0.219555	0.168471	0.227002	0.250993	0.119205	0.142648	0.165561	0.168041	0.249171	0.209637	0.168849	0.22375
0.193323	0.340572	0.106885	0.322132	0.06976	0.364338	0.178128	0.418729	0.382932	0.326483	0.311842	0.430223	0.276294
0.093917	0.066381	0.340088	0.075947	0.294925	0.160192	0.302676	0.029012	0.103524	0.065052	0.068404	0.036386	0.187374
0.056893	0.025276	0.023017	0.047574	0.057031	0.014051	0.028778	0.034013	0.026637	0.03605	0.074762	0.061181	0.015313
0.042321	0.030352	0.032281	0.012637	0.010574	0.020449	0.035032	0.041539	0.016291	0.027242	0.024979	0.002396	0.005928
0.015724	0.010016	0.025082	0.005433	0.015971	0.020206	0.012892	0.006771	0.00422	0.002964	0.016384	0.005181	0.003471
2.33E-04	0.002273	8.01E-04	0.005792	0.001047	0.002257	0.001444	9.80E-05	0.001808	0.002651	0.003825	0.006355	5.73E-04
0	0	0	1.21E-04	0	5.40E-05	0	0	8.50E-05	0	0	0	2.21E-04
0	1.18E-04	5.10E-05	0.002484	2.95E-04	3.20E-05	2.77E-04	0	4.68E-04	2.61E-04	4.80E-04	0.001382	4.38E-04
0	0	0	4.95E-04	3.90E-05	0	8.00E-05	0.00599	8.70E-05	7.20E-05	2.20E-05	1.21E-04	1.26E-04
0	7.10E-05	0	0	0	0	0	6.24E-04	0	2.70E-05	5.60E-05	0	0
0	0	0	0	0	0	0	5.14E-04	0	0	0	0	0
0	0	0	0	0	0	0	1.28E-04	0	1.02E-04	0	0	0
0	0	0	0	0	0	0	0	0	0	2.40E-05	0	0
0	9.20E-05	0	0	0	0	0	0	0	0	0	0	0
0	0	0	0	0	0	0	0	0	0	0	0	0
0	0	0	0	0	0	0	0	0	0	0	0	0

H107	H005	H049	H052	H020	H057	H097	H142	H067	H137	H119	H087	H115
0.283541	0.279834	0.279564	0.277502	0.277265	0.272819	0.265745	0.263717	0.26311	0.261211	0.257742	0.253435	0.25044
0.262069	0.233691	0.163408	0.103826	0.164621	0.18434	0.12463	0.383229	0.141324	0.128927	0.344488	0.069366	0.295913
0.292866	0.14836	0.336361	0.541767	0.31657	0.320528	0.429916	0.122032	0.38252	0.422182	0.267897	0.540408	0.169943
0.055826	0.232783	0.117809	0.01804	0.089879	0.099064	0.102718	0.13065	0.154953	0.148625	0.077071	0.066348	0.217802
0.086252	0.065573	0.030207	0.016133	0.089592	0.055702	0.033783	0.077275	0.03704	0.016083	0.010283	0.021856	0.048823
0.01002	0.015611	0.032009	0.032184	0.04854	0.033893	0.035157	0.018017	0.013478	0.014931	0.028181	0.041019	0.012847
0.008498	0.02308	0.017742	0.009172	0.011239	0.033236	0.007182	0.004605	0.005937	0.006231	0.009512	0.006765	0.004057
5.58E-04	9.02E-04	0.014924	0.00129	0.00199	3.91E-04	5.32E-04	2.77E-04	0.001077	0.001468	0.003584	5.42E-04	4.30E-05
0	3.20E-05	0	0	0	0	2.13E-04	1.34E-04	4.20E-05	0	0	0	1.32E-04
5.90E-05	1.06E-04	0.006672	0	8.30E-05	0	1.24E-04	4.10E-05	3.71E-04	3.09E-04	8.82E-04	1.75E-04	0
6.20E-05	0	6.94E-04	4.30E-05	1.26E-04	0	0	0	1.48E-04	3.30E-05	6.90E-05	6.30E-05	0
1.06E-04	2.80E-05	5.63E-04	1.40E-05	5.80E-05	0	0	2.30E-05	0	0	3.10E-05	2.30E-05	0
0	0	0	0	0	0	0	0	0	0	0	0	0
1.43E-04	0	0	2.90E-05	1.50E-05	2.70E-05	0	0	0	0	2.19E-04	0	0
0	0	0	0	0	0	0	0	0	0	0	0	0
0	0	4.70E-05	0	0	0	0	0	0	0	4.10E-05	0	0
0	0	0	0	2.20E-05	0	0	0	0	0	0	0	0
0	0	0	0	0	0	0	0	0	0	0	0	0

H133	H077	H140	H110	H123	H147	H131	H069	H064	H066	H121	H031	H024
0.249437	0.245328	0.24304	0.240821	0.240725	0.238073	0.237632	0.235318	0.235088	0.232286	0.227624	0.223834	0.223581
0.284923	0.08319	0.303439	0.259407	0.110677	0.12467	0.252042	0.0944	0.162027	0.392922	0.10133	0.450832	0.2141
0.298025	0.535264	0.315797	0.266286	0.476858	0.428378	0.156781	0.485205	0.429469	0.223855	0.529852	0.180078	0.505341
0.064784	0.10091	0.043247	0.14075	0.083638	0.150179	0.235287	0.111949	0.143336	0.06236	0.090032	0.071626	0.022627
0.06731	0.01913	0.053981	0.06841	0.045827	0.030035	0.065354	0.037748	0.019255	0.065451	0.015321	0.058952	0.012286
0.032442	0.009936	0.013178	0.015032	0.030104	0.018967	0.036909	0.023365	0.001635	0.004466	0.028855	0.005936	0.012209
0.002134	0.005514	0.008449	0.007848	0.009862	0.007493	0.010023	0.011024	0.00743	0.006844	0.004005	0.002703	0.004942
5.35E-04	5.06E-04	0.017417	0.001072	0.001926	0.001813	0.005414	7.27E-04	0.001463	0.009553	0.002352	0.004996	3.70E-05
0	1.20E-04	0	0	0	1.72E-04	0	3.70E-05	6.80E-05	0	2.00E-05	0	0
2.78E-04	5.30E-05	0.001399	2.43E-04	2.98E-04	2.20E-04	4.64E-04	1.80E-04	1.22E-04	9.90E-04	4.54E-04	4.30E-05	0
6.70E-05	4.90E-05	0	4.90E-05	0	0	1.80E-05	4.70E-05	0	4.91E-04	1.33E-04	7.03E-04	0.004546
0	0	0	5.60E-05	2.30E-05	0	3.60E-05	0	7.90E-05	7.40E-05	0	1.33E-04	4.40E-05
0	0	0	0	0	0	0	0	0	0	0	1.41E-04	2.87E-04
6.50E-05	0	0	2.60E-05	4.60E-05	0	1.60E-05	0	0	1.39E-04	2.20E-05	2.30E-05	0
0	0	0	0	0	0	0	0	0	4.26E-04	0	0	0
0	0	5.30E-05	0	0	0	2.40E-05	0	2.80E-05	1.43E-04	0	0	0
0	0	0	0	1.60E-05	0	0	0	0	0	0	0	0
0	0	0	0	0	0	0	0	0	0	0	0	0

H004	H048	H062	H006	H045	H044	H068	H075	H096	H122	H132	H139	H084
0.220714	0.215718	0.214338	0.213087	0.20526	0.201654	0.201127	0.192271	0.192233	0.186859	0.182311	0.178282	0.166955
0.049219	0.108017	0.252984	0.097792	0.510898	0.224138	0.101277	0.148174	0.208528	0.244289	0.224196	0.47481	0.206818
0.582023	0.518465	0.33082	0.565796	0.076185	0.255098	0.597827	0.565776	0.418806	0.451079	0.46504	0.188042	0.518987
0.078461	0.045089	0.089353	0.05382	0.098696	0.186554	0.074136	0.04212	0.091442	0.073924	0.046466	0.025917	0.073309
0.031781	0.099075	0.071458	0.033445	0.082598	0.115889	0.008606	0.021019	0.037449	0.023416	0.054126	0.10497	0.017179
0.033072	0.006888	0.029939	0.01875	0.002028	0.005822	0.010036	0.021428	0.040622	0.009643	0.01479	0.018561	0.006217
0.003876	0.002461	0.005784	0.008856	0.015319	0.00976	0.004888	0.003033	0.00553	0.008082	0.008972	0.004516	0.004016
7.44E-04	0.002267	0.00428	0.00714	0.005946	0.001003	0.001605	0.005918	0.004763	0.00262	0.003506	0.003351	0.00445
0	6.48E-04	0	0	0	0	0	0	0	0	0	1.06E-04	0
8.10E-05	0.001099	7.68E-04	0.001041	0.001485	8.20E-05	4.47E-04	2.10E-04	4.18E-04	3.50E-05	3.64E-04	0.001304	0.001126
0	2.73E-04	9.30E-05	6.20E-05	3.78E-04	0	0	1.90E-05	4.80E-05	3.10E-05	1.52E-04	1.03E-04	9.43E-04
0	0	7.80E-05	1.21E-04	0.001207	0	0	3.20E-05	3.20E-05	0	7.70E-05	3.80E-05	0
0	0	0	0	0	0	0	0	0	0	0	0	0
0	0	1.05E-04	5.70E-05	0	0	2.30E-05	0	0	2.20E-05	0	0	0
0	0	0	0	0	0	0	0	0	0	0	0	0
0	0	0	3.30E-05	0	0	2.80E-05	0	1.29E-04	0	0	0	0
2.90E-05	0	0	0	0	0	0	0	0	0	0	0	0
0	0	0	0	0	0	0	0	0	0	0	0	0

H070	H017	H063	H101	H149
0.160249	0.160059	0.148999	0.133677	0.115233
0.229192	0.096519	0.131764	0.235405	0.227664
0.426412	0.637032	0.570739	0.50221	0.537353
0.131003	0.06548	0.054591	0.034575	0.071831
0.038133	0.02379	0.06789	0.078447	0.029715
0.005259	0.01067	0.017611	0.004079	0.005187
0.008794	0.001947	0.006046	0.006282	0.004916
7.38E-04	0.003968	0.001855	0.004248	0.005052
2.20E-04	2.80E-05	0	0	2.41E-04
0	3.84E-04	2.50E-04	9.83E-04	0.001795
0	1.23E-04	0	7.40E-05	9.09E-04
0	0	2.55E-04	2.00E-05	3.40E-05
0	0	0	0	0
0	0	0	0	0
0	0	0	0	0
0	0	0	0	7.00E-05
0	0	0	0	0
0	0	0	0	0

Table S3 .The abundance and composition at the genus level of each sample											
ID	CC19	CC35	CC50	CC74	CC05	CC53	CC24	CC49	CC22	CC06	CC47
Prevotella	0.921263	0.871393	0.86155	0.642051	0.598125	0.516236	0.423304	0.399472	0.389925	0.334897	0.332573
Neisseria	0.00937	0.00157	7.73E-04	4.58E-04	1.09E-04	3.32E-04	0.02583	8.41E-04	0.025933	9.70E-05	0.005651
Streptococcus	9.89E-04	0.006074	0.006001	0.025651	0.021348	0.034356	0.035086	0.029724	0.010576	0.087806	0.004299
Veillonella	0.004498	0.00815	0.0095	0.091315	0.173098	0.122294	0.146269	0.339403	0.271248	0.288827	0.056016
Leptotrichia	0.050485	0.001127	5.66E-04	0.002493	0.026175	0.139887	0.110416	0.023469	0.02661	0.077076	0.557836
Fusobacterium	9.03E-04	4.27E-04	2.16E-04	7.90E-05	1.42E-04	7.55E-04	0.050021	0.008273	7.13E-04	1.10E-05	0.008184
Alloprevotella	8.30E-05	0.001308	0.001771	0.032967	0.00578	8.50E-05	0.004616	3.74E-04	4.92E-04	0.002528	8.64E-04
Porphyromonas	3.34E-04	8.70E-05	2.89E-04	0.009535	0.003195	5.88E-04	0.002505	3.41E-04	0.008506	3.80E-05	1.03E-04
Haemophilus	8.40E-05	6.25E-04	5.20E-04	2.10E-05	8.01E-04	2.01E-04	0.001851	6.29E-04	0.001548	1.30E-05	7.80E-05
Actinomyces	8.80E-05	0.091603	0.100218	0.057733	0.001119	0.099958	0.068269	0.113002	0.089208	0.003255	0.021455
Rothia	4.98E-04	0.001309	0.001301	0.01227	0.002147	0.005199	0.006227	0.019325	0.055306	0.020737	2.75E-04
Capnocytophaga	8.50E-05	3.35E-04	4.52E-04	0.005787	0.009068	5.60E-04	0.002522	0.014468	0.00105	7.45E-04	9.00E-05
Gemella	2.53E-04	1.67E-04	0	1.20E-05	3.75E-04	0.001556	0.001234	0.00141	7.09E-04	0.002778	0
Lachnoanaerobaculum	7.10E-05	7.70E-05	0	0.009595	0.028652	0.009961	0.011414	2.77E-04	0.017897	0.01567	0.001313
TM7x	0	3.30E-04	7.09E-04	0.005126	0.072592	0	0.005819	7.57E-04	0.00464	0.043868	4.70E-04
Granulicatella	4.18E-04	0	0	0.008416	2.53E-04	0.001025	0.009907	0	6.02E-04	0.027095	2.08E-04
Campylobacter	0.0011	5.32E-04	2.73E-04	0.010452	0.003707	0.002067	0.019561	4.57E-04	0	5.51E-04	4.96E-04
Halomonas	8.80E-05	0	0	1.50E-05	5.90E-05	0.001361	1.69E-04	1.17E-04	0	0	0
Moraxella	0	0	0	0	0	0	0	0	0	0	0
Actinobacillus	0	0	0	0	0	0	2.10E-05	0	0	0	0
Lautropia	3.48E-04	9.00E-05	6.60E-05	0	0	4.40E-05	7.00E-05	0	1.09E-04	0	0
g__Absconditabacteriales_	0	0	0	0	0.006571	0	8.04E-04	6.10E-05	0	1.20E-05	0
Oribacterium	0	9.20E-05	0	5.10E-05	2.02E-04	0.008882	0.00506	1.14E-04	0.007604	0.031241	4.66E-04
Peptostreptococcus	0.007871	0	7.60E-05	0.002736	0.003211	0.00103	0.005507	0.007498	0.003751	0.002046	1.85E-04
Selenomonas	8.60E-05	6.54E-04	3.47E-04	0.001582	0	0.020793	0.003013	0.002086	0.00182	3.41E-04	8.28E-04
Bacteroides	0	9.10E-05	0	0	0	0	1.90E-05	0	0	1.30E-05	0
Solobacterium	8.10E-05	7.90E-05	0	9.11E-04	0.001159	0.002972	0.025917	0.002291	0.015866	0.001445	1.74E-04
Stomatobaculum	8.30E-05	0	1.27E-04	0.037722	3.39E-04	0.00591	0.001363	0	0.041209	0.036697	0.001415
Megasphaera	0	0.003375	0.004321	0.01012	0.006836	0.007511	0.008908	0.009718	0	0.00692	0.001877
Clostridia_UCG-014	0	0	0	3.60E-05	0	0.001993	0.003735	0	0	4.33E-04	0
Pseudomonas	0	0	0	0	0	0	0	0	0	2.10E-05	0
Lachnospiraceae_uncultured	0	0	7.70E-05	0	0	0	0	0	0.014635	0	1.83E-04
[Eubacterium]_nodatum_group	8.60E-05	0.001028	0.001736	0.002833	0.013303	0.001892	0.003135	0.010214	8.24E-04	8.30E-05	0.001354
Atopobium	7.80E-05	0.005199	0.006262	0.002779	1.18E-04	0.003609	0.003689	0.003616	5.03E-04	9.18E-04	9.50E-05
Aggregatibacter	0	0	0	0	0	5.70E-05	1.80E-04	0	0	0	0
Muribaculaceae	0	0	0	0	0	0	0	0	0	0	0
Tannerella	8.80E-05	0	0	0	0.009306	0.001891	4.72E-04	0.004078	0	0	0
Corynebacterium	3.37E-04	0	0	0.019406	0	0	5.63E-04	0	0	0	0
Treponema	0	0	0	0	0	3.77E-04	6.10E-04	0	0	3.60E-05	0
Faecalibacterium	0	0	0	0	0	0	0	0	0	0	0
Lachnospiraceae_unclassified	0	0	7.50E-05	2.23E-04	3.40E-05	7.00E-05	2.85E-04	0	1.37E-04	4.00E-05	2.77E-04
Neisseriaceae_unclassified	0	0	0	7.81E-04	0	9.80E-05	1.52E-04	0	1.14E-04	1.36E-04	6.48E-04
Saccharimonadales	0	0	0	0	0	2.30E-05	1.04E-04	0	0	9.50E-05	0
Lactobacillales_unclassified	0	0.001044	9.52E-04	6.35E-04	1.15E-04	0.001108	2.83E-04	0.001636	0.001309	5.31E-04	3.00E-04
Parvimonas	0	0	0	0	0	0	9.00E-06	0	0	0	0
Bergeyella	0	0	0	0	4.10E-05	6.70E-05	3.35E-04	0	0	1.50E-05	0
Rhodococcus	0	0	0	8.00E-06	0	0	0	0	0	1.80E-05	0
Johnsonella	0	0	0	0	0	0	6.15E-04	0	1.30E-04	3.40E-05	1.88E-04
F0332	0	0	0	0	0	1.42E-04	1.48E-04	0	0	2.40E-05	0
Abiotrophia	0	0	0	1.59E-04	0	0	0	0	0	0	0
Filifactor	0	0	0	0	0	0	4.30E-05	0	0	0	0
Dialister	0	0	0	0.004237	0	6.20E-05	2.64E-04	0	0	4.89E-04	9.30E-05
Lachnospiraceae_NK4A136_group	0	0	0	0	0	0	0	0	0	0	0
Fusicatenibacter	0	0	0	0	0	0	0	0	0	0	0
Saccharimonadaceae	0	0	0	0	0	2.40E-05	0.001067	0	0	0	8.60E-05
Acinetobacter	0	0	0	0	0	0	0	0	0	0	0

F0058	0	0	0	0	0.006291	0	2.10E-05	0	0	2.30E-05	0
Sphingomonas	0	0	0	0	0	0	0	0	0	0	0
Bifidobacterium	0	0	0	0	0	2.80E-05	0	5.30E-05	0	0	0
Kingella	0	8.70E-05	0	0	0	0	1.78E-04	0	0	0	0
Lactobacillus	3.32E-04	0	0	1.50E-05	0	0	0	0	0	2.40E-05	0
Roseburia	0	0	0	0	0	0	0	0	0	0	0
Leptotrichiaceae_uncultured	0	0	0	0	0	0	0	0	0	0	0
Amnipila	0	0	0	0	0	0	0.00119	0	0	0	0
Bacteria_unclassified	0	0.001027	3.54E-04	2.21E-04	3.90E-05	2.21E-04	2.36E-04	7.23E-04	0.002686	2.23E-04	1.00E-04
Actinobacteria_unclassified	0	0.001537	0.00112	3.36E-04	0	0.001047	5.01E-04	0.003777	0.002167	2.10E-05	8.08E-04
Megamonas	0	0	0	0	0	0	0	0	0	0	0
Scardovia	0	8.20E-05	0	0	0	4.04E-04	2.80E-05	0	0	3.21E-04	0
Butyrivibrio	0	0	0	0	0	4.64E-04	0.001162	0	1.32E-04	2.87E-04	0
Pelagibacterium	0	0	0	0	0	9.00E-05	4.30E-05	0	0	0	0
[Eubacterium]_ruminantium_group	0	0	0	0	0	0	0	0	0	0	0
Burkholderiales_unclassified	0	0	0	0	0	0	4.60E-05	5.80E-05	1.18E-04	0	0
Shuttleworthia	0	0	8.50E-05	0	0	8.40E-04	0	0	0	0.002874	0
Selenomonadaceae_uncultured	0	0	0	0	0	0	0	0	0	0	0
Christensenellaceae_R-7_group	0	0	0	0	0	0	0	0	0	0	0
Gracilibacteria	0	0	0	0	0	0	0	0	0	0	0
Helicobacter	0	0	0	0	0	0	0	0	0	0	0
Prevotellaceae_NK3B31_group	0	0	0	0	0	0	0	0	0	0	0
Acholeplasma	0	0	0	0	0	0	0	0	0	0	0
Xanthomonadaceae_unclassified	0	0	0	0	0	1.03E-04	3.00E-05	0	0	0	0
Phascolarctobacterium	0	0	0	0	0	0	0	0	0	0	0
Rhizobiaceae_unclassified	0	0	0	1.10E-05	0	1.67E-04	3.70E-05	0	0	0	0
Serratia	0	0	0	0	0	0	0	0	0	0	0
Chloroplast	0	0	0	0	0	0	0	0	0	2.01E-04	0
Mucispirillum	0	0	0	0	0	0	0	0	0	0	0
Propionibacterium	0	0	0	0	0	2.00E-05	0	0	0	0	0
Others	0	5.01E-04	2.63E-04	0.001222	0.00569	0.00164	0.005137	0.001738	0.001923	0.008446	0.001012

CC23	CC33	CC56	CC66	CC02	CC01	CC72	CC29	CC17	CC41	CC44	CC40	CC08
0.324891	0.314679	0.314387	0.303656	0.272016	0.261168	0.252413	0.251753	0.240955	0.232602	0.216897	0.204899	0.19955
0.5029	0.00462	7.73E-04	0.035194	5.30E-04	0.009157	0.077688	0.020225	0.001427	0.408071	0.091353	0.329016	0.001715
0.015429	0.103492	0.020378	0.26907	0.031288	0.003805	0.047749	0.162845	0.018697	0.061987	0.188636	0.011771	5.06E-04
0.042081	0.03254	0.22669	0.186417	0.180337	0.195675	0.0289	0.05186	0.154233	0.034127	0.075722	0.162678	0.211957
0.014453	0.472303	0.215677	0.03695	0.054877	0.491052	0.137554	0.098235	0.23083	0.170592	0.185432	0.049331	0.32818
0.057151	0.001006	1.33E-04	0.016056	0.030034	8.13E-04	0.128684	0.004748	0.00559	0.001345	0.02043	3.11E-04	2.06E-04
2.78E-04	8.31E-04	2.70E-04	0.004484	0.014985	1.34E-04	0.009701	0.00499	7.28E-04	0.003487	0.00145	0.002045	0
9.37E-04	7.96E-04	1.66E-04	0.003763	7.80E-05	6.88E-04	0.012226	0.00145	7.18E-04	0.005384	0.016125	0.022011	5.70E-05
3.45E-04	0.001504	5.84E-04	0.001808	7.70E-05	8.37E-04	0.131644	8.59E-04	0.002978	0.001651	0.022869	0.015967	2.80E-05
0.016204	0.018064	0.183491	0.05462	0.018513	0.012591	0.013662	0.238293	0.035807	0.016784	0.081073	0.030903	0.238252
0.00555	0.008526	0.011995	0.00663	0.18693	0.002254	0.019779	0.019699	5.59E-04	0.010712	0.008603	0.013251	4.80E-04
3.67E-04	1.26E-04	0	7.12E-04	0.001116	1.34E-04	0.013257	9.81E-04	3.61E-04	1.25E-04	0.004454	0.013337	0
0	0.006591	1.56E-04	0.003439	2.30E-05	0	0.013216	0.03289	0.002922	0.003686	0.019422	0.001216	9.70E-05
0.003228	0.004316	0.002888	0.003217	0.07151	0.002359	0.010126	0.014532	0.011581	0.003377	0.006673	0.003895	0.008754
6.50E-05	7.43E-04	0.001725	0.012155	0	1.41E-04	0.014732	8.52E-04	0.003544	7.08E-04	0.008049	0.002061	4.47E-04
0.002348	5.12E-04	3.01E-04	7.65E-04	0.050813	7.80E-04	0.006422	0.004399	6.82E-04	0.001886	0.003443	6.29E-04	5.81E-04
3.59E-04	0.002941	4.80E-05	0.002173	0.001161	0.001503	0.01504	0.001288	0.014116	0.001475	0.001159	0.027255	1.29E-04
0	2.03E-04	4.00E-05	4.30E-05	0.004238	1.30E-04	0.004866	0.00228	0.009998	0	0.001802	0.029917	2.70E-05
0	0	0	0	0	0	0	0	0	0	0	0	0
0	0	0	7.90E-05	0	0	0.00827	1.90E-05	0	0	0	0	0
0	1.76E-04	1.78E-04	3.01E-04	3.75E-04	7.89E-04	0.001216	8.90E-05	0.003847	0.001761	0.001108	1.21E-04	0
5.30E-05	1.22E-04	0	0.001343	0	0	1.17E-04	0	0	0	0	0	0
8.95E-04	3.50E-04	2.81E-04	0.00894	0.032313	0.001487	0.003015	0.006934	0.012232	0.002899	0.001498	0.001432	3.02E-04
0.0015	0	1.16E-04	0.001298	0.007411	1.40E-04	0.00499	0.002156	0.006757	8.51E-04	0.002202	1.36E-04	3.60E-05
2.54E-04	0.010227	0.005463	0.004362	0.00283	0.004421	8.02E-04	0.025929	0.02542	0.003875	0.00189	0.011321	2.04E-04
0	0	0	0	1.02E-04	0	0	6.70E-05	0.034363	0	0	2.10E-05	0
0.004914	1.62E-04	4.07E-04	0.003318	0.003302	0	0.005223	0.003359	0.005327	7.22E-04	4.45E-04	0.00354	8.31E-04
5.40E-05	7.10E-04	1.97E-04	0.001212	1.02E-04	0.001467	2.63E-04	7.13E-04	0.004934	0.00118	0.00107	1.50E-05	0.002851
1.84E-04	4.93E-04	0.007137	0.004482	0.008869	0.003483	8.97E-04	2.63E-04	0.021854	0.001355	0	0.00468	0.002644
2.41E-04	0.002432	2.39E-04	0.003882	8.30E-05	0	3.08E-04	0.008376	0.012774	0	2.76E-04	6.96E-04	2.50E-05
0	0	0	0	0	0	0	0	0	0	0	6.10E-05	2.20E-05
0	0.001827	0	2.30E-05	3.00E-05	0	6.52E-04	0.001903	0.003895	0.008529	0	2.50E-05	0
7.69E-04	4.33E-04	3.43E-04	0.001942	0.005547	9.28E-04	0.003276	0.010602	0.005232	3.56E-04	0.00227	1.90E-05	9.10E-05
3.12E-04	4.27E-04	5.90E-04	0.002378	0.010417	6.73E-04	0.003052	0.001764	0.011516	3.66E-04	4.87E-04	0.003869	0.001196
0	5.50E-05	0	7.30E-05	0	0	4.10E-05	1.60E-05	0	9.92E-04	0	0	0
0	0	0	0	2.30E-05	0	0	0	2.50E-04	0	0	0	0
0	2.48E-04	2.04E-04	4.56E-04	0.001125	7.02E-04	4.51E-04	0.001366	1.80E-05	0	0.001885	0	0
0	1.03E-04	0	2.64E-04	0	0	3.14E-04	3.30E-05	2.60E-05	1.03E-04	1.07E-04	4.90E-05	0
0	5.20E-05	0	9.10E-05	0	0	0.001853	1.86E-04	0.002643	0	4.50E-05	0	0
0	0	0	0	1.17E-04	0	0	7.80E-05	0.014375	0	1.56E-04	6.10E-05	0
1.03E-04	0	4.10E-05	0	1.93E-04	1.29E-04	1.25E-04	6.40E-05	0.003308	0	2.94E-04	0	0
5.96E-04	2.92E-04	4.70E-05	9.60E-05	0	0	1.51E-04	1.62E-04	1.50E-04	0.006417	0.002015	0.014013	0
0	5.10E-05	0	2.75E-04	0	0	0.003355	0	3.74E-04	0	0	0	0
3.60E-04	8.26E-04	8.77E-04	0.004354	4.30E-05	1.28E-04	0.002728	0.006398	0	0.001133	0.005366	0	2.25E-04
0	0	0	2.50E-05	8.40E-05	0	9.40E-05	4.40E-05	2.10E-05	1.06E-04	1.61E-04	0	0
0	5.70E-05	0	1.39E-04	5.15E-04	1.33E-04	0.001254	8.20E-05	3.00E-05	0	6.70E-05	0.001632	0
0	0	0	0	2.40E-05	0	0	4.60E-05	1.13E-04	0	0	3.60E-05	3.00E-05
0	0	4.30E-05	0	0	0	3.11E-04	0	7.40E-05	1.21E-04	2.12E-04	1.30E-05	0
0	0	0	0	0	0	3.10E-05	0.002664	4.90E-05	0	6.40E-05	3.30E-05	0
0	5.50E-05	0	8.70E-05	5.88E-04	0	0.006444	2.41E-04	0	1.31E-04	0	0.019792	0
0	0	0	4.63E-04	0	0	5.78E-04	0	0	1.23E-04	0	0	0
0	1.87E-04	5.42E-04	0.005422	2.50E-05	1.34E-04	6.20E-05	6.40E-05	0.004364	1.25E-04	0	0	0
0	0	0	0	0	0	0	0	0.00177	0	5.20E-05	3.20E-05	0
0	0	0	0	0	0	0	0	0.002052	0	0	0	0
0	1.23E-04	0	2.27E-04	0	0	2.51E-04	8.60E-05	3.54E-04	1.34E-04	3.83E-04	0	0
0	0	0	0	0	0	0	0	2.00E-05	0	0	7.90E-05	0

0	0	0	6.30E-05	3.80E-05	0	1.00E-04	2.20E-05	2.20E-05	0	6.30E-05	0	0
0	0	0	0	0	0	0	0	2.70E-05	0	0	0	0
0	0	2.58E-04	4.08E-04	0	0	0	6.30E-05	0.008373	0	1.01E-04	0	0
0	0	0	8.10E-05	0	0	5.70E-05	0	2.90E-05	0	1.09E-04	0.008858	0
0	0	3.40E-05	0	2.10E-05	0	0	4.20E-05	0.001086	1.16E-04	0	1.30E-05	5.70E-05
0	0	0	0	1.05E-04	0	0	3.30E-05	0.005423	0	5.60E-05	0	0
0	0	0	0	0	0	0	0	0	0	0	0	0
7.30E-05	0	0	0	0	0	0	1.70E-05	0	0	0	1.80E-05	0
4.29E-04	9.83E-04	3.88E-04	9.63E-04	2.30E-04	2.77E-04	2.85E-04	8.45E-04	0	3.66E-04	0.001175	0	0
5.73E-04	6.78E-04	0.002082	0.00406	2.20E-05	2.71E-04	8.60E-05	0.005371	0	0.001849	0.008446	0	2.80E-04
0	0	0	0	4.50E-05	0	0	1.40E-05	0.001696	0	0	7.00E-06	0
0	0	0	0	0	0	0	1.23E-04	1.80E-05	0	9.70E-05	0	0
1.29E-04	0.001035	0	3.40E-04	0.003521	1.30E-04	5.90E-05	3.06E-04	0.008132	1.35E-04	1.73E-04	3.50E-05	0
0	0	0	3.00E-05	4.95E-04	0	4.02E-04	2.17E-04	4.57E-04	0	5.50E-05	0.001527	0
0	0	0	0	0	0	0	0	2.46E-04	0	0	0	0
4.42E-04	6.38E-04	4.10E-05	0.001893	0	0	2.40E-05	3.10E-04	0	0.002996	0.004528	0	2.40E-05
0	1.67E-04	0	3.08E-04	0	0	0	1.60E-05	0.001487	0.001712	2.36E-04	2.10E-05	2.60E-05
0	0	0	2.16E-04	0	0	0	0	0	0	0	0	0
0	0	0	0	0	0	0	0	8.11E-04	0	0	0	0
0	0	0	1.02E-04	0	0	0	0	0	0	0	0	0
0	0	0	0	0	0	0	0	2.00E-05	0	0	0	0
0	0	0	0	0	0	0	0	2.90E-05	0	0	0	0
0	0	0	0	0	0	0	0	0	0	0	0	0
0	0	0	8.40E-05	1.25E-04	1.39E-04	4.17E-04	2.69E-04	3.46E-04	0	0	0.001533	0
0	0	0	0	2.90E-05	0	0	0	0.001017	0	0	0	0
0	0	0	0	2.23E-04	0	2.48E-04	3.41E-04	4.59E-04	0	1.78E-04	0.001103	0
0	0	0	0	2.30E-05	0	3.50E-05	3.70E-05	4.50E-05	0	0	1.17E-04	0
0	0	0	0	2.50E-05	0	0	0	7.84E-04	0	5.20E-05	2.00E-05	0
0	0	0	0	0	0	0	0	0	0	0	0	0
0	0	0	0	0	0	0	0	0	0	0	0	0
0.001533	0.003298	7.90E-04	0.004768	0.002454	0.001348	0.010504	0.006091	0.055625	0.003548	0.009086	0.004579	1.90E-04

CC61	CC54	CC34	CC48	CC31	CC09	CC70	CC18	CC04	CC67	CC15	CC10	CC69
0.192295	0.177073	0.159755	0.149235	0.13598	0.127817	0.113815	0.105027	0.099062	0.09836	0.096842	0.096125	0.09154
0.112968	0.001545	0.070666	7.75E-04	0.002496	0.00664	0.48627	1.61E-04	3.53E-04	0.03792	0.002132	0.067052	0.569753
0.224938	0.027236	0.133463	0.005366	0.036673	0.023566	0.027996	0.305548	0.252002	0.630452	0.034168	0.083109	0.032161
0.174371	0.166704	0.021506	0.309109	0.005949	0.073862	0.111282	0.01019	0.242131	0.032028	0.428454	0.172684	0.039724
0.010534	0.173238	0.051547	3.09E-04	0.688922	0.399263	0.031557	0.001817	0.078583	0.036119	0.035393	0.019084	0.004027
0.001114	0.002029	0.030139	1.17E-04	0.07169	0.091465	0.036432	0.015099	0.155484	0.002922	8.60E-05	0.002229	0.107078
0.001198	0.002294	0.010999	3.40E-05	8.71E-04	8.07E-04	0.001078	7.48E-04	2.18E-04	0.001939	5.60E-05	0.02624	0.001543
2.45E-04	7.44E-04	0.090217	0.306825	0.001752	0.003434	0.020049	0.023017	0.001217	0.002834	1.00E-04	0.03321	0.05184
0.010062	1.94E-04	0.004298	2.00E-04	2.24E-04	0.001696	0.002939	0.001061	0.01016	0.004892	4.40E-05	0.005112	0.004311
0.007739	0.261941	0.032339	0.020016	0.008479	0.013374	0.00356	0.027543	0.009839	0.04048	0.143786	0.054712	0.004299
0.025313	0.009582	0.014729	0.012439	0.007938	1.40E-04	0.01949	0.149985	0.01359	0.023864	0.021772	0.081398	0.038063
0.005844	0.002141	0.070416	1.16E-04	6.00E-05	0.029619	0.002532	0.065003	0.001592	8.28E-04	0.004906	0.055502	0.003036
0.107186	0.004478	0.02984	2.80E-05	0.001929	0.005798	0	0.113569	0.01973	0.015244	0.006255	0	8.05E-04
0.001823	0.026952	0.010239	1.14E-04	0.002877	0.128736	0.004924	0.005358	0.009039	0.00179	0	0.001593	0.012988
0	0.007415	0.059284	0	6.67E-04	0.014515	0	0.001231	9.90E-04	0.001834	0	7.10E-05	0.001428
0.032267	8.51E-04	0.014826	8.70E-05	7.90E-04	0.005296	0.023013	0.020839	0.070428	0.003559	0.13722	0.02631	0.001353
0.017476	0.009002	6.63E-04	0.08024	9.27E-04	0.001854	0.048493	0.003044	0.001384	5.71E-04	0.007317	0.007767	1.88E-04
0.015764	8.80E-05	1.70E-05	0.02989	2.24E-04	0.003015	0.003978	0.006785	0	0	0	0.18784	6.50E-05
0	0	0	0	0	0	0	0	0	0	0	0	0
0	0	9.10E-05	2.30E-05	0	3.20E-05	0	0	0	3.28E-04	0	0	5.90E-05
3.80E-05	1.15E-04	5.28E-04	0	0	1.08E-04	0.007378	1.90E-05	6.00E-05	0.001171	0.004731	7.10E-05	0.00335
2.10E-05	0	0.007509	2.50E-05	5.90E-05	4.00E-05	0	2.00E-06	0	2.04E-04	0	2.71E-04	6.37E-04
0.001116	0.01376	0.023828	2.20E-05	0.001379	0.007702	6.90E-05	0.006587	4.74E-04	5.96E-04	9.70E-05	0	0.002655
0	6.72E-04	0.035642	7.80E-05	0.001358	0.006822	0.002935	0.005458	0.004825	1.52E-04	0	0.004149	0.00445
0.005503	0.011716	0.002456	0.001906	0.005105	5.68E-04	0.005649	0.02852	2.50E-05	0.005824	0	2.78E-04	2.44E-04
2.40E-05	0	4.40E-05	1.19E-04	0	0	0	2.90E-05	0	0	9.60E-05	0.001424	0
7.57E-04	0.001964	0.005148	0	6.40E-04	0.003124	0.003653	6.72E-04	0.002002	6.10E-04	0.002187	3.89E-04	9.47E-04
6.65E-04	0.011622	0.014803	2.00E-05	8.65E-04	0.024529	7.21E-04	0.011723	0.007423	2.63E-04	3.90E-05	0	0.00291
0.013434	0.008948	7.93E-04	0.001974	3.01E-04	0	0.002374	4.30E-05	0.001178	5.40E-05	0	0.001081	1.02E-04
3.30E-05	0.045238	0.01053	6.20E-05	0.002243	0	0	2.77E-04	0.003023	0.002927	0	0	9.50E-04
1.12E-04	0	0	4.26E-04	0	0	7.00E-05	7.60E-05	2.80E-05	0	5.50E-05	5.51E-04	0
0	0.017326	0.011508	0	6.24E-04	0.001469	0	0	0	0.001861	0	3.20E-05	0
0.003939	5.81E-04	0.008211	2.80E-05	0.001698	0.006611	4.80E-05	3.40E-05	0.002639	3.04E-04	0	6.89E-04	0.001958
0.023502	0.002172	0.005461	0	1.85E-04	0.001994	0.001227	0.011128	8.06E-04	0.00131	0.040454	2.14E-04	3.24E-04
4.00E-05	0	1.18E-04	0	0	0	0	0	0	2.44E-04	0	0	5.90E-05
0	0	0	0	0	0	0	0	0	0	0	0	0
2.00E-05	4.40E-05	0.019298	7.50E-05	0.008905	1.80E-05	2.40E-05	0.001858	0.001652	5.14E-04	0	0.001958	0.002346
1.50E-05	5.50E-05	2.60E-05	0	0	0	0.024022	0.001336	2.50E-05	7.29E-04	0	0	1.19E-04
2.48E-04	5.30E-05	8.70E-04	0	1.49E-04	5.71E-04	2.90E-05	0.007504	4.00E-05	2.80E-05	0	0	0
3.80E-05	0	0	4.70E-05	0	0	0	9.00E-06	5.40E-05	0	4.60E-05	6.34E-04	0
0	2.00E-04	1.59E-04	0	3.46E-04	4.71E-04	0	1.27E-04	0	0	0	1.26E-04	3.49E-04
0.002729	5.50E-05	7.26E-04	0.008543	3.27E-04	2.90E-05	0.003803	0.001227	0	3.27E-04	0	0	2.72E-04
0	0	0.004093	2.10E-05	0	0	0	0.003994	0	7.04E-04	0	3.40E-05	0
1.90E-05	0.001483	0.001217	0	9.70E-04	6.80E-05	0	5.51E-04	3.60E-05	0.012865	0.001097	0	0.001247
7.10E-05	4.10E-05	0.003745	0	0	0	0	3.30E-05	6.45E-04	0	0	5.00E-05	0.001114
2.71E-04	0	6.17E-04	0	0	3.88E-04	3.58E-04	6.20E-05	7.73E-04	3.40E-05	0	0.004069	2.06E-04
7.90E-05	0	0	1.97E-04	0	3.80E-05	0	3.10E-05	0	0	5.00E-05	0.001183	0
1.19E-04	5.30E-05	0.016822	0	0	0.004535	0	0.003681	0	5.40E-05	0	0	9.36E-04
1.70E-05	9.20E-05	0	8.80E-05	0	3.60E-05	0.002216	0.002202	0	2.90E-05	0	0	5.30E-05
0	0	3.10E-05	2.50E-05	0	0	0	0	1.30E-04	2.30E-04	0.001206	0.011008	3.11E-04
0	8.20E-05	3.92E-04	0	3.01E-04	0	0	1.70E-05	0	0	0	0	0.001082
1.39E-04	3.55E-04	1.02E-04	0	3.97E-04	2.80E-05	0	2.18E-04	0	2.74E-04	0	4.91E-04	0
0	0	0	0	0	0	0	0	0	0	0	2.20E-05	0
0	0	0	0	0	0	0	0	0	0	0	2.80E-05	0
0	1.86E-04	2.74E-04	6.40E-05	1.69E-04	0	0	0.001141	0	4.63E-04	0	0	0
3.60E-05	0	0	9.70E-05	0	0	0	1.70E-05	0	0	0	5.25E-04	0

3.90E-05	0	6.62E-04	0	1.22E-04	0	0	0.001071	2.10E-05	2.60E-05	0	0.001177	0
0	0	0	0	0	0	0	1.20E-05	0	0	0	2.56E-04	0
2.10E-05	0	0	0	0	0	0	1.00E-05	0	0	0	3.93E-04	0
4.30E-05	0	0	0.064038	0	0	9.60E-05	0	0	1.32E-04	0	0	3.30E-05
3.90E-05	0	7.10E-05	0	0	0	0	0.005824	0	3.20E-05	0	0.001138	6.20E-05
0	0	0	0	0	0	0	0	0	0	0	3.75E-04	0
0	0	0	0	0	0	0	0	0	0	0	0	0
0	0	0.001563	0	2.15E-04	0.001775	0	0	0	1.71E-04	0	0	4.53E-04
1.80E-05	5.16E-04	6.67E-04	0	0.001159	6.40E-05	1.68E-04	0.003872	3.17E-04	0.006837	0	2.50E-05	0.0022
0	0.003442	7.15E-04	0	0.00142	0	2.20E-05	4.57E-04	0	0.012427	2.60E-05	0	4.69E-04
0	0	0	0	0	0	0	0	0	0	0	1.10E-04	0
0	0	4.20E-05	8.56E-04	0	0	4.50E-05	0.00124	0	0	0.029704	9.00E-05	0
0	1.80E-04	2.00E-05	0	5.12E-04	2.11E-04	0	1.23E-04	2.14E-04	2.40E-05	0	0	0
7.94E-04	4.60E-05	0	0.001517	0	5.11E-04	1.77E-04	3.81E-04	0	0	0	0.01417	0
0	0	0	0	0	0	0	0	0	0	0	0	0
1.60E-05	0	5.97E-04	0	6.30E-05	0	6.40E-04	0	0	0.006718	0	0	0.002556
1.02E-04	2.03E-04	3.54E-04	0	0	2.76E-04	2.00E-05	0.017112	0	3.70E-05	0	2.50E-05	0
0	1.47E-04	0	0	0	0	0	0	0	0	0	0	0
0	0	0	0	0	0	0	0	0	0	0	1.80E-04	4.00E-05
0	0	4.30E-05	0	0	0	0	0	0	0	0	0	5.90E-05
0	0	0	0	0	0	0	0	0	0	0	0	0
0	0	0	0	0	0	0	0	0	0	0	0	0
0	0	0	0	0	0	0	0	0	0	0	0	0
8.96E-04	0	0	0.00114	0	1.42E-04	3.27E-04	3.91E-04	0	0	0	0.00971	0
3.10E-05	0	0	0	0	0	0	0	0	0	0	4.82E-04	0
7.17E-04	0	0	8.71E-04	0	1.78E-04	1.77E-04	3.19E-04	0	4.10E-05	0	0.006634	0
0	0	0	1.70E-05	0	0	2.20E-05	5.00E-06	0	0	0	5.70E-05	0
0.001747	0	2.20E-05	0.001229	0	0	0.001357	4.54E-04	0	0	2.45E-04	1.03E-04	0
0	0	0	0	0	0	0	0	0	0	0	0	0
0	0	0	1.90E-05	0	0	2.40E-05	3.10E-05	0	0	0	0	0
0.001415	0.005146	0.015259	0.001573	0.00204	0.006765	0.004941	0.024097	0.007808	0.00582	0.001436	0.01576	0.003246

CC59	CC20	CC37	CC13	CC71	CC45	CC38	CC30	CC25	CC27	CC60	CC46	CC57
0.077326	0.069573	0.06545	0.064771	0.062549	0.059831	0.056623	0.055179	0.042724	0.042538	0.03756	0.037142	0.036046
0.217827	9.57E-04	0.246709	0.001354	0.089015	0.028616	5.46E-04	0.138098	0.370428	0.056327	0.025544	0.004042	0.032964
0.197324	0.040097	0.209709	0.112589	0.04533	0.441029	0.035343	0.149044	0.069127	0.466505	0.030685	0.435723	0.295832
0.045321	0.004572	0.124467	0.653314	0.047416	0.055456	0.860641	0.197094	0.09659	0.037522	0.028454	0.015411	0.048714
0.037901	0.06389	0.017757	0.00335	0.2356	0.03933	9.48E-04	0.274191	0.115868	0.094719	0.194908	0.010765	0.054419
0.0602	0.003467	0.012449	0	0.232251	0.00751	6.69E-04	0.007477	0.026165	0.040065	0.551247	0.004856	0.042217
0.006262	6.34E-04	8.85E-04	4.54E-04	7.76E-04	0.002663	7.60E-05	0.005183	0.001213	0.001468	8.10E-04	0.001399	0.003914
0.012852	0.001268	0.018193	0.060577	0.002683	0.057417	8.00E-05	0.001076	0.002413	0.036701	0.024294	0.025129	0.011537
0.027279	1.10E-04	0.006328	0.005639	0.136484	0.030606	2.28E-04	0.004374	0.002972	0.009132	6.29E-04	0.019244	0.021732
0.012906	0.601804	0.085924	0.001631	0.004158	0.011453	0.014419	0.07772	0.126735	0.012061	0.004541	0.004333	0.049164
0.045891	0.042043	0.034898	0.012721	0.031889	0.02348	6.90E-05	0.009946	0.026151	0.018927	7.87E-04	0.010213	0.043992
0.022361	0.002453	0.00629	0.049934	0.003306	0.041795	0	0.013059	0.059029	0.00472	0.024975	0.028358	0.01184
0.155197	1.80E-05	0.043463	0	0.02904	0.095873	4.68E-04	0.001595	0.003449	0.043363	0.003151	0.063588	0.044638
0.003527	0.002588	0.00657	4.55E-04	0.02053	0.00259	6.57E-04	0.007161	0.006505	0.004036	0.005523	0.001227	0.002927
0.001602	0.018661	0.002354	0	0.007912	0.004193	1.44E-04	0.005365	7.00E-04	0.026347	0.001152	0.043358	0.002067
0.020854	0.003193	0.012638	0.010024	0.011619	0.007737	8.00E-05	0.002019	0.003224	0.026387	3.82E-04	0.009069	0.004232
0.009843	5.91E-04	0.001238	5.67E-04	0.010999	0.002383	0	0.003314	9.64E-04	0.004742	6.90E-04	0.014975	0.004602
0.005488	0.019488	0.001267	0.001633	4.20E-05	0	7.40E-05	3.50E-04	2.55E-04	2.50E-05	0	0	0.054507
1.70E-05	0	0	0	0	4.20E-05	0	0	0	0	0	0	0
0	0	0	0	2.00E-05	3.30E-04	0	0	0	3.14E-04	0	9.70E-05	0
0	1.90E-05	0.006264	5.70E-05	0.001123	0.004533	0	1.64E-04	0.002665	0.00322	8.65E-04	0.155298	0.050106
0	0	0.003151	0	0	0.001732	0	3.13E-04	3.49E-04	0.002053	2.45E-04	0	1.17E-04
0.004583	0.002215	0.001416	1.61E-04	0.00212	0.004327	9.50E-04	0.001705	0.001934	0.004065	0.005258	0.005043	0.003698
0.003502	0.049738	0.004736	0.006136	0.004292	0.003348	0	0.002029	0.001424	0.001359	0.004312	0.007939	0.003588
5.20E-04	4.22E-04	0.003	0.003256	1.41E-04	0.003318	1.57E-04	0.011609	0.002272	0.006952	0.002854	0.01021	0.003246
2.40E-05	1.98E-04	6.90E-05	9.80E-05	0	1.76E-04	0	0	0	1.60E-05	0	5.30E-04	0.00311
9.55E-04	0.015402	0.001446	0.001302	0.005499	0.001619	1.43E-04	0.003423	0.00237	0.002529	2.13E-04	0.001185	0.001341
0.002963	0.008991	0.002626	0	8.56E-04	0.001387	0.001226	0.002548	0.001589	0.002302	0.012056	7.13E-04	0.001877
3.60E-05	0.001714	2.65E-04	3.70E-04	0.001301	7.96E-04	6.32E-04	1.85E-04	3.83E-04	7.13E-04	5.30E-05	0.001449	0.003739
0.002701	1.50E-05	4.78E-04	0	2.34E-04	0.002711	0	0.003209	0.001221	0.005378	9.55E-04	8.30E-04	9.43E-04
6.20E-05	2.80E-04	0	4.20E-05	0	0	0	4.20E-05	0	3.00E-05	0	8.07E-04	1.46E-04
0.003268	1.60E-05	8.43E-04	0	1.10E-05	0.002516	0	3.70E-05	0	9.21E-04	0.001395	0	0
0.001587	0.009964	0.002053	5.40E-05	0.002466	0.00266	0	0.002661	0.002404	0.001356	0.002197	5.25E-04	0.001369
7.57E-04	0.009431	0.001142	4.47E-04	3.14E-04	9.94E-04	2.14E-04	0.001515	3.71E-04	0.002036	2.00E-04	0.001149	0.0015
0	0	0	0	1.60E-05	0.003129	0	3.70E-05	0	0.004547	0	0	0.002255
0	0	0	0	0	0	0	0	0	0	0	0	2.70E-05
6.10E-05	0.004953	1.41E-04	4.22E-04	7.10E-05	0.003981	0	6.64E-04	2.36E-04	0.002096	0.013453	3.29E-04	0.002362
1.50E-05	0.003066	1.33E-04	2.57E-04	1.20E-04	1.18E-04	8.30E-05	1.08E-04	0.003752	9.54E-04	4.50E-05	0	0.062472
0.001941	1.60E-04	3.32E-04	0	1.34E-04	5.67E-04	0	1.61E-04	0	0.002479	3.86E-04	0.013663	7.56E-04
0	2.58E-04	0	5.80E-05	0	0	0	0	0	0	0	9.30E-05	2.71E-04
1.04E-04	3.50E-05	1.38E-04	0	0	0	7.00E-05	0	2.50E-04	0	5.80E-05	4.06E-04	3.20E-04
8.40E-05	0	0.001314	0	3.90E-05	0.00333	0	0.001348	0.001284	8.23E-04	5.10E-05	0.031132	0.020957
2.94E-04	7.30E-05	0	9.70E-05	0	0.001045	0	2.43E-04	0	0.001333	0	9.90E-04	0.002216
0	0.001702	0.023675	2.06E-04	0	0.015956	0.001018	0.002552	0.004286	0.004962	3.42E-04	0	0.00263
0	7.29E-04	0.001586	9.80E-05	2.30E-05	5.69E-04	0	0	1.17E-04	1.41E-04	2.13E-04	8.25E-04	0.004252
7.38E-04	0	2.77E-04	0	0.002625	0.00309	0	3.70E-05	0	0.001096	0	0.001206	0.001807
0	1.57E-04	0	4.40E-05	0	0	0	0	0	0	5.60E-05	9.90E-05	8.10E-05
0.001568	1.70E-04	0.002171	0	8.48E-04	0.001416	1.49E-04	4.56E-04	7.96E-04	0.001729	0.005293	0	0.002219
3.50E-05	3.90E-05	7.00E-05	0	0	0	6.27E-04	4.70E-05	2.34E-04	0	0	0	5.61E-04
0	3.30E-05	0.015138	5.20E-05	0.002777	0.007132	0	0	0	9.01E-04	0	7.39E-04	6.68E-04
0	1.69E-04	6.62E-04	5.50E-05	0	0.001016	0	1.07E-04	5.92E-04	9.21E-04	0.002939	0	0.002121
1.70E-05	3.40E-05	0	5.61E-04	3.31E-04	0	6.60E-05	0	0	8.08E-04	2.30E-04	0.011064	0.006826
0	1.60E-05	0	1.02E-04	0	0	0	0	0	0	0	0	0
0	6.20E-05	0	0	0	0	0	0	0	0	0	0	0
0.002277	4.58E-04	6.10E-05	0	5.90E-05	0.002063	0	0	0	0.007163	5.20E-05	0.004342	0.00529
0	0	0	0	0	0	0	0	0	0	0	4.97E-04	1.47E-04

6.40E-05	0	0	0	1.70E-05	2.00E-04	0	3.26E-04	0.00107	0.002303	0.001703	1.04E-04	3.27E-04
2.30E-05	1.50E-05	0	0	0	0	0	0	0	0	0	0	0
0	1.58E-04	0	1.02E-04	0	0	8.00E-05	0	0	0	0	1.09E-04	2.25E-04
0	0	1.34E-04	0	0	4.00E-05	7.90E-05	0	0	0	0	5.98E-04	0.003003
4.00E-05	0	0	0	1.50E-05	0	7.40E-05	0	1.18E-04	0	0	0	1.44E-04
0	3.90E-05	0	2.05E-04	0	0	0	0	0	0	0	0	4.10E-05
0	0	0	0	0	0	0	0	0	0	0	0	0
7.80E-04	0	0	0	0	0	0	5.07E-04	0	9.14E-04	0.001705	0	7.60E-05
1.56E-04	1.31E-04	0.002927	0.001014	6.00E-05	7.56E-04	7.50E-05	0.001096	6.99E-04	8.70E-05	3.25E-04	0	2.20E-04
3.20E-05	4.49E-04	0.009045	1.95E-04	1.70E-05	6.09E-04	0.001955	0.003281	0.00794	2.90E-05	3.77E-04	0	0
0	0	0	0	0	0	0	0	0	0	0	0	8.10E-05
0	0	0	4.70E-05	0	0	0.014679	0	0	0	0	0.001021	1.28E-04
1.05E-04	0	0	0	1.01E-04	5.10E-05	0	4.30E-05	0	6.10E-05	1.12E-04	0	2.73E-04
2.76E-04	0.00259	0	0	0	0	0	0	0	0	0	0	0.003265
0	0	0	1.02E-04	0	0	0	0	0	0	0	0	0
4.36E-04	1.60E-05	0.005419	1.12E-04	0	8.63E-04	0	0.00184	0.00194	0	7.68E-04	0	2.68E-04
5.87E-04	0	0	0	0	4.30E-05	0.002586	0	0	2.80E-05	5.60E-05	0.003603	0.003159
0	0	0	0	0	0	0	0	0	0	0	0	7.70E-05
0	0	0	3.80E-05	0	0	0	0	0	4.00E-05	3.73E-04	0	0
0	0	0	0	0	1.35E-04	0	0	0	4.60E-05	0	0	0
0	0	0	0	0	0	0	0	0	0	0	0	0
0	0	0	0	0	0	0	0	0	0	0	0	0
0	0	0	0	0	0	0	4.00E-05	0	9.80E-05	0	0	0
1.40E-04	0.001623	1.28E-04	0	0	0	0	3.70E-05	0	0	0	0	0.004147
0	3.50E-05	0	0	0	0	0	0	0	0	0	0	1.95E-04
1.86E-04	0.001633	0	2.13E-04	0	0	0	0	0	0	0	0	0.002071
3.50E-05	1.48E-04	0	0	0	0	0	0	0	0	0	0	0
8.40E-05	5.10E-05	0	4.60E-05	0	0	0	0	0	8.20E-05	0	8.06E-04	3.00E-05
0	0	0	0	0	0	0	0	0	0	0	0	0
0	0	0	0	0	0	0	0	0	0	0	0	0
0.008986	0.007186	0.012571	0.005038	0.002771	0.01144	0.004072	0.005385	0.005192	0.00753	0.005528	0.013767	0.023908

CC42	CC73	CC36	CC16	CC39	CC52	CC62	CC65	CC55	CC32	CC07	CC63	CC68
0.034295	0.033817	0.031369	0.027436	0.027075	0.02557	0.025261	0.024155	0.02291	0.01843	0.017141	0.015771	0.014059
0.005554	0.711121	0.13721	0.301231	0.003982	0.079531	0.579741	0.759284	0.002176	0.790009	0.084307	0.532785	0.365039
0.109891	0.010805	0.225342	0.378986	0.583643	0.173175	0.024294	0.054896	0.039923	0.008253	0.003076	0.013093	0.365815
0.395621	0.017442	0.069217	0.017096	0.02374	0.046841	0.052944	0.013928	0.28501	0.019817	0.060292	0.022008	0.066379
0.007675	0.053373	0.02632	0.032461	0.053598	0.091876	0.151044	0.054822	1.87E-04	0.01184	0.599875	0.034455	0.061025
3.57E-04	0.043373	0.059082	0.007419	0.008524	0.017147	0.019187	0.011259	5.14E-04	0.037801	0.001624	0.139224	0.008444
5.75E-04	0.001396	0.004775	5.92E-04	1.96E-04	6.20E-04	9.85E-04	0.001714	1.91E-04	5.68E-04	7.30E-05	3.11E-04	0.001231
0.381462	0.003078	0.075518	0.002349	7.54E-04	0.005075	0.005053	9.73E-04	0.087458	0.044313	0	0.020669	0.00592
0.023787	0.051714	0.0778	0.00572	0.013419	0.045217	1.07E-04	0.003428	1.39E-04	4.37E-04	1.17E-04	0.002654	0.018393
2.39E-04	0.004358	0.048949	0.004543	0.00619	0.171284	0.08797	0.005947	0.25623	0.001118	0.203554	0.010473	0.002965
0.021027	0.004477	0.029724	0.006277	0.024818	0.022654	0.015527	0.007153	0.175148	0.001938	0.015237	0.013597	0.006435
7.80E-05	0.003504	0.026293	8.53E-04	0.005976	0.022159	0.002546	0.00806	4.50E-05	0.044075	0	0.071238	0.003295
2.04E-04	0.007192	0.053007	0.102153	0.210469	0.057831	5.41E-04	9.55E-04	0	5.52E-04	3.10E-05	0.002688	0.004581
0.001008	0.009116	8.06E-04	0.001893	0.003905	0.008707	0.011195	0.005957	4.60E-05	0.003412	0.001336	0.032873	0.010278
7.30E-05	0.00169	0.007021	6.30E-05	0.001695	7.59E-04	4.15E-04	0.00192	0	0.001022	8.84E-04	0.001873	0.002192
8.08E-04	0.006472	0.017238	0.005946	3.10E-05	0.001591	0.001196	0.001533	0.075448	1.50E-04	0.001687	0.003185	0.001699
0.001364	0.005156	0.003262	0.009846	0.005491	0.006919	3.21E-04	2.02E-04	0.01097	1.47E-04	1.55E-04	1.10E-04	0.001134
5.57E-04	0.003051	0.023442	0.026706	1.77E-04	0.044328	1.95E-04	5.93E-04	0.006941	1.52E-04	0	1.06E-04	0
0	0	0	0	0	0	0	0	0	0	0	6.00E-05	0
0	0	0.042805	1.50E-05	0.001029	1.65E-04	0	5.20E-05	0	0	0	0	0.001313
2.98E-04	0	0.004058	3.70E-05	0	0.018086	6.50E-04	0.008772	0.013021	8.26E-04	0	0.004073	7.40E-04
0	0	1.96E-04	0	0	1.87E-04	3.90E-05	0.001544	0	7.00E-05	0	0.003023	5.70E-05
0	0.001012	0.001066	0.001719	0.002459	0.006437	0.003323	0.001181	1.70E-05	0.001237	5.30E-04	0.016842	0.002129
0.001297	0	0.001085	0.005375	0.001618	7.20E-05	3.13E-04	0.001126	4.30E-05	0.001248	2.83E-04	0.006541	0.001001
0.004666	8.50E-05	1.57E-04	0.001648	0.001927	0.003377	4.59E-04	0.00293	9.16E-04	7.69E-04	0.001265	0.001126	0.009171
0	0	7.66E-04	0.002583	6.80E-05	0.002366	0	0	0	0	0	0	0
0.002519	0.002802	0.001416	2.12E-04	7.82E-04	1.27E-04	9.11E-04	8.41E-04	4.32E-04	3.54E-04	7.30E-05	0.003432	1.30E-04
0	9.05E-04	4.87E-04	7.05E-04	6.89E-04	0.003176	4.20E-04	1.39E-04	0	0.002523	0.001011	0.002549	2.84E-04
1.29E-04	1.10E-04	2.30E-05	0.001002	0	5.94E-04	0	4.30E-05	0.003563	7.00E-05	1.17E-04	0	0
0	1.62E-04	0.001435	5.69E-04	3.46E-04	0.001362	3.23E-04	0.003768	2.40E-05	2.92E-04	2.70E-05	1.73E-04	0.004012
0	0	1.69E-04	3.31E-04	0	0.002606	0	0	0	0	0	0	0
0	6.30E-04	0	1.06E-04	0	2.60E-04	0	0.002213	0	9.45E-04	2.69E-04	7.30E-05	0.009406
0	0.001426	4.37E-04	1.60E-04	2.80E-05	6.14E-04	0.001366	0.001329	0	3.85E-04	4.32E-04	0.014493	0.001059
1.48E-04	0.001351	3.85E-04	0.001483	5.20E-05	3.19E-04	2.49E-04	1.65E-04	1.80E-05	8.10E-05	1.75E-04	1.04E-04	1.27E-04
0	0.00371	0.002685	2.10E-05	6.70E-05	4.78E-04	3.40E-05	0	0	0	0	0	5.03E-04
0	0	1.70E-05	0	0	7.80E-05	0	0	0	0	0	0	0
0	7.52E-04	6.44E-04	3.87E-04	4.04E-04	0.001951	1.99E-04	8.66E-04	0	7.60E-05	0	0.008257	0
6.36E-04	1.24E-04	6.57E-04	0.002102	2.11E-04	0.001267	0	4.60E-05	0.007951	0	0	4.51E-04	0
0	5.00E-05	0.001829	0.006074	0.001836	0.013007	3.40E-05	0	0	0	0	4.70E-05	1.33E-04
0	0	2.49E-04	0.001043	5.30E-05	0.001045	0	0	0	0	0	0	0
0	2.20E-05	2.60E-05	2.02E-04	3.82E-04	0.002009	2.06E-04	0	0	0	0	5.30E-05	5.40E-05
3.76E-04	0.008139	6.82E-04	0.019398	3.26E-04	0.004182	0.001983	0.005027	1.84E-04	8.67E-04	0.002965	0.00256	3.05E-04
0	0	0	4.80E-05	4.46E-04	0.002862	0	6.77E-04	0	0	0	0	8.87E-04
7.00E-05	1.63E-04	2.27E-04	0	0	1.50E-05	0.00296	4.24E-04	0.001522	0	6.61E-04	2.90E-04	0.005942
0	1.60E-04	6.26E-04	0.003333	1.30E-04	9.80E-05	0	0	2.30E-05	0	0	5.58E-04	6.00E-05
0	0.001027	0.002758	1.23E-04	3.31E-04	2.91E-04	1.54E-04	8.00E-05	0	0	0	1.70E-04	0
0	0	0	8.90E-05	1.29E-04	4.00E-04	0	0	8.70E-05	0	0	0	0
7.80E-05	4.19E-04	6.94E-04	0.001026	2.66E-04	0.017771	2.84E-04	0	0	7.43E-04	0	0.002822	1.16E-04
0	1.07E-04	2.12E-04	6.46E-04	6.31E-04	0.001951	0	0	3.00E-05	0	0	4.60E-05	0
0	0.002418	7.36E-04	3.50E-05	0	0.038302	3.00E-05	3.55E-04	0.001085	0	0	0	0
0	0	0.001282	0	7.10E-05	1.13E-04	2.43E-04	0	0	4.54E-04	0	3.74E-04	0
0	2.10E-05	3.47E-04	2.23E-04	7.81E-04	0.002833	1.14E-04	0	0	0	4.00E-05	2.90E-05	0
0	0	3.80E-05	2.94E-04	0	9.20E-05	0	0	0	0	0	0	0
0	0	7.20E-05	2.46E-04	0	2.50E-05	0	0	0	0	0	0	0
0	0	1.18E-04	3.10E-05	0.001036	0.006989	0	0	3.70E-05	0	0	1.38E-04	0
0	0	1.20E-05	1.10E-05	0	1.81E-04	0	0	2.10E-05	0	0	0	0

0	0	4.39E-04	1.53E-04	9.81E-04	0.001979	5.39E-04	0	0	0	0	0.008984	0
0	0	1.50E-05	0	0	5.14E-04	0	0	0	0	0	0	0
0	0	4.43E-04	0.001159	0	9.15E-04	0	0	0	0	0	0	0
0	5.00E-05	3.00E-04	2.03E-04	1.78E-04	2.12E-04	4.00E-05	2.09E-04	2.20E-05	0	0	0	0
6.44E-04	0	0	1.83E-04	2.20E-05	3.18E-04	4.00E-05	0	0	0	4.40E-05	0	0
0	0	2.35E-04	3.37E-04	0	2.26E-04	0	0	0	0	0	0	0
0	0	0	0	0	0	0	0	0	0	0	0	0
5.70E-05	3.10E-05	1.28E-04	0	0	0	0	0	0	6.90E-04	0	7.48E-04	2.58E-04
0.002781	9.20E-05	1.18E-04	0	6.40E-05	0	3.96E-04	3.26E-04	0.001392	7.48E-04	1.72E-04	2.51E-04	0.00172
7.70E-05	0	0	0	0	0	0.002939	3.68E-04	0.001345	2.41E-04	0.001545	9.20E-05	0.00143
0	0	3.20E-05	1.11E-04	0	1.23E-04	0	0	0	0	0	0	0
0	0	0	0.001069	1.74E-04	1.36E-04	0	0	0	0	0	0	0
0	1.40E-05	0	7.00E-05	5.80E-05	4.20E-05	5.48E-04	0.002153	0	0	0	2.29E-04	6.40E-05
0	1.46E-04	0.001663	0.001392	1.84E-04	0.002453	0	1.80E-05	9.65E-04	0	0	0	0
0	0	0	0	0	2.10E-05	0	0	0	0	0	0	0
1.40E-04	9.20E-05	1.19E-04	5.40E-05	0	0	1.78E-04	0.005578	6.10E-05	0.001397	0	6.33E-04	0.009864
0	0	1.80E-05	0.002153	0.002026	7.55E-04	0	0	0	7.30E-05	3.92E-04	0	0
0	0	0	0	3.50E-05	5.60E-05	0	0	0	0	0	0	0
0	0	0	6.60E-05	0	6.20E-05	0	0	0	0	0	0	0
0	0	0	0	0	0	0	5.70E-05	0	0	0	0	0
0	0	0	0	0	0	0	0	0	0	0	0	0
0	0	0	0	0	0	0	0	0	0	0	0	0
0	0	0	0	0	0	0	0	0	0	0	0	0
0	4.05E-04	0.001355	0.001015	2.59E-04	0.002036	0	0	6.37E-04	0	0	6.50E-05	5.80E-05
0	0	1.40E-05	1.14E-04	0	2.10E-05	0	0	0	0	0	0	0
6.30E-05	1.15E-04	9.58E-04	8.10E-04	5.70E-05	0.001951	3.30E-05	7.10E-05	6.90E-04	0	0	2.10E-05	0
0	0	1.60E-05	2.70E-05	0	7.60E-05	0	0	4.60E-05	0	0	0	0
0	0	1.50E-05	9.00E-04	0	0.009272	0	0	0	7.80E-05	0	0	1.37E-04
0	0	0	0	0	0	0	0	0	0	0	0	0
0	0	0	0	0	0	0	0	0	0	0	0	0
0.001446	0.002325	0.009361	0.007337	0.006181	0.02186	0.002471	0.002863	0.002532	0.001799	6.10E-04	0.00358	0.010156

CC58	CC21	CC11	CC28	CC26	CC43	CC51	CC03	CC64	CC12	ID...75	SC29	SC01
0.013332	0.012501	0.012492	0.011237	0.010957	0.006553	0.004665	0.002963	0.002028	1.72E-04	Prevotella	0.636665	0.561803
0.196766	0.162214	8.60E-05	0.11793	0.003727	0.189964	0.044193	0.508	0.001587	0.036779	Neisseria	6.79E-04	0.048741
0.354804	0.226127	0.297854	0.368676	0.667097	0.172731	0.223875	0.180623	0.965499	0.71271	Streptococ cus	0.001505	0.046059
0.026582	0.009135	0.174054	0.019287	0.081817	0.30003	0.018957	0.015638	0.002343	3.88E-04	Veillonella	0.04681	0.104149
0.224234	0.053143	0.039727	0.0569	0.059084	3.23E-04	0.041432	0.010673	8.27E-04	0.140442	Leptotrichi a	0.132002	0.039503
0.00323	0.048487	1.51E-04	0.005565	0.003587	6.63E-04	0.008091	0.00801	5.97E-04	5.00E-05	Fusobacteri um	0.001315	0.015264
4.17E-04	0.001661	2.20E-04	6.20E-05	5.54E-04	1.36E-04	1.54E-04	1.40E-05	6.74E-04	4.30E-05	Alloprevot ella	3.91E-04	0.003585
0.017635	0.080289	0.003154	0.013865	1.40E-04	0.00394	0.001084	0.010896	8.83E-04	1.00E-04	Porphyrom onas	2.53E-04	0.007024
0.002867	0.070842	1.54E-04	0.003016	0.018276	1.99E-04	0.025456	0.100207	0.002008	0.063255	Haemophil us	2.50E-05	2.90E-05
0.013109	0.004731	0.043466	0.006425	0.007338	0.004313	0.013766	0.00432	0.001166	0.003466	Actinomyc es	0.019523	0.025914
0.028966	0.010554	0.363835	0.241687	0.001762	0.001203	0.043269	0.021006	3.00E-04	0.022608	Rothia	2.49E-04	0.014207
0.020034	0.015884	0.01709	0.012629	1.17E-04	0.006544	0.003318	0.006524	1.36E-04	2.70E-05	Capnocyto phaga	0.002242	0.00705
0.013783	0.062756	2.20E-05	0.062085	0.063669	1.70E-05	0.466673	0.046322	0.009627	0	Gemella	4.17E-04	0.00271
0.001903	0.004046	6.60E-05	0.001881	0.002757	4.30E-05	0.001175	3.51E-04	2.65E-04	3.20E-05	Lachnoana erobaculum	0.072284	0.008444
0.001398	0.001354	0	0.003671	2.30E-04	8.00E-06	1.25E-04	1.91E-04	1.00E-04	1.13E-04	TM7x	0.029504	0.047462
0.003836	0.015093	0.014161	0.007699	0.008487	0.020363	0.02599	0.004384	2.61E-04	0	Granulicate lla	0	0.006942
6.62E-04	7.23E-04	1.05E-04	3.34E-04	9.75E-04	8.10E-04	7.94E-04	0.007067	6.90E-05	0	Campyloba cter	0.00175	7.30E-05
6.67E-04	0.071288	7.78E-04	0.00124	4.30E-05	0.044805	0.008183	0.0332	2.60E-05	2.49E-04	Halomonas	1.09E-04	5.29E-04
0	0	0	0	0	0	0	0	0	0	Moraxella	0	0
0	0.016553	0	5.90E-05	0	0	0.001862	0	1.90E-05	0	Actinobacil lus	0	0
0.025152	0.022576	0	0.001709	4.70E-05	0.123433	0	0	5.20E-05	0.012367	Lautropia	5.80E-05	0
0	5.78E-04	0	0.000649	7.80E-05	0	0	0	2.00E-05	0	g__Abscon ditabacteria	0	0
0.001096	0.005187	8.80E-05	0.00457	0.001088	0	0.00112	1.21E-04	8.60E-05	0	Oribacteriu m	0.015178	0.002645
1.13E-04	0.00324	5.65E-04	0.007618	2.70E-05	2.10E-05	8.84E-04	9.23E-04	4.10E-05	6.90E-05	Peptostrept ococcus	0.005589	0.006084
3.64E-04	1.91E-04	6.77E-04	3.90E-04	2.41E-04	8.10E-05	0.001685	4.22E-04	5.50E-05	0	Selenomon as	0.006932	3.54E-04
0	9.30E-05	3.80E-05	0	0	0.001497	7.30E-05	2.36E-04	0	2.10E-05	Bacteroide s	3.50E-04	0
1.51E-04	0.001387	3.90E-05	2.96E-04	5.75E-04	3.10E-05	2.15E-04	3.00E-05	1.72E-04	2.80E-05	Solobacteri um	0.003437	0.005956
2.97E-04	6.03E-04	0	0.012976	0.001985	5.00E-06	1.31E-04	9.60E-05	1.25E-04	0	Stomatobac ulum	0	5.87E-04
1.40E-04	0	0	0	1.65E-04	2.92E-04	0	8.00E-05	1.10E-05	0	Megasphae ra	7.70E-05	0.002939
6.99E-04	5.48E-04	0	0.001102	1.06E-04	2.80E-05	1.30E-05	6.82E-04	1.77E-04	0	Clostridia_ UCG-014	0.004765	0.003653
0	1.21E-04	6.70E-05	0	0	8.97E-04	6.70E-05	0.001337	0	2.43E-04	Pseudomon as	1.90E-05	0
0.00118	6.40E-05	0	0	0	0	1.42E-04	1.23E-04	0	0	Lachnospir aceae_uncu	0	0
7.31E-04	4.17E-04	3.63E-04	0.001033	4.99E-04	0	3.39E-04	0	1.65E-04	0	[Eubacteriu m]_nodatu	0.006111	0.015681
6.75E-04	2.33E-04	3.80E-05	0	7.00E-05	5.60E-05	7.10E-05	1.23E-04	2.30E-05	0	Atopobium	7.95E-04	0.003357
0.002426	0.005695	0	1.26E-04	1.59E-04	0	0	3.00E-04	4.90E-05	0	Aggregatib acter	0	0
0	0	0	0	0	0	0	0	0	0	Muribacula ceae	0	0
0.002458	0.001459	1.50E-04	5.10E-05	0	5.50E-05	3.47E-04	6.70E-05	5.90E-05	3.90E-05	Tannerella	0.002198	0.007475
0.003513	0.003806	0	4.25E-04	3.90E-05	0	5.70E-05	0	4.00E-05	0	Corynebact erium	0	0
0	8.00E-05	0	3.61E-04	7.46E-04	1.50E-05	0.002228	0.003218	4.00E-05	0	Treponema	0	0
0	1.70E-05	3.10E-05	0	0	6.08E-04	6.90E-05	2.30E-04	0	0	Faecalibact erium	6.80E-05	0
0	1.56E-04	0	0	2.92E-04	1.50E-05	7.80E-05	1.39E-04	0	0	Lachnospir aceae_uncl	0.003949	5.02E-04
0.00183	0.002258	0	0.001036	9.30E-05	0.010504	0.001733	0.003372	0	0	Neisseriace ae_unclassi	0	0
2.26E-04	0	0	0	2.70E-05	0	1.90E-05	0	0	0	Saccharimo nadales	0	0
0.003952	0.019554	0.001544	0.002819	0.00207	0	1.28E-04	0	0.006049	1.01E-04	Lactobacill ales_unclas	2.80E-05	0.001045
0	0	0	7.00E-05	0	9.00E-06	6.25E-04	0	2.50E-05	2.40E-05	Parvimonas	0	3.10E-05
5.22E-04	0.002881	9.60E-05	6.80E-05	3.40E-05	0	0.001401	9.24E-04	1.30E-05	0	Bergeyella	5.10E-05	0
0	4.66E-04	0	0	0	3.34E-04	2.00E-05	2.35E-04	0	0	Rhodococc us	0	0
5.10E-05	2.43E-04	0	2.91E-04	0	0	4.40E-05	1.39E-04	0	0	Johnsonella	0	0
3.07E-04	0.002956	0	7.29E-04	0.043009	0	0.040803	0.002927	0	1.51E-04	F0332	0	0
4.84E-04	0.04096	0.007064	2.62E-04	0	0.053043	0.002491	0	1.97E-04	0	Abiotrophi a	0	0
1.17E-04	0	0	0.001203	4.00E-05	0	0	0	4.90E-05	0	Filifactor	0	7.90E-05
0	0	3.80E-05	2.43E-04	0	6.00E-05	2.50E-05	1.69E-04	0	2.60E-05	Dialister	0	3.80E-05
0	0	0	0	0	3.50E-05	0	0	0	0	Lachnospir aceae_NK4	0	0
0	0	0	0	0	1.20E-05	0	0	0	0	Fusicatenib acter	0	0
0.008292	2.40E-05	0	5.30E-05	0	1.70E-05	0	0	2.01E-04	0	Saccharimo nadaceae	2.60E-05	1.21E-04
0	9.80E-05	0	0	0	1.65E-04	0	1.04E-04	0	1.90E-05	Acinetobac ter	0	0

0	1.52E-04	0	0	0	0	4.59E-04	1.70E-05	0	3.30E-05	F0058	0	1.30E-04
0	1.85E-04	3.50E-05	0	0	3.11E-04	0	2.40E-05	0	0	Sphingomo nas	0	0
0	5.10E-05	0	0	3.20E-05	4.48E-04	5.30E-05	5.20E-05	0	1.31E-04	Bifidobacte rium	0	4.50E-05
0	4.88E-04	0	6.70E-05	3.80E-05	0.02683	5.00E-05	4.40E-05	1.20E-05	0	Kingella	2.50E-05	0
0	0	1.63E-04	0	1.18E-04	2.90E-05	0	1.66E-04	3.20E-05	7.35E-04	Lactobacill us	0	0
0	1.03E-04	0	0	0	1.67E-04	3.80E-05	9.10E-05	0	0	Roseburia	2.60E-05	2.50E-05
0	0	0	9.80E-05	0	0	0	0	0	0	Leptotrichi aceae_uncu	0	0
0	0	0	0	1.08E-04	0	6.80E-05	0	1.20E-05	0	Amnipila	0	0
0.002499	0	0.010514	0.012878	7.08E-04	0	7.10E-05	0	3.41E-04	6.26E-04	Bacteria_u nclassified	0	7.01E-04
0.001341	0	5.38E-04	0.001608	0.002061	1.00E-05	0	0	0.001202	1.19E-04	Actinobact eria_unclas	0	6.03E-04
0	0	0	0	0	6.80E-05	0	0	0	0	Megamona s	0	0
0	0	0	0	0	1.25E-04	0	0	0	0	Scardovia	7.80E-05	0
0	0	0	0	0	0	1.67E-04	1.02E-04	0	0	Butyrivibri o	2.30E-05	0
0	0.004395	4.10E-05	5.80E-05	0	0.002137	6.36E-04	0.002294	0	4.90E-05	Pelagibacte rium	0	4.60E-05
0	0	0	0	0	0	0	0	0	0	[Eubacteriu m]_rumina	0	0
0.010326	0	0	0.002729	4.89E-04	0	2.70E-05	1.40E-05	0.001254	3.66E-04	Burkholder iales_uncla	0	6.00E-05
0	0	3.40E-05	0	0.001178	0	0.001022	6.40E-05	0	0	Shuttlewort hia	5.50E-05	0
0	0	0	0	0	0	0	5.00E-05	0	0	Selenomon adaceae_un	0	0
0	0	0	0	0	7.50E-05	0	0	0	0	Christensen ellaceae_R	0	0
0	3.79E-04	0	0	0	0	0	0	0	0	Gracilibact eria	0	0
0	0	0	0	0	0	0	0	0	0	Helicobact er	0	0
0	0	0	0	0	0	0	0	0	0	Prevotellac eae_NK3B	0	0
0	0	0	0	0	0	0	0	0	0	Acholeplas ma	0	0
4.30E-05	0.003144	0	6.10E-05	3.90E-05	0.002111	4.40E-04	0.001659	0	0	Xanthomon adaceae_un	5.10E-05	0
0	0	0	0	0	5.30E-05	0	8.60E-05	0	0	Phascolarct obacterium	0	0
4.80E-05	0.001982	4.70E-05	0	4.10E-05	0.001569	4.63E-04	0.001239	1.90E-05	0	Rhizobiace ae_unclassi	0	0
0	8.50E-05	0	0	0	1.62E-04	2.10E-05	1.78E-04	0	0	Serratia	0	0
0	1.11E-04	6.60E-04	5.90E-05	0	6.05E-04	3.31E-04	0.011878	0	0.001639	Chloroplast	4.90E-05	0
0	0	0	0	0	0	0	0	0	0	Mucispirill um	0	0
5.21E-04	0	0	0	0	0	0	0	0	0	Propioniba cterium	0	0
0.006221	0.005653	0.009755	0.010114	0.012794	0.021442	0.008284	0.005656	0.001064	0.00278	Others	0.004339	0.008355

SC27	SC22	SC26	SC28	SC03	SC04	SC17	SC25	SC23	SC09	SC08	SC07	SC20
0.52656	0.491189	0.453739	0.428353	0.375188	0.3624	0.262834	0.255013	0.239628	0.234224	0.190914	0.190025	0.149065
0.031555	0.018676	0.04892	0.018378	0.122298	0.111978	0.013247	0.037949	0.045824	0.164496	0.035944	0.199306	0.191421
0.012284	0.026308	0.078229	0.071241	0.084145	0.071231	0.112444	0.050638	0.14453	0.102306	0.081513	0.01473	0.081298
0.038775	0.077474	0.10633	0.059168	0.110395	0.120292	0.021646	0.026666	0.039168	0.105311	0.097827	0.041131	0.040006
0.036634	0.210686	0.169548	0.018846	0.025023	0.089963	0.237995	0.158849	0.147541	0.110002	0.038767	0.248728	0.225839
9.90E-05	0.01829	0.029475	0.017013	0.033169	0.0182	0.028368	0.054647	0.019166	0.002948	0.07646	0.074532	0.016237
0.043168	0.003478	4.41E-04	0.021413	0.0022	0.006141	0.006161	0.005736	0.006304	0.032616	0.00157	0.001615	0.008306
0.007667	0.008823	0.005638	0.047552	0.048374	1.75E-04	0.038663	0.017791	0.037299	0.042462	0.075622	0.010368	0.011826
0.018649	0.014213	0.015076	0.012564	0.031042	0.016509	0.003322	0.011429	0.047336	0.018925	0.035728	0.052416	0.050965
0.012043	0.035751	0.00639	0.046375	0.014181	0.030869	0.046456	0.024648	0.003518	0.030119	0.031121	2.82E-04	0.006669
0.005738	0.018038	0.00138	0.001039	0.052309	0.02731	0.002818	0.006035	0.001514	0.013273	0.105786	0.030823	0.005399
0.010794	0.002112	0.004605	0.027989	0.003696	0.001177	0.004308	0.025966	0.161246	0.003082	0.022154	0.046262	0.02726
0.031531	0.010583	0.02567	0.018348	0.005728	0.003998	0.00605	0.016581	0.016391	0.004969	0.031111	0.014361	0.083214
0.022721	0.011878	0.006523	0.053813	0.007222	0.019232	0.005914	0.010181	0.002693	0.002283	0.004025	0.021236	0.007527
0.008586	0.017552	8.65E-04	0.01533	0.002979	0.015988	0.032336	0.022267	1.79E-04	0.005933	0.00572	1.31E-04	0.001361
0.001949	0.003312	0.007833	0.028352	0.004688	0.018028	0.003837	9.07E-04	3.84E-04	0.011	0.021129	0.011875	0.003405
0.006403	8.85E-04	0.00513	0.004535	0.002736	0.008079	0.005463	0.025503	0.008072	0.003442	6.14E-04	9.48E-04	0.005347
4.01E-04	0.001431	6.21E-04	6.20E-05	3.70E-04	5.50E-04	4.90E-05	0.031475	0	0.02609	0.018897	0.002659	5.37E-04
0	0	0	0	0	0	0	0	0	0	0	0	0
0	3.60E-05	0	0	0.002437	0	6.10E-05	0	0	0	0	0	0
0	6.50E-05	1.55E-04	0	4.17E-04	5.10E-05	9.80E-04	1.07E-04	7.00E-05	6.48E-04	0.016977	0	0.001019
8.63E-04	2.90E-05	0	0	2.17E-04	3.00E-05	0.004554	0.003138	1.23E-04	0.002444	0.003023	7.80E-05	0.004102
0.032253	0.00116	7.19E-04	0.011417	0.004882	0.002737	0.001963	0.003867	0.004832	0.005301	0.002191	9.36E-04	0.003251
0.005869	0.003929	0	1.38E-04	0.006455	0.003182	0.002032	0.001714	0.035004	0.004137	0.019352	0.012766	0.006842
0.004998	0.001579	0.004756	0.001539	0.003814	0.004983	0.034123	0.011493	2.82E-04	0.010542	0.001613	1.17E-04	0.004365
3.20E-05	0	0	9.50E-05	3.10E-05	0	2.00E-05	9.50E-05	6.80E-05	3.40E-05	3.14E-04	7.40E-05	0
0.020136	0.006318	0.011421	0.035347	0.002513	0.006214	0.002697	0.004488	0.011282	0.005826	0.01956	0.006038	0.003279
0.006099	1.41E-04	0.001641	0.002865	0.009558	0.005255	0.001077	0.007408	0.001313	0.001587	4.80E-05	0	0.003739
0.00446	5.68E-04	1.98E-04	3.71E-04	8.96E-04	0.012732	0.001669	0.001375	0	0.002655	0.012851	0	0.00144
0.005506	3.31E-04	1.55E-04	0.001041	0.002267	5.30E-04	0.022791	0.026912	7.90E-05	0.001965	9.15E-04	0	0.011551
0	0	0	0	0	0	0	1.51E-04	0	7.00E-05	2.90E-05	0	0
0.005805	0	9.72E-04	0	0.00238	0.004439	0.002002	0.014145	0	0.010608	0	3.70E-05	0.002943
0.04854	0.007036	0.002477	0.026112	0.01625	0.016496	0.006044	3.35E-04	0.012795	0.011909	0.003214	0.001629	0.003593
0.004663	0.001302	0.001012	0.004605	7.30E-04	0.008718	0.00507	0.013197	4.52E-04	0.004867	0.008272	0	6.68E-04
0	1.90E-04	8.70E-05	5.40E-04	0.001807	0	3.15E-04	0.001503	7.75E-04	3.01E-04	0.007821	5.49E-04	0.001128
0	0	0	0	0	0	0	0	0	0	0	0	0
9.80E-05	0.001016	2.21E-04	1.70E-04	0.001102	2.51E-04	0.024408	0.011306	5.65E-04	4.22E-04	3.69E-04	3.60E-05	0.002372
0.003041	0	0	0	5.90E-04	1.29E-04	5.60E-05	4.38E-04	0	1.33E-04	0.003678	0	5.43E-04
0.008868	0	7.25E-04	6.51E-04	2.64E-04	1.71E-04	5.28E-04	0.039406	6.08E-04	6.16E-04	1.20E-04	1.17E-04	0.002172
0	0	0	2.80E-05	0	0	0	2.30E-05	0	0	0	4.20E-05	0
1.39E-04	2.28E-04	1.88E-04	0.001935	6.40E-05	1.87E-04	1.70E-05	9.00E-05	1.13E-04	0	1.39E-04	3.05E-04	4.40E-05
8.07E-04	6.97E-04	6.25E-04	3.40E-05	0.001098	4.37E-04	2.74E-04	3.82E-04	6.63E-04	5.13E-04	3.95E-04	0.005965	0.001427
5.80E-05	3.80E-05	1.65E-04	8.00E-05	0	7.30E-05	0.002673	0.001152	4.10E-05	2.35E-04	5.16E-04	0	8.00E-05
0	2.47E-04	1.44E-04	0.001978	8.44E-04	5.39E-04	0.003636	9.60E-05	2.01E-04	7.31E-04	0.003225	0	0.003062
0	1.43E-04	0	1.99E-04	0.001175	1.65E-04	3.64E-04	0.001324	1.23E-04	3.75E-04	5.47E-04	0	0.004019
2.15E-04	7.50E-04	6.56E-04	6.27E-04	1.86E-04	0	2.44E-04	1.17E-04	4.29E-04	0.006219	0.002339	0.003842	0.002048
0	0	0	0	0	0	0	1.27E-04	0	6.90E-05	2.80E-05	1.18E-04	0
3.16E-04	3.37E-04	2.90E-04	0.002297	2.79E-04	1.34E-04	6.00E-05	0.001727	4.00E-05	0	5.70E-05	0	0.004742
0	0	9.37E-04	0	7.50E-05	2.39E-04	6.69E-04	0	4.40E-05	1.43E-04	0	1.31E-04	2.43E-04
0	0	0	0.002406	8.61E-04	6.30E-05	2.15E-04	0	0	1.95E-04	8.64E-04	0.002696	1.18E-04
0	3.80E-05	5.80E-05	0.002668	7.76E-04	1.97E-04	5.35E-04	0.003277	0	2.99E-04	8.88E-04	0	6.57E-04
1.53E-04	6.20E-05	2.55E-04	1.03E-04	3.87E-04	3.24E-04	0.014065	0.009667	0	8.30E-05	0.002458	0	1.69E-04
0	0	0	0	0	0	0	0	0	0	0	0	0
0	0	0	0	0	0	0	0	0	0	0	0	0
1.31E-04	9.00E-05	3.29E-04	2.13E-04	7.30E-05	1.00E-04	4.46E-04	0.006557	0	8.20E-05	0.001163	3.50E-05	0
0	0	0	0	0	0	0	5.10E-05	0	3.20E-05	0	0	0

0	2.90E-05	0	4.88E-04	4.10E-05	4.70E-05	0.002655	0.009923	1.87E-04	3.70E-05	1.81E-04	8.50E-05	0.003991
0	0	0	0	0	0	0	0	0	0	0	3.50E-05	0
0	0	0	3.40E-05	0	3.30E-05	8.11E-04	7.20E-05	1.21E-04	3.00E-05	5.10E-05	3.40E-05	0
0	0	9.40E-05	0	7.60E-05	3.20E-05	0	0	0	0	0	0	4.35E-04
0	0	0	0	0	3.00E-05	0	3.70E-05	0	0	6.06E-04	0	0
1.00E-04	0	0	1.04E-04	0	0	1.45E-04	1.90E-04	0	0	0	0	0
0	0	0	0	0	0.002513	0	0	0	9.47E-04	0	0	0
0.018914	0	0	0.001589	0	0	6.80E-05	1.19E-04	0.003471	0	0	1.13E-04	2.47E-04
0	1.31E-04	3.73E-04	3.07E-04	0.001973	7.64E-04	2.35E-04	5.50E-05	7.36E-04	2.70E-04	0.001516	2.45E-04	5.05E-04
0	2.00E-04	1.96E-04	5.87E-04	3.38E-04	4.23E-04	9.06E-04	1.31E-04	2.55E-04	4.44E-04	3.26E-04	0	2.07E-04
0	0	0	0	0	0	0	0	0	2.70E-05	0	0	0
0	0	4.80E-05	0	0	7.60E-05	0	0	0	0	2.10E-05	0	0
2.94E-04	2.00E-04	4.34E-04	1.48E-04	9.55E-04	5.13E-04	9.03E-04	0.002875	0	0.002034	2.60E-05	2.47E-04	3.27E-04
0	7.50E-05	2.70E-05	3.50E-05	3.70E-05	1.02E-04	0	0.002505	0	0.002082	0.001655	2.85E-04	1.56E-04
0	0	0	0	0	0	0	0	0	0	0	0	0
3.30E-05	0	4.42E-04	7.40E-05	7.09E-04	1.25E-04	9.00E-05	0	2.03E-04	3.00E-05	9.60E-05	3.90E-05	0.001203
0	0	1.00E-04	4.89E-04	3.30E-05	6.50E-05	0.009224	4.35E-04	1.04E-04	0	7.20E-05	7.70E-05	0
0	0	0	0	0	0	6.54E-04	3.70E-05	0	0	3.60E-05	0	0
0	0	0	0	0	0	0	9.00E-06	0	0	1.31E-04	0	0
0	0	0	0	4.22E-04	0	9.90E-05	8.00E-05	0	0	1.04E-04	0	0
0	0	0	0	0	0	0	0	0	0	0	0	0
0	0	0	0	0	0	0	0	0	0	0	0	0
0	0	0	0	0	0	0	0	0	0	0	0	0
0	9.40E-05	2.30E-05	0	7.10E-05	1.05E-04	0	0.00113	0	0.001245	4.92E-04	2.42E-04	1.19E-04
0	4.00E-05	0	0	0	0	0	0	0	0	0	0	0
3.20E-05	3.30E-05	9.90E-05	0	3.40E-05	9.60E-05	1.10E-05	0.001313	0	0.00129	5.72E-04	1.72E-04	4.20E-05
0	0	0	0	0	0	0	6.70E-05	0	1.00E-04	5.30E-05	4.20E-05	0
0	0	0	0	0	2.70E-05	0	4.30E-05	0	0	0	0	0
0	0	0	0	0	0	0	0	0	0	0	0	0
0	0	1.07E-04	0	0	8.60E-05	5.10E-05	0	0	0	0	0	0
0.01202	0.002189	0.003458	0.008315	0.00714	0.004467	0.018649	0.03303	0.004228	0.004942	0.006194	0.001445	0.00747

SC11	SC13	SC32	SC15	SC19	SC35	SC10	SC02	SC16	SC12	SC18	SC21	SC31
0.144364	0.13871	0.107074	0.095059	0.088586	0.08042	0.077033	0.065693	0.061953	0.041175	0.03776	0.037082	0.035628
0.050998	0.044042	0.200395	0.309121	0.006927	0.023899	1.28E-04	0.0779	3.99E-04	0.671493	0.162622	0.04662	0.174148
0.108209	0.086196	0.026623	0.116981	0.006129	0.325152	0.365921	0.016786	0.656487	0.030572	0.355859	0.662632	0.361896
0.11977	0.098438	0.076596	0.043149	0.007995	0.049572	0.118953	0.096809	0.0836	0.067185	0.054657	0.067226	0.121885
0.16401	0.251394	0.365423	0.105593	0.378733	0.096955	0.181649	0.387117	1.44E-04	0.005834	0.110637	0.001041	0.106153
0.079204	0.159439	0.016952	0.008736	0.022937	3.92E-04	0.018572	0.199056	8.50E-05	1.15E-04	0.01763	4.51E-04	1.03E-04
0.001748	0.004806	3.60E-04	0.00108	0.056743	6.05E-04	0.001328	2.86E-04	3.00E-04	0.001012	0.001722	0.001643	3.60E-05
0.0125	0.014866	0.01153	0.091165	0.069067	0.004658	0.011229	0.010029	6.10E-05	0.004471	0.014534	0.016822	0.008055
0.016634	0.026631	0.01961	0.019415	0.010734	0.001043	9.40E-05	0.049501	9.30E-05	0.012723	0.021463	0.01825	0
0.033729	0.010042	0.012085	0.009327	0.016496	0.020567	0.019555	0.00166	0.043822	0.009715	0.01704	0.037225	0.013011
0.068579	0.025919	0.01187	0.034735	6.04E-04	0.13391	0.009493	0.010927	0.095015	0.011302	0.008088	0.044622	0.028573
0.022834	0.006355	0.021906	0.036224	0.195434	0.081078	0.011895	0.00124	1.46E-04	0.097239	0.014327	0.002627	0.034925
0.028113	0.034645	0.011568	0.012636	0.012608	0.015156	0.058069	0.014115	6.90E-05	6.60E-04	0.053688	0.020141	0
0.007748	0.02022	0.013914	0.006282	0.01859	0.023695	0.00571	0.012512	0.021127	0.01089	0.001914	2.41E-04	0.00882
0.001597	0.001959	0.006314	0.010908	0.010132	7.15E-04	0.02941	1.31E-04	3.60E-05	0.001589	0.002626	0	0
0.031985	0.002688	0.001623	0.015413	1.30E-04	0.015089	0.011021	5.10E-05	0.004622	0.007803	0.011054	0.008635	0.035908
0.009744	0.005955	0.005733	0.004177	0.009655	5.04E-04	0.001034	0.001688	0.001672	0.001763	0.001794	1.37E-04	2.13E-04
0.022132	0.003672	0.025723	2.64E-04	9.37E-04	0.034283	0.008961	0.008046	0.001547	0	0.015016	3.26E-04	0.052375
0	0	0	0	0	0	0	0	0	0	0	3.60E-05	0
0	0	1.92E-04	0	0	0	0	0	0	4.80E-05	9.99E-04	0	0
1.50E-04	2.64E-04	1.38E-04	0.004194	0.001092	0.007017	0.015663	6.00E-05	0	6.58E-04	0.029205	0.002875	0
5.32E-04	0	1.99E-04	0.003828	0.004031	0	9.51E-04	4.40E-05	0	8.68E-04	3.66E-04	0	0
0.001876	0.006333	0.002275	0.011272	0.002059	3.69E-04	0.003549	0.001003	3.60E-05	0.007673	0.001341	6.10E-04	0
0.002925	0.009447	0.013439	0.006285	6.90E-05	0.043969	0.004847	3.40E-05	0	0.001756	0.001648	0	5.98E-04
0.002181	0.002035	0.001227	0.009167	0.008975	1.82E-04	0	1.15E-04	0	0.002328	0.002455	0	4.50E-05
1.41E-04	0	1.51E-04	6.40E-05	5.10E-05	9.57E-04	3.90E-05	0	3.60E-05	0	2.40E-04	3.20E-05	2.30E-05
0.005637	0.009888	0.010048	0.002091	0.007166	0.001341	0.002105	0.00906	1.34E-04	0.001775	9.97E-04	0	2.04E-04
2.35E-04	0.007162	0.002568	0.004277	3.71E-04	1.17E-04	0.009712	1.99E-04	2.50E-05	9.79E-04	0.001591	1.23E-04	0
0.001932	1.59E-04	0.001399	9.40E-04	0	0.001091	6.86E-04	0	0.004869	0	0.003054	0	0.00249
0.001729	0.001501	0.00214	0.001373	0.001281	7.50E-05	0.003405	3.69E-04	0	2.32E-04	0.001876	0	0
1.49E-04	2.90E-05	1.60E-05	0	2.40E-05	1.79E-04	0	0	0	0	3.10E-05	0	2.54E-04
0	0.00888	0.001382	0.005527	0.003316	0	0	0	0	0.001287	0.003832	0	0
7.67E-04	2.79E-04	0.001602	0.004219	8.47E-04	4.70E-04	0.003298	0.006705	6.80E-05	0.001384	0.001904	0	2.60E-04
0.002986	3.88E-04	0.002061	0.001148	2.50E-05	6.20E-05	0.009839	0	0.003692	7.48E-04	0.001965	3.00E-04	0.00201
0.003334	7.78E-04	8.30E-05	1.41E-04	2.70E-05	1.60E-05	0	2.72E-04	0	0	0.001939	4.22E-04	0
0	0	0	0	0	0	0	0	0	0	0	0	0
2.76E-04	4.22E-04	8.66E-04	7.76E-04	0.00725	0.004213	0	7.10E-05	0	0	0.005659	0	3.50E-04
0.017368	1.09E-04	0	4.11E-04	2.22E-04	0.010682	0	2.40E-05	0	0	0.00108	2.86E-04	2.10E-05
0.001393	0.00138	0.001406	6.02E-04	0.0116	6.50E-05	1.04E-04	2.31E-04	0	5.70E-05	0.003001	0	0
0	0	0	0	0	0	0	0	3.20E-05	0	6.00E-05	0	1.01E-04
3.35E-04	1.16E-04	8.60E-05	0	0.001544	1.31E-04	9.80E-05	1.32E-04	1.40E-05	0	4.70E-05	0	0
6.88E-04	0.001811	2.64E-04	6.25E-04	0.001481	1.20E-05	2.45E-04	0.019509	0	2.31E-04	0.00222	4.01E-04	0
4.67E-04	2.60E-04	7.33E-04	0.006567	2.11E-04	0	0	0	0	0	5.70E-04	0	2.60E-05
6.69E-04	1.13E-04	2.80E-05	0.001754	5.70E-05	3.26E-04	0.001391	0	0.00472	0	0.001124	0.0088	0
1.70E-04	0.001265	4.42E-04	0	1.63E-04	8.70E-05	0	2.30E-05	4.40E-05	0	0.001384	0	0
6.55E-04	4.26E-04	0.005245	4.78E-04	0.001779	0.00126	0.000649	7.31E-04	6.80E-05	1.17E-04	0.004428	0.00404	5.48E-04
4.20E-05	0	5.10E-05	0	0	1.14E-04	0	0	3.60E-05	0	3.10E-05	0	2.22E-04
3.48E-04	4.76E-04	9.29E-04	0	0.001962	2.60E-04	0.001679	2.35E-04	0	0	2.14E-04	2.80E-05	0
0.005856	0.003227	6.60E-05	1.49E-04	4.30E-04	2.11E-04	0	0.001267	0	0	7.00E-05	5.30E-05	2.10E-05
8.50E-04	3.30E-05	3.00E-05	6.80E-05	0	0.003695	0	0	3.00E-05	6.70E-05	0.002853	1.79E-04	2.80E-05
2.02E-04	5.90E-05	7.03E-04	2.03E-04	9.50E-05	0	0	0	0	1.19E-04	0.001912	0	0
9.42E-04	3.36E-04	4.62E-04	0	6.65E-04	5.11E-04	0	4.70E-05	0	0	4.19E-04	0	0
0	0	0	0	0	0	0	0	0	0	0	0	3.50E-05
0	0	1.60E-05	0	0	0	0	0	0	0	0	0	0
0.002676	1.55E-04	4.23E-04	1.96E-04	5.07E-04	4.78E-04	0	1.70E-05	0	0	7.39E-04	3.39E-04	0
1.80E-05	3.30E-05	4.30E-05	0	0	1.05E-04	0	2.50E-05	0	0	6.40E-05	0	2.10E-05

4.56E-04	1.23E-04	1.67E-04	0	5.28E-04	2.07E-04	0	1.36E-04	0	0	2.56E-04	0	3.10E-05
2.30E-05	2.40E-05	2.90E-05	0	0	3.50E-05	0	0	0	0	0	0	2.50E-05
3.30E-05	0	7.40E-05	0	0	0	0	0	6.30E-05	0	0	0	0
1.70E-05	7.49E-04	0	1.06E-04	0	0	0	5.63E-04	0	0	4.85E-04	7.41E-04	0
4.85E-04	0	0	0	0	0	0	0	0	0	2.70E-05	0	0.001455
0	0	1.60E-05	0	0	0	0	0	3.00E-05	0	0	0	7.60E-05
0	0	0	0	0	0	0	0	0	0	0.00353	0	0
2.80E-05	0	6.40E-05	0.00093	0.004807	1.80E-05	0	2.00E-05	0	0	0	0	0
0.001093	6.78E-04	1.30E-04	0.001756	3.20E-05	0.003498	5.35E-04	7.10E-05	0.008928	5.29E-04	2.56E-04	0.006802	0
1.20E-04	9.40E-05	3.90E-05	4.69E-04	0	6.42E-04	9.96E-04	0	0.002425	6.50E-05	2.44E-04	0.003139	0
0	0	1.95E-04	0	0	3.20E-05	0	0	0	0	0	0	0
1.80E-04	0	2.20E-05	0	0	0	0	1.40E-05	3.00E-05	0	0	0	0
2.00E-05	3.03E-04	4.50E-05	0.001435	0.012817	2.10E-05	9.41E-04	1.90E-05	0	1.26E-04	1.07E-04	0	1.55E-04
0.001793	3.35E-04	0.002133	7.00E-05	9.30E-05	0.00214	0.001008	9.78E-04	1.30E-04	0	0.001069	0	0.003804
0	0	0	0	0	0	0	0	0	0	0	0	0
2.75E-04	1.85E-04	1.96E-04	1.78E-04	0	0	0	7.00E-05	0	1.09E-04	0.002265	5.35E-04	2.30E-05
7.70E-05	1.38E-04	4.22E-04	0	2.40E-05	1.09E-04	0	2.90E-05	0	0	5.40E-05	0	0
0	0	0	0	0	1.90E-05	0	0	0	0	0.001022	0	0
0	0	1.21E-04	0	8.80E-05	0	0	0	0	0	0	0	0
5.73E-04	0	0	0	6.00E-05	0	0	0	0	0	0	0	0
0	0	0	0	0	0	0	0	0	0	0	0	0
0	0	0	0	0	0	0	0	0	0	0	0	0
0	0	0	0	0	0	0	0	0	0	0	0	0
8.48E-04	1.76E-04	0.001255	6.90E-05	5.30E-05	0.001642	4.44E-04	3.43E-04	0	0	5.74E-04	8.60E-05	0.001886
0	0	0	0	0	0	0	0	0	0	6.30E-05	0	0
0.001219	3.14E-04	0.001104	0	7.10E-05	0.00159	4.74E-04	4.43E-04	1.58E-04	5.00E-05	8.18E-04	0	0.001506
3.80E-05	0	4.80E-05	0	0	8.80E-05	3.90E-05	2.90E-05	0	0	2.80E-05	0	3.80E-05
0.00102	0	4.90E-05	0	3.50E-05	2.20E-05	3.40E-05	0	0	0	0	0	4.22E-04
0	0	0	0	0	0	0	0	0	0	0	0	0
3.71E-04	0	0	0	0	0	0	0	0	0	0	0	0
0.009904	0.00354	0.007879	0.008367	0.011655	0.004249	0.007214	0.003565	0.003254	0.003253	0.007453	0.004452	0.001593

SC34	SC24	SC05	SC36	SC06	SC14	SC30	SC33	ID...112	H042	H008	H130	H036
0.032309	0.030666	0.029585	0.028504	0.027675	0.021897	0.018509	0.003443	Prevotella	0.473924	0.422388	0.404445	0.387203
0.001338	0.235355	0.830866	0.036111	0.085712	0.153867	0.220936	0.263213	Neisseria	0.00924	0.025544	0.009113	0.063338
0.104497	0.023755	0.015885	0.032512	0.038885	0.284124	0.301783	0.3916	Streptococcus	0.040448	0.059484	0.103768	0.032294
0.016212	0.028308	0.001509	0.216036	0.062988	0.076083	0.0569	0.011604	Veillonella	0.154125	0.10381	0.171179	0.123626
0.125058	0.081217	0.038751	0.141456	0.06187	0.053533	0.064643	0.02975	Leptotrichia	0.026298	0.069583	0.051983	0.037326
0.003844	0.034746	0.001787	0.028786	0.007979	0.017394	0.020474	0.004246	Fusobacterium	0.018866	0.018081	0.012966	0.094612
3.43E-04	0.005998	0.001321	0.064742	0.014602	0.001567	2.57E-04	8.60E-05	Alloprevotella	0.065989	0.086973	0.044196	0.049221
0.046257	0.050077	0.016314	0.088722	0.096924	0.02666	0.024188	0.037451	Porphyromonas	0.004249	0.004692	0.01036	0.024621
2.92E-04	0.152385	0.008273	0.104502	0.03826	0.059614	0.022764	0.007638	Haemophilus	0.010931	0.011254	0.009696	0.026878
0.064076	0.0047	1.09E-04	0.00299	0.005374	0.030748	0.007912	0.008048	Actinomyces	0.040584	0.020976	0.023147	0.022681
0.020882	0.047574	0.00105	0.008022	0.001216	0.016825	0.013943	0.093106	Rothia	0.004809	0.00236	0.004368	0.009072
0.050379	0.045293	0.031527	0.026085	0.076991	0.041509	0.032544	0.033136	Capnocytophaga	8.79E-04	3.29E-04	0.00217	0.005867
0.006534	0.003569	0.006232	0.015676	0.005691	0.009449	0.073992	0.001623	Gemella	3.04E-04	0.001985	0.002946	0.005911
0.111551	0.015443	0.001075	0.031954	0.002218	0.003438	0.00546	0.004084	Lachnoanaerobaculum	0.017485	0.00914	0.006162	0.020378
0.082919	0.020483	3.80E-05	0.003521	0.001452	6.61E-04	0.001667	3.78E-04	TM7x	0.007864	0.020591	0.021673	0.004299
0.015113	0.003741	1.02E-04	2.22E-04	0.008855	0.004645	0.006658	0.032762	Granulicatella	0.001543	0.002152	0.007367	0.004121
2.50E-05	0.00171	2.06E-04	0.001824	0.001662	0.004774	0.001504	1.14E-04	Campylobacter	0.015428	0.01277	0.003309	0.005488
0.009356	0	3.60E-05	4.66E-04	0.37646	0.036032	0.006454	0.004582	Halomonas	3.10E-05	2.20E-05	0	0
0	1.41E-04	0	0	0	3.40E-05	0	0	Moraxella	0	0	0	0
3.10E-05	0	0	4.90E-05	0.006272	0.01511	0	0	Actinobacillus	7.00E-05	0.002899	5.73E-04	1.92E-04
8.45E-04	0.141417	0	0	8.28E-04	0.056613	0.013923	0.052481	Lautropia	6.85E-04	8.34E-04	0.001905	0.001976
0.018565	3.79E-04	0	0.001099	0	8.90E-05	0.001016	0	g__Absconditabacteria	8.96E-04	3.09E-04	3.70E-05	0.007349
0.05791	0.004566	3.80E-05	0.004397	0.002769	0.002389	0.003204	1.12E-04	Oribacterium	0.004041	0.00752	0.005647	0.006554
0.019696	7.00E-04	0.008018	0.105845	0.001423	0.003162	0.005136	1.53E-04	Peptostreptococcus	6.37E-04	8.19E-04	5.07E-04	0.006045
0.004196	0.001727	1.36E-04	0.001296	5.82E-04	0.021929	0.007727	0	Selenomonas	0.010033	0.003993	0.012073	2.22E-04
1.04E-04	1.80E-05	8.20E-05	9.90E-05	1.25E-04	3.08E-04	1.53E-04	3.30E-05	Bacteroides	7.00E-05	4.54E-04	5.00E-05	0
0.018328	0.002179	0.001205	0.020302	6.06E-04	1.47E-04	4.65E-04	3.11E-04	Solobacterium	0.002111	0.011241	0.004928	0.004798
0.011926	1.75E-04	0	1.47E-04	8.80E-05	2.34E-04	0.001008	0	Stomatobaculum	0.007857	0.007188	0.00363	0.010584
4.06E-04	2.50E-05	0	0	5.40E-05	4.50E-05	0	0	Megasphaera	0.008996	0.03357	0.011823	0.00944
0.035004	0.012149	0	0.001035	2.91E-04	2.83E-04	0.002495	1.27E-04	Clostridia_UCG-014	0.006731	0.005089	0.001122	0.009342
0	0	0	0	1.97E-04	0	0	0	Pseudomonas	4.01E-04	8.82E-04	5.82E-04	6.60E-05
0.016052	0.002348	3.10E-05	2.05E-04	1.00E-05	7.90E-05	7.80E-05	0	Lachnospiraceae_uncu	0.029929	0.013657	0.009953	1.72E-04
0.008205	6.82E-04	1.49E-04	9.30E-05	0.001027	2.52E-04	0.00194	8.10E-05	[Eubacterium]_nodatu	0.002055	0.002725	0.001122	0.004193
0.007071	1.08E-04	1.82E-04	4.83E-04	1.99E-04	3.92E-04	6.95E-04	1.98E-04	Atopobium	0.009837	0.020716	0.006575	0.001815
0	0.001508	0	1.03E-04	5.70E-05	7.18E-04	7.07E-04	0	Aggregatibacter	0.001175	3.83E-04	0.007425	0.002809
0	0	0	0	0	0	0	0	Muribaculaceae	6.30E-05	0	0	5.60E-05
0.044136	1.00E-04	0	0	4.30E-05	0.003375	0.0055	5.48E-04	Tannerella	3.92E-04	3.72E-04	3.80E-04	0.002316
1.95E-04	0.011011	0	4.50E-05	0	0.004011	0.002516	0	Corynebacterium	3.45E-04	4.22E-04	0.001443	1.82E-04
2.80E-04	8.20E-05	5.10E-05	7.88E-04	1.15E-04	0.001108	0.005163	2.78E-04	Treponema	0.001238	3.19E-04	0.002775	2.42E-04
0	0	4.00E-05	0	2.00E-05	0	0	2.20E-05	Faecalibacterium	9.80E-05	1.76E-04	0	6.70E-05
6.90E-05	2.62E-04	0	1.85E-04	0	7.10E-05	2.99E-04	5.80E-05	Lachnospiraceae_uncl	3.07E-04	6.50E-05	0	5.80E-05
0	0.019566	4.14E-04	4.90E-05	2.92E-04	0.004088	0.006715	1.43E-04	Neisseriaceae_unclassi	1.48E-04	8.40E-05	8.20E-05	3.40E-04
0.001484	2.40E-05	0	1.36E-04	0	0	0.002203	7.40E-05	Saccharimonadales	0.006293	9.20E-04	0.010375	0
0.001281	2.62E-04	0	0	8.40E-05	3.07E-04	1.81E-04	9.65E-04	Lactobacillales_unclas	0	5.55E-04	0.000649	7.46E-04
0.007477	1.93E-04	0	5.21E-04	1.24E-04	9.12E-04	6.41E-04	4.18E-04	Parvimonas	1.53E-04	3.80E-05	0.001091	0.001717
5.18E-04	0.001683	6.70E-05	0.001007	0.00146	7.92E-04	9.66E-04	0.001826	Bergeyella	1.83E-04	7.34E-04	0.001307	4.00E-04
2.20E-05	0	0	0	6.53E-04	5.70E-05	0	3.00E-05	Rhodococcus	0	1.04E-04	0	1.75E-04
0.025414	7.20E-05	0	0	5.60E-05	0.002431	0.001417	0	Johnsonella	1.73E-04	5.20E-05	1.10E-04	1.17E-04
0	0	8.20E-05	4.80E-05	0	9.03E-04	0.005702	8.87E-04	F0332	0.001265	4.10E-05	0.00142	0
2.76E-04	0.001535	0	9.69E-04	1.69E-04	0.002372	8.04E-04	0	Abiotrophia	0	1.60E-05	4.00E-05	1.60E-04
0.0029	0	0	5.10E-05	1.56E-04	0.002469	0.001065	0	Filifactor	5.64E-04	3.70E-05	3.56E-04	0.001003
0	0	0	0.001153	1.10E-05	9.57E-04	8.51E-04	9.10E-05	Dialister	5.00E-04	0.001592	0.001977	1.23E-04
3.20E-05	0	0	0	0	0	0	0	Lachnospiraceae_NK4	0	2.40E-05	0	0
3.60E-05	0	0	0	0	3.90E-05	0	0	Fusicatenibacter	0	1.64E-04	0	1.29E-04
3.91E-04	1.90E-05	4.00E-05	2.03E-04	0	0.00569	0.005298	1.90E-05	Saccharimonadaceae	9.61E-04	3.20E-05	6.45E-04	1.13E-04
3.70E-05	0	0	0	4.54E-04	0	0	1.90E-05	Acinetobacter	9.20E-05	1.25E-04	0	0

0.002789	7.70E-05	0	3.83E-04	0	1.81E-04	9.45E-04	0	F0058	5.20E-05	1.08E-04	1.12E-04	5.40E-05
0	0	0	0	2.45E-04	7.10E-05	0	0	Sphingomonas	0	6.00E-05	0	0
2.90E-05	0	2.12E-04	1.06E-04	2.30E-05	0	0	0	Bifidobacterium	0	7.38E-04	4.20E-05	6.50E-05
0	9.60E-05	2.80E-05	4.50E-05	1.51E-04	0.001829	6.16E-04	0	Kingella	2.69E-04	1.50E-05	8.50E-05	6.50E-05
0	0	0	0	0	0	0	5.10E-05	Lactobacillus	0	0	1.18E-04	0
0	0	0	0	0	0	7.60E-05	0	Roseburia	2.05E-04	4.00E-05	2.21E-04	0
0	0	0	0	0	0	0	0	Leptotrichiaceae_uncu	0	0	0	0.004146
0.001278	0.001536	0.001228	0.015151	7.67E-04	2.83E-04	3.17E-04	0	Amnipila	0	0	0	0
5.68E-04	2.39E-04	7.00E-05	1.78E-04	0	1.83E-04	5.24E-04	0.006183	Bacteria_unclassified	4.80E-05	0	7.30E-05	5.20E-05
0.001202	0	0	8.20E-05	0	1.32E-04	1.81E-04	5.90E-04	Actinobacteria_unclas	7.10E-05	0	1.84E-04	6.00E-05
0	0	0	0	0	0	0	5.36E-04	Megamonas	4.70E-05	4.80E-05	4.10E-05	0
0	0	0	0	0	0.00301	0.00892	1.83E-04	Scardovia	7.60E-05	0	0.005802	0
4.06E-04	3.37E-04	0	3.40E-05	0	0	0	0	Butyrivibrio	5.41E-04	3.87E-04	2.26E-04	1.19E-04
0.00132	0	4.60E-05	5.10E-05	0.022759	0.003094	6.01E-04	2.98E-04	Pelagibacterium	0	0	0	0
0	0	0	0	0	0	0	0	[Eubacterium]_rumina	0	6.40E-05	0	0
0	3.60E-04	4.19E-04	1.49E-04	0	0.001503	0.001752	4.56E-04	Burkholderiales_uncla	0	0	0	0
0	0	2.61E-04	6.02E-04	0	1.14E-04	7.41E-04	2.10E-05	Shuttleworthia	7.16E-04	4.70E-05	1.45E-04	0
0	0	0	0	0	3.24E-04	0	0	Selenomonadaceae_un	1.84E-04	0.002169	0.003906	0
8.77E-04	0	0	0	0	0	0	0	Christensenellaceae_R	0	2.50E-05	0	0
0	0	3.90E-05	4.90E-05	0	3.50E-05	2.31E-04	0	Gracilibacteria	0	0	0	1.14E-04
0	0	0	0	0	0	0	0	Helicobacter	2.00E-05	0	0	0
0	0	0	0	0	0	0	0	Prevotellaceae_NK3B	0	0	0	0
0	0	0	0	0	0	0	0	Acholeplasma	1.76E-04	0	0	0
3.69E-04	0	0	0	0.01631	0.001139	5.93E-04	3.23E-04	Xanthomonadaceae_un	0	0	0	0
0	0	0	1.92E-04	0	0	0	2.60E-05	Phascolarctobacterium	0	4.80E-05	0	0
6.41E-04	0	0	6.20E-05	0.014996	0.001568	5.11E-04	1.82E-04	Rhizobiaceae_unclassi	0	0	0	0
8.30E-05	0	0	0	0.001588	1.45E-04	1.15E-04	2.00E-05	Serratia	3.10E-05	2.10E-05	0	6.10E-05
9.40E-05	0	0	0	0	0	0	1.69E-04	Chloroplast	0	0	0	0
0	0	0	0	0	0	0	0	Mucispirillum	2.70E-05	0	0	0
0	0	0	0	0	5.80E-05	0	0	Propionibacterium	0	0	3.41E-04	0
0.016472	0.009404	0.002496	0.010477	0.010182	0.012145	0.025421	0.005224	Others	0.006241	0.005945	0.009254	0.004827

H038	H016	H019	H079	H041	H073	H056	H100	H136	H103	H094	H138	H114
0.381254	0.37871	0.371931	0.367292	0.362583	0.359749	0.358848	0.327605	0.313487	0.307879	0.307194	0.306685	0.297949
0.086868	0.041195	0.010986	0.012101	0.008348	0.014759	6.95E-04	0.017383	0.1009	0.083826	0.163439	0.209425	0.155366
0.028857	0.025617	0.049806	0.035346	0.010369	0.109846	0.103215	0.093588	0.077425	0.028633	0.022863	0.033937	0.030028
0.088481	0.145898	0.132626	0.181333	0.150754	0.106322	0.18089	0.126314	0.211845	0.09863	0.08995	0.086863	0.091082
0.088805	0.137832	0.042117	0.050242	0.148423	0.045557	0.008228	0.05456	0.034807	0.036201	0.075802	0.054586	0.017178
0.037873	0.034746	0.061182	0.055416	0.011816	0.019763	0.02186	0.023577	0.027831	0.110731	0.03291	0.020757	0.084118
0.079137	0.041363	0.125171	0.069281	0.036424	0.080918	0.075471	0.105118	0.038024	0.026886	0.067676	0.048897	0.048871
0.015777	0.00613	0.005587	0.011873	0.001781	0.016111	0.033495	0.002131	0.004022	0.063907	0.024183	0.005246	0.020862
0.00808	0.005598	0.015031	0.009125	0.003437	0.032374	0.004495	0.042874	0.013309	0.013908	0.020416	0.039398	0.054831
0.038099	0.012231	0.024279	0.020102	0.0393	0.033621	0.020735	0.024015	0.022745	0.01935	0.026813	0.037687	0.044749
0.006847	0.006034	0.00924	0.009048	0.00173	0.008944	0.018313	0.004514	0.01302	0.01028	0.014221	0.011676	0.022285
0.002721	0.002209	0.0031	5.41E-04	4.45E-04	0.004728	0.001802	8.99E-04	0.00837	0.01331	0.003717	0.002499	0.01525
0.001371	6.47E-04	0.002486	0.001159	3.15E-04	0.001532	0.005497	0.004267	0.001602	0.004089	0.001215	0.002121	0.002354
0.021322	0.023077	0.014832	0.015843	0.017348	0.011723	0.005282	0.013092	0.006718	0.026185	0.016383	0.013822	0.009894
0.026344	0.017835	0.008596	0.017779	0.042749	0.026644	0.001054	0.046241	0.010679	0.029691	0.025655	0.013352	0.004927
0.00146	0.002005	0.005505	0.004412	0.001343	0.008529	0.009273	0.004564	0.004417	0.007444	0.012687	0.008649	0.003556
0.008378	0.008187	0.023339	0.020756	0.004784	0.010023	0.010729	0.013671	0.017885	0.005473	0.011032	0.00736	0.011828
0	0	0	0	3.60E-05	1.01E-04	1.96E-04	1.35E-04	9.40E-05	1.75E-04	4.50E-05	6.40E-05	4.50E-05
0	0	0	0	0	0	0	0	0	0	0	0	1.80E-05
2.20E-05	3.20E-04	6.32E-04	8.10E-05	2.80E-05	0.001166	0.001324	1.23E-04	2.30E-05	3.53E-04	1.62E-04	0.002267	0.014548
0.001502	1.40E-04	4.02E-04	0	3.11E-04	0.002041	4.37E-04	7.02E-04	0.001361	3.51E-04	2.70E-04	7.02E-04	0.004175
2.80E-05	3.60E-05	2.93E-04	0.016272	9.20E-05	5.40E-05	4.63E-04	0.002762	6.01E-04	0.002105	0.010077	3.84E-04	1.94E-04
0.005015	0.007477	0.008555	0.008132	0.007613	0.009513	0.005872	0.010258	0.019099	0.004594	0.004585	0.004873	0.004636
0.005831	0.004931	0.001535	0.004374	0.001584	0.001632	6.92E-04	0.002106	0.002341	0.004768	0.00204	0.00258	0.002583
0.004562	0.018156	0.004355	0.003941	0.013575	0.00622	0.004339	0.007039	0.01088	0.002999	0.004556	0.002479	0.002185
1.07E-04	3.30E-05	1.13E-04	1.07E-04	6.00E-05	1.98E-04	7.56E-04	0.00109	2.70E-05	1.09E-04	1.13E-04	4.70E-05	1.11E-04
0.004439	0.004211	0.00915	0.003994	0.009416	0.003153	0.006229	0.001783	0.004149	0.003217	0.013071	0.021514	0.002475
0.004506	0.007117	0.012287	0.01262	0.006838	0.005341	0.004918	0.015234	0.003558	0.011182	0.005203	0.010185	0.008021
0.005141	0.007091	0.016337	0.032431	0.045986	0.009477	0.018094	0.008704	0.01439	0.00392	0.010237	0.004527	0.003734
0.003842	9.07E-04	0.001374	0.003104	0.006538	0.006702	0.002952	0.002515	0.003878	0.005588	0.003784	0.003419	0.001672
8.80E-05	2.80E-05	5.42E-04	1.80E-04	6.81E-04	9.63E-04	8.68E-04	0.003573	3.80E-04	5.85E-04	4.81E-04	0.001204	8.27E-04
0.017494	0.035523	0.00703	0.00413	0.017924	0.007708	3.61E-04	0.011541	0.004721	0	0.002951	0.008464	0.001687
0.007111	0.003574	0.003651	0.003363	0.004303	0.003341	8.65E-04	0.003389	0.003454	0.004475	0.002642	0.00584	0.001528
0.002881	0.002037	0.010075	0.006224	0.006766	0.005634	0.025671	0.003037	0.005682	8.31E-04	0.003156	0.00409	0.011092
4.29E-04	9.16E-04	3.70E-04	0.001383	2.00E-04	0.002771	0.001433	4.34E-04	5.15E-04	0.001374	0.005675	0.003901	0.002209
0	2.90E-05	3.80E-05	0	0	0	6.90E-05	2.30E-05	0	4.00E-05	0	0	0
1.58E-04	4.23E-04	0.005534	0.001339	3.04E-04	4.90E-04	0.002464	6.28E-04	6.00E-04	0.007939	5.25E-04	0.001476	0.00127
3.49E-04	1.44E-04	4.83E-04	2.44E-04	3.30E-04	0.00149	6.27E-04	1.29E-04	2.00E-04	0.001989	4.73E-04	0.002369	8.70E-04
6.48E-04	0.001449	6.45E-04	5.45E-04	0	0.006492	0.007453	4.24E-04	5.90E-04	0.006339	5.92E-04	3.18E-04	2.27E-04
0	1.68E-04	1.02E-04	7.20E-05	5.30E-05	4.10E-05	1.79E-04	4.62E-04	0	1.07E-04	0	2.30E-05	5.40E-05
0	8.90E-05	0	0	5.10E-05	1.31E-04	1.11E-04	1.28E-04	0	4.71E-04	3.00E-05	3.40E-05	2.30E-05
3.13E-04	5.00E-05	1.24E-04	7.30E-05	7.20E-05	7.05E-04	6.90E-05	2.40E-05	1.34E-04	0	7.49E-04	2.56E-04	5.96E-04
1.23E-04	0.003439	0.00138	0.003437	6.73E-04	0.002021	0.001727	0.001128	0.006698	0.001341	1.64E-04	0.003849	2.05E-04
0.001058	0	0	1.12E-04	0	4.31E-04	2.02E-04	2.09E-04	7.80E-05	1.57E-04	3.93E-04	1.96E-04	0.001275
7.99E-04	3.99E-04	6.41E-04	9.01E-04	7.20E-05	9.72E-04	0.00117	3.60E-05	8.20E-05	0.004988	2.09E-04	7.38E-04	1.04E-04
2.70E-04	0	1.81E-04	2.09E-04	6.50E-05	2.16E-04	4.50E-05	7.64E-04	7.59E-04	1.73E-04	5.78E-04	6.56E-04	0.001188
6.30E-05	0	4.70E-05	0	0	5.80E-05	9.30E-05	3.90E-04	0	0	0	0	1.20E-04
3.85E-04	9.70E-05	3.10E-05	0	1.01E-04	2.40E-04	1.27E-04	1.75E-04	3.40E-05	5.46E-04	1.20E-04	9.80E-05	9.60E-05
2.50E-05	3.30E-05	2.00E-05	3.05E-04	0	1.84E-04	1.74E-04	2.05E-04	1.27E-04	0.002256	7.20E-05	6.70E-05	0.002004
8.80E-05	2.20E-05	0	2.04E-04	0	0	0	0	0	0	0	1.90E-05	4.72E-04
6.82E-04	8.19E-04	1.74E-04	5.67E-04	0	0.002407	0.001453	5.50E-05	0	0.011242	0.001024	0.001176	6.40E-05
2.07E-04	5.14E-04	6.94E-04	0.001881	0.004418	0.00164	0.003955	2.70E-05	6.60E-05	7.55E-04	3.09E-04	5.85E-04	2.73E-04
3.30E-05	0	0	0	7.50E-05	2.10E-05	2.70E-05	1.38E-04	0	3.40E-05	0	0	1.40E-05
3.80E-05	0	1.60E-05	0	3.62E-04	0	1.51E-04	2.04E-04	0	0	0	2.40E-05	6.00E-05
1.15E-04	1.12E-04	1.90E-05	7.70E-05	1.77E-04	5.91E-04	3.32E-04	1.33E-04	0	0.00295	1.77E-04	2.25E-04	0.006169
0	0	4.40E-05	0	3.00E-05	3.50E-05	1.99E-04	3.33E-04	5.70E-05	0	0	6.60E-05	8.30E-05

1.22E-04	1.47E-04	0	8.80E-05	5.00E-05	3.89E-04	1.78E-04	2.10E-05	2.40E-05	4.86E-04	7.40E-05	2.70E-04	1.45E-04
0	0	3.30E-05	0	0	0	6.20E-05	4.08E-04	0	3.60E-05	0	3.10E-05	3.20E-05
0	0	1.20E-05	0	8.80E-05	2.50E-05	0.001364	1.73E-04	0	0	0	0	0
0	0	1.61E-04	3.60E-05	3.10E-05	4.31E-04	2.38E-04	9.40E-05	6.67E-04	0	0	2.80E-05	0.001322
0	3.60E-05	0	0	0	0	0.00816	0	0	0	3.60E-05	0	3.60E-05
5.60E-05	0	2.50E-05	0	0	2.45E-04	4.20E-05	5.00E-05	0	3.00E-05	6.30E-05	0	0
0	0	0	0	0	0	0	0	0	0	0	0	0
2.82E-04	1.54E-04	4.60E-05	0	0	5.10E-05	0	1.02E-04	3.13E-04	0	0	2.20E-05	0
3.10E-05	5.80E-05	2.30E-05	2.50E-05	0	2.32E-04	0	0	2.57E-04	7.00E-05	1.00E-04	1.41E-04	0
2.28E-04	9.40E-05	0	4.30E-05	6.10E-05	3.02E-04	0	0	1.58E-04	0	1.69E-04	7.30E-05	3.40E-05
2.00E-05	0	7.00E-05	0	3.50E-05	0	0	4.70E-05	0	7.00E-05	0	0	0
0	0	0	6.07E-04	0	0	0.001585	0	0	8.00E-05	0	0	2.20E-05
0.00109	0.001176	2.76E-04	1.36E-04	7.47E-04	0.001147	3.23E-04	1.96E-04	5.95E-04	1.45E-04	2.49E-04	7.39E-04	3.48E-04
0	0	0	0	0	0	0	0	0	0	0	0	0
0	0	0	0	0	0	0	1.00E-04	0	0	0	0	3.50E-05
0	3.70E-05	1.70E-05	0	0	2.30E-05	0	0	1.57E-04	0	2.75E-04	7.50E-05	7.40E-05
4.61E-04	6.90E-05	0	2.45E-04	5.59E-04	0.001678	0.001479	0	3.40E-05	2.11E-04	6.80E-05	4.40E-05	1.31E-04
0	0	0	0	0.018698	0.00212	0.002107	0.001552	1.08E-04	0	6.12E-04	1.10E-04	5.40E-05
0	0	0	0	0	0	6.00E-05	9.20E-05	0	0	0	0	2.50E-05
0	0	0	0	0	0	0	0	0	0	4.11E-04	2.67E-04	4.40E-05
0	0	0	0	0	0	0	0	0	0	0	0	0
0	0	2.30E-05	0	0	0	0	0	0	0	0	0	0
0	0	0	1.44E-04	0	0.001071	8.99E-04	0	0	0	0	0	0
0	0	0	0	0	0	0	0	0	0	0	0	0
0	0	0	0	0	2.90E-05	5.50E-05	2.00E-05	0	0	0	0	0
0	0	0	0	0	0	0	0	0	0	0	0	0
0	0	0	0	7.60E-05	0	0	6.90E-05	9.50E-05	9.80E-05	2.40E-05	1.70E-05	6.80E-05
0	0	0	0	0	0	8.70E-05	0	0	0	0	0	0
0	0	0	0	0	0	0	0	0	0	0	0	0
0	0	0	0	0	0	5.60E-05	0	3.90E-05	0	0	0	0
0.007684	0.008631	0.006626	0.006725	0.008972	0.016934	0.027326	0.012623	0.005889	0.024398	0.0073	0.006508	0.005595

H085	H080	H125	H081	H043	H060	H026	H014	H071	H078	H129	H095	H011
0.292458	0.289924	0.284382	0.282063	0.266184	0.263004	0.262723	0.259213	0.259086	0.257339	0.255344	0.254995	0.249129
0.00113	0.07052	0.00445	0.165193	0.073245	0.058207	0.06233	0.178545	0.047344	0.087203	0.105836	0.114886	0.026566
0.098545	0.105435	0.00602	0.063541	0.049203	0.048198	0.038655	0.014924	0.048214	0.036389	0.072717	0.031437	0.081014
0.151649	0.106495	0.063127	0.046085	0.098593	0.042083	0.063897	0.101264	0.080426	0.080806	0.112677	0.093925	0.114492
0.053986	0.015154	0.065586	0.015295	0.026115	0.035738	0.022107	0.17543	0.108656	0.050412	0.049483	0.035305	0.015786
0.016666	0.027951	0.004458	0.0948	0.097928	0.03298	0.058988	0.01514	0.038795	0.085383	0.069751	0.111274	0.019895
0.015363	0.073431	0.016465	0.037465	0.077957	0.178569	0.177223	0.044509	0.12286	0.09805	0.050406	0.050858	0.098389
0.008448	0.011762	0.001666	0.080618	0.054026	0.020336	0.011288	0.00252	0.035598	0.035676	0.038505	0.048482	0.014733
0.001883	0.077002	0.003843	0.06546	0.054139	0.042844	0.00902	0.010766	0.01331	0.032012	0.018022	0.047163	0.015138
0.069744	0.033545	0.013113	0.009177	0.021915	0.02545	0.017171	0.011812	0.009209	0.017229	0.035532	0.017001	0.0297
0.016006	0.008686	5.12E-04	0.034129	0.006463	0.013828	0.004933	0.020568	0.006868	0.003337	0.013975	0.0056	0.027858
0.00461	0.004064	7.10E-05	0.004634	0.024798	0.005869	0.011035	0.004275	0.003943	0.022156	0.012756	0.005327	0.006129
7.90E-04	0.008856	7.90E-05	0.006603	0.008032	0.001172	0.00434	2.13E-04	0.001522	0.004397	0.004516	0.003982	0.001399
0.009054	0.018885	0.007334	0.005892	0.012492	0.005831	0.007335	0.012746	0.017458	0.01534	0.015145	0.017365	0.001911
0.004527	0.010736	0.018493	0.001326	0.002671	0.003301	0.021643	0.03416	0.011893	0.029048	0.014513	0.01687	4.52E-04
9.04E-04	0.007195	5.95E-04	0.019894	0.017128	0.003954	0.003015	0.00245	0.001183	0.005965	0.004525	0.006607	0.007156
0.011951	0.007228	0.00222	0.004677	0.010136	0.016366	0.039195	0.030296	0.027103	0.010046	0.009636	0.012481	0.012827
1.39E-04	1.11E-04	0	3.72E-04	8.00E-05	5.09E-04	2.70E-05	0	3.00E-05	0	3.50E-05	6.60E-05	3.10E-04
0	0	7.20E-05	0	0	3.40E-05	0	0	2.47E-04	0	0	2.70E-05	3.69E-04
2.97E-04	0.05276	0	0.003532	0	0.002597	0.009738	0	0.01668	7.48E-04	6.43E-04	0.001128	0.006517
0	0.001451	0	0.002756	0.00439	8.61E-04	1.35E-04	9.70E-05	3.18E-04	3.68E-04	0.002094	4.32E-04	0.009793
7.16E-04	6.76E-04	2.21E-04	7.30E-04	0.001066	0.001172	0.011406	0.002031	0.026618	0.04426	0.005833	0.034333	3.18E-04
0.007557	0.004816	0.003812	0.004745	0.005637	0.005625	0.00313	0.007057	0.00591	0.006551	0.005023	0.00922	0.001505
7.66E-04	0.004621	0.001103	0.002929	0.007591	0.004172	0.003571	5.07E-04	0.004693	0.006516	0.004056	0.006236	0.001591
0.054883	0.008887	0.004234	1.98E-04	9.42E-04	0.002099	0.004504	0.019422	0.013254	0.009108	0.003621	0.006101	4.17E-04
1.50E-04	3.40E-05	0.113397	0.002417	1.63E-04	0.01712	0.003811	1.75E-04	0.002956	6.70E-05	2.92E-04	0.002288	0.022531
0.002639	0.00888	0.003949	0.001255	0.01085	0.002534	0.008678	0.004285	0.011833	0.00282	0.005316	0.0094	0.006854
0.024561	0.001263	0.003584	0.005454	0.007435	0.007437	0.003453	0.002333	0.004969	0.001575	0.006163	0.004307	6.07E-04
0.039815	0.006778	0.021133	2.25E-04	1.24E-04	0.007919	0.005329	0.008012	0.01813	0.001883	0.006952	0.001475	0.00691
0.002801	9.54E-04	0.005893	0.001125	8.11E-04	0.001592	0.01767	0.004732	0.004704	0.005181	0.003574	0.002716	0.001078
0.001583	0.001432	0.006077	0.008998	0.002252	0.012946	0.001965	3.70E-05	5.37E-04	1.34E-04	0.001366	3.54E-04	0.003943
6.98E-04	0	0.008797	0.001331	0.00108	0.003403	0.008221	0.0143	0.007955	0.006281	0.010852	0.011519	0
0.001564	0.002617	0.001899	4.77E-04	0.003255	0.001611	0.004218	9.04E-04	0.005933	0.002282	0.004974	0.005248	0.003755
0.010441	0.003219	0.002511	2.16E-04	0.003551	0.001059	0.008631	0.001785	0.003237	0.001738	0.006797	0.003097	0.005749
7.55E-04	0.002549	2.10E-04	0.001811	0.002647	0.006167	1.22E-04	3.20E-04	0.005466	0.00178	0.001652	2.90E-04	0.001189
0	0	0.005258	4.30E-05	2.00E-05	0.001122	0.020133	1.41E-04	0	0	0	2.90E-05	0.001453
0.002096	2.92E-04	1.47E-04	4.16E-04	6.84E-04	0.004968	0.002728	3.26E-04	0.001673	0.001089	0.003268	2.28E-04	0.001494
0.002393	5.48E-04	1.33E-04	0.001456	4.58E-04	7.29E-04	2.82E-04	7.70E-05	4.43E-04	4.00E-05	0.001697	1.89E-04	0.046873
0.003922	0.00314	0	3.59E-04	0.002429	0.006695	6.32E-04	3.90E-05	0.002097	0.002383	0.001939	0.002048	0.001299
1.61E-04	0	0.06355	0.001438	2.15E-04	0.010626	0.00138	1.26E-04	8.19E-04	4.20E-05	8.60E-05	0.001128	0.014614
1.00E-04	3.10E-05	0.015489	3.12E-04	1.36E-04	0.002844	0.008098	9.30E-05	3.11E-04	4.10E-05	1.39E-04	2.63E-04	0.003578
7.90E-05	5.57E-04	0	3.05E-04	5.23E-04	0	9.20E-05	2.29E-04	3.43E-04	0.009882	0.001328	1.42E-04	5.37E-04
2.08E-04	2.90E-05	7.90E-05	0.001817	4.21E-04	4.12E-04	3.35E-04	0.005403	0.002739	0.005428	5.52E-04	0.001136	2.04E-04
0	4.23E-04	0	2.85E-04	7.51E-04	0	5.10E-05	0	6.70E-05	5.96E-04	6.20E-04	0	4.09E-04
7.70E-04	8.48E-04	7.60E-05	2.49E-04	0.013914	0.002654	0.004522	1.73E-04	9.83E-04	3.18E-04	0.001687	0.004047	9.24E-04
0	0.001245	0	8.08E-04	0.002617	2.07E-04	7.22E-04	2.70E-04	2.31E-04	0.001919	6.16E-04	0.00253	0.001191
9.20E-05	0	0	6.51E-04	6.20E-05	0.008544	4.33E-04	0	0	0	3.00E-05	0	0.001353
1.69E-04	2.15E-04	8.60E-05	1.95E-04	0.00498	3.49E-04	7.23E-04	4.70E-05	3.00E-05	6.33E-04	0.001219	3.64E-04	0
0.002106	2.10E-04	0	4.40E-04	8.72E-04	0.001205	2.10E-05	7.40E-05	3.80E-05	1.11E-04	7.18E-04	1.43E-04	0.028526
0	1.50E-04	0	0	0.001189	0	1.80E-04	0	3.80E-05	4.20E-05	6.20E-04	0	7.65E-04
4.56E-04	4.44E-04	0	9.30E-05	0.001333	0.003278	1.80E-05	0	7.32E-04	7.64E-04	0.001378	4.51E-04	4.59E-04
0.009489	0.001329	0.002542	3.68E-04	5.30E-04	3.14E-04	1.48E-04	0	0.002555	2.37E-04	0.004381	2.89E-04	5.18E-04
0	0	0.010911	2.81E-04	0	0.001943	0.004429	0	3.56E-04	0	0	2.63E-04	0.002403
4.70E-05	4.20E-05	0.035161	7.31E-04	2.27E-04	0.004414	9.39E-04	4.70E-05	4.12E-04	0	9.10E-05	4.79E-04	0.00829
0.001275	4.20E-04	1.30E-04	6.72E-04	1.44E-04	7.69E-04	2.40E-05	3.70E-05	3.00E-04	8.40E-05	6.29E-04	1.33E-04	1.18E-04
1.28E-04	2.34E-04	0	5.41E-04	2.68E-04	0.007664	8.34E-04	0	3.40E-05	0	7.00E-05	2.70E-05	0.00163

0	0	0	4.18E-04	4.70E-05	6.24E-04	8.00E-05	4.40E-05	1.47E-04	3.48E-04	2.56E-04	3.20E-05	4.72E-04
3.80E-05	3.90E-05	0	4.75E-04	1.12E-04	0.005723	4.89E-04	0	4.40E-05	0	0	0	0.002842
0.02846	0	0.01143	2.05E-04	2.80E-05	0.001379	3.31E-04	4.60E-05	3.51E-04	3.30E-05	0	8.10E-05	0.002465
5.80E-05	3.90E-05	0	7.13E-04	7.30E-05	0	0	0	3.30E-05	7.60E-05	6.76E-04	3.50E-05	0.001972
0.00443	2.86E-04	3.05E-04	3.70E-05	0	9.80E-05	0.001409	0	1.49E-04	0	0.001294	8.10E-05	1.16E-04
9.80E-05	1.63E-04	0.012105	3.15E-04	1.19E-04	0.002818	4.82E-04	0	7.80E-05	1.27E-04	1.26E-04	1.21E-04	0.003618
0	0	0	0	5.01E-04	0	0	4.60E-05	0	0	0.006028	0	0.022651
0	0	0	0	3.18E-04	0	7.80E-05	2.25E-04	2.69E-04	9.15E-04	1.57E-04	0.002002	0
0	4.20E-05	0	0	0	0	0	4.00E-05	0	1.18E-04	6.90E-05	0	0
0	0	0	0	0	0	0	0	3.50E-05	3.70E-04	1.33E-04	0	0
0	3.80E-05	0.013555	3.16E-04	2.50E-05	0.002193	3.07E-04	0	0	0	0	3.48E-04	0.003437
0.009071	0.001044	0	8.90E-05	0	0	0	0	3.51E-04	0	7.50E-05	0	1.37E-04
1.76E-04	1.51E-04	4.84E-04	7.30E-04	2.38E-04	7.04E-04	3.50E-04	0.001204	0.001103	8.80E-05	0.001094	3.45E-04	3.32E-04
0	0	0	0	0	0	0	0	0	0	0	0	0
0	3.40E-05	0.012991	3.55E-04	8.10E-05	0.001872	2.96E-04	4.30E-05	0	0	0	8.90E-05	0.002518
0	1.18E-04	0	0	0	0	0	0	0	3.06E-04	1.01E-04	0	0
0.001555	5.79E-04	5.18E-04	4.50E-05	9.50E-05	0	0	4.80E-05	0.00105	3.60E-05	6.87E-04	1.45E-04	6.70E-05
0.004959	7.60E-05	0.008333	0	0	0	0	2.13E-04	3.40E-05	1.51E-04	1.82E-04	0	5.20E-05
0	0	0.007965	3.17E-04	2.20E-05	0.001836	2.54E-04	0	0	0	0	1.23E-04	0.002227
0	0	0	0	0	0	6.50E-05	1.22E-04	1.01E-04	1.57E-04	0	0	0
0	0	0	0	0	0	0.001109	0	0	0	0	0	0
0	0	7.30E-05	0	0	0	0.006543	0	0	0	0	0	0
3.31E-04	1.18E-04	0	0	0.001107	0	0	0	4.10E-05	0	4.74E-04	0	0.001021
0	0	0	0	0	0	0	0	0	0	0	0	0
0	0	0.004623	7.80E-05	2.20E-05	0.001015	1.03E-04	0	6.10E-05	0	7.10E-05	1.24E-04	0.001575
0	0	0	0	0	0	0	0	0	0	0	0	0
6.10E-05	1.13E-04	0	3.97E-04	1.20E-05	4.78E-04	1.09E-04	0	0	3.90E-05	3.10E-05	3.00E-05	5.80E-05
0	0	0	1.61E-04	2.68E-04	0	0	0	0	0	1.52E-04	0	0
0	0	2.04E-04	0	0	0	1.90E-05	0	0	0	0	0	0
4.90E-05	3.42E-04	0	0	0	0	0	0	0	0	3.80E-05	0	6.80E-05
0.020144	0.008744	0.124546	0.008466	0.01226	0.045936	0.031745	0.006059	0.015014	0.011547	0.014706	0.01076	0.041724

H112	H141	H090	H040	H135	H126	H039	H116	H117	H057	H013	H050	H106
0.246103	0.244181	0.238892	0.234824	0.232238	0.22905	0.226091	0.221657	0.220061	0.218167	0.216031	0.215632	0.214676
0.105964	0.11106	0.228176	0.123267	0.167306	0.045635	0.012337	0.043784	0.052089	0.276002	0.332065	0.088127	0.162345
0.032272	0.082338	0.028598	0.057079	0.04105	0.075868	0.045829	0.010107	0.010317	0.045045	0.03362	0.107687	0.048257
0.03613	0.12956	0.088435	0.054427	0.162621	0.102327	0.100581	0.103881	0.120947	0.070904	0.058135	0.070541	0.044297
0.051361	0.06983	0.101317	0.072129	0.050197	0.026992	0.086297	0.12671	0.083354	0.034083	0.026334	0.016731	0.023239
0.046437	0.040062	0.009003	0.039441	0.04372	0.080748	0.165699	0.099428	0.211571	0.064981	0.099308	0.045092	0.090802
0.194513	0.031285	0.078934	0.121762	0.038351	0.075835	0.085709	0.085654	0.038338	0.017819	0.075464	0.091295	0.076445
0.025626	0.048668	0.001516	0.016749	0.022199	0.07629	0.06354	0.029302	0.008378	0.010854	0.032523	0.039503	0.120311
0.030426	0.037941	0.037613	0.037682	0.017184	0.03328	0.023877	0.012636	0.010715	0.034902	0.021829	0.065486	0.027802
0.027072	0.045731	0.020822	0.028976	0.038483	0.027718	0.019617	0.031374	0.051589	0.029614	0.010266	0.031202	0.014685
0.01273	0.030106	0.006612	0.011091	0.004687	0.006122	0.002649	0.002546	0.00212	0.022024	0.003399	0.036011	0.005934
0.01337	0.004292	0.008112	0.016306	0.012716	0.009951	0.012207	0.023773	0.022033	0.017578	8.87E-04	0.001279	0.016938
0.001366	0.003172	2.15E-04	0.004732	0.001057	0.00529	0.005497	0.003118	0.001082	0.002796	0.001492	0.020142	0.006741
0.017648	0.009165	0.008651	0.006128	0.025413	0.014105	0.015442	0.019742	0.030311	0.010569	0.009932	0.004018	0.004125
0.01041	0.001553	0.028758	0.025507	0.014231	0.006059	0.009515	0.026979	0.008079	0.007933	0.002717	0.010253	0.010388
0.00341	0.010951	0.002766	0.005196	0.002474	0.005569	0.006237	0.002043	0.001668	0.005051	0.004137	0.005534	0.006523
0.019152	0.003831	0.01842	0.007409	0.015724	0.010383	0.007313	0.007546	0.015971	0.033236	0.00455	0.007441	0.01767
8.20E-05	1.71E-04	0	3.60E-04	0	1.74E-04	0	1.60E-05	2.60E-05	7.50E-05	0	1.86E-04	4.80E-05
0	0	0	0	0	0	0	0	0	0	4.99E-04	0	0
0.002545	0.00138	0.012762	0.016965	4.17E-04	0.001335	1.83E-04	2.40E-05	1.47E-04	0.002289	0.001461	3.72E-04	5.90E-05
4.52E-04	0.002591	4.03E-04	0.002368	2.05E-04	7.66E-04	3.47E-04	7.30E-05	4.30E-04	0.002121	4.60E-05	0.009083	0.001935
0.020349	1.84E-04	0	0.003499	0.022281	0.010886	2.51E-04	0.002014	0.001278	0.023169	0.003044	1.29E-04	0.00743
0.004338	0.013768	0.003817	0.004841	0.004342	0.00406	0.007699	0.007851	0.009289	0.005375	0.003144	0.004509	0.002617
0.002586	0.005305	0.001167	0.001622	8.98E-04	0.004838	0.005699	0.006167	0.005395	0.005166	0.003325	0.001931	0.005939
0.01141	0.002576	0.020028	0.010588	0.008066	0.002106	0.005162	0.012488	0.01032	0.006242	0.001251	0.001183	0.002112
5.17E-04	4.00E-05	1.05E-04	2.78E-04	5.00E-05	3.78E-04	5.50E-05	8.30E-05	2.90E-05	4.78E-04	0	1.83E-04	0.001126
0.004946	0.004678	0.001381	0.004967	0.00247	0.006974	0.002029	0.00761	0.001668	0.005765	0.003102	0.004666	0.010106
0.00423	0.005016	4.72E-04	0.001996	0.012178	0.002725	0.041665	0.010681	0.018254	0.002072	0.006101	0.001843	0.003442
0.006825	0.003444	0.002962	0.021133	0.006248	0.00115	0.008312	0.003713	0.022649	0.003133	0.010428	0.018026	0.001871
0.002243	8.12E-04	0	0.004416	0.004723	0.005503	0.011383	0.008684	0.002522	0.007644	0.003693	0.001873	0.006643
0.001556	0.002431	1.36E-04	0.003858	3.26E-04	0.002504	1.83E-04	3.36E-04	2.83E-04	7.55E-04	0	0.001016	0.002846
0.009402	0.010025	0.022219	0.002747	0.012843	0.005068	0.005028	0.053952	0	0	0.001283	0.00191	0.002259
0.003136	0.003548	0.001599	0.002604	0.001841	0.006101	0.001837	0.004806	0.008542	0.002552	0.005876	0.004987	0.003237
0.003203	0.002696	0.001247	0.004898	0.003195	0.004565	0.001404	0.003955	0.002805	0.001583	0.002132	0.006575	0.002119
0.010466	9.43E-04	0.012657	0.009481	0.00579	0.006186	8.75E-04	6.08E-04	0.003332	5.73E-04	5.67E-04	0.008467	0.003324
0	0	0	7.70E-05	0	0	1.18E-04	0	0	2.30E-05	5.10E-05	1.20E-05	8.20E-05
0.007545	0.001223	4.30E-05	8.24E-04	6.86E-04	0.003335	0.003599	0.005885	0.008915	0.004134	0.004972	0.00256	0.005474
0.002095	0.011086	8.78E-04	0.002663	0.010275	7.05E-04	4.67E-04	1.97E-04	3.34E-04	0.001444	8.10E-05	0.004446	8.52E-04
0.001331	9.05E-04	1.91E-04	8.90E-04	2.33E-04	0.039131	9.01E-04	9.39E-04	0.001047	3.91E-04	6.83E-04	0.011048	0.009038
3.34E-04	0	0	2.15E-04	0	2.51E-04	6.30E-05	1.18E-04	8.80E-05	2.39E-04	0	1.67E-04	3.08E-04
8.90E-05	8.80E-05	0	9.90E-05	0	2.54E-04	6.30E-05	1.47E-04	2.13E-04	1.18E-04	8.60E-05	5.23E-04	1.31E-04
2.09E-04	3.12E-04	6.44E-04	0.002647	5.99E-04	2.26E-04	2.56E-04	1.52E-04	0.001856	9.97E-04	4.80E-05	4.57E-04	2.13E-04
0.002783	0.002487	1.06E-04	0.00216	5.35E-04	0.001177	3.70E-05	2.62E-04	2.90E-05	4.25E-04	2.94E-04	6.61E-04	0.001177
0	3.19E-04	0	1.86E-04	1.91E-04	9.63E-04	2.07E-04	2.30E-05	3.30E-05	1.87E-04	0.001	0.001634	9.39E-04
0.001016	3.29E-04	0	0.001659	0.00177	0.005766	4.23E-04	3.58E-04	2.27E-04	0.001203	0.002443	0.004102	0.001092
8.78E-04	3.60E-04	1.81E-04	0.002249	2.47E-04	0.002345	5.47E-04	8.54E-04	8.61E-04	0.002862	2.30E-04	5.53E-04	0.001355
2.52E-04	3.90E-05	0	2.02E-04	0	2.17E-04	0	1.40E-05	3.00E-05	1.16E-04	0	1.83E-04	1.55E-04
7.76E-04	2.06E-04	1.02E-04	4.31E-04	1.82E-04	0.004038	1.98E-04	0.00164	7.40E-04	1.54E-04	1.05E-04	4.33E-04	0.001718
0.001624	0.00718	4.50E-05	2.52E-04	7.90E-05	8.07E-04	5.34E-04	5.94E-04	9.40E-05	1.95E-04	4.70E-05	0.001841	1.04E-04
2.20E-05	4.50E-05	0	1.04E-04	1.19E-04	8.60E-05	0	0	0	1.63E-04	0	4.25E-04	2.33E-04
4.45E-04	2.94E-04	9.00E-05	0.001455	4.20E-05	0.004833	5.90E-04	5.50E-04	4.63E-04	3.04E-04	0.001734	0.003146	0.004182
0.005688	5.70E-04	0	0.004028	3.70E-05	8.80E-04	9.47E-04	7.35E-04	3.34E-04	7.96E-04	3.36E-04	0.00178	0.001504
5.60E-05	0	0	7.50E-05	0	2.50E-05	0	0	0	5.40E-05	0	0	4.80E-05
9.80E-05	0	0	1.03E-04	0	2.30E-05	0	1.70E-05	5.60E-05	1.87E-04	0	0	2.65E-04
3.00E-05	4.78E-04	1.69E-04	7.82E-04	0.003354	4.47E-04	2.54E-04	6.00E-05	2.66E-04	1.41E-04	0	6.84E-04	4.93E-04
6.50E-05	7.20E-05	0	1.83E-04	0	1.45E-04	3.10E-05	0	0	1.80E-04	4.10E-05	0	1.22E-04

8.94E-04	3.24E-04	1.99E-04	4.63E-04	0	2.53E-04	6.60E-05	3.66E-04	1.27E-04	6.80E-05	2.84E-04	1.27E-04	0.001288
1.28E-04	4.10E-05	0	0	0	9.80E-05	0	0	0	8.50E-05	0	6.10E-05	1.60E-04
3.10E-05	0	4.60E-05	1.39E-04	0	2.20E-05	0	8.10E-05	0	2.70E-04	0	3.50E-05	1.01E-04
0	4.30E-04	4.70E-05	1.43E-04	7.58E-04	0	9.20E-05	2.00E-05	2.90E-05	4.90E-05	0	1.53E-04	2.20E-05
0	0	0	3.20E-05	0	3.00E-05	0	0	0	9.30E-05	0	6.10E-05	0
2.50E-05	1.60E-05	1.59E-04	6.50E-05	0	0.00103	1.18E-04	0	2.40E-05	4.00E-05	0	2.55E-04	2.81E-04
0	0	0	0	0	1.18E-04	0	2.10E-05	0	0	0	0	0
1.69E-04	0.001079	0	2.99E-04	8.40E-05	6.36E-04	0	9.56E-04	0.001078	0	3.85E-04	2.10E-05	5.42E-04
0	1.39E-04	3.90E-05	0	0	0	7.20E-05	2.00E-05	0	0	3.60E-05	0	0
0	1.76E-04	7.70E-05	0	8.80E-05	0	4.70E-05	3.00E-05	3.20E-05	0	1.64E-04	0	0
0	0	0	3.80E-05	0	1.80E-05	0	1.70E-05	0	7.10E-05	0	1.10E-05	1.50E-04
0	0	0	0	0	2.00E-05	8.80E-05	2.61E-04	0	2.99E-04	0	1.37E-04	0
3.56E-04	3.91E-04	0.003425	6.10E-04	8.10E-04	2.64E-04	1.50E-04	8.83E-04	2.35E-04	0.002335	1.30E-04	5.35E-04	9.80E-05
0	0	0	0	0	0	0	0	0	0	0	0	0
2.80E-05	0	0	0	0	0	0	0	0	0	0	0	1.10E-04
0	5.10E-05	9.00E-05	0	0	0	5.10E-05	0	0	0	5.05E-04	2.20E-05	0
1.06E-04	1.41E-04	0	3.80E-05	0	5.40E-04	5.65E-04	5.13E-04	0	9.10E-05	0	1.45E-04	0
0	6.20E-05	1.01E-04	0.001512	1.14E-04	5.00E-05	1.16E-04	1.44E-04	0	2.00E-05	0	0.001152	5.30E-05
0	0	0	7.90E-05	0	2.36E-04	0	1.60E-05	0	2.50E-05	0	0	3.70E-05
1.05E-04	0	1.58E-04	0	0	5.90E-04	0	0	0	4.85E-04	0	0	3.73E-04
0	0	0	0	0	0	0	0	0	0	0	0	0
0	0	0	0	0	0	7.80E-05	0	0	0	0	0	0
1.23E-04	0	0	0	0	2.30E-05	0	0	0	1.23E-04	2.21E-04	0.013719	6.27E-04
0	0	0	0	0	0	0	0	0	0	0	0	0
3.30E-05	0	0	3.60E-05	0	2.30E-05	2.60E-05	0	0	7.40E-05	0	0	3.70E-05
0	0	0	0	0	0	0	0	0	0	0	0	0
2.50E-05	0	3.90E-05	2.25E-04	0	0	0	0	0	0	0	0	1.80E-05
0	0	0	0	0	5.50E-05	0	0	0	0	0	1.79E-04	0
0	0	0	0	0	0	0	0	0	0	0	0	0
0	0	0	0	0	0	0	0	0	0	3.60E-05	5.10E-05	0
0.010365	0.007823	0.005376	0.015711	0.004273	0.033799	0.008747	0.010786	0.007297	0.011009	0.007447	0.025768	0.018327

H034	H150	H030	H091	H053	H115	H021	H105	H023	H146	H010	H076	H058
0.214486	0.214012	0.209473	0.206035	0.205858	0.205661	0.203333	0.196352	0.196037	0.194206	0.19206	0.19025	0.189019
0.132045	0.266065	0.030587	0.044864	0.02148	0.094741	0.09777	0.049038	0.172836	0.119322	0.155496	0.085654	0.057025
0.034083	0.08177	0.079173	0.079643	0.011427	0.064071	0.064517	0.070554	0.050403	0.022034	0.121161	0.009318	0.032899
0.065379	0.088876	0.094959	0.191162	0.005423	0.111965	0.063936	0.041894	0.060925	0.010249	0.043033	0.054841	0.031826
0.031348	0.016453	0.051847	0.038179	0.002043	0.145842	0.034436	0.012416	0.019044	0.020002	0.009388	0.276493	0.03885
0.017436	0.042358	0.041373	0.044063	9.60E-04	0.07196	0.10635	0.064373	0.075143	0.175879	0.0414	0.063595	0.038292
0.029245	0.063292	0.086658	0.064455	0.002251	0.011672	0.049172	0.114774	0.085049	0.010045	0.115904	0.076551	0.018382
0.007227	0.036042	0.033475	0.039373	0.006343	0.011387	0.096733	0.027714	0.069649	0.200019	0.022708	0.01988	0.023585
0.033577	0.02469	0.036144	0.021661	0.004451	0.073307	0.010359	0.122836	0.039123	0.005327	0.040921	0.014845	0.016426
0.02916	0.030653	0.022006	0.051428	5.91E-04	0.016194	0.017486	0.010365	0.00913	0.010897	0.020763	0.014995	0.022492
0.005246	0.022854	0.015284	0.002518	3.51E-04	0.03037	0.008979	0.014313	0.013226	0.033181	0.027906	0.003653	0.011311
0.002349	0.004875	0.00292	0.013512	3.54E-04	0.019867	0.014639	0.007649	0.013658	0.049103	0.003299	0.007713	0.00433
0.001992	0.002419	0.003898	0.004763	3.46E-04	0.005428	0.007217	0.024353	0.006898	4.19E-04	0.00603	0.001142	0
0.009886	0.007912	0.007374	0.004207	1.18E-04	0.02661	0.007433	6.73E-04	0.006933	0.019031	0.002627	0.015542	0.002984
0.066058	0.00745	0.014416	0.005515	3.44E-04	0.008184	0.01144	0.00386	0.005508	0.018126	0.009452	0.004792	0.004339
0.005873	0.019967	0.002858	0.004507	0	0.011622	0.003859	0.006679	0.00679	0.002522	0.009714	0.001534	0.008364
0.006	0.006057	0.009429	0.018693	0	0.004057	0.019726	0.006921	0.008928	0.008483	0.007806	0.025082	0.006758
0	0	2.80E-05	5.86E-04	0	5.10E-05	2.39E-04	0.00121	0	0	0.001368	0	0
0	0	0	0	0	0	1.90E-05	6.33E-04	0.079584	0.002258	1.02E-04	0	0
9.61E-04	0	7.79E-04	0	0	0.001505	0.003686	0.016007	0.007833	3.00E-05	0	9.87E-04	0
4.32E-04	1.85E-04	0.001901	0.003038	0	4.00E-05	0.001447	0.005591	0.00298	0.001145	0.001842	9.90E-05	3.88E-04
0.009064	0.0078	0.003337	0.001632	0.001603	2.35E-04	0.004446	6.79E-04	0.009294	0.030073	0.001617	0.023667	0.003117
0.00458	0.004962	0.004378	0.003179	0	0.007561	0.005886	0.001574	0.003399	0.005232	0.002793	0.007989	0.004555
0.001546	0.002337	7.30E-04	0.003185	0	0.00111	0.003947	0.00388	0.002636	0.006328	0.004001	9.50E-04	0.0018
0.002334	0.001597	0.008432	0.00617	2.25E-04	0.005798	0.001845	4.97E-04	5.95E-04	0.003987	8.78E-04	0.01615	0.001997
0.00563	0	0.002287	0.002983	0.148804	1.17E-04	2.71E-04	0.018295	3.80E-05	0.002702	0.020211	5.00E-05	0.099022
0.003954	0.006555	0.003583	0.004448	0	0.005468	0.00586	8.40E-04	0.00385	0.002426	0.003354	0.007248	0.00186
0.003886	0.006144	0.003506	0.001464	0.002017	0.016346	0.004822	5.01E-04	9.81E-04	1.48E-04	1.44E-04	0.005413	0.004479
0.005369	0.003579	0.017891	0.002589	8.00E-04	0.012752	0.00359	1.89E-04	0.003045	2.60E-05	0.001522	0.013867	0.00185
0.012988	0.001576	0.004897	6.04E-04	0.004563	0.009489	0.003326	0.001119	0.001807	0.001956	0.0011	0.002948	0.003856
1.37E-04	3.07E-04	7.89E-04	0.010904	0.016922	0	0.001741	0.045022	4.40E-04	2.43E-04	0.035404	3.66E-04	0.011082
0.009085	0.00339	0.006198	9.05E-04	1.17E-04	0.011674	1.93E-04	0	0.00156	0.001263	0.001202	0.020095	6.78E-04
0.002041	0.002871	0.002225	9.44E-04	1.21E-04	0.002256	0.002046	0.002287	0.002775	0.008241	0.002996	0.001743	0.001277
0.003122	0.002692	0.00205	0.004268	0	0.002072	0.004203	0.001304	0.001812	0	0.001362	0.001959	2.87E-04
0.001816	3.23E-04	0.017392	0.003381	1.04E-04	1.34E-04	0.007791	0.006127	0.008952	7.66E-04	3.57E-04	0.003465	0.002525
0.038128	0	0.045375	2.78E-04	0.017037	0	1.90E-05	0.001189	0	1.84E-04	0.001242	2.00E-05	0.005103
7.25E-04	6.75E-04	0.003048	0.002632	0	5.60E-05	0.004349	1.60E-04	6.14E-04	0.00193	3.81E-04	0.006005	0.004655
3.61E-04	5.20E-05	0.002272	0.004908	0	8.90E-05	0.018666	5.91E-04	6.66E-04	3.96E-04	7.51E-04	2.83E-04	0.001012
9.67E-04	2.00E-04	0.003896	0.006165	0	4.30E-05	0.009006	0.006804	0.001527	0	2.01E-04	8.01E-04	0.003768
5.10E-05	0	5.60E-05	0.001957	0.088513	2.47E-04	1.24E-04	0.013961	1.09E-04	0.001845	0.012247	2.50E-05	0.060029
0.049579	3.80E-05	0.010051	2.91E-04	0.02359	3.64E-04	3.10E-04	0.003012	7.00E-05	2.59E-04	0.002749	5.30E-05	0.012499
3.00E-04	1.22E-04	5.01E-04	5.46E-04	0	3.60E-05	6.78E-04	0.001016	5.67E-04	4.28E-04	7.70E-05	6.97E-04	0
0.004411	0.009337	0.002913	0.002146	0	0.002244	0.001139	0	0.001434	0.012279	0.003629	0.002219	0
3.23E-04	1.48E-04	7.60E-04	8.10E-05	0	0	7.53E-04	0.00109	0.004306	0	0	1.00E-04	0.001383
2.52E-04	2.60E-04	0.001152	0.004748	0	9.40E-05	0.003748	0.001542	0.003147	4.50E-05	0.001434	0.001375	0
7.65E-04	1.59E-04	7.96E-04	0.001714	0	0.00168	5.12E-04	0.004805	0.002315	0.00108	3.49E-04	5.45E-04	0
0	0	2.00E-05	9.42E-04	0.049922	0	2.80E-05	0.006679	3.40E-05	0	0.001637	1.50E-05	0.023548
6.70E-05	6.10E-05	2.13E-04	4.10E-04	1.21E-04	0	0.003689	9.76E-04	6.02E-04	6.60E-05	6.00E-05	0.002407	9.99E-04
0	0	0.034395	0.012665	0	0	2.45E-04	3.30E-05	1.12E-04	0	9.41E-04	0.001607	0.004885
8.50E-05	0	2.40E-05	0	0	0	1.75E-04	0	8.61E-04	0	0	0	0
4.01E-04	6.30E-05	0.001874	0.001416	0	0	0.014249	0.001002	0.00112	0	1.10E-04	3.43E-04	9.90E-05
9.20E-05	1.22E-04	0.00104	0.005828	6.83E-04	1.27E-04	0.003499	6.89E-04	7.13E-04	0	6.24E-04	6.42E-04	0.001273
0.022804	0	0.007834	2.80E-04	0.013947	0	5.20E-05	0.002599	0	4.48E-04	0.001929	2.20E-05	0.00804
3.10E-05	0	8.30E-05	9.81E-04	0.039963	0	3.80E-05	0.00535	3.90E-05	7.74E-04	0.005917	0	0.021459
2.77E-04	3.50E-05	4.45E-04	0.022928	0	0	0.001376	3.44E-04	6.29E-04	2.54E-04	0	1.12E-04	0.001568
3.40E-05	6.40E-05	6.90E-05	7.39E-04	0.02436	0	2.14E-04	0.00606	7.60E-05	0	0.00368	2.60E-05	0.00687

1.39E-04	4.80E-05	2.31E-04	0.005684	0	0	9.98E-04	0.001547	3.10E-05	1.76E-04	0.001338	0.00197	0
0	0	0	4.44E-04	0.028562	0	1.70E-05	0.006051	0	0	9.87E-04	2.60E-05	0.021061
0	0	7.90E-05	0.001008	0.015956	5.40E-05	2.40E-05	0.002392	8.10E-05	2.69E-04	0.001523	2.00E-05	0.009896
0	9.40E-05	2.08E-04	0.002735	0	0	4.30E-05	0	0	0	8.09E-04	2.00E-05	1.01E-04
0.003874	0	0.001157	1.79E-04	4.51E-04	0	2.90E-05	1.21E-04	4.20E-05	2.40E-05	2.11E-04	2.10E-05	4.01E-04
3.48E-04	0	3.35E-04	3.47E-04	0.016551	4.70E-05	4.72E-04	0.002622	0	2.62E-04	0.002444	0	0.007761
0	0	0	0	0	0	0.02174	0	0	0	0	0	0
3.21E-04	1.56E-04	6.50E-05	1.47E-04	0	0	5.31E-04	5.30E-05	4.11E-04	0.001851	0	2.40E-05	0.001305
0	1.34E-04	8.10E-05	0	0	1.32E-04	0	0	0	0	0	0	0
0	5.40E-05	2.50E-05	0	0	0	0	0	2.30E-05	0	0	3.50E-05	0
0	0	0	3.57E-04	0.016552	4.80E-05	8.50E-05	0.002227	0	3.32E-04	0.002965	0	0.01078
0	0	0.002564	0.004035	0	4.40E-05	2.72E-04	0	0	0	8.80E-05	1.91E-04	0
1.83E-04	1.87E-04	7.40E-05	3.70E-05	0	3.05E-04	1.72E-04	0	2.11E-04	2.60E-05	5.73E-04	6.02E-04	2.86E-04
0	0	0	0	0	0	0	0	0	0	0	0	0
0	0	2.00E-05	2.23E-04	0.014445	4.00E-05	7.00E-05	0.002317	3.90E-05	3.32E-04	0.002304	0	0.010165
0	1.55E-04	1.90E-05	0	0	0	0	0	3.30E-05	0	0	0	0
0	0	2.91E-04	7.21E-04	0	0	3.07E-04	0	0	0	1.48E-04	1.01E-04	2.08E-04
3.80E-05	0	0.001515	2.28E-04	0	0	1.90E-04	0	3.40E-05	0	0	0.001155	0
0	0	0	1.11E-04	0.01073	0	6.16E-04	0.001669	4.00E-05	1.69E-04	0.001975	0	0.008902
1.17E-04	0	6.40E-05	0	0	4.60E-05	4.30E-05	0	2.86E-04	1.04E-04	1.19E-04	1.31E-04	0
0.010519	0	0.002344	0	0	0	0	0	0	0	0	0	0
0.012835	0	0.01146	0	0	0	0	2.50E-05	0	0	0	0	0
0	0	0	0	0	0	0.00107	0	1.91E-04	0	0	0	0
0	0	0	0	0	0	0	0	0	0	0	0	0
0	0	0	1.54E-04	0.018252	0	1.50E-05	0.001045	0	2.45E-04	0.001007	0	0.00501
0	0	0	0	0	0	0	0	0	0	0	0	0
0	0	5.20E-05	1.41E-04	0	0	0	9.73E-04	2.80E-05	0	0.001006	1.70E-05	0
0	0	0	2.46E-04	0	0	0	1.84E-04	0	0	1.16E-04	0	0
0.026175	0	1.70E-05	0	0	0	0	0	0	0	0	0	0
0	0	2.13E-04	8.53E-04	0	0	1.56E-04	0	0	0	5.30E-05	0	5.13E-04
0.067463	0.007773	0.040092	0.025837	0.182749	0.004758	0.037538	0.040353	0.008748	0.010553	0.034455	0.005514	0.120766

H120	H093	H098	H035	H028	H067	H113	H127	H003	H044	H092	H142	H029
0.185874	0.181027	0.180327	0.178113	0.177375	0.176786	0.176761	0.175645	0.173383	0.172777	0.171099	0.170413	0.169099
0.046731	0.268727	0.054211	0.088094	0.173307	0.303732	0.131613	0.168372	0.073752	0.18714	0.09638	0.040731	0.105832
0.076673	0.070816	0.098889	0.050206	0.023337	0.027772	0.086317	0.050147	0.16202	0.045008	0.069329	0.067191	0.066918
0.044698	0.063654	0.087422	0.050013	0.049775	0.053066	0.038711	0.063809	0.039286	0.113098	0.064697	0.239579	0.075981
0.005852	0.023434	0.072818	0.008716	0.028729	0.042553	0.019434	0.050821	0.013353	0.060344	0.033358	0.076694	0.016272
0.030242	0.073123	0.030717	0.113977	0.037432	0.1124	0.037616	0.089741	0.058637	0.12621	0.11591	0.053956	0.019621
0.081461	0.043726	0.127276	0.10486	0.06858	0.057276	0.125026	0.082207	0.074369	0.008489	0.150897	0.044401	0.1095
0.013685	0.103091	0.008927	0.165351	0.036895	0.021218	0.080763	0.045763	0.059444	0.010936	0.060746	0.030301	0.0393
0.133126	0.053264	0.121267	0.072951	0.027437	0.066892	0.026385	0.042757	0.101093	0.059542	0.046283	0.046697	0.052727
0.017154	0.009598	0.018425	0.009664	0.013126	0.028484	0.009848	0.022632	0.024025	0.089321	0.006207	0.044135	0.016779
0.039073	0.007086	0.0165	0.005179	0.008142	0.006275	0.011677	0.007952	0.033475	0.021856	0.003353	0.018921	0.011875
0.003445	0.025796	0.002536	0.003602	0.002803	0.004528	0.056118	0.030277	0.006698	0.004749	0.014158	0.015392	0.002303
0.004125	0.00238	4.95E-04	0.006266	0.002361	0.002595	0.021163	0.006335	0.014033	0.006693	0.014258	0.009764	0.005659
0.004215	0.009651	0.006521	0.0065	0.006418	0.013695	0.003741	0.015597	0.002929	0.009977	0.006764	0.012134	0.002853
8.77E-04	0.005879	0.00973	0.014376	0.013354	0.010774	0.008787	0.011563	6.28E-04	0.004268	0.010559	0.014805	0.011421
0.005484	0.005028	0.003048	0.009737	0.003794	0.003756	0.011751	0.013614	0.005198	0.006157	0.007514	0.011101	0.004855
0.005103	0.004619	0.019176	0.00514	0.012204	0.005854	0.006276	0.015604	0.004419	0.009734	0.008232	0.004605	0.010411
1.19E-04	0	1.59E-04	1.59E-04	0	1.06E-04	8.70E-05	3.60E-05	6.12E-04	1.14E-04	0	1.34E-04	1.46E-04
0	6.60E-05	0	0	0	3.00E-05	0	0	0	0	0	0	2.50E-05
0.232531	0.001497	0.010234	2.70E-04	0.014309	0.004405	8.60E-04	3.99E-04	0.056267	7.02E-04	2.09E-04	0.002195	1.30E-04
6.23E-04	2.02E-04	7.49E-04	3.12E-04	3.99E-04	0.004275	0.002137	0.001381	0.005048	4.27E-04	4.90E-04	0.021159	6.52E-04
8.50E-04	0.008238	0.051143	0.011856	0.007841	4.86E-04	0.019712	0.014171	1.51E-04	2.92E-04	0.010658	0	0.001031
0.003486	0.001949	0.006737	0.006214	0.004729	0.00509	0.00551	0.011641	0.003728	0.004859	0.003706	0.006552	0.004339
0.002451	0.001847	0.001385	0.011165	0.002144	0.005815	0.003767	0.005422	0.003684	0.00291	0.009254	0.0012	0.001639
6.72E-04	0.001294	0.00679	4.33E-04	0.00266	0.002796	9.87E-04	0.009397	6.04E-04	6.11E-04	0.002122	0.0047	0.002173
0.001592	3.40E-05	7.92E-04	0.001242	0.001766	4.13E-04	0.002802	2.00E-04	0.009167	2.54E-04	1.66E-04	0	0.005135
0.002542	0.002869	0.003209	0.005514	0.004917	0.002258	0.002644	0.005472	0.002154	0.002029	0.009878	0.003413	0.002436
0.002728	0.002094	0.003988	5.63E-04	0.002491	0.003556	9.10E-04	0.005172	0.003279	0.007475	0.001429	0.005311	0.003062
0.003129	0.001298	0.008724	1.50E-05	0.005684	7.27E-04	0.003619	6.53E-04	5.60E-04	0.011714	8.72E-04	0.005867	0.008117
0.001448	0.00415	8.10E-04	2.66E-04	0.004264	2.21E-04	0.004527	0.004056	6.51E-04	0.002072	0.002622	0.00761	0.012178
0.005471	1.99E-04	0.00722	0.001726	4.57E-04	7.39E-04	0.005595	9.94E-04	0.008317	0.001205	0.001278	0.002329	0.002923
0.001082	0.00124	0.009542	0	0.006317	5.79E-04	0.00112	0.003223	9.80E-04	3.10E-05	0.001936	0.001939	0.004082
0.002259	0.001282	0.001296	0.008588	0.002667	0.004673	0.001303	0.002398	0.00203	0.001209	0.004636	0.00297	0.002616
0.002537	0.001409	0.007779	7.15E-04	0.002294	0.001208	0.001323	0.001346	9.01E-04	0.003545	0.003265	0.003111	0.002565
0.00794	8.74E-04	0.001629	0.003546	7.02E-04	6.59E-04	0.01535	0.008969	0.005645	0.003068	0.012908	0.003334	0.002259
4.40E-05	0	3.70E-05	3.40E-05	0.034891	0	1.24E-04	3.00E-05	4.39E-04	5.10E-05	0	2.40E-05	0.076415
3.17E-04	0.002184	9.70E-04	0.001976	0.001965	9.52E-04	0.001032	0.003073	3.61E-04	0.002488	0.003424	0.001434	7.19E-04
7.73E-04	2.60E-04	5.60E-04	6.67E-04	7.36E-04	6.94E-04	0.006162	7.80E-05	0.001312	7.18E-04	2.91E-04	6.89E-04	7.42E-04
0.006355	4.18E-04	9.72E-04	0.003406	3.01E-04	0.001077	0.001229	9.24E-04	1.17E-04	0.001003	0.004738	2.77E-04	0.001982
0.001009	9.10E-05	5.83E-04	7.35E-04	4.20E-05	3.80E-05	0.002005	7.50E-05	0.004819	1.44E-04	1.13E-04	5.10E-05	0.001715
2.65E-04	0	1.92E-04	2.82E-04	0.04929	3.60E-05	4.88E-04	7.90E-05	0.001755	1.79E-04	0	5.70E-05	0.025043
1.08E-04	5.60E-04	3.09E-04	2.29E-04	2.48E-04	1.00E-04	0.003598	0.001087	4.10E-05	6.11E-04	3.52E-04	0.003546	5.43E-04
0	0.004895	0	3.73E-04	0.002432	4.91E-04	0.008857	0.003582	0	1.24E-04	0.012766	1.32E-04	7.93E-04
2.90E-05	4.19E-04	0	6.34E-04	3.90E-05	6.40E-05	0.005401	3.10E-04	0.002522	0	0.00281	4.08E-04	1.65E-04
8.96E-04	5.43E-04	1.34E-04	0.012045	7.18E-04	7.63E-04	0.0019	0.005092	0.001618	9.29E-04	0.006939	2.99E-04	5.34E-04
3.85E-04	8.56E-04	0.002178	7.03E-04	0.00108	5.29E-04	0.002468	0.001118	0.001138	9.93E-04	0.001355	0.001465	0.001499
9.65E-04	3.80E-05	5.67E-04	7.50E-05	5.00E-05	1.39E-04	0.001847	0	0.001062	5.50E-05	0	2.60E-05	3.39E-04
8.90E-05	0.00105	4.10E-05	0.00175	1.36E-04	0.002438	7.09E-04	0.002608	4.90E-04	2.12E-04	9.42E-04	1.79E-04	5.80E-05
3.02E-04	6.60E-05	1.15E-04	4.30E-05	0	7.50E-05	3.91E-04	1.03E-04	8.05E-04	1.36E-04	1.22E-04	0.009068	3.59E-04
0	1.06E-04	0	0	3.70E-05	0	0.005552	0	0	0	5.36E-04	0	0
0.001516	2.30E-04	5.60E-05	0.005259	0	0.001575	6.60E-05	3.28E-04	7.60E-05	8.27E-04	0.002	2.30E-05	9.41E-04
6.81E-04	3.65E-04	2.05E-04	0.001751	0.001631	6.33E-04	3.80E-04	2.73E-04	7.70E-05	0.003907	0.001673	2.01E-04	0.001374
2.18E-04	0	2.09E-04	7.10E-05	0.038458	0	2.41E-04	7.60E-05	0.001145	4.70E-05	4.40E-05	0	0.021649
5.16E-04	0	2.85E-04	0.003056	0	0	9.17E-04	8.20E-05	0.002129	2.70E-05	0	2.80E-05	7.59E-04
3.14E-04	2.21E-04	0	6.31E-04	1.24E-04	3.32E-04	8.24E-04	7.60E-05	5.24E-04	2.35E-04	3.26E-04	1.41E-04	7.41E-04
8.34E-04	2.70E-05	6.22E-04	1.59E-04	0	0	0.002801	2.60E-04	0.001228	1.12E-04	3.60E-05	0	4.25E-04

2.51E-04	1.04E-04	7.80E-05	3.97E-04	1.69E-04	3.23E-04	7.15E-04	0	2.12E-04	1.34E-04	3.17E-04	0	0
6.05E-04	0	4.30E-04	0	2.90E-05	0	8.50E-04	0	9.04E-04	8.40E-05	0	0	5.23E-04
1.32E-04	3.00E-05	5.30E-05	2.64E-04	0	0	8.89E-04	0	7.86E-04	2.20E-05	0	2.00E-05	3.84E-04
6.73E-04	0	4.40E-05	2.20E-05	0	3.40E-05	1.47E-04	4.00E-05	2.99E-04	0.001267	7.90E-05	5.19E-04	7.70E-05
1.88E-04	0	4.20E-05	2.00E-05	0.026305	0	1.28E-04	0	7.90E-05	0	3.40E-05	0	0.00319
1.83E-04	0	1.61E-04	2.01E-04	1.55E-04	0	4.79E-04	7.60E-05	0.001298	1.83E-04	2.58E-04	0	0.002143
2.92E-04	0	0	0	0	0	0	1.53E-04	0	0	0	0	0
0	5.50E-05	6.01E-04	9.64E-04	5.86E-04	0.003535	1.81E-04	8.60E-04	0	0	2.99E-04	0	0
0	2.58E-04	0	8.70E-05	0	4.20E-05	0	0	0	0	0	1.34E-04	0
2.70E-05	1.75E-04	0	3.00E-05	0	3.50E-05	0	4.50E-05	0	0	3.40E-05	1.26E-04	0
2.28E-04	0	1.34E-04	0.001577	0	0	3.75E-04	4.00E-05	0.001437	0	0	0	1.83E-04
0	8.90E-05	0	0	0	0	0	0	0	0	0	7.93E-04	6.90E-05
1.45E-04	2.80E-05	0.001901	0	3.71E-04	3.10E-05	1.27E-04	3.77E-04	1.68E-04	8.80E-05	4.30E-04	3.55E-04	0.001051
0	0	0	0	0	0	0	0	0	0	0	0	0
1.99E-04	0	1.15E-04	5.20E-05	0	0	3.06E-04	4.10E-05	0.001047	2.40E-05	0	0	3.54E-04
0	3.77E-04	0	4.30E-05	0	9.70E-05	0	0	0	0	3.30E-05	9.60E-05	0
0	3.61E-04	2.10E-05	1.20E-05	2.80E-04	2.23E-04	5.20E-05	4.50E-05	0	0	7.80E-05	3.02E-04	6.20E-05
0	8.80E-05	4.03E-04	1.20E-05	0.00093	0	0	0	0	0	0	0	2.80E-05
1.11E-04	0	6.60E-05	0	0	0	2.37E-04	0	0.001018	2.10E-05	4.70E-05	0	1.36E-04
0	3.70E-05	5.00E-05	0	0	0	0.001076	0	0	0	0	0	3.30E-05
0	0	0	0	0.038365	0	0	0	0	0	0	0	0.00288
0	0	0	0	0	0	0	0	0	0	0	0	0.011767
0	0	0	0	0	0	0	2.94E-04	0	1.66E-04	0	0	0
0	0	0	0	0	0	0	0	0	0	0	0	0
9.20E-05	0	1.40E-04	0	0	0	7.97E-04	0	3.48E-04	3.50E-05	0	0	1.91E-04
0	0	0	0	0	0	0	0	0	0	0	0	0
1.45E-04	0	4.37E-04	1.50E-05	0	1.80E-05	1.31E-04	3.30E-05	3.66E-04	0	0	0	2.90E-05
0	0	6.70E-05	0	4.50E-05	0	2.99E-04	8.70E-05	0	0	0	2.30E-05	4.80E-05
0	0	0	0	0.009057	0	0	0	0	0	0	0	0.001031
0	0	0	0	0	0	3.10E-05	0	0	0	0	2.50E-05	0
0.011835	0.004628	0.006751	0.017096	0.04282	0.010034	0.021948	0.010889	0.019491	0.006362	0.014751	0.006915	0.062112

H118	H054	H143	H099	H083	H045	H059	H032	H055	H131	H086	H015	H107
0.168937	0.167084	0.166864	0.161958	0.158337	0.157414	0.145427	0.145024	0.144393	0.144242	0.141211	0.13814	0.135437
0.142442	0.017555	0.084362	0.065606	0.28692	0.045907	0.088808	0.014735	0.032544	0.118848	0.292432	0.208137	0.138725
0.062097	0.059388	0.079036	0.068365	0.05595	0.26645	0.036309	0.046344	0.05904	0.089404	0.046667	0.037957	0.094297
0.040696	0.041214	0.0623	0.032993	0.043688	0.163021	0.016662	0.074021	0.005507	0.048015	0.032584	0.077463	0.042571
0.011612	0.025803	0.026031	0.032242	0.021979	0.036545	0.002457	0.091519	0	0.198801	0.021781	0.047087	0.012867
0.094511	0.08329	0.141628	0.037349	0.081545	0.036705	0.029453	0.08672	0.007702	0.036486	0.08098	0.128647	0.042959
0.10524	0.030778	0.054795	0.231605	0.035411	0.003371	0.006042	0.042133	0.038126	0.043108	0.078249	0.069166	0.051568
0.133178	0.06392	0.1677	0.025213	0.084214	0.018236	0.049003	0.017047	0.006347	0.03284	0.077299	0.07544	0.06709
0.053429	0.019077	0.03256	0.048605	0.079464	0.00925	0.030746	0.01097	0.025084	0.018597	0.022013	0.04392	0.081607
0.007418	0.007242	0.012163	0.014083	0.012756	0.01189	0.010701	0.033551	0.008727	0.023566	0.012638	0.008127	0.022466
0.007396	0.007007	0.006115	0.003803	0.010929	0.056801	0.006235	0.004893	0.001484	0.010187	0.009868	0.005873	0.044178
0.009892	0.012396	0.003625	0.010845	0.012052	0.020041	8.50E-05	0.0196	7.20E-05	0.004648	0.024631	0.026967	0.004601
0.009754	0.007249	0.004919	0.006475	0.006391	0.01096	0.005415	0.003947	0.015956	0.004113	0.00353	0.00186	0.021001
0.007033	0.005508	0.007678	0.001841	0.011054	0.004526	0.005638	0.011501	0	0.0058	0.011758	0.018557	0.005612
0.005967	0.001473	0.00548	0.012458	0.00736	9.40E-05	8.20E-05	0.006387	0	0.015228	0.014773	0.004929	0.002539
0.011252	0.004584	0.004109	0.002854	0.007535	0.01004	0.00586	0.001287	0.002804	0.004629	0.008901	0.019336	0.030854
0.005327	0.016397	0.003808	0.030779	0.00422	0.015319	0.001238	0.011383	0.001944	0.010023	0.013562	0.011861	0.008498
0	5.80E-04	8.30E-05	0.002015	5.50E-05	6.06E-04	0	2.24E-04	7.60E-05	2.14E-04	1.94E-04	2.10E-05	0.001324
0	0	3.80E-05	2.69E-04	0	2.17E-04	0	1.60E-05	0	0	0	0	0
7.94E-04	0	0.002074	0.003006	1.09E-04	1.61E-04	0	0.001954	0.010155	3.79E-04	4.66E-04	2.48E-04	0.003482
0.007173	0.00109	2.11E-04	0.018326	0.001349	7.04E-04	0	0.002827	0.012472	0.002726	0.001423	3.43E-04	0.00773
0.028447	0.00333	0.016481	0.011494	0.006092	1.17E-04	0.004479	0.006739	0	0.014177	0.010049	0.010715	0.00147
0.004111	0.007571	0.00669	0.00294	0.006637	0.003624	0.002784	0.003005	0	0.002686	0.004446	0.006772	0.003303
0.005853	0.006988	0.00546	0.002434	0.003277	7.88E-04	1.77E-04	0.002241	0.005261	0.001463	0.005687	0.004196	0.001291
7.51E-04	1.69E-04	0.004002	0.007309	2.88E-04	0.002594	0	0.018367	0.001303	0.024389	0.001014	0.003276	0.002813
0.006548	0.052238	1.26E-04	0.003733	0.001833	7.87E-04	0.098671	0.003643	0.099892	0.001041	1.11E-04	2.70E-05	0.013205
0.005508	0.002921	0.001886	0.003741	0.003448	3.76E-04	0.003885	0.003123	8.89E-04	0.00189	0.005339	0.002092	0.003139
0.001149	0.001428	0.006555	3.12E-04	0.008861	0.004259	0.002015	9.57E-04	0	0.004088	0.001794	0.005349	0.002462
5.40E-04	0.001524	0.001063	0.008769	1.70E-04	0.005095	5.95E-04	0.001775	8.75E-04	0.005459	0	7.15E-04	0.001006
0.003904	0.008252	0.005069	0.014704	0.002255	2.67E-04	0.00219	0.010345	0.003738	0.005629	0.004511	0.003052	0.001111
0.002222	0.015337	8.75E-04	0.051089	0.001022	0.005515	0.018855	0.001667	0.016364	0.007038	0.001006	1.02E-04	0.037322
1.08E-04	5.82E-04	0.001899	0.004032	1.05E-04	3.10E-05	0	6.20E-04	7.80E-05	0.005003	0.001848	0.001832	7.60E-05
0.003182	0.003347	0.009618	0.001031	0.001622	6.61E-04	0	0.001603	4.50E-04	0.001844	0.004186	0.002024	5.69E-04
6.19E-04	9.88E-04	0.001776	0.00254	5.63E-04	0.003529	4.89E-04	0.001074	0	0.006068	0.001542	0.001481	9.94E-04
0.006432	0.003389	0.001932	0.012288	0.010751	0.001085	8.00E-05	0.009992	7.86E-04	0.004738	0.002483	7.58E-04	0.002868
4.19E-04	0.003155	0	3.07E-04	0	0	0.004849	0.060779	0.00563	7.00E-05	0	2.40E-05	8.50E-04
0.002924	0.003671	0.011408	0.004297	0.002458	0.001715	0	0.029016	0	0.005009	0.003974	0.002749	0.003694
0.001771	0.001527	8.73E-04	7.60E-04	0.001844	0.005573	0	0.0597	1.57E-04	0.003967	0.001276	0.002707	0.001317
0.001302	0.009697	0.007333	9.58E-04	0.001808	0.005875	4.37E-04	0.002904	0	0.005414	0.004177	1.94E-04	5.58E-04
0.003539	0.034226	0	0.002452	3.10E-04	5.06E-04	0.061744	3.29E-04	0.060609	8.80E-04	2.90E-05	1.26E-04	0.010557
7.14E-04	0.008204	0.001505	8.50E-04	3.52E-04	4.31E-04	0.015761	0.01855	0.017018	2.51E-04	2.95E-04	4.60E-05	0.002472
3.55E-04	2.32E-04	3.75E-04	4.25E-04	0.001155	0.005303	0	1.00E-04	0	4.35E-04	0.002128	2.94E-04	4.90E-05
0.003153	0.00154	2.41E-04	2.60E-05	5.90E-05	2.91E-04	0	0.006624	0	0.002186	0.00275	0.00904	0.002123
3.83E-04	0.00187	0.001257	0.001537	6.60E-05	1.81E-04	0	0.00121	0.008153	2.08E-04	1.20E-04	4.60E-05	7.53E-04
0.001355	0.006495	0.004713	0.001463	0.001875	0.001841	0.004816	9.08E-04	0	0.002584	0.00842	5.44E-04	0.001174
0.001414	0	8.44E-04	0.00121	8.17E-04	0.001831	0.001084	0.00113	0	0.001055	0.002153	7.07E-04	0.003435
8.93E-04	0.018927	4.60E-05	0.00211	1.60E-05	1.34E-04	0.059193	1.46E-04	0.074774	8.77E-04	3.10E-05	0	0.011321
3.56E-04	0.002399	7.39E-04	6.86E-04	0.003571	6.79E-04	0	0.007289	0	1.36E-04	0.003334	0.003442	0
5.60E-05	3.65E-04	1.66E-04	2.40E-04	2.59E-04	0.001724	0	5.80E-05	0.001524	0.005256	2.90E-04	0.003411	4.59E-04
3.08E-04	0	2.63E-04	0.00171	8.50E-05	5.69E-04	0	3.20E-05	0	2.42E-04	6.00E-05	0	0
9.53E-04	0.006208	0.011244	2.20E-05	0.002262	0.004197	0	3.66E-04	0	0.002095	0.004034	1.98E-04	0
2.39E-04	0.004128	3.49E-04	7.26E-04	3.76E-04	0.002272	9.31E-04	0.019617	0.001679	0.005973	1.72E-04	3.13E-04	0.005942
5.30E-04	0.006979	0	4.17E-04	2.30E-05	1.22E-04	0.009972	0.011663	0.010794	1.39E-04	0	1.70E-05	0.00148
0.001588	0.017038	0	9.28E-04	1.00E-04	3.11E-04	0.027804	1.44E-04	0.029295	3.22E-04	2.50E-05	0	0.004255
1.87E-04	0.00296	5.49E-04	6.84E-04	2.22E-04	0.001485	0	0.009437	0	0.002143	2.79E-04	5.23E-04	3.35E-04
4.72E-04	0.009924	0	0.002903	7.10E-05	2.94E-04	0.018903	2.52E-04	0.02409	7.00E-04	5.70E-05	1.80E-05	0.005694

0.002713	0.001918	7.50E-04	0.001341	8.70E-05	3.01E-04	0	0.005257	0.001934	0.001872	2.73E-04	1.80E-05	6.01E-04
5.21E-04	0.010582	0	0.001739	0	0	0.021131	1.54E-04	0.02188	4.26E-04	0	2.40E-05	0.004156
5.37E-04	0.005041	0	3.23E-04	2.00E-05	9.00E-05	0.011102	5.40E-05	0.01444	0.002161	0	0	0.001272
0	4.24E-04	3.80E-05	7.20E-05	2.50E-05	0.003331	0	9.59E-04	0	3.05E-04	1.96E-04	5.00E-05	0
5.20E-05	2.49E-04	0	0	1.40E-05	0.006617	6.52E-04	0.002562	0.001031	1.70E-05	2.10E-05	0	2.15E-04
5.47E-04	0.006249	0	3.59E-04	5.30E-05	6.45E-04	0.010716	1.67E-04	0.012499	1.58E-04	1.46E-04	7.40E-05	0.001182
0	0	0	0	0	0.024755	0	0	0	0	0	0	0
4.98E-04	8.05E-04	3.90E-05	6.74E-04	8.36E-04	0	0.001906	1.60E-04	0	0	0.001128	8.86E-04	0
0	0	1.86E-04	0	8.50E-05	0	0	0	0	0	0	1.50E-05	0
0	0	1.29E-04	0	9.80E-05	0	0	0	0	0	0	3.20E-05	0
6.80E-04	0.006067	0	3.22E-04	4.30E-05	9.00E-05	0.010721	9.90E-05	0.012522	1.49E-04	0	0	0.001596
0	0	0	0	0	2.04E-04	0	5.50E-05	0	0	0	0	0
3.30E-04	7.39E-04	4.90E-05	4.59E-04	1.25E-04	1.06E-04	0	4.70E-05	0	3.48E-04	1.67E-04	7.75E-04	4.25E-04
0	0	0	0	0	0	0	0	0	0	0	0	0
6.56E-04	0.004703	0	2.32E-04	4.30E-05	1.97E-04	0.008912	6.50E-05	0.013047	1.47E-04	0	2.30E-05	0.002398
0	0	3.90E-04	0	1.00E-04	0	0	0	0	0	9.80E-05	1.90E-05	0
0	0	3.13E-04	4.45E-04	3.10E-05	4.05E-04	0	4.81E-04	0	0.004284	0	0	4.67E-04
5.80E-05	0	3.90E-05	0	0	4.30E-04	0	0	9.71E-04	0.021239	0	0	5.26E-04
4.19E-04	0.004618	0	3.24E-04	0	5.60E-05	0.007308	2.60E-05	0.009151	9.40E-05	8.90E-05	0	0.001952
6.50E-05	0	1.30E-04	0	8.80E-05	0	0	7.80E-05	0	0	1.28E-04	1.80E-05	0
0	0	0	0	0	0	0	0.001251	0	0	0	0	0
0	7.60E-05	0	0	0	0	0	0.002236	7.30E-05	0	0	0	0
0	0.0133	0	0	0	0.002442	0	0	0	0	0	0	0
0	0	0	0	0	0	0	0	0	0	0	0	0
3.76E-04	0.002935	0	1.20E-04	5.20E-05	5.80E-05	0.005644	2.60E-05	0.005684	4.80E-05	0	0	0.001153
0	0	0	0	0	0	0	0	0	0	0	0	0
2.80E-05	0.002001	8.20E-05	0.001803	0	3.70E-05	0	5.70E-05	0	3.60E-04	1.19E-04	0	5.48E-04
0	7.30E-05	0	2.60E-05	0	0.001177	0	0	0	1.40E-05	0	3.10E-05	0
0	0	0	0	0	0	0	9.18E-04	0	0	0	0	0
0	0	0	0	0	0	0	1.31E-04	0	0.012197	0	0	4.90E-05
0.016087	0.120976	0.026939	0.029944	0.012329	0.026739	0.142033	0.063729	0.170976	0.018872	0.025055	0.007166	0.035957

H064	H111	H088	H047	H124	H046	H139	H048	H070	H009	H072	H020	H002
0.13425	0.131624	0.12816	0.123058	0.122772	0.122242	0.121282	0.120264	0.119093	0.118528	0.117706	0.117074	0.115849
0.345139	0.136119	0.248758	0.210789	0.187886	0.323649	0.032603	0.36266	0.267999	0.100569	0.130936	0.273621	0.260728
0.050485	0.076273	0.109888	0.084491	0.130718	0.050405	0.161754	0.046203	0.115853	0.032794	0.032271	0.057623	0.044779
0.050659	0.046474	0.04709	0.04605	0.056555	0.036164	0.25661	0.017976	0.034931	0.044313	0.041537	0.052795	0.118189
0.029592	0.009332	0.014179	0.008595	0.02167	0.01314	0.008416	0.002382	0.107193	0.044017	0.046115	0.021299	0.020765
0.113744	0.052997	0.061768	0.104442	0.039668	0.103208	0.017501	0.042685	0.02381	0.175058	0.201216	0.06845	0.047639
0.03125	0.225211	0.063543	0.036362	0.131148	0.028738	0.028936	0.014511	0.016177	0.102344	0.082771	0.09973	0.094852
0.018954	0.091241	0.091051	0.117037	0.038318	0.152875	0.020655	0.064092	0.018037	0.095072	0.051002	0.045118	0.058372
0.072573	0.074487	0.04085	0.046214	0.070711	0.052668	0.130326	0.141023	0.145486	0.023675	0.033237	0.031814	0.038134
0.010792	0.013534	0.030186	0.007145	0.016881	0.009162	0.076562	0.014074	0.0166	0.008968	0.007378	0.040797	0.062563
0.007157	0.007996	0.012285	0.007007	0.010887	0.005752	0.019733	0.082101	0.016799	0.005217	0.003582	0.037452	0.004044
0.046541	0.003413	0.004353	0.026372	0.014138	0.013696	0.00538	0.011455	0.004412	0.055682	0.054107	0.008977	0.016028
0.001666	0.013149	0.007342	0.015258	0.01059	0.007224	0.015881	0.008842	0.024465	0.004003	0.006078	0.005272	7.29E-04
0.015518	0.004884	0.004281	0.008676	0.00282	0.006945	0.004495	0.004938	0.006156	0.006906	0.013166	0.009555	0.005308
0.00103	0.008786	0.008874	6.34E-04	0.006641	0.006832	0.016319	0.001799	0.002619	0.01324	0.00527	0.016778	0.011584
0.007638	0.010599	0.006478	0.009435	0.014851	0.009804	0.008837	0.006887	0.004029	0.003437	0.00193	0.005577	0.002688
0.00743	0.001813	0.005325	0.004829	0.012668	0.002058	0.004516	0.002461	0.008794	0.018877	0.014624	0.011239	0.016384
6.80E-05	1.19E-04	2.29E-04	2.48E-04	2.05E-04	6.50E-05	3.23E-04	1.97E-04	3.90E-05	2.60E-05	0	1.06E-04	1.02E-04
0	2.80E-05	1.61E-04	3.60E-05	0	0	0	0	3.10E-05	0	0.020868	0	0
0.007757	0.021302	0.022043	0.030915	0.003553	7.94E-04	0.005757	4.10E-05	3.70E-05	1.10E-04	0.002343	0.002603	0.001527
2.10E-05	0.001475	0.0019	3.39E-04	0.002203	4.49E-04	0.011728	0.001622	0.006246	0.00326	5.61E-04	0.001295	0.001434
1.11E-04	0.011187	1.67E-04	0.017913	0.002316	0.017943	1.50E-04	0.001364	2.03E-04	0.015624	0.013496	0.002253	0.010343
0.004979	0.002339	0.004011	0.011815	0.002842	9.07E-04	0.004309	0.002184	0.003436	0.010758	0.003831	0.002414	0.003327
0.002783	0.00477	0.003677	0.004188	0.002009	0.004169	4.03E-04	0.002483	9.91E-04	0.003885	0.00562	0.002297	0.001977
0.001471	3.66E-04	0.001449	6.00E-04	0.002163	1.65E-04	3.06E-04	4.76E-04	0.009853	0.001989	0.001858	2.23E-04	5.36E-04
2.34E-04	5.56E-04	0.002143	0.001873	0.001505	2.37E-04	2.94E-04	7.20E-05	1.09E-04	0.011136	1.12E-04	0.001293	0.001611
0.001308	0.007009	0.00302	0.002635	0.002761	0.001536	0.002461	0.002852	0.002314	0.003965	0.003269	0.004393	0.002453
0.007065	0.001478	0.002458	0.006928	0.002209	0.002009	0.0011	4.99E-04	0.001955	0.002157	0.005169	9.07E-04	0.00145
6.16E-04	1.28E-04	0.001318	0	0.003192	2.51E-04	0.004521	8.42E-04	0.004077	4.90E-05	4.48E-04	0.001557	1.86E-04
5.68E-04	0.002162	0.001141	0.006644	0.004696	0.001534	0.00218	5.65E-04	0.002254	0.00597	0.007796	0.006332	0.00476
6.98E-04	0.001073	0.002112	0.003552	0.003167	0.001074	0.003993	0.001186	8.57E-04	0.004798	2.11E-04	0.001523	0.002807
0	1.31E-04	0.00118	3.40E-05	0.001115	3.00E-05	0	5.40E-05	0.004033	0.001154	0.007499	8.77E-04	0.001526
0.003036	0.003961	0.001752	0.002578	0.001666	0.002283	0.00158	0.002799	6.46E-04	0.00184	0.001283	0.001883	0.001675
7.17E-04	0.001197	0.002456	6.55E-04	0.001412	6.02E-04	0.008046	9.36E-04	6.66E-04	0.001073	0.001535	0.00313	0.002636
3.10E-04	0.005323	0.004707	0.002665	0.022905	7.32E-04	2.25E-04	0.008163	0.003193	0.001983	0.008929	0.003466	0.00384
0	3.90E-05	0	4.90E-04	9.30E-05	6.40E-05	0	9.00E-05	0	7.16E-04	0	7.10E-05	1.40E-04
0.001358	2.73E-04	0.002547	0.00119	7.33E-04	5.81E-04	7.21E-04	0.001121	6.96E-04	0.005102	0.003715	0.00238	0.001044
1.62E-04	5.53E-04	8.87E-04	8.16E-04	8.54E-04	4.78E-04	6.00E-05	0.001678	0.003362	0.001236	4.13E-04	0.003978	6.55E-04
0.001463	0.002213	0.005792	0.001325	0.007088	0.001236	0.003351	0.002267	7.38E-04	4.30E-04	0.002171	0.00199	0.003825
4.90E-05	2.95E-04	0	0.002915	8.75E-04	6.70E-05	3.00E-05	0	7.00E-05	0.006889	1.06E-04	9.06E-04	0.001066
1.07E-04	7.90E-05	2.00E-05	6.32E-04	3.78E-04	3.10E-05	3.80E-05	7.50E-05	0	0.002066	3.80E-05	1.49E-04	3.56E-04
0.001423	3.64E-04	2.20E-04	3.51E-04	0.002276	2.00E-04	5.23E-04	0.001865	7.00E-04	0.00145	5.92E-04	2.22E-04	7.94E-04
6.40E-05	0.004136	3.88E-04	0.005672	0.001549	0.00258	0	6.67E-04	1.46E-04	0.002991	0.006506	0.00399	1.24E-04
1.24E-04	3.52E-04	9.32E-04	1.82E-04	0.004096	6.55E-04	0.0021	0.001382	0.010753	4.32E-04	0.002877	0.001607	0
0.003568	0.002182	0.004119	0.00521	8.17E-04	0.00334	8.30E-05	0.001476	4.40E-05	0.003443	0.004369	0.0022	0.001034
9.85E-04	0.004636	4.91E-04	0.001806	0.00278	0.001651	8.00E-04	0.001521	7.51E-04	0.001608	0.002096	0.001388	0.001042
2.40E-05	3.44E-04	4.30E-05	0	0.001157	2.09E-04	0	0	0	3.53E-04	3.50E-05	1.96E-04	3.98E-04
0.004952	2.70E-04	5.53E-04	0.001716	0.001151	0.002225	4.70E-05	4.80E-05	4.20E-05	0.008943	0.010357	0.001184	0.001122
3.10E-04	2.70E-05	4.35E-04	1.39E-04	8.60E-05	0	6.40E-05	1.51E-04	3.77E-04	1.40E-04	0	0.001259	0.003862
0	7.93E-04	9.50E-05	0	0.001291	1.61E-04	0	8.40E-05	2.36E-04	4.29E-04	8.20E-05	2.52E-04	0
0.001122	5.27E-04	0.009027	0.001946	0.001503	8.78E-04	0.001845	0.002089	0	4.39E-04	0.00732	4.09E-04	0.001405
1.31E-04	3.13E-04	0.003838	0.001282	0.001258	4.61E-04	2.70E-05	8.40E-04	1.36E-04	3.10E-05	5.71E-04	1.83E-04	0.001538
0	1.50E-05	0	2.97E-04	1.54E-04	0	0	0	0	0.001337	0	7.20E-05	5.30E-05
0	1.79E-04	2.50E-05	4.37E-04	3.00E-04	1.73E-04	0	0	0	0.003582	0	3.89E-04	4.28E-04
0	1.02E-04	8.20E-04	5.69E-04	7.06E-04	2.07E-04	6.40E-05	4.43E-04	0.00116	9.60E-05	0	0.022574	4.37E-04
1.05E-04	2.94E-04	0	2.50E-04	6.53E-04	7.70E-05	1.77E-04	5.90E-05	7.60E-05	0.00113	4.40E-05	3.03E-04	1.47E-04

5.30E-05	1.55E-04	0.001308	6.17E-04	1.23E-04	0	9.30E-05	9.70E-04	7.59E-04	0	0.015602	3.53E-04	1.45E-04
2.30E-05	1.96E-04	2.70E-05	0	1.62E-04	0	0	0	0	6.44E-04	0	2.16E-04	6.20E-05
2.50E-05	5.90E-05	0	0.001636	1.36E-04	6.10E-05	0	2.60E-05	3.90E-05	0.001609	0	1.29E-04	2.00E-04
3.00E-05	0	5.60E-05	8.70E-05	5.00E-05	0	7.79E-04	4.70E-05	1.16E-04	0	0	2.52E-04	8.00E-05
0	2.30E-05	8.17E-04	1.15E-04	0	0	0	0	0	5.90E-05	0	3.60E-05	0
2.60E-05	1.20E-04	5.99E-04	4.78E-04	3.90E-04	0	3.33E-04	0	0	0.001401	3.50E-05	1.61E-04	4.75E-04
0	0	0	0	0	0	0	2.20E-05	0	0	0	0	0
0	9.49E-04	0	0	1.95E-04	1.68E-04	0	0	0	5.82E-04	4.44E-04	1.29E-04	7.44E-04
6.80E-05	0	1.21E-04	0	0	0	1.06E-04	6.48E-04	2.20E-04	0	0	0	0
6.80E-05	0	2.41E-04	0	0	0	2.14E-04	7.70E-05	1.74E-04	0	3.40E-05	0	0
2.80E-05	0	0	2.23E-04	2.61E-04	7.30E-05	0	5.70E-05	0	0.001387	0	9.00E-05	2.19E-04
0	2.80E-05	2.80E-04	3.20E-05	0	0	0	0	0	0	0	1.80E-05	0
4.20E-04	2.20E-05	9.83E-04	7.70E-05	2.10E-04	6.80E-05	0	7.60E-05	0.001105	3.00E-05	1.36E-04	7.60E-05	1.71E-04
0	0	0	0	0	0	0	0	0	0	0	0	0
0	8.90E-05	0	2.59E-04	6.70E-05	0	0	0	4.00E-05	8.99E-04	0	1.39E-04	1.67E-04
7.50E-05	0	2.40E-04	7.80E-05	0	1.21E-04	1.25E-04	2.15E-04	2.71E-04	0	4.20E-05	0	0
0	3.40E-05	4.70E-05	1.12E-04	0	0	0	1.11E-04	0	0	2.02E-04	5.10E-05	2.90E-05
0	0	6.80E-05	3.50E-05	3.11E-04	0	0	2.90E-05	0	0	0	4.00E-05	0
2.20E-05	6.20E-05	0	1.81E-04	2.24E-04	0	0	0	0	0.001146	0.001234	1.17E-04	1.62E-04
0	0	2.50E-05	0	4.25E-04	3.20E-05	0	1.61E-04	0	5.30E-04	0.002091	5.70E-05	5.17E-04
0	0	0	3.00E-05	0	0	0	0	0	5.10E-05	0	0	0
0	0	0	0	0	2.70E-05	0	0	0	0	0	0	0
0	0	0	0	0.004323	0	0	7.20E-05	0	0	1.01E-04	0	0.003987
0	0	0	0	0	0	0	0	0	0	0	0	0
0	9.70E-05	0	3.74E-04	1.04E-04	0	2.20E-05	0	0	5.25E-04	0	7.20E-05	1.08E-04
0	0	0	0	0	0	0	0	0	0	0	0	0
2.00E-05	6.60E-05	5.10E-05	1.47E-04	1.47E-04	0	1.96E-04	0	3.30E-05	1.86E-04	0	1.69E-04	2.62E-04
0	0	0	3.90E-05	0	0	3.80E-05	0	0	0	0	5.80E-05	5.60E-05
0	0	0	0	0	0	0	0	0	0	0	0	0
0	5.00E-05	0	0	0	0	0	0	0	0	0	0	0
0.007035	0.007528	0.02457	0.018243	0.014233	0.004834	0.010982	0.010945	0.004563	0.025631	0.011033	0.012412	0.012321

H066	H097	H109	H018	H122	H062	H051	H108	H061	H024	H082	H005	H031
0.115221	0.114119	0.112792	0.109225	0.10856	0.107937	0.105724	0.102996	0.102522	0.101634	0.100145	0.099678	0.098749
0.041486	0.369574	0.273048	0.180842	0.101344	0.188159	0.091425	0.216064	0.263688	0.488094	0.208525	0.141492	0.091955
0.271699	0.014899	0.075171	0.039825	0.163238	0.077601	0.092357	0.11903	0.054136	0.015647	0.028924	0.028297	0.251365
0.048202	0.046641	0.051461	0.052848	0.03138	0.066786	0.044681	0.04186	0.105453	0.069119	0.0564	0.068492	0.096348
0.017804	0.04029	0.014622	0.02185	0.014101	0.020617	0.008666	0.027975	0.017984	0.020667	0.079527	0.072731	0.039983
0.044556	0.062428	0.072965	0.094071	0.059785	0.063244	0.081098	0.037419	0.16939	0.001389	0.111256	0.160052	0.03139
0.038435	0.060577	0.116353	0.103394	0.042491	0.029405	0.153502	0.122985	0.039917	0.049276	0.120423	0.061586	0.043494
0.049485	0.038313	0.070548	0.149043	0.022354	0.053549	0.083556	0.05946	0.122167	0.00589	0.051305	0.030584	0.045058
0.063635	0.042847	0.043598	0.062139	0.301838	0.050045	0.114993	0.08713	0.002498	0.006826	0.020236	0.005057	0.029432
0.0136	0.018942	0.019252	0.012761	0.008267	0.030739	0.011648	0.01717	0.003389	0.006404	0.0163	0.041614	0.029359
0.025458	0.012706	0.008375	0.007906	0.011268	0.028024	0.005727	0.005344	0.010774	0.00158	0.004273	0.019938	0.018182
0.003104	0.047506	0.009706	0.026901	0.006803	0.007101	0.016961	0.014963	0.018145	0.006816	0.056939	0.06841	0.004573
0.005816	5.89E-04	0.013086	0.007835	0.009234	0.007104	0.007644	0.010689	0.006672	5.92E-04	0.001424	0.008613	0.016384
0.001793	0.009559	0.006195	0.006662	0.00379	0.005719	0.005796	0.00652	0.012952	0.004079	0.008725	0.018353	0.002047
0.001808	0.019213	0.004782	0.01878	0.003224	0.017622	0.007558	0.012669	4.87E-04	0.005678	0.019884	0.011687	0.002119
0.006886	0.002139	0.015574	0.007783	0.003416	0.026314	0.003367	0.005375	0.007485	0.004488	0.004061	0.007756	0.013242
0.006844	0.007182	0.002689	0.005479	0.008082	0.005765	0.012042	0.009979	0.003471	0.002457	0.010721	0.02308	0.002261
0.004003	0	4.00E-05	2.50E-05	3.08E-04	0.001736	1.06E-04	2.34E-04	0	0	4.10E-05	5.80E-05	4.01E-04
0	0	0.023669	1.32E-04	0.013505	0	3.44E-04	0.013993	0	0.003285	0	0	6.30E-05
0.002909	0.001465	0.005175	3.50E-05	0.004542	0.008396	0.004554	0.004812	1.04E-04	0.001068	8.68E-04	0	3.41E-04
0.001819	0.002849	5.73E-04	0.003491	0.001429	0.0025	0.005731	0.001748	0.007082	0.0019	0.00593	6.96E-04	0.048136
6.90E-04	0.012508	0.015659	0.014018	0.004057	0.00294	0.009507	0.011724	0.004635	7.11E-04	0.005977	0.001737	2.84E-04
0.003237	0.008651	0.001194	0.003629	0.003288	0.00348	0.00269	0.00391	0.007478	0.0021	0.0029	0.013451	0.001888
9.07E-04	0.002079	0.004953	0.007825	0.003086	0.007367	0.004835	0.003347	0.004183	0	0.006917	0.006354	0.004957
0.001157	0.012607	8.00E-05	0.001102	6.19E-04	0.001559	6.41E-04	0.00172	8.50E-05	0.001007	0.008255	0.002054	0.001023
0.012886	1.07E-04	1.33E-04	0.002316	0.001017	0.008673	0.001573	6.05E-04	2.41E-04	0.002343	4.14E-04	9.50E-05	0.001677
7.69E-04	0.003645	0.003557	0.002607	0.001598	0.002673	0.003543	0.002186	0.002581	0.003954	0.003835	0.023792	0.001722
3.23E-04	8.18E-04	0.001475	0.001451	0.003181	5.50E-04	0.001994	0.001184	0	0	8.57E-04	0.023427	0.002172
0.001408	3.22E-04	4.20E-05	1.37E-04	0.003692	0.004029	5.95E-04	4.00E-05	3.37E-04	4.80E-05	6.50E-05	2.52E-04	1.77E-04
0.002463	0.005428	0.00102	0.004697	0.002525	0.004923	0.004519	0.005112	0.005288	0.002827	0.00395	0.012576	0.004835
0.055561	2.31E-04	8.94E-04	0.001451	0.009109	0.047312	0.0019	0.003693	3.16E-04	3.80E-05	0.001051	4.95E-04	0.004006
0.001554	0.008595	1.52E-04	7.89E-04	0.00159	1.59E-04	5.81E-04	0.007129	4.00E-05	0.001119	8.39E-04	0.005872	1.37E-04
6.34E-04	0.001878	0.001715	0.001938	0.002335	0.001161	0.004264	0.001292	0.00275	0.001731	0.001844	0.00207	6.39E-04
8.85E-04	0.001795	6.79E-04	0.001541	7.32E-04	0.002139	0.002562	7.82E-04	8.88E-04	0.002567	0.001449	0.003801	0.001844
0.009009	0.010344	0.002177	9.10E-04	0.013501	0.004525	0.006023	0.010963	0	0.002626	0.002996	6.40E-05	0.003298
8.03E-04	0	0	1.09E-04	9.90E-05	7.01E-04	1.29E-04	2.80E-05	0	0.041313	0	2.90E-05	0.012711
0.00278	0.001365	0.002229	6.78E-04	0.001182	0.001964	0.003085	0.001501	0.001926	0	0.006008	0.017364	6.64E-04
0.007832	2.03E-04	0.001462	0.001509	0.001684	0.002698	6.28E-04	7.63E-04	6.70E-05	0.001039	0.013775	5.70E-05	0.005762
0.009553	5.32E-04	5.03E-04	0.001045	0.00262	0.00428	0.00591	0.002273	5.73E-04	3.70E-05	7.66E-04	9.02E-04	0.004941
0.008121	0	5.60E-05	0.001703	3.84E-04	0.006317	9.78E-04	4.26E-04	0	2.80E-05	2.55E-04	4.30E-05	5.91E-04
0.002335	0	1.83E-04	4.89E-04	8.50E-05	0.001655	3.63E-04	2.06E-04	1.38E-04	0.036715	7.80E-05	4.20E-05	0.008025
2.20E-04	6.66E-04	2.55E-04	4.40E-04	4.89E-04	8.34E-04	0.001676	4.83E-04	8.01E-04	6.80E-04	0.001617	9.10E-05	7.32E-04
8.42E-04	8.53E-04	0.003286	7.08E-04	1.05E-04	0.006125	0.00426	0.002589	4.80E-05	0.003691	2.30E-05	2.20E-05	7.42E-04
0	8.30E-05	6.33E-04	0.001776	6.70E-05	0	0.002388	7.60E-04	4.21E-04	9.40E-05	0	6.60E-05	0.005619
0	3.74E-04	0.003316	0.009595	0.001288	0.00179	0.007817	0.001284	6.77E-04	0	0.001284	0.001038	0.002459
4.62E-04	4.58E-04	7.25E-04	0.001564	0.00128	0.001735	8.84E-04	0.001869	3.76E-04	3.60E-05	8.54E-04	0.001799	0.006571
0.007863	0	2.90E-05	1.70E-04	9.21E-04	0.00528	5.04E-04	5.96E-04	0	0	1.32E-04	2.30E-05	1.28E-04
0	0.001358	0.001577	0.00188	9.15E-04	4.72E-04	0.001888	9.54E-04	0.002874	7.50E-05	0.003892	0.005183	1.28E-04
0.004165	0	3.76E-04	5.75E-04	1.48E-04	1.84E-04	3.10E-05	4.95E-04	1.43E-04	3.58E-04	0.009649	5.80E-05	3.32E-04
0	0	8.44E-04	5.20E-04	0	0	0.001631	2.80E-05	4.80E-05	4.80E-05	0	0	0.009083
0.003447	3.05E-04	0.002021	0	0.001985	0.001267	0.003426	1.75E-04	0.005024	1.28E-04	2.79E-04	2.43E-04	0.001254
0.002854	5.60E-05	6.89E-04	1.28E-04	5.09E-04	7.17E-04	0.002453	4.52E-04	4.60E-05	0	7.59E-04	5.87E-04	0.001439
0.001431	6.90E-05	0	1.29E-04	9.50E-05	8.34E-04	1.70E-04	3.40E-05	0	0.023709	1.80E-05	2.10E-05	0.006601
0.003137	0	0	7.91E-04	1.85E-04	0.002113	5.16E-04	5.40E-05	0	4.10E-05	6.40E-05	2.00E-05	2.40E-05
0	0	3.01E-04	0	3.06E-04	0.001055	1.70E-04	1.78E-04	0	2.05E-04	2.54E-04	9.60E-05	0.00118
0.007363	0	5.30E-05	1.23E-04	9.40E-04	0.008906	8.54E-04	2.16E-04	0	0	7.30E-05	2.30E-05	3.28E-04

0.003274	0.003016	1.19E-04	2.76E-04	3.56E-04	4.34E-04	2.89E-04	2.06E-04	3.92E-04	5.30E-05	7.88E-04	8.40E-05	4.00E-05
0.006017	0	0	1.03E-04	5.83E-04	0.003124	3.35E-04	1.99E-04	0	0	3.20E-05	2.60E-05	1.70E-04
0.00161	0	6.70E-05	2.36E-04	1.43E-04	0.001183	2.35E-04	5.30E-05	0	3.90E-05	0	1.90E-05	8.30E-05
7.06E-04	0	7.70E-05	0	1.40E-05	3.62E-04	2.20E-05	1.13E-04	0	0	0.002396	1.09E-04	9.90E-05
4.88E-04	0	0	0	3.60E-05	1.50E-05	0	0	0	0.002836	0	0	4.39E-04
0.001176	3.30E-05	2.30E-05	3.17E-04	3.10E-05	0.001326	4.88E-04	5.80E-05	0	0.003885	4.20E-05	6.00E-05	4.56E-04
0	0	4.84E-04	0	0	0.005492	0.023626	0	0	0	0	0	0
0	0.001533	2.48E-04	0.002965	2.06E-04	9.52E-04	3.14E-04	3.24E-04	5.96E-04	0	5.34E-04	2.57E-04	5.30E-05
0	2.13E-04	7.30E-05	0	0	0	0	0	2.21E-04	0	0	3.20E-05	0
0	1.37E-04	8.70E-05	0	0	0	0	0	0	5.80E-05	0	4.90E-05	0
0.001129	3.30E-05	5.50E-05	2.65E-04	4.00E-05	0.001103	1.14E-04	8.30E-05	8.50E-05	0	5.10E-05	0	9.90E-05
0.002218	0	4.80E-05	0	0	1.89E-04	5.00E-05	3.90E-05	0	0	0	0	3.62E-04
6.60E-05	6.83E-04	0	0	3.02E-04	4.61E-04	1.57E-04	2.45E-04	2.56E-04	3.50E-05	0	1.16E-04	7.10E-05
0	0	0	0	0	0	0	0	0	0	0	0	0
0.00141	0	3.90E-05	1.69E-04	8.30E-05	9.99E-04	2.00E-04	9.40E-05	0	0	1.80E-05	0	1.17E-04
0	2.29E-04	1.99E-04	0	0	0	0	0	6.66E-04	7.00E-05	0	0	0
2.03E-04	0	0	0	4.60E-05	0	3.26E-04	3.00E-05	0	0	4.50E-05	0	4.28E-04
6.91E-04	0	1.13E-04	0	0	0	0	0	0	0	0	0	0.001512
0.001236	0	4.20E-05	3.01E-04	8.20E-05	7.99E-04	7.10E-05	5.30E-05	0	0	5.90E-05	0	7.00E-05
1.45E-04	9.00E-05	2.40E-04	4.38E-04	0	8.50E-05	2.43E-04	3.09E-04	0	0	2.24E-04	0	3.80E-05
0	0	0	0	0	0	0	0	0	0.002485	0	0	4.42E-04
0	0	0	0	0	0	1.40E-05	0	0	0.005594	0	0	0.003323
0	0	4.90E-04	0	0	1.47E-04	0.007358	5.80E-05	0	0	0	0	0
0	0	0	0	0	0	0	0	0	0	0	0	0
8.95E-04	0	0	1.37E-04	3.70E-05	4.50E-04	7.00E-05	6.20E-05	5.20E-05	0	0	0	4.80E-05
0	0	0	0	0	0	0	0	0	0	0	0	0
0.021236	0	0	5.70E-05	5.13E-04	0.001119	2.60E-05	1.02E-04	0	0	0	2.20E-05	2.12E-04
0	0	0	0	0	5.80E-05	0	7.10E-05	0	0	0	2.80E-05	0
0	0	0	0	0	0	0	0	0	2.87E-04	0	0	1.41E-04
0	0	0	0	0	0	0	0	0	0	0	0	0.002091
0.038246	0.006865	0.005654	0.015366	0.012952	0.042922	0.022955	0.010435	0.007432	0.058501	0.008775	0.007277	0.026851

H077	H133	H025	H074	H065	H104	H137	H027	H069	H006	H110	H140	H147
0.097801	0.092944	0.092595	0.089483	0.088621	0.08588	0.085503	0.085013	0.084965	0.078021	0.077512	0.076057	0.073384
0.495116	0.21314	0.160215	0.309022	0.073029	0.098438	0.350983	0.366469	0.415921	0.382606	0.162048	0.103442	0.37773
0.039807	0.202503	0.055223	0.011874	0.111254	0.096672	0.037609	0.033561	0.025248	0.028387	0.116819	0.096533	0.032728
0.01687	0.016017	0.067857	0.020917	0.053439	0.026309	0.033439	0.015456	0.026999	0.013305	0.055183	0.09961	0.036317
0.019537	0.009374	0.018873	0.027937	0.010873	0.006666	0.036434	0.009822	0.075699	0.011673	0.026311	0.006676	0.019448
0.081373	0.05541	0.096222	0.149931	0.059148	0.058386	0.112191	0.01919	0.03625	0.042147	0.10559	0.036571	0.130731
0.073348	0.027637	0.04863	0.060382	0.168437	0.108361	0.044827	0.031505	0.05554	0.021085	0.051491	0.038645	0.044964
0.053734	0.101358	0.117521	0.071728	0.066947	0.063567	0.076783	0.064683	0.041308	0.065125	0.077062	0.104756	0.078536
0.029425	0.027919	0.081515	0.063318	0.177592	0.068625	0.048305	0.035228	0.042929	0.042121	0.070409	0.161935	0.039712
0.010134	0.016262	0.010845	0.004325	0.017376	0.018219	0.009786	0.012242	0.006317	0.009089	0.02858	0.027304	0.014646
0.007938	0.039944	0.004813	0.005263	0.015723	0.010701	0.003636	0.017518	0.0145	0.017305	0.025443	0.016707	0.013947
0.017385	0.016785	0.016993	0.095851	0.01439	0.01307	0.047208	0.023206	0.047368	0.030329	0.022343	0.011444	0.038753
3.83E-04	0.019257	0.011349	0.003168	0.010331	0.072415	0.004126	0.001931	0.001068	0.002344	0.026617	0.012604	0.002236
0.007176	0.004086	0.008283	0.008803	0.002512	0.00381	0.010907	0.00295	0.010332	0.002195	0.005179	0.006805	0.011384
0.00362	0.006507	0.017374	0.009395	0.003097	0.013184	0.002703	0.002609	0.007596	0.004427	0.004205	0.003381	0.010698
0.003058	0.012988	0.006686	0.003906	0.009112	0.003399	0.003826	0.005455	0.003833	0.006988	0.017969	0.010953	0.00802
0.005514	0.002134	0.007672	0.009467	0.004625	0.002887	0.006231	0.005611	0.011024	0.008856	0.007848	0.008449	0.007493
0	5.90E-05	5.70E-05	0	3.68E-04	7.91E-04	0	1.45E-04	3.50E-05	2.42E-04	1.78E-04	0.001101	0
6.83E-04	0.001579	0.006096	0	3.61E-04	6.91E-04	0	0	0	0.105309	0	0	5.61E-04
0.001891	0.00272	0.003531	2.01E-04	0.005274	0.074675	0.005854	3.62E-04	0.008645	0	0.004723	0.011399	0.003306
9.53E-04	0.039938	0.003415	0.002886	0.009589	0.020514	0.005683	0.006879	4.40E-04	0.012704	0.00877	0.01414	7.93E-04
0.004855	0.016112	0.008182	0.008452	0.007141	0.008283	0.008516	0.034496	0.002927	0.007593	0.006875	0.00467	0.004181
4.60E-04	0.001511	0.006613	0.001235	0.00222	0.002458	0.00545	0.001212	0.002626	0.003938	0.003591	0.005851	0.003619
0.001845	0.005011	0.005087	0.002267	9.78E-04	0.002567	0.002125	7.74E-04	9.73E-04	0.003332	0.002552	0.009449	0.004287
3.75E-04	4.68E-04	0.001605	7.13E-04	2.43E-04	3.38E-04	0.002256	5.79E-04	0.00493	0.001744	6.91E-04	0.006826	4.06E-04
1.77E-04	0.003287	0.00108	7.90E-05	0.002186	0.008344	4.40E-05	0.010897	1.04E-04	0.003783	0.001503	0.002334	0
0.001834	0.002625	0.001763	0.001514	0.002186	0.003053	0.00302	0.003905	0.001419	0.001256	0.001177	0.002319	0.002339
2.47E-04	1.95E-04	0.002205	5.88E-04	0.001096	0.00423	0.003798	5.97E-04	2.99E-04	9.50E-05	0.00395	4.49E-04	0.001245
0	3.40E-05	8.70E-05	0	0.001456	0	0.001171	9.15E-04	2.31E-04	2.80E-05	2.87E-04	4.17E-04	4.20E-05
0.0017	0.002129	0.005385	0.00233	0.002613	6.56E-04	0.002459	0.004203	0.003782	0.001444	0.002421	2.85E-04	0.004704
1.66E-04	0.003458	6.78E-04	1.16E-04	0.009352	0.046399	5.77E-04	0.002288	2.58E-04	0.006147	0.006041	0.011448	1.82E-04
7.40E-05	0	1.82E-04	0	5.61E-04	1.65E-04	0.001433	8.83E-04	0.007314	3.60E-04	3.63E-04	1.35E-04	1.41E-04
0.002032	0.004179	0.00134	5.47E-04	3.85E-04	7.66E-04	0.003425	3.04E-04	0.001179	0.001987	8.14E-04	0.003078	0.00256
1.59E-04	9.30E-05	6.01E-04	5.89E-04	7.78E-04	1.84E-04	0.002013	5.94E-04	8.60E-05	9.60E-04	8.22E-04	0.001296	5.95E-04
0.004171	0.005664	9.74E-04	8.60E-04	0.010035	9.80E-04	0.0083	3.65E-04	0.00445	0.005551	0.009855	0.007848	0.002942
4.50E-05	3.41E-04	0.024638	0	1.39E-04	3.37E-04	0	0.060239	0	1.77E-04	2.48E-04	0	0
0.001248	0.001284	0.001311	0.001427	0.001042	0.001164	0.004074	4.27E-04	0.003641	9.97E-04	0.004799	0.001203	0.001719
8.63E-04	0.009263	2.57E-04	0	0.001861	9.58E-04	4.73E-04	0.001078	0.016582	0.003314	0.008657	0.004028	4.95E-04
5.06E-04	5.35E-04	0.006185	0.001766	0.002456	0.002651	0.001468	9.80E-05	7.27E-04	0.00714	0.001072	0.017371	0.001813
3.60E-05	0.001872	6.30E-05	0	0.001428	0.004394	0	0.004715	0	0.00217	9.47E-04	2.20E-05	4.50E-05
1.24E-04	2.71E-04	0.002426	9.10E-05	3.20E-04	0.001292	5.50E-05	0.032677	6.90E-05	5.67E-04	3.24E-04	3.87E-04	1.63E-04
0.001099	5.32E-04	4.54E-04	0.003256	8.98E-04	3.72E-04	0.001192	7.37E-04	0.010646	0.004744	3.67E-04	2.30E-04	0.001815
3.84E-04	0.003771	0.030266	0.003653	0.005437	0.002928	0.002233	9.72E-04	0.002352	0.001072	0.001573	0.001984	6.78E-04
2.20E-04	9.76E-04	5.39E-04	8.10E-05	0.001963	0	1.55E-04	2.70E-05	4.40E-05	7.79E-04	0.004494	2.84E-04	1.75E-04
0.001628	0.001964	0.006501	0.002869	0.001227	0.002335	0.002463	0	2.49E-04	0.004135	0.003786	0.010211	0.002249
9.07E-04	0.0029	0.004475	0.003126	0.010565	0.004642	6.04E-04	0.001425	0.001982	0.010301	0.004073	0.003061	4.66E-04
0	1.44E-04	3.10E-05	0	0.00137	0.003429	0	0.001506	3.50E-05	2.62E-04	8.66E-04	1.07E-04	0
0.001682	2.04E-04	0.002164	0.00453	0.001183	0.001392	0.005088	0	1.46E-04	2.91E-04	0.002321	0.001714	0.003519
0	0.001049	2.23E-04	0	4.04E-04	0	6.70E-05	0	1.56E-04	7.33E-04	0.003086	0.002368	1.48E-04
3.42E-04	1.01E-04	2.78E-04	9.20E-05	0.005478	4.42E-04	6.34E-04	2.45E-04	3.40E-05	1.99E-04	0.001188	3.72E-04	1.32E-04
0.001252	4.93E-04	0.001902	0.002512	2.91E-04	7.44E-04	9.00E-04	0	4.36E-04	0.003489	8.94E-04	0.011428	0.002439
0	1.03E-04	0.001792	2.61E-04	4.77E-04	6.69E-04	1.41E-04	1.20E-04	1.93E-04	0.002317	9.31E-04	0.004725	5.93E-04
0	2.99E-04	0.002044	0	1.58E-04	9.58E-04	0	0.010737	0	1.98E-04	2.96E-04	0	0
4.60E-05	9.82E-04	5.90E-05	0	7.22E-04	0.001951	0	0.001976	0	0.001325	3.81E-04	0	3.80E-05
4.30E-05	0.003425	9.04E-04	2.90E-05	6.60E-05	2.65E-04	5.90E-05	2.21E-04	1.53E-04	5.50E-04	5.50E-04	0.001965	7.80E-05
0	1.13E-04	3.60E-05	0	8.58E-04	0.003561	3.10E-05	0.002014	3.40E-05	2.97E-04	6.35E-04	3.86E-04	0

1.71E-04	0.002152	1.08E-04	1.69E-04	2.93E-04	6.10E-05	0.001702	4.40E-05	3.31E-04	6.09E-04	0.001138	6.12E-04	0
0	5.60E-05	0	0	4.77E-04	0.002398	0	6.40E-04	0	1.25E-04	6.55E-04	5.90E-05	0
0	3.35E-04	2.60E-05	3.40E-05	2.61E-04	0.001066	0	7.22E-04	0	3.23E-04	2.50E-04	1.89E-04	0
5.97E-04	2.47E-04	8.30E-05	0	1.80E-05	0	0	0	2.79E-04	0	9.60E-05	2.66E-04	0
0	0	0.00154	0	9.50E-05	1.25E-04	0	8.93E-04	0	1.59E-04	0	0	0
9.40E-05	2.95E-04	2.99E-04	0	1.84E-04	7.26E-04	5.20E-05	0.002615	0	7.09E-04	8.90E-05	3.10E-05	0
0	0	0	0	0	0	0	0	0	0	0	0	0
1.38E-04	5.71E-04	0.00159	0.001272	9.80E-05	4.65E-04	1.86E-04	0	5.17E-04	0.00121	2.72E-04	0.001778	5.40E-05
1.20E-04	0	0	0	0	0	0	0	3.70E-05	0	0	0	1.72E-04
0	0	0	0	0	0	2.80E-05	0	0	0	0	0	7.00E-05
0	2.46E-04	0	2.80E-05	3.25E-04	0.001344	0	6.96E-04	0	4.03E-04	2.75E-04	0	0
3.60E-05	0	0	0	0	0	0	0	0	4.90E-04	0	6.24E-04	0
1.16E-04	1.49E-04	1.29E-04	0	1.96E-04	8.80E-05	1.06E-04	0	1.10E-04	0	0	3.60E-05	1.27E-04
0	0	0	0	0	0	0	0	0	0	0	0	0
0	2.19E-04	1.70E-05	0	9.30E-05	8.71E-04	0	7.72E-04	0	3.37E-04	1.28E-04	1.50E-05	0
6.80E-04	0	1.90E-05	0	0	0	5.80E-05	0	0	0	0	1.80E-05	4.10E-05
9.10E-05	0	4.96E-04	0	0	0	0	0	0	2.70E-05	2.07E-04	8.42E-04	1.68E-04
0	0	2.10E-05	0	0	0	0	0	0	0	1.96E-04	0	0
0	3.28E-04	2.00E-05	2.42E-04	1.58E-04	7.14E-04	0	5.19E-04	0	3.14E-04	1.22E-04	0	0
3.57E-04	1.45E-04	2.50E-05	1.75E-04	1.22E-04	1.85E-04	3.05E-04	3.30E-04	0.00922	0.003627	1.62E-04	5.00E-05	0
0	0	3.97E-04	0	0	0	0	0.00116	0	0	0	0	0
0	0	0.002719	0	0	0	0	0.004107	0	0	5.90E-05	0	0
0	0	0.004628	0	0	0	0	0	0	0	0	0	0
0	0	0	0	0	0	0	0	0	0	0	0	0
0	1.07E-04	2.20E-05	0	1.09E-04	2.79E-04	0	3.90E-04	0	1.21E-04	0	0	0
0	0	0	0	0	0	0	0	0	0	0	0	0
0	2.00E-05	2.80E-05	3.40E-05	2.46E-04	0.001399	3.00E-05	0	0	1.93E-04	3.65E-04	3.16E-04	0
0	0	1.40E-05	0	0	0	0	2.70E-05	0	1.21E-04	2.60E-05	0	0
0	0	4.30E-05	0	0	0	0	5.14E-04	0	0	0	0	0
0	0	0	0	0	0	0	0	0	2.40E-05	0	0	0
0.003334	0.011181	0.029681	0.007206	0.015654	0.030112	0.007275	0.06551	0.005432	0.023675	0.01925	0.028401	0.010173

H096	H089	H123	H132	H049	H075	H119	H017	H102	H121	H004	H101	H084
0.072433	0.072416	0.071501	0.070107	0.069697	0.06636	0.066079	0.059026	0.055598	0.055576	0.054116	0.049748	0.048347
0.23782	0.16138	0.256412	0.233684	0.27262	0.343847	0.092471	0.592309	0.100881	0.402911	0.351372	0.387589	0.285357
0.123306	0.013358	0.039892	0.111905	0.034798	0.077263	0.230215	0.025041	0.0401	0.027907	0.010797	0.131648	0.136224
0.024614	0.050707	0.021302	0.017111	0.043186	0.010791	0.027098	0.029099	0.017279	0.01783	0.009414	0.020369	0.012381
0.011879	0.099403	0.024889	0.007535	0.019741	0.004841	0.007812	0.013628	0.024857	0.009859	0.011117	0.003416	0.007271
0.079563	0.203273	0.058749	0.036124	0.098068	0.037117	0.06906	0.051852	0.135272	0.080173	0.066355	0.031159	0.066038
0.047837	0.056436	0.091529	0.05212	0.021281	0.029032	0.087334	0.023121	0.107018	0.042964	0.051414	0.012479	0.030248
0.049088	0.047612	0.028868	0.047049	0.099885	0.082654	0.075575	0.062966	0.062016	0.076527	0.075291	0.059184	0.061635
0.160343	0.013338	0.086196	0.164044	0.05661	0.091134	0.157737	0.037552	0.056766	0.068295	0.060488	0.09369	0.077833
0.008202	0.018772	0.014257	0.021473	0.013562	0.002203	0.004479	0.009632	0.006259	0.008452	0.003647	0.015738	0.006742
0.026199	0.004859	0.023917	0.028368	0.014595	0.01507	0.0033	0.003957	0.005896	0.004225	0.027764	0.057745	0.008322
0.014355	0.095504	0.040748	0.009352	0.077019	0.008291	0.010023	0.010632	0.063832	0.043636	0.035273	0.005362	0.017609
0.010681	8.83E-04	0.00437	0.038924	0.008273	0.02764	0.026033	0.002812	0.005123	0.003802	6.10E-04	0.024194	0.012869
0.003291	0.020938	0.004319	0.004786	0.008441	0.003056	0.00369	0.003942	0.006611	0.009905	0.005616	0.002148	0.001858
0.005517	0.019275	0.005714	0.006825	0.014786	0.003519	0.004417	0.00286	0.006518	0.005185	0.002272	0.0021	9.98E-04
0.0158	0.001638	0.007284	0.022086	0.008567	0.006197	0.003833	0.007768	0.003736	0.006442	0.009455	0.027403	0.003734
0.005414	0.012892	0.00982	0.008972	0.017742	0.002963	0.009404	0.001947	0.020206	0.003964	0.003876	0.006226	0.003942
5.20E-04	0	2.77E-04	2.93E-04	1.65E-04	0.002928	3.80E-04	4.50E-05	7.80E-05	4.80E-05	5.30E-05	1.76E-04	3.16E-04
8.00E-05	4.30E-05	9.60E-05	0	0	0	0	0	0.193053	0.046014	0.1433	0	1.17E-04
0.0033	1.71E-04	0.10348	0.038758	2.20E-05	0.071599	4.16E-04	1.04E-04	0.002074	7.10E-04	2.46E-04	0.009481	0.137324
0.001533	2.50E-04	0.002535	0.003821	2.79E-04	8.86E-04	0.002604	0.001825	0.001014	0.001406	0.002376	0.001018	0.003605
0.021951	0.011371	0.02254	6.48E-04	0.014143	0.009946	0.016672	0.002612	0.008626	0.016479	0.007056	3.00E-04	0.004265
0.002418	0.004869	0.003206	0.002462	0.002735	7.88E-04	0.003691	0.003355	0.0014	0.001955	0.001936	6.38E-04	0.001484
0.001022	0.00348	0.002746	0.001267	0.004427	9.26E-04	0.003455	0.001939	0.002264	0.001738	0.001464	0.00102	0.00198
0.001216	0.002606	0.002303	0.001016	0.001344	4.43E-04	0.00149	0.004401	8.12E-04	3.98E-04	2.40E-05	6.20E-05	3.28E-04
0.001015	1.00E-04	0.002233	3.31E-04	7.34E-04	5.85E-04	0.004234	1.90E-04	1.71E-04	0.004424	1.75E-04	0.001165	0.001349
0.001404	0.003204	0.002717	0.001574	0.002187	0.001002	0.002157	0.002618	9.14E-04	0.001585	0.002023	0.0016	8.90E-04
0.001986	0.001828	0.003618	0.002278	0.00322	6.50E-05	7.69E-04	0.001586	0.003831	0.001891	9.90E-05	2.66E-04	8.79E-04
0.001082	0.001117	0.001146	1.88E-04	5.00E-05	1.75E-04	3.90E-04	3.96E-04	4.13E-04	0	0	4.20E-05	7.58E-04
0.001979	0.004262	0.002322	5.41E-04	4.60E-04	6.20E-04	0.003087	7.83E-04	0.006949	7.94E-04	8.46E-04	9.13E-04	6.59E-04
0.008081	2.75E-04	0.007208	0.004546	0.003402	0.042935	0.005558	1.36E-04	0.001117	0.002207	7.12E-04	0.003374	0.006785
3.67E-04	0.001778	0.002146	0.004611	5.07E-04	1.72E-04	0.001401	2.75E-04	3.70E-05	1.14E-04	2.17E-04	1.36E-04	6.68E-04
0.001424	0.001729	0.001431	4.81E-04	0.002519	5.97E-04	0.002532	0.001739	8.98E-04	0.001249	6.90E-04	0.001265	0.001184
8.65E-04	0.001195	6.96E-04	0.00111	7.89E-04	7.40E-05	7.96E-04	5.11E-04	0.001	2.29E-04	1.90E-05	2.02E-04	3.03E-04
0.001336	0.001274	0.00719	0.014037	1.55E-04	0.001753	0.003893	0.003099	0.00627	0.003879	2.72E-04	0.005437	0.003469
3.30E-05	0	1.62E-04	3.40E-05	7.00E-05	5.10E-05	3.55E-04	3.10E-05	0	2.17E-04	2.50E-05	0	4.50E-05
0.001794	0.014498	0.002729	3.23E-04	0.006913	3.19E-04	0.002273	0.002306	0.005853	6.34E-04	1.66E-04	0.001255	0.00383
8.42E-04	0.00278	0.00531	0.002297	5.88E-04	0.002449	5.61E-04	0.007431	5.11E-04	5.81E-04	2.87E-04	0.004406	4.80E-04
0.004763	0.001444	0.001926	0.003467	0.014883	0.005918	0.003584	0.003968	0.002257	0.002352	7.44E-04	0.004248	0.00445
9.92E-04	4.80E-05	8.06E-04	1.59E-04	4.61E-04	4.49E-04	0.002152	3.90E-05	0	0.002194	1.91E-04	2.55E-04	8.56E-04
1.28E-04	4.00E-05	1.49E-04	3.60E-04	3.10E-04	1.04E-04	4.76E-04	1.73E-04	0.003724	3.44E-04	6.90E-05	1.61E-04	3.21E-04
7.86E-04	4.26E-04	0.009005	5.61E-04	5.29E-04	0.001578	3.78E-04	4.19E-04	0.001755	0.001549	0.020801	1.90E-05	7.01E-04
0.009965	6.10E-04	4.37E-04	0.00429	2.18E-04	0.001678	0.004368	0.00264	0.001368	0.00102	1.89E-04	5.32E-04	3.62E-04
0	1.93E-04	8.20E-05	0.004578	0	0.003023	0.005481	3.98E-04	5.98E-04	7.50E-05	4.70E-05	0.006458	0.017172
0.002061	0.002493	0.00174	0.001818	0.004913	7.18E-04	0.003924	0.002225	0.004626	0.004128	0.00093	0.001865	0.001246
0.002517	0.0015	0.001496	0.001937	0.001703	0.002708	0.008594	5.06E-04	0.003541	0.002137	0.003264	0.00116	0.001962
6.34E-04	0	9.59E-04	2.15E-04	2.96E-04	2.33E-04	9.50E-05	0	6.80E-05	0.00118	0	2.62E-04	5.39E-04
0.00169	0.009742	0.001521	1.64E-04	0.005827	3.43E-04	0.001617	6.30E-05	0.005522	0.001708	6.94E-04	9.00E-05	0.001424
8.30E-05	3.96E-04	1.76E-04	5.45E-04	1.29E-04	1.50E-05	2.50E-05	0.002078	9.60E-05	1.43E-04	2.20E-05	0	3.64E-04
5.70E-05	1.92E-04	8.70E-05	5.81E-04	0	8.80E-05	0	3.60E-04	0.001234	4.80E-05	1.70E-05	5.50E-05	0.002144
7.58E-04	0.003331	4.77E-04	9.39E-04	0.012799	0.001373	0.002702	0.001729	0.003408	0.002351	4.81E-04	0.001951	0.002279
0.002891	0.003381	4.26E-04	3.46E-04	4.32E-04	0.001023	6.64E-04	9.76E-04	4.82E-04	7.80E-05	4.50E-05	2.00E-05	3.85E-04
1.79E-04	0	1.47E-04	0	5.90E-05	1.50E-04	4.26E-04	0	3.90E-05	2.46E-04	8.80E-05	5.90E-05	4.10E-05
3.38E-04	0	4.00E-04	3.40E-05	1.87E-04	2.44E-04	0.00108	2.60E-05	0	8.49E-04	4.80E-05	4.40E-05	2.46E-04
0.001398	0.001627	2.80E-05	3.91E-04	9.10E-05	2.74E-04	2.09E-04	5.59E-04	2.45E-04	2.24E-04	0	3.40E-05	1.20E-05
8.04E-04	0	9.05E-04	5.26E-04	3.38E-04	0.002197	4.30E-04	0	1.06E-04	5.04E-04	1.12E-04	4.67E-04	5.08E-04

5.27E-04	0.009116	5.71E-04	2.49E-04	0.001046	1.02E-04	7.19E-04	4.40E-04	8.38E-04	4.38E-04	3.62E-04	8.63E-04	5.50E-04
3.64E-04	0	6.41E-04	1.95E-04	3.73E-04	1.87E-04	2.15E-04	0	0	6.06E-04	0	1.88E-04	4.76E-04
1.84E-04	0	2.59E-04	4.40E-05	7.00E-05	1.23E-04	1.78E-04	0	0	3.34E-04	0	0	9.50E-05
1.24E-04	0	0	8.80E-05	4.90E-05	3.70E-05	2.08E-04	1.95E-04	3.60E-05	2.10E-05	0	0	1.37E-04
7.30E-05	0	2.70E-05	3.47E-04	0	4.60E-05	1.50E-05	0	0	8.00E-05	0	0	0
3.31E-04	0	2.49E-04	3.30E-05	0.00112	4.81E-04	7.32E-04	7.20E-05	0	7.08E-04	0	4.10E-05	3.29E-04
0	0	0	0.002807	0	0	0	0	0	0	9.89E-04	0	0
0	0.001232	1.93E-04	2.15E-04	5.36E-04	1.75E-04	5.91E-04	3.20E-05	5.81E-04	7.88E-04	8.80E-05	0	2.68E-04
0	0	0	0	0	0	0	2.80E-05	5.40E-05	2.00E-05	0	0	0
0	3.80E-05	3.30E-05	0	0	0	0	1.51E-04	0	4.20E-05	0	0	0
1.40E-04	0	1.40E-04	7.40E-05	6.80E-05	7.80E-05	3.92E-04	0	0	3.86E-04	2.10E-05	2.40E-05	6.50E-05
4.90E-05	0	0	0	0	0	0	0	0	0	0	0	0
3.76E-04	1.17E-04	1.72E-04	7.20E-05	2.57E-04	1.05E-04	4.80E-05	1.02E-04	8.12E-04	5.30E-05	1.80E-05	2.10E-05	1.72E-04
0	0	0	0	0	0	0	0	0	0	0	0	0
1.81E-04	0	2.25E-04	0	1.02E-04	6.00E-05	2.57E-04	0	0	1.77E-04	3.10E-05	3.10E-05	1.01E-04
0	2.80E-05	0	0	0	0	0	1.55E-04	1.11E-04	2.40E-05	0	0	0
0	3.96E-04	0	0	0	1.23E-04	1.70E-05	2.09E-04	0	0	0	0	2.20E-05
2.10E-05	1.54E-04	3.00E-05	0	0	0	1.85E-04	7.30E-05	0	0	0	0	0
8.40E-05	0.001905	9.90E-05	4.50E-05	7.80E-05	8.10E-05	2.49E-04	0	0	3.50E-04	1.30E-04	7.40E-05	1.66E-04
5.80E-05	0	4.89E-04	2.44E-04	9.90E-05	0.00547	0	0.001048	2.25E-04	0.004673	0.022979	0	2.72E-04
0	0	0	0	0	0	0	0	0	0	0	0	0
0	0	0	0	0	0	0	0	0	0	0	0	0
0	7.70E-05	8.40E-05	0	0	0.003048	0	1.35E-04	0	0.002803	8.84E-04	9.70E-05	0
0	0	0	0	0	0	0	0	0	0	0	0	0
6.30E-05	7.80E-05	5.20E-05	0	1.70E-05	3.40E-05	6.60E-05	0	0	1.43E-04	0	1.80E-05	1.33E-04
0	0	0	0	0	0	0	0	0	0	0	0	0
4.69E-04	0	2.12E-04	2.33E-04	1.77E-04	0.001246	1.00E-04	0	3.10E-05	1.59E-04	2.00E-05	9.70E-05	2.81E-04
0	0	0	7.70E-05	5.63E-04	3.20E-05	0	0	0	0	0	2.00E-05	0
0	0	0	0	0	0	0	0	0	0	0	0	0
0	0	0	0	0	0	4.00E-05	0	0	0	0	0	0
0.016322	0.011922	0.009899	0.013285	0.02869	0.016165	0.024509	0.007675	0.01299	0.01286	0.005873	0.017892	0.009795

H068	H087	H033	H149	H052	H063
0.043401	0.034027	0.03161	0.029516	0.023253	0.012352
0.169206	0.260711	0.217617	0.406833	0.410569	0.42904
0.036728	0.025662	0.02628	0.159983	0.021887	0.074793
0.020026	0.008026	0.004564	0.014118	0.040834	0.005208
0.01079	0.010431	0.031519	0.017232	0.014386	0.01926
0.062947	0.055917	0.036587	0.054527	0.003607	0.035331
0.072926	0.107158	0.020435	0.008205	0.048838	0.015144
0.065241	0.0631	0.032371	0.050286	0.030687	0.078526
0.027163	0.053166	0.02535	0.092341	0.10832	0.069864
0.003731	0.002491	0.003884	0.013039	0.004708	0.019458
0.00239	0.018537	0.002011	0.015014	0.005775	0.037003
0.010623	0.044709	0.045998	0.013448	0.165554	0.033818
0.005889	0.004801	0.002386	0.006587	0.004257	0.005704
0.003302	0.004287	0.003821	0.002557	2.87E-04	0.001946
0.00396	0.002401	0.002549	0.001069	0.003298	0.003092
0.004382	0.011127	0.001748	0.019426	0.015098	0.025832
0.004858	0.006748	0.006035	0.004916	0.009123	0.006046
2.31E-04	3.10E-05	8.90E-05	1.20E-04	3.10E-05	1.40E-04
0.380934	0.21226	0.115589	4.66E-04	0	0
0.006604	0.00857	0.025149	0.019295	0.004926	0.00251
0.003089	0.001432	0.002924	0.002238	0.006103	0.031574
0.004345	0.019892	0.006622	0.001606	0.027462	0.006378
0.001378	5.00E-04	5.40E-04	0.001529	4.65E-04	9.05E-04
0.002683	0.001071	6.28E-04	7.75E-04	1.54E-04	7.39E-04
2.41E-04	1.27E-04	1.97E-04	4.46E-04	9.27E-04	3.15E-04
0.001164	3.57E-04	0.001581	4.01E-04	0.003716	2.82E-04
0.001976	0.001312	8.19E-04	7.68E-04	0.002272	2.91E-04
8.14E-04	1.43E-04	3.59E-04	4.66E-04	1.15E-04	5.10E-05
1.26E-04	0	0	0	1.30E-04	0
0.004447	0.001982	0.004219	0.002154	0.001208	0.001721
0.003541	0.001729	2.41E-04	0.001619	0.002212	0.002943
0.001514	0	3.52E-04	2.41E-04	0	0
0.001241	2.42E-04	3.76E-04	0.001946	1.23E-04	5.31E-04
0.00154	5.50E-05	1.31E-04	8.90E-05	0	6.00E-05
0.002997	2.31E-04	0.004334	0.007299	0.003564	0.008088
5.60E-05	7.80E-05	0.134603	0	2.01E-04	3.80E-05
0.001791	3.50E-04	0.002997	0.002723	3.46E-04	0.001389
1.69E-04	4.44E-04	0.009569	0.001033	0.003825	0.010472
0.001605	5.42E-04	0.001231	0.005052	0.00129	0.001855
7.57E-04	2.66E-04	3.70E-05	2.80E-05	0.002008	2.08E-04
1.47E-04	1.70E-05	0.018764	3.40E-05	4.01E-04	1.13E-04
0.001677	0.001281	0.003482	0.003339	0.001552	0.021596
3.19E-04	0.013288	5.96E-04	9.73E-04	3.19E-04	0.003607
9.68E-04	0.002462	0.002365	0.00188	9.44E-04	2.50E-04
0.00339	0.00155	6.84E-04	0.001473	4.58E-04	0.001091
0.001352	0.002191	0.018911	0.003383	0.004174	0.006733
5.00E-04	1.61E-04	3.60E-05	0	0.001229	1.46E-04
0.001916	0.001565	0.004688	7.00E-04	0.001189	0.001756
0	6.70E-05	2.39E-04	1.20E-04	2.27E-04	2.84E-04
2.90E-05	2.20E-05	1.02E-04	1.31E-04	0.002356	0.004216
0.00165	2.38E-04	0.001445	0.003517	0.001511	0.001418
6.17E-04	3.97E-04	2.89E-04	7.03E-04	1.90E-04	0
2.80E-05	2.80E-05	0.012485	8.70E-05	3.84E-04	1.05E-04
3.20E-04	1.57E-04	4.40E-05	0	8.07E-04	2.90E-05
2.62E-04	8.80E-05	1.60E-04	3.00E-04	4.17E-04	5.28E-04
4.72E-04	9.30E-05	0	9.70E-05	0.001157	2.28E-04

0.003429	0.001014	0.002123	0.004603	8.50E-05	1.09E-04
3.43E-04	1.91E-04	0	0	7.85E-04	6.70E-05
1.07E-04	1.01E-04	0	0	2.77E-04	7.70E-05
0	0	0	0	1.71E-04	4.80E-05
6.50E-05	0	0.041245	0	3.80E-05	1.80E-05
1.58E-04	2.40E-05	0.001851	4.36E-04	3.08E-04	2.00E-05
3.13E-04	0	0	0	4.70E-05	0
1.20E-04	5.62E-04	0	1.21E-04	0	3.36E-04
0	0	0	2.41E-04	0	0
0	0	0	1.80E-04	0	0
1.06E-04	5.50E-05	0	0	4.74E-04	4.20E-05
0	0	0	0	0	0
2.27E-04	0	0	0	1.80E-05	4.10E-05
0	0	0	0	0	0
1.20E-04	0	4.20E-05	0	2.90E-04	4.60E-05
0	0	8.70E-05	0.001006	0	0
1.70E-05	0	0	0	4.40E-05	0
0	1.80E-05	0	3.10E-05	0	0
0.001233	2.51E-04	2.38E-04	0	2.21E-04	1.97E-04
4.38E-04	0.003954	0.006509	5.97E-04	2.27E-04	0.00352
0	0	0.008708	0	0	0
0	0	0.007717	0	0	0
3.41E-04	0	0	8.06E-04	4.20E-05	0
0	0	0	0	0	0
2.30E-05	6.00E-05	0	0	2.87E-04	2.80E-05
0	0	0	0	0	0
2.75E-04	7.10E-05	4.40E-05	8.60E-05	6.80E-05	1.29E-04
0	2.30E-05	0	3.40E-05	0	2.55E-04
0	0	1.56E-04	0	0	0
0	0	0	0	0	0
0.008232	0.005158	0.059638	0.015731	0.007744	0.01006

Supplementary table S14. The relative abundance and distribution of the key 58 OTUs among CC (n=73), SC (n=36), and H (n=140) groups.								
	CC01	CC02	CC03	CC04	CC05	CC06	CC07	CC08
OTU44 (Pseudomonas)	0	0	0.000263225	0	0	0	0	0
OTU153 (Acinetobacter)	0	0	0.000268386	0	0	0	0	0
OTU92 (Faecalibacterium)	0	0.000377119	0.000593547	9.47E-05	0	0	0	0
OTU132 (Bacteroides)	0	0	0.000252903	0	0	5.48E-05	0	0
OTU146 ([Eubacterium]_ruminantium_group)	0	0	0	0	0	0	0	0
OTU234 (Prevotella)	0	0.000135376	0	0	0	0	0	0
OTU99 (Prevotella)	0	0	6.45E-05	0	0	0	0	0
OTU80 (Fusicatenibacter)	0	0	0	0	0	0	0	0
OTU208 (Bacteroides)	0	0	5.16E-05	0	0	0	0	0
OTU33 (Rothia)	0.010236177	0.000161162	0	0.002020245	0	0.000421329	0	0
OTU114 (Prevotella)	0	0	0.000105806	0	6.04E-05	0.002220406	0	0
OTU86 (Corynebacterium)	0	0	0	0	0	0	0	0
OTU2 (Fusobacterium)	0.01513093	0.088316728	0.018245114	0.255122512	0.000142549	4.63E-05	0.013740028	0.001148018
OTU5 (Haemophilus)	0.0155776	0.000248189	0.258598042	0.017817434	0.001935283	5.48E-05	0.002196152	0.001071483
OTU26 (Prevotella)	0.007128101	0.001028213	0.000611611	0.001197767	0.000604021	0	0.001576725	0.003979795
OTU65 (Alloprevotella)	0	0	0	0	0	0	0.000638198	0
OTU48 (g__Absconditabacteriales_)	0	0	0	0	0	5.06E-05	0	0
OTU53 (g__Absconditabacteriales_)	0	0	0	0	0.015876084	0	0	0
OTU78 (Saccharimonadales)	0	0	0	0	0	0	0	0
OTU12 (Alloprevotella)	0	0.034572454	0	0.000327939	0.00589766	0.008658319	0	0
OTU84 (Alloprevotella)	0	0.000441584	0	0	0	0.001377747	0.000732051	0
OTU54 (Alloprevotella)	0	0.013079901	0	0	0.007506771	0.000615141	0	0
OTU308 (Alloprevotella)	0	6.45E-05	0	5.44E-05	0	0	0	0
OTU35 (Actinobacillus)	0	0	0	0	0	0	0	0
OTU77 (Haemophilus)	0	0	0	0	0	0	0	0
OTU42 (Haemophilus)	0	0	0	0	0	0	0	0
OTU46 (Campylobacter)	0	0	0.01697544	5.26E-05	0	0.000311784	0.000769592	0
OTU367 (Fusobacterium)	0	0.00849001	0.001607738	0.016765224	0.000200535	0	0.001482872	0.003444053
OTU63 (Porphyromonas)	0	6.12E-05	0.000129032	0.000324432	0.005972558	0	0	0
OTU37 (Porphyromonas)	0	6.45E-05	0.026712189	0	0	0	0	0
OTU126 (Prevotella)	0	0	0	0	0	0.000117972	0	0.001186285
OTU38 (Leptotrichia)	0	0.000415798	0.002559993	0.086667088	0.044383452	0.005211844	0.029300798	0.006237563
OTU218 (Leptotrichia)	0	0.000731675	0.000621934	0.030186171	0.006470271	0.002304672	0.002214923	0.001989897
OTU55 (Capnocytophaga)	0	0	0.00084903	0	0	0	0	0
OTU34 (Capnocytophaga)	0.002493905	0.000103144	0.002265801	0.000592745	0	0	0	0
OTU256 (Capnocytophaga)	0	0	0.000129032	0.000105221	0	0	0	0
OTU104 (Capnocytophaga)	0	0	0	0	0	0	0	0
OTU608 (Capnocytophaga)	0	0	0	0	7.25E-05	0	0	0
OTU15 (Halomonas)	0.00241946	0.013660084	0.085677198	0	0.000142549	0	0	0.001033216
OTU82 (Pelagibacterium)	0	0.001595503	0.005919985	0	0	0	0	0
OTU9 (Gemella)	0	7.41E-05	0.119493885	0.03449322	0.000906031	0.011645544	0.000581886	0.003711924
OTU1 (Streptococcus)	0.038115799	0.091527074	0.322716587	0.311550818	0.045603574	0.203813874	0.0229939	0.00390326
OTU70 (Streptococcus)	0.010161732	0.006481932	0.130851275	0.098194056	0.004001034	0.149647769	0.001595495	0.001913363
OTU728 (Leptotrichia)	0.002549739	0	0	0.001660739	0.000314091	0.000282291	0.062580948	0.023955304
OTU444 (Leptotrichia)	0	0	0.000539353	0.000201674	0	0.000134825	0.145565462	0.03964488
OTU1106 (Leptotrichia)	0	0	0	0	0	0	0.025978414	0.006314098
OTU633 (Rothia)	0.007072267	0.001814683	5.94E-05	5.26E-05	0	0.000164318	0.138094791	0.007155977
OTU644 (Rothia)	0	0.003854993	0	5.79E-05	0.00022228	0.001289268	0.082008447	0.001186285
OTU939 (Actinomyces)	0.020137351	0.000937962	0	0	8.94E-05	0.000501382	0.183256687	0.030728609
OTU892 (Streptococcus)	0.010068675	0.00134409	0	0.005115498	0.001401328	0.004457665	0.012576255	0.011480178
OTU626 (Actinobacteria_unclassified)	0.005043643	7.09E-05	0	0	0	8.85E-05	0.029000469	0.010714832
OTU934 (Actinomyces)	0.015261209	0	0	0	0	4.63E-05	0.186804317	0.410607684
OTU761 (Prevotella)	0.077404106	0.001740549	0	0.000308648	0.001140391	0.002186699	0.00352886	0.010446962
OTU21 (Campylobacter)	0.027972679	0.00374218	0.000748385	0.002374489	0.008565016	0.001154442	0.00213984	0.004936476
OTU3 (Prevotella)	0.689024958	0.484475273	0.001651609	0.113961433	0.60536902	0.350112074	0.008390427	0.060653605
OTU11 (TM7x)	0.002624183	0	0.000492902	0.001736148	0.175388325	0.184828772	0.016593149	0.017105465
OTU16 (Lachnoanaerobaculum)	0.041577488	0.229745977	0.000867095	0.015453468	0.064934657	0.062112377	0.024289066	0.303650696
OTU23 (Solobacterium)	0	0.010643133	7.74E-05	0.003510876	0.002800241	0.00608821	0.001370249	0.031800092

CC09	CC10	CC11	CC12	CC13	CC15	CC16	CC17	CC18	CC19	CC20
0	0.000184559	9.43E-05	6.05E-05	0	0	8.65E-05	0	8.17E-05	0	0.001353722
0	0.001139651	0	2.45E-05	0	0	1.98E-05	0.000134598	3.39E-05	0	0
0	0.001462629	8.60E-05	0	0.000396042	0.000309862	0.001878765	0.096742064	1.79E-05	0	0.001308091
0	0.000908952	0.00010544	0	0	0.000357015	0.001758077	0.093949162	1.79E-05	0	0.000187594
0	0	0	0	0.000696488	0	0	0.001655551	0	0	0
0	0.0011558	0	0	0.000389214	0	0.001959824	0.098444724	0	0	0.000598274
0	6.00E-05	0	0	0	0	0.000491757	0.021360648	0	0	0.000152104
0	6.46E-05	0	0	0	0	0.000443122	0.013809719	0	0	0.000314347
0	0	0	0	0	0	0.000180131	0.005592533	0	0	0
0	0.040443772	0.058604979	0	0.000334587	0.007160515	0.003946667	0.000652799	0.092904062	0.000106416	0.010961599
7.12E-05	6.46E-05	8.32E-05	0	0.000587235	0	0.00020715	0.018217792	0.002463992	0	0
0	0	0	0	0	0	0.003195521	0	0.001477199	0	0
0.193398815	0.001688714	0.000110989	2.96E-05	0	0.000579308	0.010887108	0.008163348	0.004479442	0.000933957	0.014525893
0.003894938	0.011793313	0.000427308	0.08144092	0.038504872	0.00029639	0.010303484	0.020041591	0.002115126	0.000105164	0.000557713
0.001090858	0.114228106	0.005738132	0	0.012523131	0.651667531	0.00057822	0.007840313	0.000147521	0	0.021071418
0	0.002922951	0.000116538	0	0.00194607	0	0.000363864	0	0	0	0.000532363
0	0	0	0	0	0	0	0	3.99E-06	0	0
9.19E-05	0.000625193	0	0	0	0	0	0	0	0	0
0	0	0	0	0	0	0	0	0	0	0
0.001853311	0.051810292	0.00010544	0	0	0.000377224	0.000543995	0.00055185	0.00031697	0	0.002317045
0	5.77E-05	0	0	0	0	4.32E-05	0.000538391	0.000456516	0	0
0	0.005022309	0	5.54E-05	0.000327759	0	9.19E-05	0.003122666	0.000717668	0	0.000278857
0	6.23E-05	0	0	0	0	0	0	0	0	0
7.35E-05	0	0	0	0	0	2.70E-05	0	0	0	0
0	0	0	0	0	0	0	0	0	0	0
0	0	0	0	0	0	0	0	0	0	0
0.003522898	0.000161489	0	0	0.000327759	0	0.016019036	0.015721006	0.002834787	0.000519561	8.62E-05
0.012665437	0.00325285	0.00019978	0	0	0	0.00195442	0.027437732	0.025114378	8.76E-05	0.001510896
0.00235855	0.000768226	0.000110989	2.96E-05	0	0	0.00253264	0.003762004	0.000386743	0	0.000172384
0	0	0	0	0.000355072	0	0.000581823	0	0.000524296	0	0
0	0.000392188	0.000332967	0	0.001925585	0	0.000724126	0.009852548	0.009267879	0	0
0.137434348	0.00192864	0.000124863	0	0.001754877	0	0.004396994	0.030573857	0	0.004107658	0.22439336
0.031315667	0.00604661	0.000640962	0	0.001727564	0	0.00235431	0.011386962	0	0.00042316	0.094268737
0.055231871	0.000569826	0	0	0.003619007	0	0.000861025	0	0.005697483	0	0.000106473
0	0.035580645	0.013973518	0	0	0	0	0.000114408	0.003600299	0	0.001409493
0	0.004459404	0.004997281	0	0	0	0	0	0.000474458	0	0.000223085
0	0.001402647	0	0	0	0	0	0	0	0	0
6.89E-05	0	0	0	0	0	0	0	0.00116621	0	0
0.006924079	0.433344253	0.002158737	0.000320588	0.011150639	0	0.048105744	0.067285367	0.01352604	0.000110172	0.098806494
0.001173534	0.032689992	0.000113764	6.31E-05	0	0	0.002507421	0.003075556	0.000759532	0	0.01313161
0.013023698	0	6.10E-05	0	0	0.042134548	0.183983846	0.019664717	0.219532519	0.000316744	9.13E-05
0.043733077	0.10144971	0.434136339	0.864615333	0.368988522	0.184395061	0.562523417	0.081034518	0.349209071	0.000411893	0.125460113
0.006999865	0.090069348	0.35222644	0.052080732	0.210974469	0.043677123	0.119866271	0.040419675	0.236971842	0.000195305	0.042370991
0.025989982	0	0	0	0	0	0.000160316	0.001857448	0	0.001030357	0.003680907
0.045088038	0	0	0	0	0	0.000387281	0.001527683	0	0.000296713	0
0.000422564	0	0	0.000697826	0.000396042	0	0	0.000100948	9.97E-06	0	0
0	0	0.039969922	1.80E-05	0.001147157	0.000491738	0	0	0.001794169	0	0.006144985
0	0	0.025632915	3.22E-05	0	0.001097991	0	0	0.001255918	0	0.028433232
8.73E-05	5.08E-05	0.010319204	2.70E-05	0.003277592	0.002081467	0	0.000444172	7.77E-05	0	0.000730097
0.002066889	0	0.029406542	0	0.07228455	0.001178824	0	0.000969103	0.001411413	0	0.000324488
0	0	0.001492802	0.000153213	0.001331522	0.00017514	0	0	0.000911039	0	0.002276484
0.00012631	0	0	0	0	0	0	0	3.59E-05	0	0.010637111
0.00074408	0	0.001670385	0	0.0236328	0	0	0.000316304	4.39E-05	0.003719553	0
0.000215875	0.017756871	0.000291346	0	0.00251282	0.049288327	0.001435643	0.078423323	0.001138301	0.000857588	0.002494499
0.085325776	0.031644927	0.01637643	0.00012875	0.226891273	0	0.010694368	0.079203989	0.005117369	0.986587827	0.104865287
0.033334329	0.000163796	0	0.000145488	0	0	0.000113482	0.023850704	0.002392225	0	0.09398481
0.284498102	0.003675029	0.000183132	4.12E-05	0.003106884	0	0.003409877	0.07631014	0.010172938	8.89E-05	0.012148007
0.007174402	0.000897418	0.000108214	3.61E-05	0.008890467	0.014731935	0.000381877	0.035850085	0.001339646	0.000101408	0.078089984

CC21	CC22	CC23	CC24	CC25	CC26	CC27	CC28	CC29	CC30	CC31
6.73E-05	0	0	0	0	0	0	0	0	0	0
0.000173536	0	0	0	0	0	0	0	0	0	0
3.01E-05	0	0	0	0	0	0	0	0.000366207	0	0
0	0	0	0	0	0	2.49E-05	0	0	0	0
0	0	0	0	0	0	0	0	0	0	0
4.43E-05	0	0	0	0	0	3.58E-05	0	0	0	0
0	0	0	4.57E-05	0	0	0	0	0	0	0
0	0	0	0	0	0	0	0	0	0	0
0	0	0	0	0	0	0	0	0	0	0
0.007410713	0.000414516	0	0.00105789	0.002444566	0.001287931	0.011078767	0.012350887	0.009258477	0.00060917	0
0	0	0.000232439	0	0	0	5.92E-05	0	0	0	0
0.000178849	0	0	0.000164737	0	0	0.000322229	0.000290136	7.51E-05	0	0
0.077259116	0.002027295	0.17497341	0.086544549	0.092090686	0.004321999	0.050649128	0.005588475	0.01684085	0.014329745	0.20128204
0.12291867	0.005392188	0.001014277	0.002997025	0.011929802	0.023347878	0.012465753	0.00619553	0.003981333	0.011037636	0.001004818
0.00488204	0.007422966	0.001944032	0.001395304	0.004764695	0.000100448	0.003086862	0.000252196	0.003934383	0.001163903	0.000506895
0.001841611	0	0	0.000256037	0.000477674	0	8.25E-05	0	0	0	0
0.000906639	0	0	0.00147271	0.000939291	0	0.000759651	0.000116055	0	0.000329211	0.000264662
0.000116871	0	0	3.37E-05	0	0.000107328	0.002436177	0.001057882	0	0.000111465	0
0	0	0	0	0	0	0.001788605	0	0	0.000629907	0
0.001058926	0.001313214	0.000799448	0.007290114	0.00096739	0.000191263	0.001014944	0	0.018836211	0.006291297	0.001552085
0	0	0	3.57E-05	0	0	5.14E-05	0	0	0	0
0	0.000400582	0	0.000867351	0.001011544	4.54E-05	0.001000934	0	0	0.003382836	0.000542781
0	0	0	0	0	0	0.00010274	0	0	0.00011665	0
0.029311716	0	0	4.17E-05	0	0	0.000488792	0.000131677	8.92E-05	0	0
0.001947858	0	0	0	0	0.001742009	0.000797011	0.000535637	5.16E-05	0	0
0.000579045	0	0	0	0	5.78E-05	0.000677148	0	0	0	0
4.96E-05	0	0.000387398	0.007220647	0.001433021	0.000473342	0.00501401	0.000120518	0.000450717	0.001767888	0.000228775
0.007803826	0.000456316	0.009913857	0.010340728	0.008176651	0.000302719	0.010622665	0.004550679	0.001910852	0.001088729	0.06620134
0	0	0.002148296	0.004592792	0.004853005	0.000100448	0.001667186	0.002769687	0.000530531	0.000557325	0.0056521
0	0	0	3.97E-05	0	0	0.001640722	0	0	0	0.000278119
0	0	0	0	0	0.00011008	0.001720112	0	9.39E-05	0	0
0.044687395	0.024017528	0.022528932	0.104367916	0.272960453	0.003801873	0.042868929	0.027161238	0.021592158	0.336707529	0.037904955
0.027188551	0.003702775	0.001088235	0.007030107	0.092985822	0.001201243	0.005470112	0.004209211	0.003075204	0.008543929	0.001166306
0.000672896	0.000417999	0	0	0	0	0.000185243	0.000205327	9.39E-05	0.003144352	0
0.005600976	0	0.000250048	0.000537877	0.002392383	5.37E-05	0.001636052	0.000406191	0	0.001560511	0
0.000805705	0	0	0	0	0	0.000241283	0	5.63E-05	0	0
0.000200098	0	0	0	0	0	0	0	0	0	0
0	0	0	3.18E-05	0.001457106	0	2.18E-05	0.000419582	0	0.000168494	0
0.12623534	0	0	0.000335429	0.001023587	0.000591678	3.89E-05	0.002767456	0.010704527	0.000907274	0.001004818
0.007782577	0	0	8.53E-05	0	0	0	0.000129446	0.001018808	0	0
0.111127048	0.002469678	0	0.002407544	0.012371349	0.074076673	0.067408157	0.125318871	0.151609905	0.003753522	0.007419502
0.348007784	0.013306303	0.024571573	0.054109199	0.168667009	0.803163962	0.606541096	0.68568846	0.335633868	0.30783807	0.121722006
0.048301557	0.004733839	0.003483057	0.006488261	0.01232318	0.048381349	0.097048568	0.054795621	0.038667756	0.034014999	0.01325552
0.000671125	0.007158234	0.00021483	0.00308634	0.0130136	0.001107676	0.001271793	0.001622532	0.007216166	0.028732072	0.011977069
0.000329365	0.005009022	0.002243384	0.006089319	0.00292224	0.001787417	0.001804172	0.01152065	0.01320225	0.037804812	0.054820882
0	0.002096962	0.000683229	0.000984453	0.000931263	0.003397331	0.000311333	0.007789048	0.009385241	0.006379432	0.047482124
0	0.061087076	0.003588711	0.000629177	0.030948443	0	0	0.010723891	0.012709278	0.00330507	0.005477154
0	0.03787768	0.002739958	0.000329474	0.018484771	5.78E-05	2.96E-05	0.007878321	0.025179113	0.003079547	0.004095529
0	0.06369957	0.005184084	0.00336024	0.034545046	0.001352603	0.000110523	0.000821309	0.011446332	0.007429278	0.001332281
0	0.004890589	0.003909194	0.003540856	0.014555001	0.016829791	3.74E-05	0.007237789	0.007103486	0.016553862	0.002462701
0	0.007548366	0.002017989	0.000994377	0.031871678	0.002835925	4.51E-05	0.003588765	0.025216673	0.008505046	0.006369827
0	0.011745773	0.00456777	0.004183926	0.029037748	0.000210527	2.80E-05	0	0.002812286	0.014373812	0.002476158
0	0.049470186	0.012569291	0.006518033	0.002974422	0.000206399	0.000476339	0	0.00288271	0.001778257	0.003642464
0.001230692	0	0.000876927	0.03142311	0.002436538	0.000355007	0.002061021	0.000484305	0.005530672	0.006444238	0.00265559
0.008600677	0.55777095	0.690486924	0.554134009	0.087526694	0.003968369	0.013714197	0.00022095	0.179460454	0.087782648	0.379170666
0.002397636	0.016162629	0.000228917	0.011549462	0.002809846	0.000266943	0.040943337	0.008193008	0.004000113	0.013907214	0.002992024
0.007123847	0.054141325	0.010047685	0.021947749	0.025160161	0.003373939	0.006183064	0.004198052	0.059212936	0.01699713	0.012187901
0.002456071	0.05526644	0.017306107	0.051439664	0.009513335	0.000791197	0.0039368	0.000660618	0.015770397	0.00887314	0.002870908

CC32	CC33	CC34	CC35	CC36	CC37	CC38	CC39	CC40	CC41	CC42
0	0	0	0	8.32E-05	0	0	0	0	0	0
0	0	0	0	2.17E-05	0	0	0	0.000287557	0	0
0	0	0	0	0.000450454	0	0	6.34E-05	0.000222038	0	0
0	0	4.81E-05	0.000134165	0.000671158	0	0	4.19E-05	7.64E-05	0	0
0	0	0	0	0	0	0	0	0	0	0
0	0	0	0	0.000633168	0	0	1.91E-05	0	0	0
0	0	0	0	0	0	0	0	0	0	0
0	0	0	0	0.000130252	0	0	0	0	0	0
0	0	0	0	4.70E-05	0	0	0	0	0	0
0	0	0.000288705	0	0.023608116	0.011174188	0	0.002021167	0.009638624	0.000825251	0
0	0.000833598	0	0	0.00011397	0	0.001580022	6.82E-05	0	0	0
0	0	0	0	0.00015196	0	0	6.94E-05	0	0	0
0.363332057	0.003887663	0.069866563	0.000629546	0.081309608	0.027626512	0.011445528	0.006564902	0.00101555	0.003208577	0.001934587
0.004780397	0.006506488	0.010464283	0.000921466	0.121200993	0.018035157	0.004393233	0.015910853	0.058119303	0.007829985	0.159784777
0.001684625	0.000449995	0.041393684	0	0.001921212	0.00351267	0.001406605	0.004505442	0.041422789	0	0.05224056
0	0	0.004421742	0	0.001459904	0.000206986	0	0	0.000531435	0	0.003862456
0	0.00089999	0.011690012	0	0.000180905	0.006477437	0	0	0	0	0
0.000765739	0	0.007326518	0	0.000173669	0.000870558	0	0	0	0	0
0	0.000376225	0.009532324	0	0	0	0	0	0	0	0
0.002330033	0.000774582	0.014772062	0.00011205	0.004216898	0.00126931	0.001464411	0.00012206	0.003862002	0.007935617	0
0	0	0.000412797	0	0	0.000222205	0	0	0	0.001591085	0
0.003883389	0.004440936	0.004107712	0.001086593	0.0024585	0.00021003	0	0.000112486	0.00305029	0	0
0	0	0.002947828	0	0.000443218	0	0	0	0	0	0
0	0	0.000230457	0	0.07743643	0	0	0.001231368	0	0	0
0	0	0.000339355	0	0.01511824	0	0	8.14E-05	0	0.002343714	0
0	0	8.10E-05	0	0.003451669	0.000435279	0	0	0	0.000726221	0
0.000820434	0.00146064	0.000478642	0.000129742	0.002467546	0.002480785	0	0.002534536	0.080901837	0	0.003439265
0.013050375	0	0.006062801	0	0.024063997	0.006927935	0.001445142	0.003635467	5.82E-05	0.003162363	0.000463495
0.002526938	0.001977028	0.031061091	0	0.003133276	0.010169698	0.001541485	0.000493026	0	0	0
0	0.001209823	6.84E-05	0	0	0	0	0	0	0	0
0	0.000494257	0.000258315	0	0.001005832	0.000663572	0	0.000342246	0	0.00705755	0
0.013444183	0.003031935	0.046724593	0.00013564	0.031844726	0.010379728	0	0.000148386	0.092131126	0.001591085	0
0.003347372	0.001401624	0.015096222	0.000138588	0.00325991	0	0.002890285	0.000494223	0.004906671	0.000798843	0
0.379554778	0	0	0	2.71E-05	0	0	0	0	0	0
0.0007548	0	0.000726827	0	0.005474188	0.009984019	0	4.07E-05	0.032723275	0	0
0	0	0	0	0.000969651	0.001439769	0	0	0.003898401	0	0
0	0	7.34E-05	0	0	0	0	0	0	0	0
0	0	0.000119027	0	9.95E-05	0	0	0	0	0	0
0.001662747	0.001497525	4.31E-05	0	0.042407775	0.003856632	0.001425874	0.00021181	0.1088968	0	0.003741545
0	0	0	0	0.003008452	0	0	0.000220186	0.005558225	0	0
0.006038396	0.040831532	0.074417462	0.000246216	0.095649956	0.118541968	0.00591545	0.251668151	0.004426196	0.023476751	0.000960576
0.037280534	0.591647794	0.272203046	0.004072143	0.346335586	0.474481394	0.498208023	0.649584279	0.036836435	0.309363632	0.431029966
0.007799595	0.049580619	0.01841886	0.003068113	0.056507518	0.053804124	0.030097499	0.047907273	0.005944061	0.018492233	0.226736258
0.007515178	0.008092537	0.00068884	0	0	0.000222205	0	0	0	0.005651321	0
0.00561177	0.019084223	0.001086442	0.000107627	0.00045769	0.001567613	0	0	0	0.022030911	0
0.002209703	0.089748224	0.001251054	0	0	0.004261472	0.00148368	0	0	0.030448475	0.001296442
0.001629929	0.003356522	0.001218132	0.001037939	0	0.01668975	0	0	0	0.012774891	0
0.00074386	0.00389504	0.001365017	0.000378907	0.000103116	0.011481622	0	0	0	0.007083958	0.000409756
0.002362851	0.00219096	0.000412797	0.002353056	0	0.017870786	0.129369147	0	0	0.004192277	0.000517233
0.00449598	0.022145666	0.001539759	0.000398073	5.97E-05	0.019404916	0.122316852	0.00014001	6.55E-05	0.011256429	0.064439205
0.002636329	0.005001586	0.001810736	0.002266069	0	0.027532151	0.037670045	0	0	0.012207118	0.000517233
0.000809495	0.008785972	0.00044572	0.010142024	3.26E-05	0.002027243	0	0	0	0.008688246	0
0.003281737	0.014930988	0.000942089	0.005179376	3.08E-05	0.002209877	0.101314116	0	0	0.01063584	0.006381449
0.000787617	0.02023503	0.001023129	0.000654609	0.003147749	0.001287573	0	0.003566061	0.011025451	0.009737966	0.005723153
0.076803588	0.056183008	0.156171572	0.966091524	0.027976974	0.102619283	0.032217041	0.000594742	0.459836638	0.448976358	0.012339708
0.011179784	0.005481089	0.150096108	0.000486534	0.012701347	0.00716536	0.002774673	0.002028347	0.007501966	0.004674224	0.000490364
0.033003336	0.028371829	0.025236345	0.000113525	0.00139116	0.018488699	0.008285483	0.004643059	0.014177659	0.018472427	0.006771054
0.00387245	0.001195069	0.013037301	0.000116473	0.002561616	0.004401492	0.002755405	0.000935792	0.012885472	0.004766652	0.016920917

CC43	CC44	CC45	CC46	CC47	CC48	CC49	CC50	CC51	CC52	CC53
0.000795324	0	0	0	0	0.000991285	0	0	0	0.004496933	0
0.000717095	0	0	0.000788595	0	0.000952026	0	0	0	0.000378383	0
0.002642387	0.000332349	0	0.000147564	0	0.000461291	0	0	8.91E-05	0.002534679	0
0.002042635	0	7.27E-05	0.000171365	0	0.000206109	0	0	0	0.001831275	0
0	0	0	0	0	0	0	0	0	5.09E-05	0
0.002698885	0	0	0	0	0	0	0	3.36E-05	0.002258169	0
0.000634521	0	0	0	0	0	0	0	0	0.000790723	0
5.22E-05	0	0	0	0	0	0	0	0	6.06E-05	0
0.000160803	0	0	0	0	0	0	0	0	5.09E-05	0
0	0.002522444	0.000649137	0.013144303	0	0	0	0	0.043173377	0.053487791	5.03E-05
0	0	5.97E-05	0.011322761	0	0	0	0	4.26E-05	0.002430381	0.00010943
0	0.000115044	6.62E-05	0	0	0	0	0	0	0.002061701	0
0.001051739	0.036373049	0.007626549	0.003040136	0.017183302	0.000922582	0.020169097	0.000324885	0.009562504	0.027757767	0.001575796
0.00086486	0.04859753	0.031392705	0.030534642	0.000246242	0.00196294	0.001698536	0.000782131	0.032459806	0.10284733	0.000402703
4.78E-05	0.006139936	0.003263446	0	0	0.000176665	0.002138697	0	6.07E-05	0.000356553	0.000877631
0	0	0.001548563	0.000161844	0	0.0003337	0	0	3.36E-05	0	0
0	0	0.002058829	0	0	0.000245367	0.000164723	0	0	0.000240127	0
0	0	0.000737949	0	0	0	0	0	0	0.000213447	0
0	0	0.001687433	0	0	0.000206109	0	0	0	0	0
0.000195571	0.000402654	0.000510267	0	0.00029044	0	0.000607584	0	0.000131772	0	8.54E-05
0	0.000206653	0	0.000158671	0.000284126	0	0	0	3.36E-05	0.000121277	0
0	0.000873482	0.002031378	0.001899292	0.001859446	0	0	0.002305783	0	0.00121034	0
0	0	0.000130796	0	0	0	0	0	0	6.55E-05	0
0	0	0.000532873	0.000153911	0	0.000225738	0	0	0.002405483	0.000400212	0
0	0.000123566	0.00939795	0	0	0	0	0	0.000426321	0.005023273	3.72E-05
0	0	0.008349965	0	0	0	0	0	0	0.001649361	0
0.000847476	0.000828742	0.001083509	0.020809698	0	0.034557553	0	0	0.000897857	0.006328208	3.50E-05
0.001747104	0.0022114	0.004372792	0.004664927	0.001246997	0	0.001647228	0	0.000785464	0.010747524	2.85E-05
0	0.009785124	0.011881463	0.03907114	0	0	0	0.000222607	0.001258292	0.001513531	0.001256259
0	0.000112913	0	0	0	0	0	0	0	0.004113699	0
0	0.000724351	0.004353415	0.005609019	0.000830279	0.000157035	0	0	6.33E-05	0.000366255	0
0.000334644	0.015775929	0.003681671	0.002832277	0.003788345	0.001354428	0.013942034	0.000203053	0.004208949	0.036792867	0.013392075
0.000108651	0.0068792	0.005932658	0.000972653	0.000966028	0.00050055	0.007347719	0	0.000361727	0.019326624	0.002420597
6.08E-05	0	0.001406463	0.000176125	0	0	0	0	0.000335889	0.000965361	0
0.024555075	0.003634535	0.035917285	0.00049188	0	0.000579067	0	0	0	0.003022211	3.06E-05
0.00341598	0.001141917	0.002953411	0	0	0	0	0	0	0.000339574	0
0	0	0.0001421	0	0	0	0	0	0	0	0
0	0.000210914	0	0	0	0	0.000135019	0	0	0	0
0.194723918	0.003839058	0	0	0	0.293361338	0.000315944	0	0.010571464	0.107518901	0.002978692
0.009287468	0.000117174	0	0	0	0.014888898	0	0	0.000821636	0.005949825	0.000196974
7.39E-05	0.036848138	0.149151278	0.100895698	0	0.000274812	0.003642805	0	0.601377145	0.140270835	0.003355132
0.678506704	0.303110873	0.613003081	0.513993986	0.007797677	0.02540044	0.059632371	0.004143792	0.243392997	0.280893371	0.043726144
0.071474826	0.053254677	0.040198035	0.172959293	0.001139661	0.026960977	0.005630281	0.002531398	0.042354324	0.138861602	0.015121074
0	0.023524349	0.000494119	0	0.024444297	0	0.00367521	0	0	3.40E-05	0.004294043
6.95E-05	0.022448475	0.003381324	0.000153911	0.026376353	0	0.004209884	0	0.000113686	0.00194285	0.003992016
0	0.015162361	0.001406463	0	0.011551296	0	0.000958633	0	5.56E-05	0	0.002346185
0	0.004863801	0.000192157	0	0	0	0.013188629	0.00122584	0	0	0.000919214
0	0.001614876	0.000340716	0	0	0	0.00735042	0.000115816	0	0	0.001234373
0	0.006817417	0.000200231	0	0.004220848	0	0.038547847	0.002854779	2.45E-05	5.09E-05	0.001774959
0.000169495	0.012039132	0.003668753	0	0.000865005	0	0.011166049	0.001249906	9.69E-05	0	0.005151977
4.35E-05	0.017993719	0.000983394	0	0.002550819	0	0.010199315	0.001684591	0	0	0.00229147
0	0.00928873	7.27E-05	0.000153911	0.008716351	0	0.015605464	0.008344739	0	0	0.006808751
0	0.005119454	0.000214764	0	0.01869864	0	0.022677745	0.003176656	0	0	0.001466366
0.000743171	0.001380527	0.001107731	0.00049664	0.001565849	0.591207993	0.000918127	0.000410619	4.39E-05	0.00389055	0.004445057
0.001577609	0.314402219	0.030333417	0.002572056	0.859515534	0.002954224	0.745451894	0.969356998	0.002826636	0.003541274	0.853262773
3.48E-05	0.017147934	0.006770722	0.06879656	0.001483769	0	0.002044184	0.001066406	0.000161485	0.00178519	0
0.000186879	0.01308731	0.004025617	0.001946893	0.003829386	0.001118876	0.000748004	0	0.00151796	0.021119091	0.019828764
0.000134727	0.000948047	0.002614309	0.001880251	0.00054931	0	0.006186558	0	0.000277754	0.000308042	0.006504535

CC54	CC55	CC56	CC57	CC58	CC59	CC60	CC61	CC62	CC63	CC64
0	0	0	0	0	0	0	5.42E-05	0	0	0
0	0.000170016	0	0.000272134	0	0	0	8.87E-05	0	0	0
0	0	0	0.000501688	0	0	0	9.36E-05	0	0	0
0	0	0	0.002056737	0	0	0	5.91E-05	0	0	0
0	0	0	0	0	0	0	0	0	0	0
0	0	0	0.000186976	0	3.52E-05	0	0	0	0	0
0	0	0	6.29E-05	0	0	0	0	0	0	0
0	0	0	0	0	0	0	0	0	0	0
0	0	0	0.000112926	0	0	0	0	0	0	0
0.000182737	0.016451044	0	0.066454267	0.044511286	0.000307403	7.27E-05	0.004464879	0.002028482	0.001936642	9.36E-05
0	0	0	0.00705881	0.000132959	0.000111605	0	0.000945678	0	0	0
0	0.000704351	0	0.001244039	0.006738281	0	6.68E-05	0	0	0.001409975	1.04E-05
0.007352764	0.003343642	0.000417236	0.036552901	0.00623153	0.105709475	0.729711403	0.002090834	0.149362477	0.520098367	0.000357631
0.000932921	0.001125342	0.004777761	0.036780605	0.00562945	0.053411783	0.000933304	0.024779711	0.001107385	0.010919014	0.001712261
0.001274351	0.180985767	0.000335425	0.000212894	0.000496716	0.008532884	0	0.00020933	0.00205953	0.007700953	0.000147627
0	0	0.000237252	0.000968203	0	0	0.000240374	0	0.00154206	0	0.000287976
0	0	0	0.000216596	0	0	0	5.17E-05	0	0.010541638	1.35E-05
0	0	0	0	0	0	0.00015283	0	0.000403626	0.0019947	7.28E-06
0	0	0	0	0.000566959	0	0	0	0	0	0
0.007973109	0.001546333	0.001971644	0.00102374	0.000235815	0.011432655	0.000546035	0.002780391	0.004119059	0.000613754	9.04E-05
0	0	0	0.000359142	0	7.83E-05	0	0	0	0	0
0.000899259	0	0	0.004894701	0.000406404	0.000511033	0.00017212	0.000169926	0	0.000675959	0
0	0	0	0	0	0	0	0	0.001511012	0	0
0	0	0	0	0	0	0	0	0	0	1.98E-05
0	0	0	0	0.000122925	0	0	0	0	8.71E-05	0.000198568
0	0	0	0.003382231	0.00107371	0	0	0	0	0	0.000176736
0.00021159	0.000331935	0	0.006758908	0.00073504	0.008965598	0.000468878	0.002243522	0.00204918	0.000223937	2.08E-05
0.000504931	0.000186208	0.000319063	0.034733124	0.000494207	0.010377303	0.058643816	0.000652616	0.010970359	0.033690112	0.000237034
0.002890132	0.000218592	0	0.013382675	0	0.001754352	0.011698197	0.000248733	0.003611939	0.025918661	0.000229757
0	0	0	0.000303605	0	0	0.001463017	5.42E-05	0	0.000311024	1.46E-05
0	0.001173918	0	0.000149951	0	8.03E-05	0	0	0.000320831	0.000348347	0.000160102
0.008074095	0.00012144	0.00179166	0.01780901	0.022851452	0.044563664	0.083771793	0.000655079	0.049604653	0.037086494	7.80E-05
0.004630943	0	0.000989913	0.008780472	0.006876257	0.018361952	0.006556866	0.000108359	0.023027405	0.008273237	2.91E-05
0.000678051	0	0	0.000199935	0.001304507	0.002400486	0.000316047	0	0.000310482	0.0434065	1.66E-05
0.000476078	0	0	0.007701194	0.001633143	0.006778533	0.0013547	0.000315226	0.012698708	0.033594732	9.67E-05
0	0	0	0.000697921	0.006813541	0.000698999	6.97E-05	0.000142837	0.001604156	0.003201473	0
0	0	0	0	0	5.87E-05	0	0	0	0	0
0.00038471	0	0	0	0.000511768	0.000117479	0.000232955	5.66E-05	0	0.012951037	0
0.000423181	0.056194239	0.000327244	0.100906001	0.001673281	0.010745404	0	0.03882204	0.002018132	0.00043958	2.70E-05
0.000221208	0.007812626	0	0.006044326	0	0.000540403	0	0.001955386	0	0	0
0.020586778	0	0.00059722	0.081995498	0.030450205	0.302621738	0.004298538	0.263890873	0.005267842	0.010773869	0.005124308
0.081332442	0.273490503	0.138636866	0.450384689	0.677919713	0.349955945	0.027283923	0.42190032	0.162371667	0.041204451	0.936523663
0.01099308	0.014799462	0.005694043	0.075092192	0.133526333	0.032347816	0.00267824	0.129038216	0.008817685	0.004346041	0.047704198
0.015951026	0	0.010300001	0.000298051	0.006432223	0.000567815	0.013520291	0	0.073491058	0.001563414	0
0.031983804	0	0.044955127	9.07E-05	0.000115399	0	0.019910973	0	0.043964232	0.013315971	1.56E-05
0.004231807	0	0.010635426	0.000114777	0.000383826	0	0.002707916	0	0.017883756	0.000111969	0.000206885
0.015152754	0.078992536	0.043278002	0	0.000273445	0	0.000157282	0	0.033780427	0.001675382	7.28E-06
0.00877619	0.123107563	0.014071486	4.81E-05	0.000649745	0	6.97E-05	0	0.037226776	0.001463886	0
0.075128998	0.049693162	0.096266966	0	0.000654762	0	0.000535648	0	0.026991224	0.001003571	5.30E-05
0.015388388	0.016232452	0.013523353	0.001375478	0.013815232	0.000399428	0.001925959	0.000142837	0.015741431	0.001173597	0.003334075
0.016552135	0.010889101	0.017033043	0	0.003364123	6.27E-05	0.000559389	0	0.030416874	0.000381523	0.001249628
0.030079491	0.069414984	0.095383407	0	0.00011289	0	0.000151347	0	0	0	0
0.015667303	0	0.033812473	0	0.000544381	0	0.000914014	5.91E-05	0.004160457	0	0
0.042034345	0.087728104	0.000392693	0.001016335	0.000802774	0.010232412	0.000489651	0.040531156	0.001272976	0.000232231	3.01E-05
0.419117187	0.001416798	0.427004164	0.018245905	0.013933139	0.006326239	0.018336672	0.057041112	0.149227935	0.014232455	0.001197647
0.035657781	0	0.014112392	0.003680282	0.003354088	0.003136687	0.001709326	0	0.004294999	0.007767304	0.000103962
0.114811805	0.000372415	0.019806435	0.005366769	0.004249683	0.006905801	0.007963499	0.004489506	0.107312883	0.131098661	0.000245351
0.009444623	0.003497466	0.003329706	0.002482524	0.000378809	0.001869873	0.000316047	0.001864266	0.009428299	0.014232455	0.000178815

CC65	CC66	CC67	CC68	CC69	CC70	CC71	CC72	CC73	CC74	SC01
0	0	0	0	0	0	0	0	0	0	0
0	0	0	0	0	0	0	0	0	0	0
0	0	0	0	0	0	0	0	0	0	0
0	0	0	0	0	0	0	0	0	0	0
0	0	0	0	0	0	0	0	0	0	0
0	0	0	0	0	0	0	0	0	0	0
0	0	0	0	0	0	0	0	0	0	0
0	0	0	0	0	0	0	0	0	0	0
0	0	0	0	0	0	0	0	0	0	0
0.047607373	0.000511529	0.000941161	0	0.000160212	0.004276171	0.000307321	0.001035245	0.003341239	0	0.002180294
0	0.000517619	0	0	0	0.000147212	0	3.29E-05	0	0	0
0	0.000496305	7.49E-05	0	0.000242607	0	0.000173955	0	0.000565185	0	0
0.110554641	0.025999689	0.003582345	0.020144717	0.435997437	0.242732263	0.319330911	0.187948802	0.220322266	0.000181054	0.023012789
0.040019146	0.002152685	0.006354953	0.032150867	0.018959993	0.02060273	0.197792799	0.195741377	0.244054479	5.51E-05	0
0.004681353	0.004567225	0.001431525	0.001850572	0.010606061	0.049862952	0.000150761	0.019873575	0.020479631	0.045271463	0.008691933
0	0.000237496	4.66E-05	0.001571294	0.004101437	0.003526088	0.001082871	0.010674007	0.001540405	0.000241406	0
0.000315203	0.003958262	9.89E-05	0	0.001679941	0	0	0.000183521	0	0	0
0.017709756	0	0.000189363	0.000160797	0.000878879	0	0	0	0	0	0
0.007903431	0.000761204	0.00099486	0.002502222	0	0	0	0.005262497	0	0	0
0.015900256	0.008096167	0.000770169	0	0.001148952	0.003911645	0	0.001077596	0.005596436	0.059690213	0.004771791
0	5.79E-05	0.000329265	0	0	0	0	0.003039857	0	0.00155077	0.000118792
0	0.001385392	9.75E-05	0.001421781	0.001126064	0	4.20E-05	8.16E-05	0	0.016651754	0.001206198
0	0	0	0.000132587	0	0	0	4.08E-05	0	0	0
0.000607058	0.000240541	0.000463514	0.003703965	0.000270072	0	2.90E-05	0.012971939	0	0	0
0	0.000788608	0.000110226	0.005311931	0.000279227	0	0	0.003681395	0.011951993	0	5.30E-05
0	0.002487615	0.000402749	0.014254482	0.000338735	0	1.88E-05	0.006892225	0.030542134	0	0
0	9.13E-05	8.76E-05	0	0.000430285	0.101702757	0.01400049	0.015611814	0.00485948	0.011781654	0
0.010028135	0.019578171	0.000271326	0.000840656	0.027826604	0.008937897	0.016634461	0.013053503	0.013453611	2.62E-05	0.001198887
0.000268507	0.003869962	2.68E-05	0.000995811	0.012858189	0.000119172	0	0.001039951	0	0	0.007112911
0.000338552	0.001924324	0	0	0.000544722	0	0	0	0.000155149	0	0.000151688
0.000245158	8.22E-05	5.65E-05	0	0	0	4.06E-05	0.006608316	0	0	6.40E-05
0.015526681	0.00124533	0.000713643	0.000172081	0.012533187	0.001324912	0.219071862	0.134603862	0.098663505	0.00069273	0.049011832
0.001225791	0.000395826	0.000241649	0	0.001670786	0.000161233	0.017682541	0.006943987	0.010577818	0.00024403	0.01873627
0.000198461	0	0	0.001052231	0.00031127	0.015969043	0	0.00036861	0.012356487	0	0
0.044969005	0.000216182	0.000463514	0.0050524	0.002558821	0	1.74E-05	0.010506172	0.000426659	0.000690106	0.000232102
0.00319873	0	9.89E-05	0	0	0	0	0.001126222	0	0.000123327	0
0	0.000200958	0	0	0.000576765	0	0	0	0	0	0
0.001132397	0	0	0.000138229	0.000297537	0	0	0	0	0	0.000158999
0.006922799	0.000130927	0	0	0.000297537	0.02788624	6.09E-05	0.007632582	0.01690567	3.94E-05	0.000966786
0.000210136	9.13E-05	0	0	0	0.00124079	0	0.000630559	0.00080899	0	8.41E-05
0.010857003	0.00921057	0.013581822	0.009343132	0.003684885	0	0.041969578	0.020588835	0.039479809	3.15E-05	0.004599999
0.411387011	0.644928706	0.773135096	0.757606105	0.101972901	0.135694808	0.05514958	0.061485734	0.049675296	0.038887329	0.067855929
0.073734225	0.042721823	0.053595276	0.039110259	0.005177149	0.049785841	0.009940087	0.010490487	0.004471608	0.024489571	0.007028843
0.004622982	0.000341019	4.95E-05	0.000716532	0.000160212	0	0.000572602	0.000451743	0.001806374	0	0.002048708
0.006887776	0.001440198	0.00013425	0.000939391	0	0	0.005649193	0.002128527	0.004443903	0	0
0.007354744	0.006284502	0.006405827	0.00178851	0	0.000252364	0	4.55E-05	0.000304756	0	0
0.001295836	0.001428019	0.004698737	0.000361087	0.005218347	0	5.22E-05	0	0	0.002141165	0.000890028
0.001412578	0.001963907	0.003010018	0.00150359	0.005840886	0	0	4.55E-05	0	0.003148772	0.001085578
0.001762804	0.001948683	0.001492291	0.001057873	0.000970429	0.000231334	0	0	0.00048761	0.004059291	0.000745649
0.011207229	0.03055778	0.01809261	0.032915356	0.004032775	0.002523642	4.49E-05	0.000161561	0.000725874	0.00285751	0.003682558
0.004296104	0.012361956	0.017561264	0.004034021	0.002146846	0.000154223	2.46E-05	0.000134896	0	0.000881656	0.001102026
0.000875565	0.001881697	0.000747558	0.000200291	0	0	5.51E-05	0	0	0.000209918	0.000933889
0	0.00257287	0.000722122	0.000363908	0.003890872	0.001458104	4.35E-05	4.24E-05	0.000127444	0.005253201	0.004254588
0.002358188	0.006184023	0.000668422	0.003199007	0.000430285	0.229006456	0.001651124	0.007113391	0.023405293	0.014106497	0.000133413
0.046685112	0.100481994	0.0824434	0.026136508	0.266108212	0.039509012	0.049646799	0.204282151	0.104415089	0.72602276	0.675298899
0.022414457	0.037009746	0.002591724	0.006183618	0.006536666	0	0.011452047	0.023107932	0.009364334	0.013450503	0.086740233
0.05346782	0.008495038	0.002359967	0.022717463	0.053799323	0.03337516	0.029340435	0.015065958	0.049165522	0.024830688	0.014962334
0.009817999	0.010102702	0.000862024	0.000366729	0.00433489	0.025607952	0.007971495	0.008192556	0.015525954	0.002390442	0.01088502

SC02	SC03	SC04	SC05	SC06	SC07	SC08	SC09	SC10	SC11	SC12
0	0	0	0	0.000158159	0	0	0	0	0.000153899	0
4.10E-05	0	0	0	0.000700182	0	0	0.000114672	0	3.69E-05	0
0	0	0	0.000464716	3.29E-05	7.07E-05	0	0	0	0	0
0	7.20E-05	0	0.000952669	0	6.56E-05	0.000441466	0.000121839	5.89E-05	0	0
0	0	0	0	0	0	0	0	0	0	0
0	0	0	0	0.000151569	0	0	0	0	0	0
0	0	0	0	0	0	0	0	0	0	0
0	0	0	0	0	0	0	0	0	0	0
0	0	0	0	0	0	0	0	0	0	0
0.000414887	0.002970982	0	0	1.65E-05	0	0.002919083	0.000232927	0.000490799	0.010155274	0.000743263
0	8.12E-05	0	0	0	0	6.76E-05	0	0	0.001372778	0
0	0.000438684	0.000310915	0	0	0	0.000635171	0	0	0.034239422	0
0.306741182	0.055863743	0.037268862	0.013999582	0.011306704	0.115479264	0.154121291	0.004998979	0.026053103	0.149534302	0.001554096
0.081175263	0.068887321	0.039440449	0.096114971	0.053871183	0.087015947	0.079698181	0.066506126	0.000141954	0.031930938	0.169437012
0	0.085724432	0.001193047	0.024502173	0.005586629	0.000139708	0.023537361	0.018412009	0.001467866	0.024332438	0.036771264
0.000116431	0.000162476	0	0	0.008074335	0.000414073	0.001364942	0.005826767	0.000371497	0.000695623	0
0	0.000329593	0	0	0	0.000131291	0.005950786	0.008550225	0.001158285	0.000966485	0.009446201
7.22E-05	0.000174081	7.23E-05	0	0	0	0.000858156	0.000207843	0.000277868	0.000125171	0.002283846
0	0	8.68E-05	0	0	0	0	0.000842122	0	0	0
0.000247621	0.00158994	0.014215337	0.014998722	0.015244199	0.002226905	0.00195056	0.034039641	0.001328932	0.002448018	0.012811157
0	0.000422436	0	0	0	0	0	0.027403004	0	4.10E-05	0.000864888
6.56E-05	0.00219342	0	0	0.000453059	0	0	0.047771602	0	0.00032011	0
3.94E-05	0.000157833	0	0	0.000161454	0	0	0.000498106	9.66E-05	0	0
0	0.005656471	0	0	0.01033304	0	0	0	0	0	0.000648666
0	0.002843322	0	0	0.005194527	0.000350111	5.41E-05	0.000888707	0	0.000613544	0
0	0.000320309	0	0	0.003799105	0.000861811	0	0.000422853	0	0.001588236	0.002500068
0.001598874	0.000178723	0.000443476	0.000406627	0.00093083	0.001457672	0	0	0.000480228	0.002764024	0
0.017020223	0.01827386	0.005936313	0.001254734	0.001728214	0.007874122	0.01331156	0.005217572	0.001792548	0.008173056	0
0.010770674	0.00488123	0.000144612	0.188686479	4.12E-05	0.000595861	0.036141675	0.001250641	0	0.005281809	0.005527176
0	0	0.000209687	0	0	0	0	0	0	0.000205198	0
0	0.001796516	0.001414785	0	0	0	0.00046399	0.001451316	0	0.000178523	0
0.296223049	0.013318355	0.058413513	0.118618863	0.075830539	0.278489407	0.055836477	0.008116621	0.152546263	0.159350998	0.033554961
0.061170474	0.004635196	0.020248057	0.059518554	0.019672644	0.037505597	0.002702855	0.003185729	0.026548432	0.011909721	0
0.001639871	5.80E-05	0.00024584	0	0	0.0602914	0.002130751	0.00021501	5.29E-05	0.011924085	0.004729858
4.76E-05	0.002304832	0	0	0.030348361	0.00626496	0.008083789	0.003060307	0.007233617	0.003102601	0.017135598
0	0.000252998	0	0	0.002721649	0.000804581	0.001626218	9.68E-05	0.000443984	0.000471957	0.001783832
0	0.000266924	0	0	3.29E-05	0.003294069	0.000797342	0.000247261	0	0.000363201	0
0	0	0	0.021969468	0.002789196	0	0.000328847	9.32E-05	0	3.49E-05	0.050812184
0.013194403	0.000858799	0.001325608	0.000418245	0.62021302	0.004475693	0.042563207	0.093493444	0.013532452	0.045414532	0
0.001603794	8.59E-05	0.00024584	0.000534424	0.037495161	0.000479719	0.003727687	0.007460841	0.001522231	0.003679209	0
0.023146782	0.013134989	0.009476891	0.072402816	0.009375849	0.024097034	0.069240385	0.017580638	0.083968855	0.05717651	0.008338063
0.02456855	0.138496497	0.14454428	0.140100379	0.058441312	0.020589196	0.156369165	0.309087391	0.510031925	0.186751154	0.27757507
0.001138071	0.014198044	0.017420897	0.009979785	0.005158282	0.00231275	0.011698857	0.02695865	0.02542337	0.022857061	0.037163167
0.015444307	0.001473886	0.003911748	0.021562841	0.00125209	0.003913496	0.000211724	0.000824204	0.001904299	0.001686732	0
0.010163922	0.001244099	0.000884542	0.048911402	9.72E-05	0.035835839	0.000301819	0.00123989	8.91E-05	0.004957596	0.000824347
0.000145949	0.000875047	0.000195226	0.003264633	0	0.000225552	0.000382904	0.002185933	0	0.000652531	0
0	0.002061119	0.00134971	0	7.08E-05	0	0.003263697	0.00050169	0.000285418	0.000673051	0.005770426
4.26E-05	0.002738874	0.001328018	0	1.98E-05	0	0.004218706	0.001107301	0.000267296	0.000590972	0.005054191
0	0.001088586	0.000884542	0	0	0	0.001137451	0.000688031	0.000309581	0.000219562	0.005419065
0.000565756	0.006280841	0.004210613	0	0	0.000262583	0.003090264	0.006235285	0.006670331	0.004126542	0.014189573
0	0.000784525	0.001019513	0	0	0	0.000734276	0.001591073	0.001504109	0.000246238	0.000878402
0	0	0.000554345	0	0	0	0.000101357	0.000329682	4.53E-05	8.21E-05	0
0.000321415	0.002926881	0.001759443	0.000348537	0	0.000548731	0.001009066	0.00043002	0.000326192	0.000619699	0
0.001169228	0.005988385	0.018341592	0.001986663	0.001784229	0.000138024	0.000695985	0.011488692	0.001081267	0.015515058	0.022365469
0.096283396	0.505466142	0.514077955	0.132943746	0.009950823	0.258822618	0.24229067	0.228877971	0.076279172	0.162197101	0.089353766
0.000167267	0.006914496	0.038534215	0.000441481	0.002392151	0.000220502	0.012883608	0.021260889	0.044413503	0.003250344	0.021473553
0.019803084	0.01569514	0.045314097	0.01161791	0.003571752	0.034581837	0.009000507	0.008009116	0.008622955	0.015422719	0.137003703
0.014857233	0.005832873	0.014976959	0.013999582	0.000998377	0.010163306	0.044056535	0.020877455	0.003178865	0.011567039	0.023987135

SC13	SC14	SC15	SC16	SC17	SC18	SC19	SC20	SC21	SC22	SC23
0	0	0	0	0	0	0	0	0	0	0
5.80E-05	0	0	0	0	0.000126477	0	0	0	0	0
0	0	0	4.81E-05	0	0.000118572	0	0	0	0	0
0	0.000450925	0.000301067	0	0	0	0	0	0	0	4.93E-05
0	0	0	0	0	0	0	0	0	0	0
0	6.10E-05	0	0	0	0	0	0	0	0	0
0	0	0	0	0	4.55E-05	0	0	0	0	0
0	8.50E-05	0	0	0	0	0	0	0	0	0
0	7.62E-05	0	0	0	0	0	0	0	0	0
0.005724041	0.02463316	0.002751944	0	0.003181288	0.009620135	8.20E-05	0.006288992	0	0.000225698	0
0.000124703	0.002191454	0	0	0.000256097	0.000110667	9.32E-05	0	0	0	0
0	0.000235265	0.000268138	0	5.69E-05	0.000498002	7.39E-05	0	0	0	0
0.262074671	0.0195401	0.037054808	0.000127885	0.010209715	0.008681441	0.030665981	0.030689095	0.000696797	0.027648057	0.029975514
0.046459753	0.035888868	0.091331612	0.000139921	0.007042654	0.033611187	0.017254738	0.116184221	0.017141874	0.023587144	0.08049245
0.0067726	0.002391865	0.057635586	0.000103812	0.003474376	0.001831936	0.0004726	0.00130621	0.002466099	0.012917915	0.002154469
0.003324827	0.001187219	0	0.000150453	0.000335771	0.000466383	0.055369802	0.018124812	0.00255713	0.000116168	0.001787172
0	0.000193876	0.017264332	0	0.006749566	0.000622503	0.005692102	0.006241037	0	4.81E-05	0.000136036
0	0	0.00074326	0	0.006208918	0.000100786	0.000787667	0.003126227	0	0	7.31E-05
0	0	0.030892335	0	0.00729306	0.000954504	3.38E-05	0.000182687	0	0	0
0.000491786	0.000557666	0.004770036	0.000240724	0.007534929	0.001420887	0.0348583	0.000399627	8.77E-05	0.002739912	0.007601007
0	0	0	0	0.005235751	0.000401168	4.18E-05	0	0	0.00010953	0
5.27E-05	0.001537939	0	6.02E-05	0.003491449	0.001114576	0.000602807	0.000443015	0	0.002476044	0
0.004571856	6.75E-05	0	0	0	0	0.000128599	0	7.45E-05	0	0
0	0.032915374	0	0	0.000173577	0.001974222	0	0	0	5.97E-05	0
5.97E-05	0.037187184	0	0	0.001863814	0.003768611	0	0	0.010109352	0	0
0.000254675	0.056592224	0	0	0.000546339	0.005035354	0	0	0.002954354	0	0
0.0027944	0.006380485	0.000310476	0	0.000867883	0.000994028	0.009892459	0.004108169	0	6.64E-05	0.009796286
0.014344352	0.014932819	0.002154513	0	0.066104203	0.025247914	0.002202254	0.00429999	0	0.001968223	0.001052578
0.000108896	0.006922903	0.003363487	0	0.096363429	0.000438716	0.109969619	0.003009765	0	0.001048834	0.056840906
0	0	0	0	0.000967476	0.001369506	0	0	0	0	0
0.000890484	0	0	0	0.001445523	0.000968337	0.000181646	8.68E-05	0	4.81E-05	0.00235002
0.162400128	0.022304468	0.02592002	0.00015948	0.008294682	0.022823118	0.276069379	0.153835966	5.30E-05	0.138975462	0.100163243
0.064077997	0.003481057	0.003024786	5.72E-05	0.003411775	0.003804182	0.048012345	0.033678307	0	0.020015799	0.03314515
0.005485174	0.000141595	0.000597431	5.27E-05	0.00030447	9.88E-05	0.003677924	0	0	0	0.185675418
0.00216386	0.004663919	0.014785231	0	0.003295109	0.017137595	0.181039721	0.010917818	0.000261506	0.001510188	0.061920147
0.000310879	0.00095631	0.001862854	0	0.00049512	0.002333891	0.069502805	0.000771852	4.14E-05	0.000411568	0.004519793
0	0	0.005249862	0	0.000125203	0	0.000393834	0.000938553	0	0	0
0	0.000631731	0.011572277	0	0.000133739	0	0.000138244	0.000292299	0.00016551	0	0
0.006449426	0.078491513	0.001241903	0.002327502	0.00013943	0.029674599	0.001506213	0.001226285	0.000539563	0.002374812	0
0.000588387	0.006739918	0.000329292	0.000195588	0	0.002112556	0.000149496	0.000356239	0	0.000124466	0
0.060429997	0.020274215	0.05815775	0.000103812	0.016603591	0.10411405	0.020177145	0.187770034	0.029700774	0.017380438	0.026571215
0.120545602	0.430537886	0.430935614	0.84117016	0.253788806	0.54701179	0.007320484	0.130904208	0.819208432	0.036551527	0.218689634
0.019451904	0.164903628	0.041533186	0.053282801	0.047747773	0.129751276	0.00159784	0.032383514	0.057584249	0.002278558	0.013010135
0.016097219	0.000736294	0.001717025	0	0.003070313	0.001464363	0.015648861	0.019351097	0	0.010329022	0.013537274
0.023298376	0.00109137	0.001524154	0	0.007759725	0.001525625	0.020284846	0.02556473	0	0.009759797	0.011595361
0.000706065	0.000389931	0.005485071	0	0.003898358	0.001782531	0.000120561	0.001603076	0	0.000713605	0.002072847
0.000410993	0	0.003857426	0.004509066	0.000284552	0	0	0	0.001992741	0.001088663	0
0.000159831	0	0.005395692	0.005756318	0.000147967	5.53E-05	0	0	0.003298615	0.001068748	0
9.66E-05	0.000281011	0.000964356	0.000415249	0.00013943	7.90E-05	0	0.000205523	0.000642179	0.001055472	0.000117331
0.003602335	0.004476578	0.00397503	0.006057223	0.003958114	0.004495852	0	0.001514017	0.007070588	0.001306064	0.004057271
0.0001651	0.000287547	0.002206259	0.003648476	0.002578038	0.000482192	0	0.000472702	0.00519536	0.000331909	0.000433614
0	0	0.000376334	0	0.001533734	0	5.30E-05	0	0	6.80E-05	0
0.000337225	0	0	0.000153462	0.000193495	4.94E-05	0	0.001100688	0.000153924	0.001714312	0.000409808
0.007533112	0.00072758	0.019014287	0.002515568	0.012702387	0.001891222	0.005086081	0.00735771	0.000226749	0.001243001	0.003281866
0.103410362	0.005681224	0.021888539	0.049184471	0.284267137	0.019003126	0.024735971	0.169334655	0.037378785	0.620808399	0.104666032
0.003393325	0.001439911	0.051313171	5.42E-05	0.092012634	0.005189498	0.016287032	0.003107959	0	0.029128373	0.00030438
0.033411609	0.007413039	0.028394417	0.029284101	0.016037333	0.003628301	0.028275651	0.015334271	0.000398879	0.01821685	0.004336145
0.017367082	0.000320222	0.009836436	0.000201607	0.007674359	0.00197027	0.011519234	0.007487874	0	0.010485019	0.019184465

SC24	SC25	SC26	SC27	SC28	SC29	SC30	SC31	SC32	SC33	SC34
0	0.000125401	0	0	0	0	0	0.000115374	0	0	0
0	0.000108398	0	0	0	0	0	9.32E-05	9.68E-05	4.25E-05	9.35E-05
0	4.89E-05	0	0	4.92E-05	8.93E-05	0	0.000448182	0	4.92E-05	0
0	2.98E-05	0	0	0.000100184	0	0.000110606	0	0.000225096	3.58E-05	6.32E-05
0	0	0	0	0	0	0	0	0	0	0
0	4.68E-05	0	0	0	9.06E-05	0	0.000678929	0	0	0.000260317
0	0	0	0	0	0	0	0	0	0	0
0	0	0	0	0	0	0	0	3.60E-05	0	9.10E-05
0	0	0	0	6.68E-05	0	0	0	0	0	6.32E-05
0.003793751	0.006799309	8.97E-05	0.000158402	0	0	0.004672575	0.002085598	0.000168822	0.001427338	0.002322631
0	0.000248677	0.000108844	0.000128434	0	0	0.001016322	0	0.000285872	7.61E-05	0
0.000335248	0.000303939	0	0.000145559	0	0	0	0	0	0	0
0.09375779	0.032493852	0.050167371	0.000308242	0.026891406	0.001476666	0.037996239	0.000332808	0.031722772	0.008407422	0.008416062
0.436120861	0.020706116	0.028686983	0.079838858	0.02208259	3.28E-05	0.047291305	0	0.043938729	0.017087784	0.000737985
0.029920199	0.002828972	0.011516421	0.00241884	0.028279917	0.00103787	0.004025636	0.017971645	0.007090522	0.002040332	0.001195435
0.001767934	0.00142405	0.00012412	0	0.010037701	0	7.72E-05	8.87E-05	0	0	0.000166805
0.000475652	0.000350699	0	0.003373533	0	0	0.001350226	0	0.0002341	0	0.007364686
0.000610324	0.006318958	0	0.000321085	0	0	0.000770067	0	0.000213841	0	0.03955549
6.88E-05	0.000250803	0	0	0	0	0.00049877	0.000115374	0	0	0.003674761
0.014834023	0.007058613	0.000509847	0.180402683	0.022873514	0.000398069	0.000137736	7.10E-05	0.000130556	4.47E-05	0.000465032
0	0.000227423	0	0	0	0	7.51E-05	0	4.28E-05	0	0
0	0.003364585	4.77E-05	0	0.00260126	0.000115611	0.000191995	0	0.000542481	9.84E-05	0.000169332
7.74E-05	5.74E-05	0	0	0	0	5.43E-05	0	9.45E-05	0	6.57E-05
0	0	0	0	0	0	0	0	0.000432184	0	7.83E-05
0.000220634	0.001100982	0	0	0	0	0	0	0	0	0
0.000297999	0.002484649	4.01E-05	0	0	0	0.00014817	0	3.38E-05	0	0
0.003816674	0.042498337	0.003116365	0.001108814	0.001223295	0.000114297	0.001431615	0	0.009071367	9.40E-05	6.32E-05
0.004724996	0.078210118	0.004447313	0.000115591	0.002778779	0.000102473	0.004499362	0.000124248	0.005609391	0.000711432	0.00099072
0.023799768	0.023169511	5.35E-05	0.000132715	0.000418311	0.000332381	0.03066495	0	0.016146132	0.000472051	0.002984795
0	0.012423245	0	0	0.000166973	0	0.001099798	0	0.000252107	0	7.08E-05
0	0.041418609	0	0	0.00011776	0	0.000569724	0	0.000123803	0.000138707	0
0.120018911	0.056020438	0.055584729	0.014915469	0.011255723	0.112869068	0.046226984	0.179277141	0.434495954	0.042990161	0.041701207
0.001836703	0.01010013	0.022316766	0.010231909	0.00901653	0.04780116	0.006974429	0.001459919	0.039713678	0.002190225	0.007119533
0.008885514	0.008055449	0.001317581	0.014483075	0.008977863	0	0.007827972	0.007175346	0.000171073	0.002583974	0
0.037977621	0.027037827	0.001464615	0.000659295	0.002297194	0.000465071	0.022728458	0.000244059	0.032391307	0.035001096	0.003176874
0.003621828	0.001940534	0.000156582	0	0.000344491	3.28E-05	0.003706339	0.000860864	0.002386017	0.005991239	0.000179442
0.000532959	5.10E-05	0	0.000196932	6.50E-05	0	0	0	0	0	0.000179442
0.000796573	0	0.000101205	0	0.000724135	7.23E-05	0.000156518	0	0.000119301	0.000161079	0.001213126
0	0.066898482	0.001185823	0.001716735	0.000108972	0.0001432	0.013468871	0.232411085	0.05790143	0.01025088	0.023645848
0	0.005324248	5.16E-05	0	6.15E-05	0	0.001254229	0.016880034	0.004801297	0.000666688	0.003336097
0.010091835	0.035197422	0.048097432	0.134594555	0.031462945	0.000547838	0.151509144	0	0.026007586	0.003454247	0.016513678
0.052393301	0.087039229	0.119538961	0.042306161	0.105370372	0.00129274	0.482281152	0.237234585	0.048521682	0.79769165	0.232192321
0.011581828	0.008639947	0.01461942	0.005488413	0.012150346	0.000462444	0.083532459	0.178030219	0.006284679	0.049184986	0.018527973
0.000553017	0.000352824	0.011121147	0.002286125	0.000871774	0.002502713	0.000300514	0	0.00875173	8.73E-05	0.004261105
0.009243685	0.003779047	0.017395887	0.00014984	0.000230247	0.000168161	0.003188787	0	0.015139953	0.000163316	0.0034473
5.73E-05	0.001309276	0.002052753	0	7.21E-05	0	0.001325183	0	0.000308381	0.000105149	0.001486079
0.000338114	3.83E-05	0	0.000273993	5.10E-05	2.89E-05	0.000141909	0	8.78E-05	0.001633161	0.00098061
0.001959913	0.000148781	0	0.000158402	8.79E-05	0	6.68E-05	0	0.000218343	0.001445235	0.002499545
0.000209172	0.000208294	0.000322712	0.000154121	0.000613405	2.89E-05	7.10E-05	0	0.000148563	0	7.83E-05
0.000449863	0.000212545	0.006303384	0.000393864	0.002172404	0.000162906	0.005816198	0	0.000605508	0.001228226	0.000859298
0	0.000278434	0.000374269	0	0.001031716	0	0.000377729	0	8.78E-05	0.001319952	0.00303787
0	0.000255054	0	0.00033821	0.000435887	0.000289027	0	0	0.000110297	0	0.000576235
0	0.000257179	0.003072446	0.001524084	0.00085244	0.000475581	0	0	0.000274617	0	0
0.000876803	0.008860994	0.005927205	0.024582268	0.006336178	0.002144056	0.000671983	0.000945175	0.002692148	0.000161079	0
0.016260978	0.316081779	0.555030008	0.262801659	0.507254528	0.691893049	0.016321667	0.083312995	0.134947271	0.002447504	0.030911967
0.058691385	0.047055298	0.00165175	0.036757812	0.026944134	0.038761177	0.003478867	0	0.01412252	0.000845664	0.209484118
0.04275706	0.021220475	0.011596622	0.09132942	0.091397386	0.091553311	0.010920762	0.039138249	0.030601794	0.008975673	0.279377869
0.006243642	0.009539012	0.021808829	0.086204904	0.062126179	0.004515393	0.00097041	0.000905238	0.022617641	0.000695771	0.046321195

SC35	SC36	H002	H003	H004	H005	H006	H008	H009	H010	H011
0	0	0.007234204	0.011507954	0.002229053	0.001024829	0.021390831	0.00259256	0.007214765	0.062575589	0.008393219
0.000169313	0	0.000422834	0.001908409	0.000402668	5.06E-05	0.001102008	0.00038904	0.002154515	0.007181837	0.003589858
0	0	0.003066267	0.007489106	0.000686692	9.46E-05	0.008051709	0.000547768	0.013134915	0.024084326	0.032185386
0.000822895	0	0.001769	0.005286976	0.000327167	0	0.004842157	0.000774967	0.007927852	0.015378413	0.020693437
0	0	0.000480363	0.001627121	0.000111453	0	0.001250427	0.000199188	0.001714079	0.004530929	0.005545559
0	0	0.004176566	0.013386835	0.000744216	0.000195729	0.008868011	0.000547768	0.019760525	0.037264137	0.050887884
0	0	0.001340413	0.0050865	0.000273239	4.40E-05	0.004048117	0.000516645	0.007254805	0.013502325	0.016597035
0	0	0.001231109	0.003308634	0.000172572	4.40E-05	0.004916366	0.00051042	0.006829622	0.01163607	0.01825762
0	0	0.00051488	0.001784083	0.000237286	9.46E-05	0.001217033	0.000177402	0.003430064	0.005850483	0.006129186
0.043003568	0	0.005163179	0.038499195	0.002797102	0.000530008	0.017862178	0.000314344	0.002699817	0.001958683	0.040748188
0	0.000102741	0.008137401	0.000281288	0.000179762	0.000316685	0.019465099	0	0.000289811	0.001366752	0.001482193
0.000114696	0	0.001130434	0.001487253	0.000151	3.74E-05	0.00646734	0.000361029	0.000142999	0.001095368	0.009346844
0.000544349	0.05370183	0.107247436	0.084848042	0.223538083	0.327010622	0.106182377	0.050730772	0.311884152	0.038896373	0.021017185
0.001898849	0.206473461	0.103286601	0.07888815	0.216836542	0.00955774	0.091748668	0.033323581	0.038802242	0.077688518	0.025325014
0.000790125	0.003590001	0.019709251	0.022882257	0.065282497	0.001361307	0.010563694	0.038343749	0.056194707	0.028033156	0.024096094
0.000731867	0.127726583	0.004044251	0.000435142	0.020395837	0.001609817	0.013602565	0.001185793	0.025053386	0.000318581	0.008679527
0	0.001784134	0.007918793	0	0.020794909	0.001213961	0.016849222	0.0006318	0.01996835	0.002605677	0.000224641
0	0.000387254	0.021832051	0.000234666	0.004573154	0.002504893	0.011324339	0.000329906	0.009821156	0.000574232	0.000475711
0	0.000106692	0.00012081	0	0.000679502	0	0.003291182	0.002816647	0.005702791	0.007136606	0.000449283
0.000100131	0.000189675	0.204418761	0.093731157	0.141048734	0.097235601	0.022548496	0.180981251	0.105693258	0.202308729	0.198726151
7.46E-05	0	0.015420504	0.017508252	0.001100146	0.006738361	0.005895929	0.086702936	0.002114475	0.01376781	0.005171157
0.0001948	0	0.04459894	0.003142347	0.01367632	0.028086033	0.031553794	0.0014908	0.050036226	0.011286025	0.00156368
0	0	0.000934838	0.000257977	0.000647144	0.000145147	0.004404322	4.05E-05	0.00548162	0	0.002349925
0	9.68E-05	0.004392298	0.087443354	0.000884431	0	0	0.009022608	0.000209732	0	0.014352824
0	0	0.00239606	0.068774205	0.000503335	6.38E-05	0.028718999	0.001587282	0.003422437	0.002468019	0.003440097
0	0	0.002278127	0.00938819	0.000129429	0	0.035820829	0.000115156	0.002884762	0.000239919	0.004431162
9.28E-05	0.000774508	0.019666105	0.001669081	0.005227489	0.046513162	0.014448551	0.000513532	0.024336486	0.003400163	0.005404608
0.000169313	0.002141751	0.029025986	0.00614172	0.014665013	0.023694223	0.047809341	0.005490128	0.021733908	0.042302436	0.020999566
0.004338407	0.138216024	0.021403464	0.0010288	0.018587427	0.005333069	0.06279962	0.000700271	0.007607535	0.004121887	0.007177513
0	0	0.013691774	0	0.001402146	0.003257021	0.042904107	0	0.001266016	0	0.004439971
0	0.000784387	0.011264073	0.002155507	0.001258336	0.001939698	0.038588836	0	0.000741687	0	0.002600995
0.068016312	0.151084311	0.018115713	0.002788017	0.036204138	0.070787975	0.01723882	0.003638299	0.045689063	0.002055044	0.001314813
0.019835057	0.047211377	0.004029869	6.84E-05	0.001024645	0.017246157	0.004422874	0.000666036	0.013165421	0.000316614	0.001121005
0.001587533	0.046320298	0.000569532	0.00011811	0.000291215	0	0.005127863	0	0	0	0
0.007324133	9.68E-05	0.0007076	0.000341897	0.019701954	0.012313343	0.037483117	8.40E-05	0.006865848	0.000521135	0.002037189
0.001290781	0	0	0	0.000399072	0.000972048	0.002367277	0	0.000545302	0	0.000387617
6.74E-05	0.000102741	0.000138068	0	0.011972173	0	0.00391083	0	0.000701647	0	6.39E-05
0.000112875	0	0.00126275	0	0.000237286	5.50E-05	0	0	9.91E-05	0	0
0.062414433	0.000920716	0.000293395	0.000951096	0.000190548	0.000127554	0.000897933	6.85E-05	4.96E-05	0.002690239	0.000682734
0.003896009	0.000100765	0	0	0	0	0	0	0	0	0
0.027201063	0.030567366	0.002096912	0.021808388	0.002193101	0.018941743	0.00869733	0.00617795	0.007632322	0.01185829	0.003081111
0.533997233	0.054579079	0.089212263	0.230491648	0.033399845	0.055767412	0.072940321	0.084583447	0.053807581	0.183268601	0.118513755
0.05086841	0.00435463	0.018492524	0.018411172	0.004774488	0.003637483	0.02361711	0.017323158	0.005313835	0.024969273	0.029606415
0.000842922	0.004550232	0	0	0	0.00102263	0	0	0.000257398	7.08E-05	0
8.37E-05	0.003163231	0.000158203	0.000132097	0	0.000365068	0.000233759	4.98E-05	4.19E-05	0	0
0	8.69E-05	0	0	0	0.000153944	0	0	0	0	0
0.001824206	0	0	0	0	0.000351873	0	0	0	0	0
0.002204704	0.000100765	0	0	0	0.000323283	0	0	0	0	0
7.10E-05	0.000286489	0	0	0	0.000349673	0	0	0	0	0
0.002414069	0.003785604	0	0	0	0.000268303	0	0	0	0	0
0.001168803	0.000162014	0	0	0	0.000107761	0	0	0	0	0
0	0	0	0	0	0.000301291	0	0	0	0	0
0.000134722	0.000296368	0	0	0	0	0	0.000130717	0	0	0
0.000628095	0.002829324	0.017301685	0.004876699	0.006065182	0.002377339	0.017320451	0.037531434	0.010644829	0.01047974	0.013106284
0.114766604	0.007075285	0.123660306	0.14095495	0.088371173	0.137610785	0.088071597	0.301901003	0.043706147	0.111890739	0.245004482
0.001194291	0.006956738	0.033320485	0.000975962	0.008168401	0.025702096	0.016329756	0.064085726	0.025244051	0.018587821	0.00099547
0.042568453	0.059180682	0.015268054	0.004551897	0.020190908	0.040197049	0.008144471	0.027836566	0.013167328	0.00512286	0.004208723
0.002441378	0.040112383	0.007055866	0.003347486	0.007273185	0.052323459	0.004660344	0.034985559	0.007559869	0.006595805	0.015095021

H013	H014	H015	H016	H017	H018	H019	H020	H021	H023	H024
0	0	0.000243071	0.000151643	0.000652328	0.002798961	0.001070155	0.003407092	0.003098123	0.0009686	0.000250229
0.000113138	0	4.29E-05	0	0	0.00018958	0.000105104	0.000703235	0.000432766	0.000179984	0
0	0.000557012	0.000300265	0.000909859	0.000187065	0.003627572	0.000243651	0.002102742	0.000250762	0.000258135	0.000184379
0	0.00060564	6.43E-05	0.000178722	0.000172675	0.001580539	0.0001696	0.000784467	0.000208294	0	0.00108652
0	0.000190091	5.48E-05	0	0	0.000359988	0	0.000322606	0.000141559	9.24E-05	0
0	0.00091067	6.20E-05	0.00019497	0.000177472	0.004690495	0.000554187	0.00255764	0.000766442	0	0
0	0	6.43E-05	0	0	0.00140374	0.000131381	0.000719481	0.000254806	0	0
0	0.000207774	0	0	0.00012471	0.001684915	3.82E-05	0.000902833	7.68E-05	9.24E-05	0.000269984
0	0	0	0	0	0.000937247	0	0.000394554	2.83E-05	0	0
0.001680519	0.00078689	0.007930796	0.002469617	0.002518179	0.002215311	0.002056704	0.082656789	0.011913212	0.016054128	0.000256814
0.001815733	0	0.000293115	0.000384524	0.006259473	7.03E-05	0	0.001845121	0.008778688	0.002822912	0.000737517
0	0.000137043	0.005600172	0.000400771	0.032659581	0.002110936	0.000895777	0.00155965	0.032973166	0.000918867	0.006525704
0.244309966	0.059379241	0.284510164	0.15578627	0.18462328	0.182535253	0.133989122	0.139665232	0.167753639	0.145216561	0.005050671
0.052758369	0.046444186	0.102838215	0.029960356	0.179160031	0.130699101	0.034715537	0.066347774	0.015915291	0.068791903	0.044948999
0.011603585	0.016944657	0.041248242	0.02255692	0.051294104	0.076920291	0.032625391	0.058257092	0.009156854	0.025560142	0.036026366
0.008915858	0.004049388	0.021156733	0	0.013617352	0.070774933	0.001335305	0.002156123	0.008978894	0.044778205	0.010107928
0.002077883	0.001852286	0.018716488	0.00019497	0.011454116	0.013577302	0.000647348	0.004105685	0.004891879	0.00741488	0.004681913
0.006321953	0.007126216	0.006758335	0	0.001074423	0.016282537	5.26E-05	0.001123319	0.004099148	0.014595308	0
0.000811285	0.023885202	0.021542788	0.018435476	0.011554844	0.001508116	0.003296459	0.009042254	0.001193141	0.00339602	0.024021967
0.158015718	0.170206934	0.121597598	0.183098286	0.080754398	0.097925276	0.22181826	0.200844346	0.054405219	0.109352317	0.303277339
0.001134144	0.012749384	0.005573958	0.002994952	0.001798699	0.003915137	0.045354978	0.012702325	0.008426813	0.025460677	0.000533383
0.035169487	0.003620578	0.01393132	0.033811009	0.009924023	0.043153836	0.029252492	0.012827654	0.024750603	0.018050533	0.001119445
0.000353213	0	0	0	0	8.31E-05	0	0	0.000268962	0.000935445	0.000276569
0.00403159	0	0.000590997	0.001733065	0.000498839	7.46E-05	0.001509683	0.006041321	0.007454099	0.018550226	0.007032747
0.002820182	0	0.001703882	0.000157059	0.000623549	0.001572019	0.00056852	0.00688149	0.004198239	0.014216393	0
0.000115898	0.000366921	0.000121536	0.000200386	0.000335757	9.16E-05	0.000527911	0.000608078	0.000835199	0.009546274	0
0.006490281	0.000349238	0.014129114	0.004435562	0.002537365	0.002732927	0.011800368	0.018304995	0.017181234	0.003542849	0.000928481
0.024661413	0.005132467	0.019908014	0.028768874	0.059424224	0.017383803	0.012058352	0.018964133	0.044572926	0.021929669	0.003806112
0.04243518	0	0.000955604	0.012559303	0.036424858	0.016474247	0.00138308	0.003003254	0.035027796	0.016880635	0.001639657
0.009782333	0.001220121	0.000436098	0.013257945	0.040319641	6.82E-05	0.005850021	0.0115117	0.103360412	0.003969128	0.001079935
0.002425577	0	8.34E-05	0.002003856	0.00754974	0.000104375	0.000752453	0.000900512	0.006525876	0.002721079	0.003081766
0.031322781	0.000632164	0.04967948	0.026326336	0.006801481	0.033689771	0.024248085	0.014798104	0.037834712	0.018635482	0.001284069
0.007574754	0.000159146	0.010347211	0.005377916	0.00117515	0.002098155	0.006174889	0.003474398	0.00976758	0.00283949	0.000487288
0	0.000406707	4.77E-05	0	0.000681107	0.000438802	0	0.000392233	0.001225498	0.000532848	0.002713007
0.000110379	0.009168593	0.001970784	0	0.002815564	0.024008435	0.000570909	0.009016724	0.00604053	0.004097012	0.005445769
0	0.000972561	6.91E-05	0	0.000738666	0.00279683	0	0.000816959	0.000479279	0.000592054	0
0	0	5.72E-05	0	0.014068226	0.005725727	0	0	0	0.017671619	0.000888971
0	0	0.001441746	0	0	0	0	0	0	0.000395492	0.001185294
0	0	5.00E-05	0	0.000215844	5.33E-05	0	0.000246016	0.000483323	0	0
0	0	0	0	0	0	0	0	0	0	0
0.004117134	0.000941615	0.004358602	0.00350404	0.013248019	0.016689388	0.005938404	0.012235823	0.014594747	0.016335945	0.003898302
0.075330861	0.019959595	0.059030575	0.08750352	0.091306767	0.068495718	0.041951026	0.09644994	0.117795399	0.09787358	0.057328741
0.008926896	0.005539174	0.007060982	0.013853686	0.012288713	0.011928599	0.009810549	0.022542972	0.01128833	0.010216479	0.008007322
0.00293608	0	0.001389319	0.004208098	0.000378926	7.88E-05	0.00094594	4.41E-05	0.000368054	9.71E-05	0
0.000590527	0.001122865	0.000576699	0.002783735	0.000158286	0	5.02E-05	0.000215844	0	0.000793352	0.000283154
0	0	0.00017158	0.000167891	0.00012471	0	0	0	0	0	0.000243644
0.000118657	0.000764786	0	0.00038994	0	0	4.78E-05	0	0	0	0
0	0.001396951	0	0.000357445	0	0	4.54E-05	0	0	0	0
0.000482908	0.000216616	0.000152515	0	0	0	0.000126603	0	0	0	0.000474118
0.001164498	0.000406707	0.000245454	0.004105197	0.001275877	0	9.55E-05	0	0	0.000326814	0.002568138
0.000452554	0	7.63E-05	0.000509088	0.000724276	0	0	0	0	5.45E-05	0.000381928
0	0	0	0	0	0	0	0	0	0	0
0.000124176	0.000198933	0.000190644	0.000888196	0	0	8.12E-05	0	0	0.000108938	0.002614233
0.003992958	0.133581189	0.012730262	0.039297242	0.001611634	0.008739829	0.043776022	0.005779059	0.003652226	0.009934661	0.015250789
0.203356623	0.243374431	0.099451898	0.057359026	0.071904798	0.067963192	0.245968407	0.093003393	0.168803198	0.204869523	0.3499516
0.007497489	0.151012126	0.011746062	0.096591278	0.013583776	0.040003408	0.020533596	0.037410703	0.022865844	0.013044127	0.037389455
0.025497533	0.054481073	0.043712318	0.119326921	0.018620134	0.014190772	0.03497591	0.022134492	0.015031558	0.016167802	0.026642785
0.008559886	0.018942827	0.004985344	0.022806048	0.012557319	0.005553189	0.021856958	0.010195745	0.011850522	0.009117627	0.026036968

H025	H026	H027	H028	H029	H030	H031	H032	H033	H034	H035
0.001200795	0.003335273	0.007716401	0.001224463	0.007709724	0.001829282	0.007953456	0.004265665	0.000873963	0.000349023	0.002841663
8.51E-05	0.001639138	0.008056419	0	0.001199792	0.000187549	0.000652132	0.000744423	0	0.00010887	0.000277533
0.000148918	0.002712243	0.018860981	0.000129867	0.004841515	0.000152214	0.00125951	0.000971886	0.000160082	0.000163305	0.001282937
0.000505847	0.002277891	0.012100629	0.000247366	0.00195919	0.000440332	0.000882297	0.00066171	0.000194695	0.001104707	0.000284515
4.02E-05	0.000581756	0.003088161	0	0.000999356	5.44E-05	0.000249345	0.000192014	0.000181715	0	9.08E-05
0.000127644	0.005369455	0.025425322	0	0.006120352	0.000184831	0.001178527	0.001586331	0	0	0.0053255
0.00018201	0.001175305	0.006716349	0	0.001388936	0	0.000545575	0.000321993	0	0	0.004156019
0.000139462	0.001845504	0.007904411	0	0.002142688	0.000225602	5.11E-05	0.000425385	0.000190368	9.93E-05	0.005334227
0.000111097	0.000713438	0.003876202	0	0.000976772	0.000146777	0.000379345	0.000443109	0	8.97E-05	0.000167567
0.0036993	0.002376161	0.004380228	0.001629526	0.028230405	0.037183613	0.028297423	0.011946224	0.002275766	0.000883766	0.001886878
0.00734187	0	0.000292015	0.000763744	0.004474519	0.01402268	0.018370522	0.099480971	0	0.000419468	0.001450505
0.000245832	0.000279086	0.001640085	0.001379067	0.000383934	0.005591132	0.006314601	0.167146702	0.03844573	0.000371438	0.00056554
0.197398435	0.106359227	0.071967742	0.107165275	0.03914428	0.052296251	0.034535303	0.192988831	0.144766388	0.047134166	0.112572089
0.184775903	0.016004198	0.140139287	0.082029764	0.142704699	0.065566017	0.061828954	0.009594791	0.044559146	0.106183157	0.118833173
0.04604624	0.006383598	0.137295139	0.051968572	0.030926409	0.004294599	0.020949215	0.012274125	0.007333504	0.030307397	0.074413863
0.018643054	0.000685922	0	0.003546615	0.000355703	0.006980082	0.046207617	0.020223504	0.02026989	0.00426513	0.029799059
0.008039182	0.016597747	0.135807062	0.024099664	0.002326185	0.008146147	0.000157705	0.001140268	0.016999018	0.027121358	0.016126611
0.011301184	0.00581953	0.002184114	0.000145328	0.000584369	0.00087251	0.000447542	0.018767152	0.011651401	0.001902017	0.004567956
0.071314942	0.000624995	0.003888202	0.007519936	0.001072755	0.006727298	0	0.015600398	0.002578624	0.013813641	3.49E-05
0.081453151	0.30573659	0.105289475	0.092558293	0.247865781	0.189848439	0.026577585	0.065976007	0.042897751	0.042974704	0.139292098
0.000624035	0.0286063	0.018512963	0.074707721	0.035338821	0.007534574	0.016188223	0.011721716	0.000930208	0.020646814	0.000384008
0.011213725	0.007008593	0.000680035	0.035197135	0.007224161	0.024199248	0.002158856	0.02182756	0.018764251	0.018866475	0.009750325
0.000222194	0	0	0.000126775	0.000293596	0.0003153	0.001101805	0.003757566	0.000177389	0	0.000607432
0.008346472	0.019139	0.001448075	0.044244559	0.000366995	0.0021174	0.000726723	0.005772236	0.108808425	0.003077169	0.000471283
0.003581112	0.001334502	0.00019201	0.001991299	0.005493637	0.030660172	0.000419837	0.016672722	0.042482402	0.000505924	0.008184617
0.004325699	0.000389148	0.000588031	0.000816309	0.000400872	0.00201683	0.000475247	0.005981974	0.022636513	0.000826129	0.000265315
0.002968895	0.031135762	0.001816094	0.000225722	0.000465802	0.005131774	0.003431153	0.009355512	0.02202647	0.001408902	0.005246952
0.028507066	0.00944175	0.004668243	0.008342429	0.015320641	0.059249152	0.031347101	0.050526266	0.01280659	0.008386167	0.084989806
0.010818975	0.003588808	0.000136007	0.007532304	0.001329652	0.007099678	0.013313301	0.0031638	0.015895747	0.004441242	0.041748705
0.017255521	0.000357702	0.000168009	0.001515119	0.020447283	0.04760481	0.005941649	0.002404606	0.008570897	0.003643932	0.040697918
0.002626148	4.13E-05	0	0.00053493	0.001177208	0.002739845	0.007135094	0.003701439	0.003777079	0.000870957	0.014327008
0.016203644	0.010927587	0.010128527	0.014201919	0.002631074	0.007300818	0.035195959	0.010445561	0.086327667	0.002587256	0.011844916
0.001690096	0.003712628	0.001104057	0.004161938	0.000784805	0.002408237	0.005189353	0.007423556	0.021360181	9.61E-05	0.000684233
0.000387658	0.000434352	0.003624188	0	0	0	0	0	0.000398043	9.93E-05	0
0.0104455	0.000165093	0.028549485	0.001156438	0.000626715	0.001562908	0.006235748	0.000508099	0.015454439	0.001517771	0.000830855
0.000789499	0	0.000992052	0.000126775	0	7.34E-05	0.001001641	0	0.010989439	0.000105668	0.000202477
0.00026947	0.000182782	0	0	0	0	0	0	0.038852426	0	0
8.98E-05	4.91E-05	0.000308016	0	0	0	0	0	0.000765799	0	0.000155349
0.000134735	5.31E-05	0.00058003	0	0.000412164	7.61E-05	0.000854592	0.00066171	0.000385063	0	0.000277533
0	0	0	0	0	0	0	0	0	0	0
0.026826426	0.008529807	0.007724402	0.007300399	0.015975586	0.010595155	0.034916779	0.01165968	0.01032315	0.006378482	0.01093726
0.051251262	0.041628997	0.108437639	0.054655589	0.10531635	0.178391646	0.465251582	0.112020962	0.101855658	0.069900736	0.074845
0.017217701	0.005811668	0.0115326	0.004282529	0.024870987	0.014134122	0.05158238	0.016501387	0.010401028	0.011415306	0.010101169
0.00020092	0.000650545	0.000140007	0.000355589	0.000169382	0.000809994	0.000247214	0.000410615	0	0	0
0.000130007	0	0	0	0	0.000108724	0	0.000747378	0.000194695	0	3.32E-05
0	0	0	0	0	0.00015765	0	0	0	0	0
0	0	0	0	0	0	0	0	0	0.000131284	0
0	0	0	0.000166972	0	6.25E-05	0	0	0	0	7.16E-05
3.31E-05	0	0	0	0	8.15E-05	0	0	0	0	2.79E-05
0.000267106	0	0	0	0	0.000940463	0	0	0	0	0.000155349
0	0	0	0	0	6.80E-05	0	0	0	0	5.24E-05
0	0	0	0	0	0	0	0	0	0	0
4.73E-05	0	0	0	0	0.000252783	0	0	0	0	6.11E-05
0.014549004	0.044486679	0.020629073	0.037296657	0.028103368	0.017607854	0.0009441	0.004005707	0.001843111	0.017720141	0.0024786
0.071544227	0.227842155	0.043554265	0.250415112	0.160670529	0.113347504	0.046955651	0.013910675	0.079820535	0.295395453	0.115307277
0.041044503	0.042537008	0.010436543	0.041291623	0.032241946	0.039124337	0.004515909	0.01886759	0.010855316	0.211424912	0.025093209
0.019420735	0.014372922	0.011800614	0.019742925	0.008054135	0.019738845	0.004362466	0.03397466	0.016375995	0.030601985	0.01123923
0.004167327	0.017055684	0.015620812	0.015203753	0.006876927	0.009738954	0.003669842	0.009225534	0.003543445	0.012660903	0.009624649

H036	H038	H039	H040	H041	H042	H043	H044	H045	H046	H047
0	0.000183165	0.000312898	0.009176607	0.003668972	0.001581935	0.003708639	0.002468946	0.016058662	0.002349077	0.007060433
0	0	7.40E-05	0.000476808	0.000178394	0.000424309	0.000499455	0.000246454	0.000948042	0.000195123	0.000560708
0.000130507	0	0.000150478	0.000560185	0.000315163	0.000451982	0.000400682	0.00031687	0.001631663	0.000169782	0.006537853
0	0	0	0.000432515	0.000178394	0.000110689	0.000216182	0.000215648	0.000599781	7.86E-05	0.001718009
0	0	0	0	0	0	0.000150955	5.28E-05	0.000635252	0	0.000580893
0.000206473	7.68E-05	0.000186306	0.000698277	0.001444992	0.00017987	0.000383909	0.000440097	0.002363655	0.000780492	0.017911251
0	0.000254068	5.49E-05	0.000164147	0.000558968	0.000142974	0.000178909	5.50E-05	9.03E-05	0.000304088	0.005405224
0.000251274	0.000112263	0	0.000268368	0.002152622	0	0.000423046	5.94E-05	0.00100286	0.000438393	0.000980117
0	0	0	0.000101615	0	0	4.47E-05	3.96E-05	0.000316014	8.11E-05	0.00065715
0.000210369	0.001293974	0.001722132	0.00594838	0.000915756	0.003809559	0.005315095	0.012575779	0.083130988	0.005990528	0.001273928
0	0.000168394	0.003609074	0.000484625	0.00020218	0.004722746	0.001779774	0.000125428	0.048820914	0.000146976	0.003133235
0.000237639	7.98E-05	0.000886146	0.004158394	0	0.000396637	0.000339182	0.000809779	0.017209856	0.0002306	0.000921804
0.163963037	0.091257696	0.35194558	0.062446261	0.0570207	0.057078815	0.163176384	0.253467416	0.008964474	0.236967942	0.186282843
0.046320103	0.013885114	0.056538968	0.08665145	0.020438017	0.049574999	0.089145243	0.127474172	0.024291145	0.130103922	0.077570565
0.026759696	0.009820025	0.021582785	0.019015013	0.005060446	0.001863271	0.099726977	0.006979943	0	0.035884883	0.050223722
0.003566531	0.005234983	8.36E-05	0.001472113	0.000749255	0.000267499	0.017400785	0.000151834	0.002898943	0.002909106	0
0.013822012	0	0.000401274	0.006248013	0.000547075	0.003011673	0.000728682	0.000268459	0.000180579	0.044181409	0.038188689
0.00036425	8.27E-05	0.000198248	0.002868667	0	0.00112073	0.001257955	0.000374083	0.000196702	0.001218885	0.001987149
0	0.000363376	0	0.005445516	0.003811687	0.029023674	0.000784591	0	0.000325688	0.006266741	0.012366972
0.075652923	0.142260378	0.165845409	0.127005591	0.178637902	0.10659847	0.116518352	0.016210983	0.005701148	0.060133342	0.069767755
0.003083462	0.047481181	0.015905242	0.128592347	0.004293351	0.178837116	0.005961777	0.000470904	0.000599781	0.003720006	0.001161787
0.01013861	0.025510204	0.008796968	0.055890798	0.031105984	0.013697809	0.005223776	0.001524937	0.001576845	0.003296817	0.0074933
0.000338928	0	0	0.004291275	0	0	0.000178909	0	9.35E-05	0.000727276	0.001139358
0.000373989	6.50E-05	0.000437102	0.044202479	0.000166501	0.000322844	0	0.001544741	0.000519166	0.002012047	0.069337131
0.004762517	0.00610945	0	0.005700856	0	0.000115301	0.004362776	0.001529338	0.005427054	0.001056705	0.021304655
0.001172612	0.003813975	0.000492038	0.001070865	0	0.000724093	0.006494777	0.000941808	0.000109637	0.001857469	0.004774988
0.001455051	0.001350106	0.000919585	0.003269915	0.000142715	0.003435982	0.016416784	0.012373335	0.014665622	0.003813767	0.00346966
0.019427953	0.017317987	0.039611433	0.039908599	0.011054488	0.02762622	0.019059422	0.022372344	0.108595899	0.023769522	0.046843776
0.001121967	0.022570696	0.003167195	0.015648694	0.001676905	0.001102282	0.012976509	0.002521757	0.010370413	0.011565469	0.015477779
0.005323501	0.001468277	0	0.00268107	0.000148662	0.015574916	0.004838003	0.006986544	0.038034523	0.010161597	0
0.002074471	0.00227775	0.000291401	0.011357419	0.000594647	0.003975593	0.004442912	0.002336916	0.010854108	0.001330384	0.012564341
0.037301513	0.011512827	0.041567642	0.007837374	0.015181338	0.002767234	0.030740703	0.079349536	0.000367608	0.027859501	0.011804021
0.00949192	0.002416601	0.010375788	0.001190718	0.002652126	0.000207543	0.005993459	0.017718316	0.000128985	0.001038966	0.001143844
0	0	5.97E-05	0.0032152	0	0	0	0	0.001370468	0.000192589	0
0.001131706	0.003503776	0.000255573	0.004458028	0.000856292	0.000115301	0.001054819	0.001148654	0.005991364	0.013308905	0.038074305
0.000239587	0.000416553	0	0.000442937	0	0	0.000316818	0	0.001257606	0.001591392	0.003146692
0	9.45E-05	0	0	0	0	0	5.50E-05	0	0.00079823	0
0	8.57E-05	0.000527866	0	0	0	0	3.96E-05	0.000196702	0.000615778	0
0	0	0	0.000937984	0.000214073	0.000142974	0.000149091	0.000250855	0.001954126	0.000164714	0.000556222
0	0	0	0	0	0	0	0	0	0	0
0.011402771	0.004050317	0.013065276	0.012329274	0.001873138	0.001402065	0.014968737	0.014727855	0.035341956	0.018306079	0.034221121
0.050548907	0.053729483	0.091877315	0.121773727	0.047440937	0.075836051	0.059002768	0.07823389	0.252340276	0.109960646	0.141235576
0.009184158	0.004466871	0.009809706	0.01289467	0.008164503	0.024379332	0.010156825	0.017881152	0.153276386	0.011208166	0.020071098
0.002074471	0.001169895	0.005586779	0.000205835	0.001867192	0	0.000458455	0.000737163	0	0	0
0.000564879	0.000995592	0.003198246	0.000367377	0.001373635	0.000110689	8.01E-05	0	0.000283768	0.000326894	6.73E-05
0.000120767	0.000404736	0.000847929	0	0.000321109	0.000193706	0	0	0	0	0
0.000338928	0.000460868	0.000183917	0	0	0.000719481	0	0	0	0	0
9.54E-05	0.000549496	4.78E-05	0	0	0.000507326	0	0	0	0	0
0.000557088	0.000499273	0.000563694	0	0.000279484	0.001180687	0	0	0	0	0
0.001094697	0.001033998	0.001127388	0	0.000368681	0.002093874	0	0	0	0	0
0.000116872	0.000673576	0.000112261	0	0.000362735	0.000327456	0	0	0	0	0
0	0.000159531	0.000133758	0	0.000570861	0.000903963	0	0	0	0	0
0.000925233	0.00092469	0.00053742	0	0.000564915	0.002338313	4.66E-05	0	0.000128985	0.000101363	0
0.007403815	0.022742044	0.015217345	0.011993163	0.027240779	0.067612753	0.002050001	0.008452068	0.023004518	0.001142863	0.006019759
0.430644508	0.345130756	0.068725264	0.080934961	0.159085909	0.189288037	0.24450553	0.270659816	0.102220803	0.182949296	0.05017438
0.008373849	0.077827541	0.022726893	0.066458747	0.254205641	0.036269215	0.004977776	0.009391676	0.000303115	0.017312726	0.001421955
0.038285182	0.060999965	0.035393284	0.015781575	0.096320919	0.078095958	0.023142652	0.021954252	0.014407651	0.017487577	0.019458805
0.009345831	0.013114047	0.004846334	0.012941569	0.05599196	0.009736052	0.020220468	0.004464787	0.001212461	0.003892323	0.005909861

H048	H049	H050	H051	H052	H053	H054	H055	H056	H057	H058
0.002741489	0.007647863	0.001861602	0.002803264	0.006199449	0.035768162	0.028910785	0.033708286	0.002422187	0.001788534	0.023900965
0.000159515	0.000846147	0	0.001420764	0.004344496	0.052124457	0.020128429	0.047694082	0.000612472	0.00047765	0.014957153
0	0.001154064	0.00034014	0.001627058	0.007539972	0.189396225	0.069419148	0.126159412	0.000550917	0.000634213	0.130693298
5.14E-05	0.000398039	0.000295331	0.000998195	0.004949045	0.122969639	0.039891772	0.085688237	0.000741737	0.000812005	0.084929198
0	0.000255346	0	0.000332732	0.00108894	0.030908776	0.009538896	0.027157713	0	0	0.022130926
0.00041636	0.002090332	0.000313662	0.001941489	0.00992062	0.28556542	0.095595841	0.182287851	0.001449619	0.000904881	0.191288275
0	0.000415563	3.67E-05	0.000785247	0.004032833	0.103940789	0.033036261	0.069949794	0.00019082	0.000313126	0.066166353
0	0.000468134	0	0.000858448	0.003030258	0.08551107	0.034557454	0.060978402	0.00046474	0.000496225	0.046719877
0	0.000150204	0	0.000231248	0.001768589	0.043347178	0.013739418	0.024326833	0.000273919	0.000132681	0.030260477
0.014399576	0.003301974	0.067148022	0.005014266	0.010757977	0.000269609	0.006307881	0.002637298	0.011910881	0.002722605	0.020369596
0.000908422	0.000122666	0.014909109	0.004654916	0.000446841	0	0.001602324	0.001344668	0.016444402	0.00184426	0.004384819
0.002062876	0.001053928	0.000892102	0.000146402	0.009312316	0	0.000740314	0	0.001726616	0.002390903	0.001815759
0.073649803	0.134342033	0.020019349	0.101296822	0.011741779	0.001814513	0.118971511	0.005913625	0.013588253	0.152601202	0.062637162
0.203943526	0.141444157	0.0615734	0.131254315	0.362587021	0.008569723	0.027852035	0.045874825	0.002711495	0.073544826	0.035762183
0.067966756	0.030914389	0.00743215	0.020418077	0.010754222	0	0.012820617	0	0.000153887	0.024731654	0.009755896
0.001795216	0.015077931	0.001297418	0.042749362	0.079319901	0	0.015873145	0.076046593	0.004345779	0	0.000248197
0.001892547	0.02129886	0.000140537	0.00841312	0.089863845	0.003430029	0.006754098	0	0	0.035168305	0.006786237
0.001795216	0.014106614	0.000122206	0.007403279	0.01325503	0	0	0	0.001424997	0.026313206	0
0.001803327	0	0.000945058	0.006864254	0.001197834	0	0	0	0	0.000493572	0
0.011847342	0.035275298	0.118010082	0.177306038	0.097445121	0.000969309	0.035995489	0	0.215377655	0.04623917	0.029404849
0.021069451	0.000698446	0.031001578	0.0055666	0.000315417	0	0.002523153	0	0.006195505	0.000193714	0.004439248
0.003230847	0.001256703	0.035093437	0.024036534	7.89E-05	0.000239653	0.006701363	0.000589073	0.006081628	0.000806698	0.00209226
0.000497469	0.000210285	0.000150721	0.002352413	0	0.001900103	0.000158204	0	0.000280075	0	0
0.000110849	5.51E-05	0.000757676	0.0075763	0.018496962	0	0	0.02113793	0.004074937	0.006074115	0
0.138899403	0.000205278	0.045370946	0.05286274	0.00551229	0.000246072	0.005178142	0.006159245	0.008540748	0.013376853	0
0.038061816	6.76E-05	0.026435155	0.007163712	0.008260925	0	0.005662895	0	0.00258223	0.005694649	0
0.002230501	0.032579145	0.008584959	0.006656297	0.020100333	0	0.019956027	0	0.012055535	0.006978997	0.00408437
0.039957066	0.110522258	0.071115637	0.029395177	0.001577086	0	0.04889318	0.010118314	0.053306577	0.01905558	0.02054377
0.072384501	0.014920217	0.015575131	0.020581116	0.000428066	0.000213976	0.020166966	0	0.022295199	0.001637278	0.005011844
0.017278951	0.156239204	0.048745863	0.031318366	0.00110396	0.00096289	0.055472848	0.000154033	0.07757769	0.004359883	0
0.017341134	0.001935122	0.009978105	0.009349759	0.000270358	0	0.001196672	0.002656031	0.002936171	7.16E-05	0
0.00292804	0.022815916	0.005487041	0.007591272	0.029833204	0.002394387	0.019951971	0	0.00028623	0.028494474	0.029520239
0.000405546	0.005487435	0.001387036	0.00230583	0.002996463	0.001208962	0.004460139	0	0	0.005662806	0.003270108
0.000846239	9.26E-05	0	0.000217939	0.000717198	0	0.001953212	0	7.69E-05	0.000342316	0
0.002116949	0.002027748	0.000985794	0.006549822	0.032807137	0	0.005914399	0	9.85E-05	0.001639932	0
0.000270364	7.01E-05	0	0.005065839	0.006368422	0	0.000281928	0	0	6.10E-05	0
0.016667928	0	0	0.000103147	0	0.00026533	0	0	0	0	0
6.76E-05	0.00156462	0	0	0	0	0	0	0	0.000145949	0
0.000532617	0.00041306	0.000378838	0.000176348	0.000116404	0	0.001176389	0.000158196	0.000603238	0.000199021	0
0	0	0	0	0	0	0	0	0	0	0
0.023756867	0.020710563	0.041024492	0.012717003	0.01598489	0.000740356	0.01470284	0.033212882	0.016918375	0.007419496	0
0.102294848	0.065063161	0.163554152	0.136980627	0.066515467	0.012693033	0.104033393	0.108073003	0.124778018	0.074611578	0.059739349
0.012926093	0.009775746	0.020656856	0.014194332	0.014013533	0.006066208	0.01045364	0.009443899	0.054768507	0.00946543	0.009248615
0	0.000285387	5.09E-05	2.83E-05	7.51E-05	0	0.000162261	0	0	0.000488264	0
0	0.000112653	8.96E-05	0	7.51E-05	0	0	0	8.31E-05	0	0
0	0	0	0	0	0	0	0	0	0	0
0.000638059	0	0	0	0	0	0	0	0	0	0
0.001365337	0	0	0	0	0	0	0	0	0	0
6.49E-05	0	0	0	0	0	0	0	0	0	0
0.000538024	0	0	0	0	0	0	0	0	0	0
0.00020818	0	0	0	0	0	0	0	0	0	0
7.57E-05	0	0	0	0	0	0	0	0	0	0
0	0	3.46E-05	0	0	0	0	0	0	0	0
0.002695527	0.011400448	0.004236468	0.002633571	0.013604242	0	0.004145759	0.004046493	0.018694235	0.079828577	0.00959914
0.069488904	0.067506471	0.135450889	0.069963483	0.009199667	0.007495565	0.075035393	0.012632802	0.275018236	0.29767411	0.050246891
0.004863845	0.037015156	0.020882937	0.012557292	0.012383878	0.000736076	0.002987624	0	0.003154691	0.021051095	0.009446738
0.01304235	0.021131133	0.00813076	0.009642563	0.001077675	0.000252491	0.011171643	0	0.016031984	0.027759424	0.006496673
0.007710776	0.005474919	0.009503539	0.005894341	0.008531283	0	0.005924541	0.00185048	0.019171286	0.015298067	0.004049535

H059	H060	H061	H062	H063	H064	H065	H066	H067	H068	H069
0.04225528	0.023684053	0.000467292	0.096357113	0.008797024	0.001509296	0.013313086	0.079554786	0.00118911	0.010537145	0.000791205
0.042911333	0.016391058	0	0.012490168	0.000754031	0.000258948	0.001260328	0.011700472	0	0.001680247	0.000123399
0.140163854	0.022725912	0	0.014920649	0.000687888	0.000120842	0.00220828	0.013153313	7.84E-05	0.002694804	0
0.091180056	0.014641595	0	0.008630687	0.000271186	0.000125775	0.001359299	0.008645781	0.000218829	0.001477336	0.000112511
0.020230958	0.004003661	0	0.002359621	0.000152129	0	0.000143817	0.00228373	0	0.000427182	0
0.194477802	0.031548082	0	0.018997433	0.001564283	7.40E-05	0.002293333	0.019063476	0.000249796	0.003186062	0.00029398
0.073993276	0.011384343	8.54E-05	0.007317425	0.000582059	3.95E-05	0.000969602	0.006251913	0.00016309	0.000662131	0
0.06311732	0.009440257	0	0.004990871	9.59E-05	0	0.001116512	0.005080894	0	0.001139151	0
0.033143176	0.00508157	0	0.003063492	7.28E-05	0.000125775	0.000230416	0.002304786	0.000138317	0.000897081	0
0.003255296	0.003449736	0.011923485	0.003472116	0.038693675	0.001726319	0.012867718	0.0184642	0.00680435	0.001413259	0.00117229
0	0.004598222	0.000226109	0.000177149	0	5.67E-05	0.000425264	0.029001751	0.002539245	0.001021676	0.001125108
0	0.000637335	0	0.004929459	0.014468789	0.000342798	0.001784563	0.001355661	0.000712227	0.000348865	0.058988346
0.061455621	0.05297366	0.367213683	0.10559483	0.105779248	0.249093066	0.078268696	0.021886555	0.199477287	0.192167626	0.108271725
0.06425009	0.089990419	0.0060346	0.117192169	0.229995866	0.176508741	0.184010998	0.093509834	0.134392109	0.058961735	0.150543137
0.012326537	0.00089398	0.016646652	0.024337877	0.016238115	0.09811412	0.022903951	0.008823944	0.077339615	0.033159969	0.028087787
0.000933003	0.005010993	0.010174908	0.020317781	0.040668045	0.002522892	0.138073198	0	0.011096293	0.034409475	0
0.009604711	0.001231896	0.005610017	0.00563333	0.019541959	0	0.008633634	0.000239711	0	0.007899299	0.010231228
0.00056298	0.00127467	0.005924058	0.001310901	0.001551054	0.000273745	0.002409314	0.000877859	0.001003311	0.007568233	0.000391973
0	0.000881148	0.000120592	0.014467148	0.011217859	0.000157835	0.008315073	0	0.000408756	0.001135591	0.008536307
0.011087073	0.296172568	0.06768198	0.040217491	0.008426623	0.071186006	0.112056314	0.041429453	0.088906597	0.178558333	0.146630663
0	0.029223307	0.001180792	0.001492774	0.000277801	0.000162767	0.001541776	0.010584521	0.005712268	0.000996757	0.020738289
0.000771827	0.048993524	0.012830433	0.005801031	0.000585366	0.000586949	0.004167588	0.009002107	0.010262265	0.027364539	0.020545932
0	0.002356856	0.001889267	0.000684975	6.95E-05	0.000389655	0.000725269	0.001185596	0	0.012299269	0.000682324
0	0.005554225	0.000261282	0.019831213	0.008300951	0.019130083	0.008155792	0.00471161	0.009093799	0.023509225	0.031376008
0.001638998	0.000667277	0.000115567	0.000741663	0.000347251	0.002352726	0.034042777	0.00199057	0.000134188	0.014915756	0.003712858
0.003361989	0.000536816	0	0.00022675	0.000148822	5.67E-05	0.056577134	0.001431785	0.000132123	0.022818615	0.001549746
0	0.011234633	0.004245826	0.00547744	0.009187267	0.010180351	0.003315513	0.005615384	0.004244461	0.010850412	0.003182968
0.005405062	0.017479661	0.049553058	0.043566784	0.010119884	0.028395485	0.009128487	0.049543983	0.031616694	0.028994949	0.020313651
0.000195227	0.00404002	0.109534768	0.013106646	0.009031831	0.024829403	0.002735608	0.013331476	0.019692566	0.035342155	0.01239434
0	0.030562138	0.0111723	0.011887862	0.054511782	0.006907743	0.002434057	0.059998931	0.00016309	8.19E-05	0.001331983
0	0	0.007883669	0.006667879	0.000833402	0.000929746	0.001298988	0	0.003594167	0.004371491	0
0.002435796	0.004681632	0.014357926	0.013366464	0.055530384	0.049298744	0.008963021	0.001491713	0.069600079	0.020461997	0.004409699
0.000170256	0.001700273	0.004416664	0.003552423	0.003601488	0.006927472	0.002112403	0.000654345	0.001507031	0.00463492	0.001600557
0	0	0.000231134	0.001025101	0.003399752	0.000406918	0.000255158	0	0	0.009056249	0.013577518
0	0.0005967	0.004549817	0.001865967	0.02898057	0.000293474	0.002976849	0.000236471	0.000495462	0.009451392	0.042768638
0	0	0.001582763	3.78E-05	0.002050434	0	0.000290726	0	0.000158961	0.001292224	0.003988691
0	0	0	0	0.014941711	0	0	0	0	0	0
0	0	0.000610494	5.67E-05	5.62E-05	0.001783041	0	0	0	0.000113915	0.002493385
0	0.001088602	0	0.004100403	0.000463001	0.0001677	0.000569081	0.006483526	0.000218829	0.000822325	0.000127028
0	0	0	0	0	0	0	0	0	0	0
0.012292486	0.002506566	0.01628739	0.01677953	0.018863993	0.00410864	0.015976012	0.009419981	0.005357187	0.020963935	0.00387618
0.060638392	0.077427648	0.108597672	0.135301495	0.188623398	0.096612222	0.135606666	0.384680545	0.047721286	0.119749672	0.061467214
0.006862454	0.013608599	0.016081379	0.030835693	0.055282348	0.01172664	0.034376803	0.041337132	0.003612747	0.010683099	0.009777555
0	0.000209593	0.00068084	0.000134633	0	0.000742317	0	0	0	0	0.000217763
0	0.000220287	0.000761234	0.000108651	0.000132286	0.000106045	0.000210312	5.99E-05	0.000127994	0	0.000384714
0	0	0.000344188	0	0	0	0	0	0	0	0.000272204
0	0	0	0	0	0	0	0	9.08E-05	0	0.000134287
0	0	0	0	0	0.000128241	0	0	7.84E-05	0	0.00017784
0	0	0.000223597	0	0	0.000170166	0	0	0.000142445	0	0.00011977
0	0	0.001728478	0	0	0.000984002	0	0	7.02E-05	0	0.000112511
0	0	0	0	0	0.0001677	0	0	7.23E-05	0	0
0	0	0	0	0	0	0	0	0	0	0
0	0	0.00017335	0	0	5.18E-05	0	0	0.000245667	0	0
0.00281036	0.02079252	0.004474447	0.007246565	0.010116577	0.007487293	0.003477887	0.004486477	0.005916647	0.003851754	0.035183229
0.016664661	0.105759113	0.095392902	0.107947366	0.007685821	0.079479737	0.066364394	0.023047856	0.200265898	0.045142412	0.119239717
0.000186147	0.007059875	0.00088685	0.041622872	0.009901612	0.002540155	0.004789247	0.002928357	0.022242132	0.014096992	0.027568786
0.012798714	0.012265491	0.031358815	0.013442048	0.006435717	0.037433999	0.003884594	0.002904062	0.028051429	0.011754613	0.036224862
0.008819263	0.005419486	0.006484306	0.006313582	0.000962381	0.003225751	0.003380463	0.001245524	0.004661475	0.007034256	0.005150093

H070	H071	H072	H073	H074	H075	H076	H077	H078	H079	H080
0.002241009	0.001287155	0.000406337	0.002211899	0.000179503	0.083289409	0.001112028	0.000470166	0.000296125	0.00042225	0.002358107
0.000198736	8.61E-05	0	9.12E-05	0	0.00478522	8.68E-05	0	0	0	0.000336081
0.000183046	0.002075157	0.000204131	0.000106817	0	0.000977954	8.35E-05	0.000101964	9.28E-05	0.000214098	0
7.84E-05	0.001152865	6.16E-05	8.60E-05	0.000122587	0.00067738	0	0.00012179	0	0.000318174	0
0.000104598	0	0	0	0	0.000130684	0	0	0	0	6.28E-05
0	0.000435808	7.32E-05	6.25E-05	0	0.001707607	7.01E-05	0	7.73E-05	0	0
0	0	0	0	0	0.00036156	8.68E-05	0	0	0	6.65E-05
0	0.001043913	0	0	0	0.000531449	0	0.000130287	0	0	7.76E-05
0	0.00028885	0	0	0	0.000182958	0	0	0	0	0
0.006762252	0.001127527	0.000246498	0.009376474	8.10E-05	0.015573203	0.000537647	0.003877451	0.000377891	0.003104426	0.00711864
0.001864457	0.007613975	0.000462184	0.010384725	0.000221095	0.006597375	0.005727109	0.001515293	0.000682856	0.012741827	0.018292413
0.001082588	0.000354728	0.000215686	0.001959185	0	0.000973597	0.000945056	0.000855362	0	0.000205178	0.000858669
0.043606848	0.069242834	0.351835447	0.033053437	0.303937235	0.066951703	0.185247768	0.195594604	0.172455476	0.127870256	0.041181011
0.372833844	0.027645951	0.038074311	0.080175493	0.133429506	0.1748838	0.048755564	0.076481234	0.062586324	0.023815471	0.100637815
0.01101154	0.005285442	0.012939219	0.011510214	0.021752645	0.042846999	0.027122787	0.00581476	0.016899037	0.00788001	0.001837366
0.000209196	0.007733062	0.051720286	0.003300915	0.004822489	0.014303388	0.015468204	0.013663129	0.01177209	0.000719608	0.007428869
0.000290259	0.061106902	0.013020101	7.56E-05	0.016304086	0.021090255	0.0121388	0.005140667	0.066177397	0.047842067	0
0.000240575	0.006253341	0.012970031	6.51E-05	0.002197812	0.000572832	0.066831857	0.008610264	0.030660028	0	0.001248301
0	0.00674996	0.012529031	0.004642644	0.007939705	0.003223544	0.007129666	0.000977152	0.011995289	0.010220224	0
0.020587474	0.232668388	0.07663199	0.031146358	0.115354474	0.044258389	0.114425302	0.153296683	0.114549702	0.15925351	0.123592891
0.019962502	0.029781919	0.008966365	0.137416533	0.000553831	0.000500956	0.091647103	0.001588934	0.053147879	0.006711389	0.002463363
0.000428851	0.0335547	0.009191679	0.033686525	0.002661892	0.00384865	0.024868677	0.020191635	0.029669997	0.036170732	0.000928839
0.000279799	0.003795586	0.01105582	0.000153713	0.003587862	0.000322354	0.003215864	0.003526243	0	0.000107049	0.000546593
9.68E-05	0.042263264	0.004512069	0.003037779	0.00044	0.155947657	0.00329601	0.005355924	0.001652998	0.000240861	0.097426579
0.002766613	0.002381743	0.015483155	0.002321322	0.003611942	0.012062153	0.000140256	0.003741499	0.006607574	0.001106175	0.039602169
0.00466768	0.002967043	0.009977392	0.001216675	0.001317811	0.011138652	0.00051761	0.001931645	0.000954673	0.00221235	0.001100573
0.002975809	0.003207751	0.016906296	0.002800697	0.019049162	0.001589991	0.006077748	0.012853085	0.006212004	0.000642295	0.001682252
0.016761807	0.026201703	0.031942674	0.017491982	0.022299909	0.013338503	0.024888714	0.030801519	0.013084765	0.035828769	0.009423196
0.000622357	0.070585731	0.039245177	0.033269677	0.01770946	0.007052592	0.013314276	0.0564737	0.031581553	0.005495192	0.008462965
0	0.006879182	0.002091381	0.007677818	0.002342289	0.007756108	0	0.006163136	0.004521437	0.003125242	0.00830231
0	0.002156237	0.001436621	0.007190627	0.002510847	0.005355875	0.001035221	0.010335149	0.003876149	0.002126116	0.002452284
0.006874694	0.001760969	0.034286331	0.001839342	0.057725396	0.00488759	0.017324922	0.025791139	0.015352113	0.027232124	0.004542634
0.000826323	0.000466213	0.016045479	0.00104733	0.000704876	0.000169889	0.003977252	0.00348659	0.001794432	0.007680779	0.00144958
0.00047592	0.001403708	0.001034136	0	0.031896729	0.000272259	0.000150274	0.000118958	0.000501645	0	0.000160654
0.004521243	0.001576004	0.012413485	0.000385584	0.026717424	0.003460954	0.000684582	0.00157194	0.001111575	9.52E-05	0.001233528
0	7.85E-05	0.001802517	0	0.002532737	0.000182958	0	0.00024358	0.00019447	0	6.65E-05
0	9.12E-05	0.004962698	0	0.003911842	0	0	0.010054749	0	0	0
0	0	0.000233018	0.000174555	0.000343682	0.000298396	0	0.000252077	0.00040441	0	0
0.000101983	7.60E-05	0	0.000263135	0	0.00637739	0	0	0	0	0.000204973
0	0	0	0	0	0	0	0	0	0	0
0.063106504	0.003856396	0.011704803	0.003991319	0.006934928	0.060201864	0.00381362	0.00108478	0.009624075	0.003336366	0.016279619
0.26032577	0.099072894	0.055941565	0.196799129	0.023252149	0.150907602	0.025513186	0.092121325	0.059936665	0.068948599	0.15123279
0.02200216	0.010408722	0.004889519	0.02607905	0.002237215	0.016246226	0.002497888	0.01424942	0.003697148	0.015096909	0.027104385
8.89E-05	0	0.000223389	0.000138081	0	0	0.001933525	0.000254909	0.000548053	0.001763338	5.91E-05
0.000196121	0.000146959	0.000140581	0.000145897	0	0	0.001235586	0.000257741	0.000227619	0.000184362	0.000168041
0.002180865	0.000676516	0	0.000276162	0	0	7.01E-05	0.000135952	0.000620979	0.000220046	0
0.000397472	0	7.13E-05	0.000346505	0	0	0	0	0.00019889	0	0
0.000400087	0	0	0.000182371	0	0	7.35E-05	0.000141616	9.50E-05	9.52E-05	0
0.00025888	0	7.13E-05	0.000528876	0	0	0.000187008	0.000283232	0.000841968	0.0003152	7.76E-05
0.000721725	0.000243242	0	0.00200087	5.25E-05	0	0.000190347	0.000118958	0.001356873	0.000564982	0.000129262
0.000455001	8.87E-05	6.55E-05	0.000786801	0	0	0.00011688	0	0.00081766	0.000127864	0
0	0	0	0	0	0	0	0	0	0.00011597	0
0	0	0	0.000664351	8.54E-05	0	0.000153613	0.000167107	0.000696116	0.001204303	0.000131109
0.01981868	0.062910946	0.006848022	0.01877379	0.000967563	0.002402412	0.074348896	0.001713556	0.015657078	0.059742368	0.011024198
0.07921719	0.058894922	0.085532879	0.204523842	0.117136365	0.035256424	0.121845498	0.192898232	0.144529083	0.215290787	0.238082048
0.006738717	0.030060633	0.010085234	0.069199365	0.020566177	0.007664629	0.015919026	0.010253011	0.064020543	0.052867431	0.01982509
0.01538373	0.042278466	0.02515435	0.029127251	0.019193639	0.006656183	0.050989638	0.019993372	0.031610281	0.044868478	0.034343054
0.006050986	0.029982086	0.006295328	0.00821451	0.00331423	0.002182426	0.024204132	0.005194482	0.006231893	0.011876513	0.016397802

H081	H082	H083	H084	H085	H086	H087	H088	H089	H090	H091
0.015218531	0.002138607	0.002027777	0.01072946	0.005225129	0.001919413	0.004415497	0.00407891	0.000577793	0.000321213	0.025365479
0.001009221	0.00017502	0.000168388	0.000933956	0.000422499	0.000133098	0.000180685	0	0	0	0.001723565
0.002682551	0.000611373	0.000735217	0.001573752	0.000531425	6.77E-05	0.000750973	0	0.000100851	0	0.005079845
0.002221779	0.000290102	0.002679986	0.000922925	8.58E-05	0.000196144	0.000578758	0.000172994	0.00013867	0.00041131	0.003260238
0.000662243	4.32E-05	0.000101982	0.000185688	0	0	0	0	0	0	0.000578848
0.005322195	0.000445943	0.000175504	0.001628907	0.000442304	0.000165789	0.000575934	0.000195921	0	0.00059542	0.007257663
0.001925168	0.000112684	0.00015653	0.000786876	8.25E-05	7.47E-05	0.000152453	0	0	0	0.003439343
0.001363661	0.000153443	0.000237167	0.00045227	0.000155137	5.84E-05	0.000443244	5.21E-05	0	0	0.002546412
0.000632395	0.000134262	0.000377095	0.00035483	0.000115527	0	4.80E-05	0	0	0	0.001380929
0.013815695	0.004871803	0.008457372	0.009243955	0.050330243	0.001286614	0.002961545	0.006083975	0.001371996	0.001707915	0.005256354
7.28E-05	0.000321271	0.001117056	0.000172819	0.227387204	0.000144773	0.000982476	0.002790833	0.000310958	0.001096826	0.023325235
0.002190066	0.03183455	0.001717088	0.000196719	0.007598388	0.002617593	0.000635222	0.001782048	0.005208541	0.002127059	0.012036403
0.148898345	0.23414626	0.168414587	0.089939201	0.02766711	0.149653478	0.142188043	0.048867722	0.369211827	0.028823027	0.054832212
0.121563565	0.047118875	0.177384239	0.117698645	0.005971105	0.049201879	0.118879638	0.051650218	0.027610101	0.143840929	0.052301375
0.152994924	0.049048895	0.05320365	0.007828314	0.002003571	0.021949488	0.051740931	0.007972321	0.011230195	0.012221778	0.052553161
0	0.026224304	0.033288746	0.014237309	0.000617245	0.015089106	0.006784169	0.002092604	0.00321463	0.000219365	0.007561363
0.001205096	0.01093998	0.011640151	0.006177345	0.00046541	0.012417806	0.049386376	0.000291797	0.009076603	0	0.00387283
0.0001567	0.003390123	0.002748764	0.001663838	0.001897947	0.011047131	0.006772876	5.63E-05	0.014732671	0	0.000363402
0.002796344	0	0	0.000564418	0.000412597	0.006099156	0.037463969	0.000443949	0.000388697	0	0.004417933
0.064278612	0.210880521	0.040249594	0.024863813	0.013968887	0.11533055	0.272219352	0.114763905	0.083981687	0.173940975	0.118370504
0.000524198	0.002891435	0.005094345	0.007092916	0.021877548	0.001814335	0.000104459	0.009062393	0.002231332	0.102866634	0.009793691
0.000830136	0.040379387	0.004620011	0.004982323	0.013876465	0.043506688	0.012769369	0.00460623	0.019859271	0.027005429	0.031283744
6.90E-05	0.000122275	0.000434015	0.003719276	0.000290468	0.000261526	0.000129868	0.000404348	0.006393542	0.000180193	0.000106425
0.006588852	0.002081066	0.000258512	0.252469559	0.000980331	0.001088134	0.024194892	0.045943496	0.000359282	0.049991774	0
0	0.000748033	0.006439081	0.021887289	0.000244257	0.001718598	0.020293218	0.030125994	0.000247926	0.000164524	0.003182366
0.000550315	0.00059459	0.003972546	0.003164051	0	0.000481021	0.010925815	0.00268662	0.000165984	0.001727501	0.000742379
0.000759248	0.010594734	0.005350485	0.005785745	0.002960797	0.021108869	0.005697234	0.004228978	0.020199643	0.001057654	0.011937765
0.027502672	0.026945964	0.023524585	0.03090511	0.02524434	0.037449563	0.01552482	0.078762362	0.052121131	0.005464545	0.056021057
0.000820808	0.01924746	0.008111108	0.003754208	0.014741269	0.04520894	0.004486077	0.03467178	0.022250283	0.000591503	0.024994289
0	0.00335416	0.007971179	0.024628486	0.009872623	0.036606609	0.000810261	0.130637681	0.002611624	0.001280936	0
0.00029661	0.000522664	0.00538606	0.00283496	0.00187154	0.006360681	0.001665693	0.049749367	0.001737581	0.000830454	0.004360827
0.003574247	0.027890595	0.03379154	0.007069015	0.006482725	0.022129287	0.026749895	0.004206051	0.124164564	0.003200382	0.006388093
0.001565132	0.005617439	0.004017607	0.000801584	0.000627148	0.005499047	0.00173345	0.001525683	0.007250777	0	0.002234924
0	0.000323668	5.69E-05	0.00011031	0	0.000966711	0.001019178	0	0.00072907	0.001911611	0.000251786
0.000596951	0.015512091	0.007527677	0.01138948	0	0.0060688	0.004152939	0.001183864	0.030749093	0.001088992	0.00225569
7.46E-05	0.000791189	0.000619006	0.000774007	0	0.000819603	0.000496885	6.04E-05	0.003160002	0	0.000552891
0.000764844	0	0.000120955	0.002509546	0	0.000154113	0.037226819	0	0	0	0
0	0	5.22E-05	0.000123179	0	0.00156682	0.00141725	0	0.000850932	0.002275914	9.34E-05
0.000693956	9.83E-05	0.000130442	0.000580965	0.000458808	0.000453	8.75E-05	0.000477297	0	0	0.001521098
0	0	0	0	0	0	0	0	0	0	0
0.01231772	0.003414099	0.015157337	0.023659599	0.002607614	0.008242733	0.013554221	0.015217233	0.001855241	0.000842206	0.012363465
0.087259377	0.056428527	0.11608371	0.194913618	0.145382709	0.083900751	0.0653375	0.197980345	0.02222507	0.075594832	0.099769499
0.017526121	0.008770205	0.01021715	0.055347908	0.031733667	0.009552697	0.006693826	0.016815865	0.002300667	0.010874249	0.031984592
0	0.000124672	0.000258512	0	0	0.000112082	0.000101635	0.000379337	0.00171657	0	0.000205063
6.53E-05	0.000141455	6.40E-05	4.41E-05	0	0.000133098	6.21E-05	0	0.00264314	0.000548413	0.000301105
0	0	0	0	0	0	0	0.000104213	0.000178591	0	0
0	0	0.000123327	0	0	6.77E-05	0	0	7.98E-05	0.001085075	0
0	0	5.22E-05	0	0	9.81E-05	0	0.000147983	0.000384495	0.000403475	0
0	0	0.000163645	0	0	0.000177464	0	0.00017091	0.000390798	0.000556248	0
0	0	0.00032729	0	0	0	0	0.001117167	0.000399202	0.002009542	0
0	0	0.000232424	0	0	0	0	0.000502308	7.98E-05	0.000301627	0
0	0	0	0	0	0	0	0	0	0	0
0	0	0.000220565	0	0	0.000123758	0	8.34E-05	7.77E-05	0.000176276	0
0.007006718	0.009710041	0.003303735	0.001266723	0.00531425	0.004751831	0.012966994	0.005640026	0.002449842	0.069499612	0.01279176
0.275944536	0.063124859	0.180419975	0.043182583	0.316610498	0.197841945	0.012052275	0.088598018	0.055014298	0.124074553	0.265091577
0.002473618	0.047627154	0.01708076	0.001834819	0.014942616	0.034421001	0.006778522	0.018495785	0.039945456	0.112651891	0.013783329
0.010843996	0.020868198	0.025758697	0.003377316	0.029789509	0.027394829	0.012103092	0.008824786	0.041290138	0.031028431	0.010920238
0.002341169	0.00919457	0.008177515	0.001636261	0.00871075	0.012466842	0.003704049	0.006294486	0.006731814	0.005409704	0.011545809

H092	H093	H094	H095	H096	H097	H098	H099	H100	H101	H102
0.001859828	0.000498564	0.000963931	0.000624347	0.01368115	0.000705606	0.012010641	0.076516597	0.007084854	0.008287724	0.002175538
6.24E-05	6.76E-05	0	5.71E-05	0.001535834	0	0.001153942	0.004868339	0.000780177	0.001192033	0.000229688
0.000195863	0.000227987	0	0.002387332	0.00189496	0	0.001081589	0.00411201	0.001082408	0.000686159	0
0	8.52E-05	0.000245364	0.002101614	0.000827135	9.16E-05	0.000706836	0.002654695	0.001068351	0.000772265	0
0	0	0	0.000188362	0.000345754	0	0.000213349	0.000389065	0.000234288	8.34E-05	0
0.00014733	0.000666423	9.06E-05	0.002577811	0.001747871	0.000131347	0.001875619	0.00592485	0.001609555	0.001364245	0.000223188
8.49E-05	0.000170364	0	0.000747099	0.000529137	0.000143565	0.0006586	0.001708866	0.000719263	0.000618888	6.07E-05
0	0	0	0.001013769	0.000645662	0	0.000528735	0.001556258	0.000477947	0.000118396	0
7.28E-05	0	8.47E-05	0.000560854	0.000200575	0	0.000274571	0.000895519	0.000400632	0.00023141	0
0.000230529	0.002956312	0.003107949	0.000994722	0.008007733	0.00078808	0.018362887	0.00440884	0.002274932	0.008115511	0.000764905
0.000485323	0.001573359	0.001790576	0.001970395	0.00063611	0	0	0.002349481	0.000161658	0.000277154	8.88E-05
0.000287727	0.000348243	0.001077851	0.000338629	0.000135627	0	0.000116878	0.000362233	6.09E-05	0.001348101	0.000777907
0.157321015	0.164020183	0.070326101	0.21285138	0.100532193	0.16728369	0.051877289	0.035009106	0.047037903	0.057196058	0.274358335
0.063378024	0.128386605	0.055020038	0.096661545	0.299132368	0.127198529	0.145929109	0.046895701	0.05097159	0.228620094	0.109426971
0.031856275	0.031840479	0.020172456	0.048392149	0.020968722	0.044444037	0.013121913	0.00650677	0.010692882	0.049435735	0.020624275
0.019151204	0.013902181	0.002050545	0.049433432	0.02008619	0.0070469	0.023674358	0.004623496	0.01375502	0.003654133	0.103730268
0.007390781	0.017464787	0.028114667	0.057806025	0.039137029	0.021055172	0.071566652	0.010219654	0.003921973	0.000715758	0.015783486
0.011082704	0.00290871	0.001063246	0.014525052	0.002794683	0.016677969	0.023314447	0.009055814	0.002549048	9.15E-05	0.00290794
0.022073544	0.011842784	0.000400178	0.002404263	0.010697353	0.002605551	0	4.36E-05	0.002593563	0.001173197	0.002554741
0.191567508	0.075107855	0.13003143	0.026656423	0.025274406	0.106289977	0.074022953	0.081911515	0.134305314	0.028032957	0.081160143
0.012651339	0.013646636	0.059392782	0.020523012	0.007411737	0.037372701	0.137879344	0.280020862	0.082040457	0.000815318	0.000502714
0.02371671	0.001688605	0.00119177	0.010222352	0.038158986	0.024690114	0.000293123	0.020258191	0.016065094	0.001014439	0.028121648
0.003849654	0	0.000324231	0	4.58E-05	0.00329283	0	0.00021801	4.69E-05	0	0.016316536
0.000362259	0.003750507	0.000473203	0.002387332	0.006303797	0.004474949	0.018986238	0.00504107	0.000288174	0.025511659	0.00449409
0.009657934	0.002728325	0.003773938	0.002063518	0.003358205	0.002434495	0.078961527	0.014511103	0.023360807	0.01497441	0.00399571
0.006676662	0.000686466	0.000841249	0.001092077	0.003255052	0.001117974	0	0.019736644	0.026116029	0.008508371	0.005111648
0.005891478	0.009615529	0.00188989	0.002924905	0.005362048	0.006998027	0.000450816	0.006508447	0.000524804	0.012208248	0.037543202
0.042204061	0.015057147	0.023169407	0.02175689	0.050982437	0.015624141	0.004654727	0.027261354	0.00613599	0.026426538	0.017202787
0.025351209	0.001778798	0.006072768	0.018087002	0.014252312	0.014683332	0.005419074	0.01008214	0.000281145	0.007954062	0.015499626
0.025186546	0.00197171	0.008053209	0.002226483	0.002391622	0.000956081	3.15E-05	0	0	0.01559599	0.000253524
0.005381889	0.00147064	0.00160071	0.001329117	0.003788009	0.005599032	0	0.000830116	0	0.001875501	0.003629508
0.024066836	0.016059287	0.005272413	0.014548333	0.010164395	0.034547221	0.000656745	0.008748923	0.001492411	0.004515195	0.023272191
0.005264025	0.00244021	0.001355346	0.003348191	0.001944626	0.000720879	3.52E-05	0.002565814	0.000728634	0.001033275	0.01005428
0	0.000616316	0.000312547	0.000110054	0	0.014249583	0	3.86E-05	0	0.000150686	0.002123533
0.003918986	0.000756615	0.000221996	0.000308999	0.00419107	0.020590877	0.000402581	0.003265124	0	0.000443985	0.049469658
0.00114051	0	0	0	0.000993326	0.001026337	0.000231901	0.000223041	0	0.000247555	0.002498402
0.000155997	5.76E-05	0	0	0.00047565	0.000369603	4.45E-05	0.000590305	0	0.008150492	0.000710734
0.000214929	0.00025304	9.35E-05	0	0	0.000296294	5.57E-05	0	0	0	0.000728069
0	0	0.000131445	0.000139684	0.000993326	0	0.000294979	0.00337916	0.000316288	0.000473584	0.000169016
0	0	0	0	0	0	0	0	0	0	0
0.024644024	0.00596273	0.00354902	0.00842762	0.02040329	0.001799143	0.00091833	0.010858592	0.009997048	0.065101686	0.011100879
0.10531169	0.14245915	0.048620118	0.036385647	0.209012902	0.03619669	0.142227961	0.09162136	0.149339544	0.312457956	0.080057205
0.010112058	0.014250425	0.005348359	0.004797945	0.025188445	0.003051518	0.017520621	0.020036827	0.016636756	0.029784681	0.00608674
0.000565055	0.000686466	0.000481966	0.00032593	7.83E-05	0.00025353	3.71E-05	0	0	0.00013185	0.000312029
0.000526922	0.00017788	0.00078575	6.98E-05	0	0.000277966	0	0.000591982	0	0	0.000171183
6.76E-05	0.000481027	0.000201549	6.14E-05	0	0.000513168	0	0	0	0	0
0	8.27E-05	0.000703962	0	0	0.001154629	0	0	0	0	8.88E-05
0	7.01E-05	0.000733172	0.000122753	0	0.000745316	0	0	0	0	0
6.93E-05	0.00029062	0.000286258	0.000120636	0	0.000152729	0	0	0	0	0
0	0.001956678	0.0009318	0	0	0.000653679	0	0	0	0	0
5.89E-05	0.000438436	0.00049365	0	0	0.000418477	0	0	0	0	0
0	8.52E-05	0.0003476	7.41E-05	0	0.00024742	0	0	0	0	0
6.41E-05	0.00035576	0.000315468	0.000351327	0	0.000375712	0	0	0	0	0
0.007832771	0.001528263	0.029572248	0.020218246	0.003069758	0.014939917	0.034907666	0.027979111	0.030312118	0.002287197	0.004654438
0.101202042	0.261302882	0.320837277	0.214140285	0.020202715	0.158281864	0.079525511	0.115537094	0.211917737	0.052793878	0.030637385
0.018301888	0.014728946	0.074839053	0.035704157	0.010538803	0.058687511	0.018051211	0.020768	0.108336887	0.005650721	0.01402182
0.011183236	0.023309766	0.045062334	0.03594543	0.005940851	0.027610285	0.011969827	0.003042083	0.030103602	0.005572687	0.014325182
0.017121513	0.007187846	0.038180448	0.019894433	0.002681979	0.011133919	0.005953375	0.006273667	0.004177346	0.004305311	0.00198052

H103	H104	H105	H106	H107	H108	H109	H110	H111	H112	H113
0.001041223	0.062322585	0.06275508	0.004727689	0.07169231	0.006713052	0.001720873	0.00818018	0.001463623	0.002980313	0.009674819
0	0.005457856	0.008887895	0.000235708	0.011680923	0.000426351	0.000115891	0.001319719	0.000479182	0.000129926	0.003285748
0.000198948	0.006838272	0.021229069	0.000595067	0.022768741	0.000840859	0.000122451	0.001968148	0.000480812	0.000667622	0.004253017
0.000132012	0.005404943	0.01192453	0.000732241	0.010436482	0.0006415	0.000150877	0.001211648	0.000383019	0.000403771	0.001955752
0	0.001355515	0.003523226	0.000212524	0.005171871	0.000185542	8.53E-05	0.000266022	0.000145058	5.60E-05	0.000649089
6.69E-05	0.010083105	0.025730039	0.001493463	0.027638668	0.000663212	6.12E-05	0.003075881	0.000899688	0.000993438	0.006613919
0	0.002844871	0.009724224	0.000297533	0.006690218	0.000416482	5.47E-05	0.000928999	0.000234701	0.0003538	0.002182721
0	0.003036292	0.008135199	0.000511989	0.009176944	0.000106588	0	0.000791831	0.000291747	0.000195889	0.001945146
0	0.001683889	0.003650956	0.00034197	0.004315644	0.000132248	0	0.000278492	8.96E-05	0	0.000876058
0.007755253	0.010092443	0.007317118	0.001800657	0.021237453	0.001521836	0.004615963	0.03316132	0.001722772	0.001761003	0.009146639
0.004562789	0.000678536	0.000615842	0.004882252	0.011387606	0.000523069	0.000561962	0.002028419	0.00162661	0.00051171	0.001060603
0.003032562	0.000544696	0.000162704	0.000809523	0.002840431	0.001048113	0.002466512	0.012956108	0.000264039	0.003805846	0.006187557
0.140931411	0.078269734	0.080661643	0.135883877	0.073693768	0.06356378	0.141091913	0.1857541	0.07426827	0.080556405	0.063612838
0.025392829	0.105187064	0.174541844	0.041067333	0.174825627	0.164543795	0.089861499	0.119649682	0.100615113	0.043641299	0.048323187
0.008259131	0.010876805	0.047933811	0.022801876	0.073984929	0.055883543	0.047388515	0.049182917	0.123606054	0.020250538	0.041734722
0.004027302	0.005274216	0.052932017	0.015634026	0.017258261	0.042021219	0.055159777	0.041270838	0.00762942	0.010933811	0.031671722
6.14E-05	0.009449701	0.000599116	0.013514584	0.003170413	0.014715026	0.02884813	0.009842818	0.016592073	0.028277995	0.034768683
0.003852526	0.003440924	0.00043337	0.000840435	0	0.008426351	0.00527851	0.004445479	0.001641279	0.012396983	0.007044524
0.000687951	0.004556773	0	0.001704055	0.002456531	0.00452603	0.007185246	0.003098742	0.006741141	0.005562851	0.018698428
0.042152803	0.148625809	0.097611749	0.082211948	0.089129581	0.151609178	0.164018665	0.041455806	0.346430422	0.236244286	0.192242751
0.002722055	0.002729706	0.00076334	0.0072915	0.002743378	0.005899827	0.001981081	0.006698354	0.001685285	0.113269889	0.022915385
0.00063589	0.006245331	0.019962411	0.039114046	0.001501093	0.034587713	0.025896188	0.013486074	0.002166097	0.024835943	0.002869991
0.000141309	0.000314368	0.000997512	5.41E-05	0.000483111	0.000341475	0.000402339	0.000910294	0	0.002386649	0
0.000656342	0.116214828	0.024340213	0.00011399	0.007509781	0.00949815	0.011315779	0.0098158	0.034719483	0.005087121	0.001824237
0.000115278	0.0003844	0.006971942	0.011200006	0.000321355	0.006728843	0.001257309	0.01382068	0.01337471	0.01438186	0.003364232
0.000351413	0.001024029	0.005217172	0.00141425	9.92E-05	0.000325685	0.000443885	0.01267346	0.005719213	0.002740449	0.004149078
0.004388012	0.001909549	0.004817255	0.020023609	0.001449332	0.00583469	0.004502259	0.008219668	0.001333233	0.013836169	0.003858473
0.062614697	0.012428411	0.016428541	0.038828105	0.018004495	0.010044905	0.016073873	0.021300474	0.01200236	0.011393551	0.014943894
0.048567481	0.00231885	0.010540785	0.025755957	0.000590948	0.009926474	0.013025719	0.003183952	0.00625055	0.011063738	0.003678171
0.036533917	0.00491316	0.002793339	0.13902923	0.002739064	0	0	0.006220345	0.005601862	0.007046012	0.00059818
0.018820108	0.002376432	0.014021434	0.006708025	0.000450759	0.001267209	0.00444322	0.003539341	0.00188087	0.001427192	0.003828776
0.021517992	0.007951008	0.00851839	0.014043962	0.010831166	0.009792253	0.016054193	0.022890372	0.0119991	0.012285047	0.014752986
0.004793345	0.000266123	0.000894112	0.003263207	0.000222145	0.000254626	0.002543044	0.001893329	0.001049636	0.002710466	0.002732113
0.000211963	0	0.000430329	0.000112058	0	0.000260548	4.81E-05	0.000887433	0	0	0.006372102
0.00052433	0.005476531	0.002828312	0.000693601	0.004102126	0.004529978	0.005921378	0.013544267	0.002676246	0.002394645	0.025789619
0.001223437	0.000395294	0.000364944	0.000117854	0.000224301	0.002688379	0.000413272	0.000939391	0.000211883	6.00E-05	0.003841504
0	0.000227216	0	0	0	4.74E-05	0.001331654	0.000315901	0.000309675	0	0.035519589
0	0.000186753	0	4.06E-05	0.000131561	0.000331606	0.000104958	0.000249396	0	5.80E-05	0.000434847
0.000325382	0.001231013	0.001839924	9.27E-05	0.002855528	0.00046188	8.75E-05	0.000369937	0.000193954	0.000163907	0.000184545
0	0	0	0	0	0	0	0	0	0	0
0.007602788	0.112697647	0.037031124	0.013023847	0.04529377	0.021045152	0.028461098	0.055318052	0.021431156	0.002730455	0.044891076
0.037993489	0.118718875	0.084134688	0.078003976	0.114259957	0.207899334	0.143092673	0.179043276	0.111375512	0.058061137	0.155473771
0.007407559	0.025099602	0.009552397	0.009308544	0.026072872	0.014539354	0.014298334	0.037091381	0.009761289	0.004601396	0.019358123
0.0007177	0	0.000106442	0	0	0	0.000131197	0	3.91E-05	0	0.000108181
0.000213823	0	8.82E-05	3.48E-05	0.0003839	0	0	0.000689995	0	0.000111937	0.00035212
0	0	0	0	0	0	0	0	0	0	0
6.88E-05	0	0	0	0	0	4.81E-05	0	0	0	0
0	0	0	0	0	0	0.000104958	0	0	0	0
0.000126434	0	0	0	0	0	0.000172743	0	0	0	0
0.000280758	0	0	5.99E-05	0	4.74E-05	0.000758759	0	0	0	0
0	0	0	0	0	0	0.000190236	0	0	0	0
0	0	0	0	0	0	4.81E-05	0	0	0	0
0.000375584	0	0	0	0	0	0.000185863	0	0	0	0
0.005180085	0.002583416	0.004409734	0.011441511	0.014926822	0.012593141	0.000800304	0.007409132	0.001429396	0.024246277	0.008959973
0.385038739	0.067084786	0.116407861	0.202367129	0.08095768	0.080592154	0.125293554	0.036673643	0.035480632	0.170065743	0.102102115
0.055086282	0.020517928	0.005869508	0.020069978	0.005475972	0.025006662	0.010456436	0.008739242	0.014320035	0.020638319	0.017625098
0.047628521	0.005929407	0.001023362	0.007969644	0.012043256	0.012632618	0.013546136	0.010763504	0.007960283	0.034794306	0.00793543
0.005981455	0.004751307	0.001277302	0.019525144	0.006770018	0.004314829	0.007777822	0.002446156	0.011423756	0.009886404	0.005608468

H114	H115	H116	H117	H118	H119	H120	H121	H122	H123	H124
0.002105027	0	0.000682854	0.000535493	0.003856235	0.006985741	0.007101821	0.005647323	0.012113604	0.012932257	0.005912212
0.000180197	0	0	0	0.000891353	0.000631065	0.001164096	0.001357064	0.001384917	0.001162406	0.001325326
0.000147434	0.000763128	0.000288806	0.000184076	0.00668326	0.003158259	0.00140836	0.006241927	0.000565753	0.001587965	0.001775896
9.56E-05	0.000123583	0.000159088	6.07E-05	0.005334899	0.003108361	0.000750939	0.004597518	0.000493561	0.001219541	0.001416658
9.56E-05	0.000123583	0	0	0.00123883	0.000377171	0.000277764	0.000503565	0.000122285	0.00044329	0.000135983
4.37E-05	0.000262615	0.000252093	0.000179892	0.011053156	0.002135347	0.002251422	0.007914787	0.001096147	0.003382798	0.002301561
0	0	8.81E-05	8.58E-05	0.003995981	0.00118288	0.000544361	0.002037019	0.000172378	0.001274706	0.000635263
0.000163815	0	4.16E-05	0.000117139	0.002998874	0.001585	0.000720232	0.002415404	0.000272563	0.000788072	0.000608879
0.000117401	0	0	0	0.001452226	0.000675093	0.000434093	0.001641564	9.13E-05	0.000421618	0.000373446
0.002356211	0.00118949	0.003955171	0.000598246	0.003527643	0.002726787	0.041136961	0.003880578	0.008347809	0.016458878	0.005879738
0.000286677	0.000590111	0.001539482	0.000368152	0.000315373	0.001132982	0.002067177	0.000344245	0.000658572	0.000169435	0.003143844
0.001528943	0.000274973	0.000313281	0.000386977	0.002405898	0.000249491	0.000432697	0.000873415	0.001731146	0.000258093	0.001146721
0.207354764	0.206900281	0.223831866	0.407206985	0.159958227	0.072422761	0.029008872	0.193781402	0.069335726	0.101322384	0.05931696
0.149612577	0.221211241	0.030390696	0.019394893	0.086701087	0.097298456	0.182568994	0.188489704	0.398962196	0.132382262	0.104891528
0.145528113	0.008088535	0.006348833	0.008287593	0.149088252	0.01652069	0.018199811	0.063190268	0.005231745	0.024729691	0.034959784
0.005067355	0	0.004674739	0.001010325	0.001380464	0.047573482	0.003051215	0.018501027	0.008403795	0.012559893	0.033628369
0.000169276	0.000417094	0.002246812	0	0.047745934	0.019228399	0.001120826	0.019641872	0.003686237	0.038316048	0.002975387
0.000360394	0.000308959	0.00258212	0.002673282	0.005975087	0.005239306	6.56E-05	0.027240863	0.002291006	0.006091795	0.001725156
0	0.00693303	0	0	0.005485976	0.006119861	0	0.002312984	0.000103132	0.000860968	0.002772428
0.034554122	0.035530235	0.091514738	0.046144449	0.174233096	0.060078839	0.091543418	0.097941928	0.032417373	0.118220613	0.168478757
0.088528556	0	0.070192056	0.000598246	0.013838635	0.009489454	0.017587056	0.00018208	0.006302846	0.019201368	0.03598067
0.004556798	0.000160658	0.038252088	0.031064878	0.008533951	0.008002783	0.001367882	0.004219133	0.014504796	0.024394761	0.024466775
3.55E-05	0	0.00086397	0.000796964	0.000264384	0.000974481	8.10E-05	0.00035847	0.000175325	0	0.001315178
0.039719767	0.004649826	5.87E-05	0.00030749	0.001499437	0.000610519	0.324566326	0.002019949	0.006691801	0.203874161	0.007211153
9.01E-05	0.001946439	0.00014685	0.000397436	0.009332769	0.07704421	0.00243148	0.003994378	0.025295363	0.017116918	0.03271911
0	0.003330573	9.79E-05	0.000422538	0.001714722	0.054290654	0.000643463	0.001815109	0.020230102	0.0203224	0.002817079
0.001029307	0.000515961	0.014080508	0.02264132	0.001250161	0.005469718	0.002117426	0.008025742	0.006099528	0.007267992	0.011686411
0.020613434	0.013173993	0.017316113	0.029853743	0.017978139	0.027879857	0.012884612	0.031570951	0.017899317	0.014015856	0.020990077
0.001528943	0.000432542	0.028496325	0.001321999	0.017912043	0.008856921	0.004307433	0.01261757	0.005072627	0.01339525	0.005599654
0	0	0.000337756	0.001391027	0	0.018271528	0.000307076	0.011948995	0.004471514	0.006403083	0.009514744
0.00052967	0	0.000330413	0.000150607	0.003129178	0.003064333	0.002420314	0.004085418	0.00196835	0.00208248	0.002131075
0.011521681	0.016662135	0.033078058	0.051637438	0.01187086	0.006834579	0.002731577	0.021218001	0.004938555	0.020204189	0.005258682
0.00116855	0.005153429	0.006916655	0.008762425	0.000415461	0.000940727	0.000554132	0.001200589	0.001888791	0.001229392	0.000856489
0.001523483	0.002842419	0	0	0.000217173	0.000132083	4.47E-05	0.002827929	3.54E-05	0.000258093	0.000899111
0.010140171	0.001065907	0.000560479	0.000811607	0.011474283	0.004027074	0.000231702	0.063690988	0.001698733	0.015205844	0.003902912
0.001829272	0.000200823	0.000230066	0	0.00141068	0.000466694	3.63E-05	0.006088297	0.000472934	0.001790893	0.000397801
0.001640884	0	0	0	0	0	8.65E-05	0.009644546	3.83E-05	0.001262885	0.000418097
0.00010921	0	0.00168878	0.000535493	0	2.64E-05	0	0.000770995	3.68E-05	0.000977209	0
0.000122862	0.000157569	3.92E-05	5.44E-05	0	0.000557685	0.0001661	0.00013656	0.000453781	0.00054574	0.000416067
0	0	0	0	0	0	0	0	0	0	0
0.006427023	0.016680673	0.007631327	0.002263295	0.018420039	0.038164746	0.005757667	0.010816685	0.0136046	0.008609684	0.021493417
0.049619675	0.117472225	0.01893881	0.014843201	0.102943731	0.28913776	0.084891044	0.066971271	0.20644841	0.065183384	0.230553938
0.012463619	0.018929891	0.000976555	0.003447237	0.012393963	0.047256482	0.008204501	0.004415438	0.032053464	0.009714954	0.025777893
0.000139243	0.001010294	0.003619863	0.003056076	4.72E-05	0	0.000195412	0	9.87E-05	0	0
0.000161085	0.000627186	0.01480497	0.001242511	0	0	3.63E-05	0.000179235	3.68E-05	0.000193078	0.000140042
0	0.000410915	4.90E-05	4.81E-05	0	0	0	0	0	4.33E-05	0
0.000439571	0.00052523	0	7.32E-05	0	0	0	0	0	0	0
0.000562433	0.000271884	0	5.65E-05	0	0	0	8.82E-05	2.21E-05	0	0
0.00021842	0.000305869	0.000511529	0.001152565	0	0	0	4.55E-05	0	0	0
0.000275756	0.000722963	0.000188458	0.000407895	0	0	4.89E-05	0.000247515	0	3.35E-05	0
9.28E-05	0	7.34E-05	6.69E-05	0	0	3.77E-05	0.00011949	0	6.50E-05	0
0.000125592	0	0.000315728	0.000652632	0	0	0	8.25E-05	0	0	0
0.000166546	0.000973219	0.000531109	0.000901553	0	0	0.000142371	0	2.50E-05	0	6.29E-05
0.030393211	0.011867098	0.002714285	0.008743599	0.008456524	0.002500778	0.004699652	0.002691369	0.003026191	0.011722567	0.012169455
0.117941605	0.178136238	0.235831993	0.243155728	0.047687392	0.032545334	0.129030367	0.032253751	0.066344894	0.03525439	0.085054261
0.013451972	0.025285169	0.066031294	0.016899411	0.011268441	0.006482357	0.001224115	0.014751319	0.004749971	0.011257605	0.013478544
0.026308748	0.076878159	0.047559958	0.06151896	0.013217332	0.005377259	0.005770229	0.027858228	0.005479262	0.008438278	0.005680838
0.006757384	0.016893854	0.018625529	0.003489073	0.010401638	0.003165597	0.003548119	0.004509323	0.002354359	0.005352977	0.005603713

H125	H126	H127	H129	H130	H131	H132	H133	H135	H136	H137
0.015785991	0.004394595	0.001861513	0.003136615	0.002039504	0.020954368	0.008000825	0.005773243	0.000976325	0.001312199	0.001412512
0	0.000288203	0.000596786	0.000172071	0	0.002034026	0.001063004	0.000232992	0	0.000211885	8.59E-05
0.168888842	0.000498889	0.00017215	0.000211402	0	0.002859334	0.000321326	0.003859826	0	0	0
0.123040371	0.00027429	0.000174445	7.87E-05	0.000204769	0.000981271	7.28E-05	0.003352605	0	0	0
0.034524547	0	9.41E-05	0	0	0.000477639	0	0.00045155	0	0	0
0.236276951	0.000657897	0.000107881	0.000157322	0.000155625	0.004133037	0.000828577	0.004346428	0	8.55E-05	0
0.082321019	0.00014907	0.000107881	0	0.000155625	0.000812311	0.000191987	0.002595898	0	0	9.97E-05
0.093442967	4.57E-05	0.000188217	0.000223693	0	0.001046256	6.87E-05	0.002024759	0	0	0
0.04350449	0.000137145	0.000107881	0	0	0.000445146	0	0.000542273	0	0	5.54E-05
0.000201976	0.008033901	0.001388675	0.013419107	0.00456636	0.024050896	0.007057055	0.057398412	0.007205886	0.012947282	0.003500813
0.000366745	0.00919665	0.000922723	0.00698118	0.014645114	0.081312304	0.000499167	8.04E-05	0.000222967	0	0.000722874
0	0.000983865	9.18E-05	0.003615956	0.003993005	0.012428354	0.001319661	0.0036557	0.03455312	0.000516702	0.000456989
0.010125358	0.115100235	0.191843771	0.134825274	0.02001417	0.055630288	0.054918516	0.093320357	0.133236264	0.092002647	0.281147956
0.00939984	0.048336573	0.093876745	0.037794242	0.036506305	0.056176161	0.256250707	0.048183903	0.054762033	0.04802724	0.113701639
0.001185278	0.013297079	0.077123124	0.020685434	0.01260561	0.017399696	0.025134189	0.08727907	0.024560147	0.060684574	0.011427495
0.000608584	0.012804153	0.060261622	0.002873591	0.003284503	0.000958527	0.047742228	0.008239243	0.001212805	0.0031411	0.048825815
0.000587324	0.017073529	0.011699302	0.005147391	0.000151529	0.02051572	0.000955895	0.013703206	0.018708954	0.002148587	0.010391653
0	0.004521802	0.020827834	0.009023913	0	0.025548797	0.000353661	0.019517687	0.056268749	8.55E-05	0.013194519
0.000209948	0.002094936	0.008221876	0.001288077	0.041433059	0.000113724	0.008499992	0.007775322	0.001807384	0.024898332	0.006095957
0.036347643	0.102713479	0.106496476	0.077648424	0.018552116	0.046340703	0.055595525	0.044862226	0.077031702	0.116737419	0.003099216
0.000417239	0.01756248	0.013230288	0.018534542	0.145931025	0.061995555	0	7.01E-05	0.028090457	0.014709272	0.032473917
0.005243394	0.008425459	0.007117822	0.018625494	0.01002961	0.030406415	0.001782452	0.001185577	0.014776628	0.003672671	0.005932548
0	9.14E-05	0	8.11E-05	0.00015972	0.000367164	0.000210176	0.000457736	0	0	0.033196791
0	0.002653453	0.000915837	0.001580598	0.002346658	0.001231463	0.078326839	0.005608294	0.001408746	8.55E-05	0.016213417
0	0.008558628	0.001732975	0.004262453	0.000679835	0.002323209	0.06794335	0.008783578	0.00124321	0.001081728	0.010139617
0	0.009252303	0.001817902	0.002148434	0.00252276	0.001926801	0.005359481	0.000113403	0.002047242	0.000364293	0.009533068
0.000159454	0.013471989	0.008811776	0.008783013	0.002117317	0.014572855	0.002827268	0.000612376	0.004675549	0.007014505	0.010660308
0.00151216	0.044631677	0.013466707	0.033372008	0.031956327	0.061537412	0.017879084	0.020231095	0.013448961	0.010962255	0.025981903
0.000855739	0.048775834	0.009032128	0.01098061	0.015509241	0.020899131	0.004593552	0.016610446	0.004678928	0.000193298	0.020414946
0	0.030080419	0.002130067	0.016307447	0.004975899	6.17E-05	0.005056343	0.000136084	0.001331045	0	0.004356629
0	0.011699045	0.001854628	0.003367682	0.006425667	0.002563653	0.001701615	0.000709284	0.000412151	0.000111518	0.000484685
0.003903977	0.027512432	0.016466705	0.02444888	0.00157263	0.008545509	0.00190977	0.003053633	0.007557228	0.011742883	0.030230516
0.001158702	0.003788376	0.002407802	0.003202985	0.000880509	0.00287558	0.000765929	0.000284538	0.002361423	0.001914399	0.010225476
0	0.000123232	7.12E-05	0.000199111	0.000290773	0	0.000218259	0.001305165	0.000844572	0.000156126	7.20E-05
0	0.001804746	0.002593724	0.005707852	0.00047097	0.002069768	0.006260812	0.014635172	0.003912056	0.000643089	0.021409243
0	0.000484976	9.87E-05	0.001025054	0	9.42E-05	0.000470874	0.001148463	0.00027702	0	0.002428966
0	0.002128725	0.000100995	7.87E-05	0	0	0	0.000486602	0	0	0
0	0.000218637	0	0.000437553	0	0	0	0	0	0	0.000484685
0	0.000345843	8.26E-05	8.60E-05	0	0.000695338	0.00059213	0.00012165	0	0.000349424	0
0	0	0	0	0	0	0	0	0	0	0
0.000209948	0.010514432	0.014540922	0.01110106	0.012065018	0.013364136	0.078662312	0.039705482	0.003570849	0.00595508	0.011427495
0.011773054	0.126461387	0.080655638	0.083850367	0.161157685	0.187361095	0.17101434	0.362528763	0.054610011	0.137487268	0.088140719
0.001538735	0.015091887	0.009002288	0.030938428	0.036858508	0.02128904	0.021231792	0.045385941	0.03725575	0.022738594	0.010219936
0.00019666	0.000153046	8.95E-05	0.000833317	0.000176102	0.000500383	0	0	0.000114862	0.000795498	0.001024763
0	0.000238513	0	0.000589959	0.000274391	0	0.000141464	0.000175259	0.000746601	0.000327121	0.000304659
0	0	0	0	0.000905081	0	0	0	0	0.000237906	0.000252036
0	0	8.03E-05	0.000233525	0.00031125	0	0	0	0.000108105	0.000683979	8.03E-05
0	5.57E-05	0.000174445	0.000317103	0.000585641	0	0	6.60E-05	0	0.000338272	0
0.000411924	3.78E-05	0	0.001027512	0.000634785	0	0	0	0.000831059	0.000992513	6.92E-05
0	0.000107331	0.000376434	0.001199583	0.00636014	0	0	6.80E-05	0.003830978	0.006490369	0.00029635
0	0	0.00010329	0.000326936	0.000753552	0	0	0	0.000297289	0.00058733	7.75E-05
0.000201976	0.000109318	0	0.000149948	0	0	0	0	0.00013851	8.92E-05	0
0.000159454	3.18E-05	0.000105585	0.000872648	0.002633336	0	0	0	0.001662117	0.001293613	0
0.0055437	0.006302696	0.026701586	0.010884742	0.01050877	0.007967144	0.014857808	0.003090747	0.040492149	0.058212584	0.005838381
0.031529461	0.215605484	0.136282987	0.302867692	0.249212661	0.108846389	0.022935428	0.038977641	0.219510959	0.269703436	0.108167917
0.04914652	0.011518173	0.026540913	0.035505693	0.088759384	0.049479471	0.013792783	0.013416605	0.048076403	0.039696819	0.007303515
0.018725268	0.027699267	0.034689338	0.035722011	0.024445382	0.018666901	0.009411427	0.008424812	0.082798438	0.023147494	0.02997571
0.010494761	0.013861559	0.012560052	0.01306759	0.020182081	0.006141069	0.003180929	0.005412416	0.008344369	0.015422989	0.008364284

H138	H139	H140	H141	H142	H143	H146	H147	H149	H150
0.003064451	0.006883527	0.02050287	0.005942857	0.006657114	0.001438197	0.000413012	0.000474	0.003780169	0.000814359
0.000195982	0.00035336	0.000773769	0.000208318	0	0	0	0	0.000251493	0.000193751
6.83E-05	5.99E-05	4.41E-05	0	0.000156819	0	0.00351155	0.000117198	7.26E-05	0
5.94E-05	5.59E-05	3.81E-05	0	0	7.94E-05	0.002116446	0	5.96E-05	0
0	0	3.01E-05	0	0	0	0.000631889	0	0	0
0	3.99E-05	5.61E-05	0.000130199	0.000922464	0.000176842	0.003977853	0	6.48E-05	0.000108985
0	0	0	6.94E-05	0.000255215	0	0.001206679	0	0	0
7.13E-05	0	0	0	8.61E-05	0	0.001473138	9.90E-05	0	0
0	0	0	0	0	6.50E-05	0.00070802	0	0	0
0.006930649	0.025441899	0.009453611	0.066375407	0.016773467	0.006467376	0.00033688	0.003851904	0.002769013	0.000432912
0.000971004	0	0.007663519	0.00235226	0.000221391	0.000537745	0	0.001062594	0.000684475	0.000357228
0.001725239	3.39E-05	0.004510311	0.029190597	0.001460568	0.000660452	0.000159875	0.000549528	0.001882307	8.48E-05
0.044847297	0.010445074	0.019909513	0.091448825	0.123723925	0.108813965	0.309307606	0.300721939	0.092134499	0.114748987
0.115917628	0.238831238	0.30631223	0.106323327	0.127828889	0.048582916	0.009740983	0.102253845	0.233691214	0.072777692
0.003013971	0.013729123	0.023848518	0.091625316	0.013458747	0.01807942	0.243661605	0.015178427	0.002678268	0.051519582
0.000679999	0	0.050126589	0.002658951	0	0.015349191	0.002558007	0.010331644	0.002149356	0.001313873
0.000617641	0.00025953	0.007483106	0.000283544	0	0.027466499	0.036723771	0.00674018	0.003917583	0.015839138
0.00052262	3.99E-05	0.001878294	0.000248825	0	0.001757596	0.020513543	0.004148805	0.000246307	0.007268687
0.011364008	0	6.82E-05	0.006923689	6.46E-05	0.000290527	0.023370365	0.00166421	0.002522706	0.028266444
0.051567117	0.055782921	0.021445023	0.074939602	0.131408049	0.062964888	0.01323921	0.068037274	0.015504399	0.158424684
0.066304396	0.000515067	0.000298683	0.004435443	0.0006365	0.008248431	0.000896444	0.001388144	0.001195239	0.023132044
0.023814826	3.19E-05	0.004229669	0.004319711	0.001088507	0.008317002	0.001684402	0.030877734	0.001174497	0.005903349
0.000100961	2.79E-05	0.000543242	0.000167812	0	0.0007597	4.19E-05	0	0.000155563	0
0.006731697	0.011493174	0.022850236	0.003992767	0.00674936	0.003742561	5.71E-05	0.008610137	0.050026316	0
0.000148471	0.01979014	0.008194733	0.001466908	7.69E-05	0.006923918	0	0.000554737	0.001783784	0
0.000733449	0.001353547	0.009798399	0.001895118	0.00012607	0.003100154	3.62E-05	0.000617242	0.003157919	0.000578225
0.000852226	0.003020527	0.01308391	0.000497649	0.006438798	0.00415399	0.000717536	0.013535053	0.010062303	0.001044439
0.015423218	0.023223918	0.052375731	0.022605425	0.039435329	0.142294908	0.025393551	0.03495622	0.046386153	0.012478771
0.004976764	0.006719824	0.083360562	0.003787342	0.004932107	0.018474609	0.047410684	0.052666121	0.020256834	0.001507624
0	0.01222984	0.036457341	0	0.001482092	0.207690475	0	0.009276863	0	0.000611526
0.001027423	0.000179674	0.005825317	0.000112839	0.000276739	0.000729023	4.38E-05	0.006919884	0.002826053	0.00048135
0.003055543	0.001421424	0.005117699	0.00915443	0.029029937	0.015809342	0.013149756	0.038013782	0.017568195	0.007238414
0.001030392	0.00018766	0.000304697	0.00157396	0.00623893	0.004518502	0.002950083	0.000354198	0.001260056	0.002043467
0.000427598	0	3.01E-05	0.000332731	0.001894126	0	0.001433169	0.000104176	0.000982637	0
0.000448384	0.00564577	0.013759454	0.000992405	0.010897373	0.000389775	0.054801117	0.029955777	0.017614863	0.002415832
0.000136594	0.00072868	0.001000287	0	0.000931688	0.000221955	0.00503227	0.002857024	0.002017127	0.000293654
0	0.000193649	4.41E-05	0	0	0.000176842	0.001583528	0.000205747	0.000165933	0.000178614
0	0	0	0	6.46E-05	0	0	0.000992275	0.000147784	0.00021797
0.000190044	0.000644832	0.002207045	0.000494756	0.000412034	0.000149775	0	0	0.000311125	0
0	0	0	0	0	0	0	0	0	0
0.006212047	0.031455004	0.025265758	0.009177577	0.029826331	0.008721213	0.000797474	0.005823432	0.016710008	0.007217222
0.076691461	0.248895002	0.131634917	0.140889693	0.153082874	0.114711115	0.031672468	0.067659637	0.334192384	0.154228765
0.007405758	0.05984556	0.042124296	0.036342857	0.01927027	0.013586182	0.004644001	0.009110182	0.051498975	0.017679772
8.91E-05	0	0	0.000222785	0.003265522	0.000685715	4.00E-05	0	0	0.000348146
0.000288035	0	0.000683563	0.000196745	0.000725672	0.000420452	0	0.000205747	0.000521134	0
9.50E-05	0	0	6.08E-05	0.000531954	0.000160602	0	0	7.52E-05	7.87E-05
0.0005731	2.99E-05	0	7.81E-05	0.000553478	0.00014797	0	0.000213561	0.000303347	0.001026274
0.000864104	0.000175682	0	0.000338517	0.00075642	0	0	0.000218769	0.000420019	0.001861825
0.000424628	0.000692745	0	0.000850633	0.001878751	0.000140752	0	0.000125011	8.04E-05	0.0006418
0.001000698	0.002609272	0.00024055	0.002621338	0.004169537	0.001661957	0	0.000104176	0.001285984	0.002558118
0.000216768	0.000427226	0	0.000509222	0.000387435	0.000232782	0	0.000182308	0.000466688	0.000163477
0.000181135	0.000255537	0	8.10E-05	0.000350536	0	0	0.000104176	0	0
0.001342182	0.000411255	0	0.000708861	0.002032495	0.000526918	0	0	0	0.000675101
0.020209939	0.005943231	0.002700173	0.009290416	0.005138124	0.002028273	0.015378496	0.001976738	0.001389692	0.016780647
0.373904652	0.163569612	0.038920973	0.220730561	0.152200384	0.111683142	0.043453766	0.104058693	0.042320785	0.220512774
0.039647826	0.032578967	0.006569017	0.004493309	0.04552359	0.009888734	0.034449353	0.027861842	0.002691232	0.022553819
0.039950707	0.008833993	0.01358706	0.025322242	0.036083711	0.013569941	0.036065237	0.029148414	0.006551775	0.023552847
0.063884311	0.004913097	0.004648627	0.013534901	0.010494564	0.003403312	0.004617355	0.006091685	0.0019912	0.019844333

Supplementary table S15. The average abundance and composition at the genus level among CC (n=73), SC (n=36), CCR (n=21), and SCR (n=36) groups.				
ID	CC	CCR	SC	SCR
Neisseria	0.136249452	0.160233571	0.130470778	0.246335167
Streptococcus	0.159705603	0.251200905	0.146383083	0.123529778
Prevotella	0.166776082	0.07783519	0.181512167	0.060714889
Leptotrichia	0.108344603	0.041513952	0.128609167	0.11427075
Veillonella	0.121021068	0.073762952	0.069739194	0.083846417
Fusobacterium	0.032644233	0.043462333	0.030828806	0.092341722
Actinomyces	0.056314959	0.010758286	0.019348111	0.025303139
Haemophilus	0.014298795	0.087767857	0.025520889	0.033963306
Rothia	0.028824973	0.034228905	0.026738944	0.023417278
Gemella	0.028267534	0.055736762	0.018227583	0.013151722
Capnocytophaga	0.011803219	0.013759571	0.033944972	0.050026333
Porphyromonas	0.020620329	0.01876719	0.028219139	0.020680611
Lautropia	0.006420123	0.055555857	0.009379361	0.009456583
Lachnoanaerobaculum	0.008327904	0.004973571	0.015894944	0.012829167
Granulicatella	0.010795466	0.01603819	0.009509333	0.0054925
Halomonas	0.009500288	1.10E-06	0.019204222	9.32E-04
TM7x	0.006080795	0.001291952	0.011654389	0.009183167
Alloprevotella	0.003266753	0.007131381	0.00890425	0.003633778
Oribacterium	0.003749041	0.002798381	0.005777333	0.005591222
Selenomonas	0.003756658	0.00183181	0.004333417	0.007308528
Campylobacter	0.00541737	0.00297019	0.003730111	0.003004778
Peptostreptococcus	0.003485548	0.001266286	0.009508056	0.001657111
Stomatobaculum	0.003828384	0.001293524	0.002299111	0.004806167
Solobacterium	0.002532795	0.001141143	0.006853056	0.002663889
Clostridia_UCG-014	0.001860247	5.86E-04	0.004100944	0.006697639
[Eubacterium]_nodatum_group	0.002024288	0.001024	0.005887167	0.003889167
Abiotrophia	0.002960726	0.004241714	5.96E-04	4.19E-04
Tannerella	0.001443342	0.001834762	0.003487611	0.001505806
Atopobium	0.002377932	4.12E-04	0.002557083	0.001101333
Kingella	0.003456767	4.35E-04	1.79E-04	4.05E-04
Lachnospiraceae_uncultured	0.001227863	3.90E-04	0.002392889	0.002633306
Corynebacterium	0.001853384	0.001750952	0.00158125	0.0012985
Megasphaera	0.002227562	0.00107819	0.001672861	7.98E-04
P5D1-392	0.002359836	5.74E-04	0.001024	0.001254
Eikenella	0.002108466	0.001233905	0.001603917	7.28E-04
Lactobacillales_unclassified	0.001405493	0.002098857	5.92E-04	0.001889889
Treponema	9.04E-04	0.00130619	0.002304333	4.65E-04
Absconditabacteriales_(SR1)	4.25E-04	3.31E-04	0.001406694	0.002564361
Actinobacillus	0.001007822	0.001742048	6.98E-04	8.30E-04
F0332	0.001428507	3.97E-04	5.97E-04	7.03E-04
Johnsonella	9.37E-04	6.50E-04	0.001269528	8.08E-04
Enterococcus	9.62E-04	4.80E-04	5.39E-04	0.001273556
Actinobacteria_unclassified	0.001424027	1.33E-04	5.98E-04	4.43E-04
Selenomonadaceae_unclassified	4.28E-04	9.97E-04	6.81E-04	0.001837278
Bergeyella	4.57E-04	0.001396048	0.001283444	5.47E-04
Dialister	5.92E-04	9.78E-04	9.64E-04	6.20E-04
Saccharimonadaceae	6.07E-04	6.47E-04	8.00E-04	9.27E-04
Catonella	5.78E-04	5.27E-04	5.74E-04	8.32E-04
Pelagibacterium	6.48E-04	0	0.001350639	9.54E-05
Others	0.012263041	0.009433429	0.014670361	0.011294667

**Supplementary table S17. The abundance and composition at the genus and phylum level of each sample among CC (n=73), CCR (n=21), and H (n=140) groups.**									
sample	reads	label	ace	chao	shannon	simpson	coverage	observed_otus	
CC01	7455	0.97	135.60751	147.5	2.524982	0.160715	0.995842	101	
CC02	44451	0.97	262.705094	237.066667	2.849096	0.099143	0.998943	165	
CC03	48453	0.97	230.534961	224.12	2.341045	0.195993	0.999113	188	
CC04	34734	0.97	192.784377	206.928571	2.769604	0.107754	0.998877	154	
CC05	31376	0.97	126.917698	133.142857	2.582905	0.13304	0.999363	106	
CC06	54380	0.97	173.429311	170.235294	3.030465	0.07619	0.999559	154	
CC07	25172	0.97	159.025722	154.3125	2.248837	0.264487	0.998967	134	
CC08	36229	0.97	189.742831	159.583333	2.179127	0.173118	0.999034	110	
CC09	29623	0.97	241.632744	228	3.156719	0.078491	0.99865	168	
CC10	35071	0.97	279.369174	284.913043	3.429069	0.064269	0.998489	225	
CC11	27951	0.97	219.865902	190.833333	2.489673	0.156193	0.998497	143	
CC12	47479	0.97	272.713558	212.4	1.342442	0.467968	0.998863	117	
CC13	19591	0.97	291.682994	243	2.391576	0.220811	0.997091	167	
CC15	22672	0.97	170.483962	110	2.506587	0.107933	0.998941	64	
CC16	65473	0.97	393.698474	408.428571	2.477593	0.185261	0.998839	327	
CC17	45610	0.97	485.689225	490.022727	3.884058	0.042297	0.998268	420	
CC18	113725	0.97	344.156927	358.272727	3.34938	0.075944	0.999578	307	
CC19	12117	0.97	91.09363	93.333333	1.046064	0.627054	0.998267	70	
CC20	52561	0.97	367.71902	316.12	2.557275	0.221046	0.998706	225	
CC21	42544	0.97	230.357516	264.545455	3.22275	0.073994	0.999036	190	
CC22	8304	0.97	180.691225	184.153846	3.443264	0.064305	0.995906	141	
CC23	16827	0.97	188.339727	179.391304	2.434734	0.181119	0.997979	155	
CC24	53682	0.97	293.157864	293.12	3.201019	0.105425	0.999106	248	
CC25	8343	0.97	226.184666	218.636364	3.438151	0.064971	0.995206	195	
CC26	26851	0.97	351.888658	294.888889	2.118671	0.351655	0.997505	213	
CC27	48813	0.97	319.578239	323.555556	3.007876	0.166827	0.998996	280	
CC28	16935	0.97	252.227901	250.913043	2.779329	0.157222	0.99687	191	
CC29	55341	0.97	320.326541	323.565217	3.415581	0.070506	0.999006	259	
CC30	25987	0.97	254.25051	250.037037	3.444605	0.059912	0.998653	228	
CC31	17336	0.97	208.825152	210.047619	1.939938	0.398615	0.997635	171	
CC32	13496	0.97	160.405892	159.142857	1.893053	0.331	0.997703	137	
CC33	16698	0.97	215.627705	227.555556	2.338563	0.235778	0.997365	175	
CC34	43841	0.97	317.79303	335.045455	3.870457	0.035621	0.998768	270	
CC35	11676	0.97	153.739469	140.5	1.504863	0.449136	0.997345	94	
CC36	64763	0.97	433.069073	424.136364	3.449841	0.064589	0.998688	343	
CC37	14751	0.97	252.833517	254.12	3.516755	0.069812	0.997424	226	
CC38	13135	0.97	213.887626	153.928571	1.874375	0.237637	0.997031	101	
CC39	32572	0.97	236.159432	237.384615	1.888748	0.342543	0.998649	201	
CC40	60581	0.97	240.274848	195.625	2.983687	0.120696	0.999389	154	
CC41	8255	0.97	229.280817	275	2.934521	0.124152	0.993701	173	
CC42	14069	0.97	139.187242	120.8125	2.401164	0.152428	0.998365	105	
CC43	76210	0.97	286.901436	280.228571	2.655941	0.105055	0.999239	233	
CC44	18457	0.97	281.515044	290.136364	3.525344	0.068021	0.997454	241	
CC45	23573	0.97	320.827186	332.291667	3.166044	0.159358	0.997497	261	
CC46	9778	0.97	222.136276	214.6	2.806427	0.147233	0.995398	175	
CC47	10638	0.97	196.432455	160.588235	1.758022	0.338065	0.996334	117	
CC48	49094	0.97	177.55053	168.136364	2.511787	0.111266	0.999144	129	
CC49	17850	0.97	159.539941	162.083333	2.823122	0.13279	0.998543	135	
CC50	13881	0.97	153.605144	122.1	1.538265	0.435624	0.998055	87	
CC51	41266	0.97	254.650167	243.636364	2.15015	0.259824	0.998764	205	
CC52	47153	0.97	406.803677	405.071429	3.724811	0.050097	0.998855	371	
CC53	47576	0.97	281.571556	252.352941	2.606173	0.182189	0.998991	186	
CC54	20934	0.97	239.768947	221.285714	3.458245	0.059758	0.998089	199	
CC55	43493	0.97	168.930915	181.545455	2.875555	0.081591	0.999149	121	
CC56	24012	0.97	212.62985	192.4	3.097301	0.079944	0.998459	148	
CC57	37653	0.97	419.009505	421.162791	3.641364	0.07791	0.998141	365	
CC58	19236	0.97	257.302617	276.588235	3.052715	0.123426	0.997557	213	
CC59	51820	0.97	268.64596	261.9375	2.981119	0.096204	0.999132	231	
CC60	18762	0.97	231.801474	230.37037	2.577832	0.254973	0.997815	200	
CC61	51264	0.97	223.914345	212.033333	2.846164	0.09038	0.999083	176	
CC62	26361	0.97	218.952315	229.176471	2.506215	0.178341	0.998596	190	
CC63	34039	0.97	244.58363	237	2.759314	0.154714	0.998825	211	
CC64	84521	0.97	275.776983	226.4	0.581201	0.813159	0.999349	167	
CC65	42728	0.97	212.460136	206.125	1.856474	0.424501	0.999298	188	
CC66	40352	0.97	318.535695	322	3.219454	0.087534	0.998587	265	
CC67	30156	0.97	262.215203	265.416667	2.434043	0.307891	0.998541	226	
CC68	15742	0.97	219.654111	230.166667	2.740964	0.147758	0.997268	180	
CC69	30088	0.97	240.153872	226.551724	2.711422	0.134943	0.998704	201	
CC70	48825	0.97	264.104153	244.066667	2.665516	0.207362	0.999099	181	
CC71	65325	0.97	207.556352	209	2.92801	0.100844	0.999388	170	
CC72	35789	0.97	283.972383	298.333333	3.498472	0.062326	0.998603	240	
CC73	35906	0.97	220.871743	213.444444	2.273064	0.238135	0.998914	186	
CC74	73418	0.97	171.978905	210.2	2.954036	0.120552	0.999673	155	
CCR01	29347	0.97	242.408423	214.615385	2.042788	0.287016	0.998433	135	
CCR02	53990	0.97	256.463849	262.05	2.826484	0.176516	0.999222	219	
CCR03	37171	0.97	239.358152	242.285714	2.833193	0.124602	0.998951	207	
CCR04	46893	0.97	210.537994	208.052632	1.974443	0.327864	0.999147	167	

CCR05	31132	0.97	195.878343	189.954545	2.836536	0.096284	0.998779	158	
CCR06	62391	0.97	332.027847	260.111111	2.583695	0.12158	0.999199	124	
CCR07	47454	0.97	343.939355	348.1	2.895174	0.172941	0.998672	283	
CCR08	35288	0.97	264.229914	277.714286	2.564633	0.140767	0.998356	199	
CCR09	38515	0.97	316.188522	333	3.444766	0.068906	0.998546	263	
CCR10	64350	0.97	413.614322	440.5	3.167235	0.094896	0.998834	348	
CCR11	53746	0.97	257.181805	220.136364	2.182154	0.275678	0.999126	171	
CCR12	37745	0.97	185.496661	193.4	1.590581	0.417404	0.99902	149	
CCR13	31776	0.97	254.07519	255.222222	1.528765	0.51749	0.998395	208	
CCR14	42768	0.97	304.161014	267.037037	2.663863	0.123885	0.998691	210	
CCR15	19234	0.97	267.691687	267.333333	3.475638	0.070988	0.997608	229	
CCR16	39196	0.97	348.695886	344.153846	2.961384	0.139521	0.99824	284	
CCR17	44983	0.97	217.601384	219.647059	3.037988	0.090594	0.999066	169	
CCR18	28749	0.97	207.76317	198.681818	2.624575	0.191633	0.998643	165	
CCR19	34880	0.97	131.911836	160.5	1.801049	0.38268	0.999226	102	
CCR20	36077	0.97	186.889821	187.4	3.263439	0.075462	0.99939	172	
CCR21	34311	0.97	229.925978	193.045455	3.416478	0.055769	0.99898	166	
H002	44182	0.97	418.713582	416	3.699402	0.057874	0.998302	341	
H003	25256	0.97	344.93561	339.341463	3.676188	0.052229	0.997941	307	
H004	46291	0.97	380.907425	328.129032	3.083469	0.085105	0.998401	241	
H005	46124	0.97	355.862397	372.555556	3.494152	0.058567	0.998352	267	
H006	33928	0.97	459.301265	467	3.801187	0.059782	0.997613	386	
H008	50924	0.97	403.437828	360.222222	3.417499	0.053912	0.998468	249	
H009	40078	0.97	415.33181	422.410256	3.914814	0.046382	0.998303	364	
H010	33172	0.97	408.102806	407.317073	3.882001	0.043362	0.998252	367	
H011	32252	0.97	446.003816	444.466667	4.143309	0.03684	0.998233	409	
H013	23478	0.97	311.297407	321.25	3.302177	0.089915	0.997402	245	
H014	23771	0.97	254.276271	250	3.393336	0.059212	0.997897	201	
H015	39861	0.97	359.0122	340.055556	3.526607	0.058624	0.99852	245	
H016	30321	0.97	303.889821	294.034483	3.34806	0.074098	0.99812	239	
H017	29082	0.97	345.604009	359.357143	2.65418	0.250202	0.997902	294	
H018	29803	0.97	437.816144	369.651163	3.594266	0.052852	0.997349	298	
H019	47629	0.97	372.110459	317.7	3.488568	0.051559	0.998593	244	
H020	52408	0.97	379.99379	375.8	3.649874	0.053269	0.998779	331	
H021	44461	0.97	440.36	437.5	4.1995	0.031625	0.998695	394	
H023	27682	0.97	354.837116	347	3.805607	0.040967	0.997544	280	
H024	23929	0.97	440.03621	483.730769	3.361027	0.145198	0.996866	377	
H025	49918	0.97	608.646258	621.721311	3.896896	0.044358	0.997836	527	
H026	41157	0.97	535.015471	527.227273	3.864113	0.052862	0.997716	461	
H027	26320	0.97	521.340715	528.72549	3.933518	0.062305	0.996847	462	
H028	25712	0.97	419.290298	455.107143	4.129717	0.031324	0.997083	356	
H029	33946	0.97	589.231541	593.982759	4.438287	0.026878	0.997201	517	
H030	46722	0.97	604.197416	621.170213	4.375987	0.02844	0.998202	547	
H031	45772	0.97	566.718484	563.171875	3.949698	0.062979	0.998187	510	
H032	36906	0.97	541.295006	547.733333	4.623533	0.020207	0.997887	481	
H033	23635	0.97	436.716891	439.219512	4.15068	0.042533	0.997081	382	
H034	33433	0.97	488.732193	526.78125	4.245549	0.030613	0.997547	423	
H035	50620	0.97	440.124318	431.978261	3.700649	0.042183	0.99836	358	
H036	17126	0.97	289.936559	271.875	3.446191	0.076332	0.996672	222	
H038	34885	0.97	324.079608	364.55	3.624085	0.047539	0.998223	270	
H039	38270	0.97	374.102781	364.923077	3.652344	0.049028	0.998171	303	
H040	27833	0.97	322.34474	325	3.979503	0.031803	0.998096	272	
H041	33245	0.97	269.247097	293.5	3.36575	0.057955	0.998376	214	
H042	39412	0.97	361.941103	375.6	3.300093	0.081121	0.998148	288	
H043	43817	0.97	327.286194	337.24	3.603384	0.051772	0.998768	280	
H044	36188	0.97	467.471787	407.833333	3.384362	0.063624	0.99743	289	
H045	31363	0.97	403.314173	399.317073	3.853847	0.046701	0.997896	347	
H046	29779	0.97	286.630971	285.333333	3.061827	0.09883	0.998119	234	
H047	26875	0.97	436.087169	427.32	3.603722	0.05671	0.996688	349	
H048	34909	0.97	367.017797	371.028571	3.40195	0.072673	0.998023	304	
H049	41981	0.97	380.680808	383.30303	3.658843	0.052274	0.998309	308	
H050	45510	0.97	452.463188	402.162162	4.000684	0.033242	0.99844	335	
H051	56479	0.97	500.790249	508	3.949736	0.041336	0.998495	423	
H052	44502	0.97	381.799751	394.617647	3.019068	0.115406	0.998315	313	
H053	8717	0.97	309.071271	333.391304	4.037103	0.04129	0.993232	259	
H054	13490	0.97	435.493728	444.371429	4.808713	0.017356	0.996071	405	
H055	13119	0.97	341.252439	358.391304	4.274174	0.029492	0.995655	289	
H056	34856	0.97	375.301595	378.029412	3.614478	0.052626	0.997992	307	
H057	40713	0.97	341.246128	342.033333	3.526476	0.067303	0.998477	279	
H058	10079	0.97	368.960441	389	4.544121	0.023236	0.994543	334	
H059	12286	0.97	311.855268	319.84	4.235218	0.029548	0.996419	282	
H060	29501	0.97	412.014169	422.2	4.061236	0.042815	0.998339	383	
H061	23259	0.97	309.824581	303.228571	3.299347	0.084408	0.997506	256	
H062	44382	0.97	452.620483	462.363636	4.074878	0.041091	0.998783	419	
H063	45742	0.97	348.872636	363.888889	3.077113	0.143506	0.998492	277	
H064	38339	0.97	372.879052	385.517241	3.112837	0.106887	0.997861	271	
H065	52089	0.97	406.936363	411.692308	3.723511	0.048696	0.998771	360	
H066	26537	0.97	396.480704	408.333333	3.949891	0.070075	0.998267	370	
H067	27830	0.97	311.578069	306.848485	3.2147	0.088234	0.99788	255	
H068	37090	0.97	392.968978	403	2.897433	0.173494	0.997924	326	

H069	26283	0.97	335.18539	354.4	3.394113	0.09502	0.997299	255	
H070	26930	0.97	330.702946	336.964286	3.310997	0.082436	0.997512	258	
H071	30209	0.97	435.281303	439.217391	3.904785	0.035978	0.997087	356	
H072	27873	0.97	335.971978	338.09375	3.813318	0.05264	0.997776	279	
H073	36445	0.97	396.376678	405.176471	3.840216	0.04214	0.998216	344	
H074	31459	0.97	299.519552	290.033333	2.976409	0.123787	0.998029	227	
H075	37042	0.97	372.245968	371.692308	3.362193	0.072541	0.998272	320	
H076	40083	0.97	436.305733	394.896552	3.587233	0.070594	0.998079	294	
H077	23011	0.97	327.685492	329.275862	2.850233	0.17234	0.996958	246	
H078	25401	0.97	344.552404	352.1	3.876766	0.039019	0.99752	287	
H079	27934	0.97	301.748806	297.103448	3.631062	0.047899	0.997995	244	
H080	26245	0.97	433.002275	380.4375	3.643722	0.048642	0.997066	289	
H081	25087	0.97	355.890843	342.5	3.369157	0.065979	0.99721	290	
H082	42683	0.97	374.295249	363.384615	3.696568	0.053222	0.99829	296	
H083	44640	0.97	413.798886	421.026316	3.500399	0.067822	0.99832	348	
H084	40666	0.97	388.42068	402.30303	3.222985	0.095712	0.998254	327	
H085	35737	0.97	333.606929	332.115385	3.977446	0.033302	0.998461	275	
H086	35797	0.97	361.596272	367.3125	3.62004	0.068939	0.998324	312	
H087	44050	0.97	353.819877	327.444444	3.090671	0.092125	0.998502	248	
H088	39983	0.97	380.43709	383.529412	3.611452	0.068028	0.998674	343	
H089	26372	0.97	350.855618	383.181818	3.622669	0.065463	0.997687	300	
H090	21110	0.97	274.730713	275.035714	3.533686	0.052846	0.997395	222	
H091	27107	0.97	416.520579	417.153846	4.217094	0.030342	0.997455	357	
H092	24318	0.97	363.401907	391.576923	3.847173	0.042134	0.99708	296	
H093	31004	0.97	321.177874	320.125	3.393301	0.07095	0.998194	272	
H094	29782	0.97	330.540243	360.954545	3.729384	0.044471	0.997918	275	
H095	33111	0.97	482.116611	438.096774	3.781852	0.042573	0.997252	306	
H096	39006	0.97	417.023146	413.341463	3.541915	0.065407	0.998154	351	
H097	23754	0.97	314.857581	318.033333	3.393218	0.095713	0.99739	255	
H098	50077	0.97	311.686119	314.677419	3.698655	0.038649	0.998882	265	
H099	39062	0.97	409.710969	415.30303	3.995593	0.047114	0.998413	358	
H100	38412	0.97	324.288815	322.193548	3.544484	0.047589	0.998282	253	
H101	44812	0.97	348.522539	348.09375	2.965165	0.131932	0.998505	279	
H102	25711	0.97	295.877713	299.038462	3.479141	0.071852	0.998016	250	
H103	27776	0.97	356.687732	340.166667	3.718208	0.063718	0.99784	291	
H104	37727	0.97	398.270171	398	3.796227	0.044463	0.998622	364	
H105	32470	0.97	416.428974	421.363636	4.064943	0.038265	0.998337	378	
H106	47327	0.97	445.156106	450.078947	3.857419	0.045856	0.998331	369	
H107	38798	0.97	415.287262	416.277778	4.062118	0.03387	0.998634	378	
H108	33537	0.97	360.303661	360.756757	3.555296	0.061401	0.998002	301	
H109	43593	0.97	378.783995	389.65625	3.453687	0.069093	0.998371	312	
H110	40098	0.97	446.003021	433.5625	3.948802	0.042391	0.998055	371	
H111	41856	0.97	356.16014	376.464286	3.288216	0.077505	0.998232	280	
H112	34330	0.97	342.403207	358.615385	3.787234	0.043023	0.998019	271	
H113	42796	0.97	403.283634	404.692308	4.042916	0.036855	0.998738	368	
H114	65401	0.97	367.055333	373	3.60635	0.047148	0.998991	308	
H115	22659	0.97	297.482063	260.625	3.549059	0.044281	0.997617	201	
H116	54448	0.97	375.614415	398.137931	3.751188	0.042729	0.998678	310	
H117	31384	0.97	320.087352	313.46875	3.410102	0.074112	0.99812	260	
H118	35534	0.97	383.348805	388.076923	3.686999	0.048241	0.998227	338	
H119	44373	0.97	487.126723	482.584906	3.701022	0.061269	0.998039	412	
H120	34403	0.97	392.992824	409.941176	3.276819	0.092451	0.997675	317	
H121	41867	0.97	455.626037	443.564516	3.118505	0.141777	0.998041	390	
H122	47318	0.97	442.626868	426.195652	3.312569	0.106497	0.998267	354	
H123	37402	0.97	441.756604	445.878049	3.72312	0.0508	0.997567	346	
H124	38914	0.97	512.228729	476.371429	3.876637	0.047911	0.997739	367	
H125	13782	0.97	385.463424	402.833333	4.247319	0.028965	0.994848	320	
H126	41427	0.97	421.65208	425.085714	4.163017	0.033719	0.998359	360	
H127	24349	0.97	503.061366	378.371429	3.828937	0.037888	0.996386	269	
H129	30574	0.97	505.670366	519.022727	4.163402	0.033715	0.996795	411	
H130	26281	0.97	337.650793	326.717391	3.630579	0.056549	0.998059	299	
H131	49287	0.97	407.459406	400.5	4.044654	0.042099	0.998864	362	
H132	26742	0.97	343.335515	336.625	3.459161	0.071901	0.997532	283	
H133	33174	0.97	357.812443	380.2	3.505306	0.070095	0.99792	302	
H135	24873	0.97	269.392266	267.37037	3.823605	0.041312	0.998352	237	
H136	33568	0.97	293.956548	312.9	3.730852	0.040577	0.998421	244	
H137	35792	0.97	358.688789	395.5	3.356914	0.101934	0.997988	289	
H138	45762	0.97	375.290961	403.777778	3.551026	0.057076	0.998361	301	
H139	51560	0.97	319.685464	355.333333	3.396349	0.064285	0.998836	257	
H140	52568	0.97	348.279894	369	3.910153	0.048043	0.999144	314	
H141	51274	0.97	367.745858	368.027778	3.976094	0.029779	0.999025	334	
H142	41740	0.97	346.22718	359.4	3.90542	0.04003	0.998682	300	
H143	24063	0.97	351.814822	338.466667	3.841079	0.043106	0.997631	303	
H146	41051	0.97	384.941864	353.12	3.313343	0.073508	0.998222	248	
H147	22931	0.97	309.349023	299.026316	3.070559	0.141815	0.997427	254	
H149	31417	0.97	371.937193	384.483871	3.122366	0.109518	0.997836	311	
H150	32212	0.97	287.434271	285.677419	3.439678	0.066554	0.998262	236	

Supplementary table S17. The abundance and composition at the phylum level of each sample among CC (n=73), CCR (n=21), and H (n=140) groups.											
ID	CC64	CC38	CC26	CC39	CC13	CC51	CC12	CC67	CC45	CC04	CC27
Firmicutes	0.986156	0.908252	0.842951	0.835837	0.793284	0.747773	0.715048	0.714336	0.655691	0.623505	0.61675
Bacteroidota	0.003841	0.056779	0.011802	0.03603	0.176666	0.011603	4.91E-04	0.104535	0.169348	0.104568	0.09172
Proteobacteria	0.005044	0.001007	0.023441	0.019838	0.009209	0.08925	0.114399	0.052226	0.076863	0.010805	0.075216
Fusobacteriota	0.001424	0.001617	0.062671	0.062122	0.00335	0.049523	0.140492	0.039041	0.047069	0.234067	0.134845
Actinobacteriota	0.002731	0.032126	0.056043	0.032666	0.015719	0.098239	0.026673	0.078974	0.03678	0.02426	0.034152
Patescibacteria	3.54E-04	1.44E-04	4.37E-04	0.003556	9.70E-05	1.63E-04	1.28E-04	0.003452	0.01046	0.001054	0.039836
Campilobacterota	6.90E-05	0	0.001015	0.005491	5.67E-04	7.94E-04	0	5.71E-04	0.002383	0.001384	0.004742
Spirochaetota	4.00E-05	0	7.46E-04	0.001836	0	0.002228	0	2.80E-05	5.67E-04	4.00E-05	0.002479
Bacteria_unclassified	3.41E-04	7.50E-05	7.08E-04	6.40E-05	0.001014	7.10E-05	6.26E-04	0.006837	7.56E-04	3.17E-04	8.70E-05
Synergistota	0	0	1.45E-04	0.002253	4.80E-05	2.50E-05	4.70E-05	0	8.30E-05	0	9.10E-05
Desulfobacterota	0	0	4.10E-05	3.07E-04	0	0	0	0	0	0	0
Cyanobacteria	0	0	0	0	4.60E-05	3.31E-04	0.001639	0	0	0	8.20E-05
Deferribacterota	0	0	0	0	0	0	0	0	0	0	0
Verrucomicrobiota	0	0	0	0	0	0	0	0	0	0	0
Deinococcota	0	0	0	0	0	0	4.57E-04	0	0	0	0
Chloroflexi	0	0	0	0	0	0	0	0	0	0	0
Elusimicrobiota	0	0	0	0	0	0	0	0	0	0	0
Planctomycetota	0	0	0	0	0	0	0	0	0	0	0

CC15	CC46	CC61	CC43	CC18	CC16	CC42	CC06	CC66	CC11	CC28	CC68	CC37
0.611093	0.575976	0.567355	0.551057	0.544027	0.531666	0.518243	0.506251	0.505618	0.502566	0.501516	0.490069	0.467612
0.10205	0.094827	0.199956	0.019264	0.200461	0.035027	0.41641	0.338354	0.313392	0.033308	0.037912	0.024505	0.09149
0.006962	0.213953	0.144729	0.420238	0.011291	0.358256	0.030897	5.62E-04	0.039708	0.001523	0.12839	0.398277	0.268461
0.035479	0.015621	0.011648	9.86E-04	0.016916	0.03988	0.008032	0.077087	0.053006	0.039878	0.062563	0.069469	0.030206
0.236854	0.016924	0.056741	0.006814	0.203786	0.017692	0.0222	0.027887	0.068962	0.411446	0.251326	0.011285	0.131426
0	0.052738	6.00E-05	4.40E-05	0.007024	3.68E-04	7.30E-05	0.048848	0.015969	0	0.004598	0.003271	0.006167
0.007317	0.014975	0.017476	8.10E-04	0.003044	0.009846	0.001364	5.51E-04	0.002173	1.05E-04	3.34E-04	0.001134	0.001238
0	0.013663	2.48E-04	1.50E-05	0.008659	0.006074	0	3.60E-05	9.10E-05	0	3.61E-04	1.33E-04	3.32E-04
0	0	1.80E-05	0	0.003872	0	0.002781	2.23E-04	9.63E-04	0.010514	0.012878	0.00172	0.002927
0	5.17E-04	2.20E-05	8.10E-05	3.49E-04	1.16E-04	0	0	9.30E-05	0	0	0	0
0	0	0	6.00E-06	7.60E-05	3.20E-05	0	0	2.50E-05	0	6.30E-05	0	1.41E-04
2.45E-04	8.06E-04	0.001747	6.05E-04	4.54E-04	9.00E-04	0	2.01E-04	0	6.60E-04	5.90E-05	1.37E-04	0
0	0	0	0	0	0	0	0	0	0	0	0	0
0	0	0	5.00E-05	0	1.20E-05	0	0	0	0	0	0	0
0	0	0	0	0	5.50E-05	0	0	0	0	0	0	0
0	0	0	1.40E-05	1.80E-05	0	0	0	0	0	0	0	0
0	0	0	1.60E-05	2.30E-05	7.60E-05	0	0	0	0	0	0	0
0	0	0	0	0	0	0	0	0	0	0	0	0

CC59	CC57	CC58	CC55	CC49	CC02	CC21	CC30	CC22	CC36	CC52	CC17	CC34
0.452282	0.450165	0.414019	0.410301	0.4059	0.400847	0.392447	0.391522	0.389522	0.38125	0.375707	0.370691	0.351474
0.120648	0.072782	0.054398	0.110604	0.418733	0.290114	0.11492	0.075524	0.399973	0.142954	0.061072	0.283298	0.352449
0.252032	0.201596	0.241567	0.025078	0.001811	0.006158	0.36411	0.147395	0.027822	0.294753	0.2079	0.02638	0.077244
0.098101	0.096636	0.227464	7.01E-04	0.031742	0.084911	0.101767	0.281668	0.027323	0.086162	0.109023	0.23642	0.081686
0.059715	0.161464	0.049149	0.440893	0.139773	0.216533	0.022997	0.092812	0.147428	0.080496	0.202202	0.057496	0.053422
0.005009	0.010691	0.010242	6.10E-05	8.61E-04	0	0.002845	0.006469	0.005246	0.008532	0.011352	0.007574	0.08146
0.009843	0.004602	6.62E-04	0.01097	4.57E-04	0.001161	7.23E-04	0.003314	0	0.003262	0.006945	0.014136	6.63E-04
0.001964	0.001276	0	0	0	0	8.00E-05	1.61E-04	0	0.001829	0.013007	0.002643	8.70E-04
1.56E-04	2.20E-04	0.002499	0.001392	7.23E-04	2.30E-04	0	0.001096	0.002686	1.18E-04	0	0	6.67E-04
1.66E-04	2.94E-04	0	0	0	2.10E-05	0	3.90E-05	0	6.01E-04	0.003461	4.00E-05	4.30E-05
0	0	0	0	0	0	0	0	0	1.40E-05	5.90E-05	4.21E-04	0
8.40E-05	3.00E-05	0	0	0	2.50E-05	1.11E-04	0	0	1.50E-05	0.009272	7.84E-04	2.20E-05
0	0	0	0	0	0	0	0	0	0	0	0	0
0	2.44E-04	0	0	0	0	0	0	0	0	0	1.17E-04	0
0	0	0	0	0	0	0	0	0	0	0	0	0
0	0	0	0	0	0	0	0	0	1.40E-05	0	0	0
0	0	0	0	0	0	0	0	0	0	0	0	0
0	0	0	0	0	0	0	0	0	0	0	0	0

CC54	CC29	CC48	CC44	CC10	CC09	CC24	CC56	CC03	CC05	CC08	CC53	CC40
0.343802	0.338671	0.319428	0.316538	0.311402	0.298482	0.267657	0.26671	0.251949	0.249519	0.229322	0.2242	0.222987
0.182296	0.260744	0.456594	0.241059	0.220782	0.162083	0.433992	0.315027	0.021677	0.631806	0.199607	0.519472	0.244023
0.002101	0.025192	0.108138	0.12563	0.298103	0.012454	0.028773	0.001747	0.656497	0.001059	0.001843	0.002482	0.40543
0.175267	0.102983	4.26E-04	0.205862	0.021313	0.490728	0.160437	0.21581	0.018683	0.026317	0.328386	0.140642	0.049642
0.277798	0.268471	0.033683	0.099151	0.138582	0.015614	0.079484	0.19845	0.028801	0.003416	0.240238	0.110429	0.048174
0.009165	0.00162	1.10E-04	0.009329	3.76E-04	0.01815	0.00925	0.00182	2.08E-04	0.084137	4.75E-04	9.00E-05	0.002381
0.009002	0.001288	0.08024	0.001159	0.007767	0.001854	0.019561	4.80E-05	0.007067	0.003707	1.29E-04	0.002067	0.027255
5.30E-05	1.86E-04	5.90E-05	4.50E-05	0	5.71E-04	6.10E-04	0	0.003218	0	0	3.77E-04	0
5.16E-04	8.45E-04	0	0.001175	2.50E-05	6.40E-05	2.36E-04	3.88E-04	0	3.90E-05	0	2.21E-04	0
0	0	8.00E-05	0	2.90E-05	0	0	0	2.20E-05	0	0	2.00E-05	0
0	0	0	0	4.70E-05	0	0	0	0	0	0	0	0
0	0	0.001229	5.20E-05	1.03E-04	0	0	0	0.011878	0	0	0	2.00E-05
0	0	0	0	0	0	0	0	0	0	0	0	0
0	0	0	0	0	0	0	0	0	0	0	0	0
0	0	0	0	0.001437	0	0	0	0	0	0	0	8.80E-05
0	0	1.30E-05	0	3.40E-05	0	0	0	0	0	0	0	0
0	0	0	0	0	0	0	0	0	0	0	0	0
0	0	0	0	0	0	0	0	0	0	0	0	0

CC01	CC25	CC74	CC70	CC71	CC33	CC20	CC72	CC41	CC63	CC60	CC69	CC62
0.215858	0.202289	0.196728	0.186543	0.175333	0.169179	0.144917	0.137359	0.131367	0.126491	0.113704	0.108681	0.103903
0.262959	0.106685	0.690353	0.137917	0.072027	0.316854	0.079142	0.290189	0.241712	0.125488	0.102859	0.150511	0.034775
0.011473	0.380398	0.001297	0.506215	0.228525	0.00754	0.028545	0.230602	0.422125	0.543555	0.027913	0.58166	0.583046
0.491865	0.142144	0.002572	0.067989	0.467851	0.473309	0.067357	0.266238	0.171937	0.173679	0.746155	0.111105	0.170231
0.015924	0.165659	0.092706	0.051263	0.036539	0.027924	0.657619	0.036974	0.029936	0.024862	0.006053	0.043327	0.106721
1.41E-04	0.001162	0.005671	0	0.008532	0.001157	0.021246	0.02146	0.001082	0.005442	0.001796	0.002328	5.73E-04
0.001503	9.64E-04	0.010452	0.048493	0.010999	0.002941	5.91E-04	0.01504	0.001475	1.10E-04	6.90E-04	1.88E-04	3.21E-04
0	0	0	5.50E-05	1.34E-04	5.20E-05	4.01E-04	0.001853	0	4.70E-05	3.86E-04	0	3.40E-05
2.77E-04	6.99E-04	2.21E-04	1.68E-04	6.00E-05	9.83E-04	1.31E-04	2.85E-04	3.66E-04	2.51E-04	3.25E-04	0.0022	3.96E-04
0	0	0	0	0	6.10E-05	0	0	0	4.90E-05	1.19E-04	0	0
0	0	0	0	0	0	0	0	0	2.60E-05	0	0	0
0	0	0	0.001357	0	0	5.10E-05	0	0	0	0	0	0
0	0	0	0	0	0	0	0	0	0	0	0	0
0	0	0	0	0	0	0	0	0	0	0	0	0
0	0	0	0	0	0	0	0	0	0	0	0	0
0	0	0	0	0	0	0	0	0	0	0	0	0
0	0	0	0	0	0	0	0	0	0	0	0	0
0	0	0	0	0	0	0	0	0	0	0	0	0

CC65	CC23	CC07	CC47	CC31	CC73	CC32	CC50	CC35	CC19	ID...75	CCR12	CCR13
0.095362	0.074038	0.071974	0.069915	0.066801	0.061818	0.04317	0.023426	0.021146	0.014768	Firmicutes	0.901118	0.812541
0.035886	0.326473	0.017214	0.333713	0.147885	0.043574	0.107462	0.864062	0.873214	0.921853	Bacteroidota	0.033812	0.029645
0.784173	0.504283	0.087471	0.006377	0.003399	0.780261	0.794214	0.001428	0.002372	0.00989	Proteobacter	0.028655	0.105148
0.066081	0.071604	0.601499	0.56602	0.760612	0.096746	0.049641	7.82E-04	0.001554	0.051388	Fusobacterio	0.023543	0.036187
0.013706	0.022696	0.220511	0.022633	0.018071	0.010474	0.003378	0.108966	0.09973	0.001001	Actinobacter	0.008779	0.010791
0.004264	1.18E-04	0.001004	7.46E-04	9.97E-04	0.001829	0.001162	7.09E-04	4.25E-04	0	Patescibacte	5.44E-04	0.00248
2.02E-04	3.59E-04	1.55E-04	4.96E-04	9.27E-04	0.005156	1.47E-04	2.73E-04	5.32E-04	0.0011	Campilobact	0.003294	0.001892
0	0	0	0	1.49E-04	5.00E-05	0	0	0	0	Spirochaetot	1.93E-04	4.82E-04
3.26E-04	4.29E-04	1.72E-04	1.00E-04	0.001159	9.20E-05	7.48E-04	3.54E-04	0.001027	0	Bacteria_unc	6.20E-05	0
0	0	0	0	0	0	0	0	0	0	Synergistota	0	6.32E-04
0	0	0	0	0	0	0	0	0	0	Desulfobacte	0	1.44E-04
0	0	0	0	0	0	7.80E-05	0	0	0	Cyanobacter	0	0
0	0	0	0	0	0	0	0	0	0	Deferribacte	0	0
0	0	0	0	0	0	0	0	0	0	Verrucomicr	0	0
0	0	0	0	0	0	0	0	0	0	Deinococcot	0	0
0	0	0	0	0	0	0	0	0	0	Chloroflexi	0	2.90E-05
0	0	0	0	0	0	0	0	0	0	Elusimicrobi	0	2.90E-05
0	0	0	0	0	0	0	0	0	0	Planctomyce	0	0

CCR11	CCR18	CCR02	CCR07	CCR03	CCR20	CCR14	CCR05	CCR09	CCR15	CCR10	CCR06	CCR21
0.658783	0.655849	0.593072	0.575019	0.543691	0.499253	0.432086	0.421706	0.377883	0.328217	0.321792	0.277796	0.274269
0.027122	0.131169	0.09632	0.099656	0.048961	0.127931	0.075455	0.101223	0.082112	0.07528	0.072812	0.102428	0.212021
0.206566	0.133306	0.179093	0.165691	0.217659	0.098338	0.445623	0.199202	0.334328	0.103796	0.434612	0.523796	0.40495
0.010629	0.075504	0.108747	0.055619	0.171704	0.259244	0.033393	0.257662	0.088353	0.079704	0.070509	0.060715	0.103035
0.089774	0.0016	0.010359	0.095665	0.011498	4.52E-04	0.00573	0.012341	0.099255	0.399761	0.08811	0.03463	0.00428
0.005133	3.01E-04	0.003057	0.001862	0.002213	0.004471	0.002275	0.004397	0.003414	0.004768	0.003131	4.50E-05	6.33E-04
2.49E-04	0.001503	0.005674	0.003247	0.003529	0.00714	0.0017	0.003113	0.007274	0.005968	0.003306	4.20E-04	7.90E-04
0	1.73E-04	0.003659	8.64E-04	5.55E-04	0.003065	0.003621	8.80E-05	0.005734	0.001347	0.004693	1.50E-05	2.20E-05
0.001744	4.24E-04	1.90E-05	0.001959	0	1.06E-04	7.30E-05	2.68E-04	0.001578	9.68E-04	8.54E-04	8.30E-05	0
0	1.71E-04	0	3.18E-04	1.90E-04	0	4.40E-05	0	4.30E-05	9.20E-05	1.81E-04	0	0
0	0	0	5.10E-05	0	0	0	0	0	0	0	1.80E-05	0
0	0	0	2.00E-05	0	0	0	0	0	9.90E-05	0	2.00E-05	0
0	0	0	0	0	0	0	0	0	0	0	0	0
0	0	0	0	0	0	0	0	0	0	0	0	0
0	0	0	0	0	0	0	0	2.60E-05	0	0	3.40E-05	0
0	0	0	0	0	0	0	0	0	0	0	0	0
0	0	0	2.90E-05	0	0	0	0	0	0	0	0	0
0	0	0	0	0	0	0	0	0	0	0	0	0

CCR16	CCR04	CCR01	CCR08	CCR17	CCR19	ID...97	H045	H139	H031	H085	H125	H066
0.269345	0.268618	0.240891	0.238617	0.184453	0.073784	Firmicutes	0.510898	0.47481	0.450832	0.431463	0.414038	0.392922
0.043757	0.039354	0.058491	0.025716	0.165874	0.898012	Bacteroidota	0.20526	0.178282	0.223834	0.323963	0.436224	0.232286
0.544997	0.592733	0.661538	0.642682	0.522589	0.001368	Proteobacter	0.076185	0.188042	0.180078	0.0067	0.023857	0.223855
0.094233	0.080832	0.01814	0.028094	0.11512	0.009782	Fusobacterio	0.098696	0.025917	0.071626	0.070652	0.070044	0.06236
0.037945	0.012041	0.019902	0.063104	0.001264	0.014009	Actinobacter	0.082598	0.10497	0.058952	0.142495	0.028126	0.065451
0.004252	0.003317	2.59E-04	2.67E-04	0.007407	1.40E-04	Patescibacte	0.002028	0.018561	0.005936	0.008621	0.021976	0.004466
0.003106	0.002601	5.42E-04	7.52E-04	0.003238	0.00286	Campilobact	0.015319	0.004516	0.002703	0.011978	0.00222	0.006844
0.002042	1.97E-04	1.72E-04	1.33E-04	5.50E-05	2.20E-05	Spirochaeto	0.005946	0.003351	0.004996	0.003922	0	0.009553
0	3.07E-04	3.00E-05	5.99E-04	0	0	Bacteria_un	0	1.06E-04	0	0	0	0
1.13E-04	0	3.50E-05	3.60E-05	0	2.30E-05	Synergistota	0.001485	0.001304	4.30E-05	2.06E-04	0	9.90E-04
1.90E-05	0	0	0	0	0	Desulfobacte	3.78E-04	1.03E-04	7.03E-04	0	0.001389	4.91E-04
1.91E-04	0	0	0	0	0	Cyanobacter	0.001207	3.80E-05	1.33E-04	0	6.50E-04	7.40E-05
0	0	0	0	0	0	Deferribacte	0	0	1.41E-04	0	2.04E-04	0
0	0	0	0	0	0	Verrucomicr	0	0	2.30E-05	0	0.001272	1.39E-04
0	0	0	0	0	0	Deinococcot	0	0	0	0	0	4.26E-04
0	0	0	0	0	0	Chloroflexi	0	0	0	0	0	1.43E-04
0	0	0	0	0	0	Elusimicrobi	0	0	0	0	0	0
0	0	0	0	0	0	Planctomyce	0	0	0	0	0	0

H053	H055	H142	H056	H136	H130	H054	H119	H091	H059	H058	H079	H041
0.38549	0.385149	0.383229	0.382462	0.372097	0.355892	0.348023	0.344488	0.33873	0.337931	0.336644	0.321211	0.320728
0.404875	0.319634	0.263717	0.478761	0.365374	0.46382	0.352816	0.257742	0.338604	0.320443	0.358051	0.451127	0.401712
0.12694	0.177919	0.122032	0.010276	0.118215	0.029692	0.104507	0.267897	0.094945	0.205073	0.12807	0.023133	0.013435
0.003003	0.007702	0.13065	0.030088	0.062638	0.064949	0.109093	0.077071	0.082242	0.03191	0.077142	0.105658	0.160239
0.074938	0.104811	0.077275	0.075121	0.042161	0.044956	0.041932	0.010283	0.083781	0.093962	0.076515	0.036639	0.048492
0.002409	0	0.018017	0.004429	0.020783	0.034216	0.01066	0.028181	0.036472	0.006282	0.010101	0.040726	0.05061
0	0.001944	0.004605	0.010729	0.017885	0.003309	0.016397	0.009512	0.018693	0.001238	0.007054	0.020756	0.004784
0	0	2.77E-04	0.007453	5.90E-04	0.002775	0.009697	0.003584	0.006165	4.37E-04	0.003768	5.45E-04	0
0	0	1.34E-04	0	2.57E-04	7.30E-05	0	0	0	0	0	2.50E-05	0
0	0	4.10E-05	3.59E-04	0	3.18E-04	0.004862	8.82E-04	4.90E-05	0	0	1.80E-04	0
0.001774	0.001405	0	2.35E-04	0	0	0.001114	6.90E-05	0	6.84E-04	0.001173	0	0
1.14E-04	3.76E-04	2.30E-05	8.70E-05	0	0	3.06E-04	3.10E-05	2.80E-04	3.07E-04	4.96E-04	0	0
0	0	0	0	0	0	0	0	0	0	0	0	0
4.57E-04	0.00106	0	0	0	0	5.93E-04	2.19E-04	0	0.001733	9.86E-04	0	0
0	0	0	0	0	0	0	0	0	0	0	0	0
0	0	0	0	0	0	0	4.10E-05	3.90E-05	0	0	0	0
0	0	0	0	0	0	0	0	0	0	0	0	0
0	0	0	0	0	0	0	0	0	0	0	0	0

H100	H073	H140	H011	H029	H115	H016	H080	H042	H141	H135	H050	H133
0.313098	0.310908	0.303439	0.300677	0.296156	0.295913	0.295072	0.29463	0.292866	0.292296	0.290983	0.29087	0.284923
0.438478	0.463831	0.24304	0.401447	0.427958	0.25044	0.429421	0.382323	0.546506	0.33089	0.306606	0.354495	0.249437
0.067327	0.057402	0.315797	0.077027	0.167154	0.169943	0.04848	0.207728	0.02365	0.158398	0.193323	0.174717	0.298025
0.078656	0.065351	0.043247	0.058332	0.035893	0.217802	0.172578	0.043105	0.045164	0.109892	0.093917	0.061823	0.064784
0.0326	0.050584	0.053981	0.145928	0.03364	0.048823	0.020573	0.047863	0.057222	0.097332	0.056893	0.080911	0.06731
0.055689	0.034308	0.013178	0.001578	0.018169	0.012847	0.024087	0.01387	0.017684	0.006098	0.042321	0.015782	0.032442
0.013671	0.010023	0.008449	0.012827	0.013291	0.004057	0.008187	0.007263	0.015448	0.003831	0.015724	0.007657	0.002134
4.24E-04	0.006492	0.017417	0.00139	0.001982	4.30E-05	0.001449	0.00314	0.001238	9.05E-04	2.33E-04	0.011048	5.35E-04
0	2.32E-04	0	0	0	1.32E-04	5.80E-05	4.20E-05	4.80E-05	1.39E-04	0	0	0
5.70E-05	8.14E-04	0.001399	2.90E-05	7.45E-04	0	6.70E-05	3.60E-05	1.24E-04	1.40E-04	0	0.002376	2.78E-04
0	2.80E-05	0	2.96E-04	0.003507	0	2.80E-05	0	2.30E-05	7.90E-05	0	1.14E-04	6.70E-05
0	0	0	7.60E-05	4.14E-04	0	0	0	0	0	0	1.79E-04	0
0	0	0	0	0.001031	0	0	0	2.70E-05	0	0	0	0
0	0	0	3.93E-04	2.80E-05	0	0	0	0	0	0	0	6.50E-05
0	0	0	0	0	0	0	0	0	0	0	0	0
0	2.70E-05	5.30E-05	0	3.20E-05	0	0	0	0	0	0	2.80E-05	0
0	0	0	0	0	0	0	0	0	0	0	0	0
0	0	0	0	0	0	0	0	0	0	0	0	0

H126	H034	H030	H129	H032	H019	H003	H028	H039	H008	H116	H124	H107
0.284056	0.28365	0.283069	0.28185	0.278085	0.276012	0.271844	0.270202	0.270083	0.269977	0.265468	0.263015	0.262069
0.400387	0.319109	0.410327	0.362936	0.341562	0.511812	0.326878	0.332531	0.39256	0.516332	0.367916	0.314144	0.283541
0.091859	0.169998	0.090153	0.132921	0.048452	0.028586	0.258397	0.21748	0.038379	0.042386	0.057868	0.296078	0.292866
0.107858	0.048784	0.09322	0.125325	0.178288	0.103299	0.07199	0.066161	0.251996	0.087664	0.226159	0.061338	0.055826
0.040537	0.037973	0.079503	0.059601	0.103008	0.044156	0.064545	0.024586	0.025264	0.045484	0.039236	0.031751	0.086252
0.020789	0.08922	0.02446	0.025106	0.031521	0.012106	0.001652	0.02614	0.012478	0.025031	0.034848	0.01314	0.01002
0.010683	0.016572	0.011773	0.009636	0.012654	0.023361	0.004419	0.050569	0.007313	0.01277	0.007546	0.012788	0.008498
0.039131	9.67E-04	0.003896	0.001939	0.002904	6.45E-04	1.17E-04	3.01E-04	9.01E-04	3.19E-04	9.39E-04	0.007088	5.58E-04
0	0	8.10E-05	6.90E-05	0	2.30E-05	0	0	7.20E-05	0	2.00E-05	0	0
0.004147	1.62E-04	0.001525	2.73E-04	1.02E-04	0	0	3.20E-05	7.23E-04	1.90E-05	0	4.66E-04	5.90E-05
4.51E-04	0.007197	0.001004	1.60E-04	0.00245	0	4.30E-05	0.002784	2.31E-04	0	0	1.47E-04	6.20E-05
7.70E-05	1.93E-04	9.28E-04	1.52E-04	3.00E-05	0	3.20E-05	1.57E-04	0	0	0	0	1.06E-04
0	0.026175	1.70E-05	0	9.18E-04	0	0	0.009057	0	0	0	0	0
0	0	4.40E-05	0	0	0	8.30E-05	0	0	1.80E-05	0	2.20E-05	1.43E-04
0	0	0	0	2.60E-05	0	0	0	0	0	0	2.30E-05	0
2.50E-05	0	0	0	0	0	0	0	0	0	0	0	0
0	0	0	3.20E-05	0	0	0	0	0	0	0	0	0
0	0	0	0	0	0	0	0	0	0	0	0	0

H110	H010	H043	H062	H131	H117	H104	H098	H122	H036	H071	H150	H103
0.259407	0.257722	0.253226	0.252984	0.252042	0.250993	0.249171	0.247769	0.244289	0.244157	0.243989	0.237685	0.235738
0.240821	0.359817	0.42748	0.214338	0.237632	0.299365	0.289925	0.323498	0.186859	0.470398	0.429933	0.319133	0.425456
0.266286	0.248821	0.139252	0.33082	0.156781	0.06976	0.326483	0.198648	0.451079	0.095981	0.084977	0.292551	0.100855
0.14075	0.050788	0.124544	0.089353	0.235287	0.294925	0.065052	0.103535	0.073924	0.136084	0.147451	0.058811	0.146932
0.06841	0.05569	0.03361	0.071458	0.065354	0.057031	0.03605	0.044058	0.023416	0.03405	0.020734	0.056538	0.035129
0.015032	0.018144	0.008588	0.029939	0.036909	0.010574	0.027242	0.062277	0.009643	0.013425	0.043359	0.028784	0.042001
0.007848	0.007806	0.010136	0.005784	0.010023	0.015971	0.002964	0.019176	0.008082	0.005488	0.027274	0.006083	0.005473
0.001072	2.01E-04	0.002429	0.00428	0.005414	0.001047	0.002651	9.72E-04	0.00262	2.42E-04	0.002097	2.00E-04	0.006339
0	0	0	0	0	0	0	0	0	5.20E-05	0	1.34E-04	7.00E-05
2.43E-04	0	3.76E-04	7.68E-04	4.64E-04	2.95E-04	2.61E-04	0	3.50E-05	5.70E-05	1.86E-04	4.80E-05	0.001367
4.90E-05	4.91E-04	9.10E-05	9.30E-05	1.80E-05	3.90E-05	7.20E-05	0	3.10E-05	6.60E-05	0	0	5.68E-04
5.60E-05	1.44E-04	2.68E-04	7.80E-05	3.60E-05	0	2.70E-05	6.70E-05	0	0	0	0	0
0	0	0	0	0	0	0	0	0	0	0	0	0
2.60E-05	2.91E-04	0	1.05E-04	1.60E-05	0	1.02E-04	0	2.20E-05	0	0	0	0
0	8.50E-05	0	0	0	0	0	0	0	0	0	0	0
0	0	0	0	2.40E-05	0	0	0	0	0	0	0	7.20E-05
0	0	0	0	0	0	0	0	0	0	0	3.30E-05	0
0	0	0	0	0	0	0	0	0	0	0	0	0

H101	H005	H143	H070	H021	H149	H095	H047	H088	H132	H044	H061	H092
0.235405	0.233691	0.232256	0.229192	0.228056	0.227664	0.227407	0.227075	0.227002	0.224196	0.224138	0.22375	0.22308
0.133677	0.279834	0.407551	0.160249	0.377446	0.115233	0.365525	0.309688	0.300383	0.182311	0.201654	0.286512	0.402828
0.50221	0.14836	0.123359	0.426412	0.128066	0.537353	0.164832	0.297897	0.322132	0.46504	0.255098	0.276294	0.15896
0.034575	0.232783	0.167659	0.131003	0.163081	0.071831	0.146579	0.113037	0.075947	0.046466	0.186554	0.187374	0.149432
0.078447	0.065573	0.021488	0.038133	0.050627	0.029715	0.026217	0.017709	0.047574	0.054126	0.115889	0.015313	0.013398
0.004079	0.015611	0.024834	0.005259	0.022167	0.005187	0.054741	0.026435	0.012637	0.01479	0.005822	0.005928	0.037262
0.006282	0.02308	0.003808	0.008794	0.019804	0.004916	0.012481	0.004859	0.005433	0.008972	0.00976	0.003471	0.008232
0.004248	9.02E-04	0.007333	7.38E-04	0.009312	0.005052	0.002048	0.001325	0.005792	0.003506	0.001003	5.73E-04	0.004738
0	3.20E-05	1.86E-04	2.20E-04	0	2.41E-04	0	0	1.21E-04	0	0	2.21E-04	0
9.83E-04	1.06E-04	0.008882	0	7.38E-04	0.001795	5.60E-05	0.001851	0.002484	3.64E-04	8.20E-05	4.38E-04	0.001513
7.40E-05	0	0.002351	0	4.99E-04	9.09E-04	2.10E-05	4.20E-05	4.95E-04	1.52E-04	0	1.26E-04	5.12E-04
2.00E-05	2.80E-05	0	0	0	3.40E-05	3.00E-05	3.90E-05	0	7.70E-05	0	0	0
0	0	0	0	0	0	0	0	0	0	0	0	0
0	0	0	0	0	0	6.30E-05	0	0	0	0	0	0
0	0	0	0	0	0	0	0	0	0	0	0	0
0	0	2.93E-04	0	0	7.00E-05	0	4.30E-05	0	0	0	0	4.50E-05
0	0	0	0	2.04E-04	0	0	0	0	0	0	0	0
0	0	0	0	0	0	0	0	0	0	0	0	0

H051	H108	H105	H065	H026	H113	H138	H025	H024	H127	H002	H096	H038
0.221357	0.219555	0.21866	0.217701	0.216853	0.216618	0.215404	0.215353	0.2141	0.212268	0.209637	0.208528	0.208163
0.368989	0.305294	0.3764	0.353437	0.504682	0.446428	0.366506	0.317271	0.223581	0.338555	0.289585	0.192233	0.479683
0.230205	0.340572	0.269351	0.290669	0.085732	0.195243	0.258295	0.260087	0.505341	0.225246	0.311842	0.418806	0.097417
0.11432	0.066381	0.076789	0.075715	0.081095	0.05705	0.075343	0.115329	0.022627	0.140715	0.068404	0.091442	0.126968
0.021848	0.025276	0.03723	0.038016	0.031973	0.035253	0.056063	0.017093	0.012286	0.032401	0.074762	0.037449	0.048492
0.023986	0.030352	0.006077	0.017157	0.037209	0.041476	0.020483	0.059665	0.012209	0.03408	0.024979	0.040622	0.030094
0.012141	0.010016	0.006921	0.004625	0.040304	0.006276	0.00736	0.008103	0.004942	0.015604	0.016384	0.00553	0.008378
0.00591	0.002273	0.006804	0.002456	6.32E-04	0.001229	3.18E-04	0.006185	3.70E-05	9.24E-04	0.003825	0.004763	6.48E-04
0	0	0	0	0	0	1.41E-04	0	0	0	0	0	3.10E-05
8.10E-04	1.18E-04	0.001214	1.00E-04	0	0	6.60E-05	2.12E-04	0	1.20E-04	4.80E-04	4.18E-04	1.26E-04
2.38E-04	0	1.74E-04	1.40E-05	0.001345	6.90E-05	2.10E-05	4.61E-04	0.004546	0	2.20E-05	4.80E-05	0
2.20E-05	7.10E-05	2.90E-04	0	8.30E-05	3.27E-04	0	6.00E-05	4.40E-05	8.70E-05	5.60E-05	3.20E-05	0
0	0	0	0	1.90E-05	0	0	4.30E-05	2.87E-04	0	0	0	0
1.70E-05	0	6.50E-05	1.10E-04	7.30E-05	3.10E-05	0	1.38E-04	0	0	0	0	0
0	0	2.50E-05	0	0	0	0	0	0	0	2.40E-05	0	0
0	9.20E-05	0	0	0	0	0	0	0	0	0	1.29E-04	0
1.57E-04	0	0	0	0	0	0	0	0	0	0	0	0
0	0	0	0	0	0	0	0	0	0	0	0	0

H084	H060	H040	H094	H014	H035	H078	H015	H109	H090	H057	H111	H099
0.206818	0.199169	0.198584	0.197276	0.196894	0.195689	0.195041	0.194038	0.188923	0.187473	0.18434	0.184325	0.181717
0.166955	0.500047	0.394044	0.404729	0.311657	0.457496	0.417197	0.313544	0.313516	0.328087	0.272819	0.457614	0.442603
0.518987	0.144623	0.199554	0.19223	0.190607	0.168399	0.133218	0.254596	0.350375	0.294129	0.320528	0.241468	0.21858
0.073309	0.068718	0.11157	0.108712	0.190616	0.122693	0.135832	0.175734	0.088201	0.11032	0.099064	0.062329	0.069591
0.017179	0.05407	0.049603	0.044956	0.034449	0.016763	0.022896	0.02166	0.030508	0.029825	0.055702	0.023845	0.027344
0.006217	0.007225	0.037925	0.040256	0.045402	0.029743	0.08294	0.028327	0.025076	0.031468	0.033893	0.026148	0.027858
0.004016	0.016549	0.007409	0.011032	0.030296	0.00514	0.010084	0.011861	0.002689	0.01842	0.033236	0.001831	0.030779
0.00445	0.006695	8.90E-04	5.92E-04	3.90E-05	0.003406	0.002383	1.94E-04	5.03E-04	1.91E-04	3.91E-04	0.002213	9.58E-04
0	0	0	1.00E-04	4.00E-05	8.70E-05	1.18E-04	1.50E-05	7.30E-05	3.90E-05	0	0	0
0.001126	7.39E-04	3.93E-04	1.17E-04	0	5.49E-04	2.91E-04	0	1.08E-04	4.80E-05	0	1.55E-04	9.80E-05
9.43E-04	0.00108	2.80E-05	0	0	3.50E-05	0	0	0	0	0	7.20E-05	0
0	1.08E-04	0	0	0	0	0	3.10E-05	2.80E-05	0	0	0	4.11E-04
0	0	0	0	0	0	0	0	0	0	0	0	0
0	4.60E-04	0	0	0	0	0	0	0	0	2.70E-05	0	6.10E-05
0	0	0	0	0	0	0	0	0	0	0	0	0
0	5.70E-05	0	0	0	0	0	0	0	0	0	0	0
0	0	0	0	0	0	0	0	0	0	0	0	0
0	4.60E-04	0	0	0	0	0	0	0	0	0	0	0

H118	H009	H093	H114	H081	H018	H120	H106	H023	H076	H083	H072	H027
0.180066	0.174967	0.174481	0.171305	0.170141	0.169747	0.168849	0.168739	0.168511	0.168471	0.168041	0.166143	0.165561
0.432748	0.391925	0.356857	0.386043	0.409268	0.394644	0.287926	0.440686	0.368819	0.303324	0.295907	0.328127	0.297021
0.215612	0.140801	0.32749	0.234813	0.253368	0.251006	0.430223	0.201139	0.312841	0.106885	0.382932	0.198538	0.418729
0.106572	0.219141	0.096557	0.101296	0.110095	0.115921	0.036386	0.114041	0.095467	0.340088	0.103524	0.248141	0.029012
0.018754	0.018669	0.018751	0.081309	0.046697	0.024913	0.061181	0.024297	0.025322	0.023017	0.026637	0.012977	0.034013
0.039385	0.034792	0.020502	0.013121	0.005159	0.036986	0.002396	0.023071	0.018383	0.032281	0.016291	0.028943	0.041539
0.005327	0.018928	0.004619	0.011828	0.004677	0.005479	0.005181	0.017906	0.008928	0.025082	0.00422	0.014704	0.006771
0.001302	4.30E-04	4.18E-04	2.27E-04	3.59E-04	0.001045	0.006355	0.009038	0.001527	8.01E-04	0.001808	0.002171	9.80E-05
0	0	2.58E-04	0	0	0	0	0	0	0	8.50E-05	0	0
1.05E-04	0	3.70E-05	4.70E-05	0	3.70E-05	0.001382	4.80E-04	2.02E-04	5.10E-05	4.68E-04	1.40E-04	0
4.30E-05	8.10E-05	3.00E-05	1.10E-05	3.70E-05	4.10E-05	1.21E-04	3.17E-04	0	0	8.70E-05	1.16E-04	0.00599
0	5.20E-05	0	0	1.61E-04	7.10E-05	0	0	0	0	0	0	6.24E-04
0	0	0	0	0	0	0	0	0	0	0	0	5.14E-04
8.60E-05	2.14E-04	0	0	3.80E-05	7.10E-05	0	1.60E-05	0	0	0	0	1.28E-04
0	0	0	0	0	3.90E-05	0	0	0	0	0	0	0
0	0	0	0	0	0	0	1.50E-05	0	0	0	0	0
0	0	0	0	0	0	0	2.55E-04	0	0	0	0	0
0	0	0	0	0	0	0	0	0	0	0	0	0

H020	H086	H049	H064	H033	H013	H112	H075	H089	H067	H082	H046	H063
0.164621	0.164073	0.163408	0.162027	0.159756	0.15591	0.152914	0.148174	0.142648	0.141324	0.139871	0.134099	0.131764
0.277265	0.331664	0.279564	0.235088	0.313848	0.330752	0.490049	0.192271	0.298045	0.26311	0.337168	0.320539	0.148999
0.31657	0.325461	0.336361	0.429469	0.401793	0.357491	0.156273	0.565776	0.178128	0.38252	0.246177	0.380428	0.570739
0.089879	0.102761	0.117809	0.143336	0.068225	0.125776	0.097798	0.04212	0.302676	0.154953	0.190783	0.116348	0.054591
0.089592	0.025702	0.030207	0.019255	0.017428	0.016163	0.047069	0.021019	0.028778	0.03704	0.045728	0.016286	0.06789
0.04854	0.030699	0.032009	0.001635	0.017725	0.008554	0.035142	0.021428	0.035032	0.013478	0.028672	0.028664	0.017611
0.011239	0.013587	0.017742	0.00743	0.014743	0.00455	0.019152	0.003033	0.012892	0.005937	0.010721	0.002058	0.006046
0.00199	0.004177	0.014924	0.001463	0.001231	6.83E-04	0.001331	0.005918	0.001444	0.001077	7.66E-04	0.001236	0.001855
0	0	0	6.80E-05	0	3.60E-05	0	0	0	4.20E-05	0	0	0
8.30E-05	0.001664	0.006672	1.22E-04	2.16E-04	8.50E-05	2.16E-04	2.10E-04	2.77E-04	3.71E-04	9.00E-05	2.95E-04	2.50E-04
1.26E-04	1.90E-04	6.94E-04	0	0.004114	0	2.20E-05	1.90E-05	8.00E-05	1.48E-04	2.40E-05	4.70E-05	0
5.80E-05	0	5.63E-04	7.90E-05	7.65E-04	0	0	3.20E-05	0	0	0	0	2.55E-04
0	0	0	0	1.56E-04	0	0	0	0	0	0	0	0
1.50E-05	0	0	0	0	0	3.40E-05	0	0	0	0	0	0
0	0	0	0	0	0	0	0	0	0	0	0	0
0	2.20E-05	4.70E-05	2.80E-05	0	0	0	0	0	0	0	0	0
2.20E-05	0	0	0	0	0	0	0	0	0	0	0	0
0	0	0	0	0	0	0	0	0	0	0	0	0

H137	H147	H097	H102	H123	H048	H052	H121	H068	H006	H017	H069	H146
0.128927	0.12467	0.12463	0.119205	0.110677	0.108017	0.103826	0.10133	0.101277	0.097792	0.096519	0.0944	0.094322
0.261211	0.238073	0.265745	0.299216	0.240725	0.215718	0.277502	0.227624	0.201127	0.213087	0.160059	0.235318	0.459739
0.422182	0.428378	0.429916	0.364338	0.476858	0.518465	0.541767	0.529852	0.597827	0.565796	0.637032	0.485205	0.129856
0.148625	0.150179	0.102718	0.160192	0.083638	0.045089	0.01804	0.090032	0.074136	0.05382	0.06548	0.111949	0.195881
0.016083	0.030035	0.033783	0.014051	0.045827	0.099075	0.016133	0.015321	0.008606	0.033445	0.02379	0.037748	0.044743
0.014931	0.018967	0.035157	0.020449	0.030104	0.006888	0.032184	0.028855	0.010036	0.01875	0.01067	0.023365	0.066904
0.006231	0.007493	0.007182	0.020206	0.009862	0.002461	0.009172	0.004005	0.004888	0.008856	0.001947	0.011024	0.008483
0.001468	0.001813	5.32E-04	0.002257	0.001926	0.002267	0.00129	0.002352	0.001605	0.00714	0.003968	7.27E-04	0
0	1.72E-04	2.13E-04	5.40E-05	0	6.48E-04	0	2.00E-05	0	0	2.80E-05	3.70E-05	0
3.09E-04	2.20E-04	1.24E-04	3.20E-05	2.98E-04	0.001099	0	4.54E-04	4.47E-04	0.001041	3.84E-04	1.80E-04	0
3.30E-05	0	0	0	0	2.73E-04	4.30E-05	1.33E-04	0	6.20E-05	1.23E-04	4.70E-05	3.10E-05
0	0	0	0	2.30E-05	0	1.40E-05	0	0	1.21E-04	0	0	0
0	0	0	0	0	0	0	0	0	0	0	0	0
0	0	0	0	4.60E-05	0	2.90E-05	2.20E-05	2.30E-05	5.70E-05	0	0	4.10E-05
0	0	0	0	0	0	0	0	0	0	0	0	0
0	0	0	0	0	0	0	0	2.80E-05	3.30E-05	0	0	0
0	0	0	0	1.60E-05	0	0	0	0	0	0	0	0
0	0	0	0	0	0	0	0	0	0	0	0	0

H077	H074	H087	H004
0.08319	0.073136	0.069366	0.049219
0.245328	0.322669	0.253435	0.220714
0.535264	0.38164	0.540408	0.582023
0.10091	0.177868	0.066348	0.078461
0.01913	0.010237	0.021856	0.031781
0.009936	0.023028	0.041019	0.033072
0.005514	0.009467	0.006765	0.003876
5.06E-04	0.001766	5.42E-04	7.44E-04
1.20E-04	0	0	0
5.30E-05	1.89E-04	1.75E-04	8.10E-05
4.90E-05	0	6.30E-05	0
0	0	2.30E-05	0
0	0	0	0
0	0	0	0
0	0	0	0
0	0	0	0
0	0	0	2.90E-05
0	0	0	0

Supplementary table S17. The abundance and composition at the phylum level of each sample among CC (n=73), CCR (n=21), and H (n=140) groups.											
ID	CC64	CC38	CC26	CC39	CC13	CC51	CC12	CC67	CC45	CC04	CC27
Firmicutes	0.986156	0.908252	0.842951	0.835837	0.793284	0.747773	0.715048	0.714336	0.655691	0.623505	0.61675
Bacteroidota	0.003841	0.056779	0.011802	0.03603	0.176666	0.011603	4.91E-04	0.104535	0.169348	0.104568	0.09172
Proteobacteria	0.005044	0.001007	0.023441	0.019838	0.009209	0.08925	0.114399	0.052226	0.076863	0.010805	0.075216
Fusobacteriota	0.001424	0.001617	0.062671	0.062122	0.00335	0.049523	0.140492	0.039041	0.047069	0.234067	0.134845
Actinobacteriota	0.002731	0.032126	0.056043	0.032666	0.015719	0.098239	0.026673	0.078974	0.03678	0.02426	0.034152
Patescibacteria	3.54E-04	1.44E-04	4.37E-04	0.003556	9.70E-05	1.63E-04	1.28E-04	0.003452	0.01046	0.001054	0.039836
Campilobacterota	6.90E-05	0	0.001015	0.005491	5.67E-04	7.94E-04	0	5.71E-04	0.002383	0.001384	0.004742
Spirochaetota	4.00E-05	0	7.46E-04	0.001836	0	0.002228	0	2.80E-05	5.67E-04	4.00E-05	0.002479
Bacteria_unclassified	3.41E-04	7.50E-05	7.08E-04	6.40E-05	0.001014	7.10E-05	6.26E-04	0.006837	7.56E-04	3.17E-04	8.70E-05
Synergistota	0	0	1.45E-04	0.002253	4.80E-05	2.50E-05	4.70E-05	0	8.30E-05	0	9.10E-05
Desulfobacterota	0	0	4.10E-05	3.07E-04	0	0	0	0	0	0	0
Cyanobacteria	0	0	0	0	4.60E-05	3.31E-04	0.001639	0	0	0	8.20E-05
Deferribacterota	0	0	0	0	0	0	0	0	0	0	0
Verrucomicrobiota	0	0	0	0	0	0	0	0	0	0	0
Deinococcota	0	0	0	0	0	0	4.57E-04	0	0	0	0
Chloroflexi	0	0	0	0	0	0	0	0	0	0	0
Elusimicrobiota	0	0	0	0	0	0	0	0	0	0	0
Planctomycetota	0	0	0	0	0	0	0	0	0	0	0

CC15	CC46	CC61	CC43	CC18	CC16	CC42	CC06	CC66	CC11	CC28	CC68	CC37
0.611093	0.575976	0.567355	0.551057	0.544027	0.531666	0.518243	0.506251	0.505618	0.502566	0.501516	0.490069	0.467612
0.10205	0.094827	0.199956	0.019264	0.200461	0.035027	0.41641	0.338354	0.313392	0.033308	0.037912	0.024505	0.09149
0.006962	0.213953	0.144729	0.420238	0.011291	0.358256	0.030897	5.62E-04	0.039708	0.001523	0.12839	0.398277	0.268461
0.035479	0.015621	0.011648	9.86E-04	0.016916	0.03988	0.008032	0.077087	0.053006	0.039878	0.062563	0.069469	0.030206
0.236854	0.016924	0.056741	0.006814	0.203786	0.017692	0.0222	0.027887	0.068962	0.411446	0.251326	0.011285	0.131426
0	0.052738	6.00E-05	4.40E-05	0.007024	3.68E-04	7.30E-05	0.048848	0.015969	0	0.004598	0.003271	0.006167
0.007317	0.014975	0.017476	8.10E-04	0.003044	0.009846	0.001364	5.51E-04	0.002173	1.05E-04	3.34E-04	0.001134	0.001238
0	0.013663	2.48E-04	1.50E-05	0.008659	0.006074	0	3.60E-05	9.10E-05	0	3.61E-04	1.33E-04	3.32E-04
0	0	1.80E-05	0	0.003872	0	0.002781	2.23E-04	9.63E-04	0.010514	0.012878	0.00172	0.002927
0	5.17E-04	2.20E-05	8.10E-05	3.49E-04	1.16E-04	0	0	9.30E-05	0	0	0	0
0	0	0	6.00E-06	7.60E-05	3.20E-05	0	0	2.50E-05	0	6.30E-05	0	1.41E-04
2.45E-04	8.06E-04	0.001747	6.05E-04	4.54E-04	9.00E-04	0	2.01E-04	0	6.60E-04	5.90E-05	1.37E-04	0
0	0	0	0	0	0	0	0	0	0	0	0	0
0	0	0	5.00E-05	0	1.20E-05	0	0	0	0	0	0	0
0	0	0	0	0	5.50E-05	0	0	0	0	0	0	0
0	0	0	1.40E-05	1.80E-05	0	0	0	0	0	0	0	0
0	0	0	1.60E-05	2.30E-05	7.60E-05	0	0	0	0	0	0	0
0	0	0	0	0	0	0	0	0	0	0	0	0

CC59	CC57	CC58	CC55	CC49	CC02	CC21	CC30	CC22	CC36	CC52	CC17	CC34
0.452282	0.450165	0.414019	0.410301	0.4059	0.400847	0.392447	0.391522	0.389522	0.38125	0.375707	0.370691	0.351474
0.120648	0.072782	0.054398	0.110604	0.418733	0.290114	0.11492	0.075524	0.399973	0.142954	0.061072	0.283298	0.352449
0.252032	0.201596	0.241567	0.025078	0.001811	0.006158	0.36411	0.147395	0.027822	0.294753	0.2079	0.02638	0.077244
0.098101	0.096636	0.227464	7.01E-04	0.031742	0.084911	0.101767	0.281668	0.027323	0.086162	0.109023	0.23642	0.081686
0.059715	0.161464	0.049149	0.440893	0.139773	0.216533	0.022997	0.092812	0.147428	0.080496	0.202202	0.057496	0.053422
0.005009	0.010691	0.010242	6.10E-05	8.61E-04	0	0.002845	0.006469	0.005246	0.008532	0.011352	0.007574	0.08146
0.009843	0.004602	6.62E-04	0.01097	4.57E-04	0.001161	7.23E-04	0.003314	0	0.003262	0.006945	0.014136	6.63E-04
0.001964	0.001276	0	0	0	0	8.00E-05	1.61E-04	0	0.001829	0.013007	0.002643	8.70E-04
1.56E-04	2.20E-04	0.002499	0.001392	7.23E-04	2.30E-04	0	0.001096	0.002686	1.18E-04	0	0	6.67E-04
1.66E-04	2.94E-04	0	0	0	2.10E-05	0	3.90E-05	0	6.01E-04	0.003461	4.00E-05	4.30E-05
0	0	0	0	0	0	0	0	0	1.40E-05	5.90E-05	4.21E-04	0
8.40E-05	3.00E-05	0	0	0	2.50E-05	1.11E-04	0	0	1.50E-05	0.009272	7.84E-04	2.20E-05
0	0	0	0	0	0	0	0	0	0	0	0	0
0	2.44E-04	0	0	0	0	0	0	0	0	0	1.17E-04	0
0	0	0	0	0	0	0	0	0	0	0	0	0
0	0	0	0	0	0	0	0	0	1.40E-05	0	0	0
0	0	0	0	0	0	0	0	0	0	0	0	0
0	0	0	0	0	0	0	0	0	0	0	0	0

CC54	CC29	CC48	CC44	CC10	CC09	CC24	CC56	CC03	CC05	CC08	CC53	CC40
0.343802	0.338671	0.319428	0.316538	0.311402	0.298482	0.267657	0.26671	0.251949	0.249519	0.229322	0.2242	0.222987
0.182296	0.260744	0.456594	0.241059	0.220782	0.162083	0.433992	0.315027	0.021677	0.631806	0.199607	0.519472	0.244023
0.002101	0.025192	0.108138	0.12563	0.298103	0.012454	0.028773	0.001747	0.656497	0.001059	0.001843	0.002482	0.40543
0.175267	0.102983	4.26E-04	0.205862	0.021313	0.490728	0.160437	0.21581	0.018683	0.026317	0.328386	0.140642	0.049642
0.277798	0.268471	0.033683	0.099151	0.138582	0.015614	0.079484	0.19845	0.028801	0.003416	0.240238	0.110429	0.048174
0.009165	0.00162	1.10E-04	0.009329	3.76E-04	0.01815	0.00925	0.00182	2.08E-04	0.084137	4.75E-04	9.00E-05	0.002381
0.009002	0.001288	0.08024	0.001159	0.007767	0.001854	0.019561	4.80E-05	0.007067	0.003707	1.29E-04	0.002067	0.027255
5.30E-05	1.86E-04	5.90E-05	4.50E-05	0	5.71E-04	6.10E-04	0	0.003218	0	0	3.77E-04	0
5.16E-04	8.45E-04	0	0.001175	2.50E-05	6.40E-05	2.36E-04	3.88E-04	0	3.90E-05	0	2.21E-04	0
0	0	8.00E-05	0	2.90E-05	0	0	0	2.20E-05	0	0	2.00E-05	0
0	0	0	0	4.70E-05	0	0	0	0	0	0	0	0
0	0	0.001229	5.20E-05	1.03E-04	0	0	0	0.011878	0	0	0	2.00E-05
0	0	0	0	0	0	0	0	0	0	0	0	0
0	0	0	0	0	0	0	0	0	0	0	0	0
0	0	0	0	0.001437	0	0	0	0	0	0	0	8.80E-05
0	0	1.30E-05	0	3.40E-05	0	0	0	0	0	0	0	0
0	0	0	0	0	0	0	0	0	0	0	0	0
0	0	0	0	0	0	0	0	0	0	0	0	0

CC01	CC25	CC74	CC70	CC71	CC33	CC20	CC72	CC41	CC63	CC60	CC69	CC62
0.215858	0.202289	0.196728	0.186543	0.175333	0.169179	0.144917	0.137359	0.131367	0.126491	0.113704	0.108681	0.103903
0.262959	0.106685	0.690353	0.137917	0.072027	0.316854	0.079142	0.290189	0.241712	0.125488	0.102859	0.150511	0.034775
0.011473	0.380398	0.001297	0.506215	0.228525	0.00754	0.028545	0.230602	0.422125	0.543555	0.027913	0.58166	0.583046
0.491865	0.142144	0.002572	0.067989	0.467851	0.473309	0.067357	0.266238	0.171937	0.173679	0.746155	0.111105	0.170231
0.015924	0.165659	0.092706	0.051263	0.036539	0.027924	0.657619	0.036974	0.029936	0.024862	0.006053	0.043327	0.106721
1.41E-04	0.001162	0.005671	0	0.008532	0.001157	0.021246	0.02146	0.001082	0.005442	0.001796	0.002328	5.73E-04
0.001503	9.64E-04	0.010452	0.048493	0.010999	0.002941	5.91E-04	0.01504	0.001475	1.10E-04	6.90E-04	1.88E-04	3.21E-04
0	0	0	5.50E-05	1.34E-04	5.20E-05	4.01E-04	0.001853	0	4.70E-05	3.86E-04	0	3.40E-05
2.77E-04	6.99E-04	2.21E-04	1.68E-04	6.00E-05	9.83E-04	1.31E-04	2.85E-04	3.66E-04	2.51E-04	3.25E-04	0.0022	3.96E-04
0	0	0	0	0	6.10E-05	0	0	0	4.90E-05	1.19E-04	0	0
0	0	0	0	0	0	0	0	0	2.60E-05	0	0	0
0	0	0	0.001357	0	0	5.10E-05	0	0	0	0	0	0
0	0	0	0	0	0	0	0	0	0	0	0	0
0	0	0	0	0	0	0	0	0	0	0	0	0
0	0	0	0	0	0	0	0	0	0	0	0	0
0	0	0	0	0	0	0	0	0	0	0	0	0
0	0	0	0	0	0	0	0	0	0	0	0	0
0	0	0	0	0	0	0	0	0	0	0	0	0

CC65	CC23	CC07	CC47	CC31	CC73	CC32	CC50	CC35	CC19	ID...75	CCR12	CCR13
0.095362	0.074038	0.071974	0.069915	0.066801	0.061818	0.04317	0.023426	0.021146	0.014768	Firmicutes	0.901118	0.812541
0.035886	0.326473	0.017214	0.333713	0.147885	0.043574	0.107462	0.864062	0.873214	0.921853	Bacteroidota	0.033812	0.029645
0.784173	0.504283	0.087471	0.006377	0.003399	0.780261	0.794214	0.001428	0.002372	0.00989	Proteobacter	0.028655	0.105148
0.066081	0.071604	0.601499	0.56602	0.760612	0.096746	0.049641	7.82E-04	0.001554	0.051388	Fusobacterio	0.023543	0.036187
0.013706	0.022696	0.220511	0.022633	0.018071	0.010474	0.003378	0.108966	0.09973	0.001001	Actinobacter	0.008779	0.010791
0.004264	1.18E-04	0.001004	7.46E-04	9.97E-04	0.001829	0.001162	7.09E-04	4.25E-04	0	Patescibacte	5.44E-04	0.00248
2.02E-04	3.59E-04	1.55E-04	4.96E-04	9.27E-04	0.005156	1.47E-04	2.73E-04	5.32E-04	0.0011	Campilobact	0.003294	0.001892
0	0	0	0	1.49E-04	5.00E-05	0	0	0	0	Spirochaeto	1.93E-04	4.82E-04
3.26E-04	4.29E-04	1.72E-04	1.00E-04	0.001159	9.20E-05	7.48E-04	3.54E-04	0.001027	0	Bacteria_un	6.20E-05	0
0	0	0	0	0	0	0	0	0	0	Synergistota	0	6.32E-04
0	0	0	0	0	0	0	0	0	0	Desulfobact	0	1.44E-04
0	0	0	0	0	0	7.80E-05	0	0	0	Cyanobacter	0	0
0	0	0	0	0	0	0	0	0	0	Deferribacte	0	0
0	0	0	0	0	0	0	0	0	0	Verrucomicr	0	0
0	0	0	0	0	0	0	0	0	0	Deinococco	0	0
0	0	0	0	0	0	0	0	0	0	Chloroflexi	0	2.90E-05
0	0	0	0	0	0	0	0	0	0	Elusimicrob	0	2.90E-05
0	0	0	0	0	0	0	0	0	0	Planctomyce	0	0

CCR11	CCR18	CCR02	CCR07	CCR03	CCR20	CCR14	CCR05	CCR09	CCR15	CCR10	CCR06	CCR21
0.658783	0.655849	0.593072	0.575019	0.543691	0.499253	0.432086	0.421706	0.377883	0.328217	0.321792	0.277796	0.274269
0.027122	0.131169	0.09632	0.099656	0.048961	0.127931	0.075455	0.101223	0.082112	0.07528	0.072812	0.102428	0.212021
0.206566	0.133306	0.179093	0.165691	0.217659	0.098338	0.445623	0.199202	0.334328	0.103796	0.434612	0.523796	0.40495
0.010629	0.075504	0.108747	0.055619	0.171704	0.259244	0.033393	0.257662	0.088353	0.079704	0.070509	0.060715	0.103035
0.089774	0.0016	0.010359	0.095665	0.011498	4.52E-04	0.00573	0.012341	0.099255	0.399761	0.08811	0.03463	0.00428
0.005133	3.01E-04	0.003057	0.001862	0.002213	0.004471	0.002275	0.004397	0.003414	0.004768	0.003131	4.50E-05	6.33E-04
2.49E-04	0.001503	0.005674	0.003247	0.003529	0.00714	0.0017	0.003113	0.007274	0.005968	0.003306	4.20E-04	7.90E-04
0	1.73E-04	0.003659	8.64E-04	5.55E-04	0.003065	0.003621	8.80E-05	0.005734	0.001347	0.004693	1.50E-05	2.20E-05
0.001744	4.24E-04	1.90E-05	0.001959	0	1.06E-04	7.30E-05	2.68E-04	0.001578	9.68E-04	8.54E-04	8.30E-05	0
0	1.71E-04	0	3.18E-04	1.90E-04	0	4.40E-05	0	4.30E-05	9.20E-05	1.81E-04	0	0
0	0	0	5.10E-05	0	0	0	0	0	0	0	1.80E-05	0
0	0	0	2.00E-05	0	0	0	0	0	9.90E-05	0	2.00E-05	0
0	0	0	0	0	0	0	0	0	0	0	0	0
0	0	0	0	0	0	0	0	0	0	0	0	0
0	0	0	0	0	0	0	0	2.60E-05	0	0	3.40E-05	0
0	0	0	0	0	0	0	0	0	0	0	0	0
0	0	0	2.90E-05	0	0	0	0	0	0	0	0	0
0	0	0	0	0	0	0	0	0	0	0	0	0

CCR16	CCR04	CCR01	CCR08	CCR17	CCR19	ID...97	H045	H139	H031	H085	H125	H066
0.269345	0.268618	0.240891	0.238617	0.184453	0.073784	Firmicutes	0.510898	0.47481	0.450832	0.431463	0.414038	0.392922
0.043757	0.039354	0.058491	0.025716	0.165874	0.898012	Bacteroidota	0.20526	0.178282	0.223834	0.323963	0.436224	0.232286
0.544997	0.592733	0.661538	0.642682	0.522589	0.001368	Proteobacter	0.076185	0.188042	0.180078	0.0067	0.023857	0.223855
0.094233	0.080832	0.01814	0.028094	0.11512	0.009782	Fusobacterio	0.098696	0.025917	0.071626	0.070652	0.070044	0.06236
0.037945	0.012041	0.019902	0.063104	0.001264	0.014009	Actinobacter	0.082598	0.10497	0.058952	0.142495	0.028126	0.065451
0.004252	0.003317	2.59E-04	2.67E-04	0.007407	1.40E-04	Patescibacte	0.002028	0.018561	0.005936	0.008621	0.021976	0.004466
0.003106	0.002601	5.42E-04	7.52E-04	0.003238	0.00286	Campilobact	0.015319	0.004516	0.002703	0.011978	0.00222	0.006844
0.002042	1.97E-04	1.72E-04	1.33E-04	5.50E-05	2.20E-05	Spirochaetot	0.005946	0.003351	0.004996	0.003922	0	0.009553
0	3.07E-04	3.00E-05	5.99E-04	0	0	Bacteria_un	0	1.06E-04	0	0	0	0
1.13E-04	0	3.50E-05	3.60E-05	0	2.30E-05	Synergistota	0.001485	0.001304	4.30E-05	2.06E-04	0	9.90E-04
1.90E-05	0	0	0	0	0	Desulfobact	3.78E-04	1.03E-04	7.03E-04	0	0.001389	4.91E-04
1.91E-04	0	0	0	0	0	Cyanobacter	0.001207	3.80E-05	1.33E-04	0	6.50E-04	7.40E-05
0	0	0	0	0	0	Deferribacte	0	0	1.41E-04	0	2.04E-04	0
0	0	0	0	0	0	Verrucomicr	0	0	2.30E-05	0	0.001272	1.39E-04
0	0	0	0	0	0	Deinococcot	0	0	0	0	0	4.26E-04
0	0	0	0	0	0	Chloroflexi	0	0	0	0	0	1.43E-04
0	0	0	0	0	0	Elusimicrobi	0	0	0	0	0	0
0	0	0	0	0	0	Planctomyce	0	0	0	0	0	0

H053	H055	H142	H056	H136	H130	H054	H119	H091	H059	H058	H079	H041
0.38549	0.385149	0.383229	0.382462	0.372097	0.355892	0.348023	0.344488	0.33873	0.337931	0.336644	0.321211	0.320728
0.404875	0.319634	0.263717	0.478761	0.365374	0.46382	0.352816	0.257742	0.338604	0.320443	0.358051	0.451127	0.401712
0.12694	0.177919	0.122032	0.010276	0.118215	0.029692	0.104507	0.267897	0.094945	0.205073	0.12807	0.023133	0.013435
0.003003	0.007702	0.13065	0.030088	0.062638	0.064949	0.109093	0.077071	0.082242	0.03191	0.077142	0.105658	0.160239
0.074938	0.104811	0.077275	0.075121	0.042161	0.044956	0.041932	0.010283	0.083781	0.093962	0.076515	0.036639	0.048492
0.002409	0	0.018017	0.004429	0.020783	0.034216	0.01066	0.028181	0.036472	0.006282	0.010101	0.040726	0.05061
0	0.001944	0.004605	0.010729	0.017885	0.003309	0.016397	0.009512	0.018693	0.001238	0.007054	0.020756	0.004784
0	0	2.77E-04	0.007453	5.90E-04	0.002775	0.009697	0.003584	0.006165	4.37E-04	0.003768	5.45E-04	0
0	0	1.34E-04	0	2.57E-04	7.30E-05	0	0	0	0	0	2.50E-05	0
0	0	4.10E-05	3.59E-04	0	3.18E-04	0.004862	8.82E-04	4.90E-05	0	0	1.80E-04	0
0.001774	0.001405	0	2.35E-04	0	0	0.001114	6.90E-05	0	6.84E-04	0.001173	0	0
1.14E-04	3.76E-04	2.30E-05	8.70E-05	0	0	3.06E-04	3.10E-05	2.80E-04	3.07E-04	4.96E-04	0	0
0	0	0	0	0	0	0	0	0	0	0	0	0
4.57E-04	0.00106	0	0	0	0	5.93E-04	2.19E-04	0	0.001733	9.86E-04	0	0
0	0	0	0	0	0	0	0	0	0	0	0	0
0	0	0	0	0	0	0	4.10E-05	3.90E-05	0	0	0	0
0	0	0	0	0	0	0	0	0	0	0	0	0
0	0	0	0	0	0	0	0	0	0	0	0	0

H100	H073	H140	H011	H029	H115	H016	H080	H042	H141	H135	H050	H133
0.313098	0.310908	0.303439	0.300677	0.296156	0.295913	0.295072	0.29463	0.292866	0.292296	0.290983	0.29087	0.284923
0.438478	0.463831	0.24304	0.401447	0.427958	0.25044	0.429421	0.382323	0.546506	0.33089	0.306606	0.354495	0.249437
0.067327	0.057402	0.315797	0.077027	0.167154	0.169943	0.04848	0.207728	0.02365	0.158398	0.193323	0.174717	0.298025
0.078656	0.065351	0.043247	0.058332	0.035893	0.217802	0.172578	0.043105	0.045164	0.109892	0.093917	0.061823	0.064784
0.0326	0.050584	0.053981	0.145928	0.03364	0.048823	0.020573	0.047863	0.057222	0.097332	0.056893	0.080911	0.06731
0.055689	0.034308	0.013178	0.001578	0.018169	0.012847	0.024087	0.01387	0.017684	0.006098	0.042321	0.015782	0.032442
0.013671	0.010023	0.008449	0.012827	0.013291	0.004057	0.008187	0.007263	0.015448	0.003831	0.015724	0.007657	0.002134
4.24E-04	0.006492	0.017417	0.00139	0.001982	4.30E-05	0.001449	0.00314	0.001238	9.05E-04	2.33E-04	0.011048	5.35E-04
0	2.32E-04	0	0	0	1.32E-04	5.80E-05	4.20E-05	4.80E-05	1.39E-04	0	0	0
5.70E-05	8.14E-04	0.001399	2.90E-05	7.45E-04	0	6.70E-05	3.60E-05	1.24E-04	1.40E-04	0	0.002376	2.78E-04
0	2.80E-05	0	2.96E-04	0.003507	0	2.80E-05	0	2.30E-05	7.90E-05	0	1.14E-04	6.70E-05
0	0	0	7.60E-05	4.14E-04	0	0	0	0	0	0	1.79E-04	0
0	0	0	0	0.001031	0	0	0	2.70E-05	0	0	0	0
0	0	0	3.93E-04	2.80E-05	0	0	0	0	0	0	0	6.50E-05
0	0	0	0	0	0	0	0	0	0	0	0	0
0	2.70E-05	5.30E-05	0	3.20E-05	0	0	0	0	0	0	2.80E-05	0
0	0	0	0	0	0	0	0	0	0	0	0	0
0	0	0	0	0	0	0	0	0	0	0	0	0

H126	H034	H030	H129	H032	H019	H003	H028	H039	H008	H116	H124	H107
0.284056	0.28365	0.283069	0.28185	0.278085	0.276012	0.271844	0.270202	0.270083	0.269977	0.265468	0.263015	0.262069
0.400387	0.319109	0.410327	0.362936	0.341562	0.511812	0.326878	0.332531	0.39256	0.516332	0.367916	0.314144	0.283541
0.091859	0.169998	0.090153	0.132921	0.048452	0.028586	0.258397	0.21748	0.038379	0.042386	0.057868	0.296078	0.292866
0.107858	0.048784	0.09322	0.125325	0.178288	0.103299	0.07199	0.066161	0.251996	0.087664	0.226159	0.061338	0.055826
0.040537	0.037973	0.079503	0.059601	0.103008	0.044156	0.064545	0.024586	0.025264	0.045484	0.039236	0.031751	0.086252
0.020789	0.08922	0.02446	0.025106	0.031521	0.012106	0.001652	0.02614	0.012478	0.025031	0.034848	0.01314	0.01002
0.010683	0.016572	0.011773	0.009636	0.012654	0.023361	0.004419	0.050569	0.007313	0.01277	0.007546	0.012788	0.008498
0.039131	9.67E-04	0.003896	0.001939	0.002904	6.45E-04	1.17E-04	3.01E-04	9.01E-04	3.19E-04	9.39E-04	0.007088	5.58E-04
0	0	8.10E-05	6.90E-05	0	2.30E-05	0	0	7.20E-05	0	2.00E-05	0	0
0.004147	1.62E-04	0.001525	2.73E-04	1.02E-04	0	0	3.20E-05	7.23E-04	1.90E-05	0	4.66E-04	5.90E-05
4.51E-04	0.007197	0.001004	1.60E-04	0.00245	0	4.30E-05	0.002784	2.31E-04	0	0	1.47E-04	6.20E-05
7.70E-05	1.93E-04	9.28E-04	1.52E-04	3.00E-05	0	3.20E-05	1.57E-04	0	0	0	0	1.06E-04
0	0.026175	1.70E-05	0	9.18E-04	0	0	0.009057	0	0	0	0	0
0	0	4.40E-05	0	0	0	8.30E-05	0	0	1.80E-05	0	2.20E-05	1.43E-04
0	0	0	0	2.60E-05	0	0	0	0	0	0	2.30E-05	0
2.50E-05	0	0	0	0	0	0	0	0	0	0	0	0
0	0	0	3.20E-05	0	0	0	0	0	0	0	0	0
0	0	0	0	0	0	0	0	0	0	0	0	0

H110	H010	H043	H062	H131	H117	H104	H098	H122	H036	H071	H150	H103
0.259407	0.257722	0.253226	0.252984	0.252042	0.250993	0.249171	0.247769	0.244289	0.244157	0.243989	0.237685	0.235738
0.240821	0.359817	0.42748	0.214338	0.237632	0.299365	0.289925	0.323498	0.186859	0.470398	0.429933	0.319133	0.425456
0.266286	0.248821	0.139252	0.33082	0.156781	0.06976	0.326483	0.198648	0.451079	0.095981	0.084977	0.292551	0.100855
0.14075	0.050788	0.124544	0.089353	0.235287	0.294925	0.065052	0.103535	0.073924	0.136084	0.147451	0.058811	0.146932
0.06841	0.05569	0.03361	0.071458	0.065354	0.057031	0.03605	0.044058	0.023416	0.03405	0.020734	0.056538	0.035129
0.015032	0.018144	0.008588	0.029939	0.036909	0.010574	0.027242	0.062277	0.009643	0.013425	0.043359	0.028784	0.042001
0.007848	0.007806	0.010136	0.005784	0.010023	0.015971	0.002964	0.019176	0.008082	0.005488	0.027274	0.006083	0.005473
0.001072	2.01E-04	0.002429	0.00428	0.005414	0.001047	0.002651	9.72E-04	0.00262	2.42E-04	0.002097	2.00E-04	0.006339
0	0	0	0	0	0	0	0	0	5.20E-05	0	1.34E-04	7.00E-05
2.43E-04	0	3.76E-04	7.68E-04	4.64E-04	2.95E-04	2.61E-04	0	3.50E-05	5.70E-05	1.86E-04	4.80E-05	0.001367
4.90E-05	4.91E-04	9.10E-05	9.30E-05	1.80E-05	3.90E-05	7.20E-05	0	3.10E-05	6.60E-05	0	0	5.68E-04
5.60E-05	1.44E-04	2.68E-04	7.80E-05	3.60E-05	0	2.70E-05	6.70E-05	0	0	0	0	0
0	0	0	0	0	0	0	0	0	0	0	0	0
2.60E-05	2.91E-04	0	1.05E-04	1.60E-05	0	1.02E-04	0	2.20E-05	0	0	0	0
0	8.50E-05	0	0	0	0	0	0	0	0	0	0	0
0	0	0	0	2.40E-05	0	0	0	0	0	0	0	7.20E-05
0	0	0	0	0	0	0	0	0	0	0	3.30E-05	0
0	0	0	0	0	0	0	0	0	0	0	0	0

H101	H005	H143	H070	H021	H149	H095	H047	H088	H132	H044	H061	H092
0.235405	0.233691	0.232256	0.229192	0.228056	0.227664	0.227407	0.227075	0.227002	0.224196	0.224138	0.22375	0.22308
0.133677	0.279834	0.407551	0.160249	0.377446	0.115233	0.365525	0.309688	0.300383	0.182311	0.201654	0.286512	0.402828
0.50221	0.14836	0.123359	0.426412	0.128066	0.537353	0.164832	0.297897	0.322132	0.46504	0.255098	0.276294	0.15896
0.034575	0.232783	0.167659	0.131003	0.163081	0.071831	0.146579	0.113037	0.075947	0.046466	0.186554	0.187374	0.149432
0.078447	0.065573	0.021488	0.038133	0.050627	0.029715	0.026217	0.017709	0.047574	0.054126	0.115889	0.015313	0.013398
0.004079	0.015611	0.024834	0.005259	0.022167	0.005187	0.054741	0.026435	0.012637	0.01479	0.005822	0.005928	0.037262
0.006282	0.02308	0.003808	0.008794	0.019804	0.004916	0.012481	0.004859	0.005433	0.008972	0.00976	0.003471	0.008232
0.004248	9.02E-04	0.007333	7.38E-04	0.009312	0.005052	0.002048	0.001325	0.005792	0.003506	0.001003	5.73E-04	0.004738
0	3.20E-05	1.86E-04	2.20E-04	0	2.41E-04	0	0	1.21E-04	0	0	2.21E-04	0
9.83E-04	1.06E-04	0.008882	0	7.38E-04	0.001795	5.60E-05	0.001851	0.002484	3.64E-04	8.20E-05	4.38E-04	0.001513
7.40E-05	0	0.002351	0	4.99E-04	9.09E-04	2.10E-05	4.20E-05	4.95E-04	1.52E-04	0	1.26E-04	5.12E-04
2.00E-05	2.80E-05	0	0	0	3.40E-05	3.00E-05	3.90E-05	0	7.70E-05	0	0	0
0	0	0	0	0	0	0	0	0	0	0	0	0
0	0	0	0	0	0	6.30E-05	0	0	0	0	0	0
0	0	0	0	0	0	0	0	0	0	0	0	0
0	0	2.93E-04	0	0	7.00E-05	0	4.30E-05	0	0	0	0	4.50E-05
0	0	0	0	2.04E-04	0	0	0	0	0	0	0	0
0	0	0	0	0	0	0	0	0	0	0	0	0

H051	H108	H105	H065	H026	H113	H138	H025	H024	H127	H002	H096	H038
0.221357	0.219555	0.21866	0.217701	0.216853	0.216618	0.215404	0.215353	0.2141	0.212268	0.209637	0.208528	0.208163
0.368989	0.305294	0.3764	0.353437	0.504682	0.446428	0.366506	0.317271	0.223581	0.338555	0.289585	0.192233	0.479683
0.230205	0.340572	0.269351	0.290669	0.085732	0.195243	0.258295	0.260087	0.505341	0.225246	0.311842	0.418806	0.097417
0.11432	0.066381	0.076789	0.075715	0.081095	0.05705	0.075343	0.115329	0.022627	0.140715	0.068404	0.091442	0.126968
0.021848	0.025276	0.03723	0.038016	0.031973	0.035253	0.056063	0.017093	0.012286	0.032401	0.074762	0.037449	0.048492
0.023986	0.030352	0.006077	0.017157	0.037209	0.041476	0.020483	0.059665	0.012209	0.03408	0.024979	0.040622	0.030094
0.012141	0.010016	0.006921	0.004625	0.040304	0.006276	0.00736	0.008103	0.004942	0.015604	0.016384	0.00553	0.008378
0.00591	0.002273	0.006804	0.002456	6.32E-04	0.001229	3.18E-04	0.006185	3.70E-05	9.24E-04	0.003825	0.004763	6.48E-04
0	0	0	0	0	0	1.41E-04	0	0	0	0	0	3.10E-05
8.10E-04	1.18E-04	0.001214	1.00E-04	0	0	6.60E-05	2.12E-04	0	1.20E-04	4.80E-04	4.18E-04	1.26E-04
2.38E-04	0	1.74E-04	1.40E-05	0.001345	6.90E-05	2.10E-05	4.61E-04	0.004546	0	2.20E-05	4.80E-05	0
2.20E-05	7.10E-05	2.90E-04	0	8.30E-05	3.27E-04	0	6.00E-05	4.40E-05	8.70E-05	5.60E-05	3.20E-05	0
0	0	0	0	1.90E-05	0	0	4.30E-05	2.87E-04	0	0	0	0
1.70E-05	0	6.50E-05	1.10E-04	7.30E-05	3.10E-05	0	1.38E-04	0	0	0	0	0
0	0	2.50E-05	0	0	0	0	0	0	0	2.40E-05	0	0
0	9.20E-05	0	0	0	0	0	0	0	0	0	1.29E-04	0
1.57E-04	0	0	0	0	0	0	0	0	0	0	0	0
0	0	0	0	0	0	0	0	0	0	0	0	0

H084	H060	H040	H094	H014	H035	H078	H015	H109	H090	H057	H111	H099
0.206818	0.199169	0.198584	0.197276	0.196894	0.195689	0.195041	0.194038	0.188923	0.187473	0.18434	0.184325	0.181717
0.166955	0.500047	0.394044	0.404729	0.311657	0.457496	0.417197	0.313544	0.313516	0.328087	0.272819	0.457614	0.442603
0.518987	0.144623	0.199554	0.19223	0.190607	0.168399	0.133218	0.254596	0.350375	0.294129	0.320528	0.241468	0.21858
0.073309	0.068718	0.11157	0.108712	0.190616	0.122693	0.135832	0.175734	0.088201	0.11032	0.099064	0.062329	0.069591
0.017179	0.05407	0.049603	0.044956	0.034449	0.016763	0.022896	0.02166	0.030508	0.029825	0.055702	0.023845	0.027344
0.006217	0.007225	0.037925	0.040256	0.045402	0.029743	0.08294	0.028327	0.025076	0.031468	0.033893	0.026148	0.027858
0.004016	0.016549	0.007409	0.011032	0.030296	0.00514	0.010084	0.011861	0.002689	0.01842	0.033236	0.001831	0.030779
0.00445	0.006695	8.90E-04	5.92E-04	3.90E-05	0.003406	0.002383	1.94E-04	5.03E-04	1.91E-04	3.91E-04	0.002213	9.58E-04
0	0	0	1.00E-04	4.00E-05	8.70E-05	1.18E-04	1.50E-05	7.30E-05	3.90E-05	0	0	0
0.001126	7.39E-04	3.93E-04	1.17E-04	0	5.49E-04	2.91E-04	0	1.08E-04	4.80E-05	0	1.55E-04	9.80E-05
9.43E-04	0.00108	2.80E-05	0	0	3.50E-05	0	0	0	0	0	7.20E-05	0
0	1.08E-04	0	0	0	0	0	3.10E-05	2.80E-05	0	0	0	4.11E-04
0	0	0	0	0	0	0	0	0	0	0	0	0
0	4.60E-04	0	0	0	0	0	0	0	0	2.70E-05	0	6.10E-05
0	0	0	0	0	0	0	0	0	0	0	0	0
0	5.70E-05	0	0	0	0	0	0	0	0	0	0	0
0	0	0	0	0	0	0	0	0	0	0	0	0
0	4.60E-04	0	0	0	0	0	0	0	0	0	0	0

H118	H009	H093	H114	H081	H018	H120	H106	H023	H076	H083	H072	H027
0.180066	0.174967	0.174481	0.171305	0.170141	0.169747	0.168849	0.168739	0.168511	0.168471	0.168041	0.166143	0.165561
0.432748	0.391925	0.356857	0.386043	0.409268	0.394644	0.287926	0.440686	0.368819	0.303324	0.295907	0.328127	0.297021
0.215612	0.140801	0.32749	0.234813	0.253368	0.251006	0.430223	0.201139	0.312841	0.106885	0.382932	0.198538	0.418729
0.106572	0.219141	0.096557	0.101296	0.110095	0.115921	0.036386	0.114041	0.095467	0.340088	0.103524	0.248141	0.029012
0.018754	0.018669	0.018751	0.081309	0.046697	0.024913	0.061181	0.024297	0.025322	0.023017	0.026637	0.012977	0.034013
0.039385	0.034792	0.020502	0.013121	0.005159	0.036986	0.002396	0.023071	0.018383	0.032281	0.016291	0.028943	0.041539
0.005327	0.018928	0.004619	0.011828	0.004677	0.005479	0.005181	0.017906	0.008928	0.025082	0.00422	0.014704	0.006771
0.001302	4.30E-04	4.18E-04	2.27E-04	3.59E-04	0.001045	0.006355	0.009038	0.001527	8.01E-04	0.001808	0.002171	9.80E-05
0	0	2.58E-04	0	0	0	0	0	0	0	8.50E-05	0	0
1.05E-04	0	3.70E-05	4.70E-05	0	3.70E-05	0.001382	4.80E-04	2.02E-04	5.10E-05	4.68E-04	1.40E-04	0
4.30E-05	8.10E-05	3.00E-05	1.10E-05	3.70E-05	4.10E-05	1.21E-04	3.17E-04	0	0	8.70E-05	1.16E-04	0.00599
0	5.20E-05	0	0	1.61E-04	7.10E-05	0	0	0	0	0	0	6.24E-04
0	0	0	0	0	0	0	0	0	0	0	0	5.14E-04
8.60E-05	2.14E-04	0	0	3.80E-05	7.10E-05	0	1.60E-05	0	0	0	0	1.28E-04
0	0	0	0	0	3.90E-05	0	0	0	0	0	0	0
0	0	0	0	0	0	0	1.50E-05	0	0	0	0	0
0	0	0	0	0	0	0	2.55E-04	0	0	0	0	0
0	0	0	0	0	0	0	0	0	0	0	0	0

H020	H086	H049	H064	H033	H013	H112	H075	H089	H067	H082	H046	H063
0.164621	0.164073	0.163408	0.162027	0.159756	0.15591	0.152914	0.148174	0.142648	0.141324	0.139871	0.134099	0.131764
0.277265	0.331664	0.279564	0.235088	0.313848	0.330752	0.490049	0.192271	0.298045	0.26311	0.337168	0.320539	0.148999
0.31657	0.325461	0.336361	0.429469	0.401793	0.357491	0.156273	0.565776	0.178128	0.38252	0.246177	0.380428	0.570739
0.089879	0.102761	0.117809	0.143336	0.068225	0.125776	0.097798	0.04212	0.302676	0.154953	0.190783	0.116348	0.054591
0.089592	0.025702	0.030207	0.019255	0.017428	0.016163	0.047069	0.021019	0.028778	0.03704	0.045728	0.016286	0.06789
0.04854	0.030699	0.032009	0.001635	0.017725	0.008554	0.035142	0.021428	0.035032	0.013478	0.028672	0.028664	0.017611
0.011239	0.013587	0.017742	0.00743	0.014743	0.00455	0.019152	0.003033	0.012892	0.005937	0.010721	0.002058	0.006046
0.00199	0.004177	0.014924	0.001463	0.001231	6.83E-04	0.001331	0.005918	0.001444	0.001077	7.66E-04	0.001236	0.001855
0	0	0	6.80E-05	0	3.60E-05	0	0	0	4.20E-05	0	0	0
8.30E-05	0.001664	0.006672	1.22E-04	2.16E-04	8.50E-05	2.16E-04	2.10E-04	2.77E-04	3.71E-04	9.00E-05	2.95E-04	2.50E-04
1.26E-04	1.90E-04	6.94E-04	0	0.004114	0	2.20E-05	1.90E-05	8.00E-05	1.48E-04	2.40E-05	4.70E-05	0
5.80E-05	0	5.63E-04	7.90E-05	7.65E-04	0	0	3.20E-05	0	0	0	0	2.55E-04
0	0	0	0	1.56E-04	0	0	0	0	0	0	0	0
1.50E-05	0	0	0	0	0	3.40E-05	0	0	0	0	0	0
0	0	0	0	0	0	0	0	0	0	0	0	0
0	2.20E-05	4.70E-05	2.80E-05	0	0	0	0	0	0	0	0	0
2.20E-05	0	0	0	0	0	0	0	0	0	0	0	0
0	0	0	0	0	0	0	0	0	0	0	0	0

H137	H147	H097	H102	H123	H048	H052	H121	H068	H006	H017	H069	H146
0.128927	0.12467	0.12463	0.119205	0.110677	0.108017	0.103826	0.10133	0.101277	0.097792	0.096519	0.0944	0.094322
0.261211	0.238073	0.265745	0.299216	0.240725	0.215718	0.277502	0.227624	0.201127	0.213087	0.160059	0.235318	0.459739
0.422182	0.428378	0.429916	0.364338	0.476858	0.518465	0.541767	0.529852	0.597827	0.565796	0.637032	0.485205	0.129856
0.148625	0.150179	0.102718	0.160192	0.083638	0.045089	0.01804	0.090032	0.074136	0.05382	0.06548	0.111949	0.195881
0.016083	0.030035	0.033783	0.014051	0.045827	0.099075	0.016133	0.015321	0.008606	0.033445	0.02379	0.037748	0.044743
0.014931	0.018967	0.035157	0.020449	0.030104	0.006888	0.032184	0.028855	0.010036	0.01875	0.01067	0.023365	0.066904
0.006231	0.007493	0.007182	0.020206	0.009862	0.002461	0.009172	0.004005	0.004888	0.008856	0.001947	0.011024	0.008483
0.001468	0.001813	5.32E-04	0.002257	0.001926	0.002267	0.00129	0.002352	0.001605	0.00714	0.003968	7.27E-04	0
0	1.72E-04	2.13E-04	5.40E-05	0	6.48E-04	0	2.00E-05	0	0	2.80E-05	3.70E-05	0
3.09E-04	2.20E-04	1.24E-04	3.20E-05	2.98E-04	0.001099	0	4.54E-04	4.47E-04	0.001041	3.84E-04	1.80E-04	0
3.30E-05	0	0	0	0	2.73E-04	4.30E-05	1.33E-04	0	6.20E-05	1.23E-04	4.70E-05	3.10E-05
0	0	0	0	2.30E-05	0	1.40E-05	0	0	1.21E-04	0	0	0
0	0	0	0	0	0	0	0	0	0	0	0	0
0	0	0	0	4.60E-05	0	2.90E-05	2.20E-05	2.30E-05	5.70E-05	0	0	4.10E-05
0	0	0	0	0	0	0	0	0	0	0	0	0
0	0	0	0	0	0	0	0	2.80E-05	3.30E-05	0	0	0
0	0	0	0	1.60E-05	0	0	0	0	0	0	0	0
0	0	0	0	0	0	0	0	0	0	0	0	0

H077	H074	H087	H004
0.08319	0.073136	0.069366	0.049219
0.245328	0.322669	0.253435	0.220714
0.535264	0.38164	0.540408	0.582023
0.10091	0.177868	0.066348	0.078461
0.01913	0.010237	0.021856	0.031781
0.009936	0.023028	0.041019	0.033072
0.005514	0.009467	0.006765	0.003876
5.06E-04	0.001766	5.42E-04	7.44E-04
1.20E-04	0	0	0
5.30E-05	1.89E-04	1.75E-04	8.10E-05
4.90E-05	0	6.30E-05	0
0	0	2.30E-05	0
0	0	0	0
0	0	0	0
0	0	0	0
0	0	0	0
0	0	0	2.90E-05
0	0	0	0

Supplementary table S18. The different degree of microbiome at phylum level (p value)							
ID	CC.median	CC.mean	CC.se	CCR.median	CCR.mean	CCR.se	H.median
Firmicutes	0.3515(0.1753,0.5063)	0.365673918	0.02756398	0.3779(0.2693,0.575)	0.426132524	0.04681201	0.2234(0.1687,0.2822)
Bacteroidota	0.143(0.072,0.2902)	0.210188726	0.02414691	0.0755(0.0438,0.1024)	0.121292905	0.040224509	0.3231(0.2626,0.4007)
Proteobacteria	0.0752(0.0092,0.2685)	0.172200027	0.02557727	0.2177(0.1333,0.5226)	0.31174619	0.046500481	0.2277(0.1322,0.343)
Actinobacteriota	0.0513(0.023,0.1314)	0.095685616	0.01345615	0.0123(0.0088,0.0631)	0.048632857	0.019084152	0.0373(0.0242,0.0569)
Patescibacteria	0.0018(0.0004,0.0076)	0.007782712	0.00189238	0.0025(0.0005,0.0043)	0.002588857	4.45E-04	0.023(0.0124,0.0333)
Campilobacterota	0.0012(0.0005,0.0055)	0.005457247	0.00136071	0.0031(0.0015,0.0033)	0.00296181	4.56E-04	0.0083(0.0052,0.0128)
Spirochaetota	<0.0001(<0.0001,0.0004)	9.27E-04	2.93E-04	0.0002(<0.0001,0.002)	0.001292	3.88E-04	0.0014(0.0006,0.0038)
Bacteria_unclassified	0.0003(<0.0001,0.0008)	9.71E-04	2.48E-04	<0.0001(<0.0001,0.0006)	4.32E-04	1.37E-04	<0.0001(<0.0001,<0.0001)
Synergistota	<0.0001(<0.0001,<0.0001)	1.22E-04	5.68E-05	<0.0001(<0.0001,0.0001)	8.94E-05	3.31E-05	0.0002(<0.0001,0.0005)
Desulfobacterota	<0.0001(<0.0001,<0.0001)	1.72E-05	7.42E-06	<0.0001(<0.0001,<0.0001)	1.10E-05	7.15E-06	<0.0001(<0.0001,0.0002)
Cyanobacteria	<0.0001(<0.0001,<0.0001)	4.52E-04	2.07E-04	<0.0001(<0.0001,<0.0001)	1.57E-05	9.99E-06	<0.0001(<0.0001,<0.0001)
Deferribacterota	<0.0001(<0.0001,<0.0001)	0	0	<0.0001(<0.0001,<0.0001)	0	0	<0.0001(<0.0001,<0.0001)
Verrucomicrobiota	<0.0001(<0.0001,<0.0001)	5.79E-06	3.74E-06	<0.0001(<0.0001,<0.0001)	0	0	<0.0001(<0.0001,<0.0001)
Chloroflexi	<0.0001(<0.0001,<0.0001)	1.27E-06	6.05E-07	<0.0001(<0.0001,<0.0001)	1.38E-06	1.38E-06	<0.0001(<0.0001,<0.0001)

H.mean	H.se	p-value	z-score	Sig_mark	q-value	fixp	fixps
0.229006643	0.007306092	3.34E-07	-5.103127961	***	7.52E-07	<0.0001	***<0.0001
0.32872055	0.007756981	2.75E-16	-8.183831587	***	1.65E-15	<0.0001	***<0.0001
0.252985871	0.013133376	6.72E-06	-4.502221421	***	1.21E-05	<0.0001	***<0.0001
0.044443079	0.00226418	0.002139944	-3.070089809	**	0.003501727	0.0021	** 0.0021
0.024120907	0.001340821	1.26E-22	-9.788758034	***	2.26E-21	<0.0001	***<0.0001
0.010312629	6.46E-04	5.89E-16	-8.091625261	***	2.65E-15	<0.0001	***<0.0001
0.002847714	3.61E-04	2.39E-14	-7.627801931	***	8.60E-14	<0.0001	***<0.0001
3.72E-05	6.84E-06	1.61E-18	-8.781969435	***	1.45E-17	<0.0001	***<0.0001
5.25E-04	9.56E-05	3.25E-12	-6.966398291	***	8.36E-12	<0.0001	***<0.0001
4.08E-04	8.87E-05	6.18E-14	-7.504246404	***	1.85E-13	<0.0001	***<0.0001
8.47E-05	1.55E-05	0.048473394	-1.973194611	*	0.063198454	0.0485	* 0.0485
2.76E-04	1.97E-04	0.010089265	-2.57275483	*	0.015133898	0.0101	* 0.0101
6.93E-05	1.94E-05	8.04E-07	-4.934317601	***	1.61E-06	<0.0001	***<0.0001
9.90E-06	2.78E-06	0.049154353	-1.967250374	*	0.063198454	0.0492	* 0.0492

Supplementary table S18. The different degree of microbiome at genus level (p value)							
ID	CC.median	CC.mean	CC.se	CCR.median	CCR.mean	CCR.se	H.median
Prevotella	0.0915(0.0271,0.2518)	0.170206603	0.024530362	0.037(0.0154,0.0568)	0.077687143	0.040876129	0.168(0.1013,0.2298)
Neisseria	0.0286(0.0016,0.1622)	0.133397356	0.024161565	0.0852(0.0275,0.2505)	0.159918571	0.038031666	0.1318(0.065,0.2575)
Streptococcus	0.062(0.0243,0.2253)	0.160325562	0.0233208	0.1887(0.0929,0.3843)	0.251226238	0.046170581	0.0504(0.0328,0.0829)
Leptotrichia	0.0531(0.0191,0.1399)	0.109328507	0.017554713	0.0305(0.0113,0.0456)	0.04148281	0.010511254	0.0259(0.0142,0.0502)
Fusobacterium	0.0074(0.0007,0.0301)	0.031138	0.008728899	0.0382(0.0169,0.0583)	0.043302667	0.007263825	0.0585(0.0364,0.0916)
Haemophilus	0.0027(0.0003,0.0134)	0.014064164	0.003301992	0.0459(0.011,0.084)	0.087566762	0.028011533	0.0393(0.0204,0.0658)
Alloprevotella	0.0008(0.0003,0.0019)	0.002536644	6.21E-04	0.0029(0.0012,0.0071)	0.007155143	0.002565415	0.056(0.0379,0.0871)
Porphyromonas	0.0032(0.0007,0.0182)	0.022523726	0.006893269	0.0151(0.0044,0.0221)	0.018677333	0.004262576	0.0451(0.0202,0.0699)
Actinomyces	0.0162(0.0047,0.0777)	0.056781384	0.010890556	0.0031(0.0001,0.0149)	0.01098981	0.003589627	0.0169(0.0101,0.0269)
Gemella	0.0029(0.0002,0.0298)	0.028027603	0.007647034	0.0213(0.0074,0.059)	0.055719429	0.019967427	0.0047(0.002,0.0081)
Capnocytophaga	0.0033(0.0004,0.0133)	0.011647562	0.002119185	0.0098(0.0057,0.0183)	0.01365181	0.002696136	0.0112(0.0044,0.0205)
Lautropia	0.0002(<0.0001,0.0027)	0.006691342	0.002782466	0.028(0.005,0.1059)	0.056190857	0.014307819	0.0014(0.0004,0.003)
Granulicatella	0.0032(0.0007,0.0116)	0.01078137	0.002441113	0.0124(0.0063,0.0188)	0.015977	0.003188544	0.0057(0.0037,0.009)
Lachnoanaerobacu	0.0034(0.0013,0.0091)	0.00842689	0.002056468	0.003(0.0014,0.0064)	0.005008143	0.001461537	0.0069(0.0043,0.0122)
TM7x	0.001(0.0001,0.0037)	0.005583836	0.001534326	0.0009(0.0001,0.002)	0.001207476	2.63E-04	0.0075(0.0034,0.0143)
Campylobacter	0.0012(0.0005,0.0055)	0.005456068	0.001360701	0.0031(0.0015,0.0033)	0.002960762	4.56E-04	0.0081(0.0051,0.0123)
Moraxella	<0.0001(<0.0001,<0.0001)	1.63E-06	1.02E-06	<0.0001(<0.0001,<0.0001)	0	0	<0.0001(<0.0001,<0.0001)
Actinobacillus	<0.0001(<0.0001,<0.0001)	0.001007603	6.33E-04	<0.0001(<0.0001,<0.0001)	0.001743619	0.001509281	0.0014(0.0002,0.005)
g__Absconditabac	<0.0001(<0.0001,0.0002)	4.42E-04	1.50E-04	<0.0001(<0.0001,0.0005)	3.39E-04	1.74E-04	0.0046(0.0007,0.0114)
Oribacterium	0.0014(0.0003,0.0046)	0.00374463	7.40E-04	0.0019(0.0004,0.0038)	0.002795952	7.21E-04	0.0041(0.0027,0.0059)
Bacteroides	<0.0001(<0.0001,<0.0001)	6.63E-04	4.73E-04	<0.0001(<0.0001,0.0001)	1.13E-04	4.45E-05	0.0005(0.0001,0.0023)
Selenomonas	0.0017(0.0003,0.0044)	0.003793164	7.05E-04	0.0004(0.0001,0.0019)	0.001891619	6.54E-04	0.0019(0.0006,0.0048)
Stomatobaculum	0.0009(0.0001,0.0025)	0.003882959	9.82E-04	0.0007(<0.0001,0.0014)	0.001262095	3.31E-04	0.0023(0.0009,0.0052)
Megasphaera	0.0004(<0.0001,0.0026)	0.002249096	4.52E-04	0.0002(<0.0001,0.0014)	0.001068905	3.59E-04	0.0014(0.0002,0.006)
Pseudomonas	<0.0001(<0.0001,<0.0001)	1.19E-04	4.33E-05	<0.0001(<0.0001,0.0001)	3.58E-04	1.86E-04	0.0014(0.0005,0.004)
Solobacterium	0.0009(0.0002,0.0025)	0.002176986	4.62E-04	0.0006(0.0002,0.0015)	0.001129905	3.31E-04	0.0029(0.002,0.0045)
Peptostreptococcu	0.0014(0.0001,0.0043)	0.003478781	8.38E-04	0.0007(0.0002,0.0015)	0.001252	3.12E-04	0.0026(0.0015,0.0048)
Halomonas	0.0002(<0.0001,0.0042)	0.008948795	0.002978576	<0.0001(<0.0001,<0.0001)	1.29E-06	1.29E-06	<0.0001(<0.0001,0.0002)
Clostridia_UCG-0	0.0003(<0.0001,0.0014)	0.001853904	6.61E-04	0.0001(<0.0001,0.0007)	5.90E-04	2.33E-04	0.003(0.0015,0.0049)
Lachnospiraceae_	<0.0001(<0.0001,0.0007)	0.001219411	3.79E-04	<0.0001(<0.0001,<0.0001)	3.93E-04	2.60E-04	0.0012(0.0002,0.005)
Aggregatibacter	<0.0001(<0.0001,<0.0001)	3.85E-04	1.28E-04	<0.0001(<0.0001,0.001)	0.001115143	5.53E-04	0.003(0.0009,0.006)
Muribaculaceae	<0.0001(<0.0001,<0.0001)	5.41E-06	3.60E-06	<0.0001(<0.0001,<0.0001)	0	0	<0.0001(<0.0001,0.0002)
Corynebacterium	<0.0001(<0.0001,0.0003)	0.001918904	9.47E-04	0.0003(<0.0001,0.0016)	0.001773619	6.34E-04	0.0008(0.0004,0.0021)
Atopobium	0.0005(0.0002,0.002)	0.002448027	6.78E-04	0.0001(<0.0001,0.0003)	4.14E-04	1.73E-04	0.0015(0.0008,0.0031)
Faecalibacterium	<0.0001(<0.0001,<0.0001)	2.69E-04	1.97E-04	<0.0001(<0.0001,<0.0001)	4.80E-05	1.35E-05	0.0002(<0.0001,0.001)
[Eubacterium]_no	0.0009(0.0003,0.0023)	0.002049425	3.64E-04	0.0005(0.0001,0.0006)	0.001018	4.62E-04	0.002(0.0013,0.0032)
Tannerella	0.0002(<0.0001,0.0015)	0.001451945	3.76E-04	0.0003(<0.0001,0.0008)	0.001828	0.00142981	0.0015(0.0007,0.0031)
Treponema	<0.0001(<0.0001,0.0004)	8.99E-04	2.88E-04	0.0002(<0.0001,0.002)	0.001288571	3.88E-04	0.0014(0.0006,0.0038)
Lachnospiraceae_	<0.0001(<0.0001,0.0002)	1.67E-04	5.28E-05	<0.0001(<0.0001,<0.0001)	1.50E-04	7.21E-05	0.0002(<0.0001,0.0005)
Saccharimonadale	<0.0001(<0.0001,<0.0001)	3.37E-04	1.02E-04	<0.0001(<0.0001,0.0001)	8.04E-05	2.60E-05	0.0011(0.0003,0.0032)
Abiotrophia	<0.0001(<0.0001,0.0006)	0.002949863	0.001089008	0.0004(<0.0001,0.0021)	0.004243381	0.001837101	<0.0001(<0.0001,0.0002)
Parvimonas	<0.0001(<0.0001,0.0001)	2.81E-04	9.20E-05	0.0002(<0.0001,0.0011)	0.001252429	7.02E-04	0.0013(0.0005,0.0027)
Rhodococcus	<0.0001(<0.0001,<0.0001)	5.55E-05	1.88E-05	<0.0001(<0.0001,<0.0001)	1.62E-05	8.42E-06	<0.0001(<0.0001,0.0004)
Bergeyella	<0.0001(<0.0001,0.0004)	4.51E-04	9.86E-05	0.0011(0.0006,0.0019)	0.001391286	1.98E-04	0.0011(0.0005,0.002)
F0332	<0.0001(<0.0001,0.0001)	0.00142474	8.05E-04	<0.0001(<0.0001,0.0001)	4.02E-04	2.41E-04	0.0002(<0.0001,0.0006)
Johnsonella	<0.0001(<0.0001,0.0005)	9.55E-04	3.45E-04	0.0002(<0.0001,0.001)	6.53E-04	1.93E-04	0.0005(0.0001,0.0017)
Filifactor	<0.0001(<0.0001,0.0001)	2.14E-04	5.86E-05	<0.0001(<0.0001,0.0002)	1.74E-04	6.83E-05	0.0009(0.0002,0.0019)
Lachnospiraceae_	<0.0001(<0.0001,<0.0001)	3.36E-05	2.45E-05	<0.0001(<0.0001,<0.0001)	3.33E-06	2.31E-06	<0.0001(<0.0001,0.0003)
Fusicatenibacter	<0.0001(<0.0001,<0.0001)	3.42E-05	2.83E-05	<0.0001(<0.0001,<0.0001)	7.57E-06	3.53E-06	<0.0001(<0.0001,0.0005)
Dialister	<0.0001(<0.0001,0.0002)	5.88E-04	2.05E-04	0.0002(<0.0001,0.0006)	9.91E-04	4.29E-04	0.0006(0.0002,0.0015)
Saccharimonadace	<0.0001(<0.0001,0.0002)	5.99E-04	1.97E-04	0.0003(<0.0001,0.0008)	6.51E-04	2.30E-04	0.0003(<0.0001,0.0006)
Acinetobacter	<0.0001(<0.0001,<0.0001)	2.78E-05	1.06E-05	<0.0001(<0.0001,0.0001)	1.78E-04	8.12E-05	0.0001(<0.0001,0.0005)
Sphingomonas	<0.0001(<0.0001,<0.0001)	1.94E-05	9.12E-06	<0.0001(<0.0001,<0.0001)	2.38E-06	1.69E-06	<0.0001(<0.0001,0.0004)
F0058	<0.0001(<0.0001,0.0001)	4.06E-04	1.55E-04	<0.0001(<0.0001,0.0002)	6.23E-04	3.03E-04	0.0003(<0.0001,0.0006)
Kingella	<0.0001(<0.0001,<0.0001)	0.001456685	9.51E-04	<0.0001(<0.0001,0.0008)	4.28E-04	1.52E-04	<0.0001(<0.0001,0.0002)
Bifidobacterium	<0.0001(<0.0001,<0.0001)	1.87E-04	1.16E-04	<0.0001(<0.0001,<0.0001)	2.13E-05	7.69E-06	<0.0001(<0.0001,0.0003)

Lactobacillus	<0.0001(<0.0001,<0.0001)	1.62E-04	8.26E-05	<0.0001(<0.0001,<0.0001)	1.32E-05	5.03E-06	<0.0001(<0.0001,0.0001)
Roseburia	<0.0001(<0.0001,<0.0001)	1.02E-04	7.44E-05	<0.0001(<0.0001,<0.0001)	1.36E-05	5.60E-06	0.0002(<0.0001,0.0005)
Leptotrichiaceae_	<0.0001(<0.0001,<0.0001)	1.34E-06	1.34E-06	<0.0001(<0.0001,<0.0001)	4.43E-06	4.43E-06	<0.0001(<0.0001,<0.0001)
Megamonas	<0.0001(<0.0001,<0.0001)	3.13E-05	2.33E-05	<0.0001(<0.0001,<0.0001)	1.12E-05	6.76E-06	<0.0001(<0.0001,0.0002)
Actinobacteria_un	0.0004(<0.0001,0.0014)	0.001271986	2.71E-04	<0.0001(<0.0001,<0.0001)	1.23E-04	6.19E-05	<0.0001(<0.0001,<0.0001)
[Eubacterium]_ru	<0.0001(<0.0001,<0.0001)	5.05E-06	3.64E-06	<0.0001(<0.0001,<0.0001)	8.67E-06	4.00E-06	<0.0001(<0.0001,0.0002)
Bacteria_unclassi	0.0003(<0.0001,0.0008)	9.71E-04	2.48E-04	<0.0001(<0.0001,0.0006)	4.32E-04	1.37E-04	<0.0001(<0.0001,<0.0001)
Burkholderiales_u	<0.0001(<0.0001,0.0006)	9.14E-04	2.43E-04	0.0002(<0.0001,0.0006)	3.30E-04	9.77E-05	<0.0001(<0.0001,<0.0001)
Selenomonadacea	<0.0001(<0.0001,<0.0001)	7.96E-06	3.81E-06	<0.0001(<0.0001,<0.0001)	0	0	<0.0001(<0.0001,0.0001)
Christensenellace	<0.0001(<0.0001,<0.0001)	2.31E-05	1.24E-05	<0.0001(<0.0001,<0.0001)	9.52E-07	9.52E-07	<0.0001(<0.0001,0.0002)
Gracilibacteria	<0.0001(<0.0001,<0.0001)	1.12E-05	5.75E-06	<0.0001(<0.0001,<0.0001)	3.30E-05	3.04E-05	<0.0001(<0.0001,0.0002)
Helicobacter	<0.0001(<0.0001,<0.0001)	2.74E-07	2.74E-07	<0.0001(<0.0001,<0.0001)	0	0	<0.0001(<0.0001,<0.0001)
Prevotellaceae_N	<0.0001(<0.0001,<0.0001)	3.97E-07	3.97E-07	<0.0001(<0.0001,<0.0001)	0	0	<0.0001(<0.0001,<0.0001)
Acholeplasma	<0.0001(<0.0001,<0.0001)	1.89E-06	1.44E-06	<0.0001(<0.0001,<0.0001)	0	0	<0.0001(<0.0001,<0.0001)
Phascolarctobacte	<0.0001(<0.0001,<0.0001)	2.85E-05	1.56E-05	<0.0001(<0.0001,<0.0001)	2.29E-06	1.28E-06	<0.0001(<0.0001,0.0001)
Pelagibacterium	<0.0001(<0.0001,0.0004)	5.96E-04	2.14E-04	<0.0001(<0.0001,<0.0001)	0	0	<0.0001(<0.0001,<0.0001)
Serratia	<0.0001(<0.0001,<0.0001)	1.58E-05	4.41E-06	<0.0001(<0.0001,<0.0001)	2.38E-06	1.65E-06	<0.0001(<0.0001,0.0001)
Mucispirillum	<0.0001(<0.0001,<0.0001)	0	0	<0.0001(<0.0001,<0.0001)	0	0	<0.0001(<0.0001,<0.0001)
Chloroplast	<0.0001(<0.0001,<0.0001)	4.52E-04	2.07E-04	<0.0001(<0.0001,<0.0001)	1.57E-05	9.99E-06	<0.0001(<0.0001,<0.0001)
Propionibacterium	<0.0001(<0.0001,<0.0001)	8.42E-06	7.15E-06	<0.0001(<0.0001,<0.0001)	0	0	<0.0001(<0.0001,<0.0001)

H.mean	H.se	p-value	z-score	Sig_mark	q-value	fixp	fixps
0.175047486	0.008138233	1.51E-08	-5.660805023	***	5.68E-08	<0.0001	***<0.0001
0.1644046	0.010652339	3.25E-05	-4.154902094	***	7.61E-05	<0.0001	***<0.0001
0.066261386	0.004203388	2.02E-05	-4.26240694	***	4.83E-05	<0.0001	***<0.0001
0.039052136	0.003438658	0.00227463	-3.051814421	**	0.004213653	0.0023	** 0.0023
0.066101664	0.003729391	1.39E-14	-7.697287499	***	1.21E-13	<0.0001	***<0.0001
0.049955393	0.003719139	7.98E-17	-8.331544895	***	1.04E-15	<0.0001	***<0.0001
0.06696755	0.00370023	2.16E-35	-12.4153966	***	7.31E-33	<0.0001	***<0.0001
0.049835936	0.003239805	1.20E-15	-8.004612111	***	1.35E-14	<0.0001	***<0.0001
0.020140629	0.001224668	3.95E-04	-3.543215154	***	8.21E-04	0.0004	***0.0004
0.007034657	7.39E-04	0.0032375	-2.944238584	**	0.005964748	0.0032	** 0.0032
0.017839043	0.001895537	1.26E-04	-3.833703814	***	2.80E-04	0.0001	***0.0001
0.003487557	5.64E-04	1.96E-10	-6.364337567	***	1.01E-09	<0.0001	***<0.0001
0.00727395	4.80E-04	2.94E-04	-3.620735836	***	6.26E-04	0.0003	***0.0003
0.008890107	5.18E-04	2.26E-06	-4.728791998	***	6.22E-06	<0.0001	***<0.0001
0.010159507	8.26E-04	6.36E-16	-8.082251274	***	7.43E-15	<0.0001	***<0.0001
0.009792136	5.74E-04	1.88E-15	-7.949026242	***	2.06E-14	<0.0001	***<0.0001
0.0097705	0.003717535	4.68E-08	-5.46290267	***	1.64E-07	<0.0001	***<0.0001
0.008773979	0.00222949	7.04E-21	-9.373153903	***	2.65E-19	<0.0001	***<0.0001
0.007893879	7.86E-04	1.13E-25	-10.47442772	***	9.60E-24	<0.0001	***<0.0001
0.004582179	2.50E-04	2.63E-07	-5.148219861	***	8.41E-07	<0.0001	***<0.0001
0.006351971	0.001817812	3.81E-20	-9.193169779	***	1.02E-18	<0.0001	***<0.0001
0.004129014	5.42E-04	0.01426303	-2.450569839	*	0.023024604	0.0143	* 0.0143
0.004052121	4.49E-04	6.34E-06	-4.514825276	***	1.68E-05	<0.0001	***<0.0001
0.004736579	6.52E-04	0.00122717	-3.232488203	**	0.002350346	0.0012	** 0.0012
0.005208914	8.97E-04	7.11E-29	-11.15068693	***	1.20E-26	<0.0001	***<0.0001
0.003876521	2.86E-04	2.69E-13	-7.309195276	***	2.02E-12	<0.0001	***<0.0001
0.00317665	1.83E-04	2.02E-05	-4.262437992	***	4.83E-05	<0.0001	***<0.0001
2.28E-04	4.29E-05	1.56E-10	-6.399436917	***	8.13E-10	<0.0001	***<0.0001
0.003732014	2.59E-04	4.61E-18	-8.662534176	***	7.45E-17	<0.0001	***<0.0001
0.003938564	6.02E-04	8.26E-12	-6.833971459	***	5.09E-11	<0.0001	***<0.0001
0.004090543	3.46E-04	8.46E-26	-10.5019907	***	9.56E-24	<0.0001	***<0.0001
0.004324393	0.001373141	9.08E-18	-8.585018589	***	1.40E-16	<0.0001	***<0.0001
0.0027329	5.84E-04	8.12E-12	-6.836378233	***	5.09E-11	<0.0001	***<0.0001
0.002589129	2.88E-04	8.89E-09	-5.750626896	***	3.46E-08	<0.0001	***<0.0001
0.00373735	0.001093432	3.07E-15	-7.887915183	***	3.16E-14	<0.0001	***<0.0001
0.002449621	1.50E-04	1.56E-08	-5.65507608	***	5.80E-08	<0.0001	***<0.0001
0.002509186	2.91E-04	2.06E-10	-6.357020463	***	1.03E-09	<0.0001	***<0.0001
0.002843079	3.60E-04	1.08E-14	-7.729983536	***	1.01E-13	<0.0001	***<0.0001
0.002861486	6.93E-04	8.84E-09	-5.751552031	***	3.46E-08	<0.0001	***<0.0001
0.002393479	3.07E-04	6.45E-20	-9.136428484	***	1.55E-18	<0.0001	***<0.0001
3.77E-04	9.34E-05	0.02261727	-2.279838431	*	0.033959204	0.0226	* 0.0226
0.002112964	2.04E-04	3.92E-20	-9.190156553	***	1.02E-18	<0.0001	***<0.0001
0.002161336	7.93E-04	6.09E-10	-6.188008238	***	2.91E-09	<0.0001	***<0.0001
0.001701693	1.93E-04	7.51E-14	-7.478598287	***	5.92E-13	<0.0001	***<0.0001
0.001235293	3.46E-04	1.75E-07	-5.224534288	***	5.92E-07	<0.0001	***<0.0001
0.001324986	1.63E-04	1.39E-06	-4.82645244	***	3.93E-06	<0.0001	***<0.0001
0.001667936	2.15E-04	1.64E-17	-8.516878184	***	2.32E-16	<0.0001	***<0.0001
0.001769564	4.42E-04	1.30E-14	-7.705661169	***	1.16E-13	<0.0001	***<0.0001
0.001727229	5.09E-04	2.17E-18	-8.747891663	***	3.88E-17	<0.0001	***<0.0001
0.001271543	1.85E-04	7.08E-12	-6.85610981	***	4.61E-11	<0.0001	***<0.0001
8.72E-04	2.42E-04	6.78E-07	-4.967644729	***	2.07E-06	<0.0001	***<0.0001
0.001175071	3.05E-04	5.91E-15	-7.80575074	***	5.77E-14	<0.0001	***<0.0001
0.001093879	3.43E-04	1.33E-11	-6.764865397	***	7.94E-11	<0.0001	***<0.0001
7.52E-04	1.47E-04	4.04E-09	-5.882446773	***	1.71E-08	<0.0001	***<0.0001
2.11E-04	4.11E-05	0.01537128	-2.423511072	*	0.024349839	0.0154	* 0.0154
9.26E-04	2.84E-04	2.70E-07	-5.14337304	***	8.55E-07	<0.0001	***<0.0001

8.17E-04	3.57E-04	0.0361342	-2.095414103	*	0.053258664	0.0361	* 0.0361
8.30E-04	2.00E-04	4.85E-17	-8.390264588	***	6.58E-16	<0.0001	***<0.0001
8.16E-04	3.33E-04	0.00901257	-2.611576993	**	0.015508939	0.009	** 0.009
7.23E-04	2.10E-04	3.66E-12	-6.949681602	***	2.43E-11	<0.0001	***<0.0001
3.70E-05	5.68E-06	2.05E-11	-6.702440018	***	1.18E-10	<0.0001	***<0.0001
6.43E-04	1.94E-04	1.19E-13	-7.418129748	***	9.15E-13	<0.0001	***<0.0001
3.72E-05	6.84E-06	1.61E-18	-8.781969435	***	3.03E-17	<0.0001	***<0.0001
5.66E-05	1.18E-05	4.53E-07	-5.045225073	***	1.40E-06	<0.0001	***<0.0001
5.74E-04	2.14E-04	1.64E-09	-6.030211763	***	7.30E-09	<0.0001	***<0.0001
5.54E-04	1.45E-04	1.13E-12	-7.113072459	***	8.02E-12	<0.0001	***<0.0001
5.48E-04	1.92E-04	4.29E-11	-6.593852998	***	2.38E-10	<0.0001	***<0.0001
4.98E-04	2.91E-04	0.02177802	-2.29421722	*	0.032958707	0.0218	* 0.0218
4.91E-04	1.72E-04	0.00340805	-2.928315064	**	0.006211437	0.0034	** 0.0034
4.80E-04	1.56E-04	7.51E-07	-4.947648046	***	2.25E-06	<0.0001	***<0.0001
4.04E-04	1.50E-04	2.82E-09	-5.941937203	***	1.21E-08	<0.0001	***<0.0001
0	0	6.61E-19	-8.88133847	***	1.32E-17	<0.0001	***<0.0001
2.97E-04	1.53E-04	4.67E-11	-6.581042343	***	2.55E-10	<0.0001	***<0.0001
2.76E-04	1.97E-04	0.01008927	-2.57275483	*	0.016765985	0.0101	* 0.0101
3.48E-05	1.02E-05	0.01152469	-2.526373423	*	0.01878303	0.0115	* 0.0115
1.25E-04	8.85E-05	0.02031131	-2.32054699	*	0.031585013	0.0203	* 0.0203

Supplementary table S19. The relative abundance and distribution of the key 90 OTUs among the CC (n=73), CCR (n=21), and H (n=140) groups.									
	CC01	CC02	CC03	CC04	CC05	CC06	CC07	CC08	CC09
OTU14 (Streptococcus)	0	0	0.012127503	6.71E-05	0	0.010277623	0.000872492	0.000131274	0
OTU23 (Solobacterium)	0	0.022628372	7.50E-05	0.004333483	0.008075023	0.008751423	0.000336994	0.003761679	0.01600664
OTU45 (Atopobium)	0.009718131	0.071386964	0.000307627	0.001744649	0.000822134	0.005559728	0.000807863	0.00541392	0.010216786
OTU21 (Campylobacter)	0.021703344	0.007956251	0.000725299	0.002930837	0.024698841	0.001659439	0.000526265	0.000583943	0.000481634
OTU56 (Selenomonas)	0.06207763	0.016097531	0.000212588	0	0	0.000835776	0.005839692	0.000923445	0.002105867
OTU466 (Prevotella)	0.100747993	0.115423888	0.000247602	0.008811993	0.088553533	0.050824875	0.003822344	0.000434562	0.011656564
OTU385 (Prevotella)	0.006108127	0.002069585	0	0.000201306	0.013091431	0.003324935	0	0.000122221	0.004534532
OTU111 (Actinomyces)	0.106119679	0	0.000110045	0.003433019	0.000278689	0.001695778	0.015543276	0.029011552	0.032090137
OTU31 (Actinomyces)	0.038728123	0.099127622	0.000587742	0.007809794	0.001992629	0.011743259	0.561746091	0.865611646	0.033145633
OTU174 (Actinomyces)	0.001949402	0.01588509	7.00E-05	0.009141009	0.005155752	0.003900288	0.146209278	0.040921272	0.00170109
OTU97 (Catonella)	0.001906082	0.005797578	0	0.009790382	0	0.000617748	0	0.000144854	0.000906906
OTU2 (Fusobacterium)	0.011739733	0.187770262	0.017682294	0.314898351	0.000411067	6.66E-05	0.003379174	0.000135801	0.431487583
OTU142 (Peptococcus)	0	0	0.000172571	0.000222951	0	0.000260423	0	0	0
OTU598 (Alloprevotella)	0	0.000301529	0	0	0.001616398	0	0	0	0
OTU144 (Prevotella)	0	0.002535584	0	0.000380966	0.013335284	0.000666198	0	0	0.000958144
OTU54 (Alloprevotella)	0	0.027809187	0	0	0.021647193	0.000884227	0	0	0
OTU12 (Alloprevotella)	0	0.07350452	0	0.000404776	0.017007016	0.012445796	0	0	0.004134878
OTU84 (Alloprevotella)	0	0.000938851	0	0	0	0.001980426	0.000180038	0	0
OTU177 (Prevotella)	0	0	0	0	0	0	0	0	0
OTU60 (Clostridia_UCG-014)	0	0.000568793	3.00E-05	0.006543517	0	0.002622399	0.000124642	0	0
OTU11 (TM7x)	0.002036042	0	0.000477697	0.002142931	0.505765385	0.265679886	0.00408086	0.00202343	0.074371442
OTU704 (Saccharimonadaceae_unclassified)	0	0	4.25E-05	0.000138533	0.034655017	0.028991739	0.000553963	0.000126747	0.006824854
OTU75 (Candidatus_Saccharimonas)	0	0	0	0	0	0.000520846	0	0	0.010775277
OTU225 (Candidatus_Saccharimonas)	0	0	0	0	0	0	0	0	0.000819802
OTU153 (Acinetobacter)	0	0	0.000260107	0	0	0	0	0	0
OTU44 (Pseudomonas)	0	0	0.000255105	0	0	0	0	0	0
OTU152 (Pseudomonas)	0	0	0.002233421	6.06E-05	0	0.000127183	0	9.96E-05	0
OTU146 ([Eubacterium]_ruminantium_group)	0	0	0	0	0	0	0	0	0
OTU92 (Faecalibacterium)	0	0.000801793	0.000575237	0.000116887	0	0	0	0	0
OTU132 (Bacteroides)	0	0	0.000245101	0	0	7.87E-05	0	0	0
OTU234 (Prevotella)	0	0.000287823	0	0	0	0	0	0	0
OTU99 (Prevotella)	0	0	6.25E-05	0	0	0	0	0	0
OTU80 (Fusicatenibacter)	0	0	0	0	0	0	0	0	0
OTU208 (Bacteroides)	0	0	5.00E-05	0	0	0	0	0	0
OTU913 (Porphyromonas)	0.003884364	0.000109647	0.000562732	0.001268442	0.002556975	0	0	0	0.005323591
OTU26 (Prevotella)	0.005530526	0.002186084	0.000592745	0.001478406	0.001741808	0	0.000387774	0.000470776	0.002433788
OTU5 (Haemophilus)	0.012086294	0.000527676	0.250620881	0.021992104	0.005580754	7.87E-05	0.000540114	0.000126747	0.008689905
OTU62 (Bergeyella)	0.001920522	0.003529259	0.002310953	0.000476207	0.000285657	0	0	0	0.001988021
OTU628 (Porphyromonas)	0	0	0	0	0	0	0	0	0
OTU308 (Alloprevotella)	0	0.000137059	0	6.71E-05	0	0	0	0	0
OTU35 (Actinobacillus)	0	0	0	0	0	0	0	0	0.00016396
OTU77 (Haemophilus)	0	0	0	0	0	0	0	0	0
OTU42 (Haemophilus)	0	0	0	0	0	0	0	0	0
OTU172 (Aggregatibacter)	0	0	0	0	0	0	0	0	0
OTU71 (Aggregatibacter)	0	0	0.00075031	0	0	0	0	0	0
OTU78 (Saccharimonadales)	0	0	0	0	0	0	0	0	0
OTU65 (Alloprevotella)	0	0	0	0	0	0	0.000156956	0	0
OTU48 (g__Absconditabacteriales_)	0	0	0	0	0	7.27E-05	0	0	0

OTU53 (g__Absconditabacteriales_)	0	0	0	0	0.045781689	0	0	0	0.000204951
OTU33 (Rothia)	0.007942009	0.000342646	0	0.002493593	0	0.000605635	0	0	0
OTU106 (Cardiobacterium)	0	0	0.003281354	0.000380966	0.000627051	0	0	0	0
OTU46 (Campylobacter)	0	0	0.016451786	6.49E-05	0	0.00044817	0.000189271	0	0.007859855
OTU100 (Campylobacter)	0	0	0.000497705	0	0.001128692	0.001229439	0	0	0.00099401
OTU126 (Prevotella)	0	0	0	0	0	0.000169578	0	0.000140327	0
OTU63 (Porphyromonas)	0	0.000130206	0.000125052	0.000400447	0.017223	0	0	0	0.005262106
OTU57 (Parvimonas)	0	0.000575646	0	0.001396152	0	0	0	0	0
OTU37 (Porphyromonas)	0	0.000137059	0.025888179	0	0	0	0	0	0
OTU161 (Clostridia_vadinBB60_group)	0	0.000143912	0	0	0	0	0	0	0
OTU367 (Fusobacterium)	0	0.018050616	0.001558143	0.020693357	0.00057828	0	0.000364692	0.000407402	0.028257561
OTU96 (Tannerella)	0	0	0	0	0	0	0	0	0
OTU86 (Corynebacterium)	0	0	0	0	0	0	0	0	0
OTU114 (Prevotella)	0	0	0.000102542	0	0.000174181	0.003191696	0	0	0.000158837
OTU134 (Prevotella)	0	0.00084291	0.000790326	0	0	0.001405073	0	0	0.000199827
OTU444 (Leptotrichia)	0	0	0.000522716	0.000248926	0	0.000193803	0.035799853	0.00468965	0.100594869
OTU562 (Leptotrichia)	0	0	0	0	0	0.000121127	0.055982569	0.002172811	0.008613048
OTU1106 (Leptotrichia)	0	0	0	0	0	0	0.006389039	0.000746904	0.000942773
OTU961 (Leptotrichia)	0	0	0	0.000699158	0	0	0	0	0.001337303
OTU966 (Streptococcus)	0	0	0	0.000482701	0	0	0	0	0.0005175
OTU767 (Gemella)	0	0	4.50E-05	0.000132039	0	8.48E-05	0	0	0.000650718
OTU1077 (Streptococcus)	0.003826604	0.000746969	0	3.46E-05	0.000710658	0.000254367	0	0.000113167	0
OTU493 (Prevotella)	0.009573731	0.005139697	0	6.28E-05	0.003455748	0.000920565	0	0	0.000251064
OTU673 (Prevotella)	0.185669728	0.01904429	0	0.001757637	0.00025082	0.009532692	0.001518782	0.002209024	0.009340623
OTU611 (Prevotella)	0.056994744	0.014487093	0	0.001225151	0	0.003555076	0.00054473	0.000144854	0.0060153
OTU1065 (Prevotella)	0.175720557	0.000781234	0	0	0.01651931	0.008466775	0.000193887	0.025584848	0
OTU761 (Prevotella)	0.060056027	0.003700582	0	0.000380966	0.003288534	0.003143245	0.000867875	0.001235786	0.0016601
OTU1016 (Veillonella)	0.002021602	0	0	0	0	0	0.000923272	0.000122221	0
OTU706 (Bacteria_unclassified)	0	0.001576174	0	0.000266243	0.000271722	0.001174932	0.000794014	0	0
OTU399 (Streptococcus)	0.001833882	0.00124038	0	0.002982787	0	0.00125972	0.002114292	0	0.00055849
OTU820 (P5D1-392)	0.005530526	0.002357408	0	0.003363753	0	0.001302115	0.000909422	0.000122221	0
OTU633 (Rothia)	0.005487206	0.003858199	5.75E-05	6.49E-05	0	0.000236198	0.033962543	0.000846491	0
OTU939 (Actinomyces)	0.015624097	0.001994202	0	0	0.000257788	0.000720705	0.045069499	0.003634932	0.000194703
OTU626 (Actinobacteria_unclassified)	0.003913244	0.000150764	0	0	0	0.000127183	0.007132272	0.001267473	0
OTU794 (Lactobacillales_unclassified)	0.001848322	0.000294676	0	7.79E-05	0.000801232	0.003215921	0.002875991	0.001018505	0.000348416
OTU15 (Halomonas)	0.001877202	0.029042714	0.083034252	0	0.000411067	0	0	0.000122221	0.01544815
OTU93 (Xanthomonadaceae_unclassified)	0.002007162	0.000856616	0.004149212	0	0	0	0	0	0.000727575
OTU82 (Pelagibacterium)	0	0.0033922	0.005737367	0	0	0	0	0	0.002618244
OTU101 (Rhizobiaceae_unclassified)	0	0.001528203	0.003098778	0	0	0	0	0	0.00091203
OTU692 (Lautropia)	0	0	0	0	0	0	0	0	0
OTU17 (Lautropia)	0.001920522	0.000143912	0	0.000129875	0	0	0	0	0.000379159
OTU1050 (Lautropia)	0.009472651	0.002425937	0	0	0	0	0	0	0.000174208
OTU9 (Gemella)	0	0.000157617	0.115807771	0.042575068	0.002612712	0.016739747	0.000143107	0.000439089	0.029056869
OTU1 (Streptococcus)	0.029573153	0.19459578	0.312761514	0.38454795	0.131506525	0.292969791	0.005655038	0.000461722	0.097571848
OTU70 (Streptococcus)	0.007884249	0.013781241	0.126814811	0.121201167	0.011537738	0.215109378	0.00039239	0.000226334	0.015617234
OTU28 (Leptotrichia)	0.024966788	0.021100169	0.007888254	0.015881502	0.015592668	0.019846653	0.052894225	0.004250561	0.002587501
OTU137 (Oribacterium)	0	0	0	0	0	0.000308874	0.000175422	0	0.000128094

CC10	CC11	CC12	CC13	CC15	CC16	CC17	CC18	CC19	CC20	CC21
0	0.000863707	0	0.083864835	0	4.81E-05	0.002178831	0.000433967	0	0.008966622	0.003308005
0.000791157	9.38E-05	3.29E-05	0.007984791	0.005902961	0.000377748	0.026200079	0.001130333	0.000532744	0.021952617	0.002302159
0.000435238	9.14E-05	0	0.002741322	0.109189942	0.002642458	0.05663978	0.018687502	0.000513013	0.013442094	0.000386736
0.015654332	0.000252616	0	0.002256838	0.019749414	0.00142012	0.057313594	0.000960447	0.004505304	0.000701252	0.001153569
0	0.000375316	0	0.013657549	0	0.000203129	0.120863663	4.04E-05	0	0.000570124	0.000317024
0.002873791	0.000979189	0	0.01562615	0	0.000527422	0.006511902	0.000570213	0.418782844	0.002927586	0.0010573
0.000256262	0.00051245	0	0.002189378	0	0	0.000221326	2.02E-05	0.070401137	0.000293614	0
0	0	0	0	0	0.000561277	0.00346744	0.005466641	0	0.038057189	0.000411633
0.070065184	0.041891014	2.23E-05	0.005090151	0.267943707	0.000992481	0.148337596	0.023077635	0.000578784	0.641906665	0.002698854
0.017053601	0.040365692	0.00267823	0.000944438	0.114437019	0.00017462	0.007284084	0.000581987	0	0.129891306	0.001266436
0	0	0	0	0	0	0.006152862	6.39E-05	0	0.001490874	0.003679802
0.001488758	9.62E-05	2.70E-05	0	0.000232124	0.010769395	0.005965965	0.003779551	0.004906507	0.004083512	0.072417582
0	0	0	0	0	0.000133637	7.87E-05	0.000650951	0	0	0
0.000581673	0.000182846	0	0	0	2.32E-05	0	0	0.000545898	0	0
0.000178976	0	0	0.000306636	0	1.60E-05	0.00326087	0.000829247	0	0.001879983	0.000300426
0.004427632	0	5.05E-05	0.00029437	0	9.09E-05	0.002282117	0.000605536	0	7.84E-05	0
0.045675585	9.14E-05	0	0	0.00015115	0.000538113	0.000403305	0.000267445	0	0.000651366	0.000992567
5.08E-05	0	0	0	0	4.28E-05	0.000393468	0.000385188	0	0	0
0	8.18E-05	0	0	0	3.03E-05	0.001441078	0.011782714	0	0	5.15E-05
0	0	0	0	0	9.62E-05	0.001873893	0.000465926	0	0	0.00079505
0.000144401	0	0.000132679	0	0	0.000112255	0.017430651	0.002018452	0	0.026420962	0.002247385
0	0	1.76E-05	0	0	3.21E-05	0.002016526	0.00015643	0	0.00290193	3.49E-05
0	0	0	0	0	0.000370621	0.010667913	0.000689638	0	0	0.000413293
0	0	0	0	0	0	0.003029707	0.000129517	0	2.57E-05	0.000126146
0.001004708	0	2.23E-05	0	0	1.96E-05	9.84E-05	2.86E-05	0	0	0.000162662
0.000162706	8.18E-05	5.52E-05	0	0	8.55E-05	0	6.90E-05	0	0.000380558	6.31E-05
0.000421001	7.94E-05	0.000165555	0	0.000148451	5.35E-05	0	8.41E-06	0	0	0
0	0	0	0.000625537	0	0	0.001209915	0	0	0	0
0.001289443	7.46E-05	0	0.000355697	0.000124159	0.001858451	0.070701357	1.51E-05	0	0.00036773	2.82E-05
0.000801326	9.14E-05	0	0	0.000143053	0.001739068	0.06866024	1.51E-05	0	5.27E-05	0
0.001018945	0	0	0.000349565	0	0.001938634	0.071945701	0	0	0.000168187	4.15E-05
5.29E-05	0	0	0	0	0.00048644	0.01561086	0	0	4.28E-05	0
5.69E-05	0	0	0	0	0.000438331	0.010092465	0	0	8.84E-05	0
0	0	0	0	0	0.000178183	0.004087153	0	0	0	0
0.031670683	0.001287141	4.23E-05	0.188703545	0	5.52E-05	0.000172142	0.011027476	0.001591655	0.000989165	0.057180511
0.100702686	0.00497534	0	0.011247394	0.261117649	0.000571968	0.005729884	0.000124471	0	0.005923586	0.004576101
0.0103969	0.000370504	0.074270678	0.034582362	0.000118761	0.010192082	0.014646862	0.001784648	0.000552475	0.000156784	0.115215825
0.007464128	0.000230964	0	0	0	0.000167492	0.000147551	0.000104287	0	0	0.003566935
0.000469813	0	0	0.005875138	0	0	0	0.013811258	0.000605092	0.000135404	0.007860868
5.49E-05	0	0	0	0	0	0	0	0	0	0
0	0	0	0	0	2.67E-05	0	0	0	0	0.027474862
0	0	0	0	0	0	0	0	0	0	0.001825793
0	0	0	0	0	0	0	0	0	0	0.000542758
0	0	0	0	0	0	0	0	0	0	0.009351379
0	0	0	0	0	3.74E-05	0	0	0	0	0.000101249
0	0	0	0	0	0	0	0	0	0	0
0.002576853	0.000101047	0	0.001747823	0	0.00035993	0	0	0	0.000149657	0.001726204
0	0	0	0	0	0	0	3.36E-06	0	0	0.000849824

0.000551166	0	0	0	0	0	0	0	0	0	0.000109548
0.035654942	0.050814387	0	0.000300503	0.002869158	0.003903995	0.00047708	0.078388266	0.000559052	0.003081519	0.006946312
0.000530828	8.42E-05	0	0.000318901	0	3.03E-05	0	5.89E-05	0	0.000222348	0.001449016
0.000142368	0	0	0.00029437	0	0.015845836	0.011489278	0.002391866	0.002729491	2.42E-05	4.65E-05
0	0	0	0	0	0.000277966	0.000624631	0.001759417	0	8.84E-05	0
0.00034575	0.000288704	0	0.001729425	0	0.000716297	0.007200472	0.00781982	0	0	0
0.000677263	9.62E-05	2.70E-05	0	0	0.002505256	0.002749361	0.000326316	0	4.85E-05	0
0.000101691	0	2.82E-05	0.000601006	0	0.005938848	0.000103285	5.55E-05	0	0.001039051	0
0	0	0	0.000318901	0	0.000575532	0	0.000442377	0	0	0
0	0	0	0	0	0	0	0.000338091	0	6.13E-05	0
0.00286769	0.000173223	0	0	0	0.001933288	0.020052135	0.021190382	0.000460396	0.000424742	0.00731479
0.002013484	0	4.58E-05	0	0	0	0	0.000649269	0	2.71E-05	0
0	0	0	0	0	0.003160971	0	0.001246394	0	0	0.000167641
5.69E-05	7.22E-05	0	0.000527413	0	0.000204911	0.013313988	0.002079006	0	0	0
0.015918728	0.001599904	0	0.004053723	0	0.012455009	0.002203423	0.059808415	0	0.000786771	3.82E-05
0	0	0	0	0	0.000383094	0.001116467	0	0.00155877	0	0.000308725
0	0	0	0.003734822	0	0	0.000826284	0	0	0	0
0	0	0.000636388	0.000355697	0	0	7.38E-05	8.41E-06	0	0	0
0	0	0	0.000367963	0	0	0	0	0	0.000119726	0
0	0	0	0	0	1.96E-05	0	0.008090628	0	0	0
0	0	0	0	0	2.49E-05	0	0.005796321	0	0	0
0	0	0	0.000944438	0	2.85E-05	0	6.22E-05	0.003314852	0	0
0	0	0	0.004311296	0	0	0	0	0.014377512	5.27E-05	0
0	0.000245399	3.29E-05	0.003869741	0	0	0.003659256	5.21E-05	0.11053452	0.000242303	0
0	0.000190064	2.11E-05	0.004145713	0	0	0.000368877	0	0.063317614	0	0
0	0	0	0.000637802	0	0	0.003433012	0.000629084	0.001631118	0.000364879	0
0	0.001448334	0	0.021225316	0	0	0.000231163	3.70E-05	0.019540525	0	0
0	0.000173223	0	0.00029437	0	0	0	2.02E-05	0	0	0
5.08E-05	0.024946469	0.000735016	0.005599166	0	0	0	0.006512872	0	0.000186716	0
0	0.001643209	0	0.013583957	0.000143053	0	0	0.002607167	0	0.000166761	0
0	0.012411885	0	0.011903594	0.000345487	0	0	0.002655946	0	2.57E-05	0
0	0.034656562	1.64E-05	0.001030296	0.000197035	0	0	0.001513839	0	0.001727475	0
4.47E-05	0.008947432	2.47E-05	0.002943702	0.000834026	0	0.000324611	6.56E-05	0	0.000205245	0
0	0.001294358	0.000139724	0.001195879	7.02E-05	0	0	0.000768694	0	0.000639964	0
0	0.003488512	9.86E-05	0.000576475	0.00012146	0	0	0.000926806	0	0.002425877	0
0.382033212	0.001871767	0.000292363	0.010014719	0	0.047585617	0.049173716	0.011412664	0.000578784	0.027776432	0.118324653
0.019748416	0	0	0	0	0.00180856	0.001701751	0.000657679	0	0.002313277	0.005218448
0.028819264	9.86E-05	5.75E-05	0	0	0.002480311	0.002247688	0.000640859	0	0.003691552	0.007294872
0.013492378	0.000113076	0	0.001306268	0	0.001443284	0.002257525	0.000536572	0	0.00232753	0.003289747
0	0	2.00E-05	0	0	0	0	0	0	0	0
7.93E-05	0	0.013803274	0.000349565	0.012189197	6.59E-05	0.017228999	0	0.002288826	2.71E-05	0.022917019
6.51E-05	0	0.000697443	0	0.000580309	0	0.001691914	3.20E-05	0	0	0.014554888
0	5.29E-05	0	0	0.016882956	0.181994583	0.014371434	0.185231659	0.001664003	2.57E-05	0.10416314
0.089437343	0.376424877	0.788492653	0.331399485	0.073885536	0.556441324	0.059221916	0.294646897	0.002163862	0.035269284	0.326199463
0.079404497	0.305403585	0.047495427	0.189482399	0.017501053	0.118570258	0.029539642	0.199946175	0.001026026	0.011911312	0.045274682
0.010142673	0.08029111	0.069818312	0.004139581	0.095122162	0.003542283	0.006197128	0.000743463	0.270739199	0	0.011854375
0	0	0	0	0	0.000652151	0.001003344	6.56E-05	0	0	0

CC22	CC23	CC24	CC25	CC26	CC27	CC28	CC29	CC30	CC31	CC32
0.005353547	0.008221349	0.003877254	0.039105717	5.08E-05	0.02095346	0.012571677	0.151064063	0.009012671	0.000714054	0
0.063768302	0.028016443	0.089480662	0.010092622	0.000730884	0.003821396	0.00060039	0.006565368	0.011892974	0.003776814	0.005321788
0.002021647	0.001778822	0.012736588	0.0015799	8.90E-05	0.003076458	0	0.003447845	0.005263761	0.001091735	0.001217697
0	0.001419637	0.054661336	0.002584904	0.000327944	0.002000604	0.000440151	0.002302472	0.008637433	0.003493553	0.001082398
0.007314907	0.001448143	0.009149352	0.005536037	0.000106773	0.003765488	0	0.049016465	0.035605958	0.023782125	0.007967648
0.073715772	0.129529011	0.107831155	0.006707122	0.000258034	0.000705651	0.000237316	0.01267337	0.012778953	0.043580892	0.007396383
0.010064026	0.004036557	0.003915232	0.000532311	0	0.000119371	0	0.001620331	0.000955468	0.001852999	0
0.001913129	0.026123594	0.120312252	0.022182476	0.00107281	0.004125113	0.000139956	0.001966288	0.119054816	0.003965655	0.002315128
0.198125455	0.028449745	0.057219702	0.371297775	0.004082779	0.012278634	0.005097229	0.388586149	0.086026885	0.02853855	0.009065079
0.049914191	0.013557815	0.030765991	0.009636964	0.000781728	0.001127229	0.001401586	0.008439792	0.006931488	0.003222094	0.001067364
0.001390636	0.00071837	0.004481456	0.001912062	5.34E-05	0.00134935	0.001348849	0.003692165	0.00310614	0.000761264	0.01029781
0.002339162	0.283260261	0.150546544	0.097698286	0.003992531	0.0491644	0.005078974	0.007011008	0.019206649	0.264795964	0.499315985
0	0.000267964	7.60E-05	0	0	3.48E-05	0	0	0.000284903	0.00032457	0
0	0	0.000825168	0	0	3.17E-05	0	0.000529686	0.000799119	0.000596028	0
0.001929206	0	0.004888861	0.001000745	4.32E-05	0.000642188	0.000103446	0.000715369	0.000819965	0.000336372	0.001157564
0.000462206	0	0.00150878	0.00107314	4.19E-05	0.000971593	0	0	0.004534131	0.000714054	0.005336821
0.001515231	0.001294207	0.012681347	0.001026296	0.000176683	0.000985192	0	0.007841696	0.008432441	0.00204184	0.003202093
0	0	6.21E-05	0	0	4.99E-05	0	0	0	0	0
0	0	0.00038669	0	0	3.48E-05	0	0	0	0	0
0	0.000307873	0.004346805	0	0	0.000933817	0.001413756	0.003854393	0.001118766	0.00032457	0
0.018648993	0.000370588	0.020090596	0.002980943	0.000246594	0.039743125	0.007446051	0.001665285	0.018640317	0.003936148	0.015364031
0.001442885	0	0.002085362	0.000481209	0	0.001982472	0.000456377	0.00017982	0.001184781	0.000283261	0.001052331
0.000486321	0	0.00182987	0	0	0.000815956	0	0.001049599	0.000618449	0	0
0.000506417	0	0.000887315	0	0	0.000947416	0	0.000103592	0.000100758	0.000318669	0
0	0	0	0	0	0	0	0	0	0	0
0	0	0	0	0	0	0	0	0	0	0
0	0	0	0	0	0	0	0	0	0	0
0	0	0	0	0	0	0	0	0	0	0
0	0	0	0	0	0	0	0.000152456	0	0	0
0	0	0	0	0	2.42E-05	0	0	0	0	0
0	0	0	0	0	3.48E-05	0	0	0	0	0
0	0	7.94E-05	0	0	0	0	0	0	0	0
0	0	0	0	0	0	0	0	0	0	0
0	0	0	0	0	0	0	0	0	0	0
0.008605063	0.001465247	0.000100125	0.000992228	8.52E-05	0.018427017	0.011778594	0.001305646	0.000903352	0.000354076	0.24923706
0.008564872	0.003147146	0.002427168	0.005054828	9.28E-05	0.002996374	0.000229203	0.001637922	0.001560019	0.000666844	0.002315128
0.00622169	0.001641989	0.005213404	0.012656233	0.021568057	0.012100332	0.005630683	0.001657467	0.014794123	0.001321885	0.006569552
0	0	0.000435026	0	4.32E-05	0.00139317	0.000137927	0.000160274	0	0	0
0	0	0.000207155	0.001085915	0	0.005474464	0.000853933	9.97E-05	0.000288378	0.000395385	0
0	0	0	0	0	9.97E-05	0	0	0.000156349	0	0
0	0	7.25E-05	0	0	0.000474464	0.000119672	3.71E-05	0	0	0
0	0	0	0	0.001609215	0.000773648	0.000486803	2.15E-05	0	0	0
0	0	0	0	5.34E-05	0.000657298	0	0	0	0	0
0	0	0.000417763	0	0	0.003921124	0.000255571	3.13E-05	0	0	0
0	0	0.000203703	0	0.000202105	0.002949532	0	0	0.000128554	0	0
0	0	0	0	0	0.001736174	0	0	0.000844286	0	0
0	0	0.000445384	0.00050676	0	8.01E-05	0	0	0	0	0
0	0	0.002561819	0.000996487	0	0.000737383	0.000105474	0	0.000441253	0.000348175	0

0	0	5.87E-05	0	9.91E-05	0.002364763	0.000961435	0	0.0001494	0	0.001052331
0.000478282	0	0.001840228	0.002593421	0.001189751	0.010754004	0.011224856	0.003854393	0.000816491	0	0
0	0	0.000203703	0	0	0.000281052	0	0.000613732	0	0	0
0	0.000627149	0.012560507	0.001520281	0.000437259	0.004867029	0.000109531	0.000187638	0.002369561	0.000300965	0.001127497
0	0	7.94E-05	0	0.000474121	0.000297673	0	0	0.000250159	0.001675961	0
0	0	0	0	0.000101688	0.001669689	0	3.91E-05	0	0	0
0	0.003477825	0.007989283	0.005148515	9.28E-05	0.001618314	0.002517175	0.000220865	0.000747002	0.007435602	0.003472692
0	0	3.11E-05	0.000498243	0	0.000213055	0.000141984	8.60E-05	0	0	0
0	0	6.91E-05	0	0	0.001592626	0	0	0	0.000365879	0
0	0	0	0	0	5.29E-05	0	0	0	0	0
0.000526513	0.016049305	0.017987971	0.008674545	0.000279642	0.010311272	0.004135794	0.000795506	0.001459261	0.087090968	0.017934725
0	0	0	0	0	0.00080689	0	0	0	0.000761264	0
0	0	0.000286565	0	0	0.000312783	0.000263685	3.13E-05	0	0	0
0	0.000376289	0	0	0	5.74E-05	0	0	0	0	0
0	0	0.000227871	0.000498243	0	0.000459353	0	6.45E-05	0.000135503	0.001050426	0
0.00577958	0.003631761	0.010592533	0.003100181	0.001651161	0.001751284	0.010470312	0.005496224	0.050671086	0.072119442	0.007712082
0.007966014	0.003398006	0.005665693	0.000574896	0.001193565	0.001790571	0.001730178	0.002773521	0.01032948	0.037868461	0.015138532
0.002419546	0.001106062	0.001712482	0.00098797	0.003138351	0.000302206	0.007078921	0.003907166	0.008550572	0.062464961	0.003036726
0.002415527	0.002519997	0.001622715	0.011319067	0.001030864	0	0.004034376	0.000506231	0.011403079	0.001705468	0.002510561
0.000458186	0.000381991	0	0.000493985	0.027089723	0.000142037	0.021340208	0.002280972	0.001355028	0.003534862	0
0	0	7.25E-05	0.001562866	0.012500016	9.07E-05	0.01191855	0.001168827	0.000510741	0.00162285	0
0.005863983	0.003751489	0.000825168	0.002670073	0.000538947	5.89E-05	0	0.000686051	0.000938096	0.004703314	0.001127497
0.008910521	0.007075378	0.00635621	0.002610455	0.000130923	5.44E-05	0	0.003959939	0.006924539	0.008686672	0.005622454
0.134120021	0.16634264	0.045501626	0.002069626	0.000838927	0.000250831	0.000111559	0.016764264	0.005955173	0.046950518	0.005938153
0.105736575	0.162283277	0.037902485	0.002065368	0.000422006	0	0.000125757	0.003928666	0.004780816	0.041232186	0
0.011125089	0.001681899	0.001456991	0.000949643	0	0.001491387	0	0.022559536	0.000538537	0.001268773	0.001067364
0.057080388	0.020348125	0.011338291	0.003155541	0.000190665	0.000462375	0	0.0012001	0.002383459	0.004791833	0.00450999
0.000470244	0	0	0.000532311	9.66E-05	0	0	0.00048864	0.000281429	0	0
0.00881808	0.001037646	0.000417763	0.002486958	0.000729612	0.00013146	0.026121015	0.00142292	0.003290285	0.003735505	0.003262226
0.00195734	0.002291943	0.001719388	0.00448419	0.013326232	0.000128438	0.012539223	0.001661376	0.004575824	0.00343454	0
0.000486321	0.003278277	0.001446633	0.004705632	0.010752251	5.44E-05	0.01359599	0.001493284	0.003634254	0.004272521	0.002164795
0.070484351	0.005809677	0.00109447	0.032832961	0	0	0.009746193	0.005290995	0.004429898	0.007205453	0.002239962
0.073498736	0.00839239	0.005845227	0.036648568	0.001249493	0.000107283	0.000746431	0.004765218	0.009957716	0.001752678	0.003247193
0.008709562	0.003266875	0.001729745	0.033812413	0.002619741	4.38E-05	0.003261577	0.010497944	0.011399605	0.008379806	0.003623025
0.004288465	0.001362623	0.000794095	0.017740871	0.002590506	0	0.002961382	0.012474004	0.008866745	0.003776814	0
0	0	0.000583487	0.001085915	0.000546574	3.78E-05	0.002515147	0.004456398	0.00121605	0.001321885	0.002285061
0	0	0.000103578	0	4.96E-05	0	0.000123729	0.000525777	0.000128554	0	0
0	0	0.000148461	0	0	0	0.000117644	0.00042414	0	0	0
0	0	0.000127746	0	5.21E-05	0	0	0.000666505	0	0	0
0	0	7.94E-05	0.003547322	0	0	0.000900585	3.52E-05	0.000309224	0	0.006780018
0.000438091	0	5.18E-05	0.003670819	5.97E-05	0.001860079	0.001377246	9.58E-05	0.000142452	0	0.001202664
0	0	0.000110483	0.004130736	0	0.00300544	0.00118861	4.30E-05	0.000118131	0	0.004434823
0.002849598	0	0.004187986	0.013124667	0.06842977	0.065432155	0.113893548	0.063116664	0.005030975	0.009760703	0.008298381
0.015353266	0.039778331	0.09412439	0.178937507	0.741938354	0.588760955	0.623174237	0.139727612	0.412605927	0.160131008	0.051233482
0.005462065	0.005638637	0.011286502	0.013073565	0.044693213	0.094203687	0.049799904	0.016097759	0.04559147	0.017438258	0.010718742
0	0.001037646	0.004957913	0.001971681	0.025361025	0.002641281	0.005839603	0.008121198	0.004030339	0.00162285	0.000977164
0	0	0	0	0.000396584	0	0	4.30E-05	0	0	0

CC33	CC34	CC35	CC36	CC37	CC38	CC39	CC40	CC41	CC42	CC43
0.034634945	0.033812482	0.000390995	0.0021285	0.010107558	0	0.000157106	0	0.012282511	0	0.000316946
0.000831362	0.012430669	0.000351007	0.002300729	0.003577853	0.002737628	0.00091684	0.017259957	0.004803886	0.006961102	8.69E-05
0.002191306	0.013186458	0.023099788	0.000625551	0.002825663	0.00409687	6.10E-05	0.018864061	0.002435211	0.000408989	0.00015707
0.014076701	0.000975523	0.001972746	0.002827167	0.001046633	0	0.003493839	0.014768478	0.009814032	0.00235445	0.000479626
0.034039649	0.004522658	0.002128255	4.39E-05	0.006410938	0	0.000264969	0.053754528	0.018590106	0.011857915	3.65E-05
0.004238919	0.009272294	0.495243639	0.003311361	0.00687116	0.001608117	5.16E-05	0.073930151	0.03388669	0.000411752	8.41E-05
0.00088268	0.000842716	0.025232486	0.000248596	0.00032166	0	0	0.000214531	0.001676702	0.000373064	0
0.062562545	0.004206338	0.009539382	0.028565765	0.001346025	0.001340098	0.000320073	0.002647502	0.032522705	0	0
0.015200579	0.059296803	0.317629861	0.035092622	0.14708786	0.065913659	0.005485796	0.088596239	0.051605176	0.000447677	0.010111408
0.000980186	0.006524411	0.009139501	0.00195952	0.024146796	0	0.000275521	0.017050303	0.004091952	0	0.000715231
0.000677406	0.002578857	0.000368779	0.001524071	0.001402934	0	0.000131312	0.000175525	0	0	0
0.002704492	0.066615637	0.001897213	0.07302866	0.022456842	0.011371686	0.006431946	0.001360319	0.003233641	0.00079587	0.000678769
0	3.62E-05	0	0	0.000309289	0	3.52E-05	0	0	0	0
0	0.000347711	0.000373222	5.36E-05	0	0	0	0	0	0	0
0.001724306	0.013019846	0.000377665	6.50E-05	0.000192996	0.002948215	0	0.009926913	0.000805083	0	2.52E-05
0.003089382	0.003916578	0.00327458	0.002208115	0.000170727	0	0.000110208	0.004085832	0	0	0
0.000538846	0.014084711	0.000337677	0.00378743	0.001031787	0.001454963	0.000119588	0.005173112	0.007997605	0	0.000126217
0	0.00039359	0	0	0.000180625	0	0	0	0.001603513	0	0
0.000251461	9.18E-05	0	2.92E-05	0	0	0	0	0.00081839	0	0
0.004223523	0.00486554	0	0.000485818	0.000504759	0	0.000200485	0.003208207	0	0	0
0.003812974	0.143112062	0.00146623	0.011407783	0.005824527	0.002756772	0.001987267	0.010048806	0.004710736	0.000201731	2.24E-05
0.000328439	0.00936888	0.000422097	0.001613435	0.0008091	0	6.80E-05	0.00133594	0	0	0
0.000277121	0.009818007	0	0.000178729	0.000677961	0	0.000334142	0.000151147	0.000745201	0	0
0	0.005524742	0	0.000152732	0	0	4.22E-05	7.31E-05	0.000851658	0	5.33E-05
0	0	0	1.95E-05	0	0	0	0.00038518	0	0	0.000462797
0	0	0	7.47E-05	0	0	0	0	0	0	0.000513284
0	0	0	2.44E-05	0	0	0	8.29E-05	0	0	0.000159875
0	0	0	0	0	0	0	0	0	0	0
0	0	0	0.000404577	0	0	6.21E-05	0.000297417	0	0	0.001705336
0	4.59E-05	0.000404324	0.000602804	0	0	4.10E-05	0.00010239	0	0	0.00131827
0	0	0	0.000568683	0	0	1.88E-05	0	0	0	0.001741799
0	0	0	0	0	0	0	0	0	0	0.000409505
0	0	0	0.000116986	0	0	0	0	0	0	3.37E-05
0	0	0	4.22E-05	0	0	0	0	0	0	0.000103779
0.000292516	0.065507308	0	0.062756618	0.018052569	0	0	0.008342313	0	0.575740325	0.003822982
0.000313044	0.039467616	0	0.001725547	0.002855354	0.00139753	0.004414196	0.0554854	0	0.021491262	3.09E-05
0.004526303	0.009977375	0.002776951	0.108857321	0.01466029	0.004364889	0.015588618	0.077850209	0.007891147	0.065733914	0.000558161
0.000292516	0.00141982	0	0.003407224	0.000685384	0	0	0.007957133	0	0	0
0	0.00035737	0	0.002890535	0.000524554	0	9.73E-05	0.04509042	0.00797099	0.004487824	0
0	0.002810664	0	0.000398078	0	0	0	0	0	0	0
0	0.000219734	0	0.069549944	0	0	0.00120643	0	0	0	0
0	0.000323564	0	0.013578528	0	0	7.97E-05	0	0.002362021	0	0
0	7.73E-05	0	0.003100135	0.000353826	0	0	0	0.000731894	0	0
0	0.000111074	0	0.003132632	0	0	7.86E-05	0	0.000765162	0	0
0.000282252	0.000173856	0	0.00122998	0	0	0	0	0.005835191	0	0
0.000261725	0.00908878	0	0	0	0	0	0	0	0	0
0	0.004215996	0	0.001311221	0.000168253	0	0	0.000711851	0	0.001588977	0
0.000626087	0.01114607	0	0.000162481	0.005265333	0	0	0	0	0	0

0	0.006985611	0	0.000155982	0.000707653	0	0	0	0	0	0
0	0.000275271	0	0.021203756	0.009083194	0	0.001980233	0.012910838	0.000831698	0	0
0	0	0	0.00059468	0	0	0	0.011453006	0	0	0.000804986
0.001016109	0.000456371	0.000390995	0.002216239	0.002016563	0	0.002483205	0.108367179	0	0.001414881	0.000546942
0	0.000101416	0	0.000225848	0	0	0.000460765	0.008371567	0	0	0.001245344
0.000343835	0.000246295	0	0.000903394	0.0005394	0	0.000335315	0	0.007112678	0	0
0.001375339	0.029615803	0	0.002814169	0.008266671	0.00153154	0.000483041	0	0	0	0
0	0.009042901	0	0.00101713	0.003924256	0	0.000152416	0	0.00070528	0	2.52E-05
0.000841626	6.52E-05	0	0	0	0	0	0	0	0	0
0	0	0	0.000155982	0	0	0	0	0	0	0
0	0.005780696	0	0.021613208	0.005631531	0.001435819	0.00356184	7.80E-05	0.003187065	0.000190677	0.001127541
0	0.000297003	0	3.74E-05	0	0	0.000298969	0	0	0	0.000126217
0	0	0	0.000136484	0	0	6.80E-05	0	0	0	0
0.000579901	0	0	0.000102363	0	0.001569829	6.68E-05	0	0	0	0
0	0.000492591	0	0.000628801	0	0	0.0010376	0	0.000858312	0	0
0.01327613	0.001035889	0.000324348	0.000411077	0.00127427	0	0	0	0.022203001	0	4.49E-05
0.040372368	0.000835472	0	0	0.002372864	0.002871638	0	0	0.02755248	0.001738203	0
0.062434248	0.001192842	0	0	0.003464035	0.001474107	0	0	0.030686317	0.000533344	0
0.000323307	0.00060125	0	4.39E-05	0.00068291	0	0	0	0.000745201	0	0
0.00688696	0.002685102	0	0.000243721	0.017047999	0	0.000369315	0	0.000845005	0.000196204	0
0.005126731	0.00109867	0	0.000217724	0.011181409	0.003082225	0.000188761	0	0.000864966	0.00016857	0
0.013835503	0.000816155	0.003878845	0	0.000628475	0.024485498	0	0	0.003805848	0.00035372	0
0.008837068	0.001390844	0.001199643	0	0.000504759	0	0	0	0.013852756	0.001798999	0
0.079446375	0.00677795	0.006611365	0	0.003367537	0	2.93E-05	0	0.146152567	0.002525783	0
0.003079118	0.003890017	0.00663358	0	0.00111344	0	0	0	0.081725939	0.000566505	0
0.059929899	0.000622982	0.023499669	0	0.000195471	0.015698287	4.69E-05	0	0.008929106	0.00061901	0
0.010386891	0.000898253	0.015608685	2.76E-05	0.001796349	0.100660477	0	0	0.010718919	0.002625267	0
0	0	0	0	0.00017815	0.014549631	0	0	0.000864966	0.001354085	0
0.004695655	0.001197671	0	0.000191727	0.005878962	0	7.50E-05	0	0.002435211	0.007284424	0
0.001893658	0.002083851	0	7.64E-05	0.014566266	0	0	0	0.00321368	0.001558579	0
0.001590878	0.002525734	0.000688684	0.000108862	0.015019065	0	0	0	0.010299744	0.002011783	0
0.002334998	0.001161451	0.003127957	0	0.013566644	0	0	0	0.01287468	0	0
0.001524163	0.00039359	0.007091222	0	0.014526677	0.128534508	0	0	0.004225024	0.000212785	0
0.003479403	0.001726482	0.006829078	0	0.022380138	0.037427013	0	0	0.012302472	0.000212785	2.80E-05
0.00231447	0.002231146	0.004638619	0.000134859	0.013554273	0.019488848	0	0	0.006779999	0.000193441	0
0.001041768	4.10E-05	0	0.03808877	0.003134951	0.001416675	0.00020752	0.145866143	0	0.001539235	0.125670354
0	0	0	0.002201616	0.000316712	0	0.000303659	0.007474439	0	0	0.005920994
0	0	0	0.002702057	0	0	0.000215727	0.007445185	0	0	0.005993919
0	0	0	0.001556567	0	0	6.68E-05	0.005377891	0	0.000174097	0.004400776
0	0.000205246	0	0	0.005869064	0	0	0	0.00457101	0.000210021	0
0.000903208	0.000859619	0	0.002079755	0.00658414	0	0	0.000507072	0.002328753	0.000408989	0.308023493
0	0.000210075	0.000399881	0.004513719	0.003045876	0	0	8.29E-05	0.004817193	0.000204494	0.038184939
0.028404863	0.070954781	0.000742001	0.08590852	0.096359547	0.005877285	0.246571231	0.005928844	0.023660135	0.000395172	4.77E-05
0.411585694	0.259537303	0.012271901	0.311063161	0.385693042	0.494993778	0.636428546	0.049342025	0.311780166	0.177321565	0.437892676
0.034491253	0.017561821	0.009246136	0.05075253	0.043735912	0.029903322	0.046937029	0.007962009	0.018636681	0.093277107	0.046128214
0.003787315	0.000318735	0.000390995	0.002214615	0.001922539	0	0.014996541	0.109903022	0.002401943	0.008055424	3.37E-05
0	0	0	6.50E-05	0	0.015009094	0.000561594	0	0	0	0

CC44	CC45	CC46	CC47	CC48	CC49	CC50	CC51	CC52	CC53	CC54
0.003795376	0.038190895	0	0.000855015	0	0	0.000324491	5.42E-05	0.000119221	0.020106511	0.011808916
0.001084741	0.002477702	0.001477965	0.001729915	0	0.010317124	0	0.000264634	0.000302821	0.01301602	0.007151622
0.001187122	0.001521208	0.001433065	0.000944494	0	0.016284034	0.02783507	8.74E-05	0.000581799	0.015805793	0.007909024
0.001579579	0.001049848	0.000390382	0.004931251	0.234246671	0.001531132	0.001213506	4.18E-05	0.003824612	0.008894865	0.031829089
0.003071403	0.003261262	0.000997782	0.008232007	0.00731474	0.007669168	0.000600085	2.95E-05	9.54E-05	0.087582226	0.035798167
0.054027438	0.00200022	0	0.304286013	7.78E-05	0.13418116	0.476116603	0.000307714	0.000395814	0.184186324	0.100475925
0.002813015	0.00019436	0	0.013242795	0	0.009790235	0.020491803	3.45E-05	0.000100146	0.005820421	0.003055097
0.017341238	0.003441848	0	0.07751807	6.61E-05	0.035310595	0.011828349	5.79E-05	0.000548417	0.072231906	0.029127203
0.12140329	0.011881952	0.001495426	0.060606663	0.048193286	0.297593422	0.342035312	8.12E-05	0.007184261	0.290118861	0.41656744
0.002245049	0.000774378	0.000248198	0.015678594	0.013766177	0.031383693	0.015877814	8.00E-05	0.000920387	0.008128443	0.006860314
0.001823341	0.000388719	0.000235726	0.000845073	7.39E-05	0	0	8.49E-05	0.002584713	0.004559111	0.003288144
0.041617508	0.007228034	0.002389689	0.054114512	0.000365543	0.033635357	0.000960137	0.009110782	0.027287316	0.003153275	0.005567633
0	4.90E-05	0.000370427	0	9.33E-05	0	0	2.22E-05	0.000281362	0	0
0.000138944	0	0	0	0	0	0	0	0	0.000201459	0.000546203
0.000294952	0.001825756	0	0.001014088	0.000260546	0	0.000320046	0	0	0.005259839	0.000152937
0.000999425	0.001925231	0.001492932	0.00585586	0	0	0.006814302	0	0.001189826	0	0.000680933
0.00046071	0.000483603	0	0.000914667	0	0.001013249	0	0.000125547	0	0.000170802	0.006037368
0.000236449	0	0.000124723	0.000894783	0	0	0	3.20E-05	0.000119221	0	0
0.00040952	0	0	0	0	0	0	0	0.000109683	0	0
0.000541152	0.000624399	0	0	0	0	0	0	0.000238442	0.000862771	0.017088882
0.019620414	0.006416927	0.054077313	0.004672758	0	0.003409019	0.003151559	0.000153857	0.001754934	0	0.027000652
0.001628331	0.000125492	0.002773835	0.000934552	0	0.000193643	0	0	0.00020506	0	0.002490687
0.000270576	0.00109882	0.001249722	0	0	0	0	2.34E-05	0.000741555	0.00010073	0.002530742
0.000287639	0.000552471	0	0	0	0	0	0	0.000274208	8.76E-05	0.000673651
0	0	0.000619872	0	0.000377209	0	0	0	0.00037197	0	0
0	0	0	0	0.000392764	0	0	0	0.004420717	0	0
0	0	0.000120981	0	7.39E-05	0	0	0	0.001425884	0	0
0	0	0	0	0	0	0	0	5.01E-05	0	0
0.000380269	0	0.000115992	0	0.000182771	0	0	8.49E-05	0.00249172	0	0
0	6.89E-05	0.000134701	0	8.17E-05	0	0	0	0.001800238	0	0
0	0	0	0	0	0	0	3.20E-05	0.002219896	0	0
0	0	0	0	0	0	0	0	0.000777321	0	0
0	0	0	0	0	0	0	0	5.96E-05	0	0
0	0	0	0	0	0	0	0	5.01E-05	0	0
0.01588354	0.018350912	0	0	0.464441264	0	0	2.83E-05	0.002527486	0	0
0.007025225	0.003092919	0	0	7.00E-05	0.003566636	0	5.79E-05	0.00035051	0.001756199	0.000964959
0.055604579	0.029752322	0.024001656	0.000775479	0.00077775	0.002832593	0.00231144	0.030926439	0.101104225	0.000805837	0.000706423
0.000163321	0.002659819	0.001504157	0	0	0	0	0.001724427	0.000283746	0.000157664	0
0	0.013534774	0.000113498	0	0	0	0	4.92E-05	0.000543648	0	0.000345929
0	0.000123962	0	0	0	0	0	0	6.44E-05	0	0
0	0.000505029	0.000120981	0	8.94E-05	0	0	0.00229185	0.000393429	0	0
0.000141382	0.008906873	0	0	0	0	0	0.000406182	0.004938136	7.45E-05	0
0	0.007913649	0	0	0	0	0	0	0.001621406	0	0
0	0.001143202	0	0	0	0	0	0	0.001139753	0	0
0	0.00364539	0	0	0	0	0	0	0	0.000249634	0
0	0.001599258	0	0	8.17E-05	0	0	0	0	0	0
0	0.001467644	0.000127217	0	0.000132218	0	0	3.20E-05	0	0	0
0	0.001951248	0	0	9.72E-05	0.000274703	0	0	0.000236058	0	0

0	0.000699388	0	0	0	0	0	0	0.000209829	0	0
0.002886144	0.000615217	0.010332037	0	0	0	0	0.041133912	0.052581255	0.00010073	0.000138372
0	0.001078925	0	0	0	0.000513379	0.00030671	0.007110644	0.002587097	0	0
0.000948235	0.001026892	0.016357396	0	0.013692291	0	0	0.000855444	0.006220955	7.01E-05	0.00016022
0.00014382	0.001570181	0.001929462	0	0.064094388	0.000234173	0	8.00E-05	0.006452244	0	0.000502507
0.000828791	0.004125933	0.004408951	0.002614756	6.22E-05	0	0	6.03E-05	0.000360048	0	0
0.011195995	0.011260613	0.030711743	0	0	0	0.000657871	0.001198852	0.001487879	0.002513861	0.002188455
0.000392457	0.000870792	0.000885532	0	0	0	0	0.000769284	0.000233673	0	0.000149296
0.000129194	0	0	0	0	0	0	0	0.004043978	0	0
0	0	0	0	0	0	0	0	0.000639025	0	0
0.002530251	0.004144297	0.00366685	0.003927105	0	0.00274703	0	0.000748359	0.01056537	5.69E-05	0.000382342
0	0.000321382	0	0	0	0	0	0	0.001177904	0	0
0.000131632	6.27E-05	0	0	0	0	0	0	0.002026758	0	0
0	5.66E-05	0.008900219	0	0	0	0	4.06E-05	0.00238919	0.000218977	0
0.000475336	0.002500658	0.00648808	0	0	0	0	0	0.001156444	0.000512407	0.000163861
0.025685215	0.003204638	0.000120981	0.083065727	0	0.007020688	0	0.000108315	0.001909921	0.007988298	0.024218656
0.012395304	7.35E-05	0	0.18514063	0	0.009240829	0	0	0	0.004585388	0.025096223
0.017348551	0.00133297	0	0.036377917	0	0.001598681	0	5.29E-05	0	0.004694877	0.003204393
0.003641806	0	0	0.000984262	0	0.002310207	0	0	0	0.000280291	0.000316798
0.007605378	0.006551602	0	0	0	0.002256167	0	0.00237801	0	0.000183941	0.00171872
0.005182383	0.005365549	0	0	0	0.000274703	0	0.001437638	0	0.00010073	0.000717347
0.001562515	0.000128553	0	0.007585775	0	0.001805835	0.00259148	0	0	0.00068321	0.000331363
0.002281614	0	0	0.00097432	0	0.005525583	0.000995697	0	0	0.000551823	0.009070617
0.007359179	0.000260166	0	0.001809451	0	0.057426438	0.008796807	0	0	0.046572127	0.001325453
0.010279449	0.000417797	0	0	0	0.046123085	0.006165321	0	0	0.046528331	0.000586258
0.000533839	0.000249454	0	0.004762236	0	0.003697232	0.019673909	0	0	0.000884669	0.012868551
0.005857604	0.000203542	0	0.058886691	0	0.037818948	0.009388002	0	0	0.002934298	0.011863537
0.001187122	5.51E-05	0	0.000914667	0	0.000238676	0	0	0	0.000613137	0.000542562
0.001645394	0.001085047	0	0	0	0.002562394	0	8.74E-05	0	0.000262773	0.000382342
0.007307989	0.001134019	0	0.001640436	0	0	0	0.000364333	0	0.000328466	0.000200275
0.006223247	0.000532576	0	0.000874899	0	0	0.000253369	0.000264634	0	0.000223357	0.000524355
0.005565089	0.000182116	0	0	0	0.021994254	0.003622737	0	0	0.001839411	0.011473912
0.007800388	0.000189768	0	0.013292505	0	0.064285007	0.008436755	2.34E-05	5.01E-05	0.003551814	0.056888899
0.020588149	0.000932008	0	0.008033167	0	0.01700907	0.004978486	0	0	0.004585388	0.012533546
0.012731696	0.001271755	0	0.002038118	0	0.007367445	0.004231713	0	0	0.004852541	0.00540013
0.004392594	0	0	0	0.116234756	0.000526889	0	0.010072079	0.105696621	0.005960567	0.000320439
0	0	0	0	0.004433176	0	0	0.000541576	0.004854681	0.000451094	0
0.000134069	0	0	0	0.005899235	0	0	0.000782823	0.005848985	0.000394159	0.000167502
0.000433897	0	0	0	0.003387102	0	0	0.000569885	0.004652006	0.000731385	0
0.001484511	0	0.000143431	0	0	0	0	0	0	0	0
0.000528964	0.006260828	0.181394351	0	0	0	0	0	0.038112588	0.000109489	0.000211199
0.000687409	0.000676433	0.012154237	0	0	0	0.000293375	0	0.005012053	8.32E-05	0.000207557
0.042161098	0.141357579	0.079308736	0	0.000108885	0.006074989	0	0.572968722	0.137893459	0.006713849	0.015588643
0.346815004	0.580971431	0.404023308	0.024556834	0.010064087	0.099446991	0.012246186	0.231895368	0.27613266	0.087499015	0.061586247
0.060933219	0.038097541	0.135954092	0.003589076	0.010682398	0.009389439	0.007481064	0.040353551	0.136508111	0.030258306	0.008324139
0.00376125	0.000904461	0.005677382	0.000884841	0	0.003526106	0	0.039735662	0.009497149	0.008509464	0.011652338
0.000148695	0	0.001926967	0	8.56E-05	0	0	0.000141548	0.005970591	0.000240875	0.000335005

CC55	CC56	CC57	CC58	CC59	CC60	CC61	CC62	CC63	CC64	CC65
0	0.000222935	0.009160784	0.008461337	0.000300415	8.17E-05	0.001673587	0.007242871	0.000516205	0.0034964	0.046801634
0.001162265	0.00193052	0.00213534	0.000255583	0.001938488	0.000348113	0.001776866	0.006296045	0.017368773	0.000176048	0.008547269
4.84E-05	0.002798543	0.002257951	0.001142509	0.001536582	0.000326867	0.055165	0.001720873	0.000526326	2.35E-05	0.001676932
0.029153484	0.000227678	0.0008742	0.000541634	0.010607894	0.00053933	0.038630992	0.00085007	0.000283407	2.97E-05	0.002052971
0.00207432	0.02591261	0.000226114	0	8.73E-05	0.003888081	0.012412242	0.002667699	0.003163019	0	0.029544484
4.84E-05	0.041242932	0.001285026	0.000869999	0.000633307	0.001673558	0.00556767	0.007256693	0.001057714	6.96E-05	0.012490599
0	0.009979888	0	7.45E-05	4.06E-05	0.000722376	0	0.001803807	0.000334015	1.33E-05	0.001016322
0.208338714	0.042983721	8.12E-05	0.000854766	0.002194247	0.001140765	0.000399032	0	0.000435231	0.000183213	0.002947334
0.339548223	0.362392327	0.002840751	0.005258926	0.002403319	0.003723013	0.010057954	0.521065144	0.039970445	0.000591604	0.029127792
0.044271001	0.016743824	0.001200631	0.000687198	0.002924986	0.00056548	0.003361258	0.014140185	0.006037572	7.68E-05	0.001270403
0	0.000175502	0.002843935	0.001812781	0.009394055	0.004639874	9.15E-05	0.009171078	0.006138788	8.19E-06	0.009553428
0.001111147	0.000241908	0.031440933	0.004204433	0.109588513	0.803747528	0.001992813	0.099741523	0.634709205	0.000352096	0.096245706
0	0	0.000230891	0	0.000608949	0	0	0.000939915	0.000308711	1.43E-05	0.000660609
0	0	0	0	0.000223281	0	0	0.000504513	0	0	0.000487835
0	0	0.000167197	0.000176031	0.002594123	0.000174874	0	0	0.001123505	0	0.002815212
0	0	0.004210171	0.000274202	0.000529786	0.000189583	0.00016196	0	0.000824915	0	0
0.000513872	0.001143134	0.000880569	0.000159105	0.01185218	0.000601435	0.002650042	0.002750632	0.000749003	8.90E-05	0.013842307
0	0	0.000308916	0	8.12E-05	0	0	0	0	0	0
0	0	0.000421973	0	0	0	0	0	0	0	0
6.46E-05	0.001133647	0.000724519	0.001183131	0.005161859	0.000163433	7.75E-05	0.002024963	0.000718638	2.46E-05	0.000518324
0	0.00818218	0.00316559	0.002263014	0.003251788	0.001882753	0	0.002868122	0.009478937	0.000102354	0.019513385
0	0.000222935	0.000337578	0	0.00024358	0.000161799	0	0.000822425	0.000647786	7.16E-06	0.000457345
6.46E-05	0.000227678	0.000789805	0.000377451	0.00059271	0.000405315	9.15E-05	0	0.000445353	1.33E-05	0
0	0	0.000288215	0.000174338	0.000397847	0	0	0	0.00097168	1.33E-05	0.000213428
5.65E-05	0	0.000234075	0	0	0	8.45E-05	0	0	0	0
0	0	0	0	0	0	5.16E-05	0	0	0	0
0	0	0	0	0	0	4.23E-05	0	0	0	0
0	0	0	0	0	0	0	0	0	0	0
0	0	0.000431527	0	0	0	8.92E-05	0	0	0	0
0	0	0.0017691	0	0	0	5.63E-05	0	0	0	0
0	0	0.000160827	0	3.65E-05	0	0	0	0	0	0
0	0	5.41E-05	0	0	0	0	0	0	0	0
0	0	0	0	0	0	0	0	0	0	0
0	0	9.71E-05	0	0	0	0	0	0	0	0
0.086755558	0.00037472	0.000259553	0.014470087	0.01280823	0.01125239	0.000206558	0.00936459	0.030263771	5.63E-05	0.001189097
0.06014453	0.000194475	0.00018312	0.000335136	0.008846001	0	0.000199516	0.001375316	0.009397964	0.000145342	0.004075452
0.00037397	0.002770083	0.031636792	0.003798208	0.055371743	0.001027996	0.023618	0.000739492	0.013325169	0.001685764	0.034839523
0	0	0.002576421	0.00088354	0.001280823	0	0.000589159	0.001064315	0.000409927	1.33E-05	0.000813058
0	0	0.001243625	0.005353712	0.000809902	0.000321964	0	0	0.000253042	2.46E-05	0
0	0	0	0	0	0	0	0.001009026	0	0	0
0	0	0	0	0	0	0	0	0	1.94E-05	0.000528488
0	0	0	8.29E-05	0	0	0	0	0.000106277	0.000195495	0
0	0	0.002909222	0.000724435	0	0	0	0	0	0.000174001	0
0	0	0.002058907	0.004106262	0	0	0	0.000234979	0	5.02E-05	0
0	0	0.00153184	0	0	0	9.39E-05	0	0	0	0
0	0	0	0.000382529	0	0	0	0	0	0	0.006880501
0	0.000137555	0.000832799	0	0	0.000264762	0	0.001029759	0	0.00028352	0
0	0	0.000186305	0	0	0	4.93E-05	0	0.012864633	1.33E-05	0.000274407

0	0	0	0	0	0.000168336	0	0.000269534	0.00243426	7.16E-06	0.015417607
0.005466951	0	0.057160555	0.030031906	0.000318683	8.01E-05	0.004255559	0.001354583	0.002363408	9.21E-05	0.041445617
0	0	0.005328	0.001144202	0	0	0.000359129	0.000587447	0.000308711	0	0.000406529
0.000110308	0	0.005813666	0.000495934	0.009294593	0.00051645	0.002138342	0.001368405	0.000273285	2.05E-05	0
0.00025021	0	0.000640124	8.29E-05	7.71E-05	7.19E-05	0.000251155	0	0	2.05E-05	0
0.000390112	0	0.00012898	0	8.32E-05	0	0	0.000214245	0.00042511	0.000157625	0.000213428
7.26E-05	0	0.011511092	0	0.001818728	0.01288509	0.000237072	0.002411987	0.031630195	0.000226202	0.000233754
6.19E-05	0	0.006770668	0	0	0.000251687	0.000166655	0	0.002823944	2.56E-05	0
0	0	0.000261145	0	0	0.001611453	5.16E-05	0	0.000379562	1.43E-05	0.000294733
0	0	0.000159235	0	0	0	0	0	0	0	0
6.19E-05	0.000184988	0.029875653	0.000333443	0.010758101	0.064593786	0.00062202	0.007325805	0.041114193	0.000233366	0.008730207
0	0	0	0	3.86E-05	0.000186314	4.69E-05	0	0.005521367	1.54E-05	0
0.000234067	0	0.001070059	0.004546339	0	7.35E-05	0	0	0.001720683	1.02E-05	0
0	0	0.006071627	8.97E-05	0.0001157	0	0.000901343	0	0	0	0
5.65E-05	0	0.016569985	0	3.45E-05	9.81E-05	0.000499964	0	0	8.19E-06	0
0	0.026064395	7.80E-05	7.79E-05	0	0.021931129	0	0.029358508	0.016250329	1.54E-05	0.005996301
0	0.041442151	0	7.45E-05	0	0.005568176	0	0.021479813	0.000597178	1.64E-05	0.003211578
0	0.006166281	9.87E-05	0.000258969	0	0.00298266	0	0.011942444	0.000136642	0.000203684	0.006402829
0	0	0	0.000858151	0.000133969	0.002412277	0	0.00279901	0.000247981	1.13E-05	0.000528488
0	0.000194475	0.001514324	0.005961358	0.001928339	0.000866197	0.000288711	0.00085007	0.000323893	0.005572131	0.000772405
0	0.000393693	0.000550953	0.002784337	0.001297062	0.000415121	7.28E-05	0.000221156	0.000455475	0.004808573	0.000254081
0	0.003149547	0	9.48E-05	3.45E-05	0.000187948	5.63E-05	0	0	0.000135107	0
0	0.006128335	0	7.28E-05	0	0.00073545	0	0.00212863	0.000151825	8.19E-06	0.001758237
0	0.061790878	0	0.000754902	0.000231401	8.83E-05	0	0.003165301	0	0	0.008354168
5.65E-05	0.014386408	0	0.000715972	3.65E-05	0	0	0.004056837	0	0	0.004756388
0	0.109147915	0	0	0	0.000794286	4.93E-05	0	0	0	0.000772405
0	0.01960403	0	0.000367295	0	0.00100675	5.63E-05	0.002778277	0	0	0
0	0.00040318	0	0.00016249	0	0	0	0.00030409	0	5.32E-05	0
0.003677816	0.000744697	0.000350317	0.003771126	0.000316654	0	4.23E-05	0.001879829	0.000936254	0.000263049	0.002642438
0.005717161	0.000365234	0.000224521	0.003293811	0.000324773	0.000598166	5.40E-05	0.001313116	0.000693334	0.002890466	0.004878346
0.004998816	0.001062498	8.76E-05	0.003207488	0.000306504	9.81E-05	0	0.002107897	0.00091601	0.00060491	0.004136431
0.026250511	0.02509202	0	0.000184494	0	0.000173239	0	0.02255795	0.002044576	7.16E-06	0.001128118
0.01651385	0.055814329	0	0.00044177	0	0.000589995	0	0.018024244	0.001224721	5.22E-05	0.001534646
0.003618626	0.009875536	0	0.002269784	6.50E-05	0.000616144	0	0.02031183	0.000465596	0.001230291	0.003740065
0.003056327	0.003955906	3.82E-05	0.001880485	0	0.000558942	4.46E-05	0.015425657	0.001310755	0.001121796	0.003902677
0.018674264	0.000189732	0.086794171	0.001128968	0.011139709	0	0.037002002	0.001347672	0.000536448	2.66E-05	0.00602679
0.001713803	0	0.006603472	7.28E-05	0.000284176	0	0.002103133	0	0.000328954	0	0
0.002596264	0	0.00519902	0	0.000560233	0	0.001863714	0	0	0	0.000182938
0.001856396	0	0.003297755	8.12E-05	0.000377548	0	0.001682976	0.000228068	0.000106277	1.94E-05	0.000721589
0.000129141	0	0.000136942	0.010941004	0	0.000447808	0	0.000234979	0.003188324	0	0.036211558
0.033291363	0.000844306	0.070181193	0.007161415	0	0.000485397	8.92E-05	0.00322059	0.013097431	3.79E-05	0.031211253
0.001611567	0	0.009468108	0.024470003	0	0.000480494	0	0.00103667	0.004327011	1.54E-05	0.021728967
0	0.00034626	0.070528326	0.02054485	0.313726525	0.004734666	0.251519256	0.003517769	0.013148039	0.00504501	0.009451796
0.090885366	0.080379843	0.387397831	0.457394572	0.362797675	0.030052135	0.402120972	0.108428822	0.050284419	0.922031105	0.358141757
0.004918103	0.003301332	0.064590456	0.090090639	0.033534828	0.002949973	0.122988703	0.005888288	0.005303751	0.046965983	0.064190906
0	0.009733237	0.03676734	0.265249956	0.003941931	0.00284701	0.011179938	0.005176441	0.002500051	8.29E-05	0.022237128
0	0	0.002694255	0	8.32E-05	0	6.57E-05	0	0	5.12E-06	0

CC66	CC67	CC68	CC69	CC70	CC71	CC72	CC73	CC74	CCR01	CCR02
0.069856296	0.005895478	0.105951369	0	0	2.60E-05	0.00202998	0.000815978	0.000194651	0.000144467	0
0.008600489	0.000846972	0.000335812	0.00543575	0.021256539	0.010228547	0.011044361	0.018145788	0.004030153	0.000591001	0.000832895
0.006163943	0.001818906	0.000328063	0.00185975	0.007139823	0.000584063	0.006453646	0.008749093	0.012293958	0	0.000369857
0.005264495	0.00065675	0.002929317	0.000539557	0.190092696	0.002118624	0.009589542	0.027354678	0.02378277	0	0.000845797
0.008797487	0.007675507	0.014840321	0.000614177	0.020342968	0.000182287	0.001063625	0.000207232	0.006569459	0	0.000160558
0.007589582	0.0080379	0.001276087	0.024337464	0.00254869	0.003372314	0.017997044	0.008515957	0.129411713	0.000423551	0.001680125
0.001474888	0.001188537	0.000335812	0.003799859	0.000139654	0.000215769	0.001664161	0.002389649	0.027392655	0	4.01E-05
0.006638292	0.001230192	0	0.001945849	0.000174568	0.004438137	0.014717357	0.010018392	0.003835503	9.85E-05	2.44E-05
0.077132237	0.030585396	0.0042674	0.018970594	0.004870442	0.002987279	0.010448055	0.009850016	0.208311583	0.007466313	0.000920342
0.006516465	0.001428744	0.001438827	0.001337413	0.00114051	0.000156246	0.00072741	0.001483007	0.018058271	0.000942318	0.000273809
0.001451559	0.000147179	0.000661292	0.007289759	0	0.00025855	0.00089446	0.000453321	0	0.000105067	0.000903139
0.022133689	0.003519792	0.018446429	0.546720469	0.201486154	0.409746416	0.253373256	0.257499223	0.000305248	0.043369625	0.045221463
0	1.00E-04	0	0.000177939	0	2.79E-05	0	0.000777122	0	0	0.000309648
0.001140511	6.53E-05	0	0.000367358	0	0	0.00012053	0.000356181	0.012731922	0.000242967	6.88E-05
0.000523598	0.000180502	0.000694873	0.000195159	0.005557075	0.018570979	0.010407878	0.000667029	0.007082629	0	0.000236536
0.001179392	9.58E-05	0.001301919	0.001412032	0	5.39E-05	0.000109957	0	0.028073932	0.000121484	0.001242891
0.006892315	0.000756721	0	0.001440732	0.003246961	0	0.001452705	0.006540773	0.100634384	0.000797851	0.002333826
4.92E-05	0.000323515	0	0	0	0	0.004098023	0	0.002614512	0	0.001467959
0	0.000105524	0	0	0.000104741	0	0	0	0	0	0
0.003548544	0.000458198	0.001555069	0.001228353	0	2.60E-05	0.000414454	0.000148948	0.00015926	0.000384151	8.31E-05
0.031506615	0.002546469	0.005662312	0.008196674	0	0.014694585	0.031151739	0.010944462	0.0226768	0.000850385	0.000613561
0.002908303	0.000144402	0.000170489	0.000177939	0	0.001043502	0.002909638	0.000738265	0.002411014	0	0
0.001259746	9.58E-05	0.000178239	0.000631396	0	0	0.001843899	0	0	0	9.89E-05
0.000552111	0.000102747	0	0.000361618	0	0	0.000886002	0	0	0	3.30E-05
0	0	0	0	0	0	0	0	0	8.54E-05	0
0	0	0	0	0	0	0	0	0	0	0
0	0	0	0	0.000104741	0	0	0	0	0.000305351	0
0	0	0	0	0	0	0	0	0	0	0
0	0	0	0	0	0	0	0	0	0	1.72E-05
0	0	0	0	0	0	0	0	0	0.000105067	0
0	0	0	0	0	0	0	0	0	0	0
0	0	0	0	0	0	0	0	0	0	0
0	0	0	0	0	0	0	0	0	0	0
0	0	0	0	0	0	0	0	0	0	0
0.001894803	0.001142718	0.005517655	0.045012829	0.052242323	0.002388335	0.005717778	0.009331935	0.021026694	0.072069292	0.005185165
0.003888106	0.001406528	0.001694561	0.013299506	0.041390025	0.000193448	0.02679151	0.023935343	0.076325173	0.0002364	0.001113871
0.001832594	0.006243986	0.029440408	0.023774947	0.017101825	0.253795946	0.263878404	0.285235986	9.29E-05	0.011527803	0.14923441
5.44E-05	0	0	0.000711756	0	0.004882694	0.002186458	0.00501891	0	0.002626671	0.004028287
0.000134788	0.000238818	0	0.002737965	0.002426492	2.79E-05	0.003197219	0.000220184	0	0.000111634	0.003626892
0	0	0.000121409	0	0	0	5.50E-05	0	0	0	0
0.000204774	0.000455421	0.003391704	0.000338658	0	3.72E-05	0.017487434	0	0	0.000131334	0.045627158
0.000671346	0.000108301	0.004864112	0.000350138	0	0	0.004962879	0.01396876	0	0	0
0.002117721	0.000395716	0.013052766	0.000424758	0	2.42E-05	0.009291389	0.035695783	0	0	0
0	0.000230487	0	0	0	0	8.67E-05	0.024026008	0	0	0
0.000189221	0.000108301	0.001299335	0.000338658	0	2.98E-05	0	0	0	0	0
0.000648018	0.000977489	0.002291273	0	0	0	0.007094358	0	0	0	0
0.000202181	4.58E-05	0.001438827	0.005143011	0.00292692	0.001389475	0.0143896	0.001800332	0.000406997	0.0001182	3.73E-05
0.003369691	9.72E-05	0	0.002106568	0	0	0.000247404	0	0	0	0

0	0.000186056	0.000147241	0.001102074	0	0	0	0	0	0	0
0.000435468	0.000924726	0	0.000200899	0.003549545	0.000394336	0.001395611	0.003905036	0	0.018179848	0.008509577
0	0.000104136	0	0	0	0.003039361	0.010788499	0	0	0.002350871	0.010573895
7.78E-05	8.61E-05	0	0.000539557	0.084420988	0.017964595	0.021046243	0.005679463	0.019863214	0.000528618	0.00322693
0.000290312	0	0	0	0.003537907	7.44E-05	5.50E-05	0.000356181	0.00143776	0.001250952	0.004034021
7.00E-05	5.55E-05	0	0	0	5.21E-05	0.008908653	0	0	0	0.012622443
0.003294521	2.64E-05	0.00091186	0.01612357	9.89E-05	0	0.001401955	0	0	0.000144467	0.005643902
6.48E-05	0	0.00015499	0.006394324	0	4.28E-05	0.000198769	0.001036162	0	9.52E-05	0.002167534
0.001638188	0	0	0.000683056	0	0	0	0.000181328	0	0	0
0	0	0	0	0	0	0	0	0	0	0.000235103
0.016667012	0.000266588	0.000769785	0.034893265	0.007419131	0.02134435	0.017597391	0.015723759	4.42E-05	0.002498621	0.004942894
0	0	0	0	0	0	0.000158592	0	0	0.000111634	0
0.000422507	7.36E-05	0	0.000304218	0	0.000223209	0	0.000660553	0	0.00012805	0.000341186
0.000440652	0	0	0	0.000122197	0	4.44E-05	0	0	0	4.59E-05
0.00081391	3.75E-05	0	0	0.000168749	6.88E-05	0.001366008	0	0	0.000219984	0.002111625
0.001226049	0.000131905	0.000860196	0	0	0.007248709	0.002869462	0.005193762	0	0	0.001330338
0.001664109	0.000778936	0.000335812	0.000154979	0.001070683	0	5.92E-05	0.0001619	0	0	3.44E-05
0.005350033	0.006293971	0.001637731	0	0.000209481	0	6.13E-05	0.000356181	0	0	2.87E-05
0.00013738	0.000223545	0.000328063	0	0	0.000133925	0.000818336	0.000654077	4.42E-05	0	0.000104649
0.001456744	0.008589126	0.003190217	0.000470677	0	8.56E-05	0.000133217	0	0	0.000682935	0.000764084
0.001073117	0.007821297	0.003123055	0	0	0.000163687	0.000190311	0.000433893	0	0	0.000658001
0.001332324	0.001655066	0.000271233	0.000143499	0.00011056	5.21E-05	6.34E-05	0	0.00027428	0	0
0.005404467	0.001191314	0.001121097	0.007817836	0.000448057	0	0.001150322	0.000738265	0.040261717	0	0
0.022011861	0.003139349	0	0.011560295	0.003264418	0.000163687	0.001442132	0.001547767	0.046251648	0	0
0.002905711	0.002832495	0	0.008868251	0.000128016	0.000187867	0.000718951	0.000699409	0.020279058	0	0
0.022742825	0.000944165	0	0.000160719	0.001157966	3.16E-05	0.000234716	0.000148948	0.028463233	0	0
0.002190299	0.000709512	0.000333229	0.004878973	0.001210337	5.58E-05	5.71E-05	0.000148948	0.008856604	0	2.29E-05
0.000311048	0.000345731	0.000901527	0	0.000122197	0	0	0	5.31E-05	0	0
0.00205292	0.00848499	0.002513426	0.010578761	0.000832107	0.000111604	0.000549786	0.000420941	0.000482203	9.85E-05	0
0.000730964	0.007383927	0.005724308	0.001308713	0.001664213	5.21E-05	0.000211456	0.000304373	0.000725516	0.001030968	6.59E-05
0.000458796	0.004838846	0.005295502	0.001073374	0.00081465	2.60E-05	0.000277008	0.000770646	0.000743212	0.001451236	0
0.001215681	0.00461669	0.000330646	0.006543563	0	6.70E-05	0	0	0.003609885	0	0
0.001658925	0.001466233	0.000968689	0.001216873	0.000192025	0	0	0.000569889	0.00684374	0	2.72E-05
0.010523806	0.017254618	0.003693935	0.002692045	0.000128016	3.16E-05	0.000181852	0	0.001486423	0.0001379	0
0.009769513	0.012493526	0.003557027	0.002163968	0	0	0	0.00012952	0.002809163	0.0002167	0
0.000111459	0	0	0.000373098	0.02314769	7.81E-05	0.010289462	0.019758315	6.64E-05	0	0
0.000217734	0	0.000149824	0	0.001902789	0	0.000881773	0.002622785	0	0	0
7.78E-05	0	0	0	0.00102995	0	0.000850054	0.000945498	0	0	0
0	5.69E-05	0	0	0.00102995	0	0.000524412	0.000744741	4.87E-05	0	0
0.000114051	0.000230487	0.001247672	0.006164726	0.001559472	0	0	0	0	0.007742113	0
0.000596176	0.001101063	0	0.00890269	0.032231035	0.001928897	0.002385227	0	0	0.038484017	0.004336501
7.00E-05	0.000294357	0.000663875	0.004161477	0.009141534	0.000159966	0.000186082	0	0	0.131474745	0.001981172
0.007841013	0.013344663	0.008555465	0.004620674	0	0.053852864	0.027755751	0.046141592	5.31E-05	0.029622285	0.56326059
0.549031603	0.759634997	0.693736584	0.127869266	0.112636963	0.070764658	0.082888747	0.058057455	0.065561877	0.541984056	0.061876494
0.036369339	0.052659422	0.035813092	0.006491904	0.041326017	0.012754528	0.014142196	0.005226142	0.041288056	0.070595072	0.013244606
0.000699859	0.000265199	0.000178239	0.000154979	0.090629782	0.0773456	0.04933275	0.061049373	0.00203056	0.008116414	0.030931796
5.18E-05	0	0	0.000109059	0.000401506	0	0	0.000744741	0	0	0.000246571

CCR03	CCR04	CCR05	CCR06	CCR07	CCR08	CCR09	CCR10	CCR11	CCR12	CCR13
0	0.000291731	0.000131491	0	0.000124633	0.00156589	0	0.000166332	0.000232001	0	0.000630553
0.000268064	0.000369244	0.000438302	0.003840952	0.002675953	0.000576429	0.0014126	0.002339303	5.46E-05	0.000121552	0.000112811
0	0	0.000124916	0	0.001660557	0.000319986	0.000463333	0.000897764	0.000148877	0.000203344	0.00033962
0.001205626	0.001639049	0.002982648	0.000220157	0.00243585	0.000721671	0.003207814	0.001767805	4.71E-05	0.000537328	0.000970173
0	3.24E-05	7.23E-05	0	0.000218109	0.000120279	0	0	0	0	3.09E-05
0.000172987	0.000658156	0.008564428	0.006388483	0.003861804	0.00202431	0.002387698	0.00118138	5.33E-05	0.000713409	0.000281433
0	0.000128249	0.000440494	0.001026089	0.000111804	7.72E-05	0.000681277	0.00021751	0	5.45E-05	0
3.83E-05	0	7.01E-05	0	0.011206012	0	0.000439117	0.001584414	0.000155081	9.09E-05	0.000548617
0.000454256	9.30E-05	0.000190662	0.000267333	0.034065249	0.013505233	0.014093716	0.010962953	0.010132352	0.001246193	0.002614835
0.000221846	0	4.82E-05	0.000104181	0.002740103	0.001901762	0.00070065	0.005075244	0.001624005	0.00013064	0.000429868
0.000422563	0.000396021	0.001571314	0	0.000764296	0	0.001175284	0.003245597	9.55E-05	0	0.000148435
0.156600637	0.091654241	0.208572756	0.027653284	0.031719208	0.03249562	0.06570287	0.081828708	0.011464805	0.009057335	0.016754184
0.000426525	0.000140933	0	0	0	0	0.000489163	0.000656796	0	2.61E-05	6.17E-05
7.00E-05	0	0	0	0	0	7.43E-05	9.60E-05	3.47E-05	0	0
5.81E-05	6.20E-05	0.000883179	0	0.000384897	0.000197438	0.000226016	0.00059922	0.000143915	3.29E-05	0.00015081
0.001571407	0.000412933	0.001071649	0	0.00973607	8.62E-05	0.011150664	0.009800765	0.000233241	0.000189712	0.00216003
0.0026397	0.001009079	0.002193703	0	0.001873167	0.003159013	0.001927594	0.011777551	0.001414336	0.000163584	0.00085855
0.000116205	2.82E-05	8.33E-05	0	0.00022544	5.90E-05	0.000431045	0.000294279	0	0	0.000225622
0	0	0	0	0.000164956	0	0	0	0	0	0
0.000471422	0.000823048	0.000392281	0	0.001402126	0	4.84E-05	0.00070371	0	0	3.56E-05
0.00111187	0.00316676	0.006129659	0	0.000227273	0.000397146	0.003874561	0.003290379	0.00084612	9.32E-05	0.001449916
0.000109602	0.000259316	0.000495282	4.72E-05	0	0	0.0002018	0.000351855	4.96E-05	0	0.000108061
6.87E-05	0.00098653	0.001189991	0	0	0	0.000456875	0.00046061	0	0	0.000142498
0	0.000262135	0.000309003	0	0	0	0.000305122	0.000313471	0	0	7.12E-05
0.000145256	0	0	8.65E-05	0	0.001007616	0.00041813	0.000567233	3.97E-05	0	0
0	0	0	0	0	0.000174744	0	0.000183391	0	0	0
0	0	0	0	0	0.003621971	9.04E-05	0.000739962	4.47E-05	3.52E-05	9.26E-05
0	0	0	2.56E-05	4.95E-05	6.58E-05	0	0.000140742	0	0	0
0	0	4.60E-05	9.44E-05	7.88E-05	0.00034495	0.000182427	0.000467008	2.61E-05	3.52E-05	7.72E-05
0	4.37E-05	0	0.000151358	9.71E-05	0.00022921	5.33E-05	0.000332663	0	0	0
0	0	0	0.000104181	3.12E-05	0.000108931	0	0.000330531	5.33E-05	0	0
0	0	0	0	6.60E-05	0.000124817	0	0.000219643	5.58E-05	0	0
0	0	0	2.75E-05	3.48E-05	0	3.71E-05	0.000100225	0	0	0
0	0	8.11E-05	0	0	0	0	3.63E-05	0	0	0
0.003682903	0	0	0.01367725	7.70E-05	5.90E-05	0.004173225	0.00884116	0.00117241	8.63E-05	0.001156608
0.001369369	0.002594573	5.92E-05	0.000133667	0.009409824	0.000372183	0.002500706	0.004934502	0.001544604	0.00016472	0.00049993
0.014497883	0.778112571	0.413075875	0.165062666	0.027824413	0.483782827	0.083106106	0.135645484	0.000238204	0.000644113	0.023496707
0.001832868	0.000489037	0.007045711	0.002968187	0.00065066	0.003510771	0.001787141	0.001998111	0.003025934	0.000695233	0.000432243
0.006959089	0	0	0	0	0	0	1.71E-05	0.007183338	0	6.89E-05
0.000137333	0	0	0	0.000175953	0	0	0.000279352	0	0	7.12E-05
0	0.000563731	0	0	0	0.004053159	0	0	0	0	0
0	0	0	0	0	0	0.002489405	0.005678729	0	0	0
0	0	0	0	0	0	0.000464947	0.008868882	0	0	4.39E-05
0	0	0	0	0	0	0.000305122	0	0	0	0
0	0	0	0.000375446	0	0.00233068	0.001373855	0.002665569	0	0	0
0	0	0	4.13E-05	0	7.04E-05	0	0	0	0	0
0	0	0	3.34E-05	0.001006232	0.001686168	0	0.000835922	0.000637692	0	0.000467868
0.00036182	0	0	0	0.001189516	0	5.00E-05	0.000198318	9.93E-06	0	0.000118748

0	0	0	0	8.61E-05	0	0	0	0	0	0.000111623
0.005214695	0.000245223	0.000674986	0	0.014620601	0.006254482	0.05732736	0.067270436	0.02717634	0.003670419	0.000942861
0.003214122	0	0	0	0.000931085	0.000658128	0.004284619	0.000835922	0.002944052	0.000293088	0.000396619
0.003144135	0.000722985	0.002932243	0.000605432	0.002631965	0.000853297	0.008088146	0.004964356	0.000168728	0.000710001	0.001130483
0.00031032	0.001303628	0.000756072	0	0.000843109	0.000131626	0.000305122	0.000290014	9.30E-05	0.002466258	0.00014606
0.000269384	0	0.000153406	0	0.00277676	0	0.006801469	0.010611098	0	0.00012496	0.000619866
0.001521228	0	0.000547878	0	0.003896628	0.000410762	0	0.021128367	0	0.002960418	0.002092343
0.001897573	0	0.000127108	0.000243745	0.002355205	0.000696708	0.023980304	0.000390239	0	0.00046008	0.003875941
0.003671019	0	0	0	0.001673387	0	0	0.006408028	0	0.000349888	0.003056579
6.47E-05	0	0	0	0	0	0	0.001889355	0	1.00E-04	3.44E-05
0.010986646	0.004249124	0.009918783	0.002038417	0.010949413	0.005718902	0.036411188	0.015692739	0.000496258	0.002757074	0.012323689
0	0	0	0	0.000106305	0	0	0	0	0	0.000203059
0.000690627	0	0	0	0.018040689	7.26E-05	0	0.000371047	0	0.001817601	0.001680287
3.04E-05	0	0.001005904	0	0.001497434	0.000138434	0.000224402	0.000162067	0	0	0.000254121
0.001129036	8.74E-05	0.000425153	0	0.00192632	0.000388068	0.007531178	0.000769816	2.48E-05	0.001101921	0.001540164
0.003791185	2.11E-05	0.000918243	4.91E-05	0	0.000245096	0.000301893	0.000567233	0	0	3.68E-05
6.60E-05	6.76E-05	0.000221343	0	0	7.49E-05	0	0	0	0	0
0.000118846	0.000109928	0.000149023	0	3.85E-05	0	0.000255075	0.000168464	0	0	0
0.000216564	5.78E-05	0.000387898	0	0.000540689	6.58E-05	0.000182427	0.000100225	0	0.000105648	0
0.000998306	0.000287503	0.000499665	4.72E-05	0	0.000978114	0.001391613	0.001136599	1.49E-05	0.004161171	8.43E-05
0.000228448	0.000129658	0.000326535	3.54E-05	0	0.000780676	0.000747467	0.00083379	0	0.003114914	3.68E-05
0	0	0	3.15E-05	3.85E-05	0	0	0	3.10E-05	0	0
5.94E-05	3.81E-05	0	0	2.93E-05	0	0	9.38E-05	0	2.27E-05	0
0	0.000102881	0.000282705	0	0.000260264	0	0	0	0	0	0
0	7.05E-05	6.79E-05	0	0.000185117	0	4.04E-05	3.20E-05	0	0	0
0	0	0	0	2.57E-05	0	0	0	0	0	0
0	9.02E-05	8.77E-05	0	5.68E-05	0	0	2.99E-05	0	0	0
0	0	0	0	4.22E-05	0	0	0	7.57E-05	0	0
0	0.000432664	0.000587325	0.000163152	0.003480572	0.001084776	0.002547524	0.001821117	0.002163686	0	0
0.001323151	0.000139523	0.001196565	7.67E-05	0.006851173	0.000676283	0.000397143	0.000882837	0.000609157	2.84E-05	0.000191185
0.000805511	0.00029455	0.001339014	0.00010025	0.006013563	0.001016694	0.000429431	0.000995857	0.000547125	0	0.00017931
0	0	0	0	0.000826613	0.000254173	0.00022763	0.000142875	2.23E-05	0	0
0	7.33E-05	0	0	0.000137463	0	3.71E-05	2.99E-05	0.000106696	0	0
0	0	0	0	0.001995968	0.000138434	0.000287363	0.000424359	0.00099996	3.29E-05	9.02E-05
0	3.38E-05	0.0002323	0	0.002089443	0	0.000332566	0.000159934	0.000586825	3.41E-05	8.07E-05
0	0	0	0	0	0	0	0	0	0	0
0	0	0	0	0	0	0	0	0	0	0
0	0	0	0	0	0	0	0	0	0	0
0	0	0	0	0	0	0	0	0	0	0
0.00088078	0	0	0.001291456	0	0	0.003267547	0.000876439	0.000847361	3.41E-05	0
0.171889505	0.000467897	0.003534909	0.341310091	0.0025	5.45E-05	0.177858498	0.044425442	0.169676886	0.003719267	0.004797427
0.061677074	0.001282489	0.012787472	0.028195814	0.006427786	5.90E-05	0.029168987	0.015065797	0.041226651	0.003854451	0.005532479
0.027667332	0.01067566	0.046254049	0.011888475	0.000679985	0.133057525	0.064856924	0.048963307	6.08E-05	0.199586268	0.008768367
0.362697961	0.085743519	0.180666044	0.329669293	0.709215543	0.225855793	0.302790491	0.38405094	0.616621921	0.702588151	0.847279122
0.135604523	0.005562618	0.062813112	0.021439748	0.037162757	0.057547589	0.056445897	0.053609927	0.09467242	0.044994723	0.048831636
0.004225633	0.003216087	0.014648065	0.040485289	0.012426686	0.003599277	0.006042701	0.00393438	7.20E-05	0.006171893	0.001031922
0.000591589	0.000277638	0.000195045	0	0.000351906	0.000437995	0.000955725	0.000179126	0	0.000418048	0

CCR14	CCR15	CCR16	CCR17	CCR18	CCR19	CCR20	CCR21	H002	H003	H004
0.000911792	0.000196108	0.000564359	0.000264667	0.002969023	0	0	0	0.007820432	0.002775576	0.000638766
0.000788974	0.002952319	0.002042164	0.017783584	0.007646137	0.001207165	0.006828542	5.77E-05	0.007055359	0.003512686	0.007219122
0	0.005706747	0.002557069	0	0	0.014712318	0	0	0.007581706	0.001469327	6.78E-05
0.000690185	0.004039828	0.004509052	0.002103763	0.001622605	0.004209986	0.00420932	5.02E-05	0.017300441	0.005117367	0.006020098
3.47E-05	7.49E-05	0.000744721	0	0	0.043337206	0.000256272	0	0.000894503	0.000345724	8.56E-05
0.000381805	0.001640177	0.001015264	0.003318517	0.006567688	0.012154638	0.000377346	0.000104175	0.008890385	0.009455224	0.005249297
3.07E-05	0.000454614	0.000142544	6.11E-05	9.37E-05	0.003613949	6.26E-05	0	0.00021284	0.002100436	0.001273963
6.01E-05	0.006172058	0.000337452	0	0	0.003779934	0	0	0.00257709	0.000957264	0.00074939
0.000287021	0.018266582	0.014885688	0	0	0.075417603	0	0	0.024329914	0.01598484	0.008435988
5.74E-05	0.005000758	0.002045073	0	0	0.002006911	0	0	0.003350792	0.001293203	0.002936894
0.000501953	0.000106968	0.002402888	0.009483899	0.000175906	0.001048724	0.002090535	0.000117197	0.004929835	0.000719171	0.003443624
0.026658247	0.039103964	0.155917185	0.179705473	0.058775467	0	0.076427607	0.087315717	0.107239724	0.089035332	0.221876472
0.000121483	0.000183629	0.000660358	0	0.0002729	0	0.00136813	0	0.000747816	0.000720802	0.000717273
0	0	0	0.001034916	7.23E-05	0	0.000159413	0	0.003416945	0.00046314	0.007861456
9.48E-05	0.000927057	0.000578904	0.001204574	0.000920627	0	0.00011502	0	0.006293161	0.003489855	0.003586365
6.41E-05	0.001600956	0.001070536	0.011146551	0.000984742	0.000611127	0.005652112	0	0.044595733	0.003297424	0.013574661
0.000304376	0.003383757	0.001684349	0.124977096	0.003412896	0	0.023417621	0.001343118	0.204404062	0.098356833	0.140000285
0.000256316	8.56E-05	0	0.000766856	0	0	0.000302684	0	0.015419395	0.018372293	0.001091968
0	0.00017828	0	0	0	0	0	0	0.000854236	6.52E-05	0
0.000122818	0.001305011	0.000101817	0.000373248	0	0.000626217	0.002306449	5.77E-05	0.003195476	0.00023157	0.000895699
0.001596638	0.004892007	0.005768677	0.005714092	0.000236733	0.001056269	0.008428729	0	0.033318089	0.001024126	0.008107684
0.000144178	0.000183629	0.000395633	0	0	0	0.000468151	0	0.002643243	0	0.00055669
0.000269666	8.91E-05	0.000991991	0	0	0	0	0	0.001351822	0.000205477	0.000371126
0.000204252	0.000201457	0.000389815	0	0	0	0	0	0.001121724	7.18E-05	0.000356852
7.74E-05	0.00096093	0.000293816	0	0	0	0	0	0.000422804	0.00200259	0.000399675
0	0.00017828	0	0	0	0	0	0	0.007233684	0.012075877	0.002212484
0	0.002916663	8.44E-05	0	0	0	0	0	0.000652901	0.000919756	0.000246228
6.27E-05	0	0	0	0	0	0	0	0.000480328	0.00170742	0.000110624
8.68E-05	0	0.000453814	8.14E-05	6.41E-05	0	0	0	0.003066047	0.007858697	0.000681588
7.48E-05	0	5.82E-05	0.000220556	0	0	0	0	0.001768873	0.005547891	0.000324736
6.81E-05	0	0.000142544	6.11E-05	0	0.001637217	0	0	0.004176266	0.014047482	0.000738684
0	0	7.85E-05	6.45E-05	0	0.000565858	0	0	0.001340317	0.005337521	0.000271208
0	0	0.000162908	0	0	0	0	0	0.001231021	0.003471916	0.000171289
0	0	9.31E-05	0	0	0	0	0	0.000514843	0.001872128	0.000235523
0.013102844	0.007254219	0.015682772	0.067954939	0.010865044	0.012184817	0	0.007835477	0.038411868	0.04160429	0.120237806
0.002321533	0.000360126	0.002810157	0.00264667	0.005055229	0.010404249	0.002508238	0.018463227	0.019707834	0.024011507	0.064797237
0.312128041	0.069955341	0.133197383	0.227128363	0.065627564	0	0.012153755	0.149242403	0.103279174	0.082781317	0.215224746
0.00180623	0.000449266	0.007176665	0.006341828	0.003483587	0	0.001816102	0.001371023	0.002864711	0.000908341	0.010063234
2.40E-05	0.000281683	0.001291625	0.000515761	0	0	0.001640545	0.025855959	0.000977914	0.000989879	0.000110624
0	0.000180063	0.000148362	0.000373248	0.000243309	0	0	0.000355312	0.00093477	0.000270708	0.000642334
0	0	0.001419624	0	0	0	0.004013584	0.000158123	0.004391982	0.091758724	0.000877857
0	0	0	0	0	0	0.000431829	0.0036573	0.002395888	0.072168244	0.000499593
7.88E-05	0	0	0	0	0	0.00015336	0.001863995	0.002277963	0.009851502	0.000128467
3.87E-05	0	7.56E-05	0.017400156	0	0	0	0.019951447	0.003949045	0.006924264	0.000453202
0.002531125	0	0.000136726	0	0.00246761	0	0.000793031	0.000306945	0.007095626	0.002281452	0.000517436
0	0	0.000232725	0	0	0	0	0	0.000120801	0	0.000674451
3.20E-05	9.63E-05	0.000485814	0.002354857	0	0	0	0.072480025	0.00404396	0.000456617	0.02024423
0.00071822	0.000103402	0	0.00318279	0	0	0	0.000961762	0.007918223	0	0.020640336

2.94E-05	0.000912794	0.001518532	0.009239591	0	0	0	0	0.021830482	0.000246247	0.004539161
0.000408504	0.378834583	0.001844348	6.79E-05	0	0	0.000421739	0	0.005162808	0.040399148	0.002776311
0.000305711	0.003562037	0.003066155	0.001326728	0.001953045	0	0.000149324	0.007749905	0.000543605	0.000120677	0.000139172
0.001579283	0.004107574	0.00274034	0.008632215	0.000697046	0.015972296	0.005680362	0.000773874	0.019664691	0.001751451	0.005188632
0	0.002492356	0.001623259	0.000251094	0.000151246	0.000550769	0.004473664	0.000645515	0.010012109	0.00033757	0.002622864
0	0	0.001178172	0	0.002607348	0	0.001154233	0	0.011263263	0.002261883	0.001248983
0.000153523	0.001934339	0.01615695	0.02610702	0.00085158	0	0.001640545	0	0.021401925	0.001079572	0.018449263
0	0.000247809	0.002079982	0.003613722	0.001325045	0.000241433	0.00011502	5.39E-05	0.002974008	0.002638591	0.003318726
0.046984677	0.001800629	0	0	5.92E-05	0	0.005819597	0	0.013690789	0	0.001391724
0	0	0	0	0	0	0.000119056	0	0.0010642	0	0.00021768
0.004087714	0.023409964	0.006862486	0.011489261	0.010001956	0	0.060482235	0.002929933	0.029023898	0.006444816	0.014556004
0	0.000561582	0	0.000156086	0.00028934	0	0	6.14E-05	0.001800511	0	0.000224817
0	0.00049027	0	0.000641308	0.000965015	0	0	0.007093228	0.001130353	0.00156065	0.000149878
3.20E-05	0.000565148	0.000401451	0	0.000106859	0	0.000306719	0	0.008136816	0.00029517	0.000178426
0.002368257	0.007081287	0.003156336	0.003518713	0.001846186	0	0.000278469	0.000146962	0.003402564	0.000242985	0.00021768
2.14E-05	0	0.001274171	0.000125547	0.000129874	0	0.006376534	0.006875576	0.000158192	0.000138616	0
0	0	0	0	0	0	0.000306719	0	7.48E-05	0	0
8.28E-05	0	0	0	0	0	0.000274433	0.000113477	0	0	0
1.87E-05	0	0	0	5.75E-05	0	0	0.000468789	0	0	0
0.00329874	0.000263855	8.73E-05	0	0.000716774	0	0.001150198	0.000872469	0	0	0
0.001931718	0.000354777	0	0	0.000484973	0	0.000744602	0.000917115	0	0	0
0	0	0	0	4.77E-05	0.000414963	5.65E-05	0	0	0	0
0	0	0	0	0	0	5.65E-05	0.000119058	0	0	0
0	0	0	0	6.90E-05	0.245054398	0	0	0	0	0
0	0	0	0	0	0.001478777	0	0	0	0	0
0	0	0	0	5.43E-05	0.443919663	0	0	0	0	0
0	0	0	0	0.00016111	0.012682772	6.05E-05	5.77E-05	0	0	0
0	0	0	0	0	0	0	0	0	0	0
9.75E-05	0.001725752	0	0	0.000593476	0	0.000213896	0	0	0	0
0.000847713	0.000171149	9.89E-05	5.77E-05	0.000231801	0	0.000159413	0.000783176	0	0	0
0.000865068	0	0.000247271	0	0.000212073	0	0.000106948	9.86E-05	0	0	0
0	0.000483139	6.40E-05	0	0	0	0	0	0	0	0
0	0.000203239	0.000203635	0	0	0.001825836	0	0	0	0	0
0	0.000197891	0	0	0	0	0	0	0	0	0
0	0.00019076	0.00034327	0	0	0	0	0	0	0	0
0	0	0	0	0	0.000203709	0	0	0.000293374	0.000998033	0.000189132
0	0	0	0	0	0	0	0	0	0	0
0	0	0	0	0	0	0	0	0	0	0
0	0	0	0	0	0	0	0	0	0	0
0.000829024	0.000106968	0.011208629	0.000206983	6.58E-05	0	0	0.002464864	0	0	0
0.033375875	0.014200012	0.231483071	0.019269791	0.002918059	0.008027644	0.081155525	0.159643199	0.002441908	0.006749771	0.005117262
0.047670858	0.035686334	0.065133977	0.039591463	0.005188392	0	0.018477824	0.077229307	0.001682587	0.001482373	0.003361548
0.192811391	0.142420866	0.031743723	0.001106172	0.069145674	0	0.188678826	0.052779716	0.002096762	0.022884642	0.002176799
0.240904103	0.077380708	0.064208894	0.167663126	0.680968171	0.008186084	0.362532211	0.256358884	0.089205848	0.241866518	0.033151577
0.049870907	0.080004992	0.170654511	0.015873231	0.041485761	0.001063814	0.077034992	0.026648436	0.018491194	0.019319772	0.004738998
0.004473523	0.038954209	0.018251477	0.004797937	0.004973031	0.071803655	0.02276786	0.003493596	0.005599993	0.001268742	0.000424654
0.000218937	0.002173235	0.001538896	0	8.55E-05	0	0.003224589	7.26E-05	5.18E-05	0	0

H005	H006	H008	H009	H010	H011	H013	H014	H015	H016	H017
0.001944678	0.003367866	0.08751093	0.00331221	0.026536926	0.021913866	0.00361628	0.039506901	0.022294863	0.026502718	0.006979625
0.057907098	0.004597869	0.03695242	0.007353255	0.006546881	0.016299256	0.009857383	0.019145789	0.005054275	0.020061648	0.011543057
0.009251214	0.002789472	0.068099487	0.001989922	0.002658573	0.013671494	0.006774964	0.007975551	0.003578098	0.009704483	0.002253057
0.002631035	0.01708826	0.039641422	0.010353902	0.010402007	0.014151863	0.004598205	0.135012444	0.012906281	0.034568348	0.001481462
0.004410208	0.000926163	0.012682362	0.00294872	0.000778833	0	0.003085596	0.078611673	0.00543842	0.072080914	0.001759236
0.010322125	0.004865102	0.019529786	0.001923159	0.007458448	0.018277806	0.013311598	0.024284099	0.005638948	0.003773171	0.004514931
0.000547625	0.001526522	0.000959888	0.000268908	0.000769073	0.000630187	0.001391855	0.001510216	0.00059192	0.000619334	0.000154319
0.036099449	0.00055643	0.016998573	0.004727225	0.002047608	0.001015434	0.001124924	0.003342135	0.001236993	0.005550183	0.001962055
0.056595232	0.022140059	0.037882723	0.00888696	0.028836338	0.024101686	0.027414455	0.03676349	0.006723733	0.040056598	0.014453072
0.001662347	0.001391075	0.008800074	0.001060797	0.002170582	0.020068488	0.001849451	0.00300256	0.005399764	0.009313826	0.005312981
0.005778054	0.008591719	0.002317539	0.007955981	0.001458116	0.004801313	0.002243492	0.003485115	0.00267693	0.015459522	0.003743337
0.361907196	0.104758941	0.053582817	0.303360243	0.038607861	0.022693872	0.281342155	0.06001546	0.288444022	0.13703949	0.169711159
0.001168267	0.001947505	0.000601574	0.000580471	0.000236187	0.000813298	0.001728696	0.0007283	0.001386785	0.001219611	0.001097869
0.001623404	0.000120804	0.000302431	0.006479766	4.68E-05	0.000145062	0.004413896	0.004637883	0.001611473	0.003215771	0.003928519
0.002752729	0.002635721	0.004132123	0.006196021	0.002238901	0.006075955	0.011601968	0.010321301	0.006846949	0.024882922	0.004960252
0.031083203	0.031130798	0.001574612	0.048668718	0.011202311	0.001688426	0.040500432	0.00365937	0.014123946	0.02974231	0.009122454
0.107611989	0.02224622	0.191155877	0.102804622	0.200808112	0.214579915	0.181967536	0.172030615	0.123278901	0.161064873	0.074231822
0.007457437	0.005816891	0.091577307	0.002056686	0.013665687	0.005583696	0.001306056	0.012885988	0.005651028	0.00263455	0.001653417
0.001102552	0.001248307	0.000644309	0.001034834	0.00029865	0	0.001379144	0.0018766	0.000649904	0.000190564	0
0.007893104	0.001054289	0.013122859	0.00412079	0.001198505	0.000741956	0.008840502	0.020781112	0.002215474	0.003696946	0.001300688
0.028444866	0.016110847	0.067688575	0.024554122	0.018449947	0.001074885	0.008633949	0.152630144	0.011908472	0.084967818	0.012486607
0.00188383	0.001500897	0.006166955	0.002269958	0.001700159	0	0.000263753	0.010991515	0.000674064	0.00633626	0.000727504
0.002297592	0.000904199	0.00272845	0.001105306	0.003128995	0.000299636	0.004725315	0.003297455	0.005049443	0.004140008	0.000599639
0.000666886	0.000765091	0.001554888	0.00076778	0.001665024	0	0.001633364	0.001845323	0.001770929	0.002062858	0.000634912
5.60E-05	0.001087235	0.000410911	0.002095631	0.007128566	0.003876246	0.000130288	0	4.35E-05	0	0
0.001134192	0.021104074	0.002738312	0.007017583	0.062111438	0.009062805	0	0	0.000246432	0.000133395	0.000599639
0	0.000893217	3.94E-05	0.001175779	0.004811587	0.000242563	0	0	0	0	0
0	0.001233664	0.000210387	0.001667232	0.004497321	0.005987967	0	0.000192128	5.56E-05	0	0
0.000104657	0.007943771	0.000578563	0.012775933	0.023905681	0.034753038	0	0.00056298	0.000304416	0.00080037	0.000171955
0	0.004777245	0.000818535	0.007711181	0.015264344	0.022344296	0	0.000612129	6.52E-05	0.000157215	0.000158728
0.000216616	0.008749131	0.000578563	0.019220462	0.036987732	0.054947564	0	0.000920428	6.28E-05	0.000171508	0.000163137
4.87E-05	0.00399385	0.00054569	0.007056528	0.013402173	0.017921096	0	0	6.52E-05	0	0
4.87E-05	0.004850459	0.000539115	0.006642966	0.01154976	0.019714157	0	0.00021	0	0	0.000114637
0.000104657	0.001200718	0.000187375	0.003336319	0.005807088	0.006618154	0	0	0	0	0
0.043561754	0.011242084	0.003500963	0.069725176	0.021688252	0.006784619	0.023229357	0.004754054	0.105170968	0.002925161	0.074024594
0.001506578	0.010422081	0.040499405	0.054658885	0.027825221	0.026018406	0.013362442	0.01712621	0.041818573	0.019842499	0.047151052
0.010577684	0.090518725	0.035197007	0.037741763	0.077112267	0.027345366	0.060755415	0.046941812	0.104260136	0.026355031	0.16468918
0.004154649	0.002968847	0.001117678	0.002592648	0.000681235	0.001127203	0.000368619	0.00084447	0.001514833	0	0.002067874
0.000253125	0.008514844	0.000355027	0.001146106	0.001823133	0.006325652	0.000908837	0.000196596	0.000251264	0.000524052	0.002649877
0.000160637	0.004345279	4.27E-05	0.005331805	0	0.002537395	0.000406752	0	0	0	0
0	0	0.009529852	0.000204	0	0.015497848	0.004642694	0	0.000599168	0.001524514	0.000458548
7.06E-05	0.028334004	0.001676518	0.003328901	0.002449713	0.003714537	0.003247661	0	0.001727441	0.000138159	0.000573185
0	0.03534063	0.00012163	0.00280592	0.000238139	0.004784666	0.000133466	0.000370852	0.000123216	0.000176272	0.000308638
0.000116827	0.008588059	0.000887568	0.00070658	0	0.002237759	0.000517973	0.000160851	0.001217665	0.001919934	0.007821766
3.89E-05	0.011732621	0.000371464	0.002970975	0.000696851	0.00058976	0.001283811	0.001268939	0.000613664	0.002443986	0.005842074
0	0.003247062	0.002974997	0.005546932	0.007083671	0.000485125	0.000934259	0.02414112	0.021840655	0.016217015	0.010621553
0.001781607	0.013420215	0.001252457	0.024368668	0.000316218	0.009371953	0.010267312	0.004092776	0.021449263	0	0.012517471
0.001343507	0.016623348	0.00066732	0.019422607	0.00258635	0.000242563	0.002392846	0.001872132	0.018975277	0.000171508	0.010528961

0.0027722	0.01117253	0.000348453	0.00955274	0.000569973	0.000513662	0.007280227	0.00720257	0.006851781	0	0.000987641
0.000586567	0.017622726	0.000332016	0.00262603	0.001944154	0.043998954	0.00193525	0.000795321	0.008040454	0.002172432	0.002314784
0.000226352	0.002573489	6.90E-05	0.000276327	5.08E-05	0.000834701	0	0.000178724	0.000504944	0.00016198	0.000850959
0.051476762	0.01425486	0.000542403	0.023671361	0.003374943	0.005835771	0.00747407	0.00035298	0.014324474	0.003901802	0.002332421
0.001635574	0.001076253	0.00179486	0.000903161	0.001460068	0.010515802	0.002262558	0	0.001287729	0.00053358	0.00477066
0.002146691	0.038071531	0	0.000721416	0	0.002808494	0.002793243	0	8.46E-05	0.001762719	0.006939943
0.005902182	0.061957755	0.00073964	0.007399618	0.004091313	0.007750113	0.04886745	0	0.000968817	0.01104796	0.033482802
0.002526377	0.015137094	0.000124917	0.006385184	0.002799114	0.002116478	0.007763245	0.000772981	0.001314305	0.001900878	0.009810276
0.00360459	0.042328953	0	0.001231415	0	0.004794178	0.011265126	0.001233194	0.000442128	0.01166253	0.037063002
0	0.001500897	0	5.19E-05	0	0.001008299	0.000260576	0	0	0.000309667	0.001269824
0.026222725	0.04716843	0.005798778	0.021139912	0.041988659	0.022674847	0.028399558	0.005187459	0.020183278	0.025306927	0.054624498
0.000304236	0.001025003	0.000128204	4.08E-05	0.000341593	0	0.000937436	0.000361916	0	0.00082419	0.001031732
4.14E-05	0.006380642	0.000381326	0.000139091	0.001087243	0.010092507	0	0.000138511	0.005677604	0.000352544	0.030021649
0.00035048	0.019204159	0	0.00028189	0.001356614	0.001600438	0.00209096	0	0.000297168	0.000338251	0.005753892
0.000693659	0.006765018	0.00050953	0	0.002771786	0.001881049	0.001903473	0.000603193	0.00027784	0.00335393	0.006666578
0.000404026	0.000230626	5.26E-05	4.08E-05	0	0	0.000680039	0.001134896	0.000584672	0.00244875	0.000145501
0.000153335	0	0	0	0	0	0	0.001907877	0.000188448	0.000967113	0
0.000170372	0	0	0	0	0	0	0	0.000173952	0.000147687	0.000114637
0.000104657	0	0	0	0	0	0.000149354	0	0	0.000171508	0
5.60E-05	0	0	0	0	0	0	0	6.52E-05	0	0
0	0	0	0	0	0	0	0	7.49E-05	0	0.000220456
0	0	0	0	0	0	0.000130288	0	0	0.000152451	0
0.00137028	0	0.000190663	0	0	0	0.008675259	0.006259802	0.002396674	0.00551207	0.002389739
0.000486778	0	0.000335304	0	0	0	0.009008923	0.014695566	0.001526913	0.051023568	0.000141092
0.00040646	0	0.000233398	0	0	0	0.005888372	0.00689874	0.000785201	0.002567853	0
0	0	0	0	0	0	0.001849451	0.010928962	0	0.003468269	0
0	0	0.000138066	0	0	0	0.000142999	0.000201064	0.00019328	0.000781313	0
0	0	0	0	0	0	0	0	0	0	0
7.79E-05	0	0	0	0	0	0	0	3.62E-05	0.000276318	0.000123455
0	0	0	0	0	0	0.000279642	0	0.000376896	0	0.000590821
0.00013143	0	0	0	0	0	0.000263753	0	0.000285088	0.000166744	0.000304229
0.000389422	0	0	0	0	0	0.000136643	0.000772981	0	0.000343016	0
0.000386988	0	0	0	0	0	0.000556106	0.000218937	0.000154624	0	0
0.000119261	0	0	0	0	0	0.000521151	0	7.73E-05	0.000447826	0.000665776
0.000111959	0	0	0	0	0	0.000120755	0	5.56E-05	0	0.000185183
0.000141166	0.000885895	7.23E-05	4.82E-05	0.002670284	0.0007372	0	0	5.07E-05	0	0.00019841
0	0	0	0	0	0	0	0	0	0	0
0	0	0	0	0	0	0	0	0	0	0
0	0	0	0	0	0	0	0	0	0	0
0	0	0	0	0	0	0	0	0	0	0
0.001158531	0.028037486	0.0021466	0.003996536	0.003460829	0.004430335	0	0.000433405	0.00051944	0.000419241	0.004365022
0.000535456	0.018468353	0.000594999	0.002049268	0.000134685	0.018858053	0.000146177	0	0.000309248	0.000247733	0.003681609
0.02096309	0.008580737	0.006525269	0.007423727	0.011770332	0.003326913	0.004741204	0.000951704	0.004418867	0.003082376	0.012177969
0.061718569	0.071962514	0.089338663	0.052337	0.181909214	0.127968419	0.086749415	0.020173451	0.059846777	0.07697365	0.08393187
0.004025653	0.023300509	0.018297053	0.005168606	0.024784064	0.031968324	0.010280023	0.005598524	0.007158613	0.012186581	0.011296147
0.000233653	0.002774829	0.000203812	0.000484035	0.002338451	0.025126632	0.000139821	0.000388724	0.004863411	0.002486863	0.003809473
4.38E-05	0.000307501	5.26E-05	0	0.00021862	0	0.000120755	0	4.59E-05	0.000114339	0.002663104

H018	H019	H020	H021	H023	H024	H025	H026	H027	H028	H029
0.003985903	0.074894103	0.014487711	0.000201361	0.011034198	0.035796108	0.053997596	0.032220399	0.011790572	0.015421231	0.054857079
0.005348043	0.024437667	0.010189644	0.012966753	0.010045321	0.030916953	0.003697575	0.019448329	0.013028349	0.017866681	0.00681656
0.003161233	0.026908141	0.007260092	0.008165072	0.004727824	0.02007178	0.001260489	0.019342997	0.001981777	0.008335604	0.007177536
0.008416963	0.048944774	0.005775601	0.003996238	0.010945487	0.018109171	0.012909004	0.050727463	0.017205428	0.043829146	0.027856673
0.001624722	0.011073067	0.00014381	0.000267744	0.001150646	0.006528997	0.001463929	0.009878801	0.001931732	0.006951182	0.003783247
0.005668064	0.016534819	0.005562205	0.011333739	0.01140731	0.025341893	0.003762592	0.017680095	0.001227768	0.015719191	0.006830551
0.000207193	0.002697491	0.000668021	0.000743486	0.000913211	0.002830535	0.000308306	0.00038547	0.000243552	0.001344452	0.003478237
0.000352844	0.007982971	0.004126423	0.003589091	0.000868855	0.013574059	0.00147861	0.005405551	0.002081867	0.003034102	0.002607978
0.015802063	0.033478268	0.020578766	0.01869558	0.010830683	0.029290568	0.016663206	0.024407899	0.031514925	0.027067095	0.020953367
0.0044167	0.017106367	0.003627727	0.004441002	0.002014283	0.002861812	0.00277056	0.003948831	0.003806747	0.006791301	0.0017685
0.004943914	0.00583032	0.000904612	0.008439453	0.001860341	0.004308356	0.007013438	0.001362593	0.001424611	0.004124198	0.002196634
0.175792052	0.149809573	0.139581652	0.183554793	0.159991964	0.005997295	0.175147179	0.121279762	0.060023821	0.125935212	0.038800666
0.001698573	0.000697074	0.000781678	0.001498036	0.000910602	0	0.001801598	0.000437016	0	0.001824095	0
0.004154119	0.001180486	0.002931871	0.002091055	0.002950976	0.004214526	0.000216024	0	0	0.00583565	7.56E-05
0.004591071	0.01063773	0.004986964	0.002515904	0.007824914	0.014598369	0.005163601	0.00337959	0.000343641	0.003920714	0.001214445
0.041559651	0.032706411	0.012819978	0.027081927	0.019887127	0.001329257	0.009949685	0.007991789	0.000567175	0.041361894	0.007160746
0.094307729	0.248008931	0.200724154	0.059529789	0.120478627	0.360119164	0.072271544	0.348626649	0.087815408	0.108769826	0.245689981
0.003770504	0.050710161	0.012694724	0.009220557	0.028051234	0.000633352	0.000553692	0.032619317	0.015440512	0.087792736	0.035028612
0.000592859	0.000654342	0.000554365	0.000628423	0.000647075	0	0.000119547	0.001891495	0	0.001765956	0.000548459
0.004911092	0.001468931	0.004321263	0.004000664	0.002966631	0	0.006040281	0.010129805	0.000767355	0.003306626	0.000352581
0.038525606	0.022958053	0.037388315	0.025019638	0.014371332	0.044397182	0.036417862	0.048504285	0.008704472	0.048523828	0.031958922
0.00392436	0.001898927	0.003126711	0.001810035	0.000761879	0.002752344	0.003567541	0.004975258	0.000587193	0.00293236	0.00189722
0.00115905	0.001901598	0.002368228	0.002814626	0.001388081	0.000703724	0.000950086	0.001550846	0.000500449	0.00305227	0.000615617
0.000953909	0.000990861	0.000767761	0.001692759	0.000641857	0.011212673	0.001159818	0.001846672	0.0086244	0.002696172	0.008853693
0.000182576	0.000117514	0.000702814	0.00047353	0.000198297	0	7.55E-05	0.001869083	0.006719358	0	0.00118926
0.002695562	0.001196511	0.003405053	0.003389943	0.001067152	0.000297128	0.001065438	0.003803159	0.006435771	0.001438927	0.007642047
0.000131291	0.00017093	3.94E-05	0.00025668	8.09E-05	0	0.000318793	0.000378747	0.000713974	0.000221653	0.000537266
0.000346689	0	0.000322413	0.000154893	0.000101758	0	3.57E-05	0.000663368	0.002575643	0	0.000990584
0.003493563	0.00027242	0.002101484	0.000274382	0.0002844	0.000218936	0.000132131	0.003092728	0.015730772	0.000152614	0.004799015
0.001522151	0.000189626	0.000783997	0.000227914	0	0.001290161	0.000448826	0.002597443	0.010092383	0.000290692	0.001941992
0.00451722	0.000619622	0.002556109	0.000838635	0	0	0.000113255	0.006122705	0.021205681	0	0.006066627
0.001351884	0.000146893	0.000719051	0.000278807	0	0	0.000161494	0.001340182	0.00560169	0	0.001376744
0.001622671	4.27E-05	0.000902293	8.41E-05	0.000101758	0.000320585	0.000123742	0.0021044	0.006592578	0	0.002123879
0.000902623	0	0.000394318	3.10E-05	0	0	9.86E-05	0.000813522	0.003232899	0	0.000968198
0.082844412	0.002681466	0.036755087	0.018958898	0.050299664	0.003839207	0.0739494	0.00117434	0.152670228	0.035802402	0.031021505
0.074078709	0.036477557	0.058222228	0.010019362	0.028160819	0.042778616	0.04085579	0.007279116	0.114509343	0.061070838	0.030654933
0.125870826	0.038814493	0.066308069	0.017414394	0.075791297	0.053373576	0.163947492	0.018249337	0.11688147	0.096397231	0.141452017
0.002262713	0.000483412	0.002623375	0.000814294	0.004633894	0	0.008259246	0.001266226	0.000470422	0.003015934	0.004194591
0.003339706	0.000811918	0.003075681	0.019983404	0.013460731	0.02387971	0.000914431	4.93E-05	0.00273245	0.001079196	0.002392512
8.00E-05	0	0	0.000294297	0.001030624	0.000328405	0.000197148	0	0	0.00014898	0.000291019
7.18E-05	0.001687935	0.006037706	0.008156221	0.020437663	0.008350861	0.007405636	0.021823903	0.00120775	0.051993968	0.000363774
0.001513946	0.000635646	0.006877372	0.004593683	0.015662874	0	0.00317744	0.001521712	0.000160144	0.002340074	0.005445413
8.82E-05	0.000590243	0.000607714	0.000913868	0.010517582	0	0.003838095	0.000443739	0.00049044	0.000959285	0.000397353
0.0014483	0.000673037	0.006478415	0.004020579	0.001755974	0.016013637	0.001256295	0.000172565	0.000483767	0.000861176	0.005526562
0.000418489	0.000315152	0.001561036	0.001960502	0.021601355	0.004519474	0.000786495	0.00010085	0.000733991	0.00168965	0.000794706
0.001452403	0.003685681	0.009036843	0.001305526	0.003741556	0.02852429	0.063276139	0.000712672	0.003242908	0.008837049	0.001063339
0.068160372	0.001492968	0.002154832	0.009824639	0.049334269	0.012002408	0.016541562	0.000782146	0	0.004167802	0.000352581
0.01307573	0.000723782	0.004103228	0.005352658	0.008169325	0.005559422	0.007132985	0.018926151	0.113268231	0.028320706	0.002305766

0.015681029	5.88E-05	0.001122647	0.004485258	0.016080342	0	0.010027286	0.006635919	0.001821633	0.000170782	0.00057924
0.002133473	0.002299544	0.082607324	0.013035349	0.017687593	0.000304947	0.003282306	0.002709499	0.003653276	0.001914936	0.027982595
0.000557985	0.000178942	0.000296898	0.000462466	0.000112194	0.001642023	0.000297819	9.19E-05	0.000223534	0.000272524	0.000226659
0.002631968	0.013193669	0.018294041	0.01879958	0.003903325	0.001102501	0.002634234	0.035503622	0.001514692	0.000265257	0.000461713
0.000190782	0.000117514	0.001906644	0.020746805	0.008445897	0	0.0005474	0.001086937	0	0.000250722	0.000814294
0.000100519	0.000841297	0.000899973	0.007140565	0.002997941	0.003659366	0.002330122	4.71E-05	0	0.000628622	0.001166874
0.015865657	0.001546383	0.003001457	0.038327156	0.018598195	0.00194697	0.009599433	0.004092262	0.000113435	0.008851583	0.00131798
0.019683343	0.001711972	0.005035674	0.008012391	0.008211072	0	0.013401874	0.010134287	0	0.002608964	0.001494271
6.56E-05	0.006540748	0.011504811	0.1130962	0.004372976	0.001282342	0.015310435	0.000407882	0.000140126	0.001780491	0.020267793
0	0	0.000155408	0.004429939	0	0	0.001002519	6.50E-05	0	0	0.000540064
0.016741612	0.013482114	0.018952784	0.048771367	0.024160955	0.004519474	0.025293677	0.010766279	0.003893491	0.009803601	0.015186154
0.000268736	0.000224346	0.002075969	0.003710793	0.000401813	0	0.001501681	8.07E-05	0	0.00033793	0.001130497
0.002032954	0.001001544	0.001558716	0.036078995	0.00101236	0.007748786	0.000218121	0.000318237	0.001367893	0.00162061	0.000380563
6.77E-05	0	0.001844017	0.009605576	0.003110136	0.000875746	0.006514275	0	0.000243552	0.000897513	0.004435241
0.001282135	0.000253724	0.002419258	0.002394203	0.004114668	0	0.001726094	0.00054683	0.000503785	0.001537036	0.009737943
0	5.61E-05	0.000215715	0	0.000874073	0.000336224	0.000115353	0	0	0	0
0	9.35E-05	0	4.20E-05	0	0.001907875	0	0	0	0.00014898	0
0	0	0	0	0	0.000289309	0	0	0	0	0
0	8.28E-05	0	0	0	0	0	0	0	0	0
0	0	0	0	8.35E-05	0	0	0	0	0	0
0	0	0	0	0	0	0	0	0	0	0
0	0	0	0	0.000109585	0	0	0	0	0.000116277	0
0	0.00371773	0	0.000139404	0.00157855	0.033528552	0.000545303	0	0	0.000559583	0
0	0.00163719	0	0	0.001662044	0.000297128	5.03E-05	0	0	0.000817572	0
0	0.000387264	0	0	0.000769706	0.000562979	4.61E-05	0	0	0.000585018	0
0	0.000830614	0	0	0	0.000312766	0	0	0	0	0
0	9.08E-05	0	0	0.000120022	0.003104206	4.19E-05	0	0	0	0
0	0	0	0	0	0	0	0	0	0	0
0	6.14E-05	0	0	0	0	0	0	0	0	0
0	0	0	0	0	0.000297128	3.36E-05	0	0	0	0
0	0	0	0	0.000164378	0	0	0	0	0	0
0	5.34E-05	0	0	0	0	0	0	0	0	0
0	0.000141552	0	0	0	0.000562979	2.94E-05	0	0	0	0
0	0	0	0	6.00E-05	0.000453511	0	0	0	0	0
0	0	0	0	0	0	0	0	0	0	0
5.13E-05	0	0.000245869	0.000528849	0	0	0.000119547	6.05E-05	0.000483767	0	0.000408546
0	0	0	0	0	0	0	0	0	0	0
0	0	0	0	0	0	0	0	0	0	0
0	0	0	0	0	0	0	0	0	0	0
0	0	0	0	0	0	0.000155202	0	0	0	0
0.002457597	0.000413971	0.002231377	0.001464845	0.006569901	0.009961608	0.006413604	0.000251004	0.020374936	0.001010156	0.000344186
0.004703899	0.000659683	0.0007724	0.001737014	0.001205439	0.004894793	0.000593542	5.15E-05	0.002575643	0.000439672	0.001480279
0.01607285	0.006639567	0.0122285	0.015969464	0.017998085	0.004628942	0.023802483	0.009726406	0.006442443	0.008579059	0.01583535
0.065965356	0.046904295	0.096392221	0.128890856	0.107831959	0.068073594	0.045474089	0.047468893	0.090441096	0.064228484	0.104391868
0.011487934	0.010968907	0.022529481	0.012351607	0.011255978	0.009508097	0.015276878	0.006626954	0.009618625	0.005032612	0.024652666
0.000617476	0.00040863	0.001567994	0.004058195	0.003251031	0.001313619	0.000652266	0	0.001004234	0.000552316	0.002378521
0	6.14E-05	0	0.001801184	0.000153941	0	0.000167786	0	0.000220197	0	0.000372169

H030	H031	H032	H033	H034	H035	H036	H038	H039	H040	H041
0.014809348	0.002711862	0.000200517	0.000854705	0.030377716	0.000240961	0.001071441	0.023955632	0.003383014	0.011590315	0.004014727
0.009278177	0.0030926	0.007926758	0.004117668	0.014289018	0.009490436	0.014944108	0.015329256	0.004663135	0.011983575	0.047490795
0.005246326	0.003311704	0.002726013	0.000658626	0.011282326	0.001230624	0.005653096	0.009948995	0.003226733	0.011817103	0.034125183
0.016774778	0.000795599	0.003441782	0.002141791	0.019998844	0.002444037	0.011838798	0.026583603	0.014642106	0.011105374	0.023104857
0.014778274	0.000143675	0.002299597	0.000191052	0.006078434	6.20E-05	0.000501459	0.011817237	0.009521621	0.018565252	0.063726232
0.005872985	0.003335051	0.000860445	0.005103093	0.019037569	0.008282186	0.046887994	0.035448257	0.006090344	0.006670945	0.008543905
0.00196543	9.88E-05	0	0.000467574	0.001394932	0.001067115	0.003370055	0.004016203	0.000429771	0.000441513	0.001069249
0.002247686	0.002002468	0.000149753	0.000985425	0.001691265	0.000130808	0.020130006	0.003363527	0.00425175	0.00650206	0.039370555
0.013392892	0.027797483	0.003642298	0.010140825	0.084975209	0.007937956	0.041646032	0.109577073	0.03238684	0.046491541	0.111065718
0.000740597	0.004281509	0.000327426	0.000990452	0.013963775	0.003858824	0.004260846	0.009814315	0.004681521	0.004779437	0.016916326
0.002221791	0.001142215	0.00135793	0.002197095	0.004412466	0.00370392	0.00202141	0.004154335	0.004136837	0.00121597	0.003122005
0.049821972	0.029103128	0.165819758	0.168226085	0.053195334	0.111002296	0.262179074	0.106672837	0.338641509	0.057823704	0.048363343
0.000396193	0	0.001771655	0.000829567	0.000126483	0.001311518	0	0.000721742	7.35E-05	0.000313643	0.000156352
0.001095358	0	0	0.000804428	0.000343312	0.002910469	0.0043574	0.01415513	0.011989934	0	0.001336561
0.002294297	0.003593666	0.000401034	0.002473617	0.002193585	0.000638548	0.004777878	0.006012218	0.009910024	0.004299322	0.001941797
0.023054315	0.001819282	0.018754664	0.021805037	0.021292589	0.009614359	0.016211772	0.029819357	0.008464429	0.051753506	0.026383215
0.180866188	0.022397106	0.056687869	0.049849421	0.04850099	0.1373497	0.120970028	0.166290831	0.159576204	0.117604057	0.15151561
0.007178093	0.013641923	0.010071526	0.001080951	0.023301869	0.000378654	0.004930497	0.055501646	0.015304	0.119073354	0.003641499
0.000344403	0.000359187	0.000411186	0	0.000542072	0.000318413	0.000376873	0.000569797	0.001604174	0.000916803	0.000776719
0.003262769	0.005926586	0.010909128	0.003921588	0.011145001	9.64E-05	0.007895647	0.011920836	0.018988084	0.003035099	0.021268977
0.037273257	0.003805586	0.016211401	0.012614443	0.238612874	0.02474329	0.013389895	0.090974076	0.021867782	0.061539167	0.215610027
0.002861397	0.000434616	0.000804605	0.000382103	0.019207418	0.002020633	0.001052753	0.009040773	0.001978787	0.007221027	0.017168508
0.002146695	0.00032686	0.00252042	0.001603829	0.002417641	0.001265047	0.003201864	0.001737016	0.003189961	0.004579188	0.012851163
0.003016767	0.001765404	0.001766578	0	0.011958109	0.000934586	0.000573096	0.001253553	0.000395298	0.002248579	0.004877188
0.000178675	0.000549556	0.000639623	0	0.00012287	0.000273663	0	0	7.12E-05	0.000441513	0.000151309
0.001742733	0.00670243	0.003665142	0.001015591	0.000393906	0.002802036	0	0.000214105	0.00030107	0.008497312	0.003111918
0.000212339	0.000316085	0.000385804	0.000196079	0	7.06E-05	0	0	0	0.000731029	0.000322792
5.18E-05	0.000210124	0.000164982	0.000211162	0	8.95E-05	0	0	0	0	0
0.000145012	0.001061398	0.000835064	0.000186024	0.000184304	0.001265047	0.000208682	0	0.000144789	0.000518717	0.000267312
0.000419499	0.000743517	0.000568554	0.000226245	0.001246766	0.000280548	0	0	0	0.000400498	0.000151309
0.000176086	0.000993152	0.001363006	0	0	0.005251237	0.000330153	8.98E-05	0.000179263	0.000646587	0.001225601
0	0.000459759	0.000276662	0	0	0.004098064	0	0.000296985	5.29E-05	0.000151996	0.000474101
0.000214928	4.31E-05	0.000365499	0.000221218	0.000112028	0.005259842	0.00040179	0.000131226	0	0.000248502	0.001825793
0.000139833	0.000319676	0.000380728	0	0.000101187	0.000165231	0	0	0	9.41E-05	0
0.011173691	0.032659079	0.006218558	0.047084199	0.006634961	0.075085283	0.017688117	0.002911143	0.046969192	0.005015875	0.002763908
0.004091409	0.017654042	0.010546167	0.008521913	0.034204744	0.073376179	0.04278911	0.011478812	0.020766924	0.017607435	0.004292127
0.062463909	0.052103669	0.008244031	0.051780049	0.119837668	0.11717607	0.07406646	0.016230571	0.05440171	0.080237114	0.017334947
0.000818282	0.005350091	0.000538096	0.005590777	0.00268145	0.000993105	0.000915708	0.000324611	0.001195086	0.001314888	0.000176527
0.003633068	0.00149781	0.004616962	0.013871362	0.000585438	0.000974172	0.004295107	0.001336432	0.00054928	0.00135349	0.000907853
0.000300382	0.000928498	0.003228574	0.000206135	0	0.000598961	0.00054195	0	0	0.003973615	0
0.00201722	0.000612414	0.004959617	0.126441058	0.003472875	0.000464711	0.000598013	7.60E-05	0.000420578	0.04093041	0.000141222
0.029209555	0.000353799	0.014325528	0.049366764	0.000570983	0.008070485	0.007615328	0.007141451	0	0.005278853	0
0.001921409	0.000400494	0.005139829	0.026304808	0.000932364	0.000261615	0.001875021	0.004458227	0.000473438	0.000991594	0
0.020226581	0.004664043	0.014881391	0.016294702	0.001109441	0.004726285	0.00750943	0.000680303	0.001110051	0.011102962	0.000549755
0.024809995	0.001223032	0.010480174	0.005495251	0.005453244	0.001376922	0.001239632	0.000801169	0.000900911	0.011771263	0.00045897
0.006409011	0	0.013404166	0.002996496	0.015589991	3.44E-05	0	0.000424757	0	0.005042414	0.003232965
0.006649835	0.038939464	0.017376428	0.023554668	0.0048136	0.029383518	0.005702931	0.006119271	8.04E-05	0.001363141	0.000635497
0.00776073	0.000132899	0.00097974	0.019753744	0.030609	0.015901729	0.022101581	0	0.000386105	0.005785507	0.000464014

0.000831229	0.000377146	0.016125102	0.013539535	0.002146605	0.004504256	0.00058244	9.67E-05	0.000190754	0.002656315	0
0.035424354	0.023846426	0.010264428	0.002644558	0.000997413	0.001860566	0.000336383	0.001512551	0.001657033	0.005508053	0.000776719
0.000582637	7.72E-05	0.006322624	0.006209182	0.000115642	0.000135971	0.000193109	0.000214105	0	0.000921628	0.000332879
0.004888975	0.002891455	0.008038438	0.025595905	0.001590078	0.005173785	0.002326646	0.001578164	0.000884824	0.003027861	0.000121047
0.002752638	0.000373554	0.017411963	0.0019256	9.40E-05	0.001228903	0.002927774	0.000770089	0.001280121	0.003742002	0.000902809
0.002610216	0.006012791	0.003180348	0.004389162	0.000982957	0.014127221	0.003317106	0.002662504	0.000280386	0.010516691	0.000504363
0.006763773	0.011219206	0.002718398	0.018471687	0.005012359	0.041166528	0.00179404	0.026383311	0.00304747	0.014490306	0.001422303
0.002983104	0.004416204	0.002236143	0.003438931	0.000910681	0.020688255	0.00534786	0.002759197	0.000972157	0.004002567	0.000363141
0.045352496	0.005007067	0.002066084	0.009959829	0.00411252	0.040130395	0.008512348	0.001716297	0	0.002482605	0.000126091
0.001147148	0.003791219	0.000337579	0.000226245	0.000104801	0.000499134	0.000395561	0.000307345	0.000266596	8.44E-05	0
0.056445912	0.026416409	0.043413151	0.014881925	0.009464577	0.083804642	0.031065554	0.02024332	0.038114062	0.036954382	0.009376103
0.006150062	0.000343024	0.000380728	0.003725509	0.000784198	0.002240941	0.00018065	0	0.000413684	0.000509067	0.000176527
0.005326601	0.005321356	0.143615698	0.044675941	0.000419202	0.000557653	0.000379988	9.32E-05	0.000852648	0.003850571	0
0.013359228	0.01548096	0.085475986	0	0.00047341	0.001430278	0	0.000196839	0.003472645	0.000448751	0.000171483
0.016057487	0.007885951	0.01574945	0	0.000224056	0.003203065	0.001681913	0.000683756	0.004171311	0.001633356	0.001795531
0.00010358	0	0.000642161	0.000226245	0	3.27E-05	0.00090325	0.001163766	0.003077347	0.000340182	0.001165078
4.92E-05	0	0	0	9.76E-05	0	0.000161962	0.001540178	0.000795192	0	0.00235033
0.000150191	0	0	0	0	0	0.000193109	0.000473104	0.000815876	0	0.000272356
0	0	0	0	0	8.43E-05	0.000218026	0	4.83E-05	0	0.00016644
9.06E-05	0	0	0	0	3.61E-05	0	0	0.000101123	0	0
0	0	0	0	0	0	0.000177535	0	6.21E-05	0	0
4.14E-05	0	0	0	0	0	0.000379988	0.000193386	0.000266596	0	0
0.00491487	0	0	0.000452491	0.00050232	0.00365917	0.007861385	0.016268557	0.006384519	0	0.001225601
0.0026387	0	0	0	0.001044392	9.98E-05	0.014096922	0.020343467	0.007064799	0	0.016734756
0.000963294	0	0	0	0.00057821	3.79E-05	0.011162918	0.00790118	0.002403962	0	0.00227972
0.000960704	0	0	0	0.000325243	0	0.0003613	0.009386105	0.001036508	0	0.009628285
0.000240823	0	0	0	0	6.02E-05	0.00147946	0.001080887	0.000517105	0	0.000479145
0	0	0	0	0	0	0	0	0	0	0
0.000209749	0	0	0	0	0	0	0.000107053	0.000124105	0	0
0.000113938	0	0	0	0.000108414	0.000189327	0.000193109	0	0.000213737	0	0
0	0	0	0	0	0.000144577	0.000526376	0	9.88E-05	0	0
0	0	0	0	0.000148166	0	0.00054195	0.000538717	0.000176965	0	0
7.77E-05	0	0	0	0	2.75E-05	0.000890791	0.00058361	0.000542385	0	0.00023705
6.47E-05	0	0	0	0	5.16E-05	0.000186879	0.000787355	0.000108017	0	0.000307661
0.000116527	0	0	0	0	0.000151461	0.000566867	0.00050073	0.000475736	0	0
7.25E-05	0.00072017	0.000568554	0.000447463	0	0.000273663	0	0	0	0.00086855	0.000181571
0	0	0	0	0	0	0	0	0	0	0
0	0	0	0	0	0	0	0	0	0	0
0	0	0	0	0	0	0	0	0	0	0
0	0	0	0.000211162	0	0	0	0	0	0	0
0.001113485	0.047980202	0.003198116	0.008054339	0.00072999	0.000469875	0.005774568	0.001481471	0.00068028	0.005713128	0.000796893
0.003809154	0.038468929	0.003977339	0.006435427	0.000831177	6.71E-05	0.000379988	0.003705405	0.00011721	0	0.000771675
0.010093869	0.0294246	0.010018224	0.011996038	0.007198716	0.010784742	0.018233182	0.004734492	0.012571389	0.011416605	0.001588743
0.169951447	0.392070587	0.09625059	0.118361581	0.078889547	0.073801303	0.080828373	0.062805402	0.088404215	0.112759479	0.040238059
0.013465398	0.043468813	0.014178313	0.012086536	0.012883245	0.00996031	0.014685591	0.005221409	0.009438884	0.011940147	0.0069249
0.004065514	0.034189216	0.074658233	0.001040729	0.000234898	0.000129086	0.001034065	0.000483464	0.000707859	0.000986769	0.001503001
0.00031074	0.000413065	0.006525679	0.000246356	0	0.000175558	0	8.98E-05	0.000763017	7.72E-05	0.000151309

H042	H043	H044	H045	H046	H047	H048	H049	H050	H051	H052
0.067848599	0.023995672	0.002182207	0.299766546	0.005652785	0.021428056	0.004198102	0.01131118	0.029108148	0.00090834	0.001560499
0.008144919	0.023107575	0.005184657	0.000840791	0.004047868	0.005435399	0.007544415	0.005274531	0.009110452	0.005716249	0.008039575
0.037861718	0.007562673	0.009058457	0.006748689	0.001586469	0.001351114	0.002423101	0.001902883	0.012742137	0.004052842	0
0.056563006	0.002342703	0.009814819	0.015952665	0.001188534	0.005536475	0.002637371	0.01098318	0.004061239	0.002554	0.01282015
0.033208581	0.000900876	0.000868794	0.001062169	0.000221368	0.000346545	0.000798883	0.000496824	0.000880586	0.000425936	0.002363748
0.013820511	0.019150536	0.026822743	0.004767463	0.013740615	0.002126716	0.006348736	0.005144296	0.009081164	0.00463689	0.001004947
0.002048769	0.003522574	0.001180538	0.000138641	0.003847583	0.000909682	0.000211625	0.000342471	0.0007361	0.000348493	3.89E-05
0.021116599	0.005100704	0.030259591	6.48E-05	0.000766881	0.000717844	0.00094702	0.003632119	0.002290304	0.001311688	8.49E-05
0.105648584	0.026561998	0.177060256	0.005474085	0.017825378	0.007089741	0.016276572	0.023854773	0.040260935	0.012768388	0.010300706
0.005343777	0.011564436	0.011815601	0.0033654	0.002619519	0.004616479	0.016207794	0.001854648	0.00547291	0.001290714	0.001546344
0.002473185	0.003501277	0.00327331	0.01339675	0.001009332	0.003488144	0.000568741	0.001172118	0.002864345	0.003341336	0.002954685
0.047750598	0.186474934	0.294334695	0.006216486	0.246437692	0.171327496	0.0720608	0.129424987	0.019191306	0.098236239	0.011065031
0.000173625	0.001616465	0.000758917	9.84E-05	0.000943448	0.001099456	0.00021956	0.001121471	0.000361216	0.000696986	0
0.002083494	0	0.000301523	0	0.00195278	0.001423311	0.000243368	0.000728353	0.000339738	0.003205811	0.005760752
0.000331816	0.005092185	0.002202649	0	0.00123597	0.001674969	0.002002497	0.004191649	0.000732194	0.002382981	0.000682939
0.011459218	0.005969634	0.001770807	0.001093475	0.003428565	0.006891715	0.003161142	0.001210706	0.033641896	0.023310294	7.43E-05
0.089177406	0.133155004	0.018824726	0.003953506	0.062536401	0.064166589	0.011591734	0.033984188	0.113128931	0.171948914	0.091828791
0.149610309	0.006813008	0.000546829	0.000415923	0.003868666	0.001068515	0.020614875	0.000672883	0.029719286	0.005398411	0.000297238
0.000555598	0.000355665	6.13E-05	0.00040027	0.000234544	0.000253721	0.000547578	0.000571588	0.00055061	0.00062761	0
0.018562389	0.001115979	0.002562943	0.000541147	0.003673651	0.009534123	0.000928503	0.000381059	0.003055692	0.001568217	0.001036794
0.030341847	0.00568851	0.010905922	0.000210198	0.01800458	0.001307796	0.004758907	0.035660366	0.020019174	0.012177886	0.011670123
0.002245544	0.000523914	0.001418179	0	0.001741954	7.63E-05	0.00086237	0.002438295	0.001923231	0.001331049	0.001397725
0.003163824	0.006440305	0.000493168	0	0.000669374	0.001765731	0.000801528	0.002778354	0.00180608	0.001421399	8.14E-05
0.000941431	0.00205306	0.000306633	0	0.00031624	0.000802418	0.000338599	0.001097353	0.000654094	0.000608249	7.78E-05
0.000354966	0.000570768	0.000286191	0.000657427	0.00020292	0.000515693	0.000156073	0.000815177	0	0.001377837	0.004094097
0.001323405	0.004238163	0.002867021	0.011136007	0.002442952	0.0064936	0.002682341	0.007367944	0.001784602	0.002718566	0.005842138
8.87E-05	0.000268346	6.64E-05	0.000695441	0.000387394	0.000705467	0.000190462	0.00064153	0.000148391	0.000164566	0.001418957
0	0.000172508	6.13E-05	0.000440521	0	0.000534257	0	0.000246	0	0.000322678	0.001026178
0.000378116	0.000457892	0.00036796	0.00113149	0.000176567	0.006012975	0	0.001111824	0.000326071	0.001577898	0.007105399
9.26E-05	0.000247049	0.000250417	0.000415923	8.17E-05	0.001580082	5.03E-05	0.000383471	0.000283115	0.000968035	0.004663803
0.000150475	0.000438724	0.000511055	0.001639095	0.000811682	0.016473282	0.000407377	0.002013824	0.000300688	0.001882829	0.009348837
0.000119608	0.000204454	6.39E-05	6.26E-05	0.00031624	0.004971276	0	0.000400353	3.51E-05	0.000761521	0.003800398
0	0.000483449	6.90E-05	0.000695441	0.000455912	0.000901431	0	0.000451	0	0.000832511	0.002855606
0	5.11E-05	4.60E-05	0.000219142	8.43E-05	0.000604392	0	0.000144706	0	0.000224262	0.001666655
0.000432132	0.033104529	0.006137775	0.00027281	0.133803652	0.097387502	0.022112119	0.047499723	0.004582561	0.035778591	0.036620406
0.001558762	0.113966133	0.008105339	0	0.03731892	0.046191611	0.066500365	0.029782893	0.007124741	0.019801166	0.010134394
0.041473107	0.101873524	0.148027198	0.016844887	0.135303155	0.071342966	0.19954342	0.136267166	0.059026591	0.127288597	0.341689018
0.000235358	0.005004866	0.001522945	0.002430691	0.004256059	0.003244737	0.004023511	0.004107237	0.00079858	0.000784109	0.006312765
0.000335674	0.002221309	6.90E-05	0.005413709	0.004124293	0.001930753	0.004028802	0.000344882	0.006142624	0.002662098	0.011365808
0	0.000204454	0	6.48E-05	0.00075634	0.001047887	0.000486736	0.000202588	0.000144486	0.002281337	0
0.000270083	0	0.001793804	0.000360019	0.002092453	0.063770537	0.000108458	5.31E-05	0.000726337	0.007347389	0.017430875
9.65E-05	0.004985699	0.001775918	0.003763434	0.001098933	0.019594253	0.135902632	0.000197765	0.043494305	0.051265545	0.005194585
0.000605757	0.00742211	0.001093659	7.60E-05	0.001931697	0.004391638	0.037240628	6.51E-05	0.02534174	0.006947268	0.0077848
0.003090516	0.004561883	0.007348977	0.000610468	0.001860544	0.003250926	0.012647211	0.000159177	0.016018463	0.002079663	0.008216502
0.001443013	0.001075514	0.000490613	0.001815751	6.85E-05	0.002246357	0.008946427	0.000214647	0.000513512	0.006080876	0.004394873
0.024280423	0.000896616	0	0.000225851	0.006517173	0.011374114	0.00176442	0	0.000905969	0.006656857	0.001128796
0.000223783	0.019885293	0.000176314	0.002010295	0.00302536	0	0.001756484	0.014526064	0.001243754	0.041457732	0.074748232
0.002519485	0.000832725	0.000311744	0.000125224	0.045946993	0.035122786	0.001851715	0.020519301	0.000134724	0.008158926	0.084684468

0.000937572	0.001437568	0.000434397	0.000136405	0.001267594	0.001827614	0.001756484	0.013590299	0.000117151	0.007179596	0.012491065
0.003186974	0.006073991	0.014603408	0.057647843	0.006229922	0.001171653	0.014088903	0.003181119	0.064370635	0.004862765	0.010137932
0.000486149	0.000187416	0.000751251	0.001831404	0	7.01E-05	0.000362407	0.00117453	0.000386599	0.000104871	0.002038202
0.00287445	0.018760795	0.014368323	0.010169992	0.003966173	0.003191105	0.002182378	0.031386717	0.008229866	0.006455183	0.018941833
0	0.000146951	0.000564716	0.008132863	0.000160755	0.001233537	0.001690351	0.000325588	0.002237586	0.010380567	0.000247698
0.003325874	0.005077277	0.002713704	0.007526867	0.001383549	0.011555638	0.016966997	0.001864295	0.009565389	0.009067266	0.000254775
0.000922139	0.014829313	0.002928347	0.007191445	0.01202765	0.014235176	0.070822796	0.014374123	0.01493091	0.019959278	0.000403394
0.000590323	0.029633069	0.002373852	0.003870769	0.008802005	0.010747032	0.003904473	0.011849004	0.008009232	0.012566714	0.001620654
0.013029555	0.00552878	0.008113005	0.026375342	0.010567677	0	0.016906155	0.1505207	0.046729629	0.030372113	0.001040332
0.00062119	3.83E-05	0.000143096	0.000261629	0.000619303	0.000829234	0.000650745	0.00658412	0.003086932	0.003997986	8.14E-05
0.023111351	0.021780753	0.025979502	0.075306687	0.024719403	0.043083016	0.039094988	0.106477038	0.068174141	0.028507031	0.001486189
0.000235358	0.000538822	0.000222309	0.001645803	0.000171297	0.001809049	0.002682341	0.014362064	0.004309209	0.001803773	0.000707709
0.000331816	0.000387611	0.000940342	0.011934311	0.000239815	0.000847799	0.002018369	0.001015353	0.000855203	0.000141979	0.008775593
0.003950922	0.002033893	0.000145651	0.03385525	0.000152849	0.00288169	0.000888823	0.000118177	0.014292437	0.004514272	0.000421087
0.002774134	0.00079013	0.001395181	0.011352913	0.001979133	0.008706953	0.001124255	0.001647236	0.004506413	0.001402038	0.001673732
9.26E-05	9.16E-05	0	0.000196781	0.000339958	6.19E-05	0	0.000108529	8.59E-05	0	7.08E-05
0.000563315	0	0	7.60E-05	0	0	0	0	0	0	0
0.00016205	0	0	0	0	0	0	0	0	0	0
0	0	5.11E-05	0	0	0	0	0	0	0	0
0	0	0	0	0	0.000202151	0.000232787	0	0	0	0
0	0	0	0	0	0	0.000145492	0	0	0	0
0.000270083	0	0	0	0	0	0	0	0	0	0
0.00692183	9.16E-05	0	0	8.96E-05	0.000156771	0.000230142	0	3.91E-05	0	0
0.020371942	0	0	0	0	0.000315604	0.001391431	0	0.000101531	0	0
0.00220696	0	0	0	0	0.000429056	0.00121155	0	0	0	0
0.019750752	0	0	0	0	0	8.46E-05	0	3.71E-05	0	0
0.001956169	5.32E-05	0	8.94E-05	0.000105413	0	0	0	3.32E-05	0	0
0	0	0	0	0	0	0	0	0	0	0
9.65E-05	0	0	0	0	0	0.001576603	0	0	0	0
0.000192916	0	0	0	9.75E-05	0.000226905	0.000140201	0	0	0	0
0	0	0	0	0	0.000158833	0.000208979	0	0	0	0
0.000601898	0	0	0	0	0	0.000624292	0	0	0	0
0.000987731	0	0	0	0	0	6.35E-05	0	0	0	0
0.000273941	0	0	0	0	0	0.000203689	0	0	0	0
0	0.000106487	0	0	0	0	0.000370343	0	3.91E-05	0	0
0.000119608	0.000170378	0.000291302	0.001355104	0.000171297	0.000511567	0.000521125	0.000397941	0.000363168	0.00017102	0.000109695
0	0	0	0	0	0	0	0	0	0	0
0	0	0	0	0	0	0	0	0	0	0
0	0	0	0	0	0	0	0	0	0	0
0	0	0	0	0	0	0.000158718	0	4.10E-05	0	0
0.002060344	0.003222282	0.00068737	0.00054562	0.000732622	0.000554885	0.002147989	0.000154353	0.015813449	0.002147425	0.012912152
0.000582607	0.006127234	0.000403734	0.001028627	0.000450642	0.000144394	0.00198398	0.00051853	0.001880275	0.007098927	0.00868359
0.00117293	0.017105995	0.017102469	0.02450816	0.01903763	0.031473746	0.02324431	0.019952536	0.039327631	0.012332772	0.015063588
0.063442395	0.067427264	0.090847764	0.174987366	0.114354911	0.129896758	0.100087824	0.062681787	0.1567892	0.132841894	0.062681793
0.020395092	0.011607031	0.020764181	0.106290726	0.011656068	0.01845973	0.012647211	0.009417945	0.019802444	0.013765464	0.013205851
0.011852766	0.000751795	0.000723143	0.015831913	6.32E-05	0.00101282	0.000891468	0.000487177	0.004902774	0.000104871	0.010735947
0	0.000223622	0	0.002236146	0	7.84E-05	0	6.51E-05	0.000134724	0.00053242	0

H053	H054	H055	H056	H057	H058	H059	H060	H061	H062	H063
0.005444561	0.001618087	0.000605813	0.118020776	0.040131721	0.000901371	0.013632075	0.008874775	0.001351172	0.015094156	0.002517095
0	0.005827908	0.001795227	0.018095144	0.018097573	0.004059442	0.008056071	0.00540074	0.006446167	0.005910735	0.000843865
0	0.001971234	0	0.074573839	0.004969377	0.000626376	0.001014007	0.002257057	0.002217821	0.004729915	0.000173993
0	0.004078139	0.003925671	0.017644872	0.094436998	0.009622625	0.00256716	0.020720596	0.00444813	0.006784189	0.008870729
0.000247577	0.000337185	0	0.005850637	0.011715549	0.000220432	0	0.003303531	0	0.002193584	0.000403083
0	0.005301182	0.001373177	0.015957076	0.025330324	0.004229677	0.000870927	0.007625827	0.005714386	0.008316601	0.000611874
0	0.000865906	0	0.007224695	0.001531937	0.000622011	0	0.000385767	0.001573454	0.0006081	0.000260989
0	0.000800065	0.003818644	0.002393707	0.010871101	0.001536477	0	0.001195665	0	0.022263106	0.001081655
0.001005248	0.00868899	0.005765324	0.043194204	0.055746176	0.029005371	0.017399869	0.039868285	0.003061992	0.030884863	0.017877753
0.000256114	0.00062449	0.002170831	0.009208799	0.005848357	0.002896172	0.001841388	0.0082162	0.003573989	0.008593011	0.001763126
0	0.00783705	0	0.001400202	0.007339484	0.000395032	0.002455184	0.004691013	0.001993042	0.006209257	0.002090813
0.001809874	0.117031019	0.005737053	0.012825503	0.180526823	0.062790408	0.056137441	0.052790418	0.365053835	0.098857214	0.092752623
0	0.010255203	0	0.000145249	0.002034211	0.000892641	0.002187685	0	0.001538488	0.001001707	0.000495879
0	0.000704297	0	0	0	0.0027805	0.000493525	8.53E-05	0.005152438	0.00078058	4.93E-05
0	0.000796075	0	0.00097898	0.023967905	0.000938473	0	0.003772419	0.01094175	0.003137798	0.000194292
0.00023904	0.00659206	0.000571484	0.005740248	0.000954321	0.002097379	0.000705036	0.04882405	0.012754968	0.005430889	0.000513279
0.000966831	0.035408383	0	0.203287861	0.054700817	0.02947679	0.010127632	0.295148073	0.067283894	0.037651362	0.007388891
0	0.002481999	0	0.005847732	0.000229163	0.004450109	0	0.02912222	0.001173847	0.001397525	0.00024359
0	0.00084795	0	6.97E-05	0.000244859	0.001811472	0	0	0.000204799	0	0.000121795
0	0.002124862	0	0.007605248	0.010767507	0.000211702	0	0.001042211	0.00322683	0.001189665	0.000350885
0.000734194	0.002938894	0	0.002977608	0.024903391	0.00946985	0.000170038	0.007035454	0.000881634	0.03896707	0.008682237
0	0.000297281	0	0.000653621	0.002564738	0.001099978	0.000165891	0.000294121	0	0.003856462	0.00024359
0.000740597	0.001945296	0	0.000964455	0.002269652	0.00125057	0.002113034	0.002225088	0.001138881	0.000203437	0.000655373
0	0.000464876	0	0.000339883	0.000263694	0	0.001289801	0.00082908	0.000574436	0.000610312	0.000510379
0.051991181	0.019800123	0.046270007	0.000578092	0.000565059	0.014993747	0.039197918	0.01633436	0	0.011693217	0.000661172
0.035676707	0.028439234	0.032701807	0.002286222	0.002115831	0.02395944	0.038598638	0.023602127	0.000464544	0.090208921	0.007713678
0.000439663	0.001027515	0	0.000148154	0.000254276	0.00022698	0.00015967	0.001994906	0.000237267	0.010788806	0.000614774
0.030829746	0.009383311	0.026346824	0	0	0.022185071	0.018480233	0.003989812	0	0.002209063	0.000133394
0.188911963	0.068286882	0.122392478	0.000519992	0.000750272	0.131013049	0.128034505	0.0226473	0	0.013968618	0.000603175
0.122655221	0.039241115	0.083129713	0.000700101	0.0009606	0.085136984	0.083289614	0.014590948	0	0.008079995	0.00023779
0.284835265	0.094036619	0.176845005	0.001368247	0.001070472	0.191756276	0.177648291	0.031438954	0	0.017785276	0.001371643
0.103675025	0.032497421	0.067861196	0.000180109	0.000370427	0.066328234	0.067590125	0.011344963	8.49E-05	0.006850527	0.000510379
0.085292429	0.033993803	0.059157677	0.000438653	0.000587033	0.046834181	0.057655341	0.009407602	0	0.004672422	8.41E-05
0.043236344	0.01351532	0.02360047	0.000258544	0.000156961	0.030334511	0.030275068	0.005063993	0	0.002868022	6.38E-05
0.005557678	0.030123162	0.001607425	0.002010249	0.012597669	0.018863312	0.055202231	0.003542237	0.067508673	0.040932892	0.06530527
0	0.012611505	0	0.000145249	0.029257482	0.009779764	0.011259837	0.000890888	0.016548741	0.022784967	0.014238405
0.008547811	0.027397752	0.044505071	0.002559291	0.087003337	0.035849678	0.058690085	0.089679131	0.005999106	0.109714569	0.20167207
0	0	0	4.36E-05	0.005744763	0	0.00224782	0	0.000729283	0.003301432	0.006492829
0	0.002340341	0	0.000313738	0.00035787	0	0	0.001084837	0.010514669	0.003022812	0.003323261
0.001895245	0.000155624	0	0.000264354	0	0	0	0.002348704	0.001878155	0.000641269	6.09E-05
0	0	0.020506783	0.003846199	0.007185663	0	0	0.005535012	0.000259745	0.018565856	0.007278696
0.000245443	0.005093683	0.005975339	0.00806133	0.015824782	0	0.001497164	0.000664969	0.000114887	0.00069434	0.000304487
0	0.00557053	0	0.002437281	0.006736755	0	0.003071053	0.000534959	0	0.000212282	0.000130495
0	0.005552574	0.001587231	0.003204197	0.000756551	0.004836411	0	0.008870512	0	0.003703884	0.005759159
0.000221966	0.001209076	0	0.000958645	0.001042219	0.000674391	0.000165891	0.004273277	0	0.005773636	0.01769506
0	0	0	0	0.000583894	0	0	0.0008781	0.000119882	0.013544053	0.009836389
0	0.015614245	0.073775954	0.004101837	0	0.000248805	0.000852264	0.004993659	0.010115062	0.019021379	0.035659809
0.003421259	0.006643935	0	0	0.041604013	0.00680284	0.008773549	0.001227635	0.005577021	0.005273888	0.017135384

0	0	0	0.001345007	0.031128454	0	0.000514261	0.001270261	0.005889214	0.001227257	0.001360043
0.00026892	0.006204996	0.002558552	0.011242287	0.003220834	0.020419432	0.002973592	0.003437803	0.011853354	0.003250573	0.033928582
0	0.002264524	0	0.000116199	0.000144404	0	0.004605543	0.000362323	0.002500044	0.000176902	0.003708945
0	0.019630533	0	0.011378822	0.008256135	0.004094362	0	0.011195771	0.004220853	0.005127944	0.008055863
0	0.009006223	0	0.002143878	0.001641809	0.001032321	0	0.002964652	0	0.000835862	0.000536478
0	0.001177154	0.002576726	0.002771355	8.48E-05	0	0	0	0.0078373	0.006242426	0.000730769
0.000213428	0.019838032	0	0.021043703	0.001936896	0.005024106	0.000178333	0.004026045	0.108890515	0.012270359	0.007919569
0	0.012958666	0	0.003311682	0.003776475	0	0.009986625	0.005656497	0.001690839	0.00395818	0.003163768
0.000960428	0.054568054	0.000149434	0.073223021	0.00515773	0	0	0.03045642	0.011106588	0.011129342	0.047798702
0	0.00255183	0	0.001417632	0.000172657	0	0	0.0015452	0	5.31E-05	0.001632632
0	0.048095705	0.009816196	0.050314319	0.022542701	0.020594032	0.004937324	0.017419197	0.0492616	0.040786948	0.008873629
0	0.003701051	0	0.006100466	0.000869562	0	0	0.00701201	0.00150602	0.002259922	0.001386142
0	0.000728239	0	0.001629696	0.002828432	0.001820202	0	0.00063513	0	0.004614929	0.01268697
0	0.001576189	0.001304518	0.015521328	0.002181754	0.004395547	0	0.004582317	0.000224779	0.000165846	0
0	0.005716178	0	0.017691351	0.004350952	0.004718556	0	0.003015804	0.0008142	0.001954766	0
0	0	0	7.84E-05	0	0	0	0.000219525	0.000756756	0.000101719	0.000115995
0	0	0	0	0	0	0	0	0.000551958	0	0
0	0	0	0	0	0	0	0	0.000342164	0	0
0	0	0	0	0	0	0	0	9.74E-05	0	0
0	0	0	0	0	0	0	0	0.000546963	0	0
0	0	0	0	0	0	0	0	0.000472036	0	0
0	0	0	0	0	0	0	0	0.000454553	0	0
0	0	0	9.01E-05	0	0	0	0	0.001838194	0	0
0	0	0	9.01E-05	0.000222884	0	0	0	0.000104897	0	0
0	0	0	0	0	0	0	0	0.000242262	0	0
0.000230503	0	0	7.55E-05	0	0	0	0	0.000102399	0	0
0	0	0	0	0	0	0	0	0.000172331	0	0
0	0	0	0	0	0	0	0	0	0	0
0	0	0	0	0	0	0	0	0.000551958	0	0
0	0	0	0	0	0	0	0	0.000591918	0	0
0	0	0	0	0	0	0	0	0.000821693	0	9.28E-05
0	0	0	0	0	0	0	0	0	0	0
0	0	0	0	0	0	0	0	0.000222282	0	0
0	0	0	0	0	0	0	0	0	0	0
0	0	0	0	0	0	0	0	0.000332174	0	0
0	0.001157202	0.000153473	0.000569377	0.000235441	0	0	0.001084837	0	0.003838772	0.000405983
0	0	0	0	0	0	0	0	0	0	0
0	0	0	0	0	0	0	0	0	0	0
0	0	0	0	0	0	0	0	0	0	0
0	0	0	0	0	0	0	0	0.001318704	0	0
0	0.002174741	0.004468883	0.000174299	0.002762509	0.00023571	0	0.001683735	0.004897688	0.003604377	0.085534825
0	0	0.020716798	0.001095179	0.003895766	0.000611099	0	0.000151323	0.01147123	0.001923808	0.006025948
0.000738463	0.014463028	0.032221195	0.015968696	0.008777244	0	0.011228732	0.002497895	0.016191592	0.01570889	0.016540909
0.012660578	0.10233655	0.104846103	0.117773853	0.088265301	0.059885506	0.055390932	0.077159816	0.10795893	0.126668406	0.165394587
0.006050698	0.010283135	0.009161918	0.051694187	0.011197579	0.009271243	0.006268598	0.013561526	0.015986793	0.028868182	0.048474374
0	0.007422053	0	0.002904984	0.001218015	0.00668935	0	0.00141519	0.007632501	0.004247857	0
0	0.001773711	0	0.00081049	0.0001601	0	0	0	8.74E-05	0	4.93E-05

H064	H065	H066	H067	H068	H069	H070	H071	H072	H073	H074
0.013495123	0.001608262	0.011371294	0.006143382	7.74E-05	0.019605789	0.015261507	0.010616104	0.000378191	0.056803892	0
0.003553064	0.003291817	0.001175023	0.005609283	0.006645837	0.005308265	0.005904552	0.028772413	0.006830424	0.00804269	0.003746227
0.001947666	0.001171561	0.00135227	0.003000892	0.005179448	0.000321713	0.001699409	0.00732623	0.003207311	0.014371239	0.001457416
0.008247019	0.003386686	0.004232529	0.007119666	0.003639066	0.03626379	0.019339068	0.060372706	0.007430097	0.018381106	0.00109368
0.003197214	0.000194256	0	0.005010595	0.000501128	0.006621303	0.020701657	0.024381051	0.002862552	0.010269544	0.001388133
0.008149229	0.003730023	0.001379774	0.018060003	0.002744435	0.009101485	0.003881082	0.004333004	0.007058175	0.017516383	0.00910823
0.000350417	0.00019275	5.65E-05	0.001818421	0.000464132	0.001155922	0.000216892	0.000384183	0.001652758	0.001721794	0.002951947
0.001225101	0.00103001	0.001317126	0.003003376	0.002102049	0.000411493	0.004419483	0.000880217	0.001548285	0.003981808	0.000386005
0.023518674	0.012314948	0.006684952	0.056112702	0.008764702	0.019953688	0.014710348	0.016663344	0.01250747	0.051786457	0.009573415
0.001007788	0.001347747	0.002360743	0.005840312	0.001079612	0.000549905	0.006690465	0.003309326	0.000543258	0.011044989	0.000571584
0.001062116	0.000894483	0.000773162	0.005070215	0.001890162	0.001331742	0.001653479	0.002599316	0.004187265	0.003423181	0.001261939
0.274368231	0.076216248	0.020647715	0.240036567	0.181556458	0.111597006	0.042551563	0.066449125	0.3817411	0.032362072	0.343554214
0.002719126	0.000173174	0.001274343	0.001177502	9.42E-05	0.000456384	7.40E-05	0.001743414	0.001552464	0.000826461	0.001160489
0.002205725	0.003806822	0	0.002196017	0.005132362	0.013212678	0	0.002098419	0.001592164	0.001364681	0.00555748
0.0038736	0.000281596	0	0.008093465	0.002946231	0.003501435	0.001081906	0.012857983	0.004615603	0.003137491	0.003199386
0.000646505	0.004058301	0.008492563	0.012348869	0.025853516	0.021176946	0.000418473	0.032200884	0.009972962	0.032981917	0.003008858
0.078409162	0.10911785	0.039084429	0.106983781	0.168698647	0.151134038	0.020089257	0.223281023	0.083145631	0.030494882	0.130390459
0.000179283	0.001501345	0.009985408	0.006873732	0.000941718	0.021375211	0.019479409	0.028580321	0.009728496	0.134542245	0.000626021
0	0.000162633	0.001043616	0.002178628	0.000726468	0.000127189	0	0.001402999	0	0.000278038	0.000170733
0.00147501	0.001356783	0	0.000402437	0.005186174	0.001283111	0.005059951	0.00742106	0.002613907	0.010810314	0.000826446
0.002797902	0.004663658	0.002762604	0.026764577	0.01331858	0.028415489	0.00657564	0.02884779	0.010942469	0.067751949	0.023246895
0.000464506	0.000459288	0.000113071	0.002282963	0.000723105	0.002880454	0.000678743	0.002356162	0.000704146	0.005453623	0.001895383
0.000426476	0.000176186	0.001129184	0	0.00057512	0.000392789	0.001400864	0.000398773	0.001659026	0.00417822	0.000539417
0.000277074	0.000147575	0.000256702	0.000998641	0.000501128	0.000905285	0.000806326	0.000928848	0.000369833	0.002698752	0.000482506
0.000285223	0.001227278	0.011038192	0	0.001587467	0.000127189	0.000193927	8.27E-05	0	8.93E-05	0
0.001662443	0.012963975	0.075051761	0.001430889	0.009955302	0.000815505	0.002186777	0.001235222	0.000440875	0.002165634	0.0002029
0.000154835	0.001005917	0.006400746	0.000404922	0.001227596	0.000149634	0	7.05E-05	0	0.000224471	0
0	0.000140045	0.002154464	0	0.000403593	0	0.000102067	0	0	0	0
0.000133104	0.002150372	0.012408798	9.44E-05	0.002546001	0	0.000178617	0.001991431	0.000221482	0.000104583	0
0.000138537	0.001323654	0.008156405	0.000263323	0.00139576	0.000115966	7.65E-05	0.001106351	6.69E-05	8.42E-05	0.000138566
8.15E-05	0.002233195	0.01798443	0.000300586	0.003010134	0.000303009	0	0.000418225	7.94E-05	6.12E-05	0
4.35E-05	0.000944176	0.005898037	0.00019625	0.00062557	0	0	0	0	0	0
0	0.001087233	0.004793301	0	0.001076249	0	0	0.001001794	0	0	0
0.000138537	0.000224374	0.002174328	0.00016644	0.000847546	0	0	0.000277196	0	0	0
0.006557413	0.03499927	0.002501318	0.01286061	0.062839481	0.081052974	0.01367182	0.003083193	0.021774197	0.000214268	0.10026476
0.108069638	0.022303338	0.008324484	0.093064909	0.031328932	0.02895043	0.010745061	0.005072192	0.014039039	0.011269459	0.024588014
0.194418865	0.179185659	0.088216913	0.161717762	0.055705969	0.155166674	0.363811269	0.026530533	0.041310589	0.078498494	0.150821498
0.002395873	0.003549319	0.000705931	0.001314132	0.001987697	0.004384275	0.001079354	0.000294216	0.002852104	0.000216818	0.002165091
0.000130388	0.00452813	0.000308654	0.000407406	0.007271406	0.001530007	0.001457001	0.000500897	0.003190596	0.000214268	0.000638393
0.000429193	0.00070625	0.001118488	0	0.011620125	0.00070328	0.000273028	0.003642447	0.011995554	0.000150498	0.004055525
0.021071189	0.007941922	0.00444492	0.010942822	0.022211086	0.032339639	9.44E-05	0.040558087	0.00489559	0.002974239	0.000497352
0.002591455	0.03315007	0.001877898	0.000161472	0.014092134	0.003826888	0.002699661	0.002285648	0.016799208	0.002272768	0.004082744
6.25E-05	0.055093507	0.001350742	0.000158988	0.02155861	0.001597343	0.004554722	0.002847333	0.01082546	0.001191226	0.001489583
0.000752445	0.006389377	0.008941791	0.001296743	0.00803487	0.011054209	0.007422793	0.004031493	0.003529087	0.001415697	0.000470134
8.96E-05	0.008679795	0.000545492	0.000340333	0.002044873	0.005592569	0.000724673	0.009259304	0.015127645	0.005652585	0.001657841
0.00017385	0.008097026	0	0.000491868	0.001072886	0.008798477	0	0.006477623	0.013593986	0.004545536	0.008974613
0.002778887	0.134452491	0	0.013352478	0.032509442	0	0.000204133	0.00742106	0.056116458	0.003231871	0.005451081
0	0.008407234	0.000226142	0	0.007463113	0.010545453	0.000283235	0.05864145	0.014126796	7.40E-05	0.018429257

0.000301521	0.002346135	0.00082817	0.001207312	0.007150328	0.000404012	0.000234753	0.006001041	0.014072471	6.38E-05	0.002484288
0.001901487	0.012530287	0.017419074	0.008187864	0.001335221	0.001208294	0.006598605	0.001082035	0.00026745	0.00918035	9.16E-05
0.000149403	0.00030569	0	0.000618561	0.000568394	0.000785578	8.68E-05	0.000160482	0	0.001571296	8.17E-05
0.011213339	0.00322857	0.005297538	0.005107478	0.01025127	0.003280725	0.002903795	0.00307833	0.018343314	0.002742116	0.021532142
0.000540566	0.00034936	0.000927489	0.002315258	0.002371111	0.001694605	0.000196478	0.002450992	0.004544562	0.004443503	0.000544366
0.001024086	0.001264925	0	0.004324961	0.004130105	0	0	0.002069241	0.001558732	0.007040224	0.002838125
0.027348811	0.002663872	0.012576877	0.023696612	0.033390621	0.012774999	0.000607296	0.067737841	0.042580977	0.032573788	0.020017816
0.009613373	0.001847694	0	0.001808484	0.011401511	0.000931471	0.000112273	0.002390204	0.009128823	0.002479383	0.007024793
0.007608663	0.002370228	0.056602822	0.00019625	7.74E-05	0.001372892	0	0.006601631	0.002269147	0.007517224	0.002647597
0.000244477	0.001516404	0.002589941	0.000571362	0.00038005	0.000160857	0	0.000155619	0.000965328	0.002022789	0.000249913
0.03127674	0.00888911	0.046739654	0.038045247	0.027393897	0.020937532	0.016356172	0.025144555	0.034657768	0.017126109	0.025206612
0.000926296	0	0.004139322	0.001088072	0.002855423	0.000688316	0.000607296	0.001128235	0.000808619	0.000484653	0.001548968
0.000377581	0.001737766	0.001278927	0.000857043	0.000329601	0.060800018	0.001056389	0.000340416	0.000234019	0.001918206	0
6.25E-05	0.000414112	0.02736017	0.003055544	0.000965261	0.001159663	0.001819337	0.007306778	0.000501469	0.010167511	0.000249913
0.001111012	0.001084221	0.021430045	0.000628498	0.001160331	0.001440227	0.001038528	0.010445896	0.000380281	0.006134688	0.000106399
0.000116806	0.000204797	5.65E-05	0.000154019	0	0.00039653	0.000191375	0.000141029	0.00015253	0.000142845	0
0	0	0	0	0	0.000583572	0.000523091	0.000318532	0	0.000150498	0
0	0	0	0	0	0.000280564	0.002128088	0.000649221	0	0.000270385	0
0.000152119	0	0	0	0	0	0	0	0	0	0
0	0	0	0	0	0	0.000939013	0	0.000173425	8.93E-05	7.92E-05
0	0	0	0	0	0	0.000847153	0	0	0	0
7.61E-05	0	0	0	0	0	9.95E-05	9.48E-05	0	0.000469348	0
0.000502536	2.26E-05	0	0.000832201	0	0.001582379	0.000181168	0.001889307	0.00034476	0.00164527	0.000522096
0.001781965	0	0	0.000305554	0	0.00031049	0.001663685	0.002618769	0.000679072	0.005828591	0.00044539
0.001219668	0	0	0.000156503	0	0.000149634	0.000696604	0.000483876	0.000409533	0.001941163	6.93E-05
7.61E-05	0	0	0	0	0.000127189	0.000308751	0.002331847	0	0.008101359	0
5.70E-05	0	0	0.000295618	0	0	0	0	0	0.000650455	9.65E-05
0	0	0	0	0	0	0	0	0	0	0
0.000124955	0	0	0.000104336	0	0	0.000561366	0	0	0.000288241	0
8.15E-05	0	0	0	0	0.000261859	0.000186272	7.54E-05	0	0.00053822	8.91E-05
0.000228178	0	0	0	0	0	0.000119928	0	0	0.000497407	0
0	0	0	0.000109304	0	0.000138411	0.000387853	0	7.73E-05	0.000339257	0
0.000187432	0	0	0.000171409	0	0.000123448	0.000252615	0	7.73E-05	0.000517814	0
0.000184716	0	0	8.69E-05	0	0	0.00044399	8.51E-05	7.10E-05	0.000770343	0
0.000336835	0	0	0.000158988	0	0	0.000201582	0	0	0.000670862	8.41E-05
0.000184716	0.000554158	0.00611654	0.000263323	0.000776917	0.00013093	9.95E-05	7.29E-05	0	0.000257631	0
0	0	0	0	0	0	0	0	0	0	0
0	0	0	0	0	0	0	0	0	0	0
0	0	0	0	0	0	0	0	0	0	0
0	0	0	0.000270776	0	0.000280564	0.000880324	0	0	0	0
5.70E-05	0.011068094	0.001980274	0.003338741	0.009588705	0.000299268	0.007956091	0.000593296	0.001013385	0.00326248	0.006411145
0	0.003371627	0.000799138	0.007010362	0.00080046	0.001066142	0.007040043	0.000179934	0.000158798	0.001943714	0.000729945
0.004525538	0.015557071	0.008886784	0.006446452	0.019806342	0.003995227	0.061579327	0.003700804	0.0126997	0.003907834	0.007838868
0.106415344	0.132050639	0.362906541	0.05742435	0.113137299	0.063355018	0.254025889	0.095075645	0.06069654	0.192682759	0.026282971
0.012916527	0.033475336	0.038997334	0.004347318	0.010093196	0.010077847	0.021469708	0.009988766	0.005305123	0.025533565	0.002528827
0.001230534	0.000415618	0.001810667	0.000621045	0.000279152	0.004919216	0.003146203	0.000335553	0	0.004940911	7.42E-05
8.96E-05	0.000664086	0.00113988	0.000101851	0	0.000145893	0	0.000238291	0	0.000219369	0

H075	H076	H077	H078	H079	H080	H081	H082	H083	H084	H085
0.000648167	0.002019855	0.000715103	0.01258116	0.019192669	0.012654137	0.015749301	0.002627953	0.002584129	4.03E-05	0.010358045
0.002042339	0.024399851	0.005828884	0.006442504	0.013299015	0.019003677	0.00285009	0.008926659	0.008658965	0.001631387	0.008366967
5.30E-05	0.006594827	0.000505339	0.003970593	0.020724353	0.006888833	0.000490533	0.003372811	0.001413862	0.000555405	0.033014486
0.002248203	0.074950093	0.001922832	0.01618622	0.066897973	0.01277612	0.008529831	0.009427111	0.003498242	0.00126295	0.005104516
0.000201788	0.047634918	0.000556191	0.016791633	0.010605248	0.014132914	0.000174866	0.000800723	7.28E-05	0.000450923	0.076127036
0.002531522	0.00990739	0.016164505	0.010090972	0.015966143	0.020469613	0.024578902	0.003696359	0.014040683	0.003083138	0.020072478
0.000654282	0.001188348	0.004064963	0.001583211	0.001698172	0.001326833	0.002075683	0.000621491	0.002066801	0.000911011	0.001192111
0	0.002036687	0.001401602	0.001905336	0.017001695	0.007479488	0	0.005595747	0.00216223	0.000137476	0.003741198
0.001975076	0.021922162	0.02040745	0.031853842	0.033294153	0.050197099	0.00931105	0.016119196	0.017870417	0.002412253	0.052909415
0.000572752	0.017151937	0.004424104	0.002736922	0.007828238	0.001521578	0.002155168	0.001878439	0.008299849	0.0007772	0.00149965
0.002992169	0.002901859	0.000772311	0.003413156	0.003769275	0.00159006	0.002255091	0.002704766	0.002772476	0.00056457	0.002048147
0.062654144	0.18674571	0.219482583	0.178283735	0.143185837	0.047725337	0.181265712	0.227323725	0.178329985	0.089671303	0.026575187
0.000163061	0.000966164	0.000667429	0.00061912	0.000376262	0	0	0.000111729	0.001067303	0.000542574	0.000339244
0	0.004107039	0.016008772	0.004425224	0.003033401	0	0.005002986	0.007928084	0.000165746	0.000483917	7.61E-05
0.001271876	0.003218303	0.002459954	0.006604709	0.01085165	0.010805131	0.006517735	0.007162277	0.004944751	0.001052153	0.001547207
0.003601609	0.025069769	0.02265764	0.030672716	0.040503058	0.001076447	0.00101059	0.039202816	0.004892014	0.004967482	0.01332881
0.041417489	0.115350563	0.172018815	0.118420993	0.178328	0.143233792	0.07825143	0.204735901	0.042619287	0.024789753	0.013417584
0.0004688	0.092388176	0.00178299	0.054944051	0.007515242	0.002854832	0.000638148	0.002807184	0.005394274	0.007071788	0.021014118
4.89E-05	0.002093917	0.000435418	0.000742487	0.001178731	0.000239686	0.000265706	0.000225785	0.000610246	0.000284118	0.000798968
0.000450456	0.004352788	0.001709891	0.006172924	0.006493009	0.001953869	0.000186221	0.002350959	0.003395279	0.000626893	0.004904774
0.007172645	0.016047749	0.011505212	0.066184164	0.059199595	0.022975616	0.00301133	0.046239397	0.018086389	0.001829353	0.014352884
0.000733774	0.001757274	0.000543478	0.004813601	0.005537371	0.0024097	0.000186221	0.004704245	0.001579608	0.000124645	0.001433069
7.95E-05	0.00125231	0.000645182	0.00231656	0.004042314	0.000873142	0.000476907	0.000197853	0.001722752	0.000223628	0.002108387
0.000226247	0.001349937	0.000117595	0.001658602	0.000945649	0	0.000190763	0.000114056	0.001531894	0.000119146	0.000488258
0.004478062	8.75E-05	0	0	0	0.00038949	0.001228604	0.000169921	0.000178302	0.000931174	0.000405825
0.077943149	0.00112102	0.000527587	0.000306133	0.000472824	0.002732849	0.018526719	0.002076292	0.002147162	0.0106975	0.005018912
0.006616199	0	0	0	0	0.000235406	0.001346696	0.000225785	0.000306379	0.001233622	0
0.000122296	0	0	0	0	7.28E-05	0.000806201	4.19E-05	0.000107986	0.000185135	0
0.00091518	8.42E-05	0.000114416	9.60E-05	0.000239742	0	0.003265681	0.000593559	0.000778503	0.001569065	0.000510452
0.0006339	0	0.000136664	0	0.000356283	0	0.002704747	0.000281649	0.00283777	0.000920176	8.24E-05
0.001597998	7.07E-05	0	8.00E-05	0	0	0.006479128	0.000432949	0.000185836	0.001624055	0.000424848
0.000338352	8.75E-05	0	0	0	7.70E-05	0.00234366	0.000109401	0.000165746	0.000784532	7.93E-05
0.000497336	0	0.000146199	0	0	8.99E-05	0.001660092	0.000148972	0.00025113	0.000450923	0.000149014
0.000171214	0	0	0	0	0	0.000769865	0.00013035	0.000399297	0.000353773	0.000110968
0.072918832	0.016906188	0.049777524	0.008420947	0.00860074	0.000361669	0.044847469	0.053836951	0.086983928	0.030836883	0.000707023
0.040096695	0.027342106	0.006524917	0.017470152	0.008823833	0.002129353	0.186252802	0.047619713	0.056336012	0.007804996	0.001924498
0.163658192	0.049149809	0.085821892	0.064701474	0.026667954	0.116630785	0.147988927	0.045745929	0.187827725	0.117348061	0.005735447
0.005105847	0.001326372	0.002625222	0.003113877	0.000576046	0.001635001	0.001212708	0.001508338	0.000971873	0.002162963	0
0.00058702	0.003262066	0.002221587	0.000925253	0.00119538	0.001307573	0.004955296	0.000256045	0.004412356	0.003433245	0.00106212
0.000301663	0.003241868	0.003956903	0	0.000119871	0.000633456	8.40E-05	0.000118712	0.000459568	0.003708198	0.000279005
0.145937539	0.003322662	0.006010043	0.001708863	0.00026971	0.112909232	0.008021129	0.002020428	0.000273732	0.251717539	0.00094164
0.011287896	0.00014139	0.004198449	0.006830882	0.001238666	0.045895591	0	0.000726237	0.006818182	0.021822094	0.000234617
0.010423673	0.000521796	0.002167557	0.000986937	0.002477333	0.001275472	0.000669941	0.000577265	0.004206429	0.003154626	0
0.003128733	0.005685892	0.004201627	0.000970945	0.001894627	0.001823326	0.002495816	0.000945039	0.025610246	0.004186616	0.002181309
0.000444341	0.005978771	0.009054793	0.0030956	0.002710415	0.003631671	0.001616943	0.006028696	0.001388749	0.002172128	0.000212424
0.003016628	0.007187318	0.001096491	0.012400678	0.011444345	0	0.003404211	0	0	0.000562737	0.000396313
0.013385268	0.015593282	0.015331808	0.012169936	0.000805799	0.008609436	0	0.025460183	0.035248619	0.014194901	0.000592885
0.019736493	0.012236956	0.005768497	0.06841391	0.05357232	0	0.001467058	0.010621212	0.012325465	0.006158945	0.000447041

0.000536063	0.067372269	0.009661836	0.031696206	0	0.001446676	0.000190763	0.003291342	0.002910598	0.001658882	0.001823041
0.014573575	0.000541994	0.004351004	0.000390662	0.003476257	0.008249907	0.016818936	0.004729849	0.008955299	0.009216421	0.048343886
0.000383193	9.76E-05	0.000266972	0.000150782	0	0.000243966	0.000887956	0.001571185	0.000911602	0.000850521	0
0.001487931	0.006126894	0.014422832	0.006421943	0.000719226	0.001949589	0.000924292	0.010286026	0.005665495	0.005768512	0.002843944
0.002303236	0.003359692	0.001179125	0.000253588	0.001495057	0.000742599	0.001167288	0.005146504	0.001361125	0.0001943	0.029942265
0.005012087	0.001043592	0.011597381	0.004007146	0.00238077	0.002841991	0.000361087	0.000507435	0.005703164	0.002826516	0.001797677
0.006599893	0.013421938	0.063370837	0.032648874	0.006153375	0.009807866	0.000999235	0.018686629	0.008588649	0.003743025	0.014159483
0.001463472	0.004628835	0.005174167	0.000726495	0.003000103	0.001814766	0.000565476	0.002988743	0.004708689	0.002283942	0.00244129
0.007258252	0	0.00691584	0.004674242	0.003499565	0.009621681	0	0.003256427	0.008440482	0.024555126	0.009482985
0.001736599	0	0.000127129	0.000187335	0	0.000967304	0	0	0.000389252	0.000221795	0.002472995
0.012482318	0.025089968	0.03456331	0.013526974	0.040120137	0.010920694	0.033481175	0.026160815	0.024909593	0.030813054	0.024248035
0.000254783	0	0.00032418	0.00067395	0.000216434	0.000160504	9.08E-05	0.0002607	0.000668006	0.004083967	0.001109677
0.000911103	0.000952698	0.000959827	0	0.000229753	0.000995125	0.00266614	0.030906958	0.001818182	0.000196133	0.007298506
0.006173896	0.005773419	0.001700356	0.000705934	0.014267971	0.021199372	8.86E-05	0.000311909	0.001182823	0.000172304	0.218413035
0.002303236	0.001434097	0.001906941	0.000980083	0.012163571	0.005686123	0.003031769	0.001000903	0.00221999	0.001072316	0.092290279
0	0.001245577	0.000289219	0.000235311	0.000206444	0.000194745	7.95E-05	0.000137333	6.78E-05	4.40E-05	0
0	0.000356841	0	0.001274793	0.000909021	0	0	5.35E-05	0	0	0
0	7.07E-05	0.000152555	0.000641966	0.000246401	0	0	0	0	0	0
0	0	0	0	0	0	0	0	4.77E-05	0	0
0	0.000111092	0.000139842	0.0004592	0	7.70E-05	0	0	0.000246107	0	0
0	0	0	9.60E-05	0.000123201	8.56E-05	0	0	0	0	0
0	0	0	0	0	0.000100583	0	0	5.52E-05	0	0
0	0.004006046	0.003594584	0.007778981	0.002603863	0.001876827	0	0.000216474	0.0014666	0	0
0	0.006857408	0	0.014481927	0.007661751	0.001688502	0	0.000123367	0.002405826	0	0
0	0.000848339	0	0.010753499	0.001971211	0.000819641	0	0	0.001250628	0	8.56E-05
0	0.001841435	0	0.001528381	0.003872497	0.000164784	0	0	8.29E-05	0	0
0	0.000154856	0.000187516	0.000719641	0.001348548	0.000151944	0	0	0.000233551	0	0
0	0	0	0	0	0	0	0	0	0	0
0	0	0.000251081	0.000187335	8.32E-05	8.99E-05	0	0	0.000155701	0	0
0	0	0	0.000559721	0.000206444	0.000248246	0	0	0.000570065	0	0
0	0	0	9.82E-05	0	0.000173344	0	0	0.000248619	0	0
0	0	0	0.000205612	0	0	0	0	0.000130588	0	0
0	0.00018852	0.000317824	0.000870423	0.000352953	8.99E-05	0	0	0.00017328	0	0
0	0.000117825	0	0.000845293	0.000143179	0	0	0	0.000246107	0	0
0	0.000158222	0.000133486	0.000347256	0	0.000231126	0	0	0	0	0
0.005968032	0	0	0	0	0.000237546	0.000844807	9.54E-05	0.000138122	0.000579234	0.0004407
0	0	0	0	0	0	0	0	0	0	0
0	0	0	0	0	0	0	0	0	0	0
0	0	0	0	0	0	0	0	0	0	0
0	0	0.000333715	0	0	0	0	0.000148972	0	0	0
0.000994672	0.000333276	0.002243834	0.000719641	0	0.001793365	0.003193009	0.005588764	0.002061778	0.005845498	0
0.000811228	0	0.000451309	0.000121083	0	0.001311853	0.003065834	0.008065417	0.001325967	0.000762536	0
0.056337569	0.003844458	0.001217264	0.009949328	0.003735978	0.018866713	0.014995333	0.003314619	0.016049724	0.023589125	0.0025047
0.141221001	0.025719489	0.103372108	0.061962268	0.077206874	0.175266116	0.10622773	0.054784317	0.122918132	0.194333038	0.139644967
0.015203398	0.002518086	0.015989703	0.003822096	0.016905132	0.031411708	0.021335931	0.00851466	0.010818684	0.055183045	0.030481251
0.000324084	0.00194916	0.0081776	0.000191904	0.001638236	0.005435736	0.001321715	0.011163562	0.002820191	0.000379435	0.025722321
0.000110066	0.001080623	0.000146199	0	0.00012986	0.00040875	0.000340648	0.000137333	5.27E-05	0	0.008138691

H086	H087	H088	H089	H090	H091	H092	H093	H094	H095	H096
0.014205615	0.000328389	0.007173993	0.001086669	0.017296721	0.016655842	0.00168143	0.01000084	0.011901763	0.026864048	0.000869689
0.013962404	0.00365124	0.006111554	0.008059465	0.00481297	0.012345473	0.017464948	0.007777829	0.04572838	0.022133583	0.002565218
0.004032596	0.000153063	0.004146548	0.003005949	0.004345962	0.011845881	0.005772733	0.003819784	0.011041142	0.007292309	0.001580422
0.005321875	0.012782122	0.005476114	0.002933001	0.06183325	0.013677719	0.007989887	0.001653704	0.035418416	0.022493843	0.002936114
0.001320662	0.000289428	4.65E-05	0.00255317	0.05365364	0.00048849	0.002181792	0.002315185	0.014847467	0.010791299	0.000314257
0.015337985	0.000812623	0.005429569	0.003408419	0.011528824	0.017699434	0.006773458	0.014430597	0.027672824	0.015971499	0.001211353
0.001098372	0.000428575	0.000254985	0.00040247	0.001191916	0.000952	0.001506392	0.001954623	0.003732858	0.002114464	7.86E-05
0.003927989	0	0.003290525	0.004864858	0.001411479	0.003347266	0.000507435	0.00274081	0.016554716	0.001660019	0.000767373
0.020034834	0.003821	0.046225205	0.031598938	0.043173842	0.019750537	0.007424106	0.017857288	0.045563952	0.028427527	0.009393008
0.004767459	0.002148443	0.002458787	0.002422367	0.014128734	0.002439674	0.001143938	0.001848895	0.01374895	0.004671599	0.001730243
0.006998201	0.001984248	0.001833466	0.005383038	0.001355717	0.002178776	0.003976379	0.00145309	0.001444864	0.00356492	0.002113929
0.167606385	0.140160855	0.047447516	0.442027947	0.025643617	0.058629897	0.16047667	0.177483063	0.084228939	0.236808149	0.096155462
0.001791393	0.000979601	0.000313672	0.000701807	0	0.000957551	0.000717834	0.000241278	0.000402323	0.002196876	0.000548123
0.006632077	0.010288593	0.000396644	0.002487769	0.001759993	0.000105469	0.009786241	0.004077328	0.003078645	0.000706391	0.000385513
0.005397715	0.001171625	0.00219166	0.001994743	0.009270457	0.001998368	0.002061564	0.002363983	0.005758466	0.009769387	0.000511582
0.048725888	0.012587315	0.004472362	0.023775925	0.024026515	0.03345046	0.024192436	0.001827207	0.001427372	0.011372894	0.036497711
0.129165969	0.268338296	0.111428606	0.100544592	0.154753897	0.126568858	0.195410103	0.081272755	0.155737476	0.029656647	0.024174069
0.002031989	0.000102969	0.008799019	0.002671396	0.091519623	0.010472003	0.012905108	0.01476676	0.071134201	0.02283291	0.007089063
0.002086907	0	0.000736624	0.002107937	0.000526255	8.60E-05	0.000153822	0.000891916	0.000647215	0.000525084	0.000438499
0.007798443	0.003186486	0.001586576	0.006364059	0	0.000366367	0.002551318	0.003283008	0.008130423	0.00602316	0.0020445
0.038550253	0.00668188	0.017958255	0.047823517	0.100225488	0.014737964	0.018669001	0.015937907	0.089634061	0.039722718	0.010079989
0.003904452	0.000748615	0.001691808	0.001546995	0.007472128	0.001423837	0.001904206	0.00095969	0.0047719	0.003291782	0.00072535
0.000959768	0.000242117	0.002373792	0.001038876	0	0.001745796	0.001923655	0.001556108	0.007024909	0.001504613	0.001061532
0.001618792	0.00059277	0.000566634	0.000767209	0.000153346	0.000527347	0.001253558	0.000824141	0.000997061	0.000485055	0.000436672
0.000149065	0.000178109	0	0	0	0.001842939	6.37E-05	7.32E-05	0	6.36E-05	0.001468971
0.002149672	0.004352545	0.003960368	0.000691746	0.000285781	0.027122295	0.001897134	0.000539487	0.001154492	0.000694618	0.013085533
0.000201368	0.000205939	0.000271175	0	0.000188197	0.002556246	0.000291731	0	0.00040932	7.77E-05	0.001059705
0	0	0	0	0	0.000618939	0	0	0	0.000209563	0.000330701
7.58E-05	0.000740267	0	0.000120741	0	0.005431675	0.000199791	0.0002467	0	0.00265603	0.001812461
0.000219674	0.000570506	0.000167967	0.000166019	0.000365939	0.003486042	0	9.22E-05	0.000293871	0.002338154	0.000791125
0.000185677	0.000567723	0.000190227	0	0.00052974	0.007760329	0.000150286	0.000721123	0.000108452	0.002867947	0.001671776
8.37E-05	0.00015028	0	0	0	0.003677552	8.66E-05	0.000184347	0	0.000831187	0.000506101
6.54E-05	0.000436924	5.06E-05	0	0	0.002722776	0	0	0	0.001127871	0.000617552
0	4.73E-05	0	0	0	0.001476572	7.43E-05	0	0.000101455	0.000623979	0.000191843
0.019038453	0.066721398	0.005935493	0.025257518	0.001097817	0.04436377	0.0221238	0.152604312	0.011982228	0.031608641	0.040520936
0.024582619	0.051003256	0.007740627	0.013445019	0.010873619	0.056192998	0.03249527	0.034453966	0.024160369	0.053838764	0.020055836
0.055104293	0.117184761	0.050149146	0.033055377	0.127974126	0.055923773	0.064649304	0.138924659	0.065897005	0.107540959	0.286109457
0.001882924	0.005145688	0.000943041	0.00191928	0.000320632	0.003622042	0.001587723	0.00131483	0.001714246	0.005161363	0.003409328
0.001542952	0.000119667	0.001023989	0.002958156	0.000160316	0.003008654	0.005496915	0.000338874	0.001325917	0.000506247	0.000297814
0.000292899	0.000128016	0.000392597	0.007654479	0.000160316	0.000113796	0.003926873	0	0.000388329	0	4.38E-05
0.00121867	0.023849943	0.044608274	0.00043014	0.044477282	0	0.000369526	0.004058351	0.000566751	0.00265603	0.006029357
0.001924767	0.020003896	0.029250464	0.000296822	0.000146376	0.003402777	0.009851659	0.002952268	0.004520011	0.002295771	0.003212003
0.000538725	0.010770044	0.002608541	0.00019872	0.001536944	0.000793796	0.006810587	0.000742811	0.001007557	0.001214992	0.003113341
0.005562471	8.63E-05	0.00687651	0.001214957	0.017077157	0.008318207	0.002593752	0.000181636	0.009564791	0.000595723	0.002033538
0.000931001	0.000556591	0.002649015	0.001989712	0.027034186	0.001065796	0.020228434	0.002187769	0.010288973	8.71E-05	0.000407438
0.00683083	0.036929842	0.000431047	0.000465356	0	0.00472392	0.02251631	0.012814848	0.000479289	0.002674867	0.010231637
0.016899243	0.006687446	0.002031788	0.003848621	0.000195168	0.008085064	0.019535352	0.015043281	0.002455919	0.054997245	0.019211725
0.013907486	0.04868227	0.000283317	0.010866694	0	0.004141063	0.00753903	0.018898308	0.033672684	0.064312187	0.037433175

0.01237238	0.006676314	5.46E-05	0.017638255	0	0.000388572	0.011305009	0.003147459	0.00127344	0.01615987	0.002673015
0.00144096	0.002919322	0.005907161	0.001642581	0.001519518	0.00562041	0.000235153	0.003198968	0.003722362	0.001106679	0.007659111
0.000651178	0.000108535	0.000157848	0	0.000832947	0.001623674	0.000252833	0	0.000104954	0.000190726	0.000577357
0.023641157	0.005616008	0.004106074	0.024183426	0.000940986	0.012764576	0.006009654	0.010404778	0.002263504	0.003254108	0.005128608
0.006362714	0.000381265	0.001193979	0.004150474	0.001421935	0.025440335	0.00048445	0.000276521	0.000913098	0.003571984	0.001759476
0.007123729	0.001641945	0.048303538	0.002080268	0.000738848	0.004662859	0.005489842	0.001591351	0.001917156	0.001478712	0.003623096
0.050632349	0.004422119	0.03366414	0.026638494	0.000526255	0.026725396	0.025859722	0.001924803	0.0072733	0.020122724	0.013631829
0.021951755	0.004313584	0.008335593	0.006270988	0	0.013178127	0.012268604	0.001472067	0.000731178	0.009529214	0.003765608
0.040998054	0.000798709	0.126841055	0.00312669	0.001139639	0	0.025691756	0.002133549	0.009645256	0.002477078	0.002287502
0.001014687	6.96E-05	0.002353555	8.80E-05	0	0.002381389	0.000742587	0	0.000601735	0	0.00171928
0.041942131	0.015303481	0.07647335	0.062400483	0.004861762	0.059901081	0.04305062	0.016293047	0.02774979	0.024205663	0.048762885
0.006187497	0.000230985	0.003689193	0.000445233	0	0.002159348	0.002800615	0.000490689	0.000444305	7.53E-05	0.000160783
0.002931608	0.000626165	0.001730258	0.006235772	0.001892428	0.012870045	0.000293499	0.000376828	0.001290932	0.000376742	0.000129723
0.000162141	0.000968469	0.002709725	0.000372285	0.000975838	0.024940743	0.000495058	0.001702501	0.002144556	0.002192167	0.000608417
0.000135989	0.000620599	0.006947339	0.000382347	0.002840384	0.011146453	0.005233473	0.001932936	0.001315421	0.000915954	0.001839868
0.000149065	6.12E-05	0	0.003164422	0.000487919	0.000321959	0.000537492	0.00019248	0.000941086	7.77E-05	0
0	0	0	0.000166019	0	0	0	0.00052051	0.000444305	0.000273138	0
0	0	0.000101185	0.000213812	0	0	6.90E-05	0.00052051	0.000241394	6.83E-05	0
0.000130759	0	0	0.000206266	0	0	7.25E-05	8.68E-05	0	0	0
8.89E-05	0	0.000368312	8.30E-05	0	0	0.000357149	0.000238567	9.80E-05	0	0
0	0	8.30E-05	0	0	0	7.07E-05	0	0	0	0
0	0	0	0	0.000163801	0	7.96E-05	0.000523221	0	0	0
0.000891774	0	0.001171718	0.001999774	0.006395221	0	0.001532913	0.006753075	0.010299468	0.001356271	0
0.000706096	0	0.000684008	8.30E-05	0.019415682	9.71E-05	0.000746124	0.00449211	0.011705849	0.003183469	0
0.000630256	0	0.000477592	0	0.003425887	0	0.000779717	0.003651703	0.007101875	0.001605862	0
0	0	5.87E-05	0	0.007472128	0	0	0.00013826	0.002623846	6.36E-05	0
0.000138604	0	8.09E-05	9.31E-05	0.000156831	0	6.54E-05	0.000384961	0.000377834	0.00039087	0
0	0	0	0	0	0	0	0	0	0	0
0	0	0.000184156	0	0.00013592	0	0	0.000463579	0.000349846	0	0
0.000279823	0	0.000269151	9.81E-05	0	0	0.000152054	0.000748233	0.000577246	0.000113023	0
0	0	0.000273199	7.80E-05	0.000174257	0	0	0.000452735	0.000482788	0	0
7.58E-05	0	0	9.56E-05	0.000965382	0	0	8.95E-05	0.000843129	0	0
0.000198753	0	0.000165943	0.000467872	0.000494889	0	7.07E-05	0.000314475	0.000342849	0.000134214	0
0	0	0.00048771	9.56E-05	0.000268355	0	6.01E-05	0.000474423	0.00059124	0	0
7.85E-05	0	0.000675914	9.06E-05	0	0	7.43E-05	0.000309053	0.000668206	0	0
0.000507343	8.63E-05	0.000463426	0	0	0.00162645	0	0	0.000157431	0.000155406	0.000950081
0	0	0	0	0	0	0	0	0	0	0
0	0	0	0	0	0	0	0	0	0	0
0	0	0	0	0	0	0	0	0	0	0
7.32E-05	0	0.00014773	0	0.000167286	0	0	0	0.000125945	0	0
0.0031199	0.003539922	0.001837514	0.000327007	0.000934016	0.004787757	0.000369526	0.000276521	0.000724181	0.001017203	0.001399542
0.000528265	0.000445273	0.001859774	0.000301853	0.000303207	0.003644246	0.000496826	0.000271099	9.45E-05	0	0.001401369
0.009231558	0.013360977	0.014774986	0.002221132	0.000749304	0.013219759	0.025138351	0.006452155	0.00425063	0.009376163	0.01951502
0.093965752	0.064405978	0.192226589	0.026608308	0.067256119	0.106679545	0.107424106	0.154152287	0.058231878	0.040480911	0.199913397
0.010698669	0.006598391	0.016327158	0.002754405	0.009674732	0.034199848	0.010314892	0.015420108	0.006405682	0.005337961	0.024091851
0.000292899	0.000205939	0.001446941	0.000586097	0.00116055	0.026544988	7.25E-05	0.000347007	0.000101455	0.000847669	0.000482349
0	0	0.001392301	0.000460325	0	0.002173225	0.000387206	7.05E-05	0.000108452	0	0.000566394

H097	H098	H099	H100	H101	H102	H103	H104	H105	H106	H107
0.002606801	0.023306959	0.001353693	0.055738921	0.010570226	0.000407951	0.008104432	0.006101495	0.014131584	0.006000691	0.056262039
0.011311656	0.005851814	0.006145834	0.004499185	0.004008619	0.00202645	0.008787313	0.004538954	0.001327503	0.021550456	0.00605273
0.005570486	0.014185496	0.004172793	0.007663502	0.000506088	0.002217123	0.002269896	0.000273556	0.00206079	0.004518644	0.001916666
0.015178411	0.034312161	0.027409008	0.032647479	0.002129579	0.00476238	0.007610026	0.002467954	0.004583046	0.01262832	0.013345314
0.037612418	0.010751727	0.008818722	0.012806149	4.26E-05	0.000365825	0.001638914	0.000133805	0.000635305	0.00282975	0.004006872
0.014557744	0.006125348	0.005125636	0.012266146	0.002164654	0.001199463	0.042423299	0.005523162	0.007678972	0.016289722	0.006411381
0.001945791	0.000561657	0.001094126	0.005011431	0.000766648	0.000445642	0.00245564	0.001286012	0.000603698	0.001558815	0.000451207
0.001837174	0.003318885	0.003008025	0.002758053	0.002097009	0.001372399	0.006817884	0.001935708	0.002130326	0.003333006	0.000703806
0.04069403	0.015631582	0.009871777	0.043798291	0.023811194	0.009416121	0.033679691	0.019049332	0.007272819	0.017739782	0.017045597
0.012425753	0.008147679	0.001248552	0.005823959	0.011614972	0.00141009	0.00559143	0.001674046	0.004581465	0.006167021	0.016488337
0.001430637	0.002970584	0.004619643	0.008256496	0.001936664	0.003576219	0.001677156	0.001458471	0.002054469	0.002597314	0.000530265
0.169954133	0.050992292	0.034295759	0.050661882	0.053254497	0.280721008	0.207041322	0.074771566	0.083831805	0.149978889	0.06588586
0.000422054	0	0.000517492	0.000297759	0.000736584	0.001272629	0.001832853	0.000732953	0.001371753	0.00220068	0.000574614
0.004952922	0.000249828	0.001301123	7.07E-05	5.76E-05	0.001946634	0.000368756	0.004919554	0.002098719	0.003695521	5.01E-05
0.007004227	0.002111686	0.002322964	0.006999854	0.002620634	0.001270411	0.002889952	0.001412383	0.004967073	0.005149847	0.000746227
0.025084256	0.000288123	0.01984541	0.017302811	0.000944531	0.02877382	0.000934181	0.005966204	0.020746973	0.043171282	0.001342052
0.107986743	0.072760163	0.080242482	0.144652707	0.026101117	0.083042336	0.06192638	0.141983164	0.101448084	0.090739659	0.079686238
0.037969302	0.135527201	0.274315144	0.088361166	0.000759132	0.000514372	0.003998951	0.002607705	0.000793341	0.008047835	0.002452715
0.000403434	0.001508087	0.002640031	0.000270002	0	0.000427905	0.001507801	0.000395467	0.000761734	0.001157916	0
0.000266887	0.001203552	0.003095095	0.002371976	0.001598437	0.003562916	0.015263756	0.000316671	0.000638466	0.006292835	0.000416499
0.059624372	0.017743268	0.02034483	0.116683573	0.005261312	0.014347002	0.080926861	0.019600905	0.006100192	0.022151804	0.004895789
0.00605771	0.001475262	0.002027255	0.01120885	0.000395851	0.001520946	0.006389035	0.001907461	0.000478849	0.002452308	0.000723088
0.00097755	0.000687483	0.002776386	0.001569542	0.000175377	0.004239139	0.007022748	0.000735926	0.000821787	0.002516281	0.005267938
0.00040964	0.000306359	0.00044685	0.00091094	0.000117753	0.001297017	0.000975154	0.000627395	0.000216509	0.001590801	0
0	0.001134256	0.004769141	0.000840285	0.001109886	0.000235015	0	0.005213924	0.009237207	0.000260158	0.01044332
0.00071687	0.011805747	0.074957492	0.007630698	0.007716591	0.002225991	0.001529653	0.059537155	0.06522148	0.005218085	0.064096458
0	0.0009829	0.006697826	0.000974024	0.00045097	0.000155199	6.83E-05	0.007215048	0.00381341	0.0005587	0.005077043
0	0.00020971	0.000381137	0.000252338	7.77E-05	0	0	0.001294932	0.003661695	0.000234569	0.004623907
0	0.001063137	0.004028224	0.001165801	0.000638874	0	0.000292273	0.006532644	0.022063414	0.000656792	0.020356376
9.31E-05	0.000694777	0.002600603	0.001150661	0.000719046	0	0.000193938	0.005163376	0.012393187	0.000808195	0.009330729
0.000133443	0.001843622	0.005804125	0.001733561	0.001270231	0.000228364	9.83E-05	0.009632453	0.026741281	0.001648377	0.024710331
0.000145857	0.000647365	0.001674046	0.000774677	0.000576239	6.21E-05	0	0.002717723	0.010106406	0.000328396	0.005981385
0	0.000519715	0.001524548	0.000514769	0.000110237	0	0	0.002900589	0.008454929	0.000565097	0.00820464
0	0.000269887	0.000877272	0.000431498	0.000215463	0	0	0.00160863	0.003794446	0.000377442	0.003858398
0.050724008	0.005483454	0.008437585	0.001476177	0.044668537	0.042757214	0.009309034	0.027444835	0.005728807	0.027290989	0.042565787
0.045153522	0.012898061	0.006374188	0.011516702	0.046028962	0.021102575	0.012133429	0.01039068	0.049817705	0.025167077	0.066146172
0.12922907	0.143439641	0.045940152	0.054898636	0.21286516	0.111964703	0.037304423	0.10048586	0.181401685	0.045327181	0.156302725
0.000887554	0.003346238	0.000961057	0.000772154	0.001706168	0.000676222	0.000188475	0.003814921	0.005423797	0.0012752	0.006623487
0.006157016	0.000931841	0.00761124	8.07E-05	0.000202936	0.003948696	7.92E-05	0.001511993	0.00293631	0.005665898	0.001897383
0.003345395	0	0.000213568	5.05E-05	0	0.016694935	0.000207596	0.000300317	0.001036717	5.97E-05	0.000431925
0.004546386	0.018662344	0.004938353	0.000310376	0.02375357	0.004598313	0.000964228	0.111020752	0.025296831	0.000125814	0.006714114
0.002473358	0.077614488	0.014215425	0.025160613	0.013942476	0.004088375	0.000169354	0.00036722	0.007245953	0.012361764	0.000287307
0.001135821	0	0.019334489	0.028128107	0.007922032	0.005230193	0.000516258	0.000978261	0.005422217	0.001560947	8.87E-05
0.00594599	0.001774327	0.008025234	0.000953837	0.012038383	0.001558637	0.002515734	0.000709165	0.007473525	0.001456458	0.004542922
0.025155632	0.001196257	0.012161885	0.000141309	0.001425565	0.012342723	0.00123738	0.00074782	0.002209344	0.005631778	0.000987256
0.002647145	0	4.27E-05	0.002793381	0.001092349	0.002613988	0.001010664	0.004353114	0	0.001880814	0.00219626
0.007159393	0.023270487	0.004529288	0.014814759	0.003402315	0.106135887	0.005916481	0.005038491	0.055012351	0.017255718	0.015429736
0.021391287	0.070345766	0.010011418	0.004224137	0.000666433	0.016149523	9.01E-05	0.009027359	0.000622662	0.01491643	0.002834505

0.016944208	0.022916716	0.008871292	0.002745436	8.52E-05	0.002975379	0.005659718	0.003287136	0.000450403	0.000927612	0
0.00080066	0.018049626	0.004319005	0.002450201	0.007556246	0.000782644	0.011393186	0.009641374	0.007604695	0.001987436	0.01898733
0.000155167	0	9.20E-05	6.31E-05	0.000102721	0.000625229	0	0	0.000346099	0.000110887	0.002579978
0.00710974	0.000443126	0.006375831	0.000565237	0.011366939	0.03841387	0.006446397	0.001824204	0.005006582	0.022100626	0.001295774
0	0.000213357	0.016702672	0.0012844	0.002102019	0.001523163	0.000893208	0	0.001300637	0.002951299	0.001745053
0.005688413	0	0.000813202	0	0.001746254	0.003713681	0.027648486	0.00227022	0.014572505	0.007403838	0.000403001
0.014917731	0.005326627	0.009876705	0.000302805	0.007405923	0.01585908	0.071350138	0.002215212	0.010955059	0.02842758	0.000528336
0.001160647	0.000244357	0.002403463	9.08E-05	0.004672546	0.01025641	0.013277938	0.003471489	0.002436916	0.002079131	0.002263748
0.000971344	3.10E-05	0	0	0.014521221	0.000259403	0.053671715	0.004693572	0.002903122	0.153450504	0.002448858
0	3.46E-05	0	0	0.004419502	0.000383562	0.004130064	0.001210189	0.001261128	0.00228811	0
0.015873558	0.00457532	0.026705876	0.00660873	0.024605402	0.017601738	0.091986801	0.011872939	0.017074215	0.042855681	0.016096905
0.000515154	0.000111237	0.000464921	0.00018673	0.00314426	0.000574235	0.007022748	0.000865271	8.38E-05	0.004273413	0.001440392
0	0.000114884	0.000354852	6.56E-05	0.001255199	0.000795947	0.004455116	0.000520352	0.000169099	0.000893493	0.002539485
0	0	0.002301608	0.000174113	0.000258055	9.09E-05	0.00670316	0.00064821	0.000640046	0.00538868	0.010181081
0.000114823	0.000206063	0.002235894	5.55E-05	0.000135291	0.000175153	0.010155806	0.001430224	0.000733287	0.0043054	0.004918927
0.000282403	0	0.00057992	0	0	0.000175153	0.000314125	0	9.17E-05	3.84E-05	0.000343226
0.00061446	0	0	0	0	0	7.10E-05	0	0	0	0
0.00052136	0	0	0	0	0	0	0	0	0	0
0	0	0	0	0	0	0.000111992	0	0	0	0
0	0	0	0	0	0	9.56E-05	0	0	0	0
0	0	0	0	0	0	0	0	0	0	0
0	0	0	0	0	0	0	0	0	0	0
0.004142952	0	0	0	0	0.000450076	0.001046174	0	0	0	0
0.001238231	0	0	0	0	0.000157416	0.003266903	0	0	0	0
0.001142027	0	0	0	0	0	0.001617062	0	0	4.05E-05	0
0.00014896	0	0	0	0	0	0	0	0	0	0
0.00038171	0	0	0	0	0	0.000551768	0	0	0	0
0	0	0	0	0	0	0	0	0	0	0
0.000397227	0	0	0	0	0.000119725	0.000191207	0	0	0	0
0.000136547	0	0	0	0	7.09E-05	0	0	0	0	0
0.00025137	0	0	0	0	7.98E-05	0.000185744	0	0	0	0
0.001173061	0	0	0	0	9.09E-05	0.000101066	0	0	0	0
0.000155167	0	0	0	0	0	0.000185744	0	0	0	0
0.000425157	0	0	0	0	0	0	0	0	0	0
0	0	0	0	0	0	0	0	0	0	0
0	0.000289947	0.003310306	0.000340656	0.000440948	0.000172936	0.000478017	0.001175995	0.001912236	0.000102357	0.002552983
0	0	0	0	0	0	0	0	0	0	0
0	0	0	0	0	0	0	0	0	0	0
0	0	0	0	0	0	0	0	0	0	0
0.001803038	0	0	0	0	0	0	0	0	0	0
0.004760516	0.000988371	0.010891976	0.000244768	0.002390139	0.002163912	0.000958765	0.018641971	0.005790441	0.003219987	0.006717971
0.002277848	0.000377478	0.019214562	0.001526644	0.000160345	8.43E-05	0	0.011856585	0.003045355	0.000906288	0.008187286
0.001827864	0.000902664	0.010637337	0.010767259	0.060615323	0.01135832	0.011169202	0.107660766	0.03848652	0.014374789	0.040494862
0.036774518	0.139801633	0.089754479	0.160845231	0.29092549	0.08191382	0.055815961	0.113412883	0.087441349	0.086095201	0.102154031
0.003100232	0.017221729	0.019628556	0.017918515	0.027732124	0.006227898	0.010882392	0.023977806	0.009927825	0.010274104	0.023310432
0.000729284	0	0.000946271	0.00034318	8.77E-05	0	0.005752589	0.000896492	0.000842332	0.000513918	0.000460848
0.00013965	0	0.000369637	0	0	0	0.00645186	0	0.000474108	0	0.001363262

H108	H109	H110	H111	H112	H113	H114	H115	H116	H117	H118
0.010799037	0.002680929	0.022963251	0.002478849	0.001564437	0.006801432	0.016792251	0.063744501	0.001439915	0.002726025	0.001757891
0.004165642	0.007973298	0.002176848	0.010428725	0.010717041	0.005504434	0.006204034	0.018265206	0.021194879	0.004277526	0.009790155
0.001490179	0.001522032	0.00152028	0.001781022	0.006940291	0.002754299	0.027804098	0.006921271	0.01101521	0.007193322	0.001100237
0.01215773	0.000820418	0.006593427	0.001304893	0.026283401	0.008793771	0.027904365	0.012830405	0.003088715	0.01071946	0.007959389
0.001669306	0	0	0.000270799	0.023018019	0.000995128	0.004672452	0.016698567	0.031956641	0.02386234	0.00091716
0.006884934	0.010205911	0.00253195	0.002276494	0.012901185	0.007001291	0.007700522	0.015192055	0.022793546	0.022226211	0.003284714
0.000512606	0.001058026	0.000136862	0.000111593	0.000927395	0.00076196	0.000741977	0.001372897	0.003278104	0.001800254	0.000300388
0.003077545	0.002501603	0.001636797	0.002734769	0.002641341	0.001005538	0.013764182	0.004496154	0.032800537	0.026021618	0.000849618
0.022282185	0.019459152	0.023360891	0.010242737	0.039509608	0.010419703	0.06058145	0.034733287	0.034449337	0.087871366	0.009932351
0.00341293	0.001748432	0.001936415	0.005395143	0.008084367	0.003418412	0.013370633	0.004172137	0.011976081	0.006429112	0.001075353
0.003717826	0.001761881	0.000813775	0.002135143	0.001133241	0.003984678	0.004241303	0.004526217	0.005442154	0.00464168	0.002746149
0.061366049	0.144637918	0.165303594	0.067799359	0.087324597	0.06243286	0.190374849	0.22369533	0.254708967	0.499226814	0.15055474
0.000228672	0.000641092	0.001383417	0.00044042	0.000572038	0.000120748	0	0	0.001328509	0.001138622	0.000815846
0.008007333	0.006668699	0.00260223	0.008356073	0.001230748	0.015168422	0.000157921	0	0.002760069	0.000710357	0.000287946
0.00436573	0.002586783	0.001614604	0.002203587	0.002470163	0.001386518	0.002253506	0.000414207	0.011847965	0.007790842	0.00240488
0.033391835	0.026547026	0.012001332	0.001977426	0.02692261	0.002816755	0.004183649	0.0001737	0.043528878	0.038084858	0.008032264
0.146367259	0.168140879	0.036891749	0.316255658	0.256093069	0.188676771	0.031724546	0.038414388	0.104138989	0.056572081	0.16399043
0.00569584	0.002030871	0.005960902	0.001538494	0.122786604	0.022490319	0.081279109	0	0.079874892	0.000733437	0.013025101
0.000710789	0.00039676	0.000318112	0.000291629	0.001397592	0.000703668	9.27E-05	0	0.001478907	0.000330816	0.000136863
0.000423043	0.001432369	0.002302613	0.001617353	0.000697713	0.003591206	0.00314087	0.019531211	0.013661088	0.000948852	0.004768879
0.024142051	0.010719233	0.0077771	0.013072732	0.02237231	0.017298164	0.012350414	0.027337683	0.075140162	0.020718306	0.010606001
0.002761215	0.000905598	0.000821173	0.001459635	0.001722614	0.001911146	0.000869818	0.002064356	0.006742812	0.001548936	0.001011365
0.001535914	0.000629884	0.001327933	0.000678485	0.001003233	0.001421909	0.001892544	0.002678986	0.006887639	0.0008155	0.00133486
0.00057168	0.000237607	0.000741645	0.000743952	0.000327188	0.000988883	0.001150566	0.002271459	0.00174628	0	0.000394592
0.00041161	0.000118804	0.001174425	0.000437444	0.000140843	0.003224799	0.000165441	0	0	0	0.000838953
0.006480947	0.001764123	0.007279587	0.001336139	0.003230713	0.009495357	0.001932651	0	0.000777053	0.000656503	0.003629538
0.000499267	0.000239849	0.000813775	0.000165157	6.28E-05	0.000433027	0.000140374	0	9.47E-05	6.92E-05	0.000271949
0.000179126	8.74E-05	0.000236735	0.000132424	6.07E-05	0.000637049	8.77E-05	0.000133615	0	0	0.001166002
0.000811786	0.000125528	0.001751466	0.000438932	0.000723714	0.004174127	0.000135361	0.000825074	0.000328646	0.000225673	0.00629037
0.00061932	0.000154669	0.001078252	0.000349658	0.000437696	0.001919474	8.77E-05	0.000133615	0.000181034	7.44E-05	0.005021276
0.000640282	6.28E-05	0.002737243	0.000821323	0.001076904	0.006491235	4.01E-05	0.000283932	0.000286869	0.000220544	0.010403373
0.000402082	5.60E-05	0.000826721	0.000214258	0.000383525	0.002142233	0	0	0.000100265	0.000105143	0.003761069
0.000102902	0	0.000704655	0.000266335	0.000212347	0.001909064	0.000150401	0	4.73E-05	0.00014361	0.002822579
0.000127675	0	0.000247831	8.18E-05	0	0.000859808	0.000107787	0	0	0	0.001366854
0.035120215	0.053741869	0.052481089	0.078954181	0.016786275	0.065532748	0.026312623	0.023917132	0.016042379	0.0036877	0.068292908
0.053951358	0.048579511	0.043768148	0.112839726	0.021951949	0.04096057	0.133611074	0.008745119	0.007224641	0.010160407	0.140323779
0.158854658	0.092119951	0.106476909	0.09185134	0.047307956	0.047426823	0.137361067	0.239167978	0.034583023	0.023777712	0.081604178
0.002543976	0.001353914	0.006360391	0.002392551	0.000985898	0.003988841	0.002639534	0.003995096	0.000788193	0.001754094	0.002044059
0.001114776	0.008300569	0.005907267	0.001769119	0.005306517	0.032701836	0.012287747	0.000694799	0.000339786	0.000433394	0.00745815
0.000329669	0.000412451	0.000810076	0	0.002587171	0	3.26E-05	0	0.000983153	0.000977061	0.000248842
0.009169749	0.011600174	0.008735135	0.031695349	0.005514531	0.001790398	0.036467185	0.005027274	6.68E-05	0.000376976	0.00141129
0.006496192	0.001288908	0.012299099	0.012209747	0.015590196	0.003301828	8.27E-05	0.00210444	0.000167108	0.000487248	0.008784122
0.000314424	0.000455041	0.011278182	0.005221058	0.002970696	0.004072116	0	0.003600931	0.000111405	0.000518022	0.001613918
0.002349605	0.00309338	0.003920916	0.005310332	0.001911126	0.009836782	0.004231276	0.000300634	0.000359282	0.001679724	0.010623776
0.018541492	0.001786539	0.014305794	0.002609785	0.020766704	0.022119749	0.00130598	0.000146977	0.00133408	0.006865071	0.000808736
0.004369542	0.00736583	0.002757588	0.006153974	0.006030231	0.018351584	0	0.007495816	0	0	0.005163471
0.040568326	0.056546085	0.036727145	0.006964882	0.011852449	0.031084232	0.004652399	0	0.005319608	0.001238636	0.001299311
0.014206251	0.029573158	0.008759178	0.015146871	0.030653856	0.034123746	0.000155414	0.000450952	0.002556754	0	0.044939087

0.008135008	0.005411173	0.003956056	0.00149832	0.013438554	0.006913853	0.000330882	0.000334038	0.002938318	0.003277385	0.005623829
0.001469218	0.004731974	0.02951044	0.001572715	0.001908959	0.008976975	0.002163265	0.001286047	0.004500778	0.000733437	0.003320263
5.15E-05	0.000100871	0.000626977	0	0.0005092	0.001134613	0.000253175	0	0.000144827	0.000387234	0.000348379
0.005632955	0.004615412	0.007314727	0.001217106	0.014998657	0.003786901	0.000945018	0.000557844	0.016022883	0.027757761	0.001176667
0.001179567	0.000508839	0.000567793	0.000175573	0.000153843	0.000485073	0.000799631	0.000163679	0.001618164	0.002213133	0.000332382
0.001223395	0.00455489	0.003149679	0.001717042	0.001547102	0.003757755	0.000486296	0	0.000375993	0.000184641	0.002945223
0.009583264	0.013353089	0.002833417	0.005706115	0.011993292	0.003609943	0.001403741	0.000467653	0.032427329	0.001620741	0.016859045
0.002446791	0.007433078	0.007002164	0.003246608	0.002201479	0.003955531	0.000260695	0.000313996	0.000997078	0.000582133	0.002408435
0	0	0.005535519	0.005113929	0.007638004	0.000587084	0	0	0.000384349	0.001705369	0
0.000163882	4.26E-05	0.000344005	0.000691876	0.00020368	0	0.000170454	0	0.000133686	0.000197464	0.000101314
0.0096976	0.016477851	0.018955409	0.010956931	0.012350815	0.014666694	0.018925436	0.014243387	0.019704831	0.036600033	0.016921256
0.000436382	0.000571603	0.000482717	0.000168133	0.000255684	0.000401799	0.000431149	0.000187061	0	0.000225673	0.000808736
0.001011874	0.002528502	0.011529712	0.000241041	0.004125606	0.006072782	0.001403741	0.000297294	0.000356497	0.000474426	0.002264462
0.000504984	0.000576086	0.001805101	0.001484929	0.000554703	0.001040929	0.000263201	0.000638013	0.00175185	0.000451346	0.000296833
0.001471123	0.000977329	0.005361667	0.000572843	0.000223181	0.003649498	0.008718234	0.000454292	0.003155558	0.000158997	0.000351934
0	0	0.00061403	0	0.000121341	0.000345589	0.000147894	0.000678097	0.016847283	0.001523292	0
0	0	0	0	0	0	0	0.000484355	0.001503973	0.001738707	0
0	0	0	0	0	0	0	0.000444271	5.57E-05	5.90E-05	0
0	0.000132253	0	0	0	0	0	0	5.29E-05	7.44E-05	7.82E-05
0	0.000208467	0	0	0	0	0	0.000273911	0	0	5.87E-05
5.15E-05	0.000156911	0	0	0	0	0	9.69E-05	0	0	0
0	0.000116562	0	0	0	0	0	0	0	0	0
0	0.004431603	0	0	9.10E-05	0	0.000726937	0.000724863	0.00308036	0.002302889	0
0	8.97E-05	0	0	0	0	0.003514366	0.005905795	0.001930099	0.004421136	0
0	0	0	0	0	0	0.000684324	0.001870614	0.00175742	0.002620882	0
0	6.05E-05	0	0	0	0	0.000616643	0.001777083	0.001902247	0.000828322	0
0	0.000190534	0	0	0	0	0.000152907	0.00105222	0.000604374	0.001105284	0
0	0	0	0	0	0	0	0	0	0	0
0	0.000114321	0	0	0	0	0	0.00044093	0	0	0
0	0.000446074	0	0	0	0	4.51E-05	0.000317336	4.46E-05	0	0
0	0.000748688	0	0	0	0	0	0.000130275	0	0	0
0	4.93E-05	0	0	0	0	0.000403576	0.000567865	0	8.98E-05	0
0	0.000177085	0	0	0	0	0.000200534	0.000330698	0.000582093	0.00141302	0
0	0.000195017	0	0	0	0	8.52E-05	0	8.36E-05	8.21E-05	0
0	0.000179326	0	0	0	0	0.000142881	0	6.41E-05	8.46E-05	0
0.00044591	8.97E-05	0.000329209	0.000177061	0.000177678	0.000181122	0.000112801	0.000170359	4.46E-05	6.67E-05	0
0	0	0	0	0	0	0	0	0	0	0
0	0	0	0	0	0	0	0	0	0	0
0	0	0	0	0	0	0	0	0	0	0
0	0	0	0	0	0	0.000376002	0	3.34E-05	0	0
0.001537819	0.001123031	0.010658603	0.002114313	0.000686879	0.003757755	0.004883013	0.000133615	0.000169893	0.001023221	0.007870517
0.00179317	0.000161394	0.005561412	8.03E-05	0.000292519	0.000691177	0.005206375	0	0	7.95E-05	0.004879081
0.020317511	0.029176399	0.049227839	0.019564461	0.002959862	0.044058375	0.005900725	0.01803472	0.008684051	0.00277475	0.017337177
0.20071117	0.146688963	0.159331595	0.101674488	0.062939321	0.152589832	0.045556408	0.127007987	0.021551376	0.018197438	0.096891963
0.014036652	0.014657688	0.033007823	0.008911062	0.004987996	0.018999042	0.011442996	0.020466518	0.001111269	0.004226237	0.011665357
0	0.004252276	0.007532967	0.000117544	0.00045503	0.002970812	0.007547614	0.003407189	0.000281299	0.000800113	0.000133308
0.000156259	0.000537979	0.000606632	4.02E-05	0	0.000341425	0.000463736	0.000183721	0.000130901	7.95E-05	6.22E-05

H119	H120	H121	H122	H123	H124	H125	H126	H127	H129	H130
0.000925679	0.013524124	0.005519125	0.001157588	0.002923324	0.007109653	0.002457348	0.00769518	0.023021119	0.062377716	0.167001798
0.00304838	0.00380165	0.004584807	0.002374616	0.00531994	0.005354542	0.010279732	0.015408035	0.012332018	0.014306668	0.016909837
0.001124947	0.003794172	0.000662411	0.001087746	0.001362782	0.002738361	0.006536441	0.010050351	0.003033424	0.018222432	0.022561319
0.002408178	0.005035467	0.002736421	0.003052228	0.011650219	0.011628335	0.005430114	0.007005861	0.026216806	0.01191684	0.008804919
0.000484745	0.000103192	0.000578525	0.00039676	0.002065711	0.001898622	0.011021622	0.002447964	0.017488388	0.006760412	0.020516217
0.001726991	0.009188568	0.001272754	0.004419341	0.002850877	0.006087616	0.001882058	0.019760462	0.00869236	0.025714862	0.013049535
0.000234599	0.000574285	0.000494639	0.000453228	0.000789082	0.000482898	0	0.006016071	0.001455863	0.002419431	0.004289224
0.000792833	0.000625134	0.000905391	0.001838172	0.001674107	0.001848199	0.007762512	0.00659934	0.00464479	0.006803472	0.001839219
0.004128103	0.009668634	0.019690084	0.007609767	0.01563479	0.021245566	0.01755546	0.02024652	0.034275888	0.057813362	0.049552548
0.000460719	0.00473636	0.002273602	0.000948063	0.003982613	0.002009165	0.002733279	0.002218191	0.006988594	0.014594631	0.012184828
0.001825919	0.000650558	0.004662908	0.001585554	0.000532581	0.002643332	0.001330196	0.010569549	0.006510819	0.005842697	0.001135787
0.06974105	0.031081706	0.197025227	0.069932283	0.100697055	0.056679408	0.009917898	0.127941479	0.188360757	0.1476095	0.016769151
0.000422562	8.97E-05	0.001249613	0.000720706	0.000281955	0.000455747	0	0.001891207	0.001115561	0.000694342	0.000260785
0.001975723	7.78E-05	0.00071737	0.000805408	0.005915179	0.00220504	0	0.009931046	0.001557278	0.005382493	0.000645099
0.001505111	0.000486049	0.00230542	0.001295785	0.00232417	0.003166957	0.000377453	0.003073211	0.006278692	0.003024961	0.000356863
0.007706451	0.001465624	0.00428976	0.014629594	0.024244204	0.02337885	0.005135961	0.009365451	0.006988594	0.020391576	0.008403448
0.057854206	0.098084668	0.099581437	0.032696288	0.117490993	0.160987284	0.035602909	0.114172785	0.104562982	0.085011101	0.015544148
0.009138073	0.018843742	0.000185128	0.006357075	0.019082863	0.034380776	0.00040869	0.019521852	0.012990086	0.020292	0.122270338
0.000175243	8.82E-05	0.00024298	0.000221413	0.000767544	0.000116361	0.000192631	0.001144445	0.000128459	0.001719707	0.000219608
0.002310663	0.001257745	0.000144631	0.003327137	0.001366698	0.005432116	0.005830995	0.007069933	0.004142224	0.004583193	0.00159216
0.006242324	0.001311584	0.01499825	0.004790839	0.011188127	0.012879215	0.048139548	0.012803206	0.02605905	0.038872368	0.074368283
0.000412669	3.14E-05	0.001214902	0.000485919	0.001118029	0.000826163	0.00362615	0.001550966	0.003152868	0.003867322	0.00455344
0.002193364	0.000206384	0.001272754	0.001154616	0.000248669	0.001644568	0.0037511	0.00140294	0.00429998	0.002949606	0.000545589
0.000706625	0.000293125	0.000954566	0.001147186	0.000387688	0.000308356	0.000570084	0.000397684	0.002684107	0.001286416	0
0.000607697	0.001247276	0.001379781	0.001396833	0.001155232	0.001266395	0	0.000320356	0.000585951	0.000188387	0
0.006727069	0.007609282	0.005741857	0.012217829	0.012852444	0.005649323	0.015462549	0.004884882	0.001827717	0.003434031	0.001708827
0.00079566	0.000323036	0.000428108	0.000695444	0.000843907	0.000352961	0.000356628	0.000437452	0.000412419	8.07E-05	0.000288236
0.000363205	0.000297611	0.000511994	0.000123337	0.000440555	0.000129936	0.033817167	0	9.24E-05	0	0
0.003041314	0.001508995	0.006346415	0.000570621	0.001578164	0.00169693	0.165428448	0.000554548	0.000169024	0.000231447	0
0.002993263	0.000804598	0.004674479	0.000497807	0.001212014	0.001353665	0.120519375	0.000304891	0.000171278	8.61E-05	0.000171569
0.002056278	0.002412298	0.008047277	0.001105578	0.00336192	0.002199222	0.23143583	0.000731296	0.000105922	0.00017224	0.000130392
0.001139079	0.000583259	0.002071118	0.000173861	0.001266839	0.000607016	0.080634329	0.000165702	0.000105922	0	0.000130392
0.00152631	0.000771696	0.002455837	0.000274909	0.000783208	0.000581805	0.091528397	5.08E-05	0.0001848	0.000244903	0
0.000650095	0.000465111	0.001669043	9.21E-05	0.000419016	0.00035684	0.042613119	0.000152445	0.000105922	0	0
0.032663029	0.007479171	0.069952301	0.006832593	0.016118421	0.021941792	0.000799159	0.028102982	0.03595261	0.014266299	0.005215697
0.015908953	0.019500282	0.064248048	0.005276759	0.024577068	0.033405283	0.001160993	0.014780578	0.075722917	0.022646841	0.010561785
0.093695634	0.195614493	0.191644948	0.402394825	0.131565241	0.100227486	0.009207245	0.053729279	0.092172369	0.041377918	0.030587315
0.002413831	0.000538393	0.003251308	0.001304701	0.002606125	0.0020848	0	0.002823554	0.002319014	0.001025365	0.001080884
0.000585085	0.000611674	0.002108722	0.003405894	0.001078869	0.002141041	0	0.002547385	0.001388253	0.021753347	0.002769123
0.000938398	8.67E-05	0.00036447	0.000176833	0	0.001256698	0	0.00010163	0	8.88E-05	0.000133824
0.000587912	0.347758268	0.002053762	0.006749377	0.202615915	0.006890506	0	0.002949488	0.00089921	0.001730472	0.00196618
0.074191374	0.002605222	0.004061243	0.025513002	0.017011278	0.031264242	0	0.009513478	0.001701512	0.004666622	0.000569609
0.052280349	0.000689442	0.001845493	0.02040416	0.020196977	0.002691816	0	0.010284543	0.001784897	0.00235215	0.00211373
0.004594475	0.011594883	0.003517429	0.017181043	0.002069627	0.014322091	0.0001666	0.005145585	0.001647424	0.002435578	0.002199514
0.000907306	0.000279665	0.007703055	0.002881339	0.012008537	0.030098693	0.000380056	0.008521478	0.018565637	0.002010361	0.023278477
0.005893252	0	0.002351702	0.000104019	0.000855655	0.00264915	0.000205647	0.002328659	0.008072604	0.001410213	0.034715264
0.045811905	0.00326924	0.018810727	0.0084761	0.012482378	0.03213307	0.000596115	0.014232658	0.059167544	0.003146067	0.002751966
0.018516399	0.001200915	0.019970669	0.003717953	0.038079574	0.002843085	0.00057529	0.018978351	0.011486896	0.005635471	0.000126961

0.005045302	7.03E-05	0.027696865	0.002310718	0.006054198	0.001648446	0	0.00502628	0.020449695	0.009879567	0
0.002625818	0.04407641	0.003945538	0.008419632	0.016357299	0.005618293	0.000197837	0.008930209	0.001363463	0.014691516	0.003825988
0.000322221	0.000367902	0.000613236	0.000622631	0.000254543	9.50E-05	0.00020044	0.000366753	0.00025241	0.000481733	0.000377452
0.005267182	0.002268727	0.008160089	0.006152008	0.007223136	0.01116677	0.000156187	0.014975001	0.008651794	0.009615825	0.001774023
0.005614841	0.000327522	0.000497531	0.002772862	0.000315241	0.001772565	0.000192631	0.000899207	0.000198322	0.004225257	0.000775492
0.002950866	0.002593258	0.004153807	0.001985286	0.002069627	0.002036316	0	0.013004257	0.001820956	0.003687008	0.005383834
0.008528962	0.004615221	0.012828783	0.005116271	0.013312578	0.005350663	0.000838206	0.054217546	0.008868145	0.012021799	0.012994633
0.005545592	0.001339999	0.011851076	0.001913958	0.003295348	0.001584448	0.000197837	0.012739135	0.011475628	0.004427101	0.003743635
0.01759496	0.000329018	0.012149016	0.004509987	0.006363565	0.009091667	0	0.033436363	0.002091395	0.017853731	0.004169126
0.003281566	0.001984575	0.001790534	0.000349208	0.000935934	0.000674893	0	0.010779438	0	0.000702415	0.000662256
0.026847506	0.013805284	0.032099437	0.01805332	0.013929355	0.020056745	0.001481177	0.049611043	0.013222213	0.036536365	0.026775053
0.001611105	0.000314062	0.000986384	0.000156029	3.92E-05	0.000882404	0	0.00239494	0.000175785	0.001011909	0.000291667
0.000240252	0.000463616	0.000888035	0.001746041	0.000256501	0.001095732	0	0.00109363	9.01E-05	0.003958824	0.003345595
0.001091029	0.002214887	0.000350007	0.000664239	0.00016839	0.003004051	0.000359231	0.010222681	0.000905971	0.007643141	0.012270612
0.001503698	0.002443704	0.000422323	0.001371571	0.00042489	0.001382756	0	0.004641853	0.000353824	0.004701608	0.007730898
0	3.89E-05	0.000182235	3.71E-05	0.000191886	0.000133815	0	0.000265122	0	0.000645899	0.000229902
0	0	0	3.71E-05	0.0001586	0	0.00074189	4.86E-05	8.34E-05	0.000543632	0.000741178
0	0	0	0	4.31E-05	0	0	0	0	0	0.000758335
0	0	0	2.23E-05	0	0	0	0	0	8.61E-05	0
6.64E-05	0	0.000133061	3.42E-05	6.66E-05	0	0	0	8.34E-05	0	0.00042206
3.96E-05	0	0	0	0	0	0	0	0	0	0
0	0	0	0	0	0	0	5.97E-05	9.47E-05	0.000290655	0.000682844
7.91E-05	0.000207879	0.000248766	0.000454714	0.000131187	4.65E-05	0	0.000262913	0.000811317	0.008076431	0.002614711
0	0.000459129	0	0.00043391	0.000107691	0	0.003061272	0.001193051	0.000570176	0.008383234	0.018453958
0	0.000213862	0	0.000115907	5.87E-05	0	0.000377453	0	0.000286215	0.00446747	0.002137749
0	5.83E-05	0	0.000301656	0	0	0.002397476	0.001239448	0.000108176	0.001041512	0.014339734
0	0.000152545	0	2.53E-05	0	6.01E-05	0.000156187	3.53E-05	0.000103668	0.000955393	0.002206377
0	0	0	0	0	0	0	0	0	0	0
0	0	0	0	0	0	0	0	0	0	0.000130392
0	0	7.23E-05	0	0	0	0	0	0.000180293	0.000166857	0.000380883
5.79E-05	0	7.81E-05	0	0	0	0	0	0	0.000188387	0.000675982
0	0	0	0	0	0	0	0	7.89E-05	0.000255668	0.000260785
0	0	4.63E-05	0	0	0	0.000403484	4.20E-05	0	0.001124941	0.000531864
0	4.04E-05	0.00012149	0	6.46E-05	0	0	0	0.000101415	0.000357936	0.000631374
0	0	0	0	0	0	0	0	8.79E-05	0.000637825	0.001259316
0.000537035	0.000177969	0.000138846	0.000457686	0.000542372	0.000397567	0	0.000384428	8.11E-05	9.42E-05	0
0	0	0	0	0	0	0	0	0	0	0
0	0	0	0	0	0	0	0	0	0	0
0	0	0	0	0	0	0	0	0	0	0
0	0	0	0	0	0	0	0	0	0	0
0.003547257	0.000620647	0.003560819	0.001170962	0.004023731	0.003649854	0	0.000706993	0.001005132	0.002570141	0.004604911
0.000132845	0.000311071	0.000506209	0.000952521	0.00093985	0.000622531	0	0.000985372	0.00210717	0.002976519	0.001931867
0.036751561	0.006169082	0.010997752	0.013721653	0.008556548	0.020537704	0.000205647	0.011687482	0.014276925	0.01215367	0.010108843
0.278431406	0.090956948	0.068092344	0.20822467	0.064781093	0.220302267	0.011531834	0.140570146	0.079191297	0.091801117	0.135028206
0.045506643	0.008790755	0.004489351	0.032329248	0.009654997	0.024631669	0.001507208	0.016775625	0.008838848	0.033872031	0.030882413
0.000327874	0.002316584	0.000127275	0.00146073	0.000601112	0.000255994	0.000564878	0.003256588	0.000367346	0.005689296	0.002038239
1.84E-05	0	0	2.23E-05	0	0.000250176	0	0.000223145	0	0.000742784	0.000953923

H131	H132	H133	H135	H136	H137	H138	H139	H140	H141	H142
0.005503994	0.030052156	0.008198404	0.043603297	0.094297545	0.000616916	0.016576167	0.008672967	0.009468538	0.04944691	0.010733851
0.00545779	0.002861418	0.004645113	0.00906033	0.015243533	0.008746879	0.076957197	0.004745258	0.004160201	0.012297323	0.009269897
0.01633583	0.002017899	0.00016457	0.011719739	0.020875814	0.005830287	0.014576582	0.01551416	0.002324976	0.007087128	0.008449648
0.007080689	0.013365402	0.002652581	0.043966444	0.057535243	0.006105438	0.024345574	0.0057402	0.002416469	0.008440937	0.004538529
0.003930186	0.000936233	0.000118561	0.021565053	0.032004438	0.0050338	0.007200652	0.000590024	0.00117684	0.002392168	0.004910628
0.005706134	0.001557964	0.00232875	0.018168346	0.024057521	0.006699183	0.047718184	0.013618756	0.001654034	0.014642174	0.009973355
2.89E-05	0.000138162	0.000254818	0.002263248	0.002887784	0.000666153	0.004786127	0.001010368	0.000814459	0.001813842	0.001597041
0.001550705	0.001905188	0.000430005	0.002087177	0.004438223	0.002230165	0.00686083	0.015508375	0.001010001	0.002237072	0.011328667
0.032198074	0.028076069	0.016614464	0.025101149	0.034605648	0.019402432	0.075368975	0.075594411	0.01543526	0.043127379	0.052778389
0.003009004	0.003337715	0.003480738	0.00259705	0.015445604	0.001859436	0.040395911	0.024451287	0.005349599	0.04219417	0.011086938
0.002284186	0.001781569	0.00126878	0.001775385	0.002733475	0.004709412	0.002843775	0.001908901	0.002002063	0.004963092	0.001510127
0.049440649	0.049402175	0.080090602	0.144668161	0.090932137	0.294008098	0.054024567	0.010088253	0.017817644	0.083087106	0.109285922
0.000976049	0.000594462	0.000215887	0.000165067	0.000804612	0.000993437	0.000722569	0.000123404	0.001399291	0.000780741	0.000616545
0	0	0.001861584	0.00787185	0.00177088	0	0.000833459	0.001232109	0.000737319	0.002915291	0.00205877
0.002261085	0.001517969	0.001240466	0.0083267	0.00203541	0.004301032	0.007064724	0.00225983	0.000608154	0.004332191	0.002159264
0.027023281	0.001603412	0.001017501	0.016044487	0.003629937	0.006203912	0.028688143	3.09E-05	0.003785263	0.003924733	0.000961484
0.041184658	0.05001118	0.038502239	0.083641152	0.115379104	0.003240979	0.062119489	0.053877287	0.01919182	0.068087422	0.116073344
0.055097692	0	6.02E-05	0.030500666	0.01453812	0.033959324	0.079872513	0.000497471	0.000267301	0.004029884	0.000562223
0.000981825	0	0	0.000803325	0.00052906	0.000961577	0.00084419	0	9.33E-05	0.000764968	0
0.006439615	0.000136345	0.000318522	0.007207914	0.006253192	0.002725435	0.009829803	0.003497724	0.000432345	0.001900591	0.0127383
0.043974195	0.012407354	0.011514572	0.05220144	0.039234921	0.00763759	0.047761109	0.031466017	0.005878818	0.004082459	0.0402112
0.003777137	0.001216193	0.000826388	0.003297667	0.004089191	0.000437344	0.005215376	0.002820932	0.000525631	0.000186642	0.004150133
0.00331799	0.000961684	0.001705863	0.002971201	0.004155323	0.001720413	0.001799269	0.00074235	0.000782168	0.002016256	0.002648154
0.001510277	0.000825339	0.000916636	0.00077398	0.002061128	0.000816762	0.00151668	0.000347073	0.000349823	0.001219743	0.0011842
0.001807713	0.00095623	0.000199961	0	0.000209419	8.98E-05	0.000236087	0.000341288	0.00069247	0.00018927	0
0.018622904	0.007197174	0.004954788	0.001060095	0.001296931	0.001477122	0.003691542	0.006648375	0.018348657	0.005399466	0.00588026
0.001094446	0.000716263	0.000902479	0	0	0.00011006	0.000296897	0.000782842	0.001485402	0.000504721	0.000401976
0.000424495	0	0.000387535	0	0	0	0	0	2.69E-05	0	0
0.002541193	0.00028905	0.003312629	0	0	0	8.23E-05	5.78E-05	3.95E-05	0	0.000138519
0.000872091	6.54E-05	0.002877316	0	0	0	7.15E-05	5.40E-05	3.41E-05	0	0
0.003673179	0.00074535	0.003730247	0	8.45E-05	0	0	3.86E-05	5.02E-05	0.000118294	0.000814817
0.00072193	0.000172703	0.002227885	0	0	0.000104267	0	0	0	6.31E-05	0.000225433
0.000929846	6.18E-05	0.001737715	0	0	0	8.58E-05	0	0	0	7.60E-05
0.000395618	0	0.000465396	0	0	5.79E-05	0	0	0	0	0
0.018224399	0.022122357	0.054224841	0.019367831	0.004603554	0.045816848	0.00642085	0.010130673	0.03550074	0.068628946	0.032247726
0.015463739	0.022609562	0.074905771	0.026667449	0.05997847	0.011950207	0.003630731	0.013260114	0.021342782	0.083247461	0.011888175
0.049925786	0.230511365	0.041353011	0.059460709	0.047468413	0.118902527	0.139638286	0.230672377	0.274128358	0.096601544	0.112911856
0.001695091	0.001021675	0.004011608	0.00029712	0.00203541	0.000952888	0.001802846	0.001330446	0.004953133	0.000778112	0.003492847
0.00111466	0.001119843	0.004561944	0.000520877	0.001216103	0.006519611	0.000829881	0.000154255	0.002377001	0.001790183	0
0.000326312	0.000189064	0.000392844	0	0	0.034715263	0.000121621	2.70E-05	0.000486164	0.000152468	0
0.001094446	0.070459227	0.004813222	0.001529618	8.45E-05	0.016955043	0.00810923	0.011100549	0.020449388	0.003627684	0.005961742
0.00206472	0.061118716	0.007538355	0.001349879	0.001069141	0.010603419	0.000178854	0.019114077	0.007333722	0.00133278	6.79E-05
0.001712418	0.004821143	9.73E-05	0.002222899	0.000360055	0.009969125	0.000883538	0.001307308	0.008768893	0.001721836	0.000111358
8.37E-05	0.005579219	0.006154554	0.019987748	0.001506351	0.00718866	0.011342906	0.000433841	0.011811454	0.002216042	0.006206187
0.013598272	0.019939027	0.003868273	0.001250839	0.000385773	0.016850776	0.002611265	0	0.00226757	0.000262876	0.002849142
0.00010107	0.007646202	0.006673037	0.00196246	0.024608624	0.006374795	0.013689467	0	6.10E-05	0.006290614	5.70E-05
0.000851877	0.042946715	0.00707119	0.001316866	0.003104552	0.051059183	0.00081915	0	0.044859847	0.002415827	0
0.018233062	0.00085988	0.011760542	0.020314214	0.002123587	0.010866984	0.000744032	0.000250664	0.006696865	0.000257618	0

0.02270614	0.000318137	0.016750721	0.061096703	8.45E-05	0.013798057	0.000629565	3.86E-05	0.001680944	0.000226073	0
0.021374901	0.006348202	0.049261206	0.007824164	0.012796632	0.003660945	0.008348894	0.024572763	0.008460331	0.060306303	0.014816082
0.001530491	0.00035995	0.000828157	0.000612581	0	0.000321491	0.000457866	0.000501328	0.000749877	0.000310193	0.000624693
0.012951423	0.00254328	0.000525561	0.005076719	0.006932887	0.011147927	0.001026621	0.002917341	0.011709199	0.000452146	0.00568742
0.008842198	0.000401762	0.000598114	0.008521112	0.001006683	0.000793591	0.000955079	5.01E-05	0.001004619	0.001177683	0.00217556
0.002278411	0.001530695	0.000608731	0.000447514	0.000110221	0.000506856	0.001237668	0.000173536	0.005213257	0.000102522	0.000244445
0.018573813	0.004132149	0.014255632	0.005080388	0.000191049	0.021348757	0.005995178	0.006490264	0.074601964	0.003441042	0.004356553
0.007461868	0.003304992	0.00347543	0.006492625	0.00030127	0.00713363	0.00250753	0.000160039	0.018318159	0.000864861	0.000812101
5.49E-05	0.004548454	0.000116791	0.001445251	0	0.004555908	0	0.011812048	0.032626811	0	0.001309139
0.002211993	0	0	0	0	0.0005503	7.87E-05	0.000320078	0.002330358	0	0.000200988
0.054690523	0.016083203	0.017362991	0.014602904	0.010834702	0.027170356	0.018579329	0.022430552	0.046872673	0.020538474	0.034833411
0.001778835	0.000390854	0.001189149	0	9.92E-05	0.000808073	0.000457866	0.00139022	0.000827017	9.20E-05	0
0.011045528	0.001187107	0.003137442	0.037517836	0.00051069	0.000477892	0.002078281	3.28E-05	0.004036417	0.026521524	0.001290126
0.072265185	0.000449028	6.90E-05	0.000242098	0	0.000755939	0.001169704	0	0.006858322	0.002137179	0.000195556
0.012330563	0.000765347	0.001904054	0.001639663	0.001116904	0.000666153	0.000450711	0.00033936	0.016520608	0.00308616	0.002634574
0	0.000127255	0.000150413	0.000810661	0.000323314	0.000318595	0.000346976	0	0.000611741	0.000178755	0.000640989
0	0	0	0.000506205	0.000845026	0	0.000575909	0.000175465	0	0.000325966	0.002047906
0	0	0	0	0.000235138	0.000263565	0.000114466	0	0	5.52E-05	0.000469878
0	0	0	0	0	0	0.000107312	4.43E-05	0	4.73E-05	0
0	0	0	0	0.000117569	6.66E-05	0	0.000194746	0	0.000155097	0.000249877
0	0	0	0	0	0	0.000103735	0.000241023	0	0	0.000173828
0	0	0	0	0.000429861	0.000101371	0	0	0	3.68E-05	0.000135803
0	0	0	0.006929135	0.008303298	0.000923925	0.003727312	0.001629315	0	0.004098231	0.004410874
0	0	0	0.010087412	0.016889496	0.001401818	0.009447056	8.87E-05	0	0.003982566	0.004717788
0	0	0	0.004621869	0.007888133	0.000894962	0.007214961	0.000121476	0	0.002087233	0.002088647
0	0	0	0.002670413	0.007101892	0.000167986	0.004825474	0	0	0.001083048	0.001387904
0	0	0	0.00180473	0.001278561	0	0.001616838	0.000397206	0	0.000644045	0.001795313
0	0	0	0	0	0	0	0	0	0	6.79E-05
0	0	0	0	0.000815634	0	0.000250395	9.64E-05	0	0.000302307	0.000304198
0	0	0	0.000286116	0.000698065	0.000333077	0.000418518	0.000322007	6.82E-05	0.000239217	0.000575804
0	0	0	0.000135722	0.000433535	0.00011006	0.000132352	0.000501328	0	0.000252361	0.00070889
0	0	0	0.000117381	0.000676021	8.40E-05	0.000690376	2.89E-05	0	7.10E-05	0.00048889
0	0	0	0.000902365	0.000980965	7.24E-05	0.000511522	0.000669079	0	0.000772854	0.00165951
0	0	0	0.000322797	0.000580496	8.11E-05	0.000261126	0.000412631	0	0.000462661	0.000342223
0	0	0	0	0.000286574	8.40E-05	0.000572332	0.000734638	0	0.000733423	0.000673582
0.000617972	0.000532653	0.000104404	0	0.000345358	0	0.000228933	0.000622803	0.001975154	0.000449517	0.000363951
0	0	0	0	0	0	0	0	0	0	0
0	0	0	0	0	0	0	0	0	0	0
0	0	0	0	0	0	0	0	0	0	0
0	0	0	0	0.000135939	0	0	0.000119547	0	2.63E-05	0.000499754
0.002622049	0.001919731	0.050537064	0.000751971	0.004757863	0.008622338	0.00122336	0.01524807	0.022207472	0.002392168	0.026894381
0.005249874	0.005026569	0.020135903	0	0.000106547	0.007837435	0.001287747	0.007213332	0.003159169	0.004392652	0.030009696
0.011877191	0.070761003	0.034076551	0.003877234	0.005885789	0.011950207	0.007483241	0.03038045	0.022611114	0.008338416	0.026345738
0.16651458	0.153836645	0.311134115	0.059295642	0.135887516	0.092172412	0.092385122	0.240392347	0.117804189	0.128007297	0.135218819
0.018920339	0.019099144	0.038951709	0.040452356	0.022474015	0.010687412	0.008921226	0.057801139	0.037698345	0.03301981	0.017021519
0.065146956	0.000398126	0.008177169	0.016668073	0.008865424	0.000573471	0.000372016	0.001293811	0.00177423	0.046720889	0.066918173
0.004311365	8.18E-05	0	0.001470928	0	8.69E-05	0	0	0.000884424	0.000299678	0.002449882

H143	H146	H147	H149	H150
0.007408308	0.003896391	0.003670487	0.01149222	0.065960524
0.003473911	0.004709838	0.006474561	0.00181121	0.019270457
0.003271297	0	0.001647013	0.000209893	0.007913969
0.002070348	0.015686518	0.00210098	0.001264073	0.016295369
0.00473196	0.007070582	0.000445662	0	0.003959925
0.008522685	0.003232432	0.006961745	0.002789923	0.019264577
0.00069073	0.000275679	0.003501633	0.000488178	0.00186384
0.002097977	0.000339745	0.00179649	0.000981072	0.008660681
0.013613825	0.013275298	0.031096717	0.024045695	0.059892756
0.001643016	0.005979514	0.003244201	0.003296968	0.014131668
0.001668803	0.001687077	0.004218568	0.000931547	0.002963329
0.111071202	0.315502862	0.319622986	0.083806182	0.11143057
0.000488116	5.05E-05	0.003025522	0.000650903	0.00016169
0.00246084	0.000712494	0.006042739	0.000837213	0.001978492
0.003772306	0.004090531	0.003377069	0.000132067	0.002928051
0.00848953	0.00171814	0.032818469	0.001068331	0.00573263
0.064271031	0.013504384	0.072313569	0.014102909	0.153843214
0.008419536	0.0009144	0.001475392	0.001087197	0.022463091
0.000300237	4.85E-05	0.000102419	0.00037026	0.000179328
0.005448477	0.003024702	0.004240713	0.001360766	0.003727679
0.010093866	0.035139354	0.029613021	0.002447963	0.021901587
0.000729411	0.003706134	0.002388861	0.000495253	0.00200789
0.002035351	0.005039876	0.003759065	0.00032781	0.008110937
0.000758882	0.002917925	0.002123125	0.000325452	0.002116663
0	0	0	0.00022876	0.000188148
0.001468031	0.000421284	0.000503792	0.003438468	0.000790809
7.92E-05	0	0	0.000297152	0.000111713
0	0.000644545	0	0	0
0	0.003581884	0.000124564	6.60E-05	0
8.10E-05	0.002158838	0	5.42E-05	0
0.000180511	0.004057528	0	5.90E-05	0.000105833
0	0.001230848	0	0	0
0	0.001502644	0.000105187	0	0
6.63E-05	0.000722201	0	0	0
0.018211323	0.077698741	0.042415435	0.065241282	0.052460915
0.01845446	0.248542008	0.016132425	0.002436171	0.050029692
0.04959072	0.009936089	0.108680729	0.212567154	0.07067304
0.001086748	0.002096713	0.001184742	0.007247197	0.00046743
0.000613368	0.001116305	0.002408238	0.007284931	0.000105833
0.000775459	4.27E-05	0	0.000141501	0
0.003820197	5.82E-05	0.009151304	0.045504285	0
0.007067548	0	0.000589603	0.001622542	0
0.003164464	3.69E-05	0.000656037	0.002872465	0.000561504
0.001388827	8.74E-05	0.006884238	0.010673874	0.000676156
0.002169813	0.00139975	0.001259481	0.006539693	0.000273402
0.000296553	0.02383846	0.001768809	0.002294671	0.027449009
0.015667595	0.002609243	0.010981011	0.001955069	0.001275878
0.028036264	0.037459328	0.007163816	0.003563461	0.015381088

0.001794056	0.020924417	0.004409567	0.000224043	0.007058485
0.006601535	0.000343628	0.004094004	0.002518713	0.000420393
8.47E-05	5.82E-05	0.000368156	0.000240551	6.76E-05
0.00424016	0.000731908	0.014385761	0.009152741	0.001014235
0.00063363	5.05E-05	0.003783978	0.001176815	0.000496828
0.000744146	4.47E-05	0.007354814	0.002570597	0.00046743
0.018857846	0.048360293	0.055976305	0.018425757	0.001464026
0.008681093	8.74E-05	0.006225433	0.003386585	0.000764351
0.211998806	0	0.009859935	0	0.000593842
0.000486274	0	0	0.001372557	0.000191088
0.145246673	0.025902169	0.037153297	0.042193167	0.012117898
0.019333068	0	0.000107955	0.00561758	0.000108773
0.000674152	0.000163078	0.000584067	0.001712159	8.23E-05
0.0005489	0	0.001129381	0.000622603	0.000346898
0.002497679	0.000108718	0.002469136	0.000999939	0.000352777
0.000429173	0	0.000218679	0.000474028	0
0.000342602	0	0	7.78E-05	0.000537985
0.000163933	0	0	6.84E-05	7.64E-05
8.29E-05	0	0.000226983	0.000372619	0
0.000222875	0	0.000124564	0.000943338	0.000102893
0.000158407	0	0	0.000334885	0.000102893
0.00026524	0	0	5.66E-05	0.00046449
0.005730295	0	0.002399934	0.000436294	0.005256381
0.004057808	0	0.00024636	0	0.004738974
0.002197442	0	0.000226983	0	0.003518953
6.82E-05	0	0	7.55E-05	0.001828562
0.000537848	0	0	0	0.000655578
0	0	0	0	0
0	0	0.000476111	0.000568361	0.000393935
0.000528639	5.05E-05	0.000470575	0.00183951	0.000911341
0.000607842	0	0.00060898	0.002289954	0.001596317
0.00015104	0	0.000226983	0.000275926	0.000996596
0.000143672	0	0.000132868	7.31E-05	0.00062324
0.000237611	0	0.000193766	0.000424502	0.00015875
0.000313131	0	0	0.000469311	0.000435092
0.000152882	0	0	0.000283002	0
0	0	0	0	0
0	0	0	0	0
0	0	0	0	0
8.66E-05	0	0	7.08E-05	0
0.000302079	0.001452168	0.00130377	0.003881837	0.000296921
0	0.000770736	0.000891325	0.00132539	0.000246944
0.008902126	0.000813447	0.006189448	0.01519954	0.007008508
0.117090683	0.03230685	0.071912196	0.303983718	0.149768637
0.013868013	0.004737018	0.009682777	0.046843826	0.017168492
0.00103149	0.00132986	0.000495488	0.000858438	0.000179328
0.000154723	0	0	0.000148576	7.64E-05

Supplementary table S20. The cladogram of oropharyngeal microbial structure and their predominant bacteria among the CC (n=73), CCR (n=21), and H (n=140) groups.				
Biomaker_names	Logarithm value	Groups	LDA_value	P_value
d__Bacteria.p__Actinobacteriota.c__Actinobacteria.o__Micrococcales.f__Dermabacteraceae.g__Brachybacterium	0.15490196			-
d__Bacteria.p__Bacteria_unclassified	2.987059901			-
d__Bacteria.p__Firmicutes.c__Clostridia.o__Clostridia_unclassified.f__Clostridia_unclassified	0.02413368			-
d__Bacteria.p__Synergistota.c__Synergistia.o__Synergistales.f__Synergistaceae.g__Fretibacterium	2.700777865	H	3.2354756	2.62E-14
d__Bacteria.p__Proteobacteria.c__Gammaproteobacteria.o__Oceanospirillales.f__Halomonadaceae.g__Halomonas	3.951764536	CC	3.6185261	1.56E-10
d__Bacteria.p__Actinobacteriota.c__Actinobacteria.o__Actinomycetales.f__Actinomycetaceae.g__Actinomyces	4.754205971	CC	4.3535641	0.000395
d__Bacteria.p__Patescibacteria.c__Saccharimonadia.o__Saccharimonadales.f__Saccharimonadales.g__Saccharimonadales	3.379029544	H	3.088748	6.45E-20
d__Bacteria.p__Actinobacteriota.c__Actinobacteria.o__Micrococcales.f__Micrococcaceae.g__Kocuria	1.468241804			-
d__Bacteria.p__Proteobacteria.c__Gammaproteobacteria.o__Xanthomonadales.f__Xanthomonadaceae.g__Stenotrophomonas	1.64528188			-
d__Bacteria.p__Bacteroidota.c__Bacteroidia.o__Cytophagales	0.99906837			-
d__Bacteria.p__Actinobacteriota.c__Coriobacteriia.o__Coriobacteriales.f__Atopobiaceae.g__Olsenella	2.197024436			-
d__Bacteria.p__Firmicutes.c__Negativicutes.o__Veillonellales_Selenomonadales.f__Veillonellaceae.g__Anaeroglobus	2.229955955	H	3.1168809	1.37E-08
d__Bacteria.p__Firmicutes.c__Clostridia.o__Peptostreptococcales_Tissierellales.f__Anaerovoracaceae.g__Anaerovoracaceae_Family_XIII_UCG_001	2.155053936	H	3.2687248	1.51E-06
d__Bacteria.p__Proteobacteria.c__Gammaproteobacteria.o__Burkholderiales.f__Comamonadaceae.g__Aquabacterium	1.405443938			-
d__Bacteria.p__Proteobacteria.c__Alphaproteobacteria.o__Caulobacterales.f__Caulobacteraceae	2.039640698	H	3.5912138	1.39E-11
d__Bacteria.p__Planctomycetota.c__Planctomycetes	0.516629796			-
d__Bacteria.p__Actinobacteriota.c__Actinobacteria.o__Propionibacteriales.f__Propionibacteriaceae.g__Cutibacterium	1.953207242			-
d__Bacteria.p__Spirochaetota.c__Spirochaetia.o__Spirochaetales.f__Spirochaetaceae.g__Sphaerochaeta	1.442887648			-
d__Bacteria.p__Firmicutes.c__Clostridia.o__Lachnospirales.f__Lachnospiraceae.g__Lachnospiraceae_UCG_001	1.851782332			-
d__Bacteria.p__Bacteroidota.c__Bacteroidia.o__Bacteroidales.f__Bacteroidaceae.g__Bacteroides	3.802908536	H	3.4875803	3.81E-20
d__Bacteria.p__Firmicutes.c__Clostridia.o__Oscillospirales.f__Oscillospiraceae	3.21248249	H	3.0661856	4.70E-15
d__Bacteria.p__Firmicutes.c__Clostridia.o__Oscillospirales.f__Oscillospiraceae.g__Colidextribacter	1.792791777			-
d__Bacteria.p__Bacteroidota.c__Bacteroidia.o__Bacteroidales.f__Barnesiellaceae.g__Barnesiella	1.765668555			-
d__Bacteria.p__Proteobacteria.c__Gammaproteobacteria.o__Cardiobacteriales.f__Cardiobacteriaceae.g__Cardiobacterium	3.215095978	CCR	3.2872268	1.87E-07
d__Bacteria.p__Verrucomicrobiota.c__Verrucomicrobiae	1.819308864			-
d__Bacteria.p__Actinobacteriota.c__Actinobacteria.o__Pseudonocardiales.f__Pseudonocardiaceae.g__Saccharopolyspora	0			-
d__Bacteria.p__Proteobacteria.c__Gammaproteobacteria.o__Burkholderiales.f__Alcaligenaceae	2.538896749	H	3.0300357	2.51E-27
d__Bacteria.p__Synergistota.c__Synergistia.o__Synergistales.f__Synergistaceae.g__Synergistaceae_uncultured	0.71531635			-
d__Bacteria.p__Firmicutes.c__Clostridia.o__Peptostreptococcales_Tissierellales.f__Anaerovoracaceae.g__Mogibacterium	2.076458388	H	3.4161974	6.09E-13
d__Bacteria.p__Firmicutes.c__Clostridia.o__Clostridiales	1.746356144			-
d__Bacteria.p__Patescibacteria.c__Gracilibacteria.o__Gracilibacteria.f__Gracilibacteria	2.73900693			-
d__Bacteria.p__Cyanobacteria.c__Cyanobacteriia.o__Cyanobacteriales	0.496336485			-
d__Bacteria.p__Actinobacteriota.c__Coriobacteriia.o__Coriobacteriales.f__Atopobiaceae.g__Atopobium	3.413153617	H	3.0576504	8.89E-09
d__Bacteria.p__Firmicutes.c__Bacilli	5.520108498	CCR	5.0875214	1.39E-06
d__Bacteria.p__Actinobacteriota.c__Coriobacteriia.o__Coriobacteriales	3.450941579	H	3.0843799	9.54E-10
d__Bacteria.p__Campilobacterota.c__Campylobacteria.o__Campylobacterales.f__Campylobacteraceae.g__Campylobacter	3.990877424	H	3.5327816	1.88E-15
d__Bacteria.p__Bacteroidota.c__Bacteroidia.o__Bacteroidales.f__Prevotellaceae.g__Prevotellaceae_unclassified	2.375820324	H	3.0750941	5.96E-15
d__Bacteria.p__Proteobacteria.c__Alphaproteobacteria.o__Sphingomonadales.f__Sphingomonadaceae.g__Sphingomonadaceae_unclassified	1.106724995			-
d__Bacteria.p__Actinobacteriota.c__Coriobacteriia.o__Coriobacteriales.f__Eggerthellaceae.g__Enterorhabdus	1.1775365			-
d__Bacteria.p__Firmicutes.c__Bacilli.o__Erysipelotrichales.f__Erysipelatoclostridiaceae.g__Erysipelotrichaceae_UCG_003	2.001424628			-
d__Bacteria.p__Firmicutes.c__Bacilli.o__RF39.f__RF39	2.411643753			-
d__Bacteria.p__Firmicutes.c__Negativicutes.o__Veillonellales_Selenomonadales.f__Veillonellaceae.g__Veillonellaceae_unclassified	2.077591877			-
d__Bacteria.p__Firmicutes.c__Clostridia.o__Lachnospirales.f__Lachnospiraceae.g__Coprococcus	2.592525703			-
d__Bacteria.p__Proteobacteria.c__Alphaproteobacteria.o__Rhizobiales.f__Beijerinckiaceae	1.365086665			-
d__Bacteria.p__Firmicutes.c__Clostridia.o__Lachnospirales	4.553332705	H	4.0956089	4.65E-13
d__Bacteria.p__Actinobacteriota.c__Actinobacteria.o__Corynebacteriales.f__Dietziaceae.g__Dietzia	1.743789648			-
d__Bacteria.p__Proteobacteria.c__Gammaproteobacteria.o__Burkholderiales.f__Oxalobacteraceae.g__Massilia	1.647801971			-
d__Bacteria.p__Firmicutes.c__Clostridia.o__Oscillospirales.f__Oscillospiraceae.g__NK4A214_group	2.068185862			-
d__Bacteria.p__Proteobacteria.c__Gammaproteobacteria.o__Xanthomonadales	2.687186776			-
d__Bacteria.p__Firmicutes.c__Negativicutes.o__Acidaminococcales.f__Acidaminococcaceae.g__Phascolarctobacterium	2.6064044			-
d__Bacteria.p__Actinobacteriota.c__Actinobacteria.o__Corynebacteriales.f__Dietziaceae	1.743789648			-
d__Bacteria.p__Proteobacteria.c__Gammaproteobacteria.o__Alteromonadales.f__Alteromonadaceae	0.280383226			-

d__Bacteria.p__Cyanobacteria.c__Cyanobacteriia.o__Cyanobacteriales.f__Chroococcidiopsaceae.g__Aliterella	0.496336485			-
d__Bacteria.p__Proteobacteria.c__Gammaproteobacteria.o__Burkholderiales.f__Neisseriaceae.g__Alysiella	2.276428976	H	3.1427728	1.23E-14
d__Bacteria.p__Fusobacteriota.c__Fusobacteriia.o__Fusobacteriales.f__Leptotrichiaceae.g__Leptotrichiaceae_uncultured	2.911655943			-
d__Bacteria.p__Firmicutes.c__Clostridia.o__Clostridia_vadinBB60_group.f__Clostridia_vadinBB60_group	2.703408075			-
d__Bacteria.p__Firmicutes.c__Clostridia.o__Lachnospirales.f__Lachnospiraceae.g__Lachnospiraceae_UCG_010	1.543890745			-
d__Bacteria.p__Firmicutes.c__Clostridia.o__Christensenellales.f__Christensenellaceae.g__Christensenellaceae_R_7_group	2.743408963			-
d__Bacteria.p__Firmicutes.c__Negativicutes.o__Veillonellales_Selenomonadales.f__Selenomonadaceae.g__Selenomonas	3.615846385	H	3.0777342	0.014263
d__Bacteria.p__Bacteroidota.c__Bacteroidia.o__Bacteroidales.f__Bacteroidales_Bacteroidales_Incertae_Sedis.g__Phocaeicola	1.567558086			-
d__Bacteria.p__Actinobacteriota.c__Actinobacteria.o__Actinomycetales	4.765899728	CC	4.3644224	0.000289
d__Bacteria.p__Bacteroidota.c__Bacteroidia.o__Bacteroidales	5.489378591	H	5.001183	2.04E-15
d__Bacteria.p__Firmicutes.c__Bacilli.o__Lactobacillales.f__Leuconostocaceae	1.23768733			-
d__Bacteria.p__Firmicutes.c__Bacilli.o__Erysipelotrichales.f__Erysipelatoclostridiaceae.g__Erysipelatoclostridium	0.817187476			-
d__Bacteria.p__Firmicutes.c__Clostridia.o__Peptostreptococcales_Tissierellales.f__Anaerovoracaceae	3.566716886	H	3.1229795	2.70E-12
d__Bacteria.p__Proteobacteria	5.493801155	CCR	4.8436723	6.72E-06
d__Bacteria.p__Proteobacteria.c__Gammaproteobacteria.o__Alteromonadales.f__Alteromonadaceae.g__Rheinheimera	0.280383226			-
d__Bacteria.p__Spirochaetota.c__Spirochaetia.o__Spirochaetales.f__Spirochaetaceae	3.454496414	H	3.0121296	2.39E-14
d__Bacteria.p__Firmicutes.c__Negativicutes.o__Veillonellales_Selenomonadales.f__Selenomonadaceae	3.771450988	H	3.1898672	0.001556
d__Bacteria.p__Firmicutes.c__Bacilli.o__Lactobacillales.f__Leuconostocaceae.g__Leuconostoc	1.23768733			-
d__Bacteria.p__Firmicutes.c__Bacilli.o__Bacilli_unclassified	2.144017919			-
d__Bacteria.p__Bacteroidota.c__Bacteroidia.o__SM1A07	1.053627142			-
d__Bacteria.p__Actinobacteriota.c__Actinobacteria.o__Actinomycetales.f__Actinomycetaceae	4.765899728	CC	4.3644224	0.000289
d__Bacteria.p__Firmicutes.c__Clostridia.o__Peptostreptococcales_Tissierellales.f__Peptostreptococcales_Tissierellales.g__W5053	1.638988159			-
d__Bacteria.p__Campilobacterota.c__Campylobacteria.o__Campylobacterales	4.013369376	H	3.5601255	5.89E-16
d__Bacteria.p__Bacteroidota.c__Bacteroidia.o__Flavobacteriales	4.295845807	H	3.6258407	1.26E-05
d__Bacteria.p__Firmicutes.c__Clostridia.o__Lachnospirales.f__Lachnospiraceae.g__Lachnoanaerobaculum	3.948906995	H	3.3530314	2.26E-06
d__Bacteria.p__Bacteroidota.c__Bacteroidia.o__SM1A07.f__SM1A07	1.053627142			-
d__Bacteria.p__Actinobacteriota.c__Actinobacteria.o__Actinomycetales.f__Actinomycetaceae.g__F0332	3.153735534			-
d__Bacteria.p__Actinobacteriota.c__Actinobacteria.o__Bifidobacteriales.f__Bifidobacteriaceae.g__Parascardovia	1.487118996			-
d__Bacteria.p__Firmicutes.c__Bacilli.o__Erysipelotrichales.f__Erysipelotrichaceae.g__Erysipelothrix	0.716460736			-
d__Bacteria.p__Proteobacteria.c__Gammaproteobacteria.o__Burkholderiales.f__Comamonadaceae.g__Pelomonas	2.028135345			-
d__Bacteria.p__Proteobacteria.c__Alphaproteobacteria.o__Caulobacterales.f__Hyphomonadaceae.g__Hyphomonadaceae_uncultured	1.758061168			-
d__Bacteria.p__Proteobacteria.c__Gammaproteobacteria.o__Alteromonadales.f__Idiomarinaceae	1.75221313			-
d__Bacteria.p__Firmicutes.c__Negativicutes.o__Veillonellales_Selenomonadales	5.108233983			-
d__Bacteria.p__Firmicutes.c__Clostridia.o__Oscillospirales.f__Oscillospirales_unclassified.g__Oscillospirales_unclassified	0			-
d__Bacteria.p__Proteobacteria.c__Gammaproteobacteria.o__Pseudomonadales.f__Moraxellaceae.g__Acinetobacter	3.070064267			-
d__Bacteria.p__Bacteroidota.c__Bacteroidia.o__Cytophagales.f__Hymenobacteraceae	0.758046333			-
d__Bacteria.p__Patescibacteria	4.382393637	H	4.0340199	1.26E-22
d__Bacteria.p__Bacteroidota.c__Bacteroidia.o__Bacteroidales.f__Bacteroidales_unclassified	1.514547753			-
d__Bacteria.p__Bacteroidota.c__Bacteroidia.o__Bacteroidales.f__Rikenellaceae.g__Alistipes	2.833998103			-
d__Bacteria.p__Desulfobacterota.c__Desulfovibrionia.o__Desulfovibrionales.f__Desulfovibrionaceae.g__Desulfovibrionaceae_uncultured	2.305765809			-
d__Bacteria.p__Patescibacteria.c__Gracilibacteria.o__JGI_0000069_P22	1.761175813			-
d__Bacteria.p__Patescibacteria.c__Saccharimonadia.o__Saccharimonadales.f__Saccharimonadaceae.g__Saccharimonadaceae_unclassified	2.968946349	H	3.0009015	1.43E-16
d__Bacteria.p__Firmicutes.c__Clostridia.o__Peptococcales.f__Peptococcaceae.g__Peptococcaceae_uncultured	1.509586514			-
d__Bacteria.p__Firmicutes.c__Bacilli.o__Lactobacillales.f__Streptococcaceae	5.40010854	CCR	4.9666183	2.02E-05
d__Bacteria.p__Desulfobacterota.c__Desulfobulbia.o__Desulfobulbales.f__Desulfobulbaceae	1.898881825			-
d__Bacteria.p__Firmicutes.c__Bacilli.o__Acholeplasmatales	2.680995585			-
d__Bacteria.p__Firmicutes.c__Clostridia.o__Peptostreptococcales_Tissierellales.f__Anaerovoracaceae.g___Eubacterium__brachy_group	2.353091517	H	3.1131384	1.27E-17
d__Bacteria.p__Actinobacteriota.c__Actinobacteria.o__Propionibacteriales	2.3979276			-
d__Bacteria.p__Proteobacteria.c__Gammaproteobacteria.o__Aeromonadales	0.656505134			-
d__Bacteria.p__Proteobacteria.c__Gammaproteobacteria.o__Burkholderiales.f__Comamonadaceae.g__Comamonas	2.406613165	H	3.0429164	7.07E-09
d__Bacteria.p__Proteobacteria.c__Gammaproteobacteria.o__Cardiobacteriales.f__Cardiobacteriaceae.g__Suttonella	1.403120521			-
d__Bacteria.p__Spirochaetota.c__Spirochaetia.o__Spirochaetales.f__Spirochaetaceae.g__Sediminispirochaeta	0.535113202			-
d__Bacteria.p__Bacteroidota.c__Bacteroidia.o__Bacteroidales.f__Tannerellaceae.g__Tannerella	3.399532806			-
d__Bacteria.p__Patescibacteria.c__Saccharimonadia.o__Saccharimonadales.f__Saccharimonadales	3.379029544	H	3.0917032	6.45E-20
d__Bacteria.p__Firmicutes.c__Bacilli.o__Brevibacillales.f__Brevibacillaceae	0.599727159			-

d__Bacteria.p__Proteobacteria.c__Gammaproteobacteria.o__Burkholderiales.f__Rhodocyclaceae	0.840827657			-
d__Bacteria.p__Spirochaetota.c__Spirochaetia.o__Spirochaetales	3.454496414	H	3.0121296	2.39E-14
d__Bacteria.p__Firmicutes.c__Bacilli.o__Lactobacillales.f__Lactobacillales_unclassified	3.353430755			-
d__Bacteria.p__Firmicutes.c__Clostridia.o__Oscillospirales.f__Ruminococcaceae.g__Faecalibacterium	3.572563771	H	3.2670695	3.07E-15
d__Bacteria.p__Firmicutes.c__Bacilli.o__Staphylococcales	4.746670518	CCR	4.4083218	0.003483
d__Bacteria.p__Cyanobacteria.c__Vampirivibrionia.o__Gastranaerophilales.f__Gastranaerophilales.g__Gastranaerophilales	1.669715255			-
d__Bacteria.p__Firmicutes.c__Bacilli.o__Staphylococcales.f__Gemellaceae	4.746006654	CCR	4.4083425	0.003238
d__Bacteria.p__Bacteria_unclassified.c__Bacteria_unclassified.o__Bacteria_unclassified.f__Bacteria_unclassified.g__Bacteria_unclassified	2.987059901			-
d__Bacteria.p__Elusimicrobiota.c__Endomicrobia.o__Endomicrobiales.f__Endomicrobiaceae.g__Endomicrobium	0.525044807			-
d__Bacteria.p__Bacteroidota.c__Bacteroidia.o__Sphingobacteriales.f__env_OPS.g__env_OPS	1.112568276			-
d__Bacteria.p__Bacteroidota.c__Bacteroidia.o__Bacteroidales.f__Marinifilaceae.g__Butyricimonas	0.482260894			-
d__Bacteria.p__Proteobacteria.c__Gammaproteobacteria.o__Burkholderiales.f__Neisseriaceae	5.220189931	H	4.3588646	6.95E-05
d__Bacteria.p__Deferribacterota.c__Deferribacteres	2.440335489			-
d__Bacteria.p__Spirochaetota.c__Spirochaetia	3.454496414	H	3.0121296	2.39E-14
d__Bacteria.p__Firmicutes.c__Clostridia.o__Peptostreptococcales_Tissierellales	4.047860117	H	3.5266817	6.30E-12
d__Bacteria.p__Proteobacteria.c__Gammaproteobacteria.o__Aeromonadales.f__Aeromonadaceae	0.656505134			-
d__Bacteria.p__Deinococcota.c__Deinococci.o__Deinococcales.f__Deinococcaceae	1.146128036			-
d__Bacteria.p__Firmicutes.c__Clostridia.o__Peptostreptococcales_Tissierellales.f__Peptostreptococcales_Tissierellales.g__Murdochiella	0.931603144			-
d__Bacteria.p__Proteobacteria.c__Gammaproteobacteria.o__Xanthomonadales.f__Xanthomonadaceae.g__Xanthomonadaceae_unclassified	2.681414722			-
d__Bacteria.p__Firmicutes.c__Bacilli.o__Lactobacillales.f__Aerococcaceae.g__Aerococcus	0.674729954			-
d__Bacteria.p__Patescibacteria.c__Saccharimonadia.o__Saccharimonadales.f__Saccharimonadaceae.g__Saccharimonadaceae	2.94044533			-
d__Bacteria.p__Proteobacteria.c__Alphaproteobacteria.o__Sphingomonadales.f__Sphingomonadaceae.g__Sphingobium	1.397463809			-
d__Bacteria.p__Firmicutes.c__Clostridia.o__Lachnospirales.f__Lachnospiraceae.g__Blautia	2.319284561	H	3.4249893	1.31E-06
d__Bacteria.p__Firmicutes.c__Clostridia.o__Eubacteriales.f__Eubacteriaceae	1.31189894			-
d__Bacteria.p__Deinococcota.c__Deinococci.o__Thermales.f__Thermaceae.g__Thermus	1.143143182			-
d__Bacteria.p__Cyanobacteria.c__Vampirivibrionia.o__Gastranaerophilales	1.669715255			-
d__Bacteria.p__Firmicutes.c__Clostridia.o__Eubacteriales	1.31189894			-
d__Bacteria.p__Deinococcota.c__Deinococci.o__Thermales	1.143143182			-
d__Bacteria.p__Actinobacteriota.c__Actinobacteria	4.968828123	CC	4.4141732	0.002257
d__Bacteria.p__Firmicutes.c__Clostridia.o__Oscillospirales.f__Ruminococcaceae.g__Subdoligranulum	2.562530769	H	3.2101653	5.18E-05
d__Bacteria.p__Actinobacteriota.c__Coriobacteriia.o__Coriobacteriales.f__Eggerthellaceae.g__Cryptobacterium	1.315670522			-
d__Bacteria.p__Firmicutes.c__Clostridia.o__Lachnospirales.f__Lachnospiraceae.g__Lachnoclostridium	2.4561599	H	3.3168635	4.12E-09
d__Bacteria.p__Firmicutes.c__Bacilli.o__Erysipelotrichales.f__Erysipelatoclostridiaceae.g__Asteroleplasma	0.312768399			-
d__Bacteria.p__Firmicutes.c__Bacilli.o__Staphylococcales.f__Gemellaceae.g__Gemella	4.746006654	CCR	4.4083425	0.003238
d__Bacteria.p__Firmicutes.c__Clostridia.o__Oscillospirales.f__Ruminococcaceae.g__UBA1819	0.643452676			-
d__Bacteria.p__Bacteroidota.c__Bacteroidia.o__Sphingobacteriales.f__env_OPS	1.112568276			-
d__Bacteria.p__Proteobacteria.c__Gammaproteobacteria.o__Pseudomonadales.f__Moraxellaceae.g__Alkanindiges	1.405255112			-
d__Bacteria.p__Deinococcota.c__Deinococci.o__Thermales.f__Thermaceae	1.143143182			-
d__Bacteria.p__Firmicutes.c__Clostridia.o__Clostridiales.f__Clostridiaceae.g__Clostridium_sensu_stricto	1.653488169			-
d__Bacteria.p__Proteobacteria.c__Gammaproteobacteria.o__Aeromonadales.f__Aeromonadaceae.g__Aeromonas	0.656505134			-
d__Bacteria.p__Firmicutes.c__Clostridia.o__Peptostreptococcales_Tissierellales.f__Anaerovoracaceae.g___Eubacterium__saphenum_group	2.415080715			-
d__Bacteria.p__Proteobacteria.c__Gammaproteobacteria.o__Burkholderiales.f__Oxalobacteraceae	1.711505949			-
d__Bacteria.p__Patescibacteria.c__Gracilibacteria.o__JGI_0000069_P22.f__JGI_0000069_P22	1.761175813			-
d__Bacteria.p__Firmicutes.c__Negativicutes.o__Veillonellales_Selenomonadales.f__Selenomonadaceae.g__Mitsuokella	1.486430479			-
d__Bacteria.p__Deinococcota.c__Deinococci	1.445668169			-
d__Bacteria.p__Actinobacteriota.c__Actinobacteria.o__Actinobacteria_unclassified	3.104482434			-
d__Bacteria.p__Proteobacteria.c__Gammaproteobacteria.o__Burkholderiales.f__Sutterellaceae	2.518401121	H	3.2177397	1.71E-10
d__Bacteria.p__Verrucomicrobiota	1.840867504			-
d__Bacteria.p__Proteobacteria.c__Gammaproteobacteria.o__Enterobacterales.f__Yersiniaceae	2.472066545	H	3.0406302	4.67E-11
d__Bacteria.p__Verrucomicrobiota.c__Lentisphaeria.o__Victivallales	0.525969822			-
d__Bacteria.p__Patescibacteria.c__Saccharimonadia.o__Saccharimonadales	4.193710024	H	3.8254	3.79E-18
d__Bacteria.p__Bacteroidota.c__Bacteroidia.o__Flavobacteriales.f__Flavobacteriaceae.g__Maribacter	0.475133633			-
d__Bacteria.p__Patescibacteria.c__Gracilibacteria.o__Absconditabacteriales__SR1_.f__Absconditabacteriales__SR1_.g__Absconditabacteriales__SR1_	3.897290442	H	3.5922456	1.13E-25
d__Bacteria.p__Firmicutes.c__Bacilli.o__Lactobacillales.f__P5D1_392.g__P5D1_392	2.970270434			-
d__Bacteria.p__Bacteroidota.c__Bacteroidia.o__Bacteroidales.f__Rikenellaceae	3.033285862			-

d__Bacteria.p__Chloroflexi.c__Anaerolineae.o__Anaerolineales	0.995635195			-
d__Bacteria.p__Bacteroidota.c__Bacteroidia.o__Bacteroidales.f__F082	1.190523567			-
d__Bacteria.p__Proteobacteria.c__Gammaproteobacteria.o__Pasteurellales.f__Pasteurellaceae.g__Haemophilus	4.94233929	CCR	4.5647773	7.98E-17
d__Bacteria.p__Firmicutes.c__Bacilli.o__Staphylococcales.f__Staphylococcaceae.g__Staphylococcus	1.938555462			-
d__Bacteria.p__Bacteroidota.c__Bacteroidia.o__Bacteroidales.f__Prevotellaceae.g__Prevotella	5.243155877	H	4.6876665	1.51E-08
d__Bacteria.p__Actinobacteriota.c__Coriobacteriia.o__Coriobacteriales.f__Eggerthellaceae.g__DNF00809	0.817659792			-
d__Bacteria.p__Desulfobacterota.c__Desulfobulbia	1.898881825			-
d__Bacteria.p__Proteobacteria.c__Alphaproteobacteria.o__Sphingomonadales.f__Sphingomonadaceae	3.046915821			-
d__Bacteria.p__Firmicutes.c__Bacilli.o__Erysipelotrichales.f__Erysipelotrichaceae.g__Turicibacter	0.95630567			-
d__Bacteria.p__Spirochaetota	3.454496414	H	3.0121296	2.39E-14
d__Bacteria.p__Firmicutes.c__Bacilli.o__Lactobacillales.f__Carnobacteriaceae.g__Dolosigranulum	0.86332286			-
d__Bacteria.p__Actinobacteriota.c__Actinobacteria.o__Micrococcales.f__Microbacteriaceae.g__Microbacteriaceae_unclassified	0			-
d__Bacteria.p__Cyanobacteria.c__Vampirivibrionia	1.669715255			-
d__Bacteria.p__Proteobacteria.c__Gammaproteobacteria.o__Burkholderiales.f__Rhodocyclaceae.g__Dechloromonas	0.145277312			-
d__Bacteria.p__Desulfobacterota.c__Desulfovibrionia.o__Desulfovibrionales.f__Desulfovibrionaceae.g__Mailhella	1.054995862			-
d__Bacteria.p__Elusimicrobiota.c__Endomicrobia	0.525044807			-
d__Bacteria.p__Proteobacteria.c__Gammaproteobacteria.o__Pasteurellales	4.958275987	CCR	4.5774901	1.11E-17
d__Bacteria.p__Actinobacteriota.c__Actinobacteria.o__Propionibacteriales.f__Propionibacteriaceae.g__Propionibacterium	2.095567832			-
d__Bacteria.p__Firmicutes.c__Clostridia.o__Oscillospirales.f__Ruminococcaceae.g__Anaerotruncus	1.549966124			-
d__Bacteria.p__Bacteroidota.c__Bacteroidia.o__Bacteroidales.f__Prevotellaceae.g__Paraprevotella	1.380986076			-
d__Bacteria.p__Bacteroidota.c__Bacteroidia	5.516826855	H	5.0122127	2.75E-16
d__Bacteria.p__Firmicutes.c__Firmicutes_unclassified.o__Firmicutes_unclassified.f__Firmicutes_unclassified.g__Firmicutes_unclassified	1.440520915			-
d__Bacteria.p__Bacteroidota.c__Bacteroidia.o__Bacteroidales.f__Barnesiellaceae	1.765668555			-
d__Bacteria.p__Bacteria_unclassified.c__Bacteria_unclassified	2.987059901			-
d__Bacteria.p__Actinobacteriota.c__Coriobacteriia	3.450941579	H	3.0843799	9.54E-10
d__Bacteria.p__Firmicutes.c__Syntrophomonadia.o__Syntrophomonadales.f__Syntrophomonadaceae.g__Pelospora	0.557163342			-
d__Bacteria.p__Proteobacteria.c__Alphaproteobacteria.o__Rhizobiales.f__Rhizobiaceae.g__Rhizobiaceae_unclassified	2.551383147	CC	3.3253052	3.89E-22
d__Bacteria.p__Proteobacteria.c__Gammaproteobacteria.o__Xanthomonadales.f__Xanthomonadaceae.g__Xanthomonas	0.698970004			-
d__Bacteria.p__Desulfobacterota.c__Desulfovibrionia.o__Desulfovibrionales.f__Desulfovibrionaceae.g__Bilophila	1.365487985			-
d__Bacteria.p__Firmicutes.c__Negativicutes.o__Veillonellales_Selenomonadales.f__Selenomonadaceae.g__Selenomonadaceae_unclassified	2.998757383			-
d__Bacteria.p__Firmicutes.c__Bacilli.o__Erysipelotrichales.f__Erysipelatoclostridiaceae	2.102016952	H	3.5323375	4.30E-08
d__Bacteria.p__Proteobacteria.c__Gammaproteobacteria.o__Burkholderiales.f__Alcaligenaceae.g__Achromobacter	2.334080189	H	3.427058	1.38E-24
d__Bacteria.p__Bacteroidota.c__Bacteroidia.o__Flavobacteriales.f__Weeksellaceae.g__Chryseobacterium	2.24723655			-
d__Bacteria.p__Firmicutes.c__Negativicutes.o__Acidaminococcales	2.6064044			-
d__Bacteria.p__Firmicutes.c__Clostridia.o__Lachnospirales.f__Lachnospiraceae.g__Oribacterium	3.66107201	H	3.0435608	2.63E-07
d__Bacteria.p__Fusobacteriota.c__Fusobacteriia.o__Fusobacteriales.f__Fusobacteriaceae	4.820212394	H	4.2671497	1.39E-14
d__Bacteria.p__Bacteroidota.c__Bacteroidia.o__Cytophagales.f__Spirosomaceae	0.62838893			-
d__Bacteria.p__Firmicutes.c__Clostridia.o__Lachnospirales.f__Lachnospiraceae.g__Marvinbryantia	0.929053819			-
d__Bacteria.p__Proteobacteria.c__Gammaproteobacteria.o__Burkholderiales.f__Burkholderiaceae.g__Lautropia	4.749665657	CCR	4.4361199	1.96E-10
d__Bacteria.p__Proteobacteria.c__Gammaproteobacteria.o__Burkholderiales.f__Methylophilaceae.g__Methylophilus	0.625227856			-
d__Bacteria.p__Proteobacteria.c__Gammaproteobacteria.o__Enterobacterales.f__Enterobacteriaceae.g__Enterobacteriaceae_unclassified	2.028513157			-
d__Bacteria.p__Actinobacteriota.c__Actinobacteria.o__Corynebacteriales.f__Nocardiaceae.g__Rhodococcus	3.33472223	H	3.0545437	6.09E-10
d__Bacteria.p__Desulfobacterota.c__Desulfobulbia.o__Desulfobulbales.f__Desulfobulbaceae.g__Desulfobulbus	1.898881825			-
d__Bacteria.p__Campilobacterota.c__Campylobacteria.o__Campylobacterales.f__Helicobacteraceae.g__Helicobacter	2.697484662			-
d__Bacteria.p__Firmicutes.c__Bacilli.o__Bacillales.f__Bacillaceae.g__Geobacillus	1.207018511			-
d__Bacteria.p__Proteobacteria.c__Alphaproteobacteria.o__Sphingomonadales.f__Sphingomonadaceae.g__Sphingomonas	3.038969115			-
d__Bacteria.p__Bacteroidota.c__Bacteroidia.o__Bacteroidales.f__Tannerellaceae	3.455233266			-
d__Bacteria.p__Proteobacteria.c__Alphaproteobacteria.o__Caulobacterales.f__Hyphomonadaceae	1.758061168			-
d__Bacteria.p__Actinobacteriota.c__Actinobacteria.o__Corynebacteriales.f__Corynebacteriaceae	3.448795731			-
d__Bacteria.p__Actinobacteriota.c__Actinobacteria.o__Micrococcales.f__Micrococcales_unclassified	2.000950837			-
d__Bacteria.p__Firmicutes.c__Bacilli.o__Acholeplasmatales.f__Acholeplasmataceae.g__Acholeplasma	2.680995585			-
d__Bacteria.p__Synergistota.c__Synergistia.o__Synergistales	2.720501877			-
d__Bacteria.p__Firmicutes.c__Clostridia.o__Oscillospirales.f__Butyricicoccaceae.g__UCG_009	1.373043428			-
d__Bacteria.p__Bacteroidota	5.516826855	H	5.0122127	2.75E-16
d__Bacteria.p__Bacteroidota.c__Bacteroidia.o__Bacteroidales.f__Porphyromonadaceae.g__Porphyromonas	4.697542617	H	4.184499	1.20E-15
d__Bacteria.p__Firmicutes.c__Clostridia.o__Lachnospirales.f__Lachnospiraceae.g__Anaerostipes	2.265036938			-

d__Bacteria.p__Firmicutes.c__Bacilli.o__Acholeplasmatales.f__Acholeplasmataceae	2.680995585			-
d__Bacteria.p__Proteobacteria.c__Gammaproteobacteria.o__Pseudomonadales.f__Moraxellaceae.g__Psychrobacter	0.166060918			-
d__Bacteria.p__Chloroflexi.c__Anaerolineae.o__Anaerolineales.f__Anaerolineaceae	0.995635195			-
d__Bacteria.p__Proteobacteria.c__Gammaproteobacteria.o__Burkholderiales.f__Nitrosomonadaceae.g__DSSD61	0			-
d__Bacteria.p__Firmicutes.c__Bacilli.o__Brevibacillales	0.599727159			-
d__Bacteria.p__Bacteroidota.c__Bacteroidia.o__Bacteroidales.f__Prevotellaceae.g__Alloprevotella	4.825864411	H	4.5044025	2.16E-35
d__Bacteria.p__Actinobacteriota.c__Coriobacteriia.o__Coriobacteriales.f__Eggerthellaceae.g__Slackia	1.284269556			-
d__Bacteria.p__Actinobacteriota.c__Actinobacteria.o__Micrococcales.f__Microbacteriaceae	0			-
d__Bacteria.p__Firmicutes.c__Clostridia.o__Oscillospirales.f__Ruminococcaceae.g___Eubacterium__siraeum_group	2.339990603			-
d__Bacteria.p__Firmicutes.c__Bacilli.o__Mycoplasmatales.f__Mycoplasmataceae.g__Mycoplasma	2.727285578			-
d__Bacteria.p__Firmicutes.c__Clostridia.o__Lachnospirales.f__Lachnospiraceae.g__Johnsonella	3.122211196			-
d__Bacteria.p__Firmicutes.c__Clostridia.o__Lachnospirales.f__Lachnospiraceae.g___Ruminococcus__torques_group	2.120432904			-
d__Bacteria.p__Synergistota.c__Synergistia.o__Synergistales.f__Synergistaceae	2.720501877			-
d__Bacteria.p__Desulfobacterota.c__Desulfovibrionia.o__Desulfovibrionales.f__Desulfovibrionaceae	2.505382574			-
d__Bacteria.p__Proteobacteria.c__Alphaproteobacteria.o__Caulobacterales.f__Caulobacteraceae.g__Brevundimonas	1.796921075			-
d__Bacteria.p__Firmicutes.c__Firmicutes_unclassified	1.440520915			-
d__Bacteria.p__Firmicutes.c__Clostridia.o__Oscillospirales.f__UCG_010	1.749901877			-
d__Bacteria.p__Campilobacterota.c__Campylobacteria.o__Campylobacterales.f__Helicobacteraceae.g__Wolinella	1.346352974			-
d__Bacteria.p__Proteobacteria.c__Gammaproteobacteria.o__Burkholderiales.f__Oxalobacteraceae.g__Janthinobacterium	0.846425482			-
d__Bacteria.p__Actinobacteriota.c__Actinobacteria.o__Propionibacteriales.f__Propionibacteriaceae.g__Pseudopropionibacterium	1.827307719			-
d__Bacteria.p__Synergistota	2.720501877			-
d__Bacteria.p__Firmicutes.c__Clostridia.o__Lachnospirales.f__Lachnospiraceae.g__Lachnospiraceae_ND3007_group	2.334353208	H	3.3508166	1.45E-11
d__Bacteria.p__Actinobacteriota.c__Actinobacteria.o__Frankiales	0.890899844			-
d__Bacteria.p__Fusobacteriota.c__Fusobacteriia.o__Fusobacteriales.f__Leptotrichiaceae	5.038809375	CC	4.5267539	0.004177
d__Bacteria.p__Desulfobacterota.c__Desulfovibrionia.o__Desulfovibrionales.f__Desulfomicrobiaceae	0.955275315			-
d__Bacteria.p__Proteobacteria.c__Gammaproteobacteria.o__Enterobacterales.f__Enterobacteriaceae.g__Escherichia_Shigella	2.295252849	H	3.2378861	3.11E-07
d__Bacteria.p__Bacteroidota.c__Bacteroidia.o__Flavobacteriales.f__Flavobacteriaceae.g__Capnocytophaga	4.251371549	H	3.541349	0.000126
d__Bacteria.p__Firmicutes.c__Clostridia.o__Peptostreptococcales_Tissierellales.f__Anaerovoracaceae.g__Anaerovoracaceae_Family_XIII_AD3011_group	0.768743782			-
d__Bacteria.p__Bacteroidota.c__Bacteroidia.o__Sphingobacteriales.f__Sphingobacteriales_unclassified.g__Sphingobacteriales_unclassified	0.770852012			-
d__Bacteria.p__Proteobacteria.c__Gammaproteobacteria.o__Pseudomonadales	4.208881494	H	3.9041018	1.01E-28
d__Bacteria.p__Firmicutes.c__Negativicutes.o__Veillonellales_Selenomonadales.f__Selenomonadaceae.g__Megamonas	2.85942996	H	3.1149896	3.66E-12
d__Bacteria.p__Synergistota.c__Synergistia	2.720501877			-
d__Bacteria.p__Proteobacteria.c__Gammaproteobacteria.o__Pasteurellales.f__Pasteurellaceae.g__Pasteurellaceae_unclassified	2.617250036			-
d__Bacteria.p__Actinobacteriota.c__Coriobacteriia.o__Coriobacteriales.f__Eggerthellaceae	1.743789648			-
d__Bacteria.p__Firmicutes.c__Clostridia.o__Peptostreptococcales_Tissierellales.f__Peptostreptococcaceae.g__Intestinibacter	1.235348055			-
d__Bacteria.p__Chloroflexi	0.995635195			-
d__Bacteria.p__Firmicutes.c__Bacilli.o__Erysipelotrichales.f__Erysipelatoclostridiaceae.g__UCG_004	1.054182649			-
d__Bacteria.p__Deferribacterota	2.440335489			-
d__Bacteria.p__Bacteroidota.c__Bacteroidia.o__Bacteroidia_unclassified.f__Bacteroidia_unclassified	0.534207478			-
d__Bacteria.p__Bacteria_unclassified.c__Bacteria_unclassified.o__Bacteria_unclassified	2.987059901			-
d__Bacteria.p__Firmicutes.c__Bacilli.o__Erysipelotrichales.f__Erysipelatoclostridiaceae.g__Erysipelatoclostridiaceae	0.26211193			-
d__Bacteria.p__Firmicutes.c__Clostridia.o__Peptococcales	2.582014636	H	3.5545261	1.64E-21
d__Bacteria.p__Actinobacteriota.c__Actinobacteria.o__Bifidobacteriales.f__Bifidobacteriaceae.g__Alloscardovia	1.760584021			-
d__Bacteria.p__Patescibacteria.c__Saccharimonadia.o__Saccharimonadales.f__Saccharimonadales_unclassified	1.306169635			-
d__Bacteria.p__Firmicutes.c__Clostridia.o__Clostridia_unclassified.f__Clostridia_unclassified.g__Clostridia_unclassified	0.02413368			-
d__Bacteria.p__Firmicutes.c__Clostridia.o__Oscillospirales.f__Oscillospiraceae.g__Oscillibacter	2.239978371			-
d__Bacteria.p__Bacteroidota.c__Bacteroidia.o__Bacteroidales.f__Porphyromonadaceae	4.697542617	H	4.184499	1.20E-15
d__Bacteria.p__Bacteroidota.c__Bacteroidia.o__Flavobacteriales.f__Flavobacteriaceae	4.251511163	H	3.5415481	0.000127
d__Bacteria.p__Firmicutes.c__Clostridia.o__Peptostreptococcales_Tissierellales.f__Peptostreptococcaceae.g__Peptoanaerobacter	1.771797386			-
d__Bacteria.p__Bacteroidota.c__Bacteroidia.o__Bacteroidales.f__Prevotellaceae.g__Prevotellaceae	0.811479251			-
d__Bacteria.p__Firmicutes.c__Negativicutes.o__Veillonellales_Selenomonadales.f__Veillonellaceae.g__Veillonella	5.082901734			-
d__Bacteria.p__Deinococcota	1.445668169			-
d__Bacteria.p__Proteobacteria.c__Alphaproteobacteria.o__Rhizobiales.f__Beijerinckiaceae.g__Methylobacterium_Methylorubrum	1.365086665			-
d__Bacteria.p__Firmicutes.c__Bacilli.o__Izemoplasmatales.f__Izemoplasmatales.g__Izemoplasmatales	1.43513877			-
d__Bacteria.p__Elusimicrobiota.c__Elusimicrobia.o__Lineage_IV	0.299476168			-

d__Bacteria.p__Proteobacteria.c__Gammaproteobacteria.o__Burkholderiales.f__Rhodocyclaceae.g__Methyloversatilis	0.504033061			-
d__Bacteria.p__Desulfobacterota	2.61110852	H	3.1929453	6.18E-14
d__Bacteria.p__Bacteroidota.c__Bacteroidia.o__Flavobacteriales.f__Weeksellaceae.g__Moheibacter	1.268343914			-
d__Bacteria.p__Firmicutes.c__Bacilli.o__Lactobacillales.f__Enterococcaceae.g__Enterococcus	1.174433645			-
d__Bacteria.p__Firmicutes.c__Clostridia.o__Oscillospirales.f__Oscillospiraceae.g__UCG_002	2.630217142	H	3.0339927	3.90E-11
d__Bacteria.p__Firmicutes.c__Clostridia.o__Oscillospirales.f__Oscillospiraceae.g__UCG_003	1.945995834			-
d__Bacteria.p__Firmicutes.c__Clostridia.o__Oscillospirales.f___Eubacterium__coprostanoligenes_group	2.576908738			-
d__Bacteria.p__Bacteroidota.c__Bacteroidia.o__Bacteroidales.f__Paludibacteraceae	2.876106448			-
d__Bacteria.p__Bacteroidota.c__Bacteroidia.o__Bacteroidales.f__Tannerellaceae.g__Parabacteroides	2.535755121	H	3.191488	1.23E-08
d__Bacteria.p__Proteobacteria.c__Alphaproteobacteria.o__Rickettsiales.f__Mitochondria	1.871556943			-
d__Bacteria.p__Firmicutes.c__Clostridia.o__Oscillospirales.f__Ruminococcaceae.g__CAG_352	1.960742509			-
d__Bacteria.p__Firmicutes.c__Bacilli.o__Erysipelotrichales.f__Erysipelatoclostridiaceae.g__Catenibacterium	0.632221477			-
d__Bacteria.p__Proteobacteria.c__Gammaproteobacteria.o__Burkholderiales.f__Comamonadaceae.g__Schlegelella	1.017490732			-
d__Bacteria.p__Firmicutes.c__Clostridia.o__Eubacteriales.f__Eubacteriaceae.g__Pseudoramibacter	1.31189894			-
d__Bacteria.p__Actinobacteriota.c__Actinobacteria.o__Propionibacteriales.f__Propionibacteriaceae	2.3979276			-
d__Bacteria.p__Firmicutes.c__Clostridia.o__Oscillospirales.f__Ruminococcaceae	3.706035882	H	3.3878086	5.52E-15
d__Bacteria.p__Bacteroidota.c__Bacteroidia.o__Flavobacteriales.f__Weeksellaceae.g__Chishuiella	1.321332074			-
d__Bacteria.p__Proteobacteria.c__Alphaproteobacteria.o__Rhizobiales.f__Devosiaceae.g__Devosia	0.572839787			-
d__Bacteria.p__Actinobacteriota	4.980846659	CC	4.4136167	0.00214
d__Bacteria.p__Proteobacteria.c__Alphaproteobacteria.o__Sphingomonadales	3.046915821			-
d__Bacteria.p__Firmicutes.c__Clostridia.o__Oscillospirales.f__Oscillospiraceae.g__Oscillospiraceae_uncultured	2.404772647			-
d__Bacteria.p__Bacteroidota.c__Bacteroidia.o__SM1A07.f__SM1A07.g__SM1A07	1.053627142			-
d__Bacteria.p__Firmicutes.c__Clostridia.o__Peptostreptococcales_Tissierellales.f__Peptostreptococcaceae.g___Eubacterium__yurii_group	2.308030554			-
d__Bacteria.p__Firmicutes.c__Clostridia.o__Clostridiales.f__Clostridiaceae.g__Clostridiaceae_unclassified	0.51568465			-
d__Bacteria.p__Proteobacteria.c__Gammaproteobacteria.o__Burkholderiales.f__Sutterellaceae.g__Parasutterella	2.259815645	H	3.4690302	3.30E-10
d__Bacteria.p__Firmicutes.c__Clostridia.o__Lachnospirales.f__Lachnospiraceae.g__Stomatobaculum	3.607682451	H	3.1690664	6.34E-06
d__Bacteria.p__Bacteroidota.c__Bacteroidia.o__Flavobacteriales.f__Weeksellaceae	3.282825959			-
d__Bacteria.p__Proteobacteria.c__Alphaproteobacteria.o__Rhodospirillales	2.241848759			-
d__Bacteria.p__Campilobacterota.c__Campylobacteria.o__Campylobacterales.f__Campylobacteraceae	3.990877424	H	3.5327816	1.88E-15
d__Bacteria.p__Proteobacteria.c__Gammaproteobacteria.o__Cardiobacteriales	3.215095978	CCR	3.2642372	1.33E-07
d__Bacteria.p__Firmicutes.c__Clostridia.o__Oscillospirales.f__Butyricicoccaceae.g__Butyricicoccus	1.651762447			-
d__Bacteria.p__Firmicutes.c__Bacilli.o__Erysipelotrichales	3.612668837	H	3.1995754	8.31E-15
d__Bacteria.p__Firmicutes.c__Bacilli.o__Erysipelotrichales.f__Erysipelotrichaceae.g__Erysipelotrichaceae_UCG_006	1.312509813			-
d__Bacteria.p__Proteobacteria.c__Gammaproteobacteria.o__Pasteurellales.f__Pasteurellaceae.g__Aggregatibacter	3.611780947	H	3.2744165	8.46E-26
d__Bacteria.p__Proteobacteria.c__Alphaproteobacteria.o__Rhodobacterales.f__Rhodobacteraceae.g__Rubellimicrobium	0.579783597			-
d__Bacteria.p__Firmicutes.c__Bacilli.o__Lactobacillales.f__Aerococcaceae.g__Abiotrophia	3.627712023	CCR	3.2680241	0.022617
d__Bacteria.p__Proteobacteria.c__Gammaproteobacteria.o__Burkholderiales.f__Burkholderiaceae.g__Ralstonia	0.748741621			-
d__Bacteria.p__Proteobacteria.c__Alphaproteobacteria.o__Rhodobacterales.f__Rhodobacteraceae	1.246920431			-
d__Bacteria.p__Planctomycetota.c__Planctomycetes.o__Pirellulales.f__Pirellulaceae.g__Rhodopirellula	0.198264238			-
d__Bacteria.p__Firmicutes.c__Clostridia.o__Lachnospirales.f__Lachnospiraceae.g__A2	2.099951806			-
d__Bacteria.p__Fusobacteriota.c__Fusobacteriia.o__Fusobacteriales.f__Fusobacteriaceae.g__Fusobacterium	4.820212394	H	4.2671497	1.39E-14
d__Bacteria.p__Deinococcota.c__Deinococci.o__Deinococcales	1.146128036			-
d__Bacteria.p__Patescibacteria.c__Gracilibacteria	3.929411992	H	3.6225662	3.48E-26
d__Bacteria.p__Campilobacterota.c__Campylobacteria	4.013369376	H	3.5601255	5.89E-16
d__Bacteria.p__Firmicutes.c__Bacilli.o__Paenibacillales	0.750518962			-
d__Bacteria.p__Firmicutes.c__Clostridia.o__Lachnospirales.f__Lachnospiraceae.g__Fusicatenibacter	3.237349813	H	3.0364156	2.17E-18
d__Bacteria.p__Actinobacteriota.c__Coriobacteriia.o__Coriobacteriales.f__Atopobiaceae	3.438785251	H	3.0765696	4.73E-09
d__Bacteria.p__Firmicutes.c__Clostridia.o__Oscillospirales.f__Oscillospirales_unclassified	0			-
d__Bacteria.p__Bacteroidota.c__Bacteroidia.o__Bacteroidales.f__Prevotellaceae	5.385730659	H	4.8948212	9.12E-13
d__Bacteria.p__Actinobacteriota.c__Actinobacteria.o__Bifidobacteriales.f__Bifidobacteriaceae	3.088599258			-
d__Bacteria.p__Firmicutes.c__Bacilli.o__Bacilli_unclassified.f__Bacilli_unclassified	2.144017919			-
d__Bacteria.p__Proteobacteria.c__Gammaproteobacteria.o__Burkholderiales.f__Comamonadaceae.g__Delftia	1.397940009			-
d__Bacteria.p__Bacteroidota.c__Bacteroidia.o__Flavobacteriales.f__Weeksellaceae.g__Bergeyella	3.230881176			-
d__Bacteria.p__Firmicutes.c__Bacilli.o__Lactobacillales	5.438022874	CCR	4.9951329	5.51E-06
d__Bacteria.p__Planctomycetota	0.516629796			-
d__Bacteria.p__Desulfobacterota.c__Desulfovibrionia	2.517450403			-
d__Bacteria.p__Firmicutes.c__Clostridia.o__Clostridia_vadinBB60_group	2.703408075			-

d__Bacteria.p__Actinobacteriota.c__Actinobacteria.o__Actinomycetales.f__Actinomycetaceae.g__Actinomycetaceae_unclassified	2.013875655			-
d__Bacteria.p__Campilobacterota.c__Campylobacteria.o__Campylobacterales.f__Helicobacteraceae	2.716414774			-
d__Bacteria.p__Proteobacteria.c__Gammaproteobacteria.o__Pasteurellales.f__Pasteurellaceae	4.958275987	CCR	4.5774901	1.11E-17
d__Bacteria.p__Proteobacteria.c__Gammaproteobacteria.o__Enterobacterales.f__Yersiniaceae.g__Serratia	2.472066545	H	3.1030001	4.67E-11
d__Bacteria.p__Actinobacteriota.c__Actinobacteria.o__Bifidobacteriales.f__Bifidobacteriaceae.g__Gardnerella	0.513254097			-
d__Bacteria.p__Firmicutes.c__Firmicutes_unclassified.o__Firmicutes_unclassified.f__Firmicutes_unclassified	1.440520915			-
d__Bacteria.p__Firmicutes.c__Clostridia.o__Oscillospirales.f___Eubacterium__coprostanoligenes_group.g___Eubacterium__coprostanoligenes_group	2.576908738			-
d__Bacteria.p__Desulfobacterota.c__Desulfobulbia.o__Desulfobulbales	1.898881825			-
d__Bacteria.p__Firmicutes.c__Clostridia.o__Lachnospirales.f__Lachnospiraceae.g__Tuzzerella	1.345233658			-
d__Bacteria.p__Patescibacteria.c__Saccharimonadia	4.193710024	H	3.8254	3.79E-18
d__Bacteria.p__Actinobacteriota.c__Actinobacteria.o__Corynebacteriales.f__Corynebacteriaceae.g__Corynebacterium	3.436623741			-
d__Bacteria.p__Actinobacteriota.c__Actinobacteria.o__Bifidobacteriales	3.088599258			-
d__Bacteria.p__Bacteroidota.c__Bacteroidia.o__Bacteroidia_unclassified.f__Bacteroidia_unclassified.g__Bacteroidia_unclassified	0.534207478			-
d__Bacteria.p__Elusimicrobiota.c__Elusimicrobia	0.299476168			-
d__Bacteria.p__Firmicutes.c__Clostridia.o__Oscillospirales.f__Ruminococcaceae.g__Negativibacillus	1.589311168			-
d__Bacteria.p__Firmicutes.c__Bacilli.o__Erysipelotrichales.f__Erysipelotrichaceae	3.599056878	H	3.180894	4.67E-14
d__Bacteria.p__Actinobacteriota.c__Coriobacteriia.o__Coriobacteriales.f__Coriobacteriaceae.g__Collinsella	1.352733653			-
d__Bacteria.p__Fusobacteriota	5.147631904			-
d__Bacteria.p__Proteobacteria.c__Alphaproteobacteria.o__Caulobacterales.f__Caulobacteraceae.g__Asticcacaulis	0.798354636			-
d__Bacteria.p__Firmicutes.c__Clostridia.o__Oscillospirales.f__UCG_010.g__UCG_010	1.749901877			-
d__Bacteria.p__Firmicutes.c__Bacilli.o__Erysipelotrichales.f__Erysipelotrichaceae.g__Holdemania	0.713010262			-
d__Bacteria.p__Firmicutes.c__Bacilli.o__RF39	2.411643753			-
d__Bacteria.p__Elusimicrobiota.c__Endomicrobia.o__Endomicrobiales	0.525044807			-
d__Bacteria.p__Proteobacteria.c__Alphaproteobacteria.o__Rhodospirillales.f__Rhodospirillales_uncultured.g__Rhodospirillales_uncultured	2.241848759			-
d__Bacteria.p__Proteobacteria.c__Gammaproteobacteria.o__Pseudomonadales.f__Pseudomonadaceae	3.716747211	H	3.4198539	7.11E-29
d__Bacteria.p__Bacteroidota.c__Bacteroidia.o__Cytophagales.f__Spirosomaceae.g__Spirosoma	0.62838893			-
d__Bacteria.p__Proteobacteria.c__Alphaproteobacteria.o__Sphingomonadales.f__Sphingomonadaceae.g__Novosphingobium	2.095002072			-
d__Bacteria.p__Bacteroidota.c__Bacteroidia.o__Bacteroidales.f__Rikenellaceae.g__Rikenella	1.640268426			-
d__Bacteria.p__Firmicutes.c__Bacilli.o__Lactobacillales.f__Lactobacillales_unclassified.g__Lactobacillales_unclassified	3.353430755			-
d__Bacteria.p__Firmicutes.c__Bacilli.o__Lactobacillales.f__Streptococcaceae.g__Streptococcus	5.400064995	CCR	4.9665776	2.02E-05
d__Bacteria.p__Firmicutes.c__Syntrophomonadia.o__Syntrophomonadales	0.557163342			-
d__Bacteria.p__Firmicutes.c__Bacilli.o__Erysipelotrichales.f__Erysipelatoclostridiaceae.g__Eggerthia	1.187722109			-
d__Bacteria.p__Proteobacteria.c__Gammaproteobacteria.o__Burkholderiales.f__Neisseriaceae.g__Neisseria	5.215913965	H	4.3652894	3.25E-05
d__Bacteria.p__Bacteroidota.c__Bacteroidia.o__Bacteroidales.f__Bacteroidales_unclassified.g__Bacteroidales_unclassified	1.514547753			-
d__Bacteria.p__Actinobacteriota.c__Actinobacteria.o__Actinomycetales.f__Actinomycetaceae.g__Actinotignum	0.794886208			-
d__Bacteria.p__Proteobacteria.c__Alphaproteobacteria.o__Rhizobiales.f__Hyphomicrobiaceae	0.6734159			-
d__Bacteria.p__Proteobacteria.c__Alphaproteobacteria.o__Rhizobiales.f__Devosiaceae.g__Pelagibacterium	2.774906741			-
d__Bacteria.p__Bacteroidota.c__Bacteroidia.o__Bacteroidales.f__Marinifilaceae.g__Odoribacter	2.155596276			-
d__Bacteria.p__Verrucomicrobiota.c__Lentisphaeria.o__Victivallales.f__Victivallaceae	0.525969822			-
d__Bacteria.p__Actinobacteriota.c__Actinobacteria.o__Bifidobacteriales.f__Bifidobacteriaceae.g__Bifidobacterium	2.966497072			-
d__Bacteria.p__Patescibacteria.c__Gracilibacteria.o__Absconditabacteriales__SR1_	3.897290442	H	3.5933173	1.13E-25
d__Bacteria.p__Proteobacteria.c__Gammaproteobacteria.o__Cardiobacteriales.f__Cardiobacteriaceae	3.215095978	CCR	3.2473845	1.33E-07
d__Bacteria.p__Firmicutes.c__Clostridia.o__Peptostreptococcales_Tissierellales.f__Peptostreptococcales_Tissierellales.g__Anaerococcus	1.166683791			-
d__Bacteria.p__Firmicutes.c__Clostridia.o__Christensenellales	2.743408963			-
d__Bacteria.p__Firmicutes.c__Clostridia.o__Peptostreptococcales_Tissierellales.f__Peptostreptococcaceae.g__Peptostreptococcaceae_unclassified	2.014250446	H	3.3969697	1.05E-06
d__Bacteria.p__Proteobacteria.c__Gammaproteobacteria.o__Alteromonadales.f__Idiomarinaceae.g__Aliidiomarina	1.75221313			-
d__Bacteria.p__Planctomycetota.c__Planctomycetes.o__Pirellulales	0.516629796			-
d__Bacteria.p__Firmicutes.c__Clostridia.o__Oscillospirales.f__Oscillospiraceae.g__UCG_005	2.318867943			-
d__Bacteria.p__Firmicutes.c__Clostridia.o__Lachnospirales.f__Lachnospiraceae.g__Butyrivibrio	2.520324421			-
d__Bacteria.p__Desulfobacterota.c__Desulfovibrionia.o__Desulfovibrionales	2.517450403			-
d__Bacteria.p__Actinobacteriota.c__Actinobacteria.o__Micrococcales.f__Micrococcaceae.g__Rothia	4.536311888			-
d__Bacteria.p__Bacteroidota.c__Bacteroidia.o__Bacteroidales.f__Prevotellaceae.g__Prevotellaceae_Ga6A1_group	1.528733105			-
d__Bacteria.p__Firmicutes.c__Clostridia.o__Clostridia_vadinBB60_group.f__Clostridia_vadinBB60_group.g__Clostridia_vadinBB60_group	2.703408075			-
d__Bacteria.p__Firmicutes.c__Clostridia.o__Peptostreptococcales_Tissierellales.f__Anaerovoracaceae.g__Anaerovoracaceae_uncultured	1.985618909	H	3.4636588	4.37E-07

d__Bacteria.p__Firmicutes.c__Bacilli.o__Bacilli_unclassified.f__Bacilli_unclassified.g__Bacilli_unclassified	2.144017919			-
d__Bacteria.p__Proteobacteria.c__Alphaproteobacteria.o__Caulobacterales.f__Caulobacteraceae.g__Phenylobacterium	0.721008364			-
d__Bacteria.p__Proteobacteria.c__Gammaproteobacteria.o__Burkholderiales.f__Sutterellaceae.g__Sutterella	2.170324591			-
d__Bacteria.p__Firmicutes.c__Clostridia.o__Peptostreptococcales_Tissierellales.f__Peptostreptococcales_Tissierellales	3.344811767	H	3.0602201	1.32E-20
d__Bacteria.p__Desulfobacterota.c__Desulfovibrionia.o__Desulfovibrionales.f__Desulfomicrobiaceae.g__Desulfomicrobium	0.955275315			-
d__Bacteria.p__Proteobacteria.c__Gammaproteobacteria.o__Burkholderiales.f__Comamonadaceae.g__Acidovorax	2.487239409			-
d__Bacteria.p__Firmicutes.c__Syntrophomonadia.o__Syntrophomonadales.f__Syntrophomonadaceae	0.557163342			-
d__Bacteria.p__Firmicutes.c__Clostridia.o__Clostridia_UCG_014	3.571943298	H	3.2027096	4.61E-18
d__Bacteria.p__Firmicutes.c__Bacilli.o__Erysipelotrichales.f__Erysipelotrichaceae.g__Holdemanella	1.218047597			-
d__Bacteria.p__Bacteroidota.c__Bacteroidia.o__Bacteroidales.f__p_2534_18B5_gut_group.g__p_2534_18B5_gut_group	1.108178297			-
d__Bacteria.p__Proteobacteria.c__Gammaproteobacteria.o__Burkholderiales	5.33959235	CCR	4.5767478	0.000129
d__Bacteria.p__Firmicutes.c__Clostridia.o__Lachnospirales.f__Lachnospiraceae.g__Roseburia	2.919007075	H	3.0066053	4.85E-17
d__Bacteria.p__Proteobacteria.c__Alphaproteobacteria.o__Rickettsiales	1.871556943			-
d__Bacteria.p__Firmicutes.c__Clostridia.o__Peptostreptococcales_Tissierellales.f__Anaerovoracaceae.g___Eubacterium__nodatum_group	3.389098972			-
d__Bacteria	6			-
d__Bacteria.p__Firmicutes.c__Bacilli.o__Mycoplasmatales.f__Mycoplasmataceae	2.727285578			-
d__Bacteria.p__Bacteroidota.c__Bacteroidia.o__Sphingobacteriales.f__Sphingobacteriales_unclassified	0.770852012			-
d__Bacteria.p__Firmicutes.c__Clostridia.o__Lachnospirales.f__Lachnospiraceae.g___Ruminococcus__gauvreauii_group	1.856685744			-
d__Bacteria.p__Firmicutes.c__Clostridia.o__Lachnospirales.f__Lachnospiraceae.g___Ruminococcus__gnavus_group	1.643170575			-
d__Bacteria.p__Firmicutes.c__Clostridia.o__Oscillospirales.f__Ruminococcaceae.g__Ruminococcaceae_uncultured	1.95469036			-
d__Bacteria.p__Patescibacteria.c__Gracilibacteria.o__Gracilibacteria	2.73900693			-
d__Bacteria.p__Proteobacteria.c__Gammaproteobacteria.o__Pseudomonadales.f__Moraxellaceae	4.040106512	H	3.7353504	4.12E-18
d__Bacteria.p__Firmicutes.c__Bacilli.o__Lactobacillales.f__Lactobacillaceae.g__Pediococcus	0			-
d__Bacteria.p__Proteobacteria.c__Gammaproteobacteria.o__Pseudomonadales.f__Moraxellaceae.g__Moraxella	3.989916789			-
d__Bacteria.p__Firmicutes.c__Clostridia.o__Peptostreptococcales_Tissierellales.f__Peptostreptococcales_Tissierellales.g__Peptostreptococcales_Tissierellales_uncultured	1.51054501			-
d__Bacteria.p__Firmicutes.c__Clostridia.o__Lachnospirales.f__Lachnospiraceae.g__GCA_900066575	1.453427555			-
d__Bacteria.p__Firmicutes.c__Clostridia.o__Monoglobales.f__Monoglobaceae.g__Monoglobus	2.059752694			-
d__Bacteria.p__Spirochaetota.c__Spirochaetia.o__Spirochaetales.f__Spirochaetaceae.g__Treponema	3.453788862	H	3.0213586	1.08E-14
d__Bacteria.p__Proteobacteria.c__Alphaproteobacteria.o__Caulobacterales.f__Caulobacteraceae.g__Caulobacter	1.608755193			-
d__Bacteria.p__Verrucomicrobiota.c__Lentisphaeria.o__Victivallales.f__Victivallaceae.g__Victivallis	0.525969822			-
d__Bacteria.p__Cyanobacteria.c__Cyanobacteriia.o__Chloroplast.f__Chloroplast	2.655493664			-
d__Bacteria.p__Firmicutes.c__Clostridia.o__Christensenellales.f__Christensenellaceae	2.743408963			-
d__Bacteria.p__Proteobacteria.c__Gammaproteobacteria.o__Burkholderiales.f__Chromobacteriaceae	0			-
d__Bacteria.p__Firmicutes.c__Clostridia.o__Lachnospirales.f__Lachnospiraceae.g__Howardella	1.040309656			-
d__Bacteria.p__Firmicutes.c__Clostridia.o__Peptococcales.f__Peptococcaceae.g__Peptococcus	2.543606916	H	3.4210447	3.45E-19
d__Bacteria.p__Firmicutes.c__Clostridia.o__Lachnospirales.f__Lachnospiraceae.g__Lachnospira	2.581965904	H	3.2728294	7.44E-10
d__Bacteria.p__Firmicutes.c__Bacilli.o__Lactobacillales.f__P5D1_392	2.970270434			-
d__Bacteria.p__Elusimicrobiota	0.727773562			-
d__Bacteria.p__Firmicutes.c__Bacilli.o__Erysipelotrichales.f__Erysipelotrichaceae.g__Erysipelotrichaceae_uncultured	0.943423847			-
d__Bacteria.p__Patescibacteria.c__Gracilibacteria.o__Absconditabacteriales__SR1_.f__Absconditabacteriales__SR1_	3.897290442	H	3.5961296	1.13E-25
d__Bacteria.p__Bacteroidota.c__Bacteroidia.o__Bacteroidales.f__Prevotellaceae.g__Prevotellaceae_NK3B31_group	2.69113835			-
d__Bacteria.p__Firmicutes.c__Bacilli.o__Staphylococcales.f__Staphylococcaceae	1.938555462			-
d__Bacteria.p__Firmicutes.c__Clostridia.o__Peptostreptococcales_Tissierellales.f__Peptostreptococcaceae.g__Peptostreptococcus	3.541427067	CC	3.1273639	2.02E-05
d__Bacteria.p__Firmicutes	5.629544682	CCR	4.9980954	3.34E-07
d__Bacteria.p__Firmicutes.c__Bacilli.o__Brevibacillales.f__Brevibacillaceae.g__Brevibacillus	0.599727159			-
d__Bacteria.p__Firmicutes.c__Bacilli.o__Erysipelotrichales.f__Erysipelotrichaceae.g__Solobacterium	3.588442189	H	3.2043404	2.69E-13
d__Bacteria.p__Proteobacteria.c__Gammaproteobacteria.o__Burkholderiales.f__Neisseriaceae.g__Neisseriaceae_unclassified	3.372427727			-
d__Bacteria.p__Chloroflexi.c__Anaerolineae.o__Anaerolineales.f__Anaerolineaceae.g__Flexilinea	0.995635195			-
d__Bacteria.p__Bacteroidota.c__Bacteroidia.o__Bacteroidales.f__Marinifilaceae	2.164713909			-
d__Bacteria.p__Firmicutes.c__Clostridia.o__Peptococcales.f__Peptococcaceae	2.582014636	H	3.1567532	1.64E-21
d__Bacteria.p__Firmicutes.c__Clostridia.o__Peptostreptococcales_Tissierellales.f__Peptostreptococcaceae	3.721441974	H	3.2628521	2.60E-09
d__Bacteria.p__Bacteroidota.c__Bacteroidia.o__Bacteroidales.f__Muribaculaceae	3.63868461	H	3.3539332	9.08E-18
d__Bacteria.p__Actinobacteriota.c__Coriobacteriia.o__Coriobacteriales.f__Coriobacteriaceae	1.352733653			-
d__Bacteria.p__Synergistota.c__Synergistia.o__Synergistales.f__Synergistaceae.g__Pyramidobacter	1.361727836			-
d__Bacteria.p__Firmicutes.c__Clostridia.o__Lachnospirales.f__Lachnospiraceae.g__Dorea	1.42975228			-

d__Bacteria.p__Proteobacteria.c__Gammaproteobacteria.o__Burkholderiales.f__Alcaligenaceae.g__Alcaligenes	2.114086503	H	3.591732	3.51E-18
d__Bacteria.p__Elusimicrobiota.c__Elusimicrobia.o__Lineage_IV.f__Lineage_IV.g__Lineage_IV	0.299476168			-
d__Bacteria.p__Firmicutes.c__Bacilli.o__Lactobacillales.f__Carnobacteriaceae	4.203495235	CCR	3.5974183	0.000291
d__Bacteria.p__Firmicutes.c__Negativicutes.o__Veillonellales_Selenomonadales.f__Selenomonadaceae.g__Selenomonadaceae_uncultured	2.758890274			-
d__Bacteria.p__Planctomycetota.c__Planctomycetes.o__Pirellulales.f__Pirellulaceae.g__Pirellula	0.232269865			-
d__Bacteria.p__Proteobacteria.c__Gammaproteobacteria.o__Burkholderiales.f__Comamonadaceae	2.860919529	H	3.0348739	1.44E-11
d__Bacteria.p__Firmicutes.c__Bacilli.o__Izemoplasmatales.f__Izemoplasmatales	1.43513877			-
d__Bacteria.p__Patescibacteria.c__Saccharimonadia.o__Saccharimonadales.f__Saccharimonadaceae.g__Candidatus_Saccharimonas	3.099098298			-
d__Bacteria.p__Firmicutes.c__Syntrophomonadia	0.557163342			-
d__Bacteria.p__Actinobacteriota.c__Actinobacteria.o__Actinomycetales.f__Actinomycetaceae.g__Mobiluncus	1.594629288			-
d__Bacteria.p__Firmicutes.c__Clostridia.o__Oscillospirales.f__Ruminococcaceae.g__Ruminococcaceae_Incertae_Sedis	1.922428859			-
d__Bacteria.p__Actinobacteriota.c__Actinobacteria.o__Pseudonocardiales	0			-
d__Bacteria.p__Bacteria_unclassified.c__Bacteria_unclassified.o__Bacteria_unclassified.f__Bacteria_unclassified	2.987059901			-
d__Bacteria.p__Firmicutes.c__Clostridia.o__Oscillospirales	3.858286637	H	3.5405953	5.05E-17
d__Bacteria.p__Cyanobacteria.c__Cyanobacteriia.o__Cyanobacteriales.f__Chroococcidiopsaceae	0.496336485			-
d__Bacteria.p__Firmicutes.c__Clostridia.o__Lachnospirales.f__Lachnospiraceae.g___Eubacterium__hallii_group	1.739628845			-
d__Bacteria.p__Patescibacteria.c__Saccharimonadia.o__Saccharimonadales.f__Saccharimonadaceae.g__TM7x	4.00687264	H	3.6532011	6.36E-16
d__Bacteria.p__Firmicutes.c__Bacilli.o__Lactobacillales.f__Enterococcaceae	1.174433645			-
d__Bacteria.p__Proteobacteria.c__Alphaproteobacteria.o__Caulobacterales	2.039640698	H	3.5182783	8.67E-06
d__Bacteria.p__Firmicutes.c__Clostridia.o__Lachnospirales.f__Lachnospiraceae.g__Shuttleworthia	2.776939884			-
d__Bacteria.p__Proteobacteria.c__Alphaproteobacteria.o__Rhizobiales.f__Rhizobiaceae.g__Aliihoeflea	1.341068472			-
d__Bacteria.p__Firmicutes.c__Clostridia.o__Clostridia_UCG_014.f__Clostridia_UCG_014	3.571943298	H	3.2027096	4.61E-18
d__Bacteria.p__Verrucomicrobiota.c__Verrucomicrobiae.o__Verrucomicrobiales	1.819308864			-
d__Bacteria.p__Firmicutes.c__Bacilli.o__Lactobacillales.f__Carnobacteriaceae.g__Granulicatella	4.203495235	CCR	3.5980156	0.000294
d__Bacteria.p__Proteobacteria.c__Alphaproteobacteria.o__Rhizobiales.f__Devosiaceae	2.777625416			-
d__Bacteria.p__Firmicutes.c__Bacilli.o__Erysipelotrichales.f__Erysipelotrichaceae.g__Bulleidia	1.517290177			-
d__Bacteria.p__Proteobacteria.c__Gammaproteobacteria.o__Enterobacterales	2.778636975			-
d__Bacteria.p__Firmicutes.c__Bacilli.o__Lactobacillales.f__Aerococcaceae	3.627712023	CCR	3.2667449	0.028159
d__Bacteria.p__Proteobacteria.c__Gammaproteobacteria.o__Burkholderiales.f__Methylophilaceae	0.625227856			-
d__Bacteria.p__Firmicutes.c__Negativicutes.o__Veillonellales_Selenomonadales.f__Veillonellaceae.g__Megasphaera	3.675464746	H	3.2576162	0.001227
d__Bacteria.p__Firmicutes.c__Clostridia.o__Lachnospirales.f__Lachnospiraceae.g__Catonella	3.120054253			-
d__Bacteria.p__Bacteroidota.c__Bacteroidia.o__Bacteroidales.f__Paludibacteraceae.g__F0058	2.876106448			-
d__Bacteria.p__Firmicutes.c__Bacilli.o__Bacillales	1.651278014			-
d__Bacteria.p__Deferribacterota.c__Deferribacteres.o__Deferribacterales.f__Deferribacteraceae.g__Mucispirillum	2.440335489			-
d__Bacteria.p__Fusobacteriota.c__Fusobacteriia.o__Fusobacteriales	5.147631904			-
d__Bacteria.p__Deinococcota.c__Deinococci.o__Deinococcales.f__Deinococcaceae.g__Deinococcus	1.146128036			-
d__Bacteria.p__Firmicutes.c__Firmicutes_unclassified.o__Firmicutes_unclassified	1.440520915			-
d__Bacteria.p__Firmicutes.c__Clostridia.o__Clostridiales.f__Clostridiaceae	1.746356144			-
d__Bacteria.p__Firmicutes.c__Bacilli.o__Erysipelotrichales.f__Erysipelotrichaceae.g__Dubosiella	0.423245874			-
d__Bacteria.p__Cyanobacteria.c__Cyanobacteriia.o__Chloroplast	2.655493664			-
d__Bacteria.p__Proteobacteria.c__Gammaproteobacteria.o__Burkholderiales.f__Comamonadaceae.g__Brachymonas	0.235889007			-
d__Bacteria.p__Actinobacteriota.c__Actinobacteria.o__Actinobacteria_unclassified.f__Actinobacteria_unclassified.g__Actinobacteria_unclassified	3.104482434			-
d__Bacteria.p__Proteobacteria.c__Gammaproteobacteria.o__Enterobacterales.f__Enterobacteriaceae	2.483077621			-
d__Bacteria.p__Firmicutes.c__Clostridia.o__Lachnospirales.f__Defluviitaleaceae.g__Defluviitaleaceae_UCG_011	2.331268185	H	3.3105737	2.03E-14
d__Bacteria.p__Actinobacteriota.c__Actinobacteria.o__Pseudonocardiales.f__Pseudonocardiaceae	0			-
d__Bacteria.p__Firmicutes.c__Clostridia.o__Lachnospirales.f__Lachnospiraceae.g__Lachnospiraceae_UCG_006	1.740531862			-
d__Bacteria.p__Firmicutes.c__Clostridia.o__Clostridiales.f__Clostridiaceae.g__Candidatus_Arthromitus	0.872572463			-
d__Bacteria.p__Firmicutes.c__Clostridia	4.774478696	H	4.3321609	1.70E-16
d__Bacteria.p__Actinobacteriota.c__Actinobacteria.o__Micrococcales.f__Micrococcales_unclassified.g__Micrococcales_unclassified	2.000950837			-
d__Bacteria.p__Proteobacteria.c__Gammaproteobacteria.o__Burkholderiales.f__Neisseriaceae.g__Kingella	3.163365628			-
d__Bacteria.p__Bacteroidota.c__Bacteroidia.o__Bacteroidia_unclassified	0.534207478			-
d__Bacteria.p__Firmicutes.c__Clostridia.o__Oscillospirales.f__Oscillospiraceae.g__Oscillospiraceae_unclassified	2.478319081			-
d__Bacteria.p__Proteobacteria.c__Gammaproteobacteria.o__Xanthomonadales.f__Xanthomonadaceae	2.687186776			-
d__Bacteria.p__Bacteroidota.c__Bacteroidia.o__Bacteroidales.f__F082.g__F082	1.190523567			-
d__Bacteria.p__Verrucomicrobiota.c__Verrucomicrobiae.o__Verrucomicrobiales.f__Akkermansiaceae.g__Akkermansia	1.819308864			-
d__Bacteria.p__Patescibacteria.c__Saccharimonadia.o__Saccharimonadales.f__Saccharimonadaceae	4.121187808	H	3.743662	2.75E-16

d__Bacteria.p__Firmicutes.c__Clostridia.o__Lachnospirales.f__Lachnospiraceae.g___Eubacterium__xylanophilum_group	2.070064267			-
d__Bacteria.p__Actinobacteriota.c__Actinobacteria.o__Corynebacteriales.f__Corynebacteriaceae.g__Lawsonella	1.89030123			-
d__Bacteria.p__Firmicutes.c__Clostridia.o__Oscillospirales.f__Ruminococcaceae.g__Ruminococcus	2.594873781	H	3.0983552	1.42E-07
d__Bacteria.p__Actinobacteriota.c__Actinobacteria.o__Micrococcales	4.537188026			-
d__Bacteria.p__Desulfobacterota.c__Desulfovibrionia.o__Desulfovibrionales.f__Desulfovibrionaceae.g__Desulfovibrio	1.921314807			-
d__Bacteria.p__Bacteroidota.c__Bacteroidia.o__Bacteroidales.f__Prevotellaceae.g__Prevotellaceae_UCG_001	2.25544481			-
d__Bacteria.p__Firmicutes.c__Bacilli.o__Izemoplasmatales	1.43513877			-
d__Bacteria.p__Proteobacteria.c__Gammaproteobacteria.o__Burkholderiales.f__Burkholderiales_unclassified	2.960744369			-
d__Bacteria.p__Firmicutes.c__Negativicutes	5.10833028			-
d__Bacteria.p__Actinobacteriota.c__Actinobacteria.o__Frankiales.f__Frankiales_uncultured	0.890899844			-
d__Bacteria.p__Firmicutes.c__Clostridia.o__Monoglobales	2.059752694			-
d__Bacteria.p__Actinobacteriota.c__Actinobacteria.o__Micrococcales.f__Micrococcaceae	4.536311888			-
d__Bacteria.p__Patescibacteria.c__Saccharimonadia.o__Saccharimonadales.f__Saccharimonadales_unclassified.g__Saccharimonadales_unclassified	1.306169635			-
d__Bacteria.p__Firmicutes.c__Clostridia.o__Peptostreptococcales_Tissierellales.f__Peptostreptococcaceae.g__Romboutsia	1.466973481			-
d__Bacteria.p__Actinobacteriota.c__Actinobacteria.o__Actinobacteria_unclassified.f__Actinobacteria_unclassified	3.104482434			-
d__Bacteria.p__Firmicutes.c__Bacilli.o__Mycoplasmatales	2.727285578			-
d__Bacteria.p__Firmicutes.c__Clostridia.o__Clostridia_unclassified	0.02413368			-
d__Bacteria.p__Bacteroidota.c__Bacteroidia.o__Cytophagales.f__Hymenobacteraceae.g__Adhaeribacter	0.510927817			-
d__Bacteria.p__Cyanobacteria	2.655493664			-
d__Bacteria.p__Proteobacteria.c__Alphaproteobacteria.o__Rhizobiales.f__Rhizobiaceae	2.584408682			-
d__Bacteria.p__Firmicutes.c__Bacilli.o__RF39.f__RF39.g__RF39	2.411643753	H	3.0999493	8.61E-21
d__Bacteria.p__Firmicutes.c__Clostridia.o__Oscillospirales.f__Ruminococcaceae.g__Ruminococcaceae_unclassified	1.321628016			-
d__Bacteria.p__Bacteroidota.c__Bacteroidia.o__Bacteroidales.f__Prevotellaceae.g__Prevotellaceae_uncultured	1.911385924	H	3.2219784	5.02E-05
d__Bacteria.p__Firmicutes.c__Clostridia.o__Lachnospirales.f__Defluviitaleaceae	2.331268185	H	3.339986	2.03E-14
d__Bacteria.p__Proteobacteria.c__Alphaproteobacteria	3.172194128			-
d__Bacteria.p__Actinobacteriota.c__Actinobacteria.o__Corynebacteriales.f__Nocardiaceae	3.33472223	H	3.0529073	6.09E-10
d__Bacteria.p__Firmicutes.c__Bacilli.o__Bacillales.f__Planococcaceae	0.280383226			-
d__Bacteria.p__Firmicutes.c__Clostridia.o__Monoglobales.f__Monoglobaceae	2.059752694			-
d__Bacteria.p__Firmicutes.c__Clostridia.o__Lachnospirales.f__Lachnospiraceae.g__Lachnospiraceae_UCG_003	2.068901142			-
d__Bacteria.p__Proteobacteria.c__Gammaproteobacteria.o__Burkholderiales.f__Comamonadaceae.g__Comamonadaceae_unclassified	0.617299958			-
d__Bacteria.p__Bacteroidota.c__Bacteroidia.o__Flavobacteriales.f__Flavobacteriaceae.g__Flavobacterium	0.75858751			-
d__Bacteria.p__Bacteroidota.c__Bacteroidia.o__Bacteroidales.f__Bacteroidales_Bacteroidales_Incertae_Sedis	1.567558086			-
d__Bacteria.p__Firmicutes.c__Clostridia.o__Lachnospirales.f__Lachnospiraceae.g___Eubacterium__eligens_group	2.6214582	H	3.0567617	2.54E-09
d__Bacteria.p__Actinobacteriota.c__Actinobacteria.o__Frankiales.f__Frankiales_uncultured.g__Frankiales_uncultured	0.890899844			-
d__Bacteria.p__Bacteroidota.c__Bacteroidia.o__Bacteroidales.f__Rikenellaceae.g__Rikenellaceae_RC9_gut_group	2.548564891	H	3.0502377	2.00E-15
d__Bacteria.p__Bacteroidota.c__Bacteroidia.o__Bacteroidales.f__Muribaculaceae.g__Muribaculaceae	3.635925141	H	3.3510624	9.08E-18
d__Bacteria.p__Firmicutes.c__Clostridia.o__Lachnospirales.f__Lachnospiraceae.g__Tyzzerella	1.358750424			-
d__Bacteria.p__Proteobacteria.c__Alphaproteobacteria.o__Rhizobiales	3.005109962	CC	3.0661916	9.39E-09
d__Bacteria.p__Proteobacteria.c__Alphaproteobacteria.o__Rhizobiales.f__Xanthobacteraceae	1.567110426			-
d__Bacteria.p__Firmicutes.c__Bacilli.o__Bacillales.f__Bacillaceae	1.632384976			-
d__Bacteria.p__Campilobacterota	4.013369376	H	3.5601255	5.89E-16
d__Bacteria.p__Fusobacteriota.c__Fusobacteriia.o__Fusobacteriales.f__Leptotrichiaceae.g__Streptobacillus	2.227794917			-
d__Bacteria.p__Proteobacteria.c__Gammaproteobacteria.o__Burkholderiales.f__Rhodocyclaceae.g__Propionivibrio	0.36967325			-
d__Bacteria.p__Firmicutes.c__Bacilli.o__Bacillales.f__Bacillaceae.g__Bacillus	1.427903232			-
d__Bacteria.p__Verrucomicrobiota.c__Verrucomicrobiae.o__Verrucomicrobiales.f__Akkermansiaceae	1.819308864			-
d__Bacteria.p__Proteobacteria.c__Gammaproteobacteria.o__Pseudomonadales.f__Moraxellaceae.g__Faucicola	0.770852012			-
d__Bacteria.p__Firmicutes.c__Clostridia.o__Lachnospirales.f__Lachnospiraceae.g__Lachnospiraceae_uncultured	3.595337938	H	3.274093	8.26E-12
d__Bacteria.p__Firmicutes.c__Bacilli.o__Lactobacillales.f__Streptococcaceae.g__Lactococcus	1.401236377			-
d__Bacteria.p__Planctomycetota.c__Planctomycetes.o__Pirellulales.f__Pirellulaceae	0.516629796			-
d__Bacteria.p__Actinobacteriota.c__Actinobacteria.o__Bifidobacteriales.f__Bifidobacteriaceae.g__Scardovia	2.839357496			-
d__Bacteria.p__Actinobacteriota.c__Actinobacteria.o__Micrococcales.f__Dermabacteraceae	0.15490196			-
d__Bacteria.p__Actinobacteriota.c__Actinobacteria.o__Corynebacteriales	3.701339122	H	3.235303	2.74E-13
d__Bacteria.p__Firmicutes.c__Bacilli.o__Paenibacillales.f__Paenibacillaceae	0.750518962			-
d__Bacteria.p__Proteobacteria.c__Gammaproteobacteria.o__Burkholderiales.f__Burkholderiaceae	4.749665657	CCR	4.4360762	1.95E-10
d__Bacteria.p__Firmicutes.c__Bacilli.o__Paenibacillales.f__Paenibacillaceae.g__Paenibacillus	0.750518962			-
d__Bacteria.p__Firmicutes.c__Clostridia.o__Peptostreptococcales_Tissierellales.f__Peptostreptococcaceae.g__Filifactor	3.222179308	H	3.0442316	1.64E-17
d__Bacteria.p__Firmicutes.c__Clostridia.o__Lachnospirales.f__Lachnospiraceae.g__Lachnospiraceae_NK4A136_group	3.247866345	H	3.0999406	1.30E-14

d__Bacteria.p__Bacteroidota.c__Bacteroidia.o__Cytophagales.f__Hymenobacteraceae.g__Hymenobacter	0.395451208			-
d__Bacteria.p__Proteobacteria.c__Alphaproteobacteria.o__Rhizobiales.f__Beijerinckiaceae.g__Bosea	0.909731833			-
d__Bacteria.p__Bacteroidota.c__Bacteroidia.o__Sphingobacteriales.f__Lentimicrobiaceae.g__Lentimicrobium	2.522723612	H	3.2807814	6.86E-20
d__Bacteria.p__Firmicutes.c__Clostridia.o__Peptostreptococcales_Tissierellales.f__Peptostreptococcales_Tissierellales.g__Parvimonas	3.324892156	H	3.0337185	3.92E-20
d__Bacteria.p__Verrucomicrobiota.c__Lentisphaeria	0.525969822			-
d__Bacteria.p__Elusimicrobiota.c__Elusimicrobia.o__Lineage_IV.f__Lineage_IV	0.299476168			-
d__Bacteria.p__Proteobacteria.c__Gammaproteobacteria.o__Pasteurellales.f__Pasteurellaceae.g__Actinobacillus	3.943196569	H	3.6105848	7.04E-21
d__Bacteria.p__Actinobacteriota.c__Actinobacteria.o__Micrococcales.f__Micrococcaceae.g__Nesterenkonia	1.091401931			-
d__Bacteria.p__Firmicutes.c__Clostridia.o__Lachnospirales.f__Lachnospiraceae.g___Eubacterium__ruminantium_group	2.808288157	H	3.0410501	1.19E-13
d__Bacteria.p__Proteobacteria.c__Gammaproteobacteria.o__Oceanospirillales.f__Halomonadaceae	3.951764536	CC	3.6185296	1.56E-10
d__Bacteria.p__Bacteroidota.c__Bacteroidia.o__Bacteroidales.f__Bacteroidaceae	3.802908536	H	3.4875914	3.81E-20
d__Bacteria.p__Elusimicrobiota.c__Endomicrobia.o__Endomicrobiales.f__Endomicrobiaceae	0.525044807			-
d__Bacteria.p__Proteobacteria.c__Alphaproteobacteria.o__Rhizobiales.f__Rhizobiaceae.g__Aquamicrobium	0.791815575			-
d__Bacteria.p__Deferribacterota.c__Deferribacteres.o__Deferribacterales.f__Deferribacteraceae	2.440335489			-
d__Bacteria.p__Bacteroidota.c__Bacteroidia.o__Bacteroidales.f__Muribaculaceae.g__Muribaculum	1.440346743			-
d__Bacteria.p__Proteobacteria.c__Gammaproteobacteria.o__Pseudomonadales.f__Pseudomonadaceae.g__Pseudomonas	3.716747211	H	3.4197807	7.11E-29
d__Bacteria.p__Bacteroidota.c__Bacteroidia.o__Sphingobacteriales.f__Lentimicrobiaceae	2.522723612	H	3.1331906	6.86E-20
d__Bacteria.p__Proteobacteria.c__Alphaproteobacteria.o__Rhodospirillales.f__Rhodospirillales_uncultured	2.241848759			-
d__Bacteria.p__Proteobacteria.c__Gammaproteobacteria.o__Pseudomonadales.f__Moraxellaceae.g__Enhydrobacter	1.190931691			-
d__Bacteria.p__Firmicutes.c__Clostridia.o__Lachnospirales.f__Lachnospiraceae.g__Lachnospiraceae_unclassified	3.456591582	H	3.1793748	8.84E-09
d__Bacteria.p__Proteobacteria.c__Alphaproteobacteria.o__Rhodobacterales.f__Rhodobacteraceae.g__Paracoccus	1.141673694			-
d__Bacteria.p__Bacteroidota.c__Bacteroidia.o__Bacteroidales.f__p_2534_18B5_gut_group	1.108178297			-
d__Bacteria.p__Proteobacteria.c__Gammaproteobacteria.o__Burkholderiales.f__Chromobacteriaceae.g__Vogesella	0			-
d__Bacteria.p__Firmicutes.c__Clostridia.o__Lachnospirales.f__Lachnospiraceae.g__ASF356	1.70616893			-
d__Bacteria.p__Patescibacteria.c__Gracilibacteria.o__Gracilibacteria.f__Gracilibacteria.g__Gracilibacteria	2.73900693			-
d__Bacteria.p__Firmicutes.c__Bacilli.o__Bacillales.f__Bacillaceae.g__Anoxybacillus	0			-
d__Bacteria.p__Fusobacteriota.c__Fusobacteriia.o__Fusobacteriales.f__Leptotrichiaceae.g__Leptotrichia	5.038733417	CC	4.5302532	0.002275
d__Bacteria.p__Bacteroidota.c__Bacteroidia.o__Sphingobacteriales	2.538412136	H	3.4726561	4.39E-19
d__Bacteria.p__Proteobacteria.c__Gammaproteobacteria.o__Oceanospirillales	3.951764536	CC	3.6185269	1.56E-10
d__Bacteria.p__Firmicutes.c__Clostridia.o__Oscillospirales.f__Butyricicoccaceae	1.835418769			-
d__Bacteria.p__Proteobacteria.c__Gammaproteobacteria.o__Alteromonadales	1.766625268			-
d__Bacteria.p__Firmicutes.c__Bacilli.o__Lactobacillales.f__Lactobacillaceae.g__Lactobacillus	2.912157504			-
d__Bacteria.p__Firmicutes.c__Clostridia.o__Oscillospirales.f__Ruminococcaceae.g__Ruminococcaceae	0.392948063			-
d__Bacteria.p__Proteobacteria.c__Alphaproteobacteria.o__Rickettsiales.f__Mitochondria.g__Mitochondria	1.871556943			-
d__Bacteria.p__Firmicutes.c__Bacilli.o__Lactobacillales.f__Carnobacteriaceae.g__Atopostipes	0.784820995			-
d__Bacteria.p__Firmicutes.c__Clostridia.o__Peptostreptococcales_Tissierellales.f__Anaerovoracaceae.g__Amnipila	2.588207659			-
d__Bacteria.p__Fusobacteriota.c__Fusobacteriia	5.147631904			-
d__Bacteria.p__Proteobacteria.c__Alphaproteobacteria.o__Rhizobiales.f__Hyphomicrobiaceae.g__Pedomicrobium	0.6734159			-
d__Bacteria.p__Cyanobacteria.c__Vampirivibrionia.o__Gastranaerophilales.f__Gastranaerophilales	1.669715255			-
d__Bacteria.p__Firmicutes.c__Negativicutes.o__Veillonellales_Selenomonadales.f__Veillonellaceae.g__Dialister	3.104331003			-
d__Bacteria.p__Proteobacteria.c__Gammaproteobacteria.o__Burkholderiales.f__Burkholderiales_unclassified.g__Burkholderiales_unclassified	2.960744369			-
d__Bacteria.p__Patescibacteria.c__Gracilibacteria.o__JGI_0000069_P22.f__JGI_0000069_P22.g__JGI_0000069_P22	1.761175813			-
d__Bacteria.p__Proteobacteria.c__Alphaproteobacteria.o__Rhodobacterales	1.246920431			-
d__Bacteria.p__Firmicutes.c__Negativicutes.o__Acidaminococcales.f__Acidaminococcaceae	2.6064044	H	3.0378796	2.82E-09
d__Bacteria.p__Firmicutes.c__Negativicutes.o__Veillonellales_Selenomonadales.f__Veillonellaceae	5.093579503			-
d__Bacteria.p__Cyanobacteria.c__Cyanobacteriia	2.655493664			-
d__Bacteria.p__Proteobacteria.c__Alphaproteobacteria.o__Rhizobiales.f__Xanthobacteraceae.g__Xanthobacteraceae_unclassified	1.567110426			-
d__Bacteria.p__Firmicutes.c__Clostridia.o__Peptostreptococcales_Tissierellales.f__Peptostreptococcaceae.g__Peptostreptococcaceae	0.94094317			-
d__Bacteria.p__Firmicutes.c__Bacilli.o__Bacillales.f__Planococcaceae.g__Lysinibacillus	0.280383226			-
d__Bacteria.p__Firmicutes.c__Negativicutes.o__Veillonellales_Selenomonadales.f__Veillonellaceae.g__Allisonella	1.455606113			-
d__Bacteria.p__Firmicutes.c__Bacilli.o__Lactobacillales.f__Lactobacillaceae	2.912506734			-
d__Bacteria.p__Firmicutes.c__Clostridia.o__Clostridia_UCG_014.f__Clostridia_UCG_014.g__Clostridia_UCG_014	3.571943298	H	3.2027096	4.61E-18
d__Bacteria.p__Firmicutes.c__Clostridia.o__Lachnospirales.f__Lachnospiraceae	4.550720391	H	4.0927755	6.10E-13
d__Bacteria.p__Proteobacteria.c__Gammaproteobacteria	5.493755578	CCR	4.8473922	5.76E-06
d__Bacteria.p__Proteobacteria.c__Gammaproteobacteria.o__Burkholderiales.f__Nitrosomonadaceae	0			-
d__Bacteria.p__Chloroflexi.c__Anaerolineae	0.995635195			-

d__Bacteria.p__Cyanobacteria.c__Cyanobacteriia.o__Chloroplast.f__Chloroplast.g__Chloroplast	2.655493664			-
d__Bacteria.p__Deferribacterota.c__Deferribacteres.o__Deferribacterales	2.440335489			-

Supplementary table S21. The corresponding LDA value and p value of microbial community gene function for samples in the discovery cohort				
Biomaker_names	Logarithm value	Groups	LDA_value	P_value
L1_Metabolism.L2_Glycan_biosynthesis_and_metabolism.L3_Glycosylphosphatidylinositol_GPI_anchor_biosynthesis	0.10374348			-
L1_Metabolism.L2_Lipid_metabolism.L3_Sphingolipid_metabolism	3.43633711			-
L1_Metabolism.L2_Metabolism_of_cofactors_and_vitamins.L3_Riboflavin_metabolism	4.04641764			-
L1_Metabolism.L2_Nucleotide_metabolism.L3_Purine_metabolism	4.00409323			-
L1_Metabolism.L2_Amino_acid_metabolism.L3_Cysteine_and_methionine_metabolism	4.17184063			-
L1_Metabolism.L2_Lipid_metabolism.L3_Fatty_acid_degradation	3.59777962			-
L1_Environmental_Information_Processing.L2_Membrane_transport.L3_Bacterial_secretion_system	4.05244459			-
L1_Metabolism.L2_Metabolism_of_cofactors_and_vitamins.L3_Porphyrin_and_chlorophyll_metabolism	3.92652857			-
L1_Metabolism.L2_Biosynthesis_of_other_secondary_metabolites.L3_Penicillin_and_cephalosporin_biosynthesis	2.98786639			-
L1_Metabolism.L2_Xenobiotics_biodegradation_and_metabolism.L3_Drug_metabolism_other_enzymes	4.11114927	H	3.1958548	0.00088
L1_Human_Diseases.L2_Neurodegenerative_diseases.L3_Alzheimer_s_disease	1.54985258			-
L1_Cellular_Processes.L2_Cell_growth_and_death.L3_Apoptosis	2.75330921			-
L1_Metabolism.L2_Metabolism_of_terpenoids_and_polyketides.L3_Biosynthesis_of_siderophore_group_nonribosomal_peptides	2.97814764			-
L1_Metabolism.L2_Amino_acid_metabolism.L3_Histidine_metabolism	3.99309841			-
L1_Metabolism.L2_Amino_acid_metabolism.L3_Valine_leucine_and_isoleucine_degradation	3.65318755			-
L1_Metabolism.L2_Metabolism_of_terpenoids_and_polyketides.L3_Limonene_and_pinene_degradation	2.9613178			-
L1_Metabolism.L2_Amino_acid_metabolism.L3_Glycine_serine_and_threonine_metabolism	4.06085294			-
L1_Metabolism.L2_Metabolism_of_terpenoids_and_polyketides.L3_Terpenoid_backbone_biosynthesis	4.15337955			-
L1_Metabolism.L2_Glycan_biosynthesis_and_metabolism.L3_N_Glycan_biosynthesis	2.85019225			-
L1_Metabolism.L2_Carbohydrate_metabolism.L3_Propanoate_metabolism	3.87401176			-
L1_Metabolism.L2_Lipid_metabolism.L3_Biosynthesis_of_unsaturated_fatty_acids	3.61816627			-
L1_Metabolism.L2_Biosynthesis_of_other_secondary_metabolites.L3_Betalain_biosynthesis	1.52360667			-
L1_Genetic_Information_Processing.L2_Translation.L3_Aminoacyl_tRNA_biosynthesis	4.28392206			-
L1_Cellular_Processes.L2_Cell_growth_and_death.L3_Cell_cycle_Caulobacter	4.22504993			-
L1_Metabolism.L2_Xenobiotics_biodegradation_and_metabolism.L3_Xylene_degradation	2.41068797			-
L1_Metabolism.L2_Xenobiotics_biodegradation_and_metabolism.L3_Chlorocyclohexane_and_chlorobenzene_degradation	2.87822892			-
L1_Metabolism.L2_Metabolism_of_cofactors_and_vitamins.L3_Nicotinate_and_nicotinamide_metabolism	4.09836819			-
L1_Metabolism.L2_Metabolism_of_other_amino_acids.L3_D_Alanine_metabolism	4.32418564	CC	3.113352	9.77E-13
L1_Metabolism.L2_Xenobiotics_biodegradation_and_metabolism.L3_Nitrotoluene_degradation	3.29753609			-
L1_Cellular_Processes.L2_Transport_and_catabolism.L3_Lysosome	1.13749196			-
L1_Metabolism.L2_Lipid_metabolism.L3_Steroid_hormone_biosynthesis	2.30592472			-
L1_Metabolism.L2_Xenobiotics_biodegradation_and_metabolism.L3_Caprolactam_degradation	2.56950466			-
L1_Metabolism.L2_Metabolism_of_terpenoids_and_polyketides.L3_Biosynthesis_of_type_II_polyketide_products	0			-
L1_Metabolism.L2_Metabolism_of_other_amino_acids.L3_Taurine_and_hypotaurine_metabolism	3.89238978			-
L1_Cellular_Processes.L2_Cellular_community_eukaryotes.L3_Focal_adhesion	0.03960331			-
L1_Metabolism.L2_Metabolism_of_terpenoids_and_polyketides.L3_Zeatin_biosynthesis	3.9095623			-
L1_Genetic_Information_Processing.L2_Replication_and_repair.L3_DNA_replication	4.15243699			-
L1_Metabolism.L2_Biosynthesis_of_other_secondary_metabolites.L3_Flavonoid_biosynthesis	2.54988711			-
L1_Environmental_Information_Processing.L2_Signal_transduction.L3_Plant_hormone_signal_transduction	0			-
L1_Genetic_Information_Processing.L2_Translation.L3_Ribosome_biogenesis_in_eukaryotes	2.91756701			-
L1_Environmental_Information_Processing.L2_Signal_transduction.L3_Two_component_system	3.49088634			-
L1_Human_Diseases.L2_Infectious_diseases__Parasitic.L3_Toxoplasmosis	0.64595511			-
L1_Metabolism.L2_Nucleotide_metabolism.L3_Pyrimidine_metabolism	4.12319044			-
L1_Metabolism.L2_Amino_acid_metabolism.L3_Arginine_and_proline_metabolism	3.80765184			-
L1_Metabolism.L2_Carbohydrate_metabolism.L3_Pyruvate_metabolism	4.12800448			-
L1_Metabolism.L2_Metabolism_of_cofactors_and_vitamins.L3_Folate_biosynthesis	4.19321737			-
L1_Metabolism.L2_Amino_acid_metabolism.L3_Valine_leucine_and_isoleucine_biosynthesis	4.35253881	CCR	3.0062578	7.69E-15
L1_Metabolism.L2_Xenobiotics_biodegradation_and_metabolism.L3_Fluorobenzoate_degradation	2.34837859			-
L1_Cellular_Processes.L2_Transport_and_catabolism.L3_Endocytosis	0			-
L1_Metabolism.L2_Xenobiotics_biodegradation_and_metabolism.L3_Dioxin_degradation	3.24004262			-
L1_Metabolism.L2_Amino_acid_metabolism.L3_Tyrosine_metabolism	3.52173579			-
L1_Human_Diseases.L2_Infectious_diseases__Bacterial.L3_Vibrio_cholerae_infection	0.89894446			-
L1_Human_Diseases.L2_Infectious_diseases__Bacterial.L3_Bacterial_invasion_of_epithelial_cells	1.64428818			-
L1_Metabolism.L2_Xenobiotics_biodegradation_and_metabolism.L3_Styrene_degradation	3.25790237			-
L1_Metabolism.L2_Metabolism_of_terpenoids_and_polyketides.L3_Geraniol_degradation	3.21600764			-

L1_Organismal_Systems.L2_Endocrine_system.L3_Insulin_signaling_pathway	2.99991042			-
L1_Metabolism.L2_Xenobiotics_biodegradation_and_metabolism.L3_Metabolism_of_xenobiotics_by_cytochrome_P450	1.79635426			-
L1_Metabolism.L2_Glycan_biosynthesis_and_metabolism.L3_Lipopolysaccharide_biosynthesis	4.2585507			-
L1_Metabolism.L2_Biosynthesis_of_other_secondary_metabolites.L3_Tropane_piperidine_and_pyridine_alkaloid_biosynthesis	3.6706811			-
L1_Metabolism.L2_Metabolism_of_cofactors_and_vitamins.L3_One_carbon_pool_by_folate	4.30554183			-
L1_Metabolism.L2_Energy_metabolism.L3_Methane_metabolism	3.64916371			-
L1_Genetic_Information_Processing.L2_Replication_and_repair.L3_Homologous_recombination	4.23478742			-
L1_Organismal_Systems.L2_Immune_system.L3_NOD_like_receptor_signaling_pathway	2.8573291			-
L1_Metabolism.L2_Metabolism_of_other_amino_acids.L3_D_Arginine_and_D_ornithine_metabolism	3.59985331			-
L1_Genetic_Information_Processing.L2_Replication_and_repair.L3_Base_excision_repair	4.05183965			-
L1_Human_Diseases.L2_Immune_diseases.L3_Systemic_lupus_erythematosus	2.27866085			-
L1_Metabolism.L2_Metabolism_of_terpenoids_and_polyketides.L3_Sesquiterpenoid_and_triterpenoid_biosynthesis	0			-
L1_Metabolism.L2_Carbohydrate_metabolism.L3_Pentose_phosphate_pathway	4.21687123			-
L1_Metabolism.L2_Biosynthesis_of_other_secondary_metabolites.L3_Isoflavonoid_biosynthesis	0			-
L1_Metabolism.L2_Amino_acid_metabolism.L3_Phenylalanine_metabolism	3.46175527			-
L1_Metabolism.L2_Energy_metabolism.L3_Photosynthesis_antenna_proteins	0.85220535			-
L1_Metabolism.L2_Metabolism_of_cofactors_and_vitamins.L3_Biotin_metabolism	4.18552056			-
L1_Genetic_Information_Processing.L2_Folding_sorting_and_degradation.L3_Protein_processing_in_endoplasmic_reticulum	2.48612904			-
L1_Genetic_Information_Processing.L2_Folding_sorting_and_degradation.L3_Proteasome	1.92768981			-
L1_Metabolism.L2_Amino_acid_metabolism.L3_Phenylalanine_tyrosine_and_tryptophan_biosynthesis	4.12522668			-
L1_Metabolism.L2_Carbohydrate_metabolism.L3_C5_Branched_dibasic_acid_metabolism	4.27046549	CCR	3.1586659	7.44E-08
L1_Metabolism.L2_Metabolism_of_terpenoids_and_polyketides.L3_Carotenoid_biosynthesis	2.57711223			-
L1_Metabolism.L2_Carbohydrate_metabolism.L3_Amino_sugar_and_nucleotide_sugar_metabolism	4.03058418			-
L1_Organismal_Systems.L2_Digestive_system.L3_Protein_digestion_and_absorption	2.83601349			-
L1_Metabolism.L2_Energy_metabolism.L3_Sulfur_metabolism	4.05218498			-
L1_Genetic_Information_Processing.L2_Translation.L3_RNA_transport	2.70787929			-
L1_Metabolism.L2_Carbohydrate_metabolism.L3_Butanoate_metabolism	3.87402066			-
L1_Metabolism.L2_Xenobiotics_biodegradation_and_metabolism.L3_Aminobenzoate_degradation	3.21000319			-
L1_Metabolism.L2_Energy_metabolism.L3_Nitrogen_metabolism	3.84934203			-
L1_Metabolism.L2_Metabolism_of_other_amino_acids.L3_Glutathione_metabolism	3.97241004			-
L1_Metabolism.L2_Energy_metabolism.L3_Carbon_fixation_in_photosynthetic_organisms	4.23715227			-
L1_Human_Diseases.L2_Infectious_diseases__Bacterial.L3_Staphylococcus_aureus_infection	2.92915503			-
L1_Metabolism.L2_Metabolism_of_cofactors_and_vitamins.L3_Vitamin_B6_metabolism	4.09218455			-
L1_Metabolism.L2_Xenobiotics_biodegradation_and_metabolism.L3_Bisphenol_degradation	2.94702404			-
L1_Metabolism.L2_Carbohydrate_metabolism.L3_Inositol_phosphate_metabolism	3.35399866			-
L1_Metabolism.L2_Metabolism_of_other_amino_acids.L3_D_Glutamine_and_D_glutamate_metabolism	4.40068551			-
L1_Cellular_Processes.L2_Cell_growth_and_death.L3_Meiosis_yeast	1.40514191			-
L1_Environmental_Information_Processing.L2_Membrane_transport.L3_ABC_transporters	3.92983403			-
L1_Cellular_Processes.L2_Cellular_community_prokaryotes.L3_Biofilm_formation_Vibrio_cholerae	3.29048912			-
L1_Metabolism.L2_Lipid_metabolism.L3_Linoleic_acid_metabolism	3.25278946			-
L1_Metabolism.L2_Glycan_biosynthesis_and_metabolism.L3_Peptidoglycan_biosynthesis	4.31597012			-
L1_Genetic_Information_Processing.L2_Folding_sorting_and_degradation.L3_Protein_export	4.22485711			-
L1_Metabolism.L2_Glycan_biosynthesis_and_metabolism.L3_Other_types_of_O_glycan_biosynthesis	1.23365699			-
L1_Metabolism.L2_Carbohydrate_metabolism.L3_Galactose_metabolism	3.9405277			-
L1_Metabolism.L2_Xenobiotics_biodegradation_and_metabolism.L3_Toluene_degradation	3.3908055			-
L1_Genetic_Information_Processing.L2_Folding_sorting_and_degradation.L3_Sulfur_relay_system	4.04340219			-
L1_Metabolism.L2_Metabolism_of_terpenoids_and_polyketides.L3_Biosynthesis_of_vancomycin_group_antibiotics	4.3545816	H	3.2759562	7.57E-11
L1_Human_Diseases.L2_Cardiovascular_diseases.L3_Hypertrophic_cardiomyopathy_HCM	1.00721888			-
L1_Metabolism.L2_Glycan_biosynthesis_and_metabolism.L3_Glycosphingolipid_biosynthesis_lacto_and_neolacto_series	0			-
L1_Genetic_Information_Processing.L2_Transcription.L3_RNA_polymerase	3.92306834			-
L1_Metabolism.L2_Amino_acid_metabolism.L3_Alanine_aspartate_and_glutamate_metabolism	4.19179557			-
L1_Metabolism.L2_Xenobiotics_biodegradation_and_metabolism.L3_Benzoate_degradation	3.23274613			-
L1_Human_Diseases.L2_Infectious_diseases__Bacterial.L3_Shigellosis	0			-
L1_Metabolism.L2_Carbohydrate_metabolism.L3_Glycolysis_Gluconeogenesis	4.08891763			-
L1_Metabolism.L2_Amino_acid_metabolism.L3_Lysine_biosynthesis	4.21086544			-
L1_Metabolism.L2_Lipid_metabolism.L3_Synthesis_and_degradation_of_ketone_bodies	3.71599556			-
L1_Human_Diseases.L2_Infectious_diseases__Parasitic.L3_Chagas_disease_American_trypanosomiasis	1.43437122			-
L1_Metabolism.L2_Energy_metabolism.L3_Oxidative_phosphorylation	3.73935381			-

L1_Metabolism.L2_Metabolism_of_cofactors_and_vitamins.L3_Ubiquinone_and_other_terpenoid_quinone_biosynthesis	3.78556063			-
L1_Organismal_Systems.L2_Endocrine_system.L3_Renin_angiotensin_system	0			-
L1_Metabolism.L2_Energy_metabolism.L3_Carbon_fixation_pathways_in_prokaryotes	4.08214217			-
L1_Metabolism.L2_Xenobiotics_biodegradation_and_metabolism.L3_Ethylbenzene_degradation	3.14691566			-
L1_Human_Diseases.L2_Infectious_diseases__Parasitic.L3_African_trypanosomiasis	1.93396169			-
L1_Metabolism.L2_Metabolism_of_other_amino_acids.L3_beta_Alanine_metabolism	3.71825834	H	3.1440111	1.82E-15
L1_Metabolism.L2_Lipid_metabolism.L3_Glycerophospholipid_metabolism	3.85058109			-
L1_Metabolism.L2_Carbohydrate_metabolism.L3_Fructose_and_mannose_metabolism	3.93648676			-
L1_Metabolism.L2_Lipid_metabolism.L3_Secondary_bile_acid_biosynthesis	3.06433509			-
L1_Metabolism.L2_Metabolism_of_other_amino_acids.L3_Cyanoamino_acid_metabolism	3.77046249	H	3.2004351	2.39E-15
L1_Genetic_Information_Processing.L2_Transcription.L3_Spliceosome	1.60489333			-
L1_Metabolism.L2_Lipid_metabolism.L3_Glycerolipid_metabolism	3.66876723			-
L1_Metabolism.L2_Metabolism_of_other_amino_acids.L3_Phosphonate_and_phosphinate_metabolism	3.35635647			-
L1_Genetic_Information_Processing.L2_Replication_and_repair.L3_Non_homologous_end_joining	1.68489163			-
L1_Metabolism.L2_Lipid_metabolism.L3_Primary_bile_acid_biosynthesis	2.17833884			-
L1_Metabolism.L2_Biosynthesis_of_other_secondary_metabolites.L3_Streptomycin_biosynthesis	4.19839342	H	3.0919045	6.53E-06
L1_Metabolism.L2_Carbohydrate_metabolism.L3_Glyoxylate_and_dicarboxylate_metabolism	3.79477342			-
L1_Organismal_Systems.L2_Excretory_system.L3_Vasopressin_regulated_water_reabsorption	0			-
L1_Cellular_Processes.L2_Cell_motility.L3_Flagellar_assembly	3.34649629			-
L1_Metabolism.L2_Xenobiotics_biodegradation_and_metabolism.L3_Naphthalene_degradation	2.75529622			-
L1_Metabolism.L2_Carbohydrate_metabolism.L3_Ascorbate_and_aldarate_metabolism	3.4572582			-
L1_Metabolism.L2_Xenobiotics_biodegradation_and_metabolism.L3_Chloroalkane_and_chloroalkene_degradation	3.40488094			-
L1_Cellular_Processes.L2_Cell_motility.L3_Bacterial_chemotaxis	3.62349179			-
L1_Environmental_Information_Processing.L2_Membrane_transport.L3_Phosphotransferase_system_PTS	3.81228899	CCR	3.1453603	5.84E-13
L1_Metabolism.L2_Carbohydrate_metabolism.L3_Citrate_cycle_TCA_cycle	4.07651249			-
L1_Metabolism.L2_Metabolism_of_cofactors_and_vitamins.L3_Pantothenate_and_CoA_biosynthesis	4.26367575			-
L1_Metabolism.L2_Lipid_metabolism.L3_Fatty_acid_biosynthesis	4.33736666	CCR	3.2539548	2.85E-06
L1_Cellular_Processes.L2_Transport_and_catabolism.L3_Peroxisome	3.42748699			-
L1_Genetic_Information_Processing.L2_Replication_and_repair.L3_Nucleotide_excision_repair	3.94420532			-
L1_Metabolism.L2_Lipid_metabolism.L3_Steroid_biosynthesis	1.43445349			-
L1_Metabolism.L2_Glycan_biosynthesis_and_metabolism.L3_Glycosaminoglycan_degradation	3.60320857	H	3.0456583	7.67E-12
L1_Metabolism.L2_Amino_acid_metabolism.L3_Tryptophan_metabolism	3.3815422			-
L1_Metabolism.L2_Amino_acid_metabolism.L3_Lysine_degradation	3.31862028			-
L1_Metabolism.L2_Metabolism_of_other_amino_acids.L3_Selenocompound_metabolism	4.10229403			-
L1_Human_Diseases.L2_Infectious_diseases__Bacterial.L3_Epithelial_cell_signaling_in_Helicobacter_pylori_infection	3.18639429			-
L1_Metabolism.L2_Xenobiotics_biodegradation_and_metabolism.L3_Polycyclic_aromatic_hydrocarbon_degradation	1.65557526			-
L1_Metabolism.L2_Metabolism_of_cofactors_and_vitamins.L3_Lipoic_acid_metabolism	4.12209509			-
L1_Metabolism.L2_Carbohydrate_metabolism.L3_Starch_and_sucrose_metabolism	3.90887777			-
L1_Genetic_Information_Processing.L2_Translation.L3_Ribosome	4.26029231			-
L1_Genetic_Information_Processing.L2_Folding_sorting_and_degradation.L3_RNA_degradation	3.82641202			-
L1_Metabolism.L2_Metabolism_of_cofactors_and_vitamins.L3_Retinol_metabolism	2.99364274			-
L1_Genetic_Information_Processing.L2_Replication_and_repair.L3_Mismatch_repair	4.28080036			-
L1_Metabolism.L2_Xenobiotics_biodegradation_and_metabolism.L3_Atrazine_degradation	2.45615916			-
L1_Organismal_Systems.L2_Environmental_adaptation.L3_Plant_pathogen_interaction	3.26167777			-
L1_Metabolism.L2_Metabolism_of_terpenoids_and_polyketides.L3_Biosynthesis_of_ansamycins	4.63333255	CC	3.4586554	0.00078
L1_Metabolism.L2_Metabolism_of_terpenoids_and_polyketides.L3_Biosynthesis_of_type_II_polyketide_backbone	0			-
L1_Metabolism.L2_Energy_metabolism.L3_Photosynthesis	3.38610868			-
L1_Metabolism.L2_Glycan_biosynthesis_and_metabolism.L3_Various_types_of_N_glycan_biosynthesis	0			-
L1_Metabolism.L2_Glycan_biosynthesis_and_metabolism.L3_Other_glycan_degradation	4.03870185	H	3.3735842	1.82E-08
L1_Metabolism.L2_Metabolism_of_cofactors_and_vitamins.L3_Thiamine_metabolism	4.18168003			-
L1_Human_Diseases.L2_Infectious_diseases__Parasitic.L3_Amoebiasis	1.98211611			-
L1_Metabolism.L2_Carbohydrate_metabolism.L3_Pentose_and_glucuronate_interconversions	3.53497604			-

Supplementary table S22. The p value between 44 oropharyngeal OTUs and 11 clinical indicators of CC (n=48) and H (n=94) obtained by spearman correlation analysis.										
x	y	grp	subx	suby	Genus	rho	p	lcol	lwd	rank
-0.5	44	RBC	-10	41	OTU599 (Burkholderiales_unclassified)	0.046946656	0.579034644	0	#FF8E8A	0
0.5	43	RBC	-10	41	OTU744 (Leptotrichia)	0.01441396	0.864811289	0	#FF8E8A	0
1.5	42	RBC	-10	41	OTU658 (P5D1-392)	0.029156037	0.730517916	0	#FF8E8A	0
2.5	41	RBC	-10	41	OTU796 (Rothia)	0.038621488	0.648152223	0	#FF8E8A	0
3.5	40	RBC	-10	41	OTU748 (Leptotrichia)	0.016238997	0.847889267	0	#FF8E8A	0
4.5	39	RBC	-10	41	OTU762 (Streptococcus)	-0.026679405	0.752634172	0	#00CCCC	0
5.5	38	RBC	-10	41	OTU34 (Halomonas)	-0.193708834	0.020897692	1	#00CCCC	1
6.5	37	RBC	-10	41	OTU1 (Streptococcus)	-0.123581942	0.142851229	0	#00CCCC	1
7.5	36	RBC	-10	41	OTU126 ([Eubacterium]_ruminantium_group)	0.026298906	0.756050401	0	#FF8E8A	0
8.5	35	RBC	-10	41	OTU82 (Faecalibacterium)	-0.056596018	0.503498719	0	#00CCCC	0
9.5	34	RBC	-10	41	OTU69 (Fusicatenibacter)	-0.035455457	0.675290721	0	#00CCCC	0
10.5	33	RBC	-10	41	OTU85 (Prevotella)	-0.021464004	0.79984969	0	#00CCCC	0
11.5	32	RBC	-10	41	OTU252 (Prevotella)	-0.015663121	0.853221779	0	#00CCCC	0
12.5	31	RBC	-10	41	OTU129 (Acinetobacter)	-0.012789843	0.879922347	0	#00CCCC	0
13.5	30	RBC	-10	41	OTU31 (Pseudomonas)	0.066096153	0.434496653	0	#FF8E8A	0
14.5	29	RBC	-10	41	OTU15 (Campylobacter)	0.002243539	0.978859666	0	#FF8E8A	0
15.5	28	RBC	-10	41	OTU87 (Corynebacterium)	-0.020918324	0.804835156	0	#00CCCC	0
16.5	27	RBC	-10	41	OTU114 (Prevotella)	0.140170214	0.096156007	0	#FF8E8A	1
17.5	26	RBC	-10	41	OTU172 (Prevotella)	0.110161027	0.191861072	0	#FF8E8A	1
18.5	25	RBC	-10	41	OTU119 (Prevotella)	0.08643828	0.306382223	0	#FF8E8A	0
19.5	24	RBC	-10	41	OTU5 (Haemophilus)	0.083611199	0.322531272	0	#FF8E8A	0
20.5	23	RBC	-10	41	OTU171 (Aggregatibacter)	0.05987883	0.479027609	0	#FF8E8A	0
21.5	22	RBC	-10	41	OTU39 (Haemophilus)	0.048990233	0.562606768	0	#FF8E8A	0
22.5	21	RBC	-10	41	OTU20 (Actinobacillus)	0.023513247	0.781200782	0	#FF8E8A	0
23.5	20	RBC	-10	41	OTU10 (Alloprevotella)	0.053646722	0.526027981	0	#FF8E8A	0
24.5	19	RBC	-10	41	OTU26 (Prevotella)	0.091140684	0.280715215	0	#FF8E8A	0
25.5	18	RBC	-10	41	OTU71 (Alloprevotella)	0.12341616	0.14339223	0	#FF8E8A	1
26.5	17	RBC	-10	41	OTU46 (g__Absconditabacteriales_)	0.110010664	0.192472005	0	#FF8E8A	1
27.5	16	RBC	-10	41	OTU9 (Porphyromonas)	0.087369027	0.301183597	0	#FF8E8A	0
28.5	15	RBC	-10	41	OTU62 (Aggregatibacter)	0.095708724	0.257204307	0	#FF8E8A	0
29.5	14	RBC	-10	41	OTU57 (Parvimonas)	0.039441449	0.641196639	0	#FF8E8A	0
30.5	13	RBC	-10	41	OTU45 (Alloprevotella)	0.0567808	0.502104167	0	#FF8E8A	0
31.5	12	RBC	-10	41	OTU67 (Alloprevotella)	0.105179898	0.212858536	0	#FF8E8A	1
32.5	11	RBC	-10	41	OTU29 (Porphyromonas)	0.108117111	0.200286977	0	#FF8E8A	1
33.5	10	RBC	-10	41	OTU365 (Fusobacterium)	0.133766639	0.112497777	0	#FF8E8A	1
34.5	9	RBC	-10	41	OTU169 (Treponema)	0.106819036	0.205775104	0	#FF8E8A	1
35.5	8	RBC	-10	41	OTU42 (g__Absconditabacteriales_)	0.025483736	0.763385069	0	#FF8E8A	0
36.5	7	RBC	-10	41	OTU380 (Porphyromonas)	0.132308599	0.116505357	0	#FF8E8A	1
37.5	6	RBC	-10	41	OTU678 (Neisseria)	0.0592316	0.483800839	0	#FF8E8A	0
38.5	5	RBC	-10	41	OTU75 (Porphyromonas)	0.130637256	0.121234728	0	#FF8E8A	1
39.5	4	RBC	-10	41	OTU134 (Prevotella)	0.105158229	0.21295333	0	#FF8E8A	1
40.5	3	RBC	-10	41	OTU2 (Fusobacterium)	0.151244161	0.072382832	0	#FF8E8A	1
41.5	2	RBC	-10	41	OTU523 (Prevotella)	0.034450237	0.683997455	0	#FF8E8A	0
42.5	1	RBC	-10	41	OTU4 (Prevotella)	0.069609284	0.410418226	0	#FF8E8A	0
-0.5	44	WBC	-9	37	OTU599 (Burkholderiales_unclassified)	0.10364808	0.219634069	0	#FF8E8A	1
0.5	43	WBC	-9	37	OTU744 (Leptotrichia)	-0.04481834	0.596376872	0	#00CCCC	0
1.5	42	WBC	-9	37	OTU658 (P5D1-392)	0.023645633	0.78000016	0	#FF8E8A	0
2.5	41	WBC	-9	37	OTU796 (Rothia)	-0.012550401	0.882153959	0	#00CCCC	0
3.5	40	WBC	-9	37	OTU748 (Leptotrichia)	-0.083878901	0.320978964	0	#00CCCC	0
4.5	39	WBC	-9	37	OTU762 (Streptococcus)	-0.063371392	0.453714841	0	#00CCCC	0
5.5	38	WBC	-9	37	OTU34 (Halomonas)	0.044124281	0.602082464	0	#FF8E8A	0
6.5	37	WBC	-9	37	OTU1 (Streptococcus)	0.09849543	0.243544699	0	#FF8E8A	0
7.5	36	WBC	-9	37	OTU126 ([Eubacterium]_ruminantium_group)	0.031040111	0.713839295	0	#FF8E8A	0
8.5	35	WBC	-9	37	OTU82 (Faecalibacterium)	0.021070719	0.803442041	0	#FF8E8A	0
9.5	34	WBC	-9	37	OTU69 (Fusicatenibacter)	0.06206207	0.463115786	0	#FF8E8A	0

10.5	33	WBC	-9	37	OTU85 (Prevotella)	0.10399282	0.218096026	0	#FF8E8A	1
11.5	32	WBC	-9	37	OTU252 (Prevotella)	0.07898686	0.350108609	0	#FF8E8A	0
12.5	31	WBC	-9	37	OTU129 (Acinetobacter)	0.047473461	0.574778512	0	#FF8E8A	0
13.5	30	WBC	-9	37	OTU31 (Pseudomonas)	0.083284732	0.324430887	0	#FF8E8A	0
14.5	29	WBC	-9	37	OTU15 (Campylobacter)	-0.003847617	0.963752963	0	#00CCCC	0
15.5	28	WBC	-9	37	OTU87 (Corynebacterium)	0.021749483	0.797244679	0	#FF8E8A	0
16.5	27	WBC	-9	37	OTU114 (Prevotella)	0.124483858	0.139935097	0	#FF8E8A	1
17.5	26	WBC	-9	37	OTU172 (Prevotella)	0.126761297	0.132773329	0	#FF8E8A	1
18.5	25	WBC	-9	37	OTU119 (Prevotella)	0.134524491	0.110457641	0	#FF8E8A	1
19.5	24	WBC	-9	37	OTU5 (Haemophilus)	0.074737075	0.376717572	0	#FF8E8A	0
20.5	23	WBC	-9	37	OTU171 (Aggregatibacter)	0.060434928	0.47494687	0	#FF8E8A	0
21.5	22	WBC	-9	37	OTU39 (Haemophilus)	0.059017155	0.485387952	0	#FF8E8A	0
22.5	21	WBC	-9	37	OTU20 (Actinobacillus)	-0.007896019	0.92569564	0	#00CCCC	0
23.5	20	WBC	-9	37	OTU10 (Alloprevotella)	0.120522915	0.153085961	0	#FF8E8A	1
24.5	19	WBC	-9	37	OTU26 (Prevotella)	0.077676088	0.35818692	0	#FF8E8A	0
25.5	18	WBC	-9	37	OTU71 (Alloprevotella)	0.038847094	0.646235384	0	#FF8E8A	0
26.5	17	WBC	-9	37	OTU46 (g__Absconditabacteriales_)	0.120757956	0.152280519	0	#FF8E8A	1
27.5	16	WBC	-9	37	OTU9 (Porphyromonas)	0.093695448	0.267394193	0	#FF8E8A	0
28.5	15	WBC	-9	37	OTU62 (Aggregatibacter)	0.193528696	0.021019269	1	#FF8E8A	1
29.5	14	WBC	-9	37	OTU57 (Parvimonas)	0.252299692	0.002452961	1	#FF8E8A	2
30.5	13	WBC	-9	37	OTU45 (Alloprevotella)	0.163388748	0.052031926	0	#FF8E8A	1
31.5	12	WBC	-9	37	OTU67 (Alloprevotella)	0.142701108	0.090240295	0	#FF8E8A	1
32.5	11	WBC	-9	37	OTU29 (Porphyromonas)	0.237115397	0.004494168	1	#FF8E8A	2
33.5	10	WBC	-9	37	OTU365 (Fusobacterium)	0.19078358	0.022948797	1	#FF8E8A	1
34.5	9	WBC	-9	37	OTU169 (Treponema)	0.15224078	0.070500494	0	#FF8E8A	1
35.5	8	WBC	-9	37	OTU42 (g__Absconditabacteriales_)	0.127714184	0.129861506	0	#FF8E8A	1
36.5	7	WBC	-9	37	OTU380 (Porphyromonas)	0.153847123	0.067550328	0	#FF8E8A	1
37.5	6	WBC	-9	37	OTU678 (Neisseria)	0.071091837	0.400497131	0	#FF8E8A	0
38.5	5	WBC	-9	37	OTU75 (Porphyromonas)	0.148349613	0.078081564	0	#FF8E8A	1
39.5	4	WBC	-9	37	OTU134 (Prevotella)	0.189004809	0.024278756	1	#FF8E8A	1
40.5	3	WBC	-9	37	OTU2 (Fusobacterium)	0.12236695	0.146852266	0	#FF8E8A	1
41.5	2	WBC	-9	37	OTU523 (Prevotella)	0.029120771	0.730831344	0	#FF8E8A	0
42.5	1	WBC	-9	37	OTU4 (Prevotella)	0.015836808	0.851612758	0	#FF8E8A	0
-0.5	44	NEUT	-8	33	OTU599 (Burkholderiales_unclassified)	0.209924443	0.012161164	1	#FF8E8A	2
0.5	43	NEUT	-8	33	OTU744 (Leptotrichia)	0.087655691	0.29959423	0	#FF8E8A	0
1.5	42	NEUT	-8	33	OTU658 (P5D1-392)	0.167300886	0.046585645	1	#FF8E8A	1
2.5	41	NEUT	-8	33	OTU796 (Rothia)	0.082663913	0.328063088	0	#FF8E8A	0
3.5	40	NEUT	-8	33	OTU748 (Leptotrichia)	0.08751598	0.300368146	0	#FF8E8A	0
4.5	39	NEUT	-8	33	OTU762 (Streptococcus)	0.083222557	0.324793484	0	#FF8E8A	0
5.5	38	NEUT	-8	33	OTU34 (Halomonas)	0.14039873	0.095609576	0	#FF8E8A	1
6.5	37	NEUT	-8	33	OTU1 (Streptococcus)	0.106449727	0.207356072	0	#FF8E8A	1
7.5	36	NEUT	-8	33	OTU126 ([Eubacterium]_ruminantium_group)	-0.148161563	0.078463953	0	#00CCCC	1
8.5	35	NEUT	-8	33	OTU82 (Faecalibacterium)	-0.121895684	0.148426788	0	#00CCCC	1
9.5	34	NEUT	-8	33	OTU69 (Fusicatenibacter)	-0.109159824	0.195955667	0	#00CCCC	1
10.5	33	NEUT	-8	33	OTU85 (Prevotella)	-0.078176698	0.355088059	0	#00CCCC	0
11.5	32	NEUT	-8	33	OTU252 (Prevotella)	-0.135509457	0.107849449	0	#00CCCC	1
12.5	31	NEUT	-8	33	OTU129 (Acinetobacter)	-0.109698352	0.19374546	0	#00CCCC	1
13.5	30	NEUT	-8	33	OTU31 (Pseudomonas)	-0.197499089	0.018476591	1	#00CCCC	1
14.5	29	NEUT	-8	33	OTU15 (Campylobacter)	-0.16548822	0.049047068	1	#00CCCC	1
15.5	28	NEUT	-8	33	OTU87 (Corynebacterium)	-0.169373633	0.043897979	1	#00CCCC	1
16.5	27	NEUT	-8	33	OTU114 (Prevotella)	-0.021585325	0.798742359	0	#00CCCC	0
17.5	26	NEUT	-8	33	OTU172 (Prevotella)	0.014327194	0.865617389	0	#FF8E8A	0
18.5	25	NEUT	-8	33	OTU119 (Prevotella)	-0.035151332	0.677920439	0	#00CCCC	0
19.5	24	NEUT	-8	33	OTU5 (Haemophilus)	-0.14818424	0.078417761	0	#00CCCC	1
20.5	23	NEUT	-8	33	OTU171 (Aggregatibacter)	-0.168365054	0.045189139	1	#00CCCC	1
21.5	22	NEUT	-8	33	OTU39 (Haemophilus)	-0.125234239	0.137543632	0	#00CCCC	1
22.5	21	NEUT	-8	33	OTU20 (Actinobacillus)	-0.204233948	0.01476992	1	#00CCCC	2
23.5	20	NEUT	-8	33	OTU10 (Alloprevotella)	-0.179727711	0.032335979	1	#00CCCC	1

24.5	19	NEUT	-8	33	OTU26 (Prevotella)	-0.171293129	0.041525753	1	#00CCCC	1
25.5	18	NEUT	-8	33	OTU71 (Alloprevotella)	-0.177441734	0.034637817	1	#00CCCC	1
26.5	17	NEUT	-8	33	OTU46 (g__Absconditabacteriales_)	-0.08053735	0.340701803	0	#00CCCC	0
27.5	16	NEUT	-8	33	OTU9 (Porphyromonas)	0.036369733	0.667408818	0	#FF8E8A	0
28.5	15	NEUT	-8	33	OTU62 (Aggregatibacter)	-0.037664639	0.656307536	0	#00CCCC	0
29.5	14	NEUT	-8	33	OTU57 (Parvimonas)	0.056727442	0.50250665	0	#FF8E8A	0
30.5	13	NEUT	-8	33	OTU45 (Alloprevotella)	-0.06606376	0.434722364	0	#00CCCC	0
31.5	12	NEUT	-8	33	OTU67 (Alloprevotella)	-0.017382572	0.837320602	0	#00CCCC	0
32.5	11	NEUT	-8	33	OTU29 (Porphyromonas)	-0.00286266	0.973027793	0	#00CCCC	0
33.5	10	NEUT	-8	33	OTU365 (Fusobacterium)	0.02759876	0.744399793	0	#FF8E8A	0
34.5	9	NEUT	-8	33	OTU169 (Treponema)	-0.072491308	0.391264099	0	#00CCCC	0
35.5	8	NEUT	-8	33	OTU42 (g__Absconditabacteriales_)	-0.075018519	0.374918134	0	#00CCCC	0
36.5	7	NEUT	-8	33	OTU380 (Porphyromonas)	0.089727427	0.288272545	0	#FF8E8A	0
37.5	6	NEUT	-8	33	OTU678 (Neisseria)	-0.095035259	0.260582842	0	#00CCCC	0
38.5	5	NEUT	-8	33	OTU75 (Porphyromonas)	0.085569805	0.311285766	0	#FF8E8A	0
39.5	4	NEUT	-8	33	OTU134 (Prevotella)	0.013767219	0.870823138	0	#FF8E8A	0
40.5	3	NEUT	-8	33	OTU2 (Fusobacterium)	0.011224205	0.894530433	0	#FF8E8A	0
41.5	2	NEUT	-8	33	OTU523 (Prevotella)	-0.119238052	0.157545697	0	#00CCCC	1
42.5	1	NEUT	-8	33	OTU4 (Prevotella)	-0.128751443	0.126747874	0	#00CCCC	1
-0.5	44	LYMPI	-7	29	OTU599 (Burkholderiales_unclassified)	-0.026740908	0.752082425	0	#00CCCC	0
0.5	43	LYMPI	-7	29	OTU744 (Leptotrichia)	-0.089185739	0.291204848	0	#00CCCC	0
1.5	42	LYMPI	-7	29	OTU658 (P5D1-392)	-0.180326902	0.031754537	1	#00CCCC	1
2.5	41	LYMPI	-7	29	OTU796 (Rothia)	-0.164619709	0.050264141	0	#00CCCC	1
3.5	40	LYMPI	-7	29	OTU748 (Leptotrichia)	-0.20248822	0.015662424	1	#00CCCC	2
4.5	39	LYMPI	-7	29	OTU762 (Streptococcus)	-0.19784073	0.018270801	1	#00CCCC	1
5.5	38	LYMPI	-7	29	OTU34 (Halomonas)	-0.138667061	0.099812242	0	#00CCCC	1
6.5	37	LYMPI	-7	29	OTU1 (Streptococcus)	0.024585689	0.771489807	0	#FF8E8A	0
7.5	36	LYMPI	-7	29	OTU126 ([Eubacterium]_ruminantium_group)	0.153866342	0.067515648	0	#FF8E8A	1
8.5	35	LYMPI	-7	29	OTU82 (Faecalibacterium)	0.116536649	0.167238719	0	#FF8E8A	1
9.5	34	LYMPI	-7	29	OTU69 (Fusicatenibacter)	0.115092099	0.172600489	0	#FF8E8A	1
10.5	33	LYMPI	-7	29	OTU85 (Prevotella)	0.149124378	0.076521929	0	#FF8E8A	1
11.5	32	LYMPI	-7	29	OTU252 (Prevotella)	0.183931223	0.02844092	1	#FF8E8A	1
12.5	31	LYMPI	-7	29	OTU129 (Acinetobacter)	0.228263228	0.00629137	1	#FF8E8A	2
13.5	30	LYMPI	-7	29	OTU31 (Pseudomonas)	0.253001969	0.002383114	1	#FF8E8A	2
14.5	29	LYMPI	-7	29	OTU15 (Campylobacter)	0.047470746	0.574800413	0	#FF8E8A	0
15.5	28	LYMPI	-7	29	OTU87 (Corynebacterium)	0.102711311	0.223852285	0	#FF8E8A	1
16.5	27	LYMPI	-7	29	OTU114 (Prevotella)	0.118600712	0.159793703	0	#FF8E8A	1
17.5	26	LYMPI	-7	29	OTU172 (Prevotella)	0.174892831	0.03736616	1	#FF8E8A	1
18.5	25	LYMPI	-7	29	OTU119 (Prevotella)	0.209877797	0.012180789	1	#FF8E8A	2
19.5	24	LYMPI	-7	29	OTU5 (Haemophilus)	0.222023589	0.007917663	1	#FF8E8A	2
20.5	23	LYMPI	-7	29	OTU171 (Aggregatibacter)	0.169740509	0.043436021	1	#FF8E8A	1
21.5	22	LYMPI	-7	29	OTU39 (Haemophilus)	0.171927533	0.040765723	1	#FF8E8A	1
22.5	21	LYMPI	-7	29	OTU20 (Actinobacillus)	0.169590488	0.043624429	1	#FF8E8A	1
23.5	20	LYMPI	-7	29	OTU10 (Alloprevotella)	0.229610703	0.005981966	1	#FF8E8A	2
24.5	19	LYMPI	-7	29	OTU26 (Prevotella)	0.196903773	0.018839982	1	#FF8E8A	1
25.5	18	LYMPI	-7	29	OTU71 (Alloprevotella)	0.137077493	0.103797091	0	#FF8E8A	1
26.5	17	LYMPI	-7	29	OTU46 (g__Absconditabacteriales_)	0.179251815	0.032804186	1	#FF8E8A	1
27.5	16	LYMPI	-7	29	OTU9 (Porphyromonas)	0.153948365	0.067367808	0	#FF8E8A	1
28.5	15	LYMPI	-7	29	OTU62 (Aggregatibacter)	0.20887511	0.012609401	1	#FF8E8A	2
29.5	14	LYMPI	-7	29	OTU57 (Parvimonas)	0.241999283	0.003713552	1	#FF8E8A	2
30.5	13	LYMPI	-7	29	OTU45 (Alloprevotella)	0.182123988	0.030063591	1	#FF8E8A	1
31.5	12	LYMPI	-7	29	OTU67 (Alloprevotella)	0.21256937	0.011092775	1	#FF8E8A	2
32.5	11	LYMPI	-7	29	OTU29 (Porphyromonas)	0.256220697	0.002085473	1	#FF8E8A	2
33.5	10	LYMPI	-7	29	OTU365 (Fusobacterium)	0.272599037	0.001030489	1	#FF8E8A	2
34.5	9	LYMPI	-7	29	OTU169 (Treponema)	0.230729523	0.005735449	1	#FF8E8A	2
35.5	8	LYMPI	-7	29	OTU42 (g__Absconditabacteriales_)	0.230236784	0.005842878	1	#FF8E8A	2
36.5	7	LYMPI	-7	29	OTU380 (Porphyromonas)	0.16885959	0.044552142	1	#FF8E8A	1
37.5	6	LYMPI	-7	29	OTU678 (Neisseria)	0.169930936	0.043197848	1	#FF8E8A	1

38.5	5	LYMPI	-7	29	OTU75 (Porphyromonas)	0.169679712	0.043512293	1	#FF8E8A	1
39.5	4	LYMPI	-7	29	OTU134 (Prevotella)	0.174315287	0.038008893	1	#FF8E8A	1
40.5	3	LYMPI	-7	29	OTU2 (Fusobacterium)	0.22033648	0.008416977	1	#FF8E8A	2
41.5	2	LYMPI	-7	29	OTU523 (Prevotella)	-0.002858724	0.973064867	0	#00CCCC	0
42.5	1	LYMPI	-7	29	OTU4 (Prevotella)	-0.003246376	0.969414044	0	#00CCCC	0
-0.5	44	Hb	-6	25	OTU599 (Burkholderiales_unclassified)	0.030026139	0.722799188	0	#FF8E8A	0
0.5	43	Hb	-6	25	OTU744 (Leptotrichia)	-0.08216648	0.330992159	0	#00CCCC	0
1.5	42	Hb	-6	25	OTU658 (P5D1-392)	-0.059132104	0.484536863	0	#00CCCC	0
2.5	41	Hb	-6	25	OTU796 (Rothia)	0.035664374	0.673486521	0	#FF8E8A	0
3.5	40	Hb	-6	25	OTU748 (Leptotrichia)	-0.051659752	0.541488385	0	#00CCCC	0
4.5	39	Hb	-6	25	OTU762 (Streptococcus)	-0.151654241	0.071603434	0	#00CCCC	1
5.5	38	Hb	-6	25	OTU34 (Halomonas)	-0.203083371	0.015353018	1	#00CCCC	2
6.5	37	Hb	-6	25	OTU1 (Streptococcus)	-0.013348426	0.874720029	0	#00CCCC	0
7.5	36	Hb	-6	25	OTU126 ([Eubacterium]_ruminantium_group)	0.175570163	0.036624059	1	#FF8E8A	1
8.5	35	Hb	-6	25	OTU82 (Faecalibacterium)	0.125186578	0.137694595	0	#FF8E8A	1
9.5	34	Hb	-6	25	OTU69 (Fusicatenibacter)	0.087551461	0.300171478	0	#FF8E8A	0
10.5	33	Hb	-6	25	OTU85 (Prevotella)	0.147959067	0.078877399	0	#FF8E8A	1
11.5	32	Hb	-6	25	OTU252 (Prevotella)	0.168614661	0.044866684	1	#FF8E8A	1
12.5	31	Hb	-6	25	OTU129 (Acinetobacter)	0.148028551	0.078735333	0	#FF8E8A	1
13.5	30	Hb	-6	25	OTU31 (Pseudomonas)	0.256063868	0.002099154	1	#FF8E8A	2
14.5	29	Hb	-6	25	OTU15 (Campylobacter)	-0.031723541	0.707822086	0	#00CCCC	0
15.5	28	Hb	-6	25	OTU87 (Corynebacterium)	0.164136822	0.050951588	0	#FF8E8A	1
16.5	27	Hb	-6	25	OTU114 (Prevotella)	0.253139707	0.002369628	1	#FF8E8A	2
17.5	26	Hb	-6	25	OTU172 (Prevotella)	0.158642393	0.059336321	0	#FF8E8A	1
18.5	25	Hb	-6	25	OTU119 (Prevotella)	0.162105331	0.053929511	0	#FF8E8A	1
19.5	24	Hb	-6	25	OTU5 (Haemophilus)	0.216436096	0.009679364	1	#FF8E8A	2
20.5	23	Hb	-6	25	OTU171 (Aggregatibacter)	0.263848883	0.001510027	1	#FF8E8A	2
21.5	22	Hb	-6	25	OTU39 (Haemophilus)	0.209016375	0.012548227	1	#FF8E8A	2
22.5	21	Hb	-6	25	OTU20 (Actinobacillus)	0.082572559	0.328599766	0	#FF8E8A	0
23.5	20	Hb	-6	25	OTU10 (Alloprevotella)	0.172716057	0.039837343	1	#FF8E8A	1
24.5	19	Hb	-6	25	OTU26 (Prevotella)	0.145872009	0.0832416	0	#FF8E8A	1
25.5	18	Hb	-6	25	OTU71 (Alloprevotella)	0.276037641	8.84E-04	1	#FF8E8A	2
26.5	17	Hb	-6	25	OTU46 (g__Absconditabacteriales_)	0.242117693	0.003696243	1	#FF8E8A	2
27.5	16	Hb	-6	25	OTU9 (Porphyromonas)	0.172521463	0.040064785	1	#FF8E8A	1
28.5	15	Hb	-6	25	OTU62 (Aggregatibacter)	0.249366965	0.002765109	1	#FF8E8A	2
29.5	14	Hb	-6	25	OTU57 (Parvimonas)	0.225686521	0.006922945	1	#FF8E8A	2
30.5	13	Hb	-6	25	OTU45 (Alloprevotella)	0.224407494	0.00725689	1	#FF8E8A	2
31.5	12	Hb	-6	25	OTU67 (Alloprevotella)	0.175736564	0.036443659	1	#FF8E8A	1
32.5	11	Hb	-6	25	OTU29 (Porphyromonas)	0.257053311	0.002014182	1	#FF8E8A	2
33.5	10	Hb	-6	25	OTU365 (Fusobacterium)	0.213028653	0.010915907	1	#FF8E8A	2
34.5	9	Hb	-6	25	OTU169 (Treponema)	0.273328201	9.98E-04	1	#FF8E8A	2
35.5	8	Hb	-6	25	OTU42 (g__Absconditabacteriales_)	0.154242147	0.066840445	0	#FF8E8A	1
36.5	7	Hb	-6	25	OTU380 (Porphyromonas)	0.188918765	0.024344731	1	#FF8E8A	1
37.5	6	Hb	-6	25	OTU678 (Neisseria)	0.148965365	0.076839963	0	#FF8E8A	1
38.5	5	Hb	-6	25	OTU75 (Porphyromonas)	0.246454814	0.003110149	1	#FF8E8A	2
39.5	4	Hb	-6	25	OTU134 (Prevotella)	0.073914484	0.38200701	0	#FF8E8A	0
40.5	3	Hb	-6	25	OTU2 (Fusobacterium)	0.204561279	0.014607602	1	#FF8E8A	2
41.5	2	Hb	-6	25	OTU523 (Prevotella)	0.128667215	0.126998545	0	#FF8E8A	1
42.5	1	Hb	-6	25	OTU4 (Prevotella)	0.14992664	0.074933523	0	#FF8E8A	1
-0.5	44	PLT	-5	21	OTU599 (Burkholderiales_unclassified)	-0.053819473	0.524694448	0	#00CCCC	0
0.5	43	PLT	-5	21	OTU744 (Leptotrichia)	-0.258888756	0.001864778	1	#00CCCC	2
1.5	42	PLT	-5	21	OTU658 (P5D1-392)	-0.119453277	0.156791942	0	#00CCCC	1
2.5	41	PLT	-5	21	OTU796 (Rothia)	-0.020180148	0.81159179	0	#00CCCC	0
3.5	40	PLT	-5	21	OTU748 (Leptotrichia)	-0.252935631	0.002389634	1	#00CCCC	2
4.5	39	PLT	-5	21	OTU762 (Streptococcus)	-0.123818374	0.142082359	0	#00CCCC	1
5.5	38	PLT	-5	21	OTU34 (Halomonas)	-0.033089327	0.69585135	0	#00CCCC	0
6.5	37	PLT	-5	21	OTU1 (Streptococcus)	0.010696465	0.89946272	0	#FF8E8A	0

7.5	36	PLT	-5	21	OTU126 ([Eubacterium]_ruminantium_group)	0.131219702	0.119570008	0	#FF8E8A	1
8.5	35	PLT	-5	21	OTU82 (Faecalibacterium)	0.172031698	0.040642052	1	#FF8E8A	1
9.5	34	PLT	-5	21	OTU69 (Fusicatenibacter)	0.223760571	0.007431182	1	#FF8E8A	2
10.5	33	PLT	-5	21	OTU85 (Prevotella)	0.183156775	0.029126902	1	#FF8E8A	1
11.5	32	PLT	-5	21	OTU252 (Prevotella)	0.193294258	0.021178407	1	#FF8E8A	1
12.5	31	PLT	-5	21	OTU129 (Acinetobacter)	0.227965694	0.006361579	1	#FF8E8A	2
13.5	30	PLT	-5	21	OTU31 (Pseudomonas)	0.277696882	8.20E-04	1	#FF8E8A	2
14.5	29	PLT	-5	21	OTU15 (Campylobacter)	0.116380882	0.167810855	0	#FF8E8A	1
15.5	28	PLT	-5	21	OTU87 (Corynebacterium)	0.272729832	0.001024522	1	#FF8E8A	2
16.5	27	PLT	-5	21	OTU114 (Prevotella)	0.213797643	0.010625318	1	#FF8E8A	2
17.5	26	PLT	-5	21	OTU172 (Prevotella)	0.244792463	0.003324082	1	#FF8E8A	2
18.5	25	PLT	-5	21	OTU119 (Prevotella)	0.235804286	0.004727364	1	#FF8E8A	2
19.5	24	PLT	-5	21	OTU5 (Haemophilus)	0.159294335	0.058285738	0	#FF8E8A	1
20.5	23	PLT	-5	21	OTU171 (Aggregatibacter)	0.210136919	0.012072122	1	#FF8E8A	2
21.5	22	PLT	-5	21	OTU39 (Haemophilus)	0.122677507	0.145821609	0	#FF8E8A	1
22.5	21	PLT	-5	21	OTU20 (Actinobacillus)	0.185290489	0.027270195	1	#FF8E8A	1
23.5	20	PLT	-5	21	OTU10 (Alloprevotella)	0.288714661	4.93E-04	1	#FF8E8A	2
24.5	19	PLT	-5	21	OTU26 (Prevotella)	0.258073744	0.00192983	1	#FF8E8A	2
25.5	18	PLT	-5	21	OTU71 (Alloprevotella)	0.226312355	0.006764576	1	#FF8E8A	2
26.5	17	PLT	-5	21	OTU46 (g__Absconditabacteriales_)	0.199711405	0.017178819	1	#FF8E8A	1
27.5	16	PLT	-5	21	OTU9 (Porphyromonas)	0.100027978	0.236251371	0	#FF8E8A	1
28.5	15	PLT	-5	21	OTU62 (Aggregatibacter)	0.259337106	0.001829848	1	#FF8E8A	2
29.5	14	PLT	-5	21	OTU57 (Parvimonas)	0.303577831	2.40E-04	1	#FF8E8A	3
30.5	13	PLT	-5	21	OTU45 (Alloprevotella)	0.262332624	0.001611327	1	#FF8E8A	2
31.5	12	PLT	-5	21	OTU67 (Alloprevotella)	0.184739844	0.027739408	1	#FF8E8A	1
32.5	11	PLT	-5	21	OTU29 (Porphyromonas)	0.234306065	0.005007058	1	#FF8E8A	2
33.5	10	PLT	-5	21	OTU365 (Fusobacterium)	0.279590254	7.52E-04	1	#FF8E8A	2
34.5	9	PLT	-5	21	OTU169 (Treponema)	0.284661052	5.96E-04	1	#FF8E8A	2
35.5	8	PLT	-5	21	OTU42 (g__Absconditabacteriales_)	0.247279725	0.003008669	1	#FF8E8A	2
36.5	7	PLT	-5	21	OTU380 (Porphyromonas)	0.103480621	0.220383956	0	#FF8E8A	1
37.5	6	PLT	-5	21	OTU678 (Neisseria)	0.121563535	0.149544155	0	#FF8E8A	1
38.5	5	PLT	-5	21	OTU75 (Porphyromonas)	0.196836227	0.018881601	1	#FF8E8A	1
39.5	4	PLT	-5	21	OTU134 (Prevotella)	0.262401402	0.001606601	1	#FF8E8A	2
40.5	3	PLT	-5	21	OTU2 (Fusobacterium)	0.078167602	0.355144219	0	#FF8E8A	0
41.5	2	PLT	-5	21	OTU523 (Prevotella)	0.232870626	0.00528882	1	#FF8E8A	2
42.5	1	PLT	-5	21	OTU4 (Prevotella)	0.259892239	0.001787423	1	#FF8E8A	2
-0.5	44	ALT	-4	17	OTU599 (Burkholderiales_unclassified)	0.029526381	0.72722922	0	#FF8E8A	0
0.5	43	ALT	-4	17	OTU744 (Leptotrichia)	0.104376169	0.216394737	0	#FF8E8A	1
1.5	42	ALT	-4	17	OTU658 (P5D1-392)	0.058418912	0.489830263	0	#FF8E8A	0
2.5	41	ALT	-4	17	OTU796 (Rothia)	0.132904107	0.114855339	0	#FF8E8A	1
3.5	40	ALT	-4	17	OTU748 (Leptotrichia)	0.059699682	0.480346247	0	#FF8E8A	0
4.5	39	ALT	-4	17	OTU762 (Streptococcus)	-0.019159486	0.82095688	0	#00CCCC	0
5.5	38	ALT	-4	17	OTU34 (Halomonas)	0.077739463	0.357793692	0	#FF8E8A	0
6.5	37	ALT	-4	17	OTU1 (Streptococcus)	-0.113687064	0.177936583	0	#00CCCC	1
7.5	36	ALT	-4	17	OTU126 ([Eubacterium]_ruminantium_group)	-0.010088416	0.905150322	0	#00CCCC	0
8.5	35	ALT	-4	17	OTU82 (Faecalibacterium)	0.010533205	0.900989336	0	#FF8E8A	0
9.5	34	ALT	-4	17	OTU69 (Fusicatenibacter)	-0.045977093	0.586905904	0	#00CCCC	0
10.5	33	ALT	-4	17	OTU85 (Prevotella)	0.01560423	0.853767472	0	#FF8E8A	0
11.5	32	ALT	-4	17	OTU252 (Prevotella)	-0.029919852	0.72374059	0	#00CCCC	0
12.5	31	ALT	-4	17	OTU129 (Acinetobacter)	-0.079959577	0.344188259	0	#00CCCC	0
13.5	30	ALT	-4	17	OTU31 (Pseudomonas)	-0.079183065	0.348909307	0	#00CCCC	0
14.5	29	ALT	-4	17	OTU15 (Campylobacter)	-0.183773398	0.028579588	1	#00CCCC	1
15.5	28	ALT	-4	17	OTU87 (Corynebacterium)	0.021964526	0.795283871	0	#FF8E8A	0
16.5	27	ALT	-4	17	OTU114 (Prevotella)	-4.29E-04	0.995955814	0	#00CCCC	0
17.5	26	ALT	-4	17	OTU172 (Prevotella)	-0.062993788	0.456415065	0	#00CCCC	0
18.5	25	ALT	-4	17	OTU119 (Prevotella)	-0.094285282	0.264380811	0	#00CCCC	0
19.5	24	ALT	-4	17	OTU5 (Haemophilus)	-0.033257539	0.694382117	0	#00CCCC	0

20.5	23	ALT	-4	17	OTU171 (Aggregatibacter)	0.019315466	0.819524012	0	#FF8E8A	0
21.5	22	ALT	-4	17	OTU39 (Haemophilus)	-0.100042339	0.236183755	0	#00CCCC	1
22.5	21	ALT	-4	17	OTU20 (Actinobacillus)	-0.042277056	0.617385008	0	#00CCCC	0
23.5	20	ALT	-4	17	OTU10 (Alloprevotella)	-0.065375369	0.439534704	0	#00CCCC	0
24.5	19	ALT	-4	17	OTU26 (Prevotella)	-0.043463361	0.607538148	0	#00CCCC	0
25.5	18	ALT	-4	17	OTU71 (Alloprevotella)	-0.079093563	0.349456068	0	#00CCCC	0
26.5	17	ALT	-4	17	OTU46 (g__Absconditabacteriales_)	-0.078552838	0.352770742	0	#00CCCC	0
27.5	16	ALT	-4	17	OTU9 (Porphyromonas)	-0.048654483	0.565290454	0	#00CCCC	0
28.5	15	ALT	-4	17	OTU62 (Aggregatibacter)	-0.076809284	0.363592245	0	#00CCCC	0
29.5	14	ALT	-4	17	OTU57 (Parvimonas)	0.032628662	0.699880757	0	#FF8E8A	0
30.5	13	ALT	-4	17	OTU45 (Alloprevotella)	-0.066499592	0.431691206	0	#00CCCC	0
31.5	12	ALT	-4	17	OTU67 (Alloprevotella)	-0.077935391	0.3565797	0	#00CCCC	0
32.5	11	ALT	-4	17	OTU29 (Porphyromonas)	0.122289821	0.147109089	0	#FF8E8A	1
33.5	10	ALT	-4	17	OTU365 (Fusobacterium)	0.037393647	0.658624653	0	#FF8E8A	0
34.5	9	ALT	-4	17	OTU169 (Treponema)	0.035155547	0.677883968	0	#FF8E8A	0
35.5	8	ALT	-4	17	OTU42 (g__Absconditabacteriales_)	-0.206787088	0.013544543	1	#00CCCC	2
36.5	7	ALT	-4	17	OTU380 (Porphyromonas)	-0.044598521	0.598181285	0	#00CCCC	0
37.5	6	ALT	-4	17	OTU678 (Neisseria)	-0.033983067	0.688058135	0	#00CCCC	0
38.5	5	ALT	-4	17	OTU75 (Porphyromonas)	-0.082742599	0.327601283	0	#00CCCC	0
39.5	4	ALT	-4	17	OTU134 (Prevotella)	-0.072441565	0.391590072	0	#00CCCC	0
40.5	3	ALT	-4	17	OTU2 (Fusobacterium)	0.154511998	0.066359007	0	#FF8E8A	1
41.5	2	ALT	-4	17	OTU523 (Prevotella)	-0.056042852	0.507685527	0	#00CCCC	0
42.5	1	ALT	-4	17	OTU4 (Prevotella)	-0.070327083	0.405596868	0	#00CCCC	0
-0.5	44	AST	-3	13	OTU599 (Burkholderiales_unclassified)	-0.108454238	0.198879094	0	#00CCCC	1
0.5	43	AST	-3	13	OTU744 (Leptotrichia)	-0.002228241	0.979003776	0	#00CCCC	0
1.5	42	AST	-3	13	OTU658 (P5D1-392)	0.032008928	0.705314719	0	#FF8E8A	0
2.5	41	AST	-3	13	OTU796 (Rothia)	0.032485966	0.701130613	0	#FF8E8A	0
3.5	40	AST	-3	13	OTU748 (Leptotrichia)	-0.021491072	0.799602598	0	#00CCCC	0
4.5	39	AST	-3	13	OTU762 (Streptococcus)	-0.013755487	0.870932262	0	#00CCCC	0
5.5	38	AST	-3	13	OTU34 (Halomonas)	0.242002333	0.003713106	1	#FF8E8A	2
6.5	37	AST	-3	13	OTU1 (Streptococcus)	-0.067317473	0.426035786	0	#00CCCC	0
7.5	36	AST	-3	13	OTU126 ([Eubacterium]_ruminantium_group)	-0.060035002	0.477879682	0	#00CCCC	0
8.5	35	AST	-3	13	OTU82 (Faecalibacterium)	-0.007755296	0.927016198	0	#00CCCC	0
9.5	34	AST	-3	13	OTU69 (Fusicatenibacter)	-0.085710714	0.310486714	0	#00CCCC	0
10.5	33	AST	-3	13	OTU85 (Prevotella)	-0.024935039	0.768333986	0	#00CCCC	0
11.5	32	AST	-3	13	OTU252 (Prevotella)	-0.057705091	0.495158999	0	#00CCCC	0
12.5	31	AST	-3	13	OTU129 (Acinetobacter)	-0.046718838	0.580879736	0	#00CCCC	0
13.5	30	AST	-3	13	OTU31 (Pseudomonas)	-0.108533108	0.198550758	0	#00CCCC	1
14.5	29	AST	-3	13	OTU15 (Campylobacter)	-0.106222031	0.208335143	0	#00CCCC	1
15.5	28	AST	-3	13	OTU87 (Corynebacterium)	-0.069024068	0.414373848	0	#00CCCC	0
16.5	27	AST	-3	13	OTU114 (Prevotella)	-0.009261058	0.912897005	0	#00CCCC	0
17.5	26	AST	-3	13	OTU172 (Prevotella)	-0.086267696	0.307341343	0	#00CCCC	0
18.5	25	AST	-3	13	OTU119 (Prevotella)	-0.084292252	0.318591581	0	#00CCCC	0
19.5	24	AST	-3	13	OTU5 (Haemophilus)	-0.130302447	0.122199749	0	#00CCCC	1
20.5	23	AST	-3	13	OTU171 (Aggregatibacter)	-0.060998443	0.47083103	0	#00CCCC	0
21.5	22	AST	-3	13	OTU39 (Haemophilus)	-0.116621269	0.166928517	0	#00CCCC	1
22.5	21	AST	-3	13	OTU20 (Actinobacillus)	-0.086739357	0.304694178	0	#00CCCC	0
23.5	20	AST	-3	13	OTU10 (Alloprevotella)	-0.123615422	0.142742163	0	#00CCCC	1
24.5	19	AST	-3	13	OTU26 (Prevotella)	-0.074245214	0.379874949	0	#00CCCC	0
25.5	18	AST	-3	13	OTU71 (Alloprevotella)	-0.188037215	0.025029578	1	#00CCCC	1
26.5	17	AST	-3	13	OTU46 (g__Absconditabacteriales_)	-0.137192176	0.103505473	0	#00CCCC	1
27.5	16	AST	-3	13	OTU9 (Porphyromonas)	0.022993339	0.785920837	0	#FF8E8A	0
28.5	15	AST	-3	13	OTU62 (Aggregatibacter)	-0.179961108	0.032108432	1	#00CCCC	1
29.5	14	AST	-3	13	OTU57 (Parvimonas)	-0.098849263	0.241847087	0	#00CCCC	0
30.5	13	AST	-3	13	OTU45 (Alloprevotella)	-0.174109568	0.038240066	1	#00CCCC	1
31.5	12	AST	-3	13	OTU67 (Alloprevotella)	-0.138575415	0.100038659	0	#00CCCC	1
32.5	11	AST	-3	13	OTU29 (Porphyromonas)	-0.00450955	0.957522524	0	#00CCCC	0
33.5	10	AST	-3	13	OTU365 (Fusobacterium)	-0.081002204	0.337913043	0	#00CCCC	0

34.5	9	AST	-3	13	OTU169 (Treponema)	-0.007340226	0.930912407	0	#00CCCC	0
35.5	8	AST	-3	13	OTU42 (g__Absconditabacteriales_)	-0.272790843	0.001021749	1	#00CCCC	2
36.5	7	AST	-3	13	OTU380 (Porphyromonas)	0.034742621	0.681460615	0	#FF8E8A	0
37.5	6	AST	-3	13	OTU678 (Neisseria)	-0.150189981	0.074417975	0	#00CCCC	1
38.5	5	AST	-3	13	OTU75 (Porphyromonas)	-0.168254983	0.045331946	1	#00CCCC	1
39.5	4	AST	-3	13	OTU134 (Prevotella)	-0.192626622	0.021637301	1	#00CCCC	1
40.5	3	AST	-3	13	OTU2 (Fusobacterium)	-0.07482588	0.376149222	0	#00CCCC	0
41.5	2	AST	-3	13	OTU523 (Prevotella)	-0.177829888	0.034237483	1	#00CCCC	1
42.5	1	AST	-3	13	OTU4 (Prevotella)	-0.199336967	0.017392743	1	#00CCCC	1
-0.5	44	Alb	-2	9	OTU599 (Burkholderiales_unclassified)	0.029794547	0.724850969	0	#FF8E8A	0
0.5	43	Alb	-2	9	OTU744 (Leptotrichia)	-0.058990898	0.485582473	0	#00CCCC	0
1.5	42	Alb	-2	9	OTU658 (P5D1-392)	-0.126994399	0.132056438	0	#00CCCC	1
2.5	41	Alb	-2	9	OTU796 (Rothia)	-0.189697403	0.023753258	1	#00CCCC	1
3.5	40	Alb	-2	9	OTU748 (Leptotrichia)	-0.106965305	0.205151345	0	#00CCCC	1
4.5	39	Alb	-2	9	OTU762 (Streptococcus)	-0.230009597	0.005893012	1	#00CCCC	2
5.5	38	Alb	-2	9	OTU34 (Halomonas)	-0.311496272	1.61E-04	1	#00CCCC	3
6.5	37	Alb	-2	9	OTU1 (Streptococcus)	0.02651745	0.754087672	0	#FF8E8A	0
7.5	36	Alb	-2	9	OTU126 ([Eubacterium]_ruminantium_group)	0.211094431	0.011677913	1	#FF8E8A	2
8.5	35	Alb	-2	9	OTU82 (Faecalibacterium)	0.183050768	0.029221887	1	#FF8E8A	1
9.5	34	Alb	-2	9	OTU69 (Fusicatenibacter)	0.164816241	0.049986564	1	#FF8E8A	1
10.5	33	Alb	-2	9	OTU85 (Prevotella)	0.227599684	0.006448904	1	#FF8E8A	2
11.5	32	Alb	-2	9	OTU252 (Prevotella)	0.213108599	0.010885375	1	#FF8E8A	2
12.5	31	Alb	-2	9	OTU129 (Acinetobacter)	0.237730387	0.00438838	1	#FF8E8A	2
13.5	30	Alb	-2	9	OTU31 (Pseudomonas)	0.414147897	3.00E-07	1	#FF8E8A	4
14.5	29	Alb	-2	9	OTU15 (Campylobacter)	0.282143281	6.69E-04	1	#FF8E8A	2
15.5	28	Alb	-2	9	OTU87 (Corynebacterium)	0.238730253	0.004221147	1	#FF8E8A	2
16.5	27	Alb	-2	9	OTU114 (Prevotella)	0.333169858	5.09E-05	1	#FF8E8A	3
17.5	26	Alb	-2	9	OTU172 (Prevotella)	0.260386191	0.001750427	1	#FF8E8A	2
18.5	25	Alb	-2	9	OTU119 (Prevotella)	0.285314749	5.78E-04	1	#FF8E8A	2
19.5	24	Alb	-2	9	OTU5 (Haemophilus)	0.438033564	4.99E-08	1	#FF8E8A	4
20.5	23	Alb	-2	9	OTU171 (Aggregatibacter)	0.39606169	1.07E-06	1	#FF8E8A	3
21.5	22	Alb	-2	9	OTU39 (Haemophilus)	0.469441665	3.79E-09	1	#FF8E8A	4
22.5	21	Alb	-2	9	OTU20 (Actinobacillus)	0.414352625	2.96E-07	1	#FF8E8A	4
23.5	20	Alb	-2	9	OTU10 (Alloprevotella)	0.49631504	3.36E-10	1	#FF8E8A	4
24.5	19	Alb	-2	9	OTU26 (Prevotella)	0.368167348	6.55E-06	1	#FF8E8A	3
25.5	18	Alb	-2	9	OTU71 (Alloprevotella)	0.499552222	2.47E-10	1	#FF8E8A	4
26.5	17	Alb	-2	9	OTU46 (g__Absconditabacteriales_)	0.417823979	2.30E-07	1	#FF8E8A	4
27.5	16	Alb	-2	9	OTU9 (Porphyromonas)	0.313912403	1.42E-04	1	#FF8E8A	3
28.5	15	Alb	-2	9	OTU62 (Aggregatibacter)	0.498601364	2.71E-10	1	#FF8E8A	4
29.5	14	Alb	-2	9	OTU57 (Parvimonas)	0.28344439	6.30E-04	1	#FF8E8A	2
30.5	13	Alb	-2	9	OTU45 (Alloprevotella)	0.394308864	1.20E-06	1	#FF8E8A	3
31.5	12	Alb	-2	9	OTU67 (Alloprevotella)	0.418871728	2.13E-07	1	#FF8E8A	4
32.5	11	Alb	-2	9	OTU29 (Porphyromonas)	0.271892125	0.001063295	1	#FF8E8A	2
33.5	10	Alb	-2	9	OTU365 (Fusobacterium)	0.351257714	1.82E-05	1	#FF8E8A	3
34.5	9	Alb	-2	9	OTU169 (Treponema)	0.235897588	0.004710418	1	#FF8E8A	2
35.5	8	Alb	-2	9	OTU42 (g__Absconditabacteriales_)	0.386699416	2.00E-06	1	#FF8E8A	3
36.5	7	Alb	-2	9	OTU380 (Porphyromonas)	0.338072632	3.87E-05	1	#FF8E8A	3
37.5	6	Alb	-2	9	OTU678 (Neisseria)	0.245710207	0.00320439	1	#FF8E8A	2
38.5	5	Alb	-2	9	OTU75 (Porphyromonas)	0.295511767	3.56E-04	1	#FF8E8A	2
39.5	4	Alb	-2	9	OTU134 (Prevotella)	0.420496496	1.89E-07	1	#FF8E8A	4
40.5	3	Alb	-2	9	OTU2 (Fusobacterium)	0.381194889	2.87E-06	1	#FF8E8A	3
41.5	2	Alb	-2	9	OTU523 (Prevotella)	0.272292967	0.001044578	1	#FF8E8A	2
42.5	1	Alb	-2	9	OTU4 (Prevotella)	0.278708813	7.83E-04	1	#FF8E8A	2
-0.5	44	TBIL	-1	5	OTU599 (Burkholderiales_unclassified)	0.117246724	0.164648949	0	#FF8E8A	1
0.5	43	TBIL	-1	5	OTU744 (Leptotrichia)	0.272531151	0.001033599	1	#FF8E8A	2
1.5	42	TBIL	-1	5	OTU658 (P5D1-392)	0.163543807	0.051806452	0	#FF8E8A	1
2.5	41	TBIL	-1	5	OTU796 (Rothia)	0.100199488	0.235444772	0	#FF8E8A	1
3.5	40	TBIL	-1	5	OTU748 (Leptotrichia)	0.235322778	0.004815689	1	#FF8E8A	2

4.5	39	TBIL	-1	5	OTU762 (Streptococcus)	0.105998159	0.209301003	0	#FF8E8A	1
5.5	38	TBIL	-1	5	OTU34 (Halomonas)	-0.046054107	0.586278887	0	#00CCCC	0
6.5	37	TBIL	-1	5	OTU1 (Streptococcus)	0.048040521	0.570213582	0	#FF8E8A	0
7.5	36	TBIL	-1	5	OTU126 ([Eubacterium]_ruminantium_group)	-0.098334676	0.244318691	0	#00CCCC	0
8.5	35	TBIL	-1	5	OTU82 (Faecalibacterium)	-0.107863793	0.20134959	0	#00CCCC	1
9.5	34	TBIL	-1	5	OTU69 (Fusicatenibacter)	-0.063006791	0.456321932	0	#00CCCC	0
10.5	33	TBIL	-1	5	OTU85 (Prevotella)	-0.114994987	0.172965453	0	#00CCCC	1
11.5	32	TBIL	-1	5	OTU252 (Prevotella)	-0.107737436	0.20188115	0	#00CCCC	1
12.5	31	TBIL	-1	5	OTU129 (Acinetobacter)	-0.174908302	0.037349069	1	#00CCCC	1
13.5	30	TBIL	-1	5	OTU31 (Pseudomonas)	-0.071814548	0.395712967	0	#00CCCC	0
14.5	29	TBIL	-1	5	OTU15 (Campylobacter)	-0.052658381	0.533690175	0	#00CCCC	0
15.5	28	TBIL	-1	5	OTU87 (Corynebacterium)	-0.148865373	0.077040497	0	#00CCCC	1
16.5	27	TBIL	-1	5	OTU114 (Prevotella)	0.021538725	0.799167638	0	#FF8E8A	0
17.5	26	TBIL	-1	5	OTU172 (Prevotella)	-0.050085553	0.553894328	0	#00CCCC	0
18.5	25	TBIL	-1	5	OTU119 (Prevotella)	0.036371282	0.667395496	0	#FF8E8A	0
19.5	24	TBIL	-1	5	OTU5 (Haemophilus)	-0.037187186	0.660392188	0	#00CCCC	0
20.5	23	TBIL	-1	5	OTU171 (Aggregatibacter)	0.013301889	0.875153246	0	#FF8E8A	0
21.5	22	TBIL	-1	5	OTU39 (Haemophilus)	-0.018827176	0.824011518	0	#00CCCC	0
22.5	21	TBIL	-1	5	OTU20 (Actinobacillus)	-0.029838843	0.724458377	0	#00CCCC	0
23.5	20	TBIL	-1	5	OTU10 (Alloprevotella)	-0.059071846	0.484982923	0	#00CCCC	0
24.5	19	TBIL	-1	5	OTU26 (Prevotella)	-0.165527641	0.048992413	1	#00CCCC	1
25.5	18	TBIL	-1	5	OTU71 (Alloprevotella)	0.019289864	0.819759157	0	#FF8E8A	0
26.5	17	TBIL	-1	5	OTU46 (g__Absconditabacteriales_)	0.007065308	0.933493936	0	#FF8E8A	0
27.5	16	TBIL	-1	5	OTU9 (Porphyromonas)	-0.093656482	0.267594084	0	#00CCCC	0
28.5	15	TBIL	-1	5	OTU62 (Aggregatibacter)	0.082342439	0.329954139	0	#FF8E8A	0
29.5	14	TBIL	-1	5	OTU57 (Parvimonas)	-0.032923474	0.69730108	0	#00CCCC	0
30.5	13	TBIL	-1	5	OTU45 (Alloprevotella)	0.053772232	0.52505895	0	#FF8E8A	0
31.5	12	TBIL	-1	5	OTU67 (Alloprevotella)	-0.080990303	0.337984258	0	#00CCCC	0
32.5	11	TBIL	-1	5	OTU29 (Porphyromonas)	-0.012551564	0.882143115	0	#00CCCC	0
33.5	10	TBIL	-1	5	OTU365 (Fusobacterium)	0.010167907	0.904406496	0	#FF8E8A	0
34.5	9	TBIL	-1	5	OTU169 (Treponema)	0.054691985	0.51798545	0	#FF8E8A	0
35.5	8	TBIL	-1	5	OTU42 (g__Absconditabacteriales_)	0.0082207	0.922649572	0	#FF8E8A	0
36.5	7	TBIL	-1	5	OTU380 (Porphyromonas)	-0.044121963	0.602101563	0	#00CCCC	0
37.5	6	TBIL	-1	5	OTU678 (Neisseria)	-0.026194987	0.756984246	0	#00CCCC	0
38.5	5	TBIL	-1	5	OTU75 (Porphyromonas)	0.018652399	0.825619146	0	#FF8E8A	0
39.5	4	TBIL	-1	5	OTU134 (Prevotella)	0.039354432	0.641933324	0	#FF8E8A	0
40.5	3	TBIL	-1	5	OTU2 (Fusobacterium)	-0.02054778	0.808225031	0	#00CCCC	0
41.5	2	TBIL	-1	5	OTU523 (Prevotella)	-0.077169724	0.361338467	0	#00CCCC	0
42.5	1	TBIL	-1	5	OTU4 (Prevotella)	-0.054147225	0.522169112	0	#00CCCC	0
-0.5	44	CREA	0	1	OTU599 (Burkholderiales_unclassified)	0.111063096	0.188225527	0	#FF8E8A	1
0.5	43	CREA	0	1	OTU744 (Leptotrichia)	-0.015997465	0.850124985	0	#00CCCC	0
1.5	42	CREA	0	1	OTU658 (P5D1-392)	0.122601024	0.146074926	0	#FF8E8A	1
2.5	41	CREA	0	1	OTU796 (Rothia)	0.004055637	0.961794723	0	#FF8E8A	0
3.5	40	CREA	0	1	OTU748 (Leptotrichia)	0.069190564	0.413246194	0	#FF8E8A	0
4.5	39	CREA	0	1	OTU762 (Streptococcus)	0.018631666	0.825809905	0	#FF8E8A	0
5.5	38	CREA	0	1	OTU34 (Halomonas)	-0.040581072	0.631581055	0	#00CCCC	0
6.5	37	CREA	0	1	OTU1 (Streptococcus)	0.022269101	0.792508883	0	#FF8E8A	0
7.5	36	CREA	0	1	OTU126 ([Eubacterium]_ruminantium_group)	-0.075906628	0.369274483	0	#00CCCC	0
8.5	35	CREA	0	1	OTU82 (Faecalibacterium)	-0.092123725	0.275537632	0	#00CCCC	0
9.5	34	CREA	0	1	OTU69 (Fusicatenibacter)	-0.12692355	0.132274016	0	#00CCCC	1
10.5	33	CREA	0	1	OTU85 (Prevotella)	-0.102834536	0.223294161	0	#00CCCC	1
11.5	32	CREA	0	1	OTU252 (Prevotella)	-0.10541525	0.211830907	0	#00CCCC	1
12.5	31	CREA	0	1	OTU129 (Acinetobacter)	-0.075779572	0.370078661	0	#00CCCC	0
13.5	30	CREA	0	1	OTU31 (Pseudomonas)	0.00445611	0.958025444	0	#FF8E8A	0
14.5	29	CREA	0	1	OTU15 (Campylobacter)	-0.218778396	0.008902643	1	#00CCCC	2
15.5	28	CREA	0	1	OTU87 (Corynebacterium)	-0.011386999	0.893009763	0	#00CCCC	0
16.5	27	CREA	0	1	OTU114 (Prevotella)	0.073786728	0.382832529	0	#FF8E8A	0

17.5	26	CREA	0	1	OTU172 (Prevotella)	-0.006142107	0.942167863	0	#00CCCC	0
18.5	25	CREA	0	1	OTU119 (Prevotella)	0.058961573	0.485799773	0	#FF8E8A	0
19.5	24	CREA	0	1	OTU5 (Haemophilus)	0.114944192	0.173156576	0	#FF8E8A	1
20.5	23	CREA	0	1	OTU171 (Aggregatibacter)	0.055510458	0.511732118	0	#FF8E8A	0
21.5	22	CREA	0	1	OTU39 (Haemophilus)	0.023938271	0.777348054	0	#FF8E8A	0
22.5	21	CREA	0	1	OTU20 (Actinobacillus)	-0.018564721	0.826425901	0	#00CCCC	0
23.5	20	CREA	0	1	OTU10 (Alloprevotella)	-0.030928548	0.714823232	0	#00CCCC	0
24.5	19	CREA	0	1	OTU26 (Prevotella)	-0.022150493	0.793589218	0	#00CCCC	0
25.5	18	CREA	0	1	OTU71 (Alloprevotella)	0.030117189	0.721993074	0	#FF8E8A	0
26.5	17	CREA	0	1	OTU46 (g__Absconditabacteriales_)	0.011336918	0.893477533	0	#FF8E8A	0
27.5	16	CREA	0	1	OTU9 (Porphyromonas)	0.136576278	0.105079181	0	#FF8E8A	1
28.5	15	CREA	0	1	OTU62 (Aggregatibacter)	0.016032775	0.84979807	0	#FF8E8A	0
29.5	14	CREA	0	1	OTU57 (Parvimonas)	0.079114849	0.349325989	0	#FF8E8A	0
30.5	13	CREA	0	1	OTU45 (Alloprevotella)	0.044067335	0.602551675	0	#FF8E8A	0
31.5	12	CREA	0	1	OTU67 (Alloprevotella)	-0.004036932	0.961970792	0	#00CCCC	0
32.5	11	CREA	0	1	OTU29 (Porphyromonas)	0.172853783	0.039677024	1	#FF8E8A	1
33.5	10	CREA	0	1	OTU365 (Fusobacterium)	0.108303386	0.199508185	0	#FF8E8A	1
34.5	9	CREA	0	1	OTU169 (Treponema)	0.176658279	0.035457913	1	#FF8E8A	1
35.5	8	CREA	0	1	OTU42 (g__Absconditabacteriales_)	-0.031576034	0.709119284	0	#00CCCC	0
36.5	7	CREA	0	1	OTU380 (Porphyromonas)	0.131062436	0.120017744	0	#FF8E8A	1
37.5	6	CREA	0	1	OTU678 (Neisseria)	0.011500479	0.891949963	0	#FF8E8A	0
38.5	5	CREA	0	1	OTU75 (Porphyromonas)	0.152395767	0.070211367	0	#FF8E8A	1
39.5	4	CREA	0	1	OTU134 (Prevotella)	-0.126346378	0.134056753	0	#00CCCC	1
40.5	3	CREA	0	1	OTU2 (Fusobacterium)	0.110284972	0.191358538	0	#FF8E8A	1
41.5	2	CREA	0	1	OTU523 (Prevotella)	-0.026785396	0.751683397	0	#00CCCC	0
42.5	1	CREA	0	1	OTU4 (Prevotella)	-0.036980331	0.662164972	0	#00CCCC	0

Supplementary method

Diagnostic, inclusion, and exclusion criteria

Diagnostic criteria are based on the "COVID-19 diagnosis and treatment program trial V.5 (or V.6) guidelines' issued by the National Health Commission of the People's Republic of China

1. Suspected cases.

Combined with the following comprehensive analysis of epidemiological history and clinical manifestations: any one of the epidemiological history and any two of the clinical manifestations, or no clear epidemiological history and three of the clinical manifestations can be diagnosed as a suspected patient.

(1) History of Epidemiology

1) Travel history or residence history of Wuhan city and surrounding areas, or other communities with case reports within 14 days before onset.

2) A history of contact with a infected person (positive nucleic acid test) within 14 days before onset.

3) Have contacted patients with fever or respiratory symptoms from Wuhan city and surrounding areas, or from communities with case reports within 14 days before onset.

4) Aggregated onset (2 or more cases of fever and / or respiratory symptoms occurred within 2 weeks in a small area such as home, office, school class, etc.,).

(2) Clinical manifestations

1) Fever and / or respiratory symptoms.

2) With the characteristic image of COVID-19.

3) The total number of white blood cells in the early stage of the disease is normal or decreased, and the lymphocyte count is normal or decreased.

2. Confirmed cases.

Suspected cases with one of the following etiological or serological evidence:

(1) Fluorescence RT-PCR detects the positive of the COVID-19 virus nucleic acid.

(2) Viral gene sequencing finds highly homologous to COVID-19 virus.

3. Cured patients (Convalescent patients).

Meet the following conditions at the same time:

(1) Body temperature returned to normal for more than 3 days.

(2) Respiratory symptoms improved significantly.

(3) Pulmonary imaging showed a significant improvement in acute exudative lesions.

(4) Two consecutive sputum, nasopharyngeal swabs and other respiratory tract specimens with negative nucleic acid test (sampling time needs to be at least 24 hours apart).

All enrolled individuals were checked by a professional dentist to ensure healthy mouth and gums. The inclusion criteria for healthy volunteers referred to our previous study. The exclusion criteria included diabetes, obesity, hypertension, metabolic syndrome, irritable bowel syndrome, nonalcoholic fatty liver disease, coeliac disease, and liver cirrhosis. Individuals who received antibiotics and/or probiotics within 8 weeks before enrolment were also excluded.

Enrollment process

We set two isolation wards in hospital, suspected cases ward and confirmed cases ward. Suspected case ward was used for admitting patients with fever, cough, or other related symptoms, or clinical confirmed COVID-19. On the second day of admission, a professional staff will collect the throat swab specimens for nucleic acid test by PT-PCR. If the nucleic acid detection was positive, patients were immediately transferred to confirmed cases ward. Our investigator would then assess whether the patient meets our enrollment criteria. If so, the patient will be included after signing the informed consent. Patients with exposure to confirmed patients or Wuhan, but without symptoms, were isolated outside the hospital and collected throat swab specimens for nucleic acid detection. If the result of nucleic acid test was positive, patients were admitted to hospital and transferred to confirmed cases ward. Then our

investigator screened and enrolled eligible patients.

Convalescent patients were transferred to the isolation area outside the hospital and observed for 15 days. If the nucleic acid test was still negative during this period, convalescent patients could departure from the isolated region. Our investigator would collect samples from them two days before discharge.

PCR amplification

We used primers targeting the hypervariable V3-V4 region of the 16S rRNA gene to amplify the extracted DNA samples. The forward primer was 5’-ACTCCTACGGGAGGCAGCA-3’ and the reverse primer was 5’-GGACTACHVGGGTWTCTAAT-3’. The PCR reaction system including 0.4 μL of Fast Pfu polymerase, 2 μL of 2.5 mM dNTP (TransGen Biotech, Beijing, China), 4 μL of 5×Fast Pfu buffer, 0.8 μL of each primer (5 μM), and 10 ng of template DNA. Each sample were carried out four reactions using a PCR machine (ABI GeneAmp 9700): 95 °C for 2 minutes, 95 °C for 30 s, 55 °C for 30 s, 72 °C for 30 s, repeating 30 cycles and finally at 72 °C for 5 minutes. Agarose gel (Axygen Biosciences, Union City, CA) was used to separate, extract and purify the PCR products, and the products were quantified using a fluorescence assay kit (Quant-iT PicoGreen, Invitrogen).

Sequence data process

We distributed the filtered readings based on the sample-specific barcode to different samples, and then trimmed the barcode and primers. Process the amplified readings through the following steps: (i) overlap pair end sequenced reads of each library using FLASH v1.2.10 software. (ii) use the customization of each program to perform more specific quality control on overlapping reads generated by FLASH 1) Ambiguous bases (N); 2) the mismatch rate in the overlap region was no more than 0.05; 3) mismatches in barcode and primer regions were not approved; (iii) de-multiplexed and assigned reads into different samples according to barcodes; (iv) detect and remove the chimeric sequences by UCHIME version 4.2.40 (version microbiome util-r20110519, http://drive5.com/uchime/gold.fa) to match Operational Taxonomy

Units (OTUs).

Construction of probability of disease (POD)

We used the abundance profile of the optimal OTUs markers in the discovery cohort to conduct fivefold cross-validation on a random forest model (R 3.4.1, randomForest 4.6-12 package). Then we acquired the cross-validation error curve through five trials of the fivefold cross-validation. The point with the minimum cross-validation error was defined as the cut-off point through the minimum error plus the standard deviation (SD). The sets of OTU markers with the error less than the cut-off value were listed and the set with the smallest number of OTUs were defined as the optimal set. We used the optimal set of OTUs to calculate POD index in the discovery and validation cohort. The constructed models were assessed using the receiver operating characteristic (ROC) curve (R 3.3.0, pROC package), and the ROC effect was showed using AUC.
